# African swine fever virus pM448R protein promotes STUB1-mediated ubiquitin-proteasome degradation of IRF1 to attenuate type III interferon induction

**DOI:** 10.1128/jvi.00580-26

**Published:** 2026-06-09

**Authors:** Jinyu Zhao, Jiaxu Xie, Yuxin Shi, Chunxiao Mou, Zhenhai Chen

**Affiliations:** 1 College of Veterinary Medicine, Yangzhou University 38043, Yangzhou, China; 2 Jiangsu Interdisciplinary Center for Zoonoses and Biosafety, Yangzhou University 38043, Yangzhou, China; 3 Jiangsu Co-Innovation Center for Prevention and Control of Important Animal Infectious Diseases and Zoonoses, Yangzhou University 38043, Yangzhou, China; 4 Jiangsu Key Laboratory of Zoonosis, Yangzhou University 38043, Yangzhou, China; University of Minnesota Twin Cities, Minneapolis, Minnesota, USA

**Keywords:** African swine fever virus, pM448R, IRF1, IFN-λ, STUB1

## Abstract

African swine fever virus (ASFV) primarily invades through the respiratory and intestinal mucosa, where type III interferons (IFN-λ) play a pivotal role in local immunity. However, whether ASFV counteracts this IFN-λ-mediated antiviral defense remains largely unknown. Here, we demonstrate that ASFV infection dampens IFN-λ production, where the viral protein pM448R serves as a suppressor. Mechanistically, pM448R targets interferon regulatory factor 1 (IRF1) for proteasomal degradation by recruiting the E3 ubiquitin ligase STIP1 homology and U box-containing protein 1 (STUB1), which catalyzes K48-linked ubiquitination of IRF1. Moreover, infection with the M448R-deficient ASFV mutant leads to increased IRF1 protein stability and elevated expression of IRF1 target genes, including OAS1, OAS2, and ZBP1. Our findings reveal a novel immune evasion strategy whereby ASFV hijacks the host ubiquitin-proteasome system to degrade IRF1, thereby subverting IRF1-dependent transcriptional programs, including IFN-λ induction, to facilitate mucosal invasion.

**IMPORTANCE:**

African swine fever virus (ASFV) remains a major threat to the global swine industry. However, how the virus evades mucosal innate immunity at its portal of entry remains poorly understood. The transcription factor IRF1 serves as a central hub in mucosal antiviral defense, coordinating the expression of IFN-λ and numerous other restriction factors. In this study, we identify pM448R as a viral antagonist that directs IRF1 for ubiquitin-mediated degradation, which reveals a novel mechanism by which ASFV destroys the mucosal immune barrier. These findings not only deepen our understanding of viral interference with IRF1-dependent defense pathways, but also provide potential insights for the development of live-attenuated vaccines.

## INTRODUCTION

African swine fever (ASF) is an acute infectious disease affecting both domestic and wild pigs, with a fatality rate reaching 100% (1–3). Since its first report in 1921, ASF has become widespread across multiple global regions, with devastating consequences for pig populations and the agricultural economy (4–6). Given the severe threats posed by ASF, the development of an effective vaccine has become one of the most urgent tasks (7–10). ASF virus (ASFV) is a DNA virus with a low genomic mutation rate. However, it exhibits considerable genetic and antigenic diversity. It can be classified into 23 genotypes based on its B646L gene sequence, among which genotype XVIII was later characterized as a recombinant of genotypes I and VIII, and genotypes I and II are the current predominant strains in Europe and Asia (11). The recent emergence of I/II recombinant strains in countries like China and Vietnam, which have been shown to abolish the efficacy of existing vaccine candidates (12, 13)**,** poses a significant challenge to vaccine development. Therefore, advancing our understanding of ASFV pathogenesis through in-depth functional studies of viral proteins is imperative for paving the way for effective vaccines.

Studies have shown that virulent strains of ASFV achieve immune evasion and efficient replication by encoding multiple viral proteins that systematically suppress the induction and signaling of host interferons (IFNs). The functions of these viral proteins nearly encompass the entire pathway from IFN production to its effector functions. For instance, at the production stage, type I IFN synthesis is blocked by proteins like DP71L and pI215L through inhibition of the cGAS-STING pathway and interferon regulatory factor 3 (IRF3) (14, 15). At the effector stage, proteins such as pB318L hinder ISG activation by directly targeting core JAK-STAT signaling molecules, including STAT1 and STAT2 (16).

In contrast to the extensively studied type I IFNs, IFN-λ plays an indispensable role in antiviral defense at mucosal sites, particularly in gastrointestinal and respiratory infections. Their receptor distribution is tissue-specific, with high expression primarily on mucosal epithelial cells. This allows IFN-λ to exert potent and localized antiviral effects at the viral entry “gateways,” while potentially avoiding the side effects associated with systemic inflammatory responses (17). The transcription factor interferon regulatory factor 1 (IRF1) serves as a central regulator of IFN-λ induction and broadly coordinates the expression of numerous antiviral restriction factors at mucosal barriers (18). A recent study has revealed that after administering a prophylactic “cocktail” of porcine-derived IFN-λ3, IFN-α2, and IFN-γ formulated as a fusion protein to ASFV-infected piglets, this treatment significantly induced the expression of various ISGs (including Mx1, OASL, and ISG12), effectively reduced viral load, delayed mortality, and alleviated tissue pathology (19). This suggests that mucosal immunity plays a critical role during the initial stages of ASFV infection. However, current research on ASFV in relation to mucosal immunity remains very limited. Significant knowledge gaps remain regarding the interaction between ASFV and IFN-λ. Most existing research on ASFV immune evasion has focused on the type I IFN pathway. It remains unclear whether and how ASFV specifically antagonizes the induction and signal transduction of IFN-λ.

In the study, we demonstrate for the first time that in poly(I:C)-treated or ASFV-infected PAMs, the previously uncharacterized ASFV protein pM448R targets IRF1 for ubiquitination and subsequent proteasomal degradation, thereby attenuating IRF1-dependent antiviral gene expression, including that of IFN-λ. This finding provides the first insight into the immune evasion mechanism employed by ASFV to modulate host mucosal immunity.

## RESULTS

### ASFV infection suppresses the production of type III interferon in primary porcine alveolar macrophages (PAMs)

**Fig 1:**
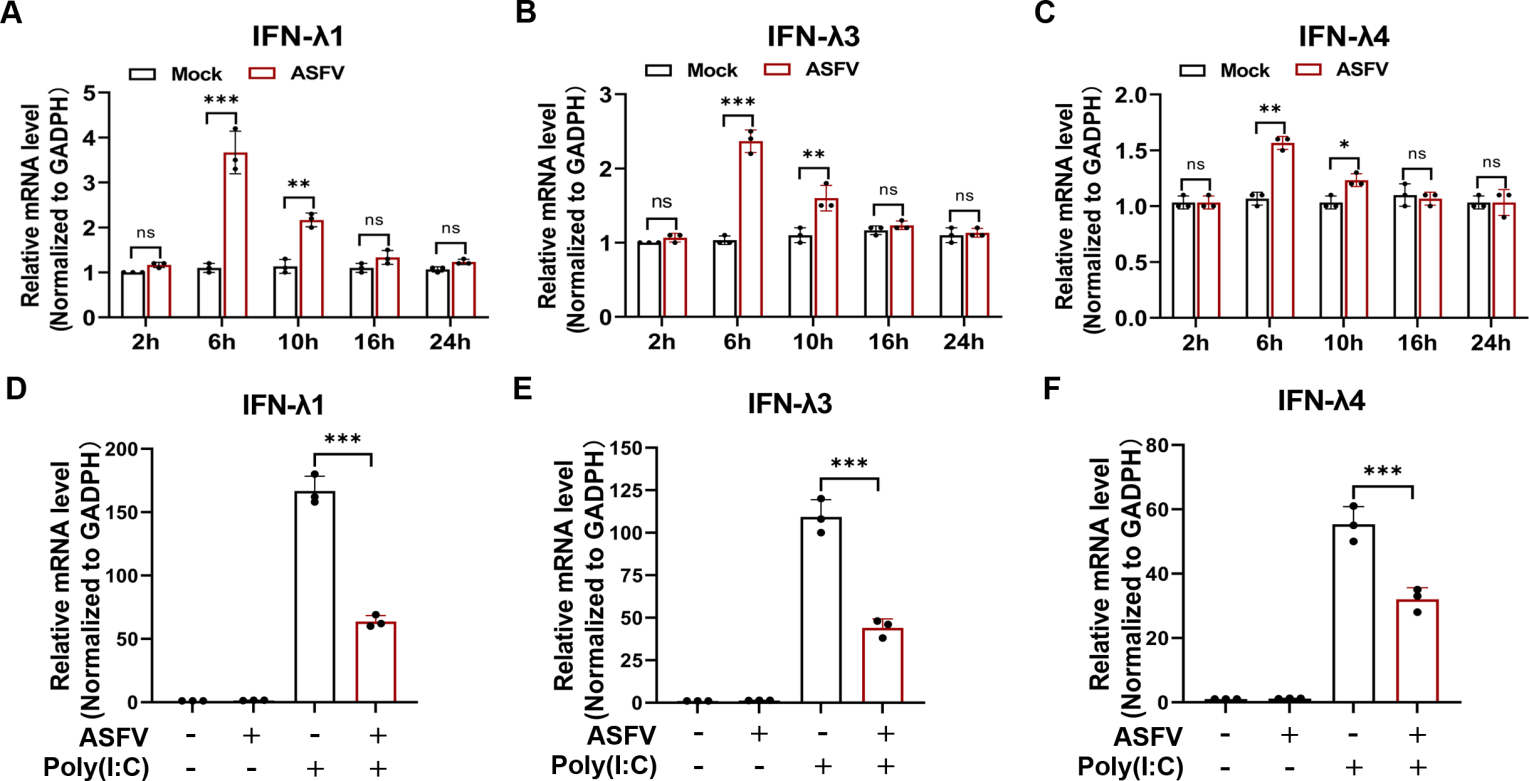
ASFV inhibits the production of type III interferon. (**A–C**) Time course of IFN-λ mRNA expression in ASFV-infected PAMs. PAMs were infected with ASFV (MOI = 1) and harvested at 2, 6, 10, 16, and 24 hpi. The mRNA levels of IFN-λ1, IFN-λ3, and IFN-λ4 were quantified by qRT-PCR. (**D–F**) ASFV suppresses poly(I:C)-induced IFN-λ expression. PAMs were infected with ASFV (MOI = 1) for 16 h. Cells were then either mock-treated or transfected with poly(I:C) (1 μg/mL) for 12 h. The mRNA levels of IFN-λ1, IFN-λ3, and IFN-λ4 were determined by qRT-PCR. Three independent experiments were performed with three technical replicates. * , *P* < 0.05; **, *P* < 0.01; ***, *P* < 0.001; ns, not significant.

The interferon response serves as the first line of defense against pathogen invasion. While previous studies have shown that ASFV encodes multiple proteins that regulate type I and type II interferon signaling pathways, its ability to regulate the IFN-λ response remains unclear (20). To investigate whether IFN-λ production can be induced during ASFV infection, we initially analyzed the transcription kinetics of IFN-λ genes (IFN-λ1, IFN-λ3, and IFN-λ4) in PAMs infected with ASFV at designated time points using qPCR. The results showed that ASFV infection induced low-level transcription of IFN-λ genes at early stages of infection (Fig. 1A through C). We further examined whether ASFV affects the production of IFN-λ. Notably, the results showed that ASFV infection significantly suppressed poly(I:C)-induced IFN-λ production (Fig. 1D through F). In summary, these findings demonstrate that ASFV can inhibit IFN-λ production in PAMs.

### ASFV pM448R suppresses the production of type III interferon

**Fig 2:**
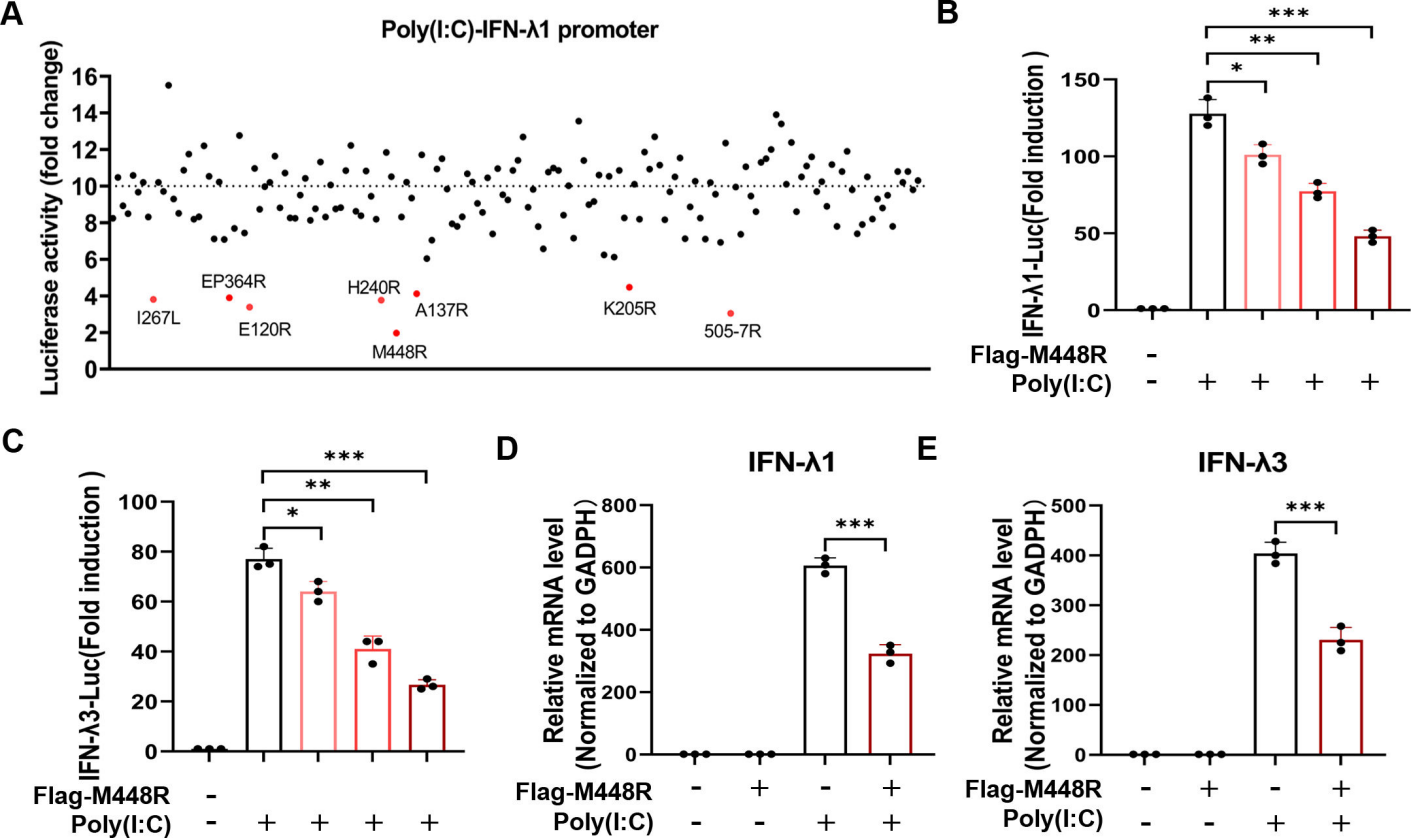
ASFV pM448R antagonizing type III interferon production. (**A**) Screening ASFV proteins which antagonize type III interferon production. IPEC-J2 cells were co-transfected with plasmids expressing individual ASFV proteins, along with the IFN-λ1 promoter-driven firefly luciferase reporter and the pRL-TK Renilla luciferase control plasmids. At 24 h post-transfection, the cells were transfected with poly(I:C) (1 μg/mL) for 12 h. Promoter activity of IFN-λ1 was analyzed by a dual-luciferase reporter assay. (**B and C**) ASFV pM448R dose-dependently inhibits IFN-λ1 and IFN-λ3 promoter activity. IPEC-J2 cells were co-transfected with increasing doses (0, 0.25, 0.5, and 1 μg) of the Flag-M448R expression plasmid, along with the IFN-λ1 or IFN-λ3 promoter reporter and the pRL-TK control plasmid. At 24 h post-transfection, the cells were either mock-treated or transfected with poly(I:C) (1 μg/mL) for 12 h. Promoter activity was analyzed by a dual-luciferase reporter assay. (**D and E**) ASFV pM448R inhibits the transcription of IFN-λ1 and IFN-λ3. IPEC-J2 cells were transfected with a Flag-M448R or an empty vector control plasmid. At 24 h post-transfection, the cells were either mock-treated or transfected with poly(I:C) (1 μg/mL) for 12 h, and then the mRNA levels of IFN-λ1 and IFN-λ3 were determined by qRT-PCR. For dual-luciferase reporter gene assays and qPCR analysis, three independent experiments were performed with three technical replicates. * , *P* < 0.05; **, *P* < 0.01; ***, *P* < 0.001.

Given that ASFV suppresses IFN-λ production, we hypothesized that specific viral proteins might mediate this effect. To identify such factors, we performed a dual-luciferase reporter screen of all 160 ASFV-encoded proteins. The results showed that eight viral proteins (pI267L, pEP364R, pE120R, pH240R, pM448R, pA137R, pK205R, and pMGF505-7R) were identified that significantly suppressed IFN-λ1 promoter activity. Among them, pM448R exhibited the strongest inhibitory effect (Fig. 2A). To validate this finding, we detected the effect of pM448R on both IFN-λ1 and IFN-λ3 promoters in a dose-dependent assay. The results demonstrated that the suppression of promoter activity was enhanced with increasing amounts of pM448R (Fig. 2B and C). Furthermore, qPCR analysis confirmed that pM448R significantly inhibited poly(I:C)-induced transcription of endogenous IFN-λ1 and IFN-λ3 (Fig. 2D and E). Taken together, these findings demonstrate that ASFV pM448R acts as a potent inhibitor of IFN-λ production.

### pM448R inhibits type III interferon production by targeting IRF1

**Fig 3:**
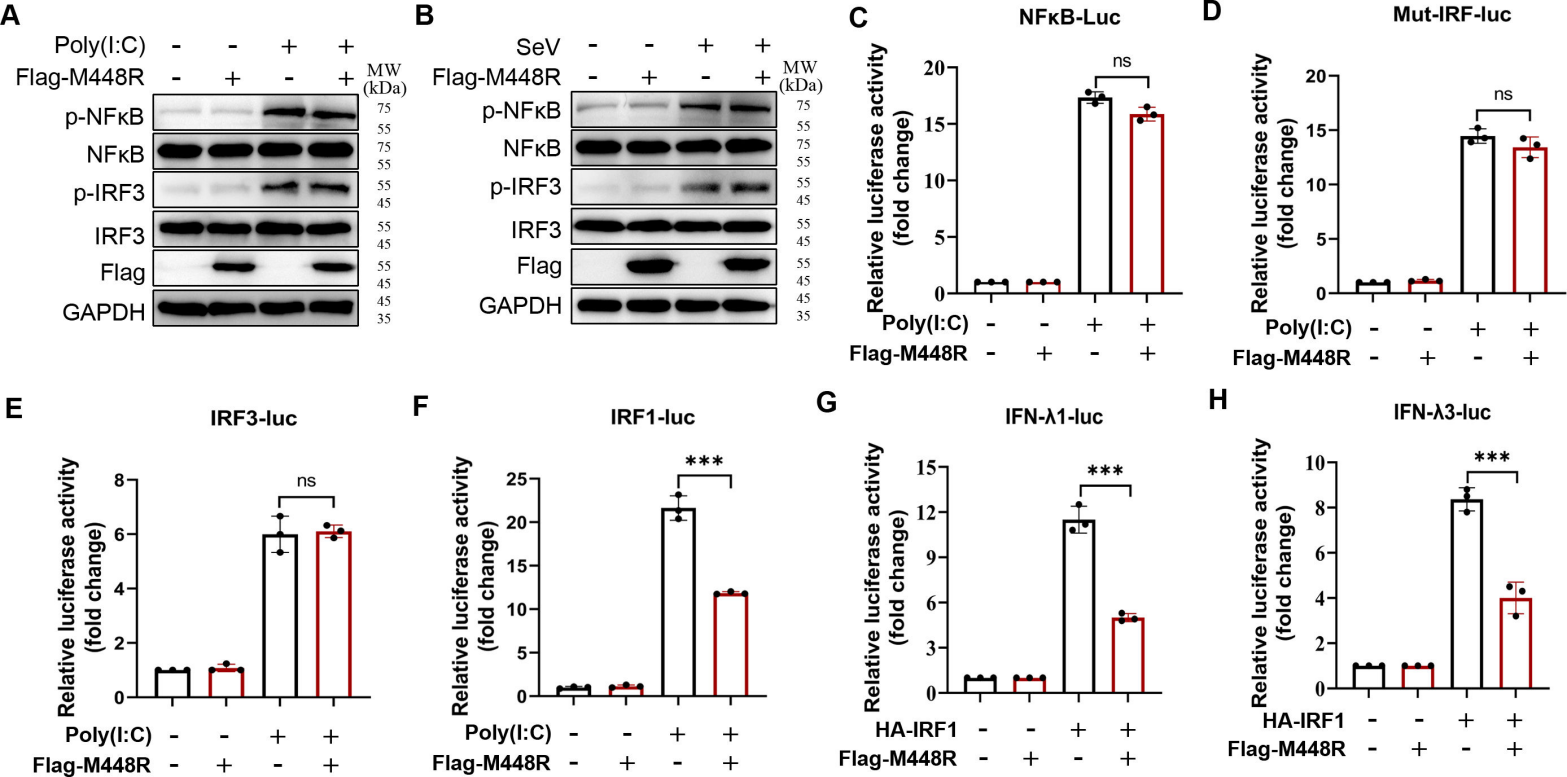
pM448R inhibits the production of type III interferons by targeting the IRF1 signaling pathway. (**A and B**) Effect of pM448R on the phosphorylation of IRF3 and NF-κB. IPEC-J2 cells were transfected with a Flag-M448R or a control plasmid. At 24 h post-transfection, the cells were stimulated with either poly(I:C) (1 μg/mL) for 1 h or SeV (MOI = 0.1) for 2 h. Whole-cell lysates were then subjected to western blot analysis using the indicated antibodies. (**C–H**) Effect of pM448R on IRF3 and NF-κB activation. IPEC-J2 cells were co-transfected with the Flag-M448R plasmid (or empty vector control), a firefly luciferase reporter plasmid (driven by the IFN-λ1, IFN-λ3, Mut-IRF, IRF3, IRF1, or NF-κB promoter), and the control plasmid. At 24 h post-transfection, the cells were either mock-treated or transfected with poly(I:C) (1 μg/mL) for 12 h. The activation of the respective promoters was analyzed using a dual-luciferase assay. For dual-luciferase reporter gene assays, three independent experiments were performed with three technical replicates. * , *P* < 0.05; **, *P* < 0.01; ***, *P* < 0.001; ns, not significant.

Current studies have indicated that the production of IFN-λ is co-regulated by multiple transcription factors, including IRF3, NF-κB, and IRF1 (21). To further identify the specific target of pM448R in inhibiting IFN-λ production, we first examined its effects on the IRF3 and NF-κB signaling pathways upon poly(I:C) or SeV stimulation. The results demonstrated that pM448R did not affect the activation of IRF3 or NF-κB induced by either stimulus (Fig. 3A and B). Subsequently, we evaluated the impact of pM448R on the promoter activities of NF-κB and IRFs by using a dual-luciferase reporter assay. Notably, we found that pM448R specifically inhibited the transcriptional activity of the IRF1 promoter-driven luciferase reporter (Fig. 3C through F). We further examined the effect of pM448R on IFN-λ1 and IFN-λ3 promoter activities under IRF1-overexpressing conditions. The results showed that pM448R significantly suppressed the promoter activities of both IFN-λ1 and IFN-λ3 mediated by IRF1 (Fig. 3G and H). These findings suggest that pM448R likely suppresses IFN-λ production by targeting IRF1.

### pM448R facilitates the proteasomal degradation of IRF1

**Fig 4:**
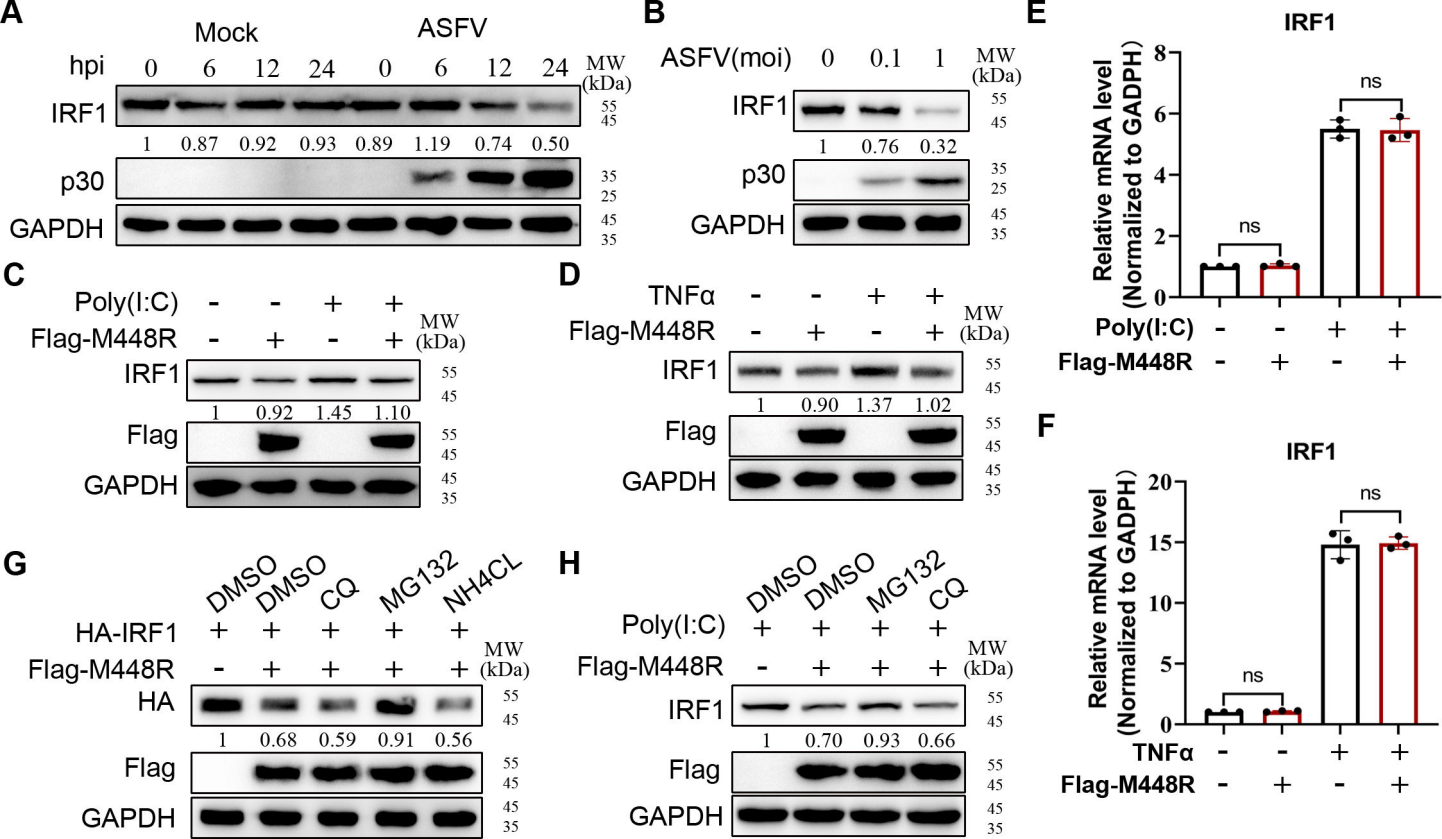
ASFV pM448R promotes the degradation of IRF1 via the proteasomal pathway. (**A and B**) ASFV infection inhibits IRF1 expression. (**A**) PAMs were infected with ASFV (MOI = 1) for 0, 6, 12, and 24 h. Cell lysates were then analyzed by western blotting with the indicated antibodies. (**B**) PAMs were infected with ASFV at MOIs of 0, 0.1, and 1 for 24 h, followed by western blot analysis. (**C and D**) pM448R inhibits IRF1 expression. IPEC-J2 cells were transfected with a Flag-M448R plasmid. At 24 h post-transfection, the cells were stimulated with poly(I:C) (1 μg/mL) for 12 h or with TNF-α for 2 h. Western blotting was then performed. (**E and F**) The effect of pM448R on IRF1 mRNA levels. IPEC-J2 cells were transfected with a Flag-M448R plasmid. At 24 h post-transfection, the cells were either transfected with poly(I:C) (1 μg/mL) for 12 h or treated with TNF-α for 2 h. The mRNA levels of IRF1 were then determined by qRT-PCR. Three independent experiments were performed with three technical replicates. (**G and H**) pM448R promotes the ubiquitination degradation of IRF1. (**G**) IPEC-J2 cells were co-transfected with Flag-M448R and HA-IRF1 plasmids. At 24 h post-transfection, the cells were treated with DMSO, MG132, CQ, or NH_4_Cl for 4 h. Cell lysates were analyzed by western blotting. (**H**) IPEC-J2 cells were transfected with Flag-M448R and treated with the same inhibitors as in panel G. Cell lysates were analyzed by western blotting. Densitometric analysis of the target bands was performed using ImageJ software, and the bands were normalized to GAPDH. ns, not significant.

Recent studies have demonstrated that multiple viruses can evade host immune responses by targeting IRF1. Thus, we further investigated whether ASFV could also regulate IRF1 expression. We first examined the protein levels of IRF1 in PAMs at different time points after ASFV infection. The results showed that ASFV infection could upregulate IRF1 expression in the early stage, but as the infection progressed, the protein levels of IRF1 gradually decreased. Additionally, IRF1 protein levels also tended to decrease with increasing MOI (Fig. 4A and B). The findings indicate that ASFV possesses a mechanism to regulate the protein level of IRF1. We then examined the effect of pM448R on IRF1 protein levels, and the results showed that pM448R suppressed the upregulation of IRF1 induced by poly(I:C) or TNF-α (Fig. 4C and D), without affecting IRF1 mRNA levels (Fig. 4E and F), suggesting that pM448R promotes IRF1 degradation. To elucidate the underlying mechanism, we treated cells with the proteasome inhibitor MG132 or lysosome inhibitors CQ/NH₄Cl. The results showed that MG132 markedly rescued pM448R-mediated IRF1 degradation (Fig. 4G and H), indicating that the regulation of IRF1 protein levels by pM448R is dependent on the ubiquitin-proteasome pathway. Collectively, these results demonstrate that ASFV pM448R promotes IRF1 degradation via the ubiquitin-proteasome system.

### pM448R interacts with IRF1 and induces its K48-linked ubiquitination

**Fig 5:**
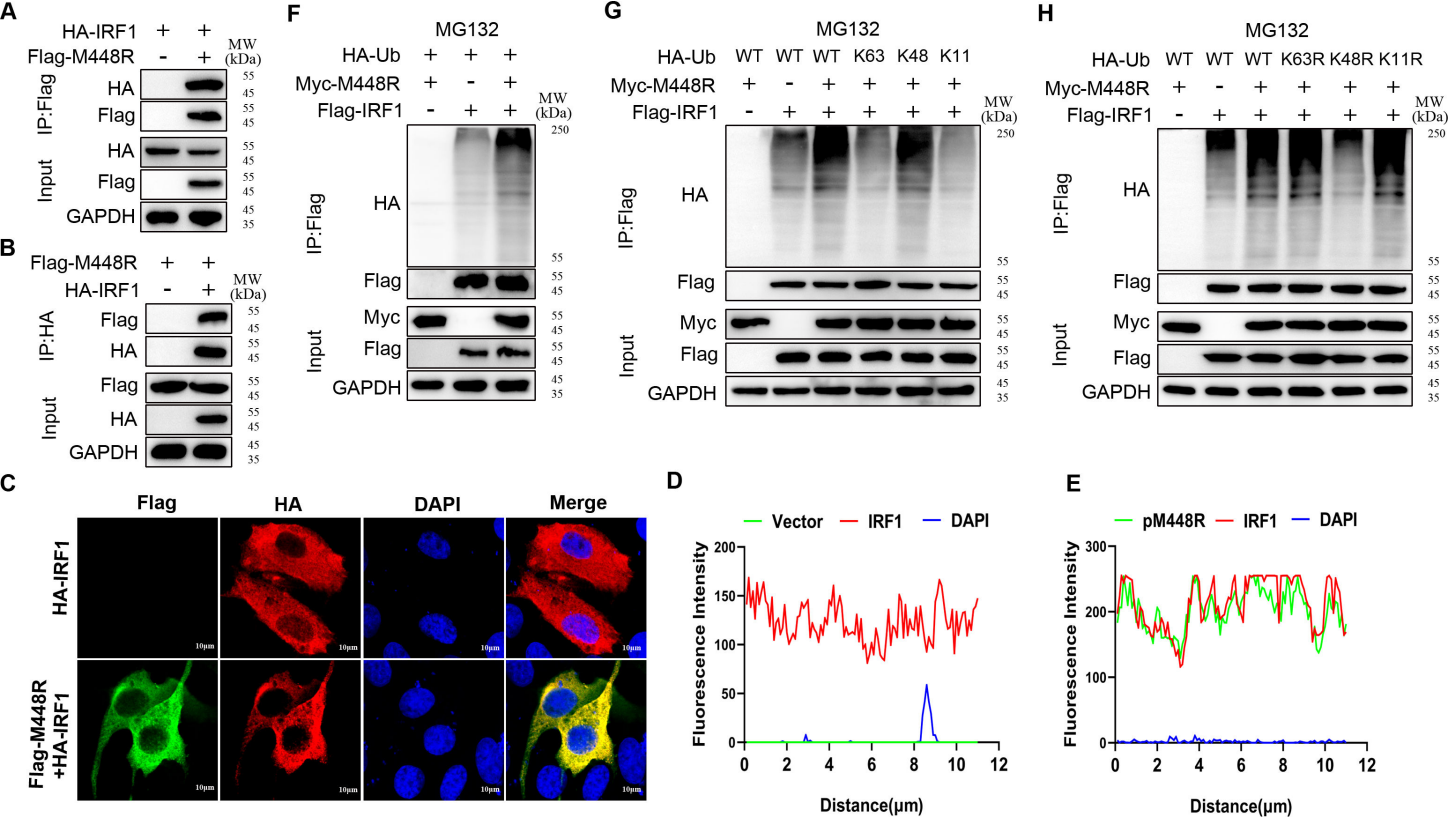
M448R interacts with IRF1 and promotes its K48-linked ubiquitination. (**A–E**) pM448R interacts with IRF1. (**A and B**) HEK293T cells were co-transfected with Flag-M448R and HA-IRF1 for 24 h. Cell lysates were subjected to Co-IP and western blot analysis with the indicated antibodies. (**C**) IPEC-J2 cells were co-transfected with Flag-M448R and HA-IRF1 for 24 h. Cells were fixed and subjected to immunofluorescence staining with mouse anti-Flag and rabbit anti-HA antibodies, followed by DAPI counterstaining. Images were captured by laser confocal microscopy. Scale bar, 10 μm. (**D and E**) Colocalization analysis of the region delineated by the white line in panel C was performed using ImageJ. (**F**) pM448R promotes the ubiquitination of IRF1. HEK293T cells were co-transfected with Myc-M448R, Flag-IRF1, and HA-Ub plasmids for 24 h, followed by treatment with MG132 for 4 h. IRF1 ubiquitination was analyzed by Co-IP and western blotting. (**G and H**) pM448R specifically promotes K48-linked ubiquitination of IRF1. HEK293T cells were co-transfected with Myc-M448R, Flag-IRF1, and either wild-type HA-tagged ubiquitin (HA-Ub) or the following HA-Ub mutants for 24 h. (**G**) lysine retention mutants (HA-Ub-K63-only, HA-Ub-K48-only, or HA-Ub-K11-only) and (**H**) lysine-to-arginine mutants (HA-Ub-K63R, HA-Ub-K48R, or HA-Ub-K11R). Cells were treated with MG132 for 4 h prior to Co-IP and western blot analysis.

To elucidate the mechanism by which pM448R regulates the ubiquitination-induced degradation of IRF1, we first examined whether there is an interaction between pM448R and IRF1. The Co-immunoprecipitation (Co-IP) assay confirmed the interaction between the two proteins, and in the meanwhile, cytoplasmic co-localization of pM448R and IRF1 was observed via confocal microscopy. (Fig. 5A through E). Furthermore, we investigated whether pM448R affects IRF1 ubiquitination. The Co-IP assays demonstrated that pM448R increased the ubiquitination levels of IRF1 (Fig. 5F). Subsequently, we further characterized the specific type of polyubiquitination conjugated to IRF1 induced by pM448R, and found that pM448R specifically promotes K48-linked polyubiquitination of IRF1 (Fig. 5G and H). Taken together, these results demonstrate that pM448R interacts with IRF1 to promote its K48-linked polyubiquitination.

### pM448R promotes the Stub1-mediated ubiquitination and subsequent degradation of IRF1

**Fig 6:**
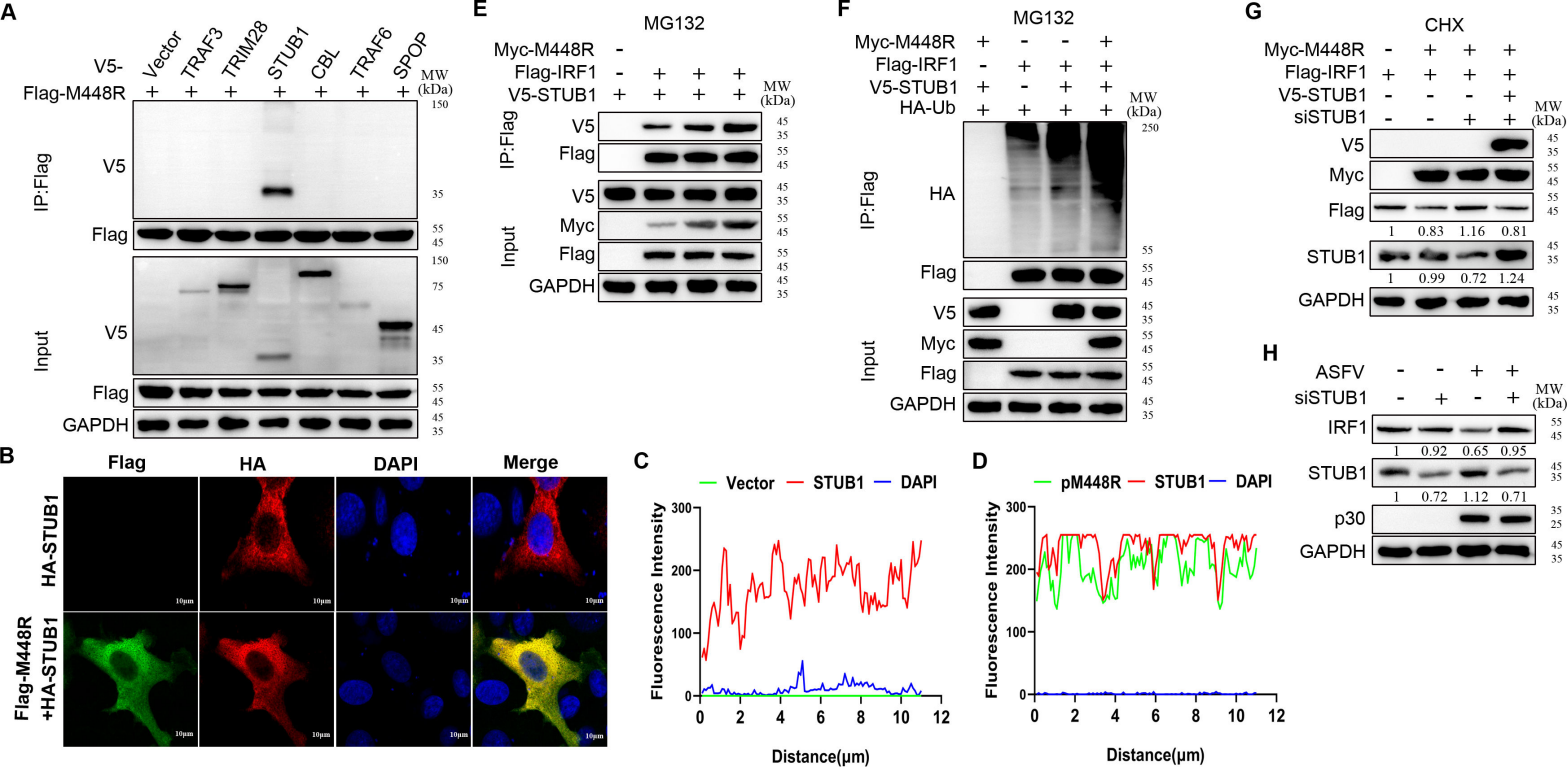
pM448R promotes STUB1-induced ubiquitination and degradation of IRF1. (**A and B**) pM448R interacts with STUB1. (**A**) HEK293T cells were transfected with the indicated plasmids for 24 h. Cell lysates were subjected to Co-IP and western blot analysis with the indicated antibodies. (**B**) IPEC-J2 cells were co-transfected with Flag-M448R and HA-STUB1 for 24 h. After fixation, the cells were subjected to immunofluorescence staining with mouse anti-Flag and rabbit anti-HA antibodies, followed by DAPI counterstaining. Images were acquired by laser confocal microscopy. Scale bar, 10 μm. (**C and D**) Co-localization analysis of the region delineated by the white line in panel B was performed using ImageJ. (**E**) pM448R promotes the interaction between STUB1 and IRF1. HEK293T cells were co-transfected with Flag-IRF1, V5-STUB1, and increasing doses of Myc-M448R. At 24 h post-transfection, the cells were treated with MG132 for 4 h. The STUB1-IRF1 interaction was analyzed by Co-IP and western blotting. (**F**) pM448R promotes STUB1-mediated ubiquitination of IRF1. HEK293T cells were co-transfected with Flag-IRF1, V5-STUB1, Myc-M448R, and HA-Ub. At 24 h post-transfection, the cells were treated with MG132 for 4 h. Ubiquitination of IRF1 was assessed by Co-IP and western blotting. (**G and H**) Knockdown of STUB1 inhibits M448R-induced IRF1 degradation. (**G**) IPEC-J2 cells were transfected with STUB1-specific siRNA. After 24 h, the cells were co-transfected with Myc-M448R, Flag-IRF1, and V5-STUB1, then treated with cycloheximide (CHX, 25 μg/mL) for 1 h, followed by western blot analysis. (**H**) PAMs were transfected with STUB1-specific siRNA for 24 h. Then, the cells were infected with ASFV (MOI = 1) for another 24 h and protein levels of IRF1 were analyzed by western blotting. Densitometric analysis of the target bands was performed using ImageJ software, and the bands were normalized to GAPDH.

The ubiquitination process requires the coordinated action of E1 ubiquitin-activating enzymes, E2 ubiquitin-conjugating enzymes, and E3 ubiquitin ligases (22). Among these, E3 ubiquitin ligases are responsible for specific substrate recognition and catalyzing ubiquitin modification. Previous studies have shown that IRF1 ubiquitination is regulated by multiple E3 ligases, including TRAF3, TRIM28, STUB1, CBL, TRAF6, and SPOP (23–27). To determine whether pM448R regulates IRF1 ubiquitination by recruiting E3 ligases, we screened a series of E3 ligases using Co-IP. The results showed that pM448R interacts with STUB1 (Fig. 6A). Confocal microscopy further confirmed co-localization of pM448R and STUB1 in the cytoplasm (Fig. 6B through D). Subsequent dose-dependent experiments revealed that pM448R enhances the interaction between STUB1 and IRF1 (Fig. 6E). Additionally, Co-IP assays showed that pM448R promotes STUB1-mediated ubiquitination of IRF1 (Fig. 6F). To validate whether pM448R-induced IRF1 degradation depends on STUB1, we knocked down STUB1 using siRNA and found that this treatment suppressed IRF1 degradation induced by pM448R. Conversely, exogenous reintroduction of STUB1 restored the ability of pM448R to promote IRF1 degradation (Fig. 6G). Furthermore, a similar effect was observed upon STUB1 knockdown in ASFV-infected PAMs (Fig. 6H). Collectively, these results indicate that pM448R mediates IRF1 ubiquitination and subsequent degradation by recruiting STUB1.

### ASFV-ΔM448R promotes expression of IRF1 target genes

**Fig 7:**
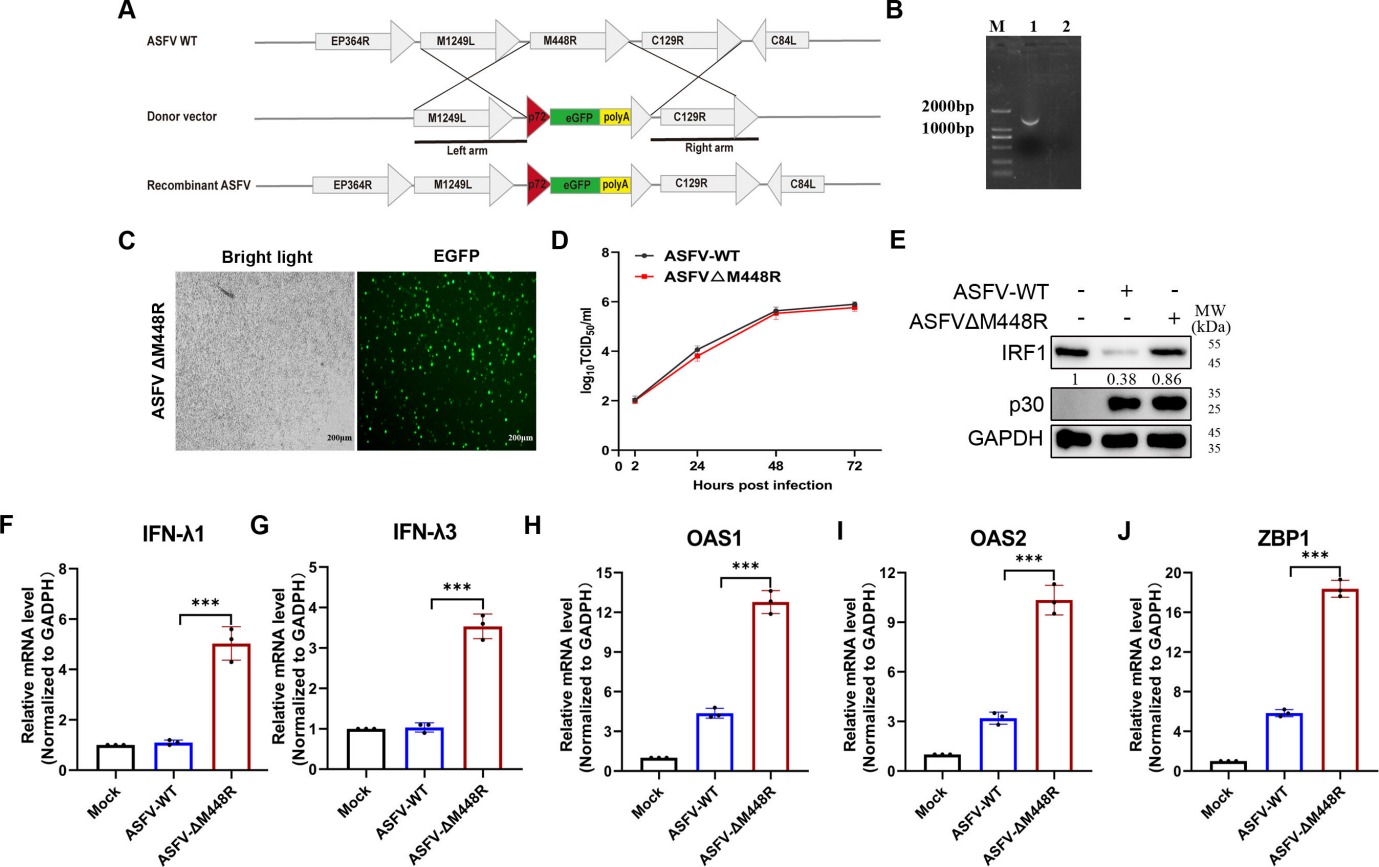
ASFV-ΔM448R promotes expression of IRF1 target genes. (**A–C**) Construction of ASFVΔM448R. (**A**) The ASFVΔM448R strain was generated via homologous recombination, as detailed in the Materials and Methods. (**B**) The M448R gene fragment was amplified by PCR from both the parental ASFV and the ASFV-ΔM448R genomes, and the products were analyzed by electrophoresis. (**C**) Growth of ASFV-ΔM448R in PAMs. (**D**) Replication kinetics of ASFV-ΔM448R. PAMs were infected with either ASFV-ΔM448R or the parental virus (MOI = 0.1). Viral titers in the culture supernatants at the indicated time points were determined by the TCID_50_ method. (**E**) ASFV-ΔM448R does not affect IRF1 expression. PAMs were infected with ASFV-WT or ASFV-ΔM448R (MOI = 1) for 24 h, and IRF1 protein levels were analyzed by western blotting. Densitometric analysis of the target bands was performed using ImageJ software, and the bands were normalized to GAPDH. (**F–J**) ASFV-ΔM448R induces enhanced transcription of IRF1 target genes. PAMs were infected with ASFV-WT or ASFV-ΔM448R (MOI = 1) for 24 h, and the mRNA levels of IFN-λ1, IFN-λ3, OAS1, OAS2, and ZBP1 were quantified by qRT-PCR. Three independent experiments were performed with three technical replicates each.* , *P* < 0.05; **, *P* < 0.01; ***, *P* < 0.001.

To further investigate whether pM448R modulates host antiviral responses during ASFV infection, we constructed an ASFV M448R deletion mutant (ASFV-ΔM448R) using homologous recombination (Fig. 7A). After multiple rounds of purification, the mutant was verified by PCR (Fig. 7B), and green fluorescence was observed in infected PAMs at 16 h post-infection (Fig. 7C). Growth curve analysis in PAMs revealed no significant difference between ASFV-ΔM448R and the parental virus (Fig. 7D). Additionally, we performed whole-genome sequencing of ASFV-ΔM448R to confirm that no additional phenotype-altering mutations were introduced during virus purification (Fig. S1; the genome sequence is provided in the supplemental text). We next examined IRF1 protein levels in infected cells. Western blot analysis showed that IRF1 protein abundance was markedly higher in ASFV-ΔM448R-infected PAMs than in wild-type virus-infected cells (Fig. 7E). Furthermore, qPCR analysis revealed that infection with ASFV-ΔM448R resulted in elevated mRNA levels of the IRF1 target genes OAS1, OAS2, and ZBP1 (Fig. 7H through J), and a similar trend was observed for IFN-λ1 and IFN-λ3 transcripts (Fig. 7F and G). Collectively, these results demonstrate that pM448R suppresses IRF1-dependent antiviral gene expression during ASFV infection by reducing IRF1 protein stability.

## DISCUSSION

Since its discovery in 2003, IFN-λ has been recognized as a critical regulator of mucosal immunity (28, 29). Similar to type I interferons, IFN-λ expression is controlled by pattern recognition receptor (PRR) signaling pathways and induced upon PRR activation. IFN-λ primarily establishes a highly localized, frontline antiviral defense at mucosal epithelial surfaces (30). However, whether ASFV can evade this defense remains unclear. In this study, we observed a dynamic pattern of IFN-λ expression during ASFV infection: a low-level transcription is induced at the early stage of infection, while its expression is significantly suppressed at the later stage. To elucidate the underlying immune evasion mechanism, we systematically screened ASFV-encoded proteins and identified a previously uncharacterized protein, pM448R, that potently inhibits IFN-λ production. Given that IFN-λ expression is finely regulated by transcription factors such as IRF1, IRF3, and NF-κB, we further investigated the specific pathway targeted by pM448R and found that its suppression of IFN-λ promoter is independent of the IRF3 and NF-κB pathways. Crucially, mutations in the IRF promoter sequence abolished the inhibitory effect, indicating that IRF1 is the key host factor targeted by pM448R.

IRF1 is not only a critical transcription factor responsible for the expression of IFN-λ, but is also involved in the induction of type I interferon (31). Its expression is tightly regulated by multiple signaling pathways. Notably, IRF1 is an interferon-stimulated gene whose expression is further amplified through a positive feedback loop mediated by interferons (32, 33). Additionally, both poly(I:C) and TNF-α can robustly induce IRF1 expression (34). In this study, we observed that ASFV infection triggered an early induction of IRF1, which was subsequently downregulated as infection progressed, a pattern consistent with the transcriptional dynamics of IFN-λ. This biphasic regulation suggests active viral manipulation, especially in light of recent evidence identifying IRF1 as a common target for viral immune evasion. For example, SADS nsp1 inhibits IRF1 nuclear translocation (35), CSFV Npro suppresses IRF1 expression (36), and HIV-1 Tat promotes IRF1 ubiquitination and degradation (37). We therefore hypothesized that the early upregulation of IRF1 during ASFV infection may result from the activation of PRR signaling pathways by viral PAMPs, whereas the subsequent decrease is likely associated with the action of viral immunomodulatory proteins.

Given that IRF1 was identified as a potential target for pM448R-mediated suppression of IFN-λ production, we showed that pM448R promotes IRF1 degradation via the ubiquitin-proteasome pathway. It is well-established that protein ubiquitination depends on E3 ubiquitin ligases. Studies have shown that the ubiquitination of IRF1 can be regulated by several E3 ligases. In our study, we found that pM448R specifically interacts with STUB1 and enhances STUB1-mediated K48-linked ubiquitination of IRF1. Additionally, infection with ASFV-ΔM448R led to increased IRF1 protein stability compared to the parental strain, and this was accompanied by elevated mRNA levels of well-characterized IRF1 target genes, including OAS1, OAS2, and ZBP1. These findings indicate that deletion of M448R relieves the suppression of IRF1-dependent transcriptional activity, leading to increased expression of both IFN-λ and other IRF1-driven antiviral effectors.

M448R is a highly conserved gene of unknown function in ASFV. In the study, we demonstrate for the first time that its encoded protein, pM448R, inhibits the production of IFN-λ. However, deletion of the M448R gene did not impair viral replication in PAMs. This discrepancy may be explained by the fact that macrophages are neither the primary source of IFN-λ nor do they express high levels of its receptor. Furthermore, direct validation of whether M448R promotes epithelial infection via IFN-λ suppression remains unfeasible due to the lack of epithelial cell lines that support efficient ASFV replication. Notably, pM448R shares structural homology with members of the T4 RNA ligase 1 (Rnl1) family and other members of the ATP-dependent nucleotidyltransferase superfamily, including RNA capping enzymes (data not shown). This superfamily is characterized by a conserved active site architecture that typically mediates covalent nucleotidyl transfer reactions (38). Based on this, it can be inferred that, in addition to its role in targeting IRF1, pM448R may also possess intrinsic RNA ligase or capping activity, which could interfere with the accumulation or recognition of immunostimulatory RNA species during ASFV infection. For example, rotavirus capping enzyme VP3, a multifunctional protein belonging to the same nucleotidyltransferase superfamily, has been shown not only to modify viral RNA through its capping activity but also to directly target and degrade MAVS, thereby suppressing interferon production (39). Similarly, ASFV pM448R may also act as a multifunctional virulence factor. However, direct biochemical and structural studies are required to confirm whether pM448R indeed possesses RNA ligase activity.

It should be noted that, although M448R strongly inhibited IFN-λ promoter activity and IRF1-dependent transcription in overexpression assays, its effect was more modest under endogenous conditions of ASFV infection. Compared with the wild-type virus, PAMs infected with the M448R-deletion virus exhibited only a mild increase in IFN-λ mRNA levels, and no significant difference in viral replication was observed. This result is likely because ASFV encodes multiple proteins targeting the interferon pathway, and functional redundancy may compensate for the loss of M448R. Future studies using *in vivo* infection models are warranted to further evaluate the actual contribution of M448R to viral pathogenicity and interferon evasion.

**Fig 8:**
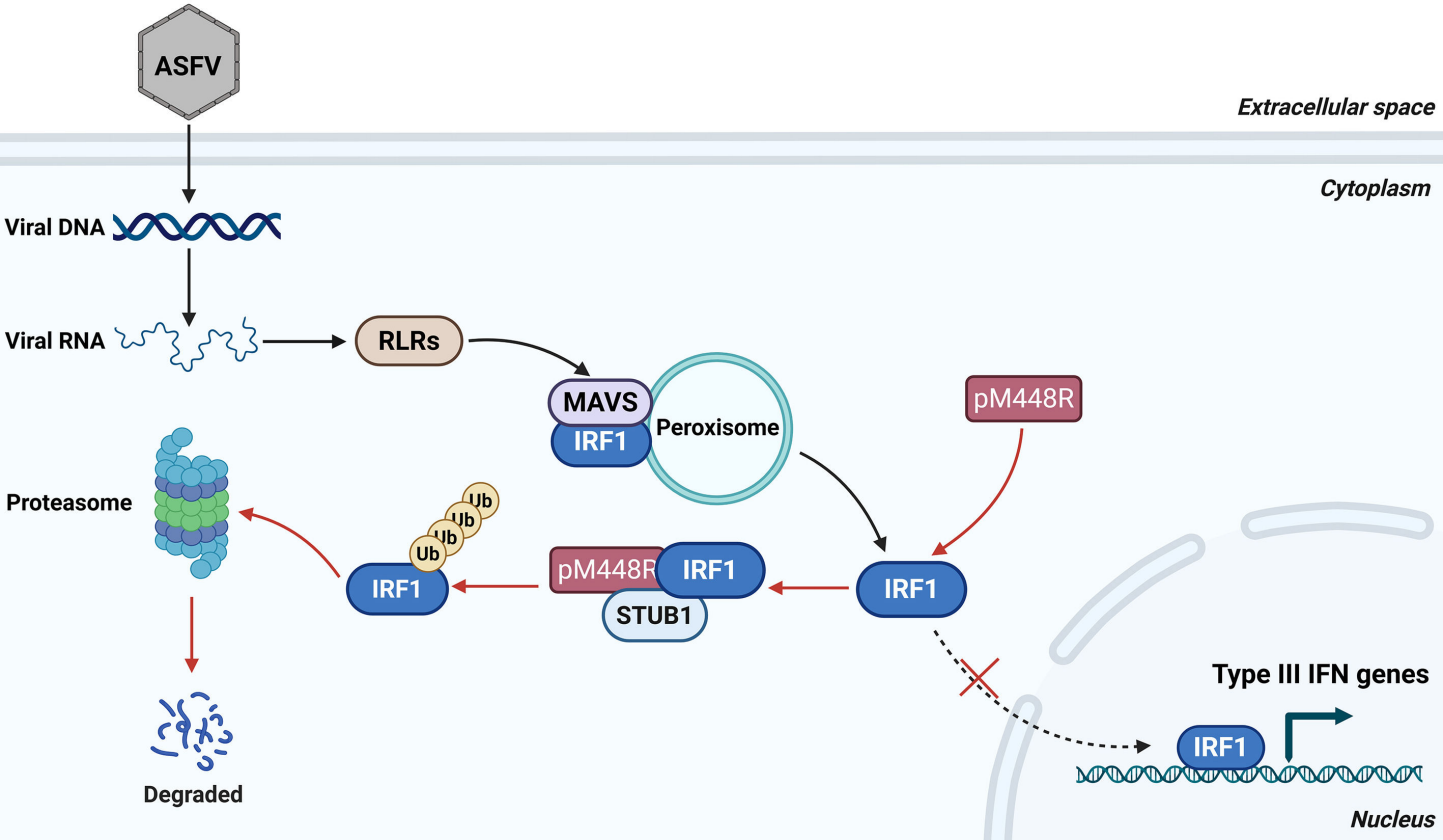
Schematic diagram illustrating the inhibition of the production of IFN-λ by pM448R through targeting the IRF1. Following ASFV entry into host cells, viral nucleic acids are recognized by host pattern recognition receptors (PRRs), which activate the MAVS-IRF1 signaling axis. This cascade drives the expression of IRF1-dependent antiviral genes, including those encoding type III interferons. However, the ASFV protein pM448R dampens the host defense by recruiting the E3 ubiquitin ligase STUB1 to promote K48-linked ubiquitination and subsequent proteasomal degradation of IRF1, thereby limiting the transcriptional output of the key mucosal immune regulator.

In summary, this study identifies the ASFV protein pM448R as a viral antagonist that targets the transcription factor IRF1 for ubiquitin-proteasomal degradation by recruiting the E3 ligase STUB1 (Fig. 8). By promoting IRF1 degradation, pM448R suppresses the expression of IRF1-dependent antiviral genes, including those encoding type III interferons. This work provides the first evidence that ASFV actively disarms IRF1-mediated transcriptional programs, offering new insight into the strategies by which the virus evades mucosal innate immune defenses.

## MATERIALS AND METHODS

### Cells and viruses

HEK293T cells (CCTCC, GDC0187) and IPEC-J2 cells (CCTCC, GDC0774) were maintained in DMEM (Sigma-Aldrich, USA) supplemented with 10% fetal bovine serum (FBS; Lonsera, S711-001). PAMs were isolated and maintained in our laboratory. The ASFV CN/JS-1/2024 strain (GenBank: PV541693) was isolated from the field samples in China, and the viral titers were quantified by TCID_50_ assay. All experiments involving infectious ASFV were conducted under biosafety level 3 (BSL-3) conditions at Yangzhou University. All work was performed in full compliance with guidelines approved by the Ministry of Agriculture and Rural Affairs of China.

### Construction of recombinant ASFV using the homologous recombination method

The recombinant virus was generated using homologous recombination as previously described (38). Briefly, PAMs were transfected with the recombinant donor plasmid using Lipofectamine 3000 reagent (Thermo Fisher Scientific, USA). Four hours after transfection, the cells were infected with the parental ASFV CN/JS-1/2024 strain. At 48 h post-transfection, GFP-positive cells were sorted and co-cultured with fresh PAMs. Subsequently, the supernatants were harvested at 48 h post-infection and used to inoculate fresh PAM monolayers. The purification was repeated multiple times until GFP expression was observed in the entire cell population, indicating a pure recombinant virus stock. The successful generation of the recombinant virus was verified by PCR using M448R-specific primers (M448R-F: 5′-ATGTCAAATGAAAGTTTTCCCGAAACGTTG-3′; M448R-R: 5′-TTAAAAATGGGAAATAATTGACAAGTAAAGCATGGC-3′). Viral titers for both the parental ASFV and recombinant ASFV-ΔM448R viruses were determined by TCID_50_ assay.

### Plasmids and antibodies

The coding sequences of 160 ASFV genes were amplified from viral genomic DNA and cloned into the pCAGGS expression vector, incorporating an N-terminal 3 × FLAG tag. Separately, genes including IRF1, TRAF3, TRIM28, STUB1, CBL, TRAF6, and SPOP were amplified from PAM cell cDNA and ligated into the pcDNA3.1 vector with C-terminal HA or V5 tags. The wild-type IFNλ1 and IFNλ3 luciferase reporter plasmids containing IRF and NF-κB response elements, along with the IFN-λ1 mutants (p-55λ1mut.IRF-Luc), were kindly provided by Professor Takashi Fujita of Kyoto University, Japan. Specifically, the mutation in mut.IRF-Luc consists of the replacement of all thymine (T) residues at positions 164–215 with adenine (A), which inactivates the IRF response element (40). The plasmids pIRF1-Luc (for measuring IRF1 transcriptional activity) and pNFκB-Luc (for measuring NFκB transcriptional activity) were purchased from Beyotime. The plasmid pIRF3-Luc, used to measure IRF3 transcriptional activity, was purchased from NovoPro. The GAPDH (60004-1-Ig) and STUB1 (68407-1-Ig) antibodies were obtained from Proteintech. Antibodies against IRF3 (A2172), phosphorylated IRF3 (AP0995), FLAG (AE092), HA (AE105), and Myc (AE070) were obtained from ABclonal. Fluorescent dye-conjugated secondary antibodies (goat anti-mouse DyLight488 and goat anti-rabbit DyLight549) were obtained from Abbkine Scientific. A monoclonal antibody against ASFV p30 produced in our laboratory has been verified for specificity by IFA (Fig. S1).

### Immunofluorescence staining

IPEC-J2 cells were fixed with 4% paraformaldehyde for 10 min and permeabilized with 0.1% Triton X-100 for 30 min. After blocking, the cells were probed with primary antibodies (mouse anti-FLAG or rabbit anti-HA) for 1 h at 37°C, washed three times with PBS, and then incubated with species-matched fluorescent secondary antibodies for 1 h at 37°C. Nuclei were visualized with DAPI, and imaging was performed on a Leica confocal microscope.

### Dual-luciferase reporter activity assays

IPEC-J2 cells were co-transfected with a firefly luciferase reporter plasmid, the pRL-TK internal control plasmid, and various viral protein expression constructs (with empty vectors as controls). At 24 h post-transfection, the cells were stimulated with 1 μg/mL poly(I:C) for 12 h. The cells were then lysed, and luciferase activity was measured using the Dual-Luciferase Reporter Assay System (TransGen Biotech, FR201-01-V2) according to the manufacturer’s instructions. Three independent experiments were performed with three technical replicates.

### RNA isolation and PCR analysis

Following total RNA extraction with FreeZol (Vazyme, R711-01), cDNA was synthesized by reverse transcription using HiScript III RT SuperMix (Vazyme, R333-01). Gene expression was quantified by qPCR on a LineGene 9600 Plus system (Bioer, China) using SYBR Green Master Mix (Yeasen, 11203ES03). Relative expression levels, calculated by the 2^−ΔΔCT^ method (41), were normalized to the GAPDH gene. Primer sequences are listed in Table S1. Three independent experiments were performed with three technical replicates.

### Co-immunoprecipitation

HEK293T cells were cultured in 100 mm dishes and transfected with the specified plasmids upon reaching 80% confluency. Cell lysates were subsequently harvested and subjected to immunoprecipitation with antibodies against the target proteins.

### RNA interference

Two siRNAs targeting STUB1 were designed by GENECREATE (Wuhan, China): STUB1-1 (5′-AGUUGGAGAUGGAGAGCUATT-3′) and STUB1-2 (5′-GAUGCAGCAGCACGAGCTT-3′). The mixture the of two siRNAs targeting STUB1 was transfected into IPEC-J2 or PAMs using RNAiMAX (Thermo Fisher Scientific, 13778150).

### Statistical analysis

Statistical analyses were performed using one-way ANOVA followed by Dunnett’s test for post hoc comparisons. Data are presented as the mean ± SD, as indicated by error bars in the figures. Statistical significance is denoted as follows: *P < 0.05, **P < 0.01, and ***P < 0.001.

## Data Availability

All data are available from the corresponding author upon reasonable request.

## SUPPLEMENTAL MATERIAL

Supplemental text jvi.00580-26-s0001.docx Complete genome sequence of ASFV-JS-1.

Supplemental material jvi.00580-26-s0002.docx Fig. S1 and S2; Table S1.

ASM does not own the copyrights to Supplemental Material that may be linked to, or accessed through, an article. The authors have granted ASM a non-exclusive, world-wide license to publish the Supplemental Material files. Please contact the corresponding author directly for reuse.
