# Supplementary material for: African swine fever virus pM448R protein promotes STUB1-mediated ubiquitin-proteasome degradation of IRF1 to attenuate type III interferon induction
Source: J Virol. 2026 Jun 9;100(7):e00580-26. doi: 10.1128/jvi.00580-26 (PMC13386975; doi:10.1128/jvi.00580-26)
Supplement: Supplemental text — Complete genome sequence of ASFV-JS-1. [file jvi.00580-26-s0001.docx]

**Complete genome sequence of ASFV-JS-1(GenBank: PV541693):**

GAATATACCATATTATTGCTATTGCCATCAATGAGAATGCCACGTAGGCATAGGTCATCCTATGGCCGGACCAATCCATGGCTGCACTTAAAAATATCAAAAAAAGTTTAAGTTTTGGGCCGGCGTTAAAATTTAAACCTTTTCTGGTTGATCTTTAGCCATGTATAGCTGCGATGTTTGGTGCCTTATCTACATGCTATTGGCATTCCTGATATTCGCACTAAAGTGCTATGTTACAACCGTCTTATGCGTGATTTTTATCCACCTTATTGGCCGAAGGGCCGCCTTGTATTTCCTGTTAGGTGGTTTGGCCGTATTCTACTGGTGGCAAGCAGCTATCAATAAAATTTAATGGCTCTCACTTAAGATCCTTGCTGTAAGCGGGCGTTTACATACTTTGATCAAGAAAAAAAATTATTTTTGGACCCCCCCCCATGTTTTATACAAAAATCATATAATAAAGTGGCGACAATCAACATATTAATCAACCACAGCATTTTATGATGTGTTAATCAACATATACCATATTAATCAACCACAGCATTTTATGATGCGTCAATCAACATATTATTACGGAGAGCGTCAATCAATATAATATTGAGAACAGCGACTTGATACCGTGTATGGTGGTGGCGGCGGCATGTTGTTTGTAACAGCATTTTTCATCATTCGAAGCTTACAAAAGATATGTATAAGATAGCATATTAATGTTATTAACAGTAATATCAATAAGGCACATTAAAATTATTACTACTGCTGTAGGCGTTAAACATTAAAATTATTACTACTGCTGTAGGCGTTAAACATTATTACTGCTGTAGGCGTAGCTAAATTTTAATGTTTTTTCGTAAAAAATCGCCAGTCACAAAAAAAAGCAGCCATGTATATCTGTGATGTTTGGTGTTTTATCTACATGCTATTGGCATTCCTGATATTCATGTTAAAGTGCTATACTATAACCATCTTATGTGGGCTCTTTATACATCTTATTGGCAGGAGGCCCACTATATATATCCTGTTAAGTAGTTTAGCTGTATACTATTTAATGTTTTTCGCTTGATCAAGAAATATACAAAAATCTAAGTGCACCATTTTATGGGCATCAATCAATCACAGCATTCTATGGGCGTGAATCAATCACACCATTTTATGGGCGTCAATCAATCACACCATTTTATGGGCATCAATCAATCACAGCATTCTATGGGCGTGAATCAACACATTATTGGGGACAGCTACCTGATATCGTGTATGGTGGTGGCACCGTGTTGTTTGAAGGCGTGTATGGTATTAGCGCCATGTTGTTTGAAGGCAGCGCGTTAGTAACAACGTTTTTTTTCGTATGACAACAGATATGTATAAGATAGCAAATTAATGCTATTGTCATCAATGAGAAACATAAGTAGATATAGACCTGCCTCTTGGTAGTCCAATGATCCATGTTGCACCTAAAAATCCCAAAAAAAAACATTAAGTTTTGGAGGGTAAGATTGGTTTTTCACCATTGGTAAAGATTATTATTCTAAATGTTTACCCCATAGATGTGAAACAATGATTCTTCATATATTAACATATTTTTTGACTTATACTTTTCTTCATCTAGTAAGGCGTTAATTTTTTCCGGATCTGTCGTTTTTATTGATAAAAGAGAAGAGTCTGGACTGTAATTTTTAAATAATAAGATATTTATTAATATCCAATTATTCGTTTGACTCGCTATTTCCATGCTCTCTTCAAACGCATCAGCTCCTAAATCTATACAAAGGAATAAGTTACCTTCACAAAAATTCATTACCGAGGTAATCATTGCCCGATTAATGTCAGCTCCCAACATACAACAATAATATATAGTTGTATAATTACAATCATACATACAGGCCAACTGCATCATTTCATCAATGTCTATATTCGTCTTCTCTTTGTTATAAATTTCATGAAGGTCAAAGACGTTGTTATAAGCAACCCCACATATTAACCGCCAATCTTTAAAATGACTATATCGTTGATAAAAATATTGGATGGCTTCAGTAAGCTTATATAGTATCGCCATACTATACCAATACCTAGTTAGCATTTCGTTGAATGAAATATTATCCAATGTAAAGTTGATTGATAATGTATCTAGTTCACCAAAAATTCTTAATTTCAGTTGAGCATTATTTAGGAATAGGGGATTATCAGATAATAATTCATGGCATAGAATAATATTACTGCTAGTTTTAACATACTGTACATTATAAAATATTTCTAAAATTTTATTTTCACTCAAAGCTTTCCTCGCACCTAACTTTTGGCATAGGTCCTGGGTGCACTCCATATTGACAGTAACCAACCCAAAGCTGATGTCTGCACCCCATTCGGTAAACAGCTCTATTAAACCATGATTGTTTTCCTGTACAGCCTTCATTAATGCAACATTTAATGTTAAACCATGTTTAAAACTTGCTGTTTTTATTAATATTTGTTTATCTATACAAGTATGATGAATCGTAATTGGGGCTTCATGCCACCACAAACCACAACGCTCTAAAATACAATAATCATCTTTGAACACAGGCTGTGTAGCTAGTACTTTTTTAGTAAGTGCTTGTAAAGTAGATGGCATCTTCTATCTGCAAAATAATTATTTCCGAAAAAAAAATCAAATTAAAATACTAAATTCTATTTTTTTTTAATAAAGCCTGTAAATTATATAATAAATCTCGCCCACCGTATTATTTTCGGACACAACTTTTTATACCTCATTATATTTTTAGATCTATAGTTTTTTAACAAGGCATTAATTTTTTCTGGATCTGTCGTTTTTAAAGATAAAAGAGAGACGTTTGAATTATAAAAATCTTTAAATGATAATATTTCTACTAATATATCATGATTCTTTTGTTTTGCTAATTCTAAGCTCTCTTCGAAAGCATTAGCTCCTAAATCTATACAAAAGAACAAGTTATTAGTATAAAAGTTTTTTACCGAAGTAACCATTGCCCGATTGATGTTAGCCCCTAATACAAAACAATAGAAAATGGTTAAAAAATTGCTATCTCTCATACAGGCCAGATACATCATTTCATCAATATTCATATCAACCTTTTTTATATGATATATTTCATGAAGATCAGACACGTTATTAAAAGAAAGCCCACATATTAGCCGCCAATCTTTAAAATGCCTATAACGTTGATAAAAATATTGGATAGCTTCAGTTAGGTGATATCGTACCGCCATACTATACCATAACCTAGCTAGCATTTCGTTCAATGTTATTTCATTCAATATAAAGTTGATCGATATCTTCTCTAGAAAACAACAAATTATTATTTTTAATTCCTCTATATTCTGGAAAAGGGGATTATTAGATAACAATTTATGGCATAGAATAATATTACTACTAGTTTTAATACGATGTATTTTATAAAATATTTGTACAATATCCATTTCATTCAAAATTTTTGCGCCTAACTCCCGGCAGAAATTCCAAGTATGCTCCGTATTGACAGTGACTAAGCTAGAGTTGATGTCTGCACCCCATTCGGTAAACAACTCTATTAGATCATAGTTGTTTTCCTGCACAGCTTTCATTAATGCGAGATTTAACTCTAAACCATCTTTAAAAATTGCTGATTTTATCATCAATTGATTATCCTCATTAGTAGAAAACATAATTGGAGCTCCATGCCACCACAAACCACAATATTTCAAAATAAAGTAGTGATTTTTGGATATGTGCTGTGTGGCCAGTATTTTTTTAGCAAGAGCCTGCAGAGAAAGTGGAGTAGACATTTTTTTTTGCAAAATGGTTTAAGTTTTAATGTTTTCAAGAATACAGATTGGATAAATTAGGTTGACATTAGTAACAGAAAGTATTCAATATTATGTAGACCTATAAAAAATGAGACCCTCCAAAAAATAATAAACAACACAAAAAAAATATGTTTAATATTAAAATGACAATTTCTACATTGCTTATTGCTCTTATTATACTAGTTATTATTATTTTAGTCGTATTTTTATATTATAAAAAACAACAACCACCAAAAAAGGTCTGTAAAGTAGATAAAGATTGTGGTAGTGGAGAGCATTGTGTTCGTGGAACATGCAGCACATTGAGCTGCTTAGACGCTGTAAAAATGGACAAACGAAATATTAAGATAGATTCTAAGATTTCCTCATGTGAATTCACTCCCAATTTTTACCGTTTTACGGATACTGCTGCCGATGAGCAGCAAGAATTTGGAAAAACACGGCATCCTATAAAAATAACTCCATCTCCAAGTGAATCCCATAGCCCCCAAGAGGTGTGTGAAAAATATTGTTCATGGGGAACCGATGACTGTACAGGTTGGGAATATGTTGGTGATGAAAAGGAGGGAACATGTTATGTATATAATAATCCACATCACCCGGTTCTTAAATATGGTAAGGATCACATCATAGCCTTACCTAGAAATCATAAACATGCATAAATAAATACATTAGGCTCATCGTATCCTTTTTAAAATCCATAAATATTCGTTTGATATATGCTGAAATTTTTATAAAAAAAAATAACTATTTCTTATAAATCATCTAGAAATAGTCCTCGTTTTGATCGGTTTATATCTTATAATATTGTGCATCGATGCACAACTGCTTTTTTTGGTCCTTCTGGAACATCATTATATTTTCTTTCATTAATATACCATTCAGATGTAAATGTTGAATAATTTTTATGGCAACAATCTACCACTGAATTATATTTAGTAACATCTAATACATCGCTTGTTTTATCAGGCTCAGCTTTATAATCTTGATAATTTTTGTTATCAGCTTCTAAAGCTCCATCATTATTTTTCAAAGAAGTGTCCATAATCATGTTTGGTAAAAACACTTTAAGTTTTAATATGATATTTAAAATGGTTGTTATATAAATTTACCGCTTACAGGTAATCTTTATTCAGTATCATAAACTATACTTTTGATGGTTCAGTATTCTGTAAATCGGTATTTTGTGATTCAGTATTCTGTAAATCGGTATTTTGTGAATCAGTACATTTATTATCATTAATATTTTTAGGCTGATTTTCCAATGTTTTATTGTTGCAATGAGCCTGCTCCTCCTTTAACGGGGAAGTGTCTGTTGGAGTCATCTGTTTAGGAAGAGTATCATCCATATCTATTATGAAGAAAATATATAAATATTGATATACAATCAAAAATATTTTTGATCACGTCTTTGTTATCTATCGATATTGTTGATAACGTCTTGAATAACCTACATCATTTTTTTACATAAAAAAATAGATATAATTTTTATTATATCTCAATTATCTTAAGATAATTATCAAAGTAACTATTTTAAAGATAATTATCAATACAACAAATATCATAAGCTAACATATTTTTCGAATAATAGTTTTTTAGTAAAGTATTAATCTTTTCAGGATTAGTTTCTTTTGATAATAAGATAGGATTCGCTTTATAAATTTTTAAAGATAATATATTCACAATGATAGAATAACCGTATATATCTGCTAATGTCTTACTGTGTTCAATAACATTAGCCCCTAAATCCATACAAAAGAACATATTTTCAATACAAAAGTTTTTTACCGAGATTAACATTGCTCGATTAGCGTTGGCTCCCAATGCAAAACAGTAGTAAATGGTCAAAAAATTATTGTCGCGCATACAGGCCAGCTCCATCATTTTATTAATACTCATATGAATTTTCGTTGTGTTACATATTTCATGAAGGTCAAACACATTGTTGAAAGAAAGTGCACAAATTAATCGCCATTCATCAAAATGCCTGTATTCTTGACAGAAATATTGAATAGCTTCTTTAAGATTATATTTTACCGCTATGCCATACCAATATTTGGTTAGCATCTCACTAAATGAGATCTCATTTAACATAGAATTTGTTGGTAAATCCTTCAACTCCCAATAAATAATCATCCTTAAATCCACCATGTTTACATTTTGTAAAAAAGGGTTATTAGAAAATAATTCATGACACAAAATGACATTACTACTTGTTATTTTACACTTTGTTTCAAAGAAAAATCGTAAAATTTCACTTGTCTCAAGCTCTTCTTTAGCCCCCAATTTTCGGCATAGGTTTCGAGTATGCTCGTTATTAATAAAAAGTAACCCATAATTAATATTTGCGCCCCATTCAGTAAACAACATGATTAGATCTTCATTGTTTTCCTTAACTGCCAATACCAATGCAGTATTAAGCTTTATACCCTCTTTAAAGCATAATGTCCTTATCATTATTTGATTATCATCATCTATATAAATTGAGATAGGAGCTTCATGCCACCATAAACCACAACGCTCTAAAATATAATAATCATCTTTAGATACGTGTTGCGTGGCCAATGCCCTTTTAGCAAGTGCTTGTAAAGTCGATGGCTGCATGTTTATTTTGTTAAAAAAAATCAAATTATCGGGTAAACATAAGGATCAACCCGTAGTTAATATTTGCAGTGACATTTTTAACAATGAATTATGATAAAAAAATAATTCATGACTATCTATCATGGAACCCATCTTTAACATTAAAGAAAAACCAGATCATCATTTTTTTTTGTTGTATTAGGACTTCTTCAAATTATTACCCACATTTTATCTAAAAAAATAAAACTACATAATATCTTGTTTCTTCATCAAATTATCATACCATTTAAAGTGTGTAGGTTGGGAACATTCCATAATATGGGTATCAAAGTGTTTATATATTTTTTCATAGGAACATTTATTTGGCAGATGTGTTGTCCAACAATCATATCTAATAAAATCATTTTCACCTATGGGGGGATGATTCTTAAAAGCCTTATTCTTACAGATGCCATTTTGACAGTCCCAGCAAAAGTTACAATATTTTCCATGAGTACACCAATGTTCAAGCTCTCTTTTGGGAGGAATGCTGCCAATTTTATGTTTTTTATCTTCTAACTCTCCATACAGCATCAGTTGGGAAAGCAGAAAGGAGATTACCAGGAAGACCAGCAAATACATAACAGTCTGTAAACTACGTTTGCGAATGTAATTCACAGCTAAAATACATCCCGCAAGATAAAATCCATAGGTTAACAATTTCTGCCATTTTCGTATATCAGCCTCATGCCTTTCATGGTTTATGTGTTGTGGACATTCTGTTCGGTACGTTTTATGAGGCTTTATAGAAGTTATGTGGTAGATACAGAAATCATTGCGACGATAAATACTGCAGTTAGCTATATAGTCATTTTCAAGAATGGGAGAATGGTTTTTAAAAGCCTTGTTCCTACAGATACCATCTTGACAGTCCCAACAGAATCTACAATGGTTTGCATAAGTGCACCAGTATCCAAGCTCCTTTTCAGGTGGGGTACTTGTTCGTTCCAGGGGCTCTATCTCATATGTATAAAGAAGAGTTGGAATAGATAGTAGGGTGAATATTTGCAGACCAAGCATGGCTACTTGTGAACAAGTGGCTGCTCGTCAGCAAATAGCTGCTCGTCAGCAAATGGCTGCTCGTCAGCAAATGCTGCTTGTGGGTAAGCCAATAAATGGGCCATACCCTTGAAAGGAGAATTCAGTTTTGATAAAAAAAATAACGAGTTTTCTAATAACCCGGTCAAGCATTTATTAAATGAGTGGCATCAGATGTCTGTATCGTGCATTCTACTTGGAAAATGGGCCCATCTCTAATATATTTACACTGACGGTGAATCATACAGTGTTTCATGGGATAGCTATGCTCCTGTACAGAAGACATATCTTTAAGAACTTTATTCTTACAAAGACCATCTTGACAAGTCCAGCAAAACCGACAATTTTTCACATATTGACACCAGTATCTTAGCTCCTCTTCCAGGAGATTGTCGGTCGAAAAACCCTGTAGACTAGTTAGACCAGCTAGCAGCAAGCCGAGGTAACTAAAGAACCTCATTGTAGTGTTATATTACGAAAAAATATGTTAAAATTTGGAAAAAAAAGCCCTTTTTATAGATCTGGAAAAAAATTTTCACAAATCTAATTAAAATCCTCACAGATCTTCCTTTTCATAAATTTTCCTTCCCAATCGGTGTGGACGATTGTGAGGTACTGGATTAGAACAATCCATTACATGGTAATGTCCATTTCCTTCATCATATGTACACTGGTTATACCAGCGAGAAACCTCACAAGATGTCAAATAACTGTTCTCAATAATCCATGGCATGCTCCTATTCACCTTGTTCTTGCAAATTCCATGTTCACATTCCCAGCAAAACTTGCAGCTTTCCATGTAAGTGCACCAGTATCCAAGTTCTTCTTGTGGAGGATTATCCGTTGAACGAAGATGCCCTTCTGCCTGAGTAGGTAGTCCTAAGACCTGATTGGCCAGCAGGCCAAGAATTCCCAAGAAGATCACCAACATTGTTACGGCTGGCTGAACAGCTGGCTAAATAGCTGCTGAATAGCAAACCAAGTGACTCGCCCTCTCTACTCTTAATATGAAAATTTAAGATTCGGTCCAGCATTTTTCCCATGTTTTACAGGGAAAAGATATTTTTATTCTATGAATGCACATGGTTCCGCACATTAAAAAAATAAATTATTTAATTTTGGCTAGCCGGGGTAACTAAAAACTCATTGTAGTGTTATATTACGAAAAAACATGTTAAATTTTGGACAAAAAAGCCCTTTTTTATAGGTCTGGGAAAAAAAATCAGATCTAATTAAAAACCTCACAGATCTTCCTTTTCATAAATTTTCATTAACAATCGGTGTGGACGATTGTGAGGTACTGGATCAGAACAATCCATAACATGGTAATGCCCATTTCCTTCACTATATGTGCACTGGTTATACCAGCGAGAAACCTCACAAGATGTCAAATAACTGTTCTCAATAATCAATGGCATGCTCTCATTCACCTTGTTCTTGCAAATTCCATGTGCACATTCCCAGCAAAACTTGCAGCTTTCCATGTAAGTACACCAGTATCCAAGTTCTTCTTGTGGAGGATTATCCGTTGAACGAAGATGCCCTCCTGCCTGAGTAGGTAGTCCTAAGACCTGATTGGCCAGCAGGCCAATAACTCCCAGAATAATCACCAGCATTGTGCTCAACCAGCAACGGCTAGCAACGACTAGCAACTGACTAGCAATAGCTAGAAATGGCTAGCAACCAGTAGTGGCTAACACTCTACTCTTTATAAGAAAATTTAAAATTCGATTAGATTTTTTTGGATTGAAAAAGAACAAAACGCTTATATTCTTTTCCTAGCGAGAAAAAATAAGCTAGTTTAAGATATGATTTCCCTTACTAACGGTTTAATTTTTGGCAAGGGTATAAGGTAAAATACATTTAGCTGAAAAAAATAAGCTTATGGTGTATAAACCACCATAAGTTTATTTAATTAAAATGTTAAACTCTGTGATAAGACTGGAATCTTAGGCAGGCTTGATGTGGAGAACAGCATGAAATACAAGAGTGCCTGCTACATGAATAAGTTCTCTCAAACCGAGGATGGTCATACTCACATCTATGAAATCCTGGTCTAGGAGATTCATTTGATGCATGATGGCCGCACCCACACTTATGAGGCACTGAAGAACTAAAGGGTTTAATTCTGATCTGAATGGTGCTATATAGGATGATGGCAATCCATATCAAGATCAGAGCAATCAAAATCACCTCCTCAAGAAGCATGATGTAGCCTTAAATCTTAGGCTGCCTTAAATCTTAGACCGCTTTAAATCTTAAGCCGCCTTAAATCTTAGGCCCTCACTATCTTTAATGAAGAAGTTTAAATTTTGATCCCCCTTTTTCAAGGCCGATATAAAAGAAAAAATAAAGTTTATAACAATCTAATTCATAGGTCATCTCTTTCATAGATCTTCATGTATTCTCTATGTGGATAAGTATGGGATGTTGGATTTGCACAGTCCATTTCATGATCCAGATATTGTCTAGAACCTTCATAATATCTACATTCGCCATTCCAGCGGGAAACCGTGCAATTTATAATCCAGTCATTTTGATGAATAACTGGCCAATCTGCTTGAATCCTGTTTCGGCAGATACCGTGGACGCATTCCCAGCAAAAGTCACATTGATTTGCGTAGGTGCACCAATAAACCAGCTCATGTTTAGGAGGATAACGGGTTGGTAGTAGATCTTCTAATTTACGTATAGGTGCGGCCTGAAGGATAATACCCCCCCCCCCCCAGTAGTACTAGAATTAGCACCTTCATAGTGGCTACCCTATACTAGACCTCTAAGTTGAAGACAAAGAACTAAAATTTAGAGCCGTTTAATTATTACTAATAATGATATTTTATTGTCTATAATAGGATTCTATTAAAAAATAATGATTTTTACCAAGAAATAATTTTATAAAAAATTAATATATTTTGTAATAAACTTTATTTCTAATGACTGTTAAAATAAGGAAACTATCCTTAGTTAGTCGAGGAAGATGGTTAGGTTATTTCGCAATCCGATAAAATGTATATTTTATCGTAGGTCTCGTAAAATCCAGGAAAAAAAATTACGGAAGAGCTTAAAAAAGCTAAATTTTTATCATCCTCCAGAAGATTGTTGTCTAATATATCGTTTGCTAGAAAATGTTCCTGGGGGAACTTACTTTATTACAGAAAATATGACGAATGAGTTAATAATGGTCGTAAAGGATTCGGTGGATAAAAAAATTAAAAGCGTTAAATTATATCTTTATGGTAGTTATATTAAGATTAATCAGCATTATTATATTAATATTTATATGTATCTTATGAGATATACCCAAATTTATAAATATCCCTTAATTTGTTATAATAAATATTATAACATCTAAGTAAATATTCTTGGAATGGATTTTCTTATAGAATGATTACAGGATAGGTCAGCGACAAGATTAATAACAAATTTGCTATTTTAATATTTTTGTTAAATAAATGAACGTTAATGTTACCCGTTGCGGAAAAAAACAAATTTATAATTACACATCATTAATTACATGTCGTTTTAACTCTATGAACCATTCTAAATCTTTGGGTTGTGAACAATTCATGTTATGTTGATAGTGTATCCTGAAATGGGCTTCGTACATACACCGGTCATGCCACCGGGAAACTGTACAATTAACAATATAATCATTTTGAGTAATAATAGGGTGGTCACTAAACACTTTATTTTGCACATTCCATCTTTACAGGTCCAGCAGAAGTCACAGTGTTTTGCATAGGTGCACCAGAACTTGAGATCCCTTTCAGGAGGCCTACGCATTTGCATCGGATTATCTGTGGTAAGAGGTAGGTTCATTATTATGTTCGTCATCAAAATTCCTAAAAGAACATAGAGGCCAAGAAAGATAAGCAGTCTTGTAGCGGCTTGCATTCGCATTCGTGAATACTGTTTGCGAACATAGCCTATGAGGGCAATAGTAGCTATCATACAAAGGCAAGTATGTTTGATATTCTCAACGTCAATGACCCTATTCTCCTTTACTTGCATTAACTCATCAAACCAATCATAATATGTGGGATTTGTACAGCTCATGATGTGAAAGCGGCGTATCCTAGAGTCTGTAAAGTAGCTACATCTATTATTATAGCGGGAAACCCTACATATTTGTATGTAATCATTTTTTTTGATGAGAGGATGTTTTTCAAAAACCTTATTTTTGCAAATCCCGTGTCGACAATTCCAGCAGAAGTCGCACGATTTTGCATAGGTGCACCAATACTCAAGCTCTCCCTTTGGAGGTCTCCGGGTCATTGGTAACTCTTCTGTTCCTGGAAAGGGTTGGCTTTGAATGACCGGCTGCATGACCGCCAATACCAAAAGGAGCACAATCACCTTCATGGCTGCACCGTATAGATCGCGACTCAAAAGGTATGAAAACCTTACCCTCAATATAGAGTTTAAGTTTTAATCCTGATAATGTATCTGTTTATAAAAAAAAATTTTCACTCATGTATGAATTCTTATACGAATCATAATATGTAGGCTGAGAATAATTATATACGGTGTTGCAGGCTCATTAAAATTTTGTTACCACAAAAAATAAATGCTGGATCTTTAAGATATATATATCTATTAATGACTAAACCCTTTATATGCTGTAGGTTGAGAACAATCCATATAATGAATATAGGGTGATTTGGGTTTAATAAAATATATACAACGGTCAAAATAGCGGGCAATACTACATTGACTAATATAATCATTTTGTTTAATAAGAGGCATATCATCCCAAACTTTATTTTTACAAATACCATTCCTACATTCCCAGCAGAAATCACAGTGTTTTCCATACGTGCACCAGTATTCAAGCTCTCTTATAGGAGGTGTATAAGTCCTTGGTAAATTTTGTTTCATATAAAAGATGGAAAGGGGTCGGTTTAAACCCGGCTGAGATAGCCAAATCAAAATACATAAAAGAGCAAGTAGTTTCATAGCGGTATTTAGGTGTAAATTTTTATAGTACGCAAATACAATGTAACCTACAAATGCAATACTAAATACAAGGTAAAAACAACAATGTCTTATAATGGTTGGCCAATAATCACCCCCCCCCATTTTTCCATGAATATTTCATTTCCTGTATAGGGTCTAGGATGTGAACATTCCATGCTATGATGATTAGGTATTTTAACTGATATTTCATATAAACAGGATTGAGTCTTGGAATCACGAAAAACTCTACAGTTTACAATAGAATGATTGGAGTCAATGAAACGAGATTCCGTTATCTTATTTTTGCAAATGCCATCTTGACAGTCCCAACAGAAGTCGCATTGTGGTACATACGTACACCAATATGAAAGCTCACTCTTGGGAGGATGCTGGGTTCTTGGTAAGTCTGGTAATTCATGTGCGAGAATGAGGACTGAGTGGCCCAACAAAAGTCCCAGAAGAACCTTCATGTTGCGTCTAAATAACACCTGCACTTACAAAAAAAAAATTTAAATTTTGAATATAACACAAAAAACCACCTTAAAATTTCTTATATTATTTCTTGGATCTGCCCGACGTCATACAATGTATTAAAATTATAGACCAATCATCTTTGTATATAGATTTTAGTTATTTTGCTTGTTGTATCAACTTAACTGCTAGTGAAGAAAATGGATAAAAACTTTCTGTATTTTTATAGGTTGAAATCATTTTACGCACATCACTAGGATCTAATATTTTATTTTGAAGAACTGAATGTGGGCTTAAAATTTTTTTCTTAGAAAAGAGTAGAATCATAATATTGCTATGTTTTTGTTTAATGGTTTCTTGTATCTTTTTTGTATACGGGTTGGCACCCAAACCTATACAAAAATATACATTACTCAAATAACTACCTTCTATACATAATCTTGTTTCCCCACTTATTTTCCTATTTATTTCCCTATTTATGGAATTAAAGGATATCAATCTCTCTAAGGCACGGTCAAGGTCTGCGCCTAAGGCAAAACAATAATATATACCTAATTTATTCCCAGGGCGTGCACAGGCAAGAAACATCATGACGTTTAGAGATAAACGTATATTTTCCTGAAAATACGCATGATGAACTTCATCAATATTACCTAAGTATATGGCCGTTTGTAAACGCCAAAGATCTAAATGAGGAAATTTTTTACTAAGATAACGAATGGGTTTTGTAAGTTTAAAATCTATGGCGAACTTATACCAAAATTTTAATACAAGTGTATTTCTCGTCATTTCTTCTTCTTTTTCATCTAAATATAAGATAAAACGATTGTAAACAAAGTCTATCAACACGTGAAAATCATGGCTGTCAAAACTGTCGAGAATCGAAATATTGTCATAATAAATATCAATCGCCAGTAAAACCTTTTTTTGTTTGATAAGATAAACAAACATATTATACAACCCTACATCTAAAAATTCTGGATCGGCTCCTAGTTGGATACACAGGTCTTTAGTCTGCTTCGTTTTGGCACACATAATGCCAAAATTAATTTCAGCACCCCATGAAAGAAATATCTTGATTAGATCAGTCTGGTTTTCCTTCACAGCTTTTACTAAGGCTCTGTCAAGCTCATAACTGTTGACATCAGAGCATGACATAGAGCCATCGGTTACCATTTTACATTGCTTACAAAAACCTATGGGTCCGTTTTCCCACCATAGTCCAAGCTGTTGTAGAATAAAAATATCATCCTCATGATACTTTGAAAAAGCCTTGGTTTCTATCAAGACTTTTTTAGTAAGAACCTGTAAAGAGTTCATCGTATTATTATGAATAACAGGAGTAAACGTAATCAATTATAAAAAGTGACTTTTTTGAAAAATCTTCAAATGGTTGAAAACGATAATGTACATGTTCATACAAAAAATATATTCAGTGATGTCCAAAATCAAAATTTAATTTTCTATGTAAAAAGTACAGACGTTGCCTATTCGGGTTAAATTGTTTATTTTAAACTTTAATTAACCATTTGAGTTAGCGATGTTTGATTTATCTTCCATGCTCATCCGGGGGGAGTTAACGATGTTTAATATTTCTTCCATGCTTATCAAAGGGGGGGGGGGTCTTAATCGTTCTAATACTATTGTTGTGGATTGTTGAACATAATGAAAACTTTATAGATGCTAAAATAATTGAATCTAAAAATAGTAGTATACTTTAATCAAAATTTGATTTCTCATAAAAAAGGTACACATCATTCATTTAAGTTTCATGATGTTTAGTTCATTACTTCCTACAATTACTGGGGGGGGTCCCTCATAGCTTTAGTATTATTGTGGCTTACTGACTATTATGTAGAATTCATAGAAGCAACAATATATGTTACTAATGCTACATTGTAAATTATAAAATACATACTAAACTAATTTCAGTATTTTTTTTTGTTCATATAAGTTAAGGTACAAAAATGATTAAACACTGCAAAAAAAGAAAATCATAATGCTATTATACATAGTGATCATAGTGGCTTGTATCATTTCTAAACTAGTTCCAAATGAATATTGGGCAATACATCTATTTTTCATCATTATGATTTTTATGGTATATATGTATGAAAAGTTAGATATACATCAAAAATATCAGTTCTGGAATTATACGATGTCAGGCTTATCTGGACATAACGTACAGGTAATATGTAAGTGTTACTAAATACTATGAAGTATCTATTTTTTTTACAACAAAAAAAATAGATGTAATCGGTTTTATCATTTAGGTGTGTATTTCTTTAGCATCTTTCAAGAATTCATTGTTTAGCGGTATAAAAACAATGAATGATCATCATATTCTATTTAACAATCTCCTAAATAAATGAACATCTTTTTCATTTTAACATGATCACCAATAGTCATTTTGCGAAAAGGCATACATATGATCAATATCAGACCTACAATGAATGTTTCCATAATATCCCTTTATCGCAATAATTCTATTTTTGCATTCCGATATCTCATCATCTGTGCTATTATATGTTTCCATAACTGTTTCATCATCAAACATAAATCCTATTAAATAAGCAAAGCGCTTTAATCCCGGATAGATTTTCACCATTTTTCTAAGAGCCGTGTATAGCTTGTAATAAATGGCCAGAAATATGCAATAAAGCGTAGAAAGAGAGTAATTTTTGGCATAAAAGGTTTTGAAGGTTTGATGAATGGCTAAATCGCAGATAATATAAGATACGATATTAAAGCGCACCTGTTCACGCAGATTTGTTGAAAACTTCGTAGAAAGATTCAACAAATAAAAGGTTATTAATAGTTGCTCATCATTCTCCTTATACGACATCGTCAGACGCTCTAAGATTTTACTACTGGACACATCTGCCACATGTTGAACATTTAAAGCCTGTTCTTCTTCTGTGTTACGACAAAAGAGCCGCGCATATTCAGGTGAAGCTCCCCAGGATAACAACGTCCGTGTCACGGCTAAATTTTTTTTGACGATGACTTTCATCAGAAATAAGTCTTTATTTTTGCATTGATCACTATGCGAATTTGTATAGTTGACGCCGTTGCATTGAGTACATTGATATAATGTTTTGCAATTCCAGCGTAGCCCTAAATGGTATAAAAGGACTGTATTTTCGACGCAAGCATGCTGATTAACGATGTTTTTGAGACAACACGTTGTTAAGGACACCATATTGTCTCCAATTTGTTGGATAAAAGTCTTTACTAAAAAAATAGGTTTTTAGTTTTAACAATCGAGATTTTATTATTTGGATATAGCATCATCAAAAAGATTTATGTTAAAATTTAATTTGTCTTACAAAAAGTACAGATTCAATAATTGGTTTAACTATTTAGTTTAACTATTTAGTTAAATTACTTGGTTAGCAATGTTTGAGCTTTCTTCCATTCTTATTCGAGGGGGGGGGGGTCCTAATAGTTTTGATACTTTTGTTGTGGATTGTTGATGAAAACTGTACAGATGCTAAAGCAATGGCATATAATATTAATTGTACTGTTTAATGTGAGTTTATAAGTATGTATCAAAATTTATTTTTTTTTATAAAAGTACAGAAATTAATGTTAGCTTAAACTTTAGTTTAGCTATTTGAGTTAATGATGTTTAACCTATCTTCCATGCTTATCAGGGGGGGGGTCCTAATTGTTTTACGTTATAAATTTGATCAAAATTTAATTTCTTATAAAAAAGGTACACATCAACATTGCTCATTTAAGTTTCATAATGTTTGATTCATTACTTCCTACAATTACTGGGGGGGGGGTCTTTAATAGCATTAATATTATTATGGTTTGCTGACTATTTGGAATTTATAGAAGCAAAAATAGATACTTTTACTAATAGCAGTGCATTATAAGTGATCAAAATTTAATATCTCATAAAAAAGGTACACATCAACATCATTCATTTGTTTCATGATGTTTGATTCATTACTTTCTACAATCACTGGGGGGGGGTCTCTCATAGCTTTAGTATTGCTATGGTTTACTAATTATTATGTAGAATTTATAGGAGCAGATATATATGGTGGTAATTGTACTCTATAAGTTTATAAAAATTTCAGTATATTTTTTTTGTTAATATAACAGAAATGATTTAAATTACAAAAAAAAGAGAATCATCATGCTATTATACATTGTTATTATAGTAGCATATGTAAGTTATAAATTGGTTCCAAAACAATATTGGCCAATACTCATGTTTATGGCTTACATGGTATATACCCATGAAAAATTAGATATAAATGAAAGATCCGGATTTTGGAAATATATTATAGCAAAACTATTCAGATGCCACGGATGTGAAATATGTAAGTGATATTAAAAACATAATACGAAGTGTAATAATTGACTATTTTTTGTTGAATGGAAACTGTTTTTGAGTAATATGTTGAACATAATGGTTTTTATAGATGTTAAATCATTTGATTGTAGTAATTGTACTAAATGATGTGAGATATAAACCTAGTTCAATCAAAATTTAATTTTTTTATAAAAAGCTACAGAGTGTTTTATTAAACGTAGCTTATTTAAAAAGTTACACAATGTTAAAATCTCTACTTACTTTAATTCTTTGTGGGGTTTTATTAACTTTATCCATATTATGGCTTATTACTTACCATGTAGAACTTATAGAAGCAATAGATGATTTCTACGACTGAAATATAGAATAGTTCATTTTCTATTTGTAAAATAATGATTTATATTCTCTCCTAAAAATACGATCGACCCTTTATATGCTTTGAAAACGAATATTAACAACTTGATTTTTTTTTCTATAAATAAACTATAAATGAAAATAGTAAAACTCATAGAGTCTTATAAGTGAACATCCATCATGTTACTCAAACGTTGGACTATTAAAAGATACTCCGTGTGCATTATTGCTTTTAATCAGTATGATTACTTTATACGAAGCCGCTATTAAAACGCTTATCACACACCGAAAACAGATTTTAAAACACCCCGATAGCCGTGAAATTTTACTAGCTTTGGGGTTGTACTGGGATAAAACTCATATTCTTGTTAAATGTCGTGAATGTGGGAAAATGAGTCTTACCGGAAAACACAGTACAAAATGTATTAACATTAATTGTCTACTTATTCTTGCCATAAAAAAAAAGAATAAGCGTATGGTTGATACCTTGATAAGAATGGGTGCGGATGTAACATATATACATCTTTTAAAGAATAAGATAAAACTGTCATACAACCAGCTGTCTATGCTTAAAAGCAACTCGCAGATTTCGTTGAAGGAGCTTCATGCTATATGCTACCTTTTATATGGTCGGCTTCCCAAAAAAATTAAACAAGGGATGCAACTGTGTAAAACAATGGCGGGACTATGTGGTGAACTTTTATGTGCATTTTTAGCTCCGTAAATGTAATATATGTATATAAAACAAATAGATAATACCAAAATATATTCTATGTACATAACATCCGGAAAATTATTTTTTTTTCTCATACCCTTAAATATAAAAATATTGGGTTTCTTCACTAAACTTTAGAGGTAAAAATTTTTCTTTGTTTTGCATCATCATGTATGGGTTTAGGTTATCCCAGGGATTGTTTATTTGAATATTTCCTAAATAGGAACACAACGCCATGATCATATATCTTTCATTCTGGTAAGCTTTTTGATACATCTTCAAAGATGCCGTACCTCCGAGTGTGTAACAGCAAACAAACGTCCGTACTTTTCCATGGGTCGCAGCCCATTCCATTCCGTAGCTCAGCATCCTTTGCTGTATTTTTTTATTCGCTTTATAAAAAAAGTATTTCATCCATTCCACGTTCTCATAAAAACAGGCACTTAAAAAGAGCACCAGGTTTAGTGTAGTCTTATTATAGAATGTAGGAATGTATGTTTTAGTTATTTTTTTCAACGCCTGTTTCATACCATGTTTTACCGCCATAAAAATACAAAACCAATACCAACTTTTTCTATAAAAGGTTTTGCTGTACACATGTAAACGAGCAAAATATATTTCAAACTCTAAATTCTTTTTATAAAAAAACTCGAGACAGTCATTTATGTTACGACATCTTCTAAAAACCTCAAAAACAGTAATTAATTCACTGTCGCTGTAGAAATGTTCATAAGCTAACTGTTTAATGTCTTTAGGGGTCAATTCTTTTTTTGGGAGCAGTGGTTTGAGATTCGGCAAAGGTCGTCTAAAGTAGTGAGCGAACTTTTCATTCGCTCCCCAACACAAAAGCCGATAAGCTAGCATGTAGTTATCACATTTTACCGCGTAAATGAGTAAATAGTTTATATTGATACATGTACCATGTTGCTGTCTGTTTGGGCATATGTTGCCGCATTCTGAACACTTATGAATGAGATCATAGTTCTTACAACATAACCCCAAATGGGTTAATATGTCTTTGTCATGTTTTAAAAACTCGATATGATTCTTTAATGTTTTGAGCGCAATGTTAAATAAACTCAGCATTTTAATAAAATGAGGTGTTTTAGTATTATGTTTAGTATAAAATTTAGCGGTATGTTTGCATGATGCTAAATAAACTTAACGTTCCTACTAAACCAAAAAAAAATCAAATTGACTGACTCATAGAGAATTTGACGATGTTGGTAGGTAATTTTTTAACATGGTATATATTTTTTTAGGATCCGTTATATTAGGTAATAAAAGAGGACGTGCCGTTAAAGTATTTTGCTTAAGATCCTTTAAATCCTTACAAAAATATAGATTGTTCGTATGATAATGCCACTGTGTTACAGTGATGGCTTGATCAATATCAGCTCCCAAGACAAAACAGTAGTATATCGTTAAAAAGTTGTAATCTTGCATACAAGCCAACTGCATCATTTTATCGATGTCCATATGAACGATCTTTTGCTCGTATATTTCATGAAGGTCAAATACATTGTTGAAGTAAATGGCGCACATGAGTCGCCACATACTAAGGTGCCCATATGTTTGATAGAAAAAGAAGATAGCTCTTTTAAGCTTATATTTTACTGCTATGGCATACCAGTATTTAACGAATATGTTCATGGGTACATTATCCAAGATATAAAATATGAAAAGCTTTAACTCTCGATGAATCTCCTCTCCCATTTCCTGCACATTTAGAGCTTCCAACATAGGATTTTTATCAAATATTTCATGACATAAAATAATGTTATTACTCGTTTTATGACGCATTAAACCGGCGAAAATTTCCTTATTATTTAAACCATCTTTAGCTCCTAACTTTCGACACAGCTCCTGAGTTTGTTCCGTCCTAGCACAGGTCAGCCCATAATAAATGTTTGCTCCCCACTCGGTGAACAGCCTTATTACGTCATAGTTATTTTCTTTTATGGCCATGATTAATGCCACATCAAGATGAAGAAGTTCCCCCTTAAAGGGGGTTGAGCTTAAAATAACGTAATTACAGTAGTGACATAAGCTAATGGGCTTGTTTTGCCACCATAAGCCACAATATTTTAAAATATAATGATACTCCTCAGGCACGCTCTGTTTGGCCACAGCCTTTTTGGCCAGGGTTTGCAAGGAGAGCATGATAACTTCTTGAAAAAAAAACTCAAATTAAGTTCCTACTTTTTTAAAATATTAGTATGGACAGATCTACCATCATATGAAGGAATTCTTTCATCGTTAAACACTGAAGAGATAATACTTTCATCGTATAGAGAATATCATGTCAATCCATATATTGAATGTTATATATCATTAAACCCATCATTAATATAGTGTTTATGTGCTATGGACAGGTTTTTTGAATGATAATCTTTTAACATACGTTTTATAACTTCGGGATCAGTTTCTTTTAAAGATAAAGAATCATTCATGTTATAACAATTTAATGATAACATGCTGGCAATGAACGAGTTGTCTTTTTGATGCGCTAGAGTCTTTCCCTCCTCAAAGGCATTGGCGCCTAAGTCTATACAAAAGAATATGTTTCCGATATTATAGAACTGAATAGAATGAAACATGGCCTGATTGATATCAGCCCCTAAGACGACGCAACAGTAATAAATCGTTAAATAGTTATAGTTCTTGCGACAGGCCCACTTTAGCATTTCATTCATGTCTATGCGAATCCTCTCCTTTTCGTACACTTCGTGAAGTTCAAACACATTATTGTAAAAAAGGGCGCACATAAGCCGCCACCGATGTAGATGAGCATATCTCTGATAAAAATAGCAAATCGCCTCCTTAAGGTTACATTCTATTGCCATCGCGTACCAATATTTAGTAAACATCTCGCTTAATATATCGGTTTCTACCATTAATCCCTCCAGTTGTTCATAAATCATTCCCTTTACTTCAAAACGATTTATGGTATCTAAAATGGGATTATTAGAAAATACCTCATGGCAGAAAATGATGTTACTGCTAGTTAGATCACGTTTCAATGTGTAAAAAAATCGTAAAATTTCCTGGTCATTTAACTGTTCTTTGGCACCTAGCTGCCTGCACAGGTCTCGGGTGTGCTCCGTGTTGACAGAAAGCAAACCGTAGTTGATGTTTGCACCCCACTCGGTGAACAATTCTATTAGATCGTGATTGTTTTCCTCCACAGCTTTCACCAAGGCCGCGTTAAGATTTGTGCCGTTCTTAAAATACGGCGTCCATATTTTCTTTTGATGATACATGATAGGGCCATTATGCCACCATAGACCGCAGCACTTCAAAAAATGAGGATGGCATTTGGCCGGATACTGGCTGGCCAGCACCTTTTTGGTGAGAGTCTGCAGAGAGAGGACCATATTTCTTTTTTTTGAAAAAATCAAATTAAAAAAATCATGCTTGTTTAGCATACATGTAATATTGTTATAATTACGTTATAATTACGTTATAATTACGTTATAACTATATTATAACAATGGTATAACAATGGTATAACAATGTTATAACAATGTTATAACGATGTATCATTGATGTCATCATTCAACTAGGCCAACATACTTTTTAATTTATAGTTTTTTAATAGATGATATATTTTGCTAGGATCTGCTTCTTTTAACGTTAATAGCGAGGAGTCTGCACTATAAATGTCTAATGATAAATGATGAGATATCAAATAGTAATTCCGTTGCTCTGCTAGGGCCTTTGCCTCTTCAAAGGCGTCGGCTCCCAGATCTATACAAAAGAACAAGTTATCCATATTATAAAATCGTACGCAGGCAAGCATAGCTGAATTAATATTAGCTCCTAAGAGAAAACAATAATATATGGTTAAAAAATTGTTATCTTTTGTGCAGGCCATCCGCATCATTTCATCCACGTCCATGCGGATCTTTTCCTTTTCATACAAATTATGTAGGTCAAACAGCTTATTAAAACAAAGAGCACAGATTAACCACCACGTATTTAGATACTTAAAATGTTGGTAAACATAAGAAATGGCCTCCCTAAGATTATCCTGCAATGCCACTATAAAACAGTATATCGTTAACATATCACCATCCGACATATTACTTAATATGTCGGTGTCTTCTACTAACCTTTTCAACTTCCAATATATGGATGACCTTATTTCCCTTATAATGACATAGGCTGGAAAGGGATTATCATTAAAAAGTTTAAGACATAAGATAATATTACTGCTAGTAGTGCCAGGGTGTATTAATTTAAAGAACATGTGCATAATCTTCTTTTTATCCACGCGGTACTTGGCTCCTAATTCCCAGCAAAATTCTCGAACAGGCGGCGTATTGGCGCAAATTAACCCATAGTTGATGTCTGCGCCCCATTCTGTAAACAGTTTTATTAACTGATAGTTGTTTTCCTTTGTAGCCAACATTAGTGCCGTATTAAGGTCCAAGCCGTCTGCAAAGCTTGGCAGCTTTATCAGCATATGTTTGCAATCAAGGGAAATTGGGGCCTTATACCACCATAGTCCGCAGCGTTCTAAGATAACATGGTACTCAATAGATACTTGCTGTCTGGCTAGTACCTTTTTGGCGAAGGATTGTAAGGAAGGAAACATCCTGTTTCTTTTTTTTTTAAAAATCAATTATCTTTGTTCATAATCAAGAAAAATCCCCATATTTATTGAGTGATAATTTTTTAACATGCAATTTATTTTTTCAGGGTCCGTAACGATCGACAACAGAGAAATAACCGGATTGTAATGCTTTAATGATAAGGCATGGGCTATCAGATAATTTTCCTTTTGTTCTGCCAAAGCTTTGCCCTCCTCAAAGGCATCGGCACCCAGGTCTATACAAAAGAACAGGTTTCCAAGATTATAGTTTTGTATGGAAACAAGCATGGCTTGATTGATGTTGGCTCCCATGATAAAACAGTAGTAAATGGCCGAATAGCTATAATCTTGGATGCAGGCTATGTGCATCATTTCATCAATATCCATGCGGACCCTTTCTATTTCGTACAGCTCGTGAAGGTCGAACACGTTGTTGTAAAAAAGGGCGCACATGAGCCGCCACCTATGTAGACGCGGGTATTTCTGGTAAAAGTAGCGGATAGCATCTTTGAGGTCATAGTCCACCGCTATCGCGTACCAGTATTTGGTTAAAACAGTGCTAAAGCTATCATCATGGTCCAGCATGAAGGTTATCTCCATGAGCCCTCTTAACTCCCACATGATTTCCCCCCTCAGATCCAGATTATCTATAATCCTTAAATTGGGGTTATTGGAAAACACCTCGTGGCAAAAGATAATATTGCTACTGGTTTTATCGCGCGTTGTATCAAAGAAAATTTTTAAAATATACTCTCTTTCTAAATATTCTTTGGCTCCCAGCTCTTTGCACAGATCACGGGTATTTTCCGTGAGAGCACAAATCATTCCATAGTTAATATCTGCACCCCATTCAGTAAACAGCTTTATCAAGTCATGATTATTCTCCTTCACGGCTTTCATCAGTCCTATGTTTAACTCGATACCTTGACTAAAACAGGTTGACCTTATAAATAATTTATTGCGTCGAATATGAAGCATAATGGGGCCATTATGCCACCACAGGCCACAACACTTCAGGACATGATATTGATCTACCGGTATACACTGCCCGGCCAGTACTTTCTTCGTGAGGGATTGCAGGGAAGGCAACATGCCTTTCCATCCTTTGACGGAAATCAAATTATCTACTAATAACTATCAGTGTTTATATTAAGTATTTAGATATTATCCCGGGCTGGATACGTAGTATCGCTATTCACATGTACTTCCAACTCTAGCCGGAGCCTGCAGGGTCATTTATTTTTAATATTGATTCTTTTTTGTATTTAATCATTTAGAGAAGGTCATCATAGGAGCCAGATGTTCTCTCTCCAGAACTTATGTCGAAAAACATTACCTAACCGTAAACTTCCTGAATTTTTTGACGAATATATATTACAACTGCTGGGATTATACTGGGAAAACCATGGAACTATTCAACGAGCAGGAAACAACTGTGTGCTTATACAGCAACATACCCTCATTCCCGTAAATGAAGCCCTGAGAACAGCAGCATCTGAAGAAAATTATGAGATCGTGAGCCTTTTATTAGCGTGGGAGGGGAACCTTTACTATGCTATTATAGGGGCTCTAGAGGGCAACCGCCACGACTTAATTCGTAAATATGATGACCAAATCAAGGACCATCATGAAATTCTGCCATTCATTGACGATCCAGTCATATTTCACAAATGCCATATCATGCGGCAATGCTTTTTTGATTGTATTTTATATCAAGCTGTAAAATATAGTAAGTTTCGCGTTCTTCTTTACTTTAAACATAGATTAGAGGATGATTTGCCCTTCACTCATTTACTTATTGAAAAGGCATGTAAAGATCATAATTATGAAGTTATTAAATGGATATATGAAAACCTACATATCTACAATATGATAGATACCTTTGAATGTGCTATTGCCCATAAGGATCTACATCTATATTGTTTGGGGTATAGATTTATATATAACAGAATCGTACCCGATAAGTATCATCATTTAGATATTCGCATGCTTTCAAGCCTACAACTCCTACATAAGGTGGCAGCCAAAGGATACTTAGATTTTATCCTAGAAACCTTAAAGTATGATCATAATAAAGATAATATAAATATTATTCTAACACAAGCTGCAACCTATAACCATAGAAAAATTTTAATCTATTTCATTCCTCAATCAACCCACGCACAGATAGAACAATGTTTACTAGTGGCGATAAAAGCAAAATCTTCCAGGAAAACCTTGAACTTACTACTGTCTCACCTAAACCTTTCCATCAACCTCATCAAAAAAATAAGCCATTATGTTGCCACTTACAATTCAACAAATATAATAGGCATTCTGAGTATGCGGCGGAAAAAGAAGATATATTTAGATATCATATTGACAAAATTTGTAAAAAAAGCTATTTTTAATAAGTTTGTCGTTCGATGTATGGATACATTTTCTATAAACCCGGAAAGAATCCTTAAAATAGCCGCGCGAATAAATAGGATGATGTTAGTGAAAAAAATATCTGAACATGTTTGGAAAAATCATGCGGTTAGACTTAAATACCTTAAACATGCGGTACACACGATGAAGCATAAAGATGGGAAAAATAGACTCATGAACTTTATCTATGATCGCTGTTATTACCATATGCAAGGGGAAGAAATCTTTAGCCTCGCAAGATTTTATGCAATCCATCATGCACCAAAGTTGTTTGACGTTTTTTATGATTGTTGTATCCTAGATACGATACGATTCAAAAGCCTTCTTTTAGATTGTTCACATATCATAGGTAAAAACGCTCATGATGCTACCAATATCAACATCGTGAACAAGTATATCGGCAACCTGTTTGTTATGGGAGTTCTTAGCAAAAAAGAAATCTTACAGGACTATCCATCCATTTATTCTAAACAATACATGCCTTAGTTTATTTTTTTTGCGGCCGAAACATTATTCTTACCCTAGAAAACGCTTATAGTCATCTTAAATCATAGGTAAGGAAGATCATCATATTTTTTGAAACGTAATTTTTTAACGCATGATCTATGATTTCAGGGTCCGTGCTTTTAGGCAACGGGGTGGTGGCCGGACTATAAATCTTTAGGGATAAAATGTTCTTTATAAGCTCATACCCTTCCCCTAAAGCTGTAGTACCCTCTTCGAAAACATCAGCCCCCAGATCTATACAAAAGAACATGTTTTCTATATTATAGTACTGTATTGAGCTAAGCATGGCTTGATTGATGTTGGCGCCCAGGACATAGCAGTAGTACATGGTTGAAAGGTTGTGGTCTTTGATGCAGGCGATCCGCATCATCTCTTCTATGTCCATATGGATCTTGTCCTTTTCATACGCCTCATGAAGGTCAAACACATTATTAAAACAAAGAGCACATGTTAACCGCCACGTATTCAGGTGTGTATATTTTTGGTAAAAATACTGTATGGCCTCTTTCAGGTTATAGCGTATGGCTATAGCGTACCAGTATTTGAGTAGTAATGTACTGAGCGAAAACTCATTATTTAGCAGATCGGTTTTTACTATTAACTCCCTTAACTCCCAGAAAATTTCTATCCTCATTTTTATATTATTTACTTTTTGTAATATCGGATTGTTGGAAAACACCTCATGGCATAAAATAATGTTACTACTAGTTTTATGAAACTTTAGATCTATAAAAATTTGTAAAATTTCTTCTTCATTCAAGGTTTCCTTGGCACCTAGCTCTCGACAGAGGTCCCAGGTGTGCTCCGTGTTGACAGATACCAGCCCGTAGTTGATGTCCGCCCCCCACTCTGCAAACAGTTTTATAAGGTTGTAGTTGTTTTCCCTTACAGCCTTCACTAACGCCGTATTTAGGTTTAAGCCCTCTTTAATACCTGCTGATTTTATGAGCCTTAGGTTATGATCAAACGTGATCGGAGCATCATGCCACCATAGGTCATAACACTTTAAAAGATAATGTTGGTTCGTGGGCACGCATTGTCCAGCCAACACCTTTTTGGTCAGAGATTGCAGGGAAGGCAACATGTCTCTTCATCTTTTAAAAAAAAATCAAATTAATTAGCCGAATAAATTTTTCTTTCGAGGGCTTTTTAAAAGAGCTCTTTAAGAGCTCTTTAAGAGCTTTTTAAGAGATTAAAAAATTATTCTTGCTGGCATTCTGCCAAGTATGCGGCATTCCTATCATCTATAGTATATTATGAGAATATTCCCAAATGATGGATAAGTTTTTTGATTTATAATCTTTTAATAAACTGCTTATTTCTTCGGGGTCCTTTAAGTTTAGTGGCAAGGAAGCATCTGAGCTGTAAATATCCAAAGCCAAACTATGGCTCAGAAAATTATAACCTTTTTGTTCCGCTATGGCACGACCCTCTTCAAAGGCATTACCACCCAAATCTATACAGAAAAATATATTACCGATGTTATAATATTGTACTGAAGTAAGCATAGCTTGGTTGATGTTGCCCCCCAGCGCGTAACAGTAATATATTGTTAATGGATTGTTATCCTTGGTAGAAGCCAGACATATCATGTCATGGACGTCTATTTGGATGTTTTCCTTGTGGTACATCTCATGAAGCTCATATATTTTGTTATAATACAGGAGACATTTTAATCGCCATTCATTAAGATCCGTATATTTCTCATCTAGAAAACAAATGGCGTCCTTACAATCGTATTGTACTGCTTTGGCGTACCAATACTTCACTAGTAAACCATTTAACTCGTCCGTTTCTTTTATTTCTATGAGCCCCCATAGTCTTTTATAAATTAAGCCCCTTAATTGTATAACAAATTTGTTTTCTAAAATAGGATTATTCATAAAAATTTCATGGCACAAAATAATACTGCCGCTGGTTTTATTGTGCATTATCCTGGTAAAAATACGGAAAATATCGTTGTCCTCTAGAGTTTCTTTGGCGCCTAGCTGTCTACACAACTCTCGGATGTGCTTCGTATTGATAGAAAGCAAACCATAGTTGATATTTGCGCCCCACTCTGTAAAGAGCTTTATCAGACTATAGTTGTTTTCCTTAACAGCTATTATTAATGCCACACGAAGGTCTATATCTTCTCCTAAAAATCCTGATTTTATTTGTATTCGGCCACGATCCATACAAAGCTTGAGAGGAGCATCATGCCACCATAGGCCACAATATTTCAAAATGCAGTGTTCATCTATTGACAAACACTGGCTGGCTATCGTCTTTTTGACGAGGGTCTGCAGAGAGAGCGGCAACGACATGTTTCTTTTTCACCAAAAAAAAATCAAATGTTCTCGTCTTTAAAGGTTAATTCATGTTCTTAAAATGTTCATTTCATGATAGTGATTAATAATATGGTTTAATAACGCTAGAAGGCTTGTTTATAAGACAGTCATAAGCAGTCTATAAGACAGTCTATAAGCAGTCTATAAGACAGTCTATGACTTAGTCTATAACTATAATTTCTGGATGGGCTGTAAGATACTCTTCGGCTCGTTTCAGATTTTTTGAAGTATATGTCTTTAGCATATCATATATTTCCTGGGGTTCGGTTACATCTAATACCAAGGTCACATCACGGCTGAAAAGCTGCTTTACTAAGAAAATGTTGCTCAAGTTATACATATAAGCTTTGTGCGCAATGAGTTGTGCCCTATCAAAATCGGCAGCCCCCCAAATCAATACAGAAAAACATGTTTAAAGTATTATTGTTATAGATAGAAAGATTCATGCCATAATCGAGACTAGCCCCCAACCTATGACAGTAATAAATGGCCGCGTAATTTTTTTCCCGCAAGCAAGCAAATTTCATCATCAGATTAGGGCTGATGCAAATCTCTTTTTCACGACACAACTCGTGTATGTCAAAAATGTTATTAAAATAAAGGCTACAAGCTACCCGCCAATAGAGGTGATTTTTATGCCTTTTATAGAAATAGTGAATAGCCTTTGTAAAATTATGTCGTAATGCCAGGGCAAACCAAAACTTTGTTAATAGGTGGTGCGCCGTATCCCCCGTCAACGGAATGTTTGAACAGGTGTACGTAACTGTGTCTAAAGTGGTTCTAGTTACGGTTTCCAAGAGTGGATTATGACAAAACATGTCATAACCCAGCAGAACTCCTGCACAGGATTTTAGCCTGGCCACTTCTTTTAAAATTTCCAGAAGACGGGGTTCGGATACAGGCGTTAAGCCTCCCAGTTCCGCACACAGCCGCTTTAGATACACGGCAGGAACACGTATAAGCCCATATTCAGGATTTGCGCCCCAATCCACAAATAAACGTATAAGTTCAAGATTATCGCTCTTCACGGCCTTTACTAGCGCCGCTTCGAGACAAAGATCATCCTCAGAAAAACACTGTAAATGTTTATACGAAAAAACTTGCTTACAATTGTTACATAGGTGAATAGGACCTAAATCCCACCACAAACCAAAACGCTGCAACGTATAATCATAGTCACTTGAAAGATAATTGCATGCCACAACTTTTTTGGCCAACGTTTGTAAAGACAACATACTAAGTTTAAAACATCTTAAATCTAAGCTAGCTAACTTTCAAGAAAACCCTCTATCCCTAAGAATATATCTTATAACTAGACTTATAGCAGTAAAAATCAACTTTGGTTATTCTTTTTAATATAAAACGTCTAATTACTTGCAAAGGACTATAAAGCCCATTTTCCTCAGCTAGAATTTTTATTTTTTAATGAAGTAGGGGGATATGTTTTCCCTTCAAGACCTTTGCCGAAAGCATCTTTTTATTCTTCCCGATGTTTTTGGCGAGCATGTACTACAACGATTAGGACTGTATTGGAGATGTCACGGCTCCCTTCAACGCATAGGAGACGACCACATACTCATACGACGGGATCTCATCCTTTCCACCAACGAGGCCTTAAGAATGGCGGGAGAGGAAGGAAACAATGAAGTAGTAAAGCTCTTGTTACTGTGGAAGGGAAATCTTCATTACGCCGTCATAGGAGCCTTGCAGGGTGATCAATATGACCTGATCCATAAGTATGAAAACCAAATCGGCGACTTTCATTTTATCTTACCATTGATTCAAGACGCGAATACGTTTGAAAAATGCCACGCTTTAGAACGTTTTTGTGGTGTTTCATGTCTGCTAAAACATGCTACAAAATACAACATGCTCCCTATTCTCCAAAAATACCAAGAAGAGCTGTCTATGAGAGCGTATCTTCACGAAACCCTATTTGAACTAGCATGCCTATGGCAGAGGTATGATGTCCTTAAATGGATAGAGCAAACCATACATGTTTACGACCTAAAGATTATGTTTAATATTGCCATCTCCAAGAGGGATCTGACTATGTACTCCTTAGGATATATTTTCCTTTTTGATAGAGGGAACACCGAAGCTACGTTGCTAACGCAACATCTCAAGAAGACAGCGGCCAAAGGGCTCCTCCACTTTGTGCTAGAAACGTTAAAATACGGCGGCAACATAGATACCGTCCTGACCCAAGCCGTAAAGTACAATCATAGAAAACTTTTAGATTATTTTCTGCGTCAACTACCTCGTAAACATATTGAAAAACTTTTGTTGCTGGCCGTGCAGGAAAAGGCTTCTAAAAAAACATTGAACTTACTGTTGTCACATTTAAACTACTCCGTGAAACGCATCAAAAAACTACCGCGCTATGTGATAGAGTACGAGTCCACCTTGGTGATAAAGATTTTATTAAAAAAAAGAGTGAACCTGATAGATGCCATGTTGGAAAAGATGGTAAGATATTTTTCTGCGACGAAAGTGAGGACGATCATGGATGAGCTTTCGATTAGTCCGGAAAGAGTCATTAAGATGGCTATACAGAAAATGAGAACGGATATCGTAATCCATACTTCTTATGTTTGGGAGGATGATCTAGAACGTCTTACTCGTCTTAAAAATATGGTATACACCATAAAGTACGAACATGGGAAAAAAATGTTAATTAAAGTCATGCACGGCATATACAAAAACTTATTATACGGCGAAAGGGAAAAAGTCATGTTTTATTTAGCCAAGCTCTATGTTGCTCAAAACGCGGCCACCCAATTCAGAGACATTTGTAAGGACTGTTACAAACTGGATGTGGCACGGTTTAAACCGCGGTTTAAGCAACTAATATTAGACTGTTTAGAAATTATTACTAAAAAATCTTGCTATAGTATCCTGGAAATCTTAGAAAAACATATTATTTCCCTGTTTACTATGAAAGTTATGACTGAAGAAGAAAAAAACCTATGTTTAGAAATATTATATAAAGTAATTCATTATAAAACAATACAATGTTAAAATTCAATAGATATCCATCATTAATATTGATTATATTTTCGAATATTATCTTCTATGGTGCAAGATAATCATCTAGCGCGTGAAACATGTCCTCTTCTCTTCAGGAACTTTGTCGAAAAAAGCTGCCTGACTGCATACTTCCAGAGTTTTTTGACGACTATGTATTGCAACTGTTAGGACTGCACTGGCAAGATCATGGTTCCCTTCAGCGTATCGAGAAGAACCAGATACTTGTTCAACAGGAACCCATCCATATCAATGAAGCACTCAAAGTAGCAGCATCGGAAGGGAACTATGAAATCGTAGAGCTGTTGTTGTCATGGGAGGCAGATCCCCGCTACGCCGTCGTAGGAGCCCTAGAAAGCAAATACTATGACCTGGTTTACAAATACTATGACCAAGTTAAAGACTGCCATGATATCTTGCCGCTGATTCAAAATCCGGAAACATTCGAAAGATGTCATGAGTTAAACAGCACCTGTTCACTGAAATGCTTATTCAAGCATGCTGTGATAAATGACATGCTGCCGATTCTTCAAAAATATACAGACTATCTGGATAGGTGGGAGTATTGCAGCCAGATGCTGTTCGAACTGGCATGTAGTAAAAAAAAATATGAGATGGTTGTGTGGATAGAGGGAGTTCTAGGCGTCGGCAAAGTTACATCTCTTTTCACCATTGCGATTAGCAACAGAGACCTACAGCTGTATTCTCTGGGCTACTCAATTATCCTTGAGAATTTGTACTCCTGTGGACAGGACCCCAAGTTTTTACTAAATCATTTCCTGCGAGACGTTTCAATAAAAGGGCTTCTACCCTTTGTAATCAAAACCATAGAATATGGTGGAAGCAAGGAGATAGCCATAACTCTGGCTAAAAAATATCAGCATAAACATATTTTGAAATACTTCGAAACCTGGGAAAGCTAGGTTCAGTATGGTGTACTCACTATTGTAGTGAATCGTATCCTGTAAATTTTGTAAAAAAGCTTAAACTTTTGACCACATCATATTGTTTTAGAAATCTCAAACCAGTGAACAACAGTCTTATCATACATTAAAATTCCAGTAAAATTTATATTTTTTTTGGTAAACAAATGTTTTCTCTTCAAGACATCTGTCGGAAACATCTTTTTCAACTTCCTGACGCTTTTGATGAATATATATTACAAGCGCTAGGACTATACTGGGAAAAACACGGATCTCTTCAACGAATAAGAAAGGACGCTGTGTTTGTACAGCGAAACATCGTCCTTTCTACCAATGAGGCCCTGAGAATCGCAGCCTCAGAGGGAAACGAAAGGGTAATAAAACTTCTGTTATCATGGGAGGGAAATTTTCATTATGTGATCATAGGAGCTCTAGAGGGTGACCAATATGACCTAATTCATAAGTATGATAGTCAAATTAAAGACTACCACATGATTTTATCATTGATCCAAAATGCAAATACCTTTGAAAAGTGTCATCAGTTATCCAATAGTAATATGTGGTGTCTTATACAGAATGCTATAAAATATAATATGCTCCCTATTCTCCAAAAACACAGAAATATTCTGACACATGAGGGAGAGAATCAGGAATTGTTTGAGATGGCATGTGAGGAACAGAAATATGACATAGTTTTATGGATAGGACAAACCCTAATGTTAAATGAGCCGGAGTTTATTTTTGATATCGCCTTCGAACGGATAGATTTTTCTTTATTAACAATGGGTTATAGCCTTCTTTTTGATAACAAGATGAGTAGTATAGACATTCATGATGAAGAAGATCTTACTTCATTACCAACAGAACACCTCGAAAAAGCAGCCACTAAGGGATGTTTCTTCTTTATGCTAGAAACTTTAAAACATGGTGGAAATGTAAATATGGCAGTCTTATCTAAAGCTGTTGAGTATAATCATAGAAAAATTTTAGACCATTTTATTCGGCGGCAAAAATGTTTATCACGTGAAGAGATTGAAAACCTATTATTAACCGCCATAACCAATTGTGCATCCATAAAAACGTTAAACTTACTCTTGTCTTACCTAAACTATTCCGTAAAAAATATCATTGGAAAAATAGTACAACATGTCATAAAAGATGGTGATTATACCATCATATTACTTTTAAAAAAAAAGAAAATAAACCTAGTGGAACCTGTTTTAACAGGTTTTATAGATTATTACTATAGCTATTGTTTTATAAAACATTTTATCCAAGAGTTTGCTATTCGTCCGGAAAAACTGATTAAAATGGCCGCGCGAAAAGGTAAACTAAATATGATTATCGAATTCCTTAACGAAAAATATGTTCATAAAGATGATCTTGGAACTATATTTAAATATCTCAAAACCCTAGTATGTACCATGAAACATAAAAAAGGAAAAGAGACATTAATTGTTCTTATTCATAAAATATATCAAGATATTCATCTGGAGACTAAAGAAAAATTTAAATTATTAAGATTTTATGTCATGCATGATGCAACTATCCAATTTCTATCTATGTGCAAAGACTGTTTTAATTTAGCCGGTTTTAAACCATTTGTTTTAGAATGTTTGGATATTGCTATTAAAAAAAATTACCCTGATATGATACAATATATAGAAATTCTATCGAAATCTGAGTAAAATTTATTTTTTTGATCAGAGTAAGAAAATGTTCTCCCTCCAGGAGATCTGTCGAAAGAACATCTACTTTCTACCTGACTGGCTCGGTGAGCATGTGATTCAGCGACTAGGTCTGTACTGGGAAAAACATGGTTCTCTTCAGCGAATCGGAGACAACTATGTACTTATACAACAGGACCTCATCATCCCCATCAATGAAGCCCTAAGAATGGCAGGGGAGGAGGGGAATGATGAGGTGGTACAACTCCTATTACTATGGGAGGGAAACATTCATTATGCCATCATAGGAGCTTTGGAGAGTGACCATTATAGCCTAATACGTAAGCTCTATGACCAAATCGAAGACTGTCACGACATCCTTCCCTTGATTCAAGACCCAAAACTCTTTGAAAAATGCCATGAATTAGATAAATCTTGTAACATTTTATGTCTCGTATTACACGCCGTAAAAAACGATATGCTTTGCATTCTTCAAGAGTATAAAATGCATCTAAGTGGAGAGGATATTCAAGTGGTGTTTGAAACAGCATGCCGTTCACAAAAAAACGATATTGTGTCATGGATGGGACAAAATATTGCAATATACAACTCCGGAGTTATTTTTGATATTGCCTTTGATAAGATGAATGTGTCCTTATTATCTATAGGGTACACGCTTCTTTTCAATCATCATATAAATAATACGAACGAAAATATTAATTCTTTATTGACACAACATCTTGAATGGGCTGCCGGCATGGGCCTTCTTCATTTTATGCTGGAAACTTTAAAGTATGGCGGGGATGTAACGATAATAGTTTTGTCTGAGGCCGTAAAATATGACCACAGAAAGATTTTAGATTATTTTCTCCGTCGAAAAAACTTGTACCAAGAAGATCTTGAAGAACTATTATTGTTGGCGATACGTGCAGATTGTTCTAAAAAGACCTTAAACTTGTTATTATCTTACTTAAACTATTCCATAAACAATATCCGTAAAAAAATATTACAATGTGTAAAAGAATATGAAACGACCGTTATTATAAAAATTCTATGGAAAAGAAAGATAAATCTGATAGAGCCCATTTTGGCAGACTTTATAGGATATCATAGCTATACCTATATGGTAGATTTTATGCGCGAGTTTTCCATCCATCCGGAAAAAATGATCAAAATGGCTGCGCGAGAATCGAGGGAGGACTTAATCATAAAATTTTCCAAAAAAGTTTGCAAAGAGCCTAAAGATAGACTTCACTATCTCAAAAGCTTAGTGTATACTATGCGACATAAAGAAGGCAAACAACTGTTAATTTATACAATCCATAACTTATACAAAGCTTGTCATCTAGAGAGTAAAGAAATGTTTAATTTGGCACGATTTTATGCACGGCATAATGCAGTGATCCAGTTCAAATCGATTTGTCACGATCTCTCCAAGCTGAATATTAATATCAAAAACTTGTTGTTAGAATGTTTAGGTATTGCTATTAAAAAAAATTACTTTCAACTTATCAAAACAATAGAAACGGATATGCGTTATGAGTAAAATTTTTGGATAAGGGAAGATTCTACCAAACTAACTAAGACCTTTCGCTAGAATGTATCTTATTGTTAATATAGATTAGGTATGACGTGAAAAAATAGATTAGGTAGGTTGTGAAAAACAGATTAAACTTAAAATTATGTGTATTATGTAAAATTTTAGAAATAAAAATTTATTTTTTTTATTGAGGGTACGGAAAATGTTCTCCCTACAGGACCTCTGTCGGAAGAACACTTTCTTCCTTCCAAATGATTTTAGCAAGCATACTCTACAACGGCTGGGGTTGTATTGGAAAGAGCATGGATCCGTCCATCGAATAGAAAAGGACAGCATAATGATACAGAATGAATTGGTTCTTTCTATCAATGATGCTTTACAGCTTGCAGGAGAGGAGGGGGACACAGATGTGGTACAGCTCTTGTTACTATGGGAGGGAAATCTGCATTATGCCATCATAGGAGCTTTGAAGACTGAGAATTATAATCTAGTATGTGAGTACCATAGCCAAATTCAGGACTGGCATATTCTCCTACCCTTGATTCAAGATCCAGAAACATTCGAAAAATGTCATGATTTAAGCCTTGGATGTGACCTTATATGCCTTCTCCAACATGCTGTAAAATGTGATATGCTTTCTATTCTTGTTAAATATAAGGAGGATTTACTAAATGTAAGGATTAGGCATCGTACCCAATCCCTGTTTGTTTTGGCATGCGAAAATCGGAGATTTGAGATTATTGAATGGATAGGTCAAAATCTGTCAATTCCTGAACCTGAGGCCATTTTTAGCATTGCTATTGTTACAAAAGATGTAGAACTGTTTTCCTTAGGATATAAAATTATTTTTGATTACATGCAAAGACAAGGAATTTTTCAATTAACCAATGTAGTTCGCATGCTTCTGCTAAATCGTCATATTGGTATGGCAATAGAAAAAGGACTTTTACCTTTTATTCTGGAAACTTTAAAATATGGTGGTAGTGTAAAAAGAGCTTTATCTTATGCAGTAATAGATAATAAAAGAAAAATTATAGACTATCTTGTACGCCATGAAAATATACCCCGAGGAACTATTGAAAGACTTTTGCATCTAGCTGTGAAAAAACAATCTTCCAGGAAAACTTTGAACTTGTTGCTATCTTACATAAATTACAAGGTGAAAAATGTTAAAAAGCTGGTAGAGCATGTAGTAGATCACAAATCCACTCTTGTGTTAAAAATTTTATTGGAAAAAAAGGAAAATCTAGTGGATGCTGTTTTAACAAGACTTGTAAAACATTCTACATATTTCCAGGTGAGAGAATTTATCCAGGAGTTTTCCATCAGCCCAGAAAAATTCATTAAAATAGCTGTGCGGGAAAAGAAAAATGTGTTAATCGAGGCTATTTCTGAAGATATTTGGGAAAATCCCACAGAAAGAATTACTTATCTCAAACAGATAGTGCACACCATAAAATATGAAAGTGGAAGGCGGTTTTTGATAGACATCATTCACAGCATTTACCAAAGTTACTCACTAAAACACGAAGATATTCTTAAACTGGCAACATTTTATGTCAAACACAATGCAATCACCCATTTTAAAGATCTCTGCAAATATCTTTGGCTGAACAGAGGAACAGAAAGTAAGAAACTGTTTTTAGAGTGCTTGGAAATTGCTGATGAGAAGGAGTTTCCTGATATTAAAAGTATTGTGAGTGAATATATTAACTACTTGTTTACTGCAGGAGCTATTACCAAGGAAGAAATCATGCAAGCTTATGCTTTAGAGTATGACATGTATTAAATTTCTGAAGGTAGACTAAAATATACTATATATTAAAAAATCCAAAACAGCCATTTTTAACTAACTTCTTCTTAAAAACTCTGGATAAAAATTTATTTTTTTTAATTTGAGTAGGGAAAATGTTCTCCCTTCAGGACCTCTGTCGGAAGAACACCTTCTTCCTTCCAGATAATTTTAGCAAGCATACCCTGTATTTGCTGGGGTTATACTGGAAGGGACATGGATCTATCCAAAGAACAATGAATGTTGGTGTACTGATAGAGCATAATCTTAATCTTTCCATCAATGAAGCCTTAATCCTTGCAGGAGAAGAGGGAAACAATGATGTAGTACAACTCTTATTGCTATGGGAAGGAAATCTTCATTATGCCATCATAGGAGCTTTGAAGACTGAGAAATATGGCTTAATATGTGAGTACCATAGCCAAATTCAGGACTGGCATGTTCTCCTCCCCTTGATTCAAGATCCAGAAACATTCGAAAAATGTCATGATTTAAGCCTTGAATGTGATCTTTCATGCCTTCTCCAACATGCTGTAAAATATAACATGCTTTCTATTCTTGTTAAATATAAAGAGGATCTATTAAATGTACTATTTAGGCAACAAATTCAAGGACTATTTATTTTAGCATGTGAACATCGGAGGATTGAGATTCTTACGTGGATGGGTCAAAATCTGCCAATTCCTGATCCTGAGCCTATTTTTAGCATTGCTGTTGTCACAAAAGATTTAGAAATGTTTTCCTTAGGGTACAAGATTGTTTTTGAATACATGGAAAATCAAGGACTATTTCATTTAACCCAGGTAGTTCGTATGGTTATGCTAAATCATCACCTTGGCATGGTAATAAATAAAGGACTTTTACCCTTTGTGCTGGAAACTTTAAAACATGGTGGGAATGTAAATAGAGCCTTATCTTATGCTGTCACACAAAACAAAAGAAAGATTTTAGACCATGTTGTTCGCCAAAAGAATATACCCCATAAAACCATTGAAAGAATGTTGCATCTGGCTGTAAAAAAGCATGCTCCCAGGAAAACTCTGAACTTGTTACTATCTTACATAAATTACAAGGTGAAAAATGTTAAAAAGTTGTTAGAACATGTAGTGAAATACAACTCTACTCTTGTGATAAGAATCTTGTTAGAAAAAAAGAAAAACCTGCTGGATGCTACTTTGACAAGATATGTCAAAGATTCTACATACTTTCAGGTGAAAGAATTTATGCAAGACTTCTCCATCAGCCCAGAAAAATTCATTAAAATAGCTGTGCGGGAAAAGAGGAATGTGTTGATCAAGGGTATTTCTGAAGATATTTGGGAAAATCCCGCGGAAAGAATCAGGAATCTTAAGCAGATAGTGTGTACCATAAAATATGAAAGTGGAAGACAATTCCTGATAAATATCATTCACACCATTTACCAGAGTTATTCTTTGAAACCTGAAGAAATTCTTAAACTGGCAACATTTTATGTCAAACACAATGCAACCACCCATTTTAAAGATCTCTGCAAATATCTTTGGCTGAACAGAGGAACAGAAAGTAAGAAACTGTTTTTAGAGTGCTTGGAAATTGCTGATGAGAAGGAGTTTCCTGATATTAAAAGTATTGTGAGTGAATATATTAACTACTTGTTTACTGCAGGAGCTATTACCAAGGAAGAAATCATGCAAGTCTATGCTTTGGAGTATGCCATGTATTAAATTTCTGAATAAGTAAGCAATAGATAGGTTTTAGAATATGCTGTATTAAGTTAGTTTCTGAATAAGTAATTAATAGATAGATTTTAGTTTATGTAAAAATGTTAACATTTGTTAATAAGTTTTAGATAATTACTATTTTAGAGTTACTATTTTAGATTTTACCATTTTAGCTATTATTATCTTAAATAATCACTATTTTAGATAGGTCCCCGTATTAAAAACCAAATTAACCATTATCTATGTTTTTAATAATACTTTTTAAAAACCCTCCATAAAAATTTATTTTTTTTTCATAAAAGTAGAGAAAATGTTCTCCCTACAGGATCTCTGTCGGAAGAACCTTTTTCTTCCACTTGAGCCCTTAGGCAAGCATGTGGTTCAACGGCTGGGATTATACTGGGAAGGCCATGGTTCACTTAAACGAGTGGGTGATTGCTTTATATGTGTAGACAAGATTTGGATCCTATCCATCCATAAGGCTATACAAATTGCAGCCTCGGAAGGAAATGAGAACATTGTCAAGCTTTTCTTACTGTGGAAGGGGAGTCTACAATATGCCATCATAGGAGCCTTAGAGGGCAGGCAATATGATCTGATTCAAAAATATTACAACCAAATTGGGGACTGCCATGAGATTCTACCACTGATTCAAGATCCAGAAATTTACGAAAGATGCCATGAATTAAATGTTACATGTACCTTTCAATGCTTATTTCAACATGCTATAAGAGATAACATGCTGCCCATTTTCCAAAAATATGGAGAAGATCTGAATGGAAACAGAAGAATGGTTCAACTTCTATATGAAATGGCATGCCGATTACAAAATTATGATATCATCAAATGGATAGGATTTAACCTGCATGTTTATAACTTGGAAGCCATTTTTAGCATTGCTTTTGTTAGAAAGGATTTAACTTTGTATTCTTTAGGCTACATGCTTCTTCTGGGTAGAATGAGTACTGAAGATAGAAACTTTATTTCAATCATAACACGCCATCTTGAATACGCATCAAAAAAGGGACTTTTTGACTTTGTACTAGAATCTTTGAAATACGGAGGTCAAGTGGATACAGTGTTGTTTCAGGCTGTAAAATACAACCATAGAAAAATTTTGGCCCATTTTATTCATGAAATTCCGCGTGAAACAGTTGAAAAGCTGATACTCCATGCTGTAGAATCGCGGGCCTCCAGGAAAACATTCAACCTGCTTTTATCTTCCATAAACTACTGTGTAAACCCTTTTGTCAAAAAACTACTGCACACCGTGGTGAAACACAAGTACATGCTTATCATAAAGCTTTTGCTCGAGCGGCCCAAAAAGAAGATAAACCTGGTAGATGCTGCTCTATTCAAACTTGTAAAATACTCTACTTATGCAGAAATAGTAAAATTCATGAAAGAGTTTTCTGTGGACCCAGAAAGGGTGGTCAAAATGGCAGCACGACTCATGAGAGTGGACCTGATTAAAAAGATTTCTAACGATGCATGGGAAAATAAACTAGAGAGAATCAAGCACCTTAAACAGATGGTAAATACCATGAACCACAGAAATGGAAAAAATCTATTGATGTACAATATTCACAATATTACTGGATATACCTGCTTGAACACCAAAGAAGCATTTAACTTAACAAGATTTTATGCTGTCCACAATGCAACATGTTTGTTTAAAGAAATGTGTAAAAGCTGTTTTGTACATGATAAAATACAGTTCAGAGAATTGCTTGAAGATTGTTTACATATTGCTAATAGGCATGATTATATCCAGATTGCAGAAACCGCAGATGAATGTATCAAATATATAGATCTTATTACACCTAAGTAAATCATGAAAATATATCAAGTAAATCCAGATTAAATCAGGCTAATTGTAAATAGTTGTAGATACCATATAATGAATGTTTTATTAGGATAGTAGTTAATAGTTTAGTTAAGACAGTAGTTCTTTCTGTTAAGATAGTAGTTCTGTTAAGATAGTAGTTTAGTTATGATAGTGGTTTAGTTAAGACAATAGTTTTGTTAAGACAGTAGTTCTGTTAAGTCAATAGTTCAGTTAAGTCAATAGTTTTGTTAAGTCAATAGTTCTGCTAATACATTAGTTCTGTTAAGATAATAAAAATTTATTTTTTTTCATCAAGGTAGAGAAAATGTTCTCCCTTCAGGAGCTCTGCCGGAAGAACATTTACATTCTTCCTTACCCCTTGGGTAAGCATGTACTTCAACAACTAGGGCTGTACTGGAAGGGACATGGATCTCTTCAACGAATCGGAGATGACCATGTACTCTTACAACAGGACCTGATCTTTTCCATCAATGAGGCCTTAAGAATGGCGGCAGAAGAAGGAAACAATGAAGTAGTAAAGCTCTTGTTACTGTGGGAGGGAAACCTTCATTATGCCATCATAGGAGCTTTAGAGGGCGACCGATACGACCTTATCCATAAATATTATGAACAAATTGGGGACTGCCACAAGATTCTTCCTTTAATCCAAGACCCGCAAATCTTTGAAAAATGCCATGAATTGAGTAACTCCTGTAACATTCGATGCCTTTTAGAACATGCAGTAAAACACAACATGCTTTCTATTCTTCAAAAACATAAGGATCAAATAAGATTACACATGGCATTAACCCAAATACTATTTGAATTGGCGTGCCATGAACGTAAGAATGACATCATTAGATGGATCGGTTATTCCCTGCACATATACCATCTAGAGACTATTTTTGATGTTGCATTCGCCCATAAAAATTTATCCTTATACGTTTTAGGGTATGAACTTCTCATGCACAAAGTAAATACAGAGGCTGCAAATATAGATTTACCCAATTTGCTATCATATCACCTTCGAACTGCGGCGGCAGGAGGTCTTCTTAATTTTATGTTAGAAACAATAAAGCATGGTGGGTGTGTGGATAAAACCGTTCTATCCGCGGCTATCAGGTACAAGCATAGGAAAATTGTGGCTCATTTTATTCATCAGGTTCCCCGTAAAACCGTTAAAAAACTGCTACTCTATGCTGTGCAGGCTCGGGCCCCCAAAAAAACACTAAATCTACTTTTATCTTCCTTAAACTATGCCGTGCACACCATCACCAAACAACTCGTACACAATGTCATCAACTACAGTTCCACGCTTGTCGTAAAGCTTTTACTCATGCGGCGAAAAAGGAAGTTAAACCTAGTAGATGCCGTTTTAGCCAGACTTGTAAAATATTCCACCTATACAGACATTGTACAATTCATGGGTGAGTTTTCTGTGAGCCCAGAAAGGGTGATCAAAATGGCTGCACGGGAATCCAGGACCTTTCTGATTGAAATGATCTCCAAAGCTGCTTGGGGAAATCACCCACAGACGTTGATTCATCATCTCAAACACCTAACCAATACCATGAAGCCTCAATCTGGAAAAGACCTCATCATATATACCATCCACTATATTTATCTAAACTCTAATATGCTGGTAGCGGAGGAGGAAAAAAATATTTTTAAATTAGCAAAATTTTATGCGAATCATAACGCGGTAAACAGGTTTAAACAAATTTGTGAAGACTATTATATGTTAGATGCACGATTTAAAACACTTATTTTAGAATGTTTTGAAATTGCCGTCCAGAAAAACTATCCTAGAATTGCAAATATTGTGGATGACTATATTCGATTCCTTTTTTACAGGGGAAATATAACCGAGGAAGAAATTCGTGAAGCCTATTCTTTAAAAGATGCTGAGGTTTATGTAGATTTAAAATGGTTACAACAAGGAGAAATGGTTTAAACTAAATCCGGTTTAAACTAAATTCGGTTTAAACTACATTTGGTTTATCATTAGTCATTGAAACCATCGAAAAAAAGTATTTGTTTATCCCCATAAACTCATCTTTTTTTTGTCTCAAAGTTTGACACTAAAATTCAGTGTTTTATAGTGTTTATAATTAAGTGTTTTGCATGCATTGCAGAAATTTTCATCTTTTTTAATTGGTTCAATACCACATGTCATACAATATGTTGTTTGATTATCAAGATTAACTTTATGAAAGGAAAGTAAGTGAGCCGCAAATTTAAAAGTAAAATATCTTTCATTTAAAATGATCTTATGAATGTATTTTCGATAAGGAGGAATAAAAGCATTTGCCAAAATAAATCGCATAAAGGGCTTGGAAAAACCCATATCTTCTAATCTTTTGTGGGTATAAACCCTATTTTTGTGTTTTACAAAAACTTCATTGTTATAATAGTCGTTATAGCTATCAATCATTTTTTTAAGTCCTATAATGCCCAAGGTTGCACGCATAAAGCCGCAGTTTCTACTCCAAAAGCCATGCACCTGTAAAGGGTGCTTTTCATATAACCAATTACAAAATTTCATTCCGCAACAGTAGCATGTTATTTCAGTGGGGGATGTATAGAAAAATCCGGCATTCGAAAATTTTTCATAATTTTTTATGCCATGGATTGCGAAGCTTTGATTTCGTGCATCTATGGAGCTATAGCCTACATATTTAGGTTTTACTTCAAATAATCGCAAAGAGATGTATGGATCTATTGTATTTATTTTAGGAAACATTTCATAATTTTAAATTCTTATATATAATATAAAAAAAATTACAAACATTTGTAATGATCATCCTCAATTGAAAGCTGAGTTGTAGACTTTATTTTTCTAATTATACGAAGAAGGTAGGTTCTCATAAAGCCTTCAAGATGACTATTGATGTTTCCAATACATTTTCTCAATGAGTTCATAAACCCAGACATTTTGCTAATGGCTTGGCAAAGTGCCAACAAGTTGTCCACAAAGTACTGGTAGATTGCCTACTAGCTATAGCTAGCTATAGTGAGCCAACCTCTCTGTATTTATTTTATATATTTCATTTTTTAATAGATTTAATATTTTTATAAAAAAATATTTAGTTTTTTATACAAGAATGTCGACAAAAAAAAAGCCCACAATTACCAAGCAAGAGCTTTACTCCTTAGTAGCGGCAGATACCCAGTTAAATAAAGCATTGATTGAAAGAATCTTTACAAGCCAGCAAAAAATAATCCAAAATGCTTTAAAGCACAATCAAGAAGTTATTATACCACCCGGAATCAAGTTCACCGTCGTTACAGTGAAAGCTAAACCTGCTCGCCAGGGCCATAACCCCGCAACAGGAGAGCCTATTCAAATTAAAGCCAAGCCTGAACATAAAGCCGTAAAGATACGAGCATTGAAACCTGTTCATGATATGTTAAATTAAATAATAAAGCCATCATCATCGTCGTATTCTTCTTTATCATTATTATCTTCAATACATTTTTGCCAATCGAAATCGAATAAATTCAGATCCTGGACATTTAAATACTTATCATCGTACATTTTAATATAATTTAAACATGAGTTGTTGTCAAAAACTTTTATCGTTTTTGTTAAAATCATTGTATGAATAATTTCCTTATTAAGAGTTGCCGGAATAATACAAAGCCTATTTTTAGGTACATCATTCATGATAATAGTAAAATTAGTAAAAATTGTTTCTTGTTTTTCTTTTGTTTCAAATAAACGTTGTAAGGTTAAAGGTTTTTCATTCAATGGTTTCTTTGAAGATAAAAAGAATGTATAATCCGGTTTAAAGATATTTTTGGTTTCAATCGTGATTCCATCTGCTTGAGCATATGCTAAACCAGACCAAATATAATGGTCCACTATTACAATAAAATTTAGTTTAAGTAGTGCTGCAATTTCTGCGCTAAATTCACTATGATGTTTTGTAAATAATTTATGCAATTGTTCCGATGACATTTCTGTCATTTTATTTAACACCTGCAATATAAGGCCACCGGTGGTCGTGTCTGGATTAGGAAAACGTATACATACAGCATTATAATCCATGCATTCTAATGCCTTTTTTAATCTCATTGCCTGTGTGCTTTTTCCCACACCATTGATTCCCTCGATGGTAATAAGTATTCCACGCATGATTAATAAATGGGTAAAAAGAGTTCAGTTTTTAACATTTCTTACAAATCTATTTTTATACAACATTGTACAACACTACATTAACCGCATACGATGTTATAGCTTCATTAAATATTTGCTTTTATATAATCTTTACCGACCTATATTTGGCAGATCACTGCAGATGGCCATAACTAAGATAAAAATTATTTCAGATGCTACTGCGGCAGTATTATTAAAATCATGTGCGGCAATGTATGACGTCTTAATAGATAAAAAATTTAAGTAAAACAAATTTGAATAAAAAAAATAATAGTTATGATGGCGTTGTTACACAAAGAAAAGCTTATAGAGTGCATCGAAAATGAGGTGCTTAGCGGTGGTACAGTATTGCTCCTAGTAAAGAATATTGTTGTGTCAGAAATTTCATACATTGACAATAGTTATAAATATTTTACCTTTAATGCCAATCATGATCTGAAAAGCAAAGAAGATCTTAAAGGAGCAACATCCAACAACATTGCTAAAATGATTTATAATTGGATTATAAAAAATCCTCAAAATAATAAGATTTGGAGTGGTGAGCCGCGCACTCAAATTTATTTTGAAAATGATTTATATCATACGAATTATAATCATGAATGTATAAAAGATTTTTGGGATGTTTCAACCTCAGTCGGTCCTTGTATCTTTAATGATCGTAGCATTTGGTGTACTAAATGCACATCCTTTTACCCATTTACCAACATTATATCGCCCAATATATTCCAATAAATTAGATATCTTTTACTACTATTAAAATAGTTAATAACCTTATAGGATAATTAGGTACTTTATTACGATGAATTAGTTTATGTTATTTTATAATTAGGTACTTTATTACGATGAATTAGTTTATGTTATTTTATAATTAGTTACTTTATTACGATGAATCTTTATTAACGATTCTTTTATTAATGAATTATCATAAGATAAATAATTATTTTTTTCTCCATATATCGCATAATAGGTCTGGTATGGGCTAAAAGTATGTTTCAAACTATTTACAATAGAATTTCTGTTAAGAAAACATACATAATTTGAATAAAATTTTTTTAAATATCACCGAAACATCCAACATGGTGTTAATAGAGTTTTTAACAGGTTTCTTCTATTTATATGGAAAGAGACTGTTTTCCATTAGCAAAGTCATGGACATGATATGTCTAGACTATTATACCATTATTCCTGCTCCTCTGGCGATGATGTTAGCGGCAAGAATAAAAAACTATGACCTCATGAAACGACTGCACGAATGGGAAATCTCTGTTGACTACGCTCTACTTGTAGTAGATGATGTGCCGAGTATCGACTTTTGCTTAAGTCTTGGCGCTAAATCCCCGACTAGAGCACAAAAAAGACAACTGTTGAGGGACAACACGTTTAATCCCGTGTATAAGTATCTTATGAACTGTTCCGGTTTTCCAACAAGAAGAGAAAAAAACATTCCTTGTGATGTTCAATGCGAAAGACTGCAAAAAAACATTATAAAAGAACTGGTATTCAACTGCTCCGTGCTGCTTGAAATGGTACTGCACACAGAAAGAGAATATGCATACGCCCTACACTGTGCTGCAAAACATAACCAATTGCCCATCCTCATGTATTGTTGGCAACAATCCACAGACGCGGAATCCATTTTGTTGAAAACCTGCTGTTCTGATAAGAACATCAATTGTTTTAATTATTGTATTCTATATGGCGGCGCCCAGAATTTGAATGCTGCAATGGTGGAAGCGGCAAAGCATGATGCCCGGATGCTGATAAACTACTGTGTCATGCTTGGTGGAAGATCCTTAAACGAAGCAAAAGAAACGGCTGCCATGTTTGGACACATTGAATGCGCACAACACTGTTTTGAACTGCAGTCTTATGTCATGGACGCATTGAATGCGGACGACGCTGATTAAAGCGACAATCTTACGTCATGAATGACTGTCTTTTGAGTATCTATACTTACATTATATTTTTTTATGAAAAAAATATAAAGGTTGTATACAAAGGTATACAAGAAATTTGGATCATTAAACAATAATTAATTTAGACACAGGAAAACGATCTAGATCGATCAAAAAGCTATTTTTTTGCACACAGAATATTTAGATAATTAAGAGATTACTTTCCATACTTGTTCAGTTTTTTTACACACAGGAAGTTTGGATTCTGTTCAGGAAGTTTTTCATAGACATTATTTTTACAGCCAGTAATAATAATTTTGGGCTTTTTCTTAAACCACCGGTGGAAAACATCCAGCTTGTAAAGAGGGAAATGCATGTAGAGAGGTTTTTGGTAGTCATGGTTAAGAGATTTGACTAACTCCATATTTCCTGTAAAGACTGCCCAGTCCCAAGCAGTAAAGCCTCTATGATAGTCTTTTTGAGTCGGATCTGCTCCAAATTTTATGAGAGAAAGCACATTTGAAGTACGGCCCCGTATTGCGGCCTTCATCACAGGAGTCATCCCATTAAAATTCGGTAAACAAATTTTGGTCCCATTTTTTCCGAAATAGCCCAACACCTCTTCCAGGATTAAATGATTTTTTTTCTCAGCTAAATAATGTAAAGCAGAGTTTCCATCTTTATCCCTCCTATGAGGGTTAATTATTTCTCCGGGATAAGATTCTTGTTCAAAAAGAAATTTTAAAAAGTCTATACGTCCGTAGATGCATATCCACATGAATACCGAGGATCCATTTTTATCGCATCTATTGACAATCCACGGATCTGTTTTAAAAAATTCCTCAAATAGTGTAAGATTCCCATTTCTGATATGTTTTTTAATCCATTTAACAAACAAGTTTTCTATCTCCCTTTCTGGAAACATGTGTTCCATTTTGAATGTCGCCCCACCCCATTATATAATTTTACTCCTTTAATTTTTAATGTCCTTTTTTTTCGGACTTCTTTGGATAAGCTGTTTATTACCATCTTTAAATGCCTTATAGCGGGGAGGAGCCAGGCCGCTTTCCCATATGTGCGGTAATTCTTGGTGTTTATGCTTGCCTTTGGCATATCCAGGCCAGTATTTTTCGATATATTCGGGATTTGTTTTTACATATTCTTTAAAGGTCCGATAGGCTTCTTGAATACAGGTAGGCTCGCCTGTATAATTTCCGTGTTCATCTTCCTTTAAAAAGCCATTAACCCTGTCCTTTCTCCACTTAAGATTGTGCTTTCCAAAAATACGGTCAAGATCTTCGGCCTGCTGGGGTGGGATCATAAACCCCTTTTTAGGTCGAAGCTTTTTATTTTTTCCATAGCTTCGGCCATCGCGTTGCGAAACAGTGGTTAGGACGCCCGACAGCCTTTCCATGGGCGTCGCGTCCAATCCTATCCATCCACCCTGATGAATATCAATGGCAACAAGCTCTCCTTTATTTTGGGCAAGCCAAGTTTCCAAGAATGCCATGCTTTCTTCCCAGGGATAAGGCCCGCCAACACCACGGGTTGTCCAATCTTGCAAGGACTCCAGGTCCGACACCTGGTAAGGCTCTAAAGAAGACGGTTCCTTGTTTTTGTACTGCAAATAGGATTTAATGACCCATTTATACCATGTGTCGAACCGCAGCGTGGCGCCTCCAAAGTGAAAGCCGTCGTTGATTTTAGGATATCTGCAACATATTTCAACCGTACGTTTGAGTTCTGCAAAGGCGGCCTTCCAAGGAAGTCTCTCGCTGCGGGTAAGGCGGTCTATTTTGCCCTGCGTACCATAGCGTATGGCGTGACGTGCCAATTGCAACAATTCTGACACCGATCCGTGGGCCCCGATCCAGTTTATCGGATAGGCAACCTCTGAAGGGTTTAAAAGATGCTCGTAAAAGCGTGGATCTTCAGACGCCAAGGCGTCTGCAAAAGGGATAATACTAGAAAACCTGTCTAGGCATACGTTTTCTGTGTTTACTTCTAAAGGTAGAAAAATGGTTGCGTGAGGCTTTTGAACCTGCTTGTTCAGCGGTCTGCATATGCTTTGAATAATGTCTCTAGGACTATGACGCGGCGCTGCAAAAAATACCGCGTTTAGTTCCGGAACCTCTACGCCCTCTTGAAAGAGTCGACAGTTTAATAAAATAACGGGTTCCTTTGAGGAACAAAATTCTGTAAATGTTTTGAGGATAACCTGTCGCGGCAGGGTTGAGTGAGCTATCAGGGCATAGACCCCTTGGTCTACCAACTCCGCGTATAGCTCCCTGGCCTGTTTAATATCACGGGTGAATACCAGCATTTTAGGAGCCGGTATATTGGTTTTTAAATAGGCTAAGGCCATTATAATTTGCTTTACTATGATCTGTTTCGTGGTCTCCTCTCTGTTACTCGGTTGGTGAGCCAATTTGGGCGCGGCTACCATTTGTAATTCAAAATCATTTACATAGCCGGCCTCTATGCCTTCTCGCAGATAGTAGCGAAAGGCAACGCCGCCAAAAAGTTCACGATTTTTCATGGAAAGCGGGGTGTCGTACCTGGGCGTTGCCGTTAAAAAAAGTCGGTGCCCTTTTTTAAAGTTGAGCAACACATGGGTAAAGGGCCGTGTCTCCCATTCACCGCAAATCCGGTGACATTCATCGCTAATAATAAGATCGAAATCATCCACCAGTAGCGTGGAGGATTGGTAGGTGGCAATCACAAGAAGAGAAGGGGCCTCCCGTATCCGTTTTGCAATAAAGACAGGATTGGTGGTCATTTCTATATTGTCGTGATTTAGCACAATGCGGGTCTGGTCAGACCCCACAAGCAAAACGTTCTTCAAAGAAATTCCATACTGATAGAGTTTTTCCAGAGTCTGCCGTAGTAGGGACAGGCCCGGCACCAGGTACAAAACTTTTCCTTGAAGATAATTGGAGAGGATAAGATAGGCGACGCGAGTTTTGCCGCAGCGGCAGGCCATCTGCAGAATGGCCCTTCCACTTCGCCGCAGCTCCTGATAGCCCATATTGGCCGCCTCCTTCTGATAAAGCCGATCCTCGATTGCAGTCCGTGTCTCATCTGTAGAAAAAAATAATACGTCATCTGCGAAATGTTCTTCATCTTCCACAGGAGTTATCACCAGGTGTCTCAGTTTCTCCTTGCTTATCAGCGGATCAGAGGGCAAAGATGGCTCAACCACTATCGTGGAATCATTCATCTCATAAGTGGGAGAATCACACAAAGTATAGCTTATGTCCAGACAGTTTGCAACATCCTCAGCCAATTGTTTTATTTTTTCGGGTAAAAGACATACGAGTTCTTTGTTTTTGACGCGAAAAAACTGTGCGCAATACAACACCCCTGCTTCAATTTTTTGCGCATCCTTCTTTGTAGATGTTTCCAATGTGAAACAATACTTCCATTCATCCGTAAAACAGGTTGTATAAGATCCATCATGGAGCCTAGCGGCCAAGTTTCCTGTGTGCCCAACTTTATGTAAGGATTGGGCCTCCAGCCAGGGATGAACCGCCACGTAAAATCCTGCGCACATGCTATATCAAATTGCAGTTTCTTAATAACTGTACACAGGATCTGAAAAACATGTGATTACAAAATTTAGATAAGAAATATTTAATATTAAAAATCACGGAATACATGTCACTGTGTAGAGAGAAAGCCAAAAACTCCTCTTGACCGCCGTGGGAAATCATCCAGGGTAGTAGGTTGTGTTTCATGAAGTTGTATGCCGTAGTGATCACCGTGGACTCCAGATGGTTATTGGCGTCTTTGCAATACTTTGCCATCTTGGCAGAAAAGACGATAAATCCACAAATTCTACCCCAGTTGATAAGATCCTTAAACAGCTCAGTCACAACCCCAGTAAACTGGGTTTTAATTTCTTGGACACTCGTAAGAGAAAAGGTAATTGTAACCTGTTTGTTCAAACACTCATCATAATAGGTTAAAATTTTTTTTATTTGTTGTTGATATGGGCTAAGCTCATGCTCTGAAATATCATTAATGTAATATTTAATATAACCCACTAGTATTTCATTAATGATATTATGATATATTAACTCTTCTCCCTCCATAGCGGCACCCTATATTTTTTTATTTAGGTTTCAATGTTATCACAATTGCGATACAATTGTGATACAATTGTGATACAATTGTGACACGACTGTGTTGTATACAACAAATGTTAGGCCACGTATAGCAACCTATATGTTAAGAAATATTTTTATCCCAACATTGGTTGGAAACGAGCAGCCGCAAAGAAGTCATTAAAATAAGCCATTTAAAGATTTAGAATTTATATGTATACAACTGTACAATGGAAGCAGTTCTTACCAAACTCGACCAGGAGGAAAAAAAGGCTCTCCAAAATTTTCATCGTTGTGCTTGGGAAGAAACTAAAAATATTATAAACGATTTTCTTGAAATCCCTGAGGAACGATGCACCTATAAATTCAACTCATACACAAAAAAAATGGAGCTTTTATTTACCCCCGAATTCCACACCGCCTGGCATGAAGTTCCTGAGTGCAGAGAGTTCATATTAAACTTTTTGAGACTCATTTCGGGACATCGAGTGGTATTAAAAGGCCCTACATTTGTTTTTACAAAAGAGACCAAGAACCTGGGCATTCCTAGTACCATCAATGTTGACTTTCAGGCCAACATTGAAAATATGGATGATCTACAGAAGGGAAATCTCATCGGCAAGATGAATATCAAAGAAGGCTAAATAAAACAACTAACATCAAAAAACATTAAAGGCTATGTTGTGGACGATGCCTTTGTCTCAATAGTTTCGAGGTCATCCAATAACTCATGTAACGTAAAAAAGTTGGTCCATTTTTTTGAAAACATTAAAAGACGTTCGTCTTCATAAATAAAAAAGTCATTCGAAGGAAAAATGATATACTCAATACCATAGTCTTGTAATATTTTTTTTAGGTCTCTCAGGGTCCAGGGATTTACCAGGCTTCTACGCGAAGTGAGCATCATAAAAATATCTAATATTTTTTGCGCCATGAGCCAGCGCGGATTCTCATTGGCCCACAAATCAACAATAATTCTCTTATCAACCGTGAGCATTCCTACTTGATTCGAAGAAATGATTAGATGCCCGGCGGTCCACCCCATAAGTAGATAACGCAGCGTTGTAGAAATGTCACATATGGAAGGCATTCCTCCACAACATGAACCCAAATTAGGATGCGTGTGAAACACAAACATAGCAGGCTTGTTGGCCACCCTGCTATAAATATCAGCAGGCATCATAGCCTCGCTGCCAAAATAAATGTTCTCTCCTGCCCTATAGGGGCTTGGAATGATTTCCACTATCTCGGGTACACCGTTTATCATATTAATGCGGCCGCACCATTCACGGTCATCGTCCAAAAATTTTTTGATGGCACCCCGAACATTGTCCCAATTAAGCAACAGAGTGTTCACAATCTCATTACGCTCCGCCCAGTATTCCTTGAAACTTCTTTTAGACTTGCTGAGCTGTTCCCAGGATTCGAACTCGGTCCAATGTTTTTTTTCTTTTGGGGAAGACTTCCCTTTTGAAACATTTTTTGCGGCTCCACCATCTACACCATGATTTTCCAAAATAATCTCCTTCATCGTTTGAGTTATATGGGCATTGCTAAGCACCTTAGTGGTAACCTGTTTACCTATGTGATTTAGCAGAAAACCAAGTTTGTCCATTTGTGTCTCAACCATTTATTCTTAACAAAACAAAAAAAATTAAAAATCATCGTCGTTTAAAAAGAGTTTGAAGGCAAACGCATCATCCTTAACACAGTTCTGATACTGCGTAGGTCTTAACTCGAAAAAGTTGGTTTTTTCTACTTCATTAAGAAAGAATTTAGTCATCTGAGGAAAAGGGTTTCCCACTTTATAAATACTTTTGCACTGCATCATGAAGCACAAATTATCTGTAAAGTAGCGTATATATTGAAACAGCATTTCTTTTGAAAAACCGGGAACTCTTCCTCTTGCCTTGTCAAAGGCATAGTTAATGAACTCATCCACCAACTCCACAGCCTCCTTCAAAATTTTGTGAATGATCTTTTCCTCGGGAATGTTATACACGTAATTCGAGATAAGAAAACACGCAAAACTACAGTGCATCCCCTCATCGCGTGAAATAAACTCATTATAGCTTACAAGCCCCGGCATAATATTCTGTTCCTTAAGAAACTGGATCGCCACAAAGTGGTTTTGAAATAAAATGCCTTCTACGGCGGCGAAGCCCACCAGCCGCTCACCCAGAGTGTTCCTGTCGGGGTCCATCCACTGCCGCACCCACTGCGCCATTTTTTTTATGATAGGGTGTTTTTCAATGCCGCTAAAGATGCGCTGTTGTTCCTTCTCATCCGGGATCAGCGTTTTTACCTGTATTGAGTAGGCTTCACTATGAACACACTCTTGGGCAGCCTGCATTGTATAAAAGTATAACACTTCCTTTACTTTAATTTCGCGCATAAAATTGGTTAAAAGGTTTTCGATAACAATTTCGTCGGCAACAACAAAGAAGGCTAAAATTTGTTTATAAAATTCGCGCTGTGGCTTTGGCATGGCTTCCCAATCATCAATGTCCTTACACATGTCTACCTCCTGCGCCGTCCACGTCAAACTTTCTAATTTTTTATACCAGTTCCAACATTCGGGGTGCTGAATAGGAAAAATAGTGAAACGTTGGGAATTTTCAATTAGTAATTCCTCCATATTTGAAATAAATATTAACATCTTCAAATTTATTGGCTGCCATGGAGACGTTTTTTATTGAGACGTTGGCATCTGATGTGTATGGAAAGGCGTTGAATGTTGATTTAGATAGACTATCGCAGGCGCAGGTTAAATATACCCTTCAAGAGCTTATTTCCTACTGCAGCGCTCTAACCATTTTACATTATGACTATTCAACCCTTGCGGCGCGACTTTCGGTGTACCAGCTGCACCAGTCAACGGCCTCCTCCTTCTCAAAGGCGGTGAGACTGCAGGCCGCACAATCCTGCTCACGCCTGTCCCCCCAGTTTGTGGACGTCGTTTACAAGTACAAAGCCATTTTTGACAGCTACATTGACTATAACAGAGATTACAAGCTGTCCCTCCTGGGGATAGAAACCATGAAAAATTCCTATTTGTTAAAAAATAAAGATGGGGTCATCATGGAACGCCCGCAGGACGCCTATATGCGCGTCGCCATTATGATCTATGGAATGGGAAAAGTAGTTAATATAAAGATGATTCTGCTGACCTATGACCTGCTTTCCCGGCACGTCATCACACACGCGTCGCCCACCATGTTCAATGCAGGCACCAAAAAGCCGCAGCTTTCCAGCTGCTTTCTGCTAAATGTAAATGATAATTTAGAGAATTTATACGATATGGTCAAAACGGCCGGCATCATTTCAGGCGGCGGCGGTGGAATAGGGCTGTGCTTGTCAGGAATACGCGCAAAAAATAGCTTTATTTCTGGCAGTGGTCTTAGAAGCAACGGCATTCAAAACTATATTATGCTACAAAATGCCTCACAATGCTACGCGAACCAGGGAGGCCTACGCCCTGGGGCCTATGCGGTCTACTTAGAACTATGGCACCAAGACATCTTTACATTTCTGCAGATGCCCCGCTTGAAAGGACAAATGGCTGAACAGAGGCTTAATGCCCCCAATCTCAAGTACGGTCTATGGGTCCCCGACCTATTTATGGAAATACTTGAAGACCAAATACATAACAGAGGCGACGGCACCTGGTACCTCTTTTCACCGGATCAGGCCCCCAATCTACATAAGGTTTTTGATTTGGAACGGTCACAGCACGAAAACGCACATCGCGAATTTAAAAAGCTTTACTATCAGTATGTTGCTGAAAAAAGGTACACCGGTGTTACGACGGCCAAGGAAATCATTAAAGAGTGGTTCAAAACAGTTATCCAGGTGGGAAACCCCTATATCGGATTTAAAGATGCCATAAATCGTAAAAGTAATCTTTCACATGTAGGCACTATCACGAACTCCAATCTTTGTATTGAAGTCACAATCCCCTGCTGGGAGGGTGATAAGGCTGAACAAGGCGTTTGTAACCTGGCCGCGGTAAATCTAGCCGCCTTTATACGTGAAAATGGCTATGACTACCGTGGGCTCATAGAAGCATCAGGCAACGTCACAGAAAATTTAGATAATATTATAGATAATGGCTACTACCCCACAGAAGCCACGCGGAGAAGCAATATGCGTCACCGACCTATTGGCATCGGGGTCTTTGGCCTAGCCGACGTGTTTGCGTCTTTAAAAATGAAATTTGGTTCACCCGAGGCCATTGCCATGGATGAGGCCATCCATGCGGCCCTATACTACGGGGCCATGCGACGATCCATAGAACTTGCAAAAGAAAAAGGAAGTCATCCCAGCTTTCCGGGGTCTGCGGCCTCAAAGGGTCTACTGCAACCCGACCTATGGGTTCGCTGCGGTGATTTAAGTTCCTCCTGGGAAGAACGCGTGGCACAGACGACGCAGGGTGTGTTAACGCGGAAAAGCTGGTGGCAACTACGGCTGGCGGCCATGCAGGGAGTTCGAAATGGATATCTCACGGCCCTTATGCCCACCGCAACCTCCTCAAATTCTACAGGAAAAAACGAATGTTTTGAGCCCTTTACCTCCAATCTATATACACGTAGAACGTTAAGCGGGGAGTTTATTGTTTTAAATAAGTATTTAATAGACGATTTAAAAGAAATTGATCTTTGGACAGAAGCCATTCAACAGCAGCTACTAAATGCAGGAGGTAGCATTCAGCACATTTTGGATATACCGGCCGAGATCCGTGATCGGTATAAAACCTCTAGGGAAATGAATCAAAAAATTTTAACAAAACACGCGGCCGCACGAAACCCCTTTGTGTCCCAAAGTATGTCTTTGAACTACTACTTTTATGAACCTGAACTAAGCCAGGTACTTACGGTGCTCGTCCTAGGCTGGAAAAAAGGTCTAACCACCGGTTCCTATTACTGTCATTTTAGCCCTGGAGCGGGTACCCAAAAAAAGATTATAAGAAACTCTGAGAAAGCGTGTAATGCGGACTGCGAGGCGTGTCTTCTGTAGGAGTCTCGCGGTAAAAGAGCAGCGGGGACCATATGGCAAACCCCAATAAGAGGATAATGAATAAAAAAAGTAAACAGGCATCCATTAGTTCCATATTAAATTTTTTTTTCTTCTATATAATGGAATATTTTGTTGCGGTAGACAATGAAACCCCCTTGGGGGTTTTTACTTCTATAGAGCAATGTGAAGAAACGATGAAACAATACCCCGGCCTCCATTATGTCGTTTTTAAGTATACTTGTCCGGCGGATGCAGAAAATACAGATGTTGTATATTTAATACCCTCGTTAACCTTGCACACCCCCATGTTTGTAGACCACTGCCCAAATCGTACCAAACAAGCACGACACGTATTGAAAAAAATAAACTTAGTGTTCGAGGAAGAGTCTATTGAAAATTGGAAGGTTTCAGTAAATACTGTATTCCCCCATGTTCACAACAGATTATCTGCGCCGAAATTTTCCATCGATGAGGCCAATGAAGCCGTAGAAAAGTTTTTGATACAAGCAGGGCGACTCATGTCTCTGTAAATGTCTCCTTTATGGGCGATGTCTCTGTAAGTGTCTCCTCCTTTACTGAGGAAGTCTCTGTTATGGGCAAGAGGTTTGAAACAACGCAGGGGCTCTGCTTAATCTGCTGTCTCACAAAGGGAATCAAACTACCTGCTTTCGTATTTTTAATGTAGTAATTACCCTTGTTATGGTGAATTTTAAGACCATAGCGTAGACCCAGCACTTTATTAATGAATTTTAAAATTGTTTGGGGATCCGTTTTACTAGGCTTTTTAAGCTTAAACTCAAAGCTGACCGCGCTTAAATCATACTGAACAAATTCATCAACGAGTTTTGTCATCAATTGTTCATTGGTCAATATATTAGGGTCCTGAACGCATTTAAAGCCGCACTTAGTTAACAGCATAATGGCGTACATATGGGATTGAAAGCTATAATTAAATTGCAGATCATGATGCTCTGCATGTTGCATGGCCCATTGATGGAAGTTTAATTCCTGAGTTTGTAACATAGTGAGCGATTCGTATACTGTTTTTCCGCGGCTTATTTGGACACGGCCAGTGTAGTTCTGTTTTGTCATAAAACTATTGTATTGTTCAACAAATTTGGGGGTAATTTTATGACCGTGCCATGCATAAAATTCGAGTAGTTTATATTTTTCATACGCAAATAGGTCTTGCTGGTCTACTGTGATGCCTTCCTTTAAGTTTTGTTTAATTTGTAAAGCTTTATTGGCATCAATGGTTTCAGCCGAGGCAATGTTTACATAGTCCTGGTGCTTAATTTCCATTTTAATGCTTGTATACTGTTTGACTGTCTCCAGCTTTTCACCCGTCAGTATAAACACCTTAGCGCCGGTGTCGGCGACCTGGTTAATAAATCGTGTTATAAAGTGATTTTTTGATAGATGTTGTATCCGCATTGTTTCGAGCCATAGATGGTAGTATGGAGTTTTATAATATACCGGCCTACCTGTTTCCTTACTATACGTGAAGGAAAGCTGGTGATTGTTTATGGTCTGAAAGAGGGTGTCACGTTTTTGTAACGTGAACATTTCAATGTCTTCGATGGTTTCTGGGTAGTAATTTTGTTTCCCCTGTAAGCAGATTTTATAACACTTACTTTTTAATTCACGCACGCGGCCCAACATTTGGCAACATGTTTCTACGTCACACGACATATTGTTAAAAAAGCCGTATAAAACATCAAATCTCTTATCTTCGTACGAAACACCCGCCGAAATCGTGGGCGTATAGATAAGGATATCAACGAGCCCCCAATAATACGATACATTATTAAAATGGGATTCCCGTTCATGAGCAGTGCTTTTAGAACTATAAAACCCGATTTTTTTTTCCGGAAACTTTTTTTGGATAAATGATTGCAACAGCCGGGCCTCCATTAATGAATTTGTAGGGATAACAATTTTTTTGTCTTCTAGCAAATCCTTTAAAAGGTTATTTAACCAAGTTTCTCGTGAAGAGGTGAAATAATACGTGTCATGCTGGGCCCTTTTATATTGATTCCAGTGAAAGAAGATAGGAACATCCCCGCGAAAACGCTGTAGAATATTATACGTTCGATTTCCTAGGTTTGCGTCCAAGCATATAACATAATTTGCCGTTTCGAGCATCCACATGAAAATGGCAAAAGAGGGAGCAAAGTATTTGTGCAGGCCGCTATTGAATTGATTAAAAATCGATTCTACTTCATCCAAAATAAGTAGGTCCACAGGCTCGGCTGTGGAGGTTAGCCGGAAAAGTGATTCCACCTGAACGATGACTCTTTCGTAGCTGTCCAAATCTCCAGTTACTTCACTGTATAATGTGAAATTTGGTAGCCGGGACTGTATATTTTTTGAGAAGATCTGTCGAAATGTCACAAACCGTATGGTTTGTTGTTTTGAAATAGAATTATTGCCGTAGTATTTTTGCAAATAGTTGCGCAGTTGGACGGTTTTACCTATTTTCATTTGAGCCTTTACAACAAGCGTAGGGACTCGTTCATATTCTCGCATACTACTTTCATCATAGATGTGTTTTTGAGTATCAGGCAGTTCTTCAAAGAGAATGGACTCATGGACCTCTATGCTCTTTGTCATCACTTGGTCCACATATGTTTCCACAAAATTAGTTGTACCGGAAAGGCTGCCCATGAGAAGGCTATGTTTATTGTCATGGCGACAGTGTTGATACACTTTGTTTCCCGTGACTCTTAAAATTAGGGTATTATCCTTATCATGCATACGCTTACATATTTCGCAGTAACTTGGACTTGTACGTTTAAACAATACTAAATTTTTATGAACACGGAGGAAGCAATGATTTTTACATAGTGTTCCTGCAAATTTTAATACCTCTTCAAGTTCACTTTGTTGGATAGTATCGCAGGAACTCGGTGTTGTTTCTTTTACATTTGTGAAGATACAAGGTAAACACGTCGTTTCAAAGGGGGTTGCTATGAGGGTATCACTCTTTTTCGTGGTTGTACTGGTCTCAAACACCTCTGCAAGCTCTTCATTAAACATTTTAACACGCATGCTACCTTTTTTATGAGACCCTATGATGCGAAAATTTTGAATGCTTTTGTTGACCTGAGGGTCAACAAAAGGATAAACGTGTTTGGGAAGATTTTCTAACACTTTGGATGTAAAGGCTTTGGCCTCATTATTGTTTAATACTGAGTATGTATAAAGTATGATATGAAAGGAGTATTTAAGTTCTCGCTTTTTATTTAACCCGATAGAATCTGTTAGCAAAATTTGTTCACGCGTTAGATTGATGTTATAAGGTAAAGAATATGTCTCGTAAAATACATCTATGATAACGTTAATTATCATGTCAAGGATGTCATAGACATTGTCATCGTCATAGACACTGTCATCGTCATTGACATTGTCATTATCATCAGAGTATGACTTATTTACCGGGAAATCGATGTCAAATTTTAAGCGCTGAGGCAAAAATCCAAATACCACTTCGTGGAAACACTTCTGCTCAAAGGGCTGAGCCGCCTCCCACTCCCAAAAGTCATCACGATTTGAAAAAACCCTAAAAAGATTATTATATTCATCTCGCACCACGAAGTGATTCTTTAAGGTTTCGAGAGAATATTTATCCTCTACGGCTTCTCCTTGGGAGTTACAGCGAAGAAACTTGAATGTTTCTTGCATTTTGATAATTAAAATTAAATCAATTATGATGCGTCAGTGGCCGCTGACGCAGCCGTCATAAATAAAGCGGCGGCCGTATTATAACGACTAGTTGGCCGCTATAGGACGAGCCATATAAAAATGAATTCTTTTAATTAGAGTTAAGTATTGTTGATTGTATAATTCATTATGGTTGAGCCACGCGAACAGTTTTTTCAAGACCTGCTTTCAGCAGTGGATCAACAAATGGACACTGTAAAAAATGACATAAAAGACATCATGAAAGAAAAAACATCTTTTATGGTGTCATTCGAAAACTTTATAGAACGTTACGATACCATGGAAAAAAATATTCAAGACCTTCAGAATAAGTACGAAGAAATGGCGGCCAACCTTATGACCGTCATGACGGATACAAAAATTCAGCTTGGAGCCATTATCGCCCAACTTGAGATTCTGATGATAAATGGCACTCCACTTCCGGCAAAAAAAACAACGATTAAGGAGGCTATGCCCCTACCTTCATCAAACACGAACAATGATCAAACGAGTCCTCCCGCCTCAGGCAAAACAAGTGAAACACCTAAAAAAAATCCCACGAATGCAATGTTCTTCACGCGTAGCGAATGGGCATCCTCGAAAACTTTTCGAGAAAAGTTTTTAACACCAGAAATTCAGGCCATATTGGATGAGCAGTTTGCAAACAAGACCGGGATCGAAAGATTGCATGCCGAGGGTCTTTACATGTGGAGAACCCAATTCTCTGACGAACAGAAGAAAATGGTCAAAGAGATGATGAAGAAGTAATATTTTTGGTAAAAATATTTTTATCAAAATTTTTTTACCAAATAATAAAAAATATTTTTTACTTTTTTTTCTTCATAATATACATAGAATGCCTACAAAAGCTGGCACAAAAAGTACCGCAAATAAAAAAACAACGAAGGGCTCCTCCAAATCTGGTTCTTCCAGAGGCCACACCGGCAAAACCCATGCTTCTTCGTCCATGCATTCCGGGATGCTCTATAAAGATATGGTAAATATTGCTAGATCTAGAGGCATTCCGATTTACCAGAATGGATCGCGTCTTACTAAAAGTGAATTGGAGAAAAAAATTAAACGGTCAAAATGAATATAATCAGGAAACTTAAGCCTGGAACAATTAGCCTTGTGCTGGGACCCATGTTTGCCGGCAAAACTACGTTTCTTATTCATTGCATTTACATGCTCGAACGTTTGGAAAAAAAAGTAGTCTTCATAAAATCTACCAAAAACACCCGAGACAAAACTATTAAAACACACTCCGGTATACAGCTACGACCCAAACAATGTAAAATCATAGAAAGCACACAGTTATCTGACGTGGGTTCTCTCACCGATATCCATGCAGTTGTCGTAGATGAAGCGCATTTTTTTGACGATTTAATCACATGCCGCACTTGGGCAGAGGAAGAAAAAATTATTATTCTTGCGGGACTCAATGCTTCCTTCGAGCAGAAAATGTTTCCGCCCATCGTTCGTATTTTTCCTTACTGCAGCTGGGTTAAGTATATTGGCCGCACCTGTATGAAATGTAACCAACATAATGCATGCTTTAATGTGCGTAAGAACGCAGACAAGACGCTTATCCTTGCGGGAGGAAGTGAACTGTACGTAACATGTTGTAACAACTGTCTAAAAAATACATTTATTAAGCAGTTGCAACCTATTAAATATTAAAAATCTTATACAATAATGGATCATTATCTTAAAAAATTACAAGATATTTATACGAAGCTCGAGGGTCATCCCTTTCTTTTTAGCCCGTCGAAAACCAATGAAAAAGAGTTTATTACTCTGCTAAACCAGGCCTTGGCCTCAACGCAGCTTTACCGCAGCATACAACAGCTGTTTTTAACGATGTATAAGCTAGATCCCATTGGGTTTATTAACTATATTAAAACGAGTAAACAAGAGTATTTATGCCTGTTAATTAATCCTAAACTCGTTACTAAGTTTTTAAAAATAACGAGCTTTAAAATTTACATTAATTTCAGGCTGAAAACTTTTTATATAAGTCCTAATAAGTATAATAATTTTTACACCGCTCCCTCTGAAGAAAAGACTAACCATCTTCTAAAAGAAGAAAAAACTTGGGCAAAGATTGTTGAAGAAGGAGGAGAAGAATCCTAAGTCGCTTACATTTTTTTTTGCTATTTTTATAGAATGTACACGCATGTTGATGTTGTCGGAATAGCTGAAGCCTCAGCGGCCCTCTACGTGCAAAAAGATAGGGATCGCTACTTAGACGTGCTAACAACCATTGAAAACTTTATTTACCAACACAAATGCATCATAACAGGGGAAAGCGCCCACCTACTCTTTTTAAAAAAAAATATTTATCTTTACGAATTTTACTCCAACAATGTGGCGGAGCACAGCAAGGCTTTGGCGACCCTGCTTTATAAACTTGATCCGGAATACCTCACTCGTTACACAGTACTCATTACCAAAATTCCCAACCATTGGTATGTGATTAACGTAGATCAGCGAGAATTTGTGCGCCTATATGCCATCCCGGCAGTTAAACAACACTTACCGATTCCCATTTTACCCTTCTATTGCACCAGCGCACTCACCCAGCAAGAATTGTTTTGTTTAGGACCTGAACTGCAGTTAATACAAATATATTCCAAGCTCTGTAACCCCAACTTTGTCGAGGAATGGCCTACGTTGCTCGACTACGAAAAAAGCATGCGGATGTTATTTTTAGAACAGTTTCCGCAAAGATTGGAAATGACGGGCGGGAAGAAGGAGGAGAAGGAAAAGCATGAAAGTATCATTAAAAAAATAATACTAGAAATGGTCTCTACCCGTCAGCGAATCGTTGTTGGGGGTTACATACAAAAAAACCTGTACAACCATGTACTCAAGAATAGAAATCGTTTACAGCTTATTACGAGCTTAAATATTTATGAAGAAAAAGATATCATCCAGCAATTTTGTGATTCAAATGGACTGAAGATCAAAATACGTATCAACAATCCGCTCTTGCCTACAAATCCGGAATTACGGCGTTTGACTATTTATTTTAATCATAATAATGATGATGATCAGTCATATCTAATAGTAGATATGTACAACACGGGAAGCTATGAGCTAGTGCCTACAAATCAGATAAACACGCTTGATGGCAGCTTTTTAATAGGAACACCCTTCGTGCAAGCGCGATTTTTGTTGGTAGAGATCTGGGTGCTTATGCTTATTGCGCAGCAAACTAAAAAGGACACCAAAAAAATAATACAATTTTTTATAAATCAATATGAAATGCTTATGAATAGTCCTTGGCCCAGTATGGAGGCCCTTTTTCCCTCAAGCAGTAAAAGATATTTAGGCAACTATGTAGACCCTAACGCGCTCATAAAGTGGGCACAACTCAAATTAAAAAGAATACCGCCTTTTTATCCTGGAAAGCCGGATGAAGAATCATGTTAAGCCGATTAAAAAATCATGTTAAGCTGGTTGAAAAATCATGTTAAGCTGGTTGAAAAACTCTTGGTGAAAGCACGGATGTAATATTAACATTGGCCGCTCGCATTTCGTGTTGAAATACGATGGAAGAGCGACGGCTATCTACCATGCCGATATCGGCCTGGACATCACAGTTCATGCACTTGTAGATGGGATGACTCGCGTTATAGATGGCAGGCTCGCCACAGTTTCTACAGATGTAGGAGATGCAGCCATCCGAGTCGTCGTGCGATTTTTCTATGATGGTTTGCATGGCGCCCTGCGCCGTAAGCACCCAATGCTCCATTTCTCCCAGACGAAGACCTCCGTGCGATCGTTTGCCGTCCAACGGCTGGCCTGTGAGGGCATCCGTGGGCCCATAGCTTGCAACGGCGTATCGGTCATCCAGCACAAATTTTTGCAGGCGCTGGTGATAGGTCGGTCCTATGAAGATGGCCGCATCAAAGTACTCGCCGGTCTGGCCGTTGAACATTTTTTGGCATCCATTGAAGCGTAGACCTTCTTGCGCCAGTCTTTCTGAAAGAAGCTGCACATTAATAGGCAGGAATGCGGTGCCGTCTGTTACCACCCCCTGTAGGGCATTTGCTAGACCAACCGTGGTTTCTATCATTTGACCGTTGGTCATTCGGGAGGGATGTGAGTGGGGGTTTACAATGAGGTCGGGCTGCAATCCGTCCTCTGTGAAGGGCATGTCTGAAGTGGGCAGGGCCAGCGCCGCAATGCCCTTGTTCCCGCTGCGAGAACTCATTTTGTCGCCTATATTGAGATTTCTTTCATAGCGCAGGCGCATGAGGCCAAAGATCTCGTCATTAGGCCCATGGGGACGCATCACAGCATCCACGACGGCCGGCTCATCGAAGCCGTACATGACAGACCGGTCGATGTATTTGTTGAGTTCGTCTTTTTCGCCCCGTATTTTGGCCACTTTTCCTATAATGATGTCGCCCTTTTTGACCACCGTTCCTACGGGCACGAATCCATCTACAAGCTTTTCGTAATTAGCACCAGGCTTAAGATTTTTGGTGATTAAAGGGTCGGGCTTCCCAAACGACTCTATATCGCTTTCTAATTCTACTTTTTCTTCTCGGTAGAAGGTGCCGGCAAAGCCGCCCCTGTCAATAAAGGACTGCGACACGATCACAGAGTCCTCCTGATTGTAGCCGCCGTAGATCATATAAGCCACAATGGTATTAAGCCCGTTGGGTATGACATAGTTATGTGCTATGGTCTTTACAAGCGGCATTTCATTGTAAAACTGGAAGAAGCGGTTCATGTCGACACGATATGGCCAGCTAAAGCAATACCAGCCCCCCGTTTGCCGGCCTTGGTTTGTTTCATAGGTAACACGCGCAGGTTGGGTACAGTTTGCGTAGGGGGACACTAGGGCGGCAAGGCCCAAAATAGCTTGGGGCACGTCCACGTGTGTGAAACGACGCGTTACATCATGTTTATGTTTGCGTAGCTCGATGATGGAGAAGGCAACAAGACAGTTTTCCGCTTCTTCGGGGGTAATGAATTCACAGATGCCCTGCGCTACGAGATCTTCAAGTGTAAGTGTTCCGGCTAAAATGTCTTTTGCCATTTGAGACGTAAATCGCGTATTTTGAATGAAAGGGATTTTATGTTTTTCCCAGTCTTTATCGCCTTTTTTTCTGGCCTCTGCGGCCTTGTAACAGGCTTGATTGTATTTTTCAATATTATTATCTACAATGAGCAGGGGGCGGGTCAGCCTACCGACGTCCAACCAGAATTCTACTTCGTCTACCATGCTATCCCAGTAGATGGTGGTATGGGGATGCACAATCTTGCCCTCACGGCGAAACATTCTATACCGCTGAGCAAGCTCAAAGGCGTTGGTGCAGCAGCCAATCCATTCTCCGTTGATAAATACGCGCGCTAGGCCCTTTCGTACAATGTCCTTGTTGGAAACATCGGCTAACTGTTGAATGGCCGGATCTGATAGAAGGCGTTGTTTTAACGAAAGTACTTCTCCAGCGGTACAGACATTGGCGGTAATGGCTAACTGTTTAGACATACCTACTTTTTCACCAGTATCGGCTGACTGGGCTACGCAGATGTATCCAGGATAAGATGCGTGCACGCGACGCATCATGTCAGCCCTTTCTGTTTGTTTGGATGCGTTGGTGGTGTTATGAGTATTTACCGTACGCAACGCTGAAATCGTATTTAATAAATTTTTTCTTTCCAAACTTTGAGTAGATACTCTGTTTACAATGGGGCGCTGTCGCACCATGATGGTTTTATTTCCTGAAATGATAGACTGTTCCATACTGCGATTGAGATCGGAGGCGGTATTTTTTGATAAAGCGGCAGAAAATGCCTCGATAATGTTTCGCTGGGTTAGCTCCTCAAAAGCTGTTTGTTTAAGAAGTTCTTTGAACCCATTGATGATGGGTGCTATCACGGATGTATTAAAAATAGCCTTAAAGGCCTTAGCAAGCGAGACCCCTGAGCCGTGCACCCGCTTGGTGCGGTAGCTATCACGGTCCGTGGGTGGAAACACATTCATAATGACAAGAAGTATTTTATGAATAAGCAGGCCTAAAAAGCGCAGTTTTCGTACACGTGTATCTGCGGTTTGGCCCATGTGTGGCAACAATATTTTGTCTAAAATAGTAAGTTGTCTTTCATTCAAGTATTGTACCGCATTTTCATCGCTTTTGTAAGCAGATGGGTTTGAGACAAATTTGGAAACCTTCTCAGATAAAAACTGGATAATTTTTTCTCGGTTCAGCTCGTGTTGGACCGGTTGAAATATGGGGTCTAAAACATGAATGGATTTTTCCAGAATTTCTATCATGAAGGTATTCACAGGGGAGTTGGATTCCAGATCGAATACCACTTGCTCAATGATGCTGTCATCGCCTGTCATTCCAAACATGCGAAAGATGAGATACCAAGGTATGCGAAGTTTTGAGAACTTGGTGCTATTGATTTCAATGGTAATGGCACCAGTAGTCATGTAGCGTATAATAATTTGAGAGCTATTTTCGAAGGCACCTCCTGGTTGAGAGATAAACTCGCCGCGAATGATTTCATTATTCCCTTGTTGCATGGTATGGTAATGGATGTGAAGCGTGTTAAAGCGGATGTTTTCTAAGAGGTCTACGACCCATTCCCCGCCTCGGGCTATAAAGTAGCCGCCGGGTTCATTAGGGTCTTCTCCTATTTCTTTTTTTGCGGTTTTTGATAGGTGATGAGTGTGGCAGCGGTTGCTGCCCCGCATGATGGGAAATGTGGACACCTGAAATGGAGGAATACTTGCTCGTTTTACCTCCTGCCGACCATTGCTGTAGTGCGCCGTTAAAATAACCTCGGCGGCTAGATTAACAGGGCCCGAATAGGAAAGGCCACACAGGCGTGCCTTATTAGGTAGTAAATTTATCTTGTTTCCCTGTGAATAGTTTCGATGTTGCGGGCGTTCAATGTTCACATCTGTAAAGTTAAATTGGATCTGAACCGATTCCCGAAGCTTATCTATTTCAGTATGGTCGCGTTGGTCTTTATAAGTAATATCCACGTTAAACATTTGTTTTACAATTTGCGGGATTCCATTGTCCATAAGATCGTCGAAGCTTTTGATGTTATACCCTATCAATCCTGTAGAGTTTACTGCAGCGGAGATAAAGCTCAGCATATCAGCCTCTGTAAGCTCCTCATTATCCACGGTTTCAATGGGGCCGTAGGTTATTTGCGGCCGCAAGGGTTCCATAATTATGAAGTACTACATTAATATTCAGTTATTCTTTAAAATAAATCTTTATTTATAAATCTTATTTATAATATAAGAATGCCTTATTCAAGAGATATCACAAAGTTTATCACGGCAACGGAACCAGAGGTGGGTCTCCCCCTGTTGGCGCTGCAACACTCCAAGTCCGTCATAGGGGTTATTCTTCTTGTAATAAGTTTGTTATTTATTTTCATTGGCATTATTATACTATCGGTGAGTAGTAGTCATACCACAGCAGCCTCTATATTTATCGTATTGAGTCTTATCCTAGGTGGCGGTGGTTTTTTTCTTATTTATAAAGATAATTCTTAACCCACATAAAATTTGAAAAAATATAAAGTAAGAAAATGTCCAATTACTATTATTACTATGGCGGGGGGAGATATGATTGGTTAAAAACAGTAGAACCCACTAATTTTTTAAAAATCGGGTTGCCTTACCAGGCACACCCATTACACCTCCAACATCAGGCAACTACTACTCCCCCATCTATCCTAGAAAAATTTAAACGAGCAGATATTCTTCTTAATGAGGTGAAGGCCGAAATGGACCCACTCATGTTACAACCAGAAACCGAAAAAAAATTATACCAGATATTGGGTAGTATTGATATGTTCAAAGGTCTGCGAAAAAAAGTAGAATTTACGTACAATGCTCAAATTGTTACGAATGCTTGGCTTAAAATGTATGAGCTGCTAAATACCATGAATTTTAATAATACATCTCAGGCATTTTGCAATTGTGAGCTTCCAGGAGGGTTTATAAGTGCAATTAATCATTTTAATTATACAATGATGCATTACCCTACTTTTAACTGGGTAGCCTCCTCCCTTTACCCCAGTTCGGAAACAGATGCCCTGGAAGACCACTATGGTCTTTATCAGTGCAATCCGGATAACTGGCTGATGCAATCTCCTTTACTAAAAAAAAATATAGATTATAATAACGGGGACGTAACCATCGCTAGCAATGTAAAAAACCTAGCGCTTAGAGCCACACAAAGGCTGACGCCCATCCATCTATATACGGCTGATGGGGGTATTAATGTAGGACATGACTACAATAAACAGGAAGAATTAAATCTTAAGCTTCACTTTGGTCAAGCCCTTACGGGTTTGTTGAGTCTTAGCAAAGGCGGAAACATGATACTCAAACACTATACCTTAAATCATGCATTTACTCTTTCTTTAATATGTGTATTTTCTCACTTTTTTGAGGAACTATACATTACCAAACCTACCTCCTCTCGGCCCACAAACTCTGAAACCTATATTGTGGGTAAAAACAGATTACGCTTATTTACCCCCAAGGAAGAACAAGTCCTTCTAAAACGGCTAGAATTTTTTAATGATACGCCCCTCGTAGACCTAAGTCTTTACCAAAATTTACTTGAAAGCGTTTACTTTGCCGTAGAAACAATACATCTAAAACAACAAATAGAATTTCTAAACTTCGGAATGAAATGTTATCGACATTTTTATAACAAGATTAAACTACTTAACGATTATTTAGCTCCGAAAAAAAAGATTTTTCAGGATAGGTGGCGTGTGCTTAATAAGCTTTATGTTCTTGAAAAAAAGCATAAACTTAAGCTTTGTGCCTCCTAGGGATCTGTTGCTTAATTTAACAGATGCAATCTTAACAGATGTAAACTAAAAAGTGTGTTCATACAAGGATTGTATTTATGAATATTTATTAACATATAAGGTTGTGATGTAACACTGTATAACCTATATAACTACACTATGAAGCACGGCGTATAATAATTTATATTGAACACGATGTTGACTCATTTATTTGCAAACAAATATTTGTTTGCAAGACGTTTGCATGCATTTACTAATATGTTGTTGACTAGTTTATTTGCAAACTAGATGTTTGATTGCAAACTAGATGTTTGCACGTATTTATTTGAACTAATATACACTCCTTGTTTTATTTGTTATATACACAGCATACATAAGTGTATATTGTTTACACTTATGTTTATAACTCGACGTAATAACATTTTACACGCTTTTTTTTTGCAAATCTTAATAATATTGTATGATAAATCAAACAATGTCTTATATATGTGGTTTATTATTTTAGGCGCCGCAAGATGTACTCCATTCTCATTGCATGCTTGGTGTTATTACTCTGTCTAGTTATATATGTCGGTCATCGTGCCGATCATGCACGAAAATATTTAGAAGGAATGTGGCATGGAGATCCGGTTTTTCTAAAACAGTCGGGGCTACAATCCTTTTATCTCTACATACAACCTGACCATACATGTTTTTTTAGCATTGTGAATAAAAATGGTGAAAAGCTGATGGAAACCAAAATACCTTGTACGATAACAAATAAAATATATATGTTTTTTAAACCTATTTTTGAATTTCATGTTGTGATGGAAGACATACATAGCTACTTCCCTAAGCAGTTTAACTTTCTGTTAGATAGTACAGAAGGTAAACTTATTTTAGAAAACAATCACGTTATTTATGCTGTATTGTATAAGGATAATTTCGCCACCGCACTAGGAAAAACGGTTGAAAAATATATAACACAAAATTAATCATGTTTTCTAACAAAAAGTACATCGGTCTTATCAATAAGAAGGAGGGTTTGAAAAAAAAAATAGATGATTATAGTATATTAATAATTGGAATATTAATTGGAACTAACATCTTAAGCCTTATTATAAATATAATAGGAGAGATTAATAAACCAATATGTTACCAAAATGATGATAAGATATTTTATTGCCCTAAAGATTGGGTTGGATATAATAATGTTTGTTATTATTTTGGCAATGAAGAAAAAAATTATAATAATGCAAGTAATTATTGTAAGCAATTAAATAGTACGCTTACTAATAATAATACTATTTTAGTAAATCTTACTAAAACATTAAATCTTACTAAAACATATAATCACGAATCTAATTATTGGGTTAATTATTCTTTAATTAAAAATGAGTCAGTACTATTACGTGATAGTGGATATTACAAAAAACAAAAACATGTAAGTTTATTATATATTTGTAGTAAATAATATTTTTAATTACTTAAAATTTTTATATATAAGTTTTTGATACTATATTATAAAACATATGTTCATAAAATGATAATACTTATTTTTTTAATATTTTCTAACATAGTTTTAAGTATTGATTATTGGGTTAGTTTTAATAAAACAATAATTTTAGATAGTAATATTACTAATGATAATAATGATATAAATGGAGTATCATGGAATTTTTTTAATAATTCTTTTAATACACTAGCTACATGTGGAAAAGCAGGTAACTTTTGTGAATGTTCTAATTATAGTACATCAATATATAATATAACAAATAATTGTAGCTTAACTATTTTTCCTCATAATGATGTATTTGATACAACATATCAAGTAGTATGGAATCAAATAATTAATTATACAATAAAATTATTAACACCTGCTACTCCCCCAAATATCACATATAATTGTACTAATTTTTTAATAACATGTAAAAAAAATAATGGAACAAACACTAATATATATTTAAATATAAATGATACTTTTGTTAAATATACTAATGAAAGTATACTTGAATATAACTGGAATAATAGTAACATTAACAATTTTACAGCTACATGTATAATTAATAATACAATTAGTACATCTAATGAAACAACACTTATAAATTGTACTTATTTAACATTGTCATCTAACTATTTTTATACTTTTTTTAAATTATATTATATTCCATTAAGCATCATAATTGGGATAACAATAAGTATTCTTCTTATATCCATCATAACTTTTTTATCTTTACGAAAAAGAAAAAAACATGTTGAAGAAATAGAAAGTCCACCACCTGAATCTAATGAAGAAGAACAATGTCAGCATGATGACACCACTTCCATACATGAACCATCTCCCAGAGAACCATTACTTCCTAAGCCTTACAGTCGTTATCAGTATAATACACCTATTTACTACATGCGTCCCTCAACACAACCACTCAACCCATTTCCCTTACCTAAACCGTGTCCTCCACCCAAACCATGTCCGCCACCCAAACCATGTCCTCCACCTAAACCATGTCCTTCAGCTGAATCCTATTCTCCACCCAAACCACTACCTAGTATCCCGCTACTACCCAATATCCCGCCATTATCTACCCAAAATATTTCGCTTATTCACGTAGATAGAATTATTTAATATGTACTATATATTAATTATTTAACCTTTCAAGCTGGTCTTCATTTAAATTTAAAATCCACTAATAAAATGTATTTTCTAGTAGCAGATCATCGAGAACATCATGTGATTCCTTTTCTTAAAACCGATTTCCATCACATGCATCAAAATCCTATACAAAAAAATCAAGCTCTCCTAGAAATCAAACAGCTTTTTACTGGAGATTATCTCATCTGCAAAAGCCCTTCTACCATTCTGGCCTGTATTGAACGAAAAACCTACAAAGACTTTGCGGCTTCTTTGAAAGATGGACGTTATAAAAATCGCCAAAAAATGCTGTCGCTGCGAGAACAAACCAACTGTCAACTTTATTTTTTTGTAGAAGGCCCGGCATTTCCTAACCCTCAAAAAAAAATTAATCACGTTGCCTATGCAAGCATTATTACTGCTATGACGCATCTTATGGTTAGAGATCATATTTTTGTCATTCAAACGAAAAATGAGGCCCACAGTTCCCAAAAGCTTGTGCAGCTTTTTTATGCCTTTTCTAAGGAAATGGTGTGCGTCGTTCCCACCTCCCTCACCCCCACGGATGAAGAGCTATGCATCAAGCTATGGTCTTCTCTTTCTGGTATTTCAGGCGTGATAGGTAAAATCTTGGCAAACACTTGTTCCGTAGCTCATTTGGTTCATGGAAAGCTTTCATCGCAGAATATTGATCAGTTAAAAACTCCCTCCAACCGACCATTCCCCAAAAAAGTAAAACGTATGCTTATAAGCATTAGCAAAGGAAATAAGGAGTTAGAAATAAAATTGCTCTCGGGGGTTCCCAATATCGGGAAAAAATTAGCTGCCGAAATTTTAAAAGATCATGCGCTTCTTTTTTTTCTAAATCAGCCCGTAGAATGCTTGGCAAATATACAAATCGTTCAAAAAACCCGTACGATTAAGTTGGGAATGAAGCGAGCCGAAGCGATTCATTATTTTTTAAACTGGTGTGGCTCTGCCCATGTAACCGATGATAGCCAAAATATCACAGAGGCGTCGCGGTCCACAATGCAGGTCGCGACGCAGTCCGCCGCAATACAGCCCGCTGCAACGCAGCCATTGCACGAAGTATCAGATGATGCATCATCAGATGCTTCATCACCCGTAGGGTATCAAACATTATCTAAAGAAATGTTATTGAACACAGCCTGATGTTAATAATTCACTACATCTAAAGAAATGTTAACCTCGATACTAAAAAGTCATTGAACACAACTACTGGGGCGCTAAGTTGTCCAACACATCTAAAGAAATGTCAACATCCTCGATGCTAAAAGGGTCATCGAGCCGGTCAATAATGTCTTCCCCAAAAAGTCCGGGAGAACTGTAGGCCGAGATGTCGTCCATGGAGCTATCTTCCCCAGAGCACACAAAGTCCTCTCCAAAAATCATAAAGTTAAATGCACCGGGCTTACTTAACAGCTTTTCGCTTTGAATAATAGTGTTGAGTTCTGTCAGCGCAAACTCTCTCACAATATTCACAACCCAGGAGGGCTCTTTAATTTCATACAGCGTTAAGAAACTTATACATAAAAATTCTATAGAGTAAAGCAAGGCGCTGGCAGGATCTGTTACCCGTAGGTGTTTAAATGTAGTGTGATATTCATTCACAACGTTAGGCAGCACCTTTTCCAAATCCTCCTTTTCCTCGTACGACAGGTGCTTTACAAGCCTTTCAACATGTATAGGAGGCTTGTTAAATGTACTAACGTGCCGCAAACAGTTATAATTATATAAGAAAATACGTACGGCAGAGTCGACCGCCATGAGCCTTGGATCATCCATTGAGGTAGGTGGTGGCGGGGCACCCTGGCCTTCCCTGATGTCTGCGTAGGAGCGCCCCTCCATGGCCCCTATGGCCTCTATCACAGCAGGACTGATATCCAAAATCTTGGCCGTCTTGATTATTTTTCCGTAATCGAAAGTCCATGGCTCCTGTGGAGGCTTGGGTTGTGTTTCGGTGGAGGGCGTGGTCATATCTTTCTTTATTTGAATAGAACGGATCGACATCTTTTCCTTATCGTACTGGTCTTTATAATTATTATAATAGTCATGAACTAATTCGGGTTGAGAAAGATGATCGTATATAATATAGGTAAAAAGTCCGCACTTGACACATTTTTTATCCTGGAAGTCGTGTAATCCTCCCTTGGGGCAGCGTGACTCGTAGAAGGCATAAAAGGTGTTAAATTCTAAGCTCGCCTTTAGGGCTGTTTGGACCTTTTTTATGTTTAATTGCCCCACCTCATGTTGTAGCACGTGGCATACAGAACAGCGTAGATCGGCAAGTGCATAATGGTTGTCAATTTTTTTTATGACGTCTTTGCGTGTTACTTCAATCTCGGCGGGTTTCTGCGAACTGTCTACGGCCTTGTAAACGTAAATGGTCCACTTATGAGGAAGCCCCCTTTCATCGTATAGGGTTGAAATGGGAAGCCTTTTATACTCAAACAGCCGAGTCCGTTGGTCGGCTCTTCCTGTGTTAGGATCAAATATGTTATAAAATCCTTGCTGAGCAAGCAGGGCCTTTTGCTCGCCATAAGCATTTTCGTACGTTTTGAATTCTGCAAGTTCGGAGTTAAAATTAGGTGCATTTTGTAAATACTTAAGAAATAATTCATAGGCTCTAAGGTAAATGAGAGTTGAGGTTTTTTCCTCATCCCGTCCTCCCCACCACACCCGCAGGCTTTCTTCTTGAAAATAGATGTCATTCAGACGCGTCAACTGCGTAAAATCAGGCCGATATTTAGAGGTATAAATTTTATCATAAAATTCTTTTTGCGATAATAGCTCGGCCGGGGTACGTCCTATCACGGTTTTAAACTCATATTCAGCCTCCTTGGGAGTCCGTGGTTTGTGCATAGGGATGCTGCCGTCAATACGGGCCACTGTGGCAGCATAATCATACATGGGGTCCAGCAGAATCTCTGTCAAAAGTACCTTGGTGTCGTCCTGCACGCTAAGCCCTTGTAGCCCATTTTGGTGGATAATTTTTTTGAAAGCCTCCCGAAAATTATTAGCAATCCACTGATCCGTAATCTCAGATAGCTGATTTATTATACCGCTATATTGCTGCATCATTTTCTCCAAAAGAAAGGTCACGTATGCATTCAAAGAGCTATCCGCCTTCATTCCATGAATGGTAATCGTAAGAAATTCTTTATTTTTTTGCGAGCTATAAATGAGATTCAAAATATAGGCATAGATGTAGATCACAGCATACAGCTGCGTTAAAGGATCGTAATCCTCTTCCTTTTTAATATTTTCGATGCTATACACGAGCGGCAGGCAGACATTTACGGCTATATTGGCAAACTGTTTCACGTCTACAAGCTTTCCAAAGTGGATAAACGTGCAGGCCTTCATGGTTTCCTGCCAAATAAAAACACGGAGCTTACTATTAAGATCGCCGATGATGCCCACATCTGCCGTACGATCCTCTTGAATAAAATGGGCCAGCTCTTCGCCACAAATTTTGCAAAAGTAGGAGTAAATAAGCCCCTGGTTGTTTTCTTTCTCCTTGTTTATTCCTGAAAATTTCATTAGCTTGGTTCGCATGGTGTCGTAGGACGCTTCTGCCGCTTGAAGCTGTATAAGCATGTCCACATGGGGACAAAGCAGCTTAAACCCGCAGGCTTTGCATAGATTCCAATTGGTGGTATTGTTTTTTTCCTTGTAGAGTACACGAATACTTTCTAATACTTTTAATAACTCCGCGTATTGAAGACCCGAACGCAACTGTTTTACCAGCTTGAGATGAGCACATGCATTTTTTTCTTGGAGTTCCCACTGTTTTTTAATGTTTAGGTATTCTGTTGTAATAAGTTCTGCCTCCTGTTTCCCACAGGCTTTAATGACTTCTTGAAGGATGCTGTTAGGGTCATCCACTTTACCCTCCATTGTAAGAATTTCACGTATAGCATCCGACTGCACCCTACCTATTTTTTCTTCCATAATTTTAAAATACTGTCTCGCCTGGGTAATGACCTCTGTGAGCTTCATGTCCACCTGCTGCAGAATCATTTGCTCCTTTTCACGCTGTTCAGCATGTTGTAAAAACTTTTGTTCTACAGGGTTCCAAAGCACCTCCAAATAGCCTGCTCTATATAGGTCATAAAGCAAGGGCATGTATCCCGATGTAAAAACCGGGGACACCGAGTACATCGTAGACAACTCTTTTAAAAAAAATATCACGCGCTTAATGTTCTCCTCCGGTTCAATCTCCTCGGTTTCAACGATATTAGATATATGACTGCCCTGATCCTCACGGTCTAGCTTTCGGTGTACCATCTCCTCTGCTAGCCGATTAATGAGCCAGCTATGCCCGCCGCTCCGCAAAAACTTATAAAGTTCGATATACTGGTGCGTAAACTGGATGATGTTTTCCTTGGTGGTTACGACAACCCCTTCTCCGTTTTTTTTCCAGGTTTCTTGATCCACGCATTTCATAAATACTCGAATAAAATTGGTCAAATTGGCTCCTGAGGCGACGTAGCCCAAGGTTTCAGGCGAGAAGGAGCCTATCTCAGCCATACGCATAAAACACTGCGGGGAAAAAGTTTTTAGCCGCAACTTAAGTCCATAGATTTCAATGGGGGCTTCTGCGGGAACGGCCAGGTGCGTCCCATTAATTAAAAAAATTTCTTTGCGTGTGCTAGGGCGAACACGTAATTCCTTTTTTTTTTCACTCACGATGGGGACCACATCGGGGTCTACCAGCAGTTGACGTATGTAGGCCTCTATGGGCATGGATAGATCGGGCAGCTTTGACTGCTCGGCGCGAACATGGTTCACAAAATCTTTTAGAGTGAAAAGAAAGTCTATTAAACGTATGTTTTTTATATCATTAGACCCTTTAAGGGTAGAGTAGATTTCATCCACTAGTGCCTCGATTTCCTCATTATTGAGCGATAAGATATCTGTGCCACGGTGGACTATTTGCGCGATCGTAATTACTTCCTCCATTAGATAGAAACTGAATATTATATTTAAAATAAATACAAAATGTCAAATGAAAGTTTTCCCGAAACGTTGGAAAACTTACTTTCAATGTTACAGACCAAACAGCAAAACGCAATTCAGTCAGAGGTGATTGAATGGCTGCACAGCTTTTGTGAAACCTTTCACTTAAAAATACACTGCCATAAACAGTTTATTCCTAGCGGGGAAAAAAAACGAGCTAAAATACCCGCTCAAGAAACACAGGGAAACACGCAGCCCTCCCACCATGTGTACCGGGTTGTTCTCTCCAGAGCACAGCCAGTCAAAGCACAGGAATCTCTGCTAACAACCATGTGCAACGGACTGGTGCTAGATGCAAACACATGGACATGCCTAGCCATTCCTCCGCCTGCGCCCTTTCAACAGGCGACCCGCCAGGTCCAACACTTTTACCGTAACAATTTCTACGAAGTGGTTCCCATCCAGGATGGCACCCTTCTCACAATCTACCACTGGGATGACCCTGAATATGGCCCCTCCTGGTGCCTAGCAAGTACCCACGGATATGATGTGAGTAACTACTGTTGGATAGGCGACAAAACCTTCGCCGAGCTTGTATACGAATTGCTGCAGCAGCACTCTACCTGCGACGTCACCCTGGAAAAAAATAAAACGCGGGGAACGCGTCTTTTCTTTGATAACTTAAATCCCGATTACTGCTATACGATTGGAATCCGGCACCATAATTTACAGCCGCTCATCTATGACCCTCAAAATATTTGGGCGATTCAATCTACAAACCTAAAAACGCTTAAAACGGTATATCCAGAATACTACGGCTATATAGGCATTCCAGGAATTCAGAGTCAAGTTCCTGAGCTTCCCCAGTATGATTTACCTTATCTAATACGATCTTATAAAACTGCTATGAATCAAGCCAAAAATGCTATAAAAAATGGCAAAAAAGACAAGGGATACTTTAATTATGGCTATTTACTCATTTCGCGAGCGCCTGCCATTACTAAAAGTACTTCTAATGTTTTGTTAAAATCGCCTCTGCTGGTATTTTTACAAAAAAGTGTGTACCAGAAAAAACACAATATCTCTAACAGCCAGCGACTAGAATTTATTATACTGCAAAACTACTTGATGCAGCATTTTCGAGATCATTTCATTGCTCTATTTCCGCAGTACATATCCTATTATACGAAATACCAAAACATGTTGAATATGATTATCCATAGTATTGCAACTAAAGATAAAGATCATCCCTTTGCAGGAGCCGTGGTAAAAAAAGTGTTGGAAGATATTGAAAACGCCGAAAACATTATTGATCATACAACCATTCAAAACTATGCCCATCAAAGCAAGTACGCCATGCTTTACTTGTCAATTATTTCCCATTTTTAATCTAATACGGCCAAAGCCGCGGGTTTTTTAATAAACTAACATTTAAAAAAACTGTTTTATTAAAAATTATAATACTTTTATTATATATGGAACATCCATCTACAAACTATACTCCCGAACAGCAACACGAAAAATTAAAACATTATGTTTTAATCCCTAAACACCTTTGGTCTTATATTAAATACGGAACGCATGTCCGGTACTACACCACACAAAATGTTTTCCGAGTCGGTGGCTTTGTGCTTCAAAATCCCTACGAAGCCGTTATAAAAAATGAGGTAAAAACAGCAATAAGACTGCAAAATAGTTTTAACACAAAAGCGAAAGGGCATGTAACGTGGGCCGTCCCATATGATAATATTAGCAAGCTATATGCCAAACCAGATGCAATTATGCTTACCATACAAGAAAATGTTGAAAAAGCTCTTCATGCTTTAAACCAAAACGTACTGACGCTCGCATCAAAAATACGTTAAATATAATTTTTGTAGAGGATAAAAAGCTATTTTAGCTAAAAAATAATTCATATACGTTTATGCAGAGGAAGAACGGTGGCTTTCAAATTCAGATTGCATCCACGTAGACCGTAGCGTTTTTTTTGCTTCTGGTTTATATCGTAAACCGTAATAAACATCATCATTTGTATCCGTTGGATCTTTTTCCCACTCCGGATAAAAAATCGGTTTTCTTTTTTTTTGGTCGTTTTTTGCAGTAAGCTGTAAATTAAGGGAATATAGCTTATCGAAAAGTTGTTCCTGATCCATATAAATAGCAGCATATATTAAAAAAAAATAAAAAAAGACGCTTCAACGAGTCAGTACCACTGCTTGCCAACGATTTACGTTGGTTGGTGCATTATGGTGATATAGTAATGAGTGCCTGCACAAGTGCTTGCACAAGTGCCTGCACAAGTGCTTGCACAAGTGCTTGCACAAGTGCTTACACAAGTGCTTGCACAAGTGCCTGTACACATTACTGCATCGCCAAAGCACCTGCAATGCCTACTTCCTCAACAGAGTACGATAACTAAATGCTTTTAAGCACCGCTTGCGTCGATGTGTCCTTCGGGGCAATCGGGTTCAATTGGATCCAATATTATTAGTCATAATTACCTAATACTTATTCAATTTTATCTTTTTTACCTTGTAAGATTTAAACAGCGTTTTAGCTTGTTTAAAGCAACGTTTAAAACAAGCTAAAATGCTGTTTAAAACAACGTTTTAAACAAGTTAAAACAAATAAGCTTATAAATATACCATGACAAAATTAGCCCAATGGATGTTTGAGCAGTATGTCAAAGATTTAAACCTAAAAAATCGGGGGTCCCCCTCGTTCCGCAAATGGCTCACATTGCAACCCTCACTGCTGCGCTATTCGGGTGTGATGCGTGCTAACGCCTTTGACATCCTAAAATATGGCTATCCTATGCAGCAGTCAGGTTATACGGTTGCTACGCTTGAAATCCACTTTAAAAATATTAGGTCTTCCTTTGCCAACATTTACTGGAACCGTGATAGCGAGGAGCCTGAGTACGTCTGCTGTTGTGCCACCTATCAATCACACGATGGCGAATACCGGTATCGATTTGTTTGGTACCAACCCTTCATAGAGGCTTATAATGCCATAGAGGCGGCCCTGGATCCCCTGGAAACCATTATCCTGAACCTCATTGCGGCACGAGATCTAGACTTCGTTGTTCACATATTTCCTTATAATAAGGGCCATGAAGACTATTTGGCCTCCACGCAACTTATTCTCAAAATCTTTATTGCGACGCTTTTAATGGACATTTTAAGAATTAAAGACAACACGTTGGACGTTCACTTAAATTCCGACTATATTATTGTGATGGAGCGGCTTTGGCCTCACATAAAGGATGCCATAGAACACTTTTTTGAAGCCCATAAGGACTTACTAGGGTACTTAATTGCCTTTCGCAATGGGGGGAACTTTGCAGGAAGTCTTAGACCCTCCTGTGGGCAAAAGATTGTTCCCCTAACGATTCGAGAGGTCCTACAAATGAATGATATTAATTTAGCCGTATGGCGGGAGGTGTTTATTATGCAGGAATGTTCCGACTTAGTCATCAATGGGATAGCGCCCTGTTTCCCCATTTTTAACACGTGGACGTATTTGCAAGGCATTAACCAGATTTTTTTTGAAAACACGTCTTTGCAGGAGAAATTTAAAAAAGATTTTATTGCCCGAGAGCTTTCCAAAGAAATTATCAAGGGCCAAAAAACGTTGAATGACAAGGAGTTTAAAAAGTTAAGTCTACATCAAATCCAGTACATGGAATCCTTTTTACTTATGTCAGATGTTGCCATTATGATTACCACAGAGTACGTTGGCTACACCCTTCAATCCCTGCCGGGTATTATTTCGCGATCCAGCTATTTATCCCCCATCGTGAAAAACATTTTGATGGACGAAGACTCTTTTATGTCCCTACTATTTGACCTATGCTATGGCGCCTACGTGTTGCATAAAAAAGAAAATGTGATTCACGCGGATTTGCACCTGAACAATATGACCTACTACCATTTCAACCCAACCAGTTTTACAGATCGCAACAAACCAGGAAAATACACCTTAAAGGTCAAGAATCCTGTGATTGCCTTTATAACCGGGCCCAAAGTCGAAACCGAAACGTACGTGTTCAAGCACATAGATGGGTTCGGCTGCATCATTGACTTTAGCAGAGCCATTATGGGGCCTAACCATGCAATTAAGCTTGAGCGGCAGTACGGCCTCGCTTTTGTAAACACCTTTTACCGCAATCAAAGTGAGCATATCTTAAAGGTATTACGGTACTATTTCCCTGAAATGTTAACCAATCGCGAAAACGAAATACAGGGGGTGATTTTATCAAACTTTAATTTCTTTTTCAATAGCATTACTGCCATTGATTTTTACGCCATTGCTAGAAACCTACGTAGTATGCTTTCTTTGGACTATTTACACACCTCTGAGGTGAAACGAAACGTAGAGATTTCGCAAACATTTTTGGATACATGTCAATTTTTGGAGGAAAAGGCCGTGGAATTTTTGTTTAAAAATCTTCATACTGTCTTATCTGGCAAGCCGGTTGAAAAAACGGCCGGGGATGTGCTTTTACCCATCGTATTTAAAAAATTTTTATACCCAAATATTCCTAAAAATATATTACGGTCTTTTACCGTAATAGATGTATACAATTATAATAATATAAAGCGTTATTCCGGCAAAGCTATACAAACGTTTCCACCCTGGGCTCAAACCAAAGAAATCTTGACGCACGCCGAGGGTCGTACATTTGAAGATATTTTTCCTAGAGGAGAATTAGTTTTTAAAAAGGCTTACGCAGAAAACAACCATTTGGACAAAATTTTACAGCGTATTCGTGAGCAGCTTGCTAATGAAAATTTGTAAGGCTTGCAGTTCTTGTATGGTCAGAACCTATGTCGATGGAAACATTATTTTTCGCTGCAGCTGCGGCGAAAGCGTTCAAGGGGATAGTCAAAACTTGCTCGTCTCTAGCAAGGTGTACCACACCGGGGAAATGGAAAATAAGTACAAGATTTTTATTAAAAATGCACCCTTTGACCCCACGAATTGCCAAATAAAAAAGGATTGCCCGAATTGTCATTTAGACTATTTGACACAAATCTGTATTGGAAGCCAAAAAATCATTATATTGGTGTGCCGCTGTGGCTATATGAGCAACAGAGGATAAACCATATCATCCCACCGATTTGTGACATTCCTTTAAAACCGTCCGCCTAAATAGTTTTCACACCTTTGGTGGCAGACTATTTTATAAAAAATAATGTTGGTTCATGAAGATAAAGTGTGCCAAAGAAACTTTTATAAACAAATGATTAATGTAGGTGCTAGTCGTGTGTACTTAAACAGGGTATTCTATAGCCAAGTATTTTCTATAGCCAGTATTAGTCAAGCATTTAGATGTCAGGGTATTTTTATAGCCAGTATTTTTCTATAATATGTACAAACTATTCCAGTAAACATATGTGTGTTCTTTATTGAACAGCATCATGGCATTAACAAGTTTATTAAACCGCTCTAATGGGCATTAAATGACAACTCGGTGCTTAGCAAAAGGGCCTATACCTTCTAGCAATTAGGGCCGGGAGGCATTCCCAGCTTTTTTCTATAATCAGCCATACAGTACCCCTGAGCCTCATACATGGGAATGAGGTCCTTCCATTCCTTGTTGGGATCGGCGGGCCAGCTCTCAAATGAGGTGTGAATGTAAGGGTCCTGTTCTTTTTCCTTAATGAAGCGTTTAATCTCCATTTGATGTTGTTTACTTTTTTGTTTGCGGCGGAGCGTGTTCCGCACCAATACGTAAAAAATACCAAGAATCACGCATAAAAGAATTATTAAAAAAAATATCATCATCGCGGGGTTTAAAAAACGATCCCATGCAACAGGAATCGTTCTTAAAACCTTGTCTGGCAGGGCTGTAAACATGAAGTCTCCTCCTATAATCGGGGTGGGGCTGTAGCCTAACAGTTCAAGGTCCTGTCGTTCTAGATACTTATTGGCGAACTGCCCACCCTTTGCCCCCGTTTTTTTATTAATCAAGCAGCGCTGCATTTTCCACCATTCTAAATCTTCAGGAGAAAGCTCAATGCCATATATCAATTTTAACGTTATTGCATCTTTTTCAATATCCTTATCAATTTGGCTGAGCTTTTGAGCTTTAAGCGGGTCTAGTGTGTACTTCCATTTAAACTTAGTGTCCTGTAGTTTGGCTACATGAAATACGGAACATTTTGGTGGGGCCTTTGTGACGCCCTTACACTGCGGAAGTTTATCATTAGGACAGGCGCATAGATGAGACTGCGCCACAGCATCGCGAACTACATCGCAGACGGAGTACATTTTCCTCCTATGTTAAACAATAAATTTTTTTCATAGCTGAAATTTGTGGGCCTATCTTTTCCCTTGCCCGGATAATAATTATAAGGGAGTGTTGAAACATCTGGGAGAGAATTGCTTAAAAAATGGGTTTTTGGGAGGGGTAACTGCGACTGTTGTACGTCGTTGGCCAGGGAGATTCTATATGCCGGGCTAAAGGTGCAACGTTCCTGTGAACAACTTAGTACGCGCGTTGTTAATACAAATGGACTGGTATTAGCAAACCTCGTAAACTCTTCCGGACTTGTTTGTTTTTGTATGATGTTTAGCAGGGAGTCTGCCTTTTCGAGAATCCAAAGCGTCGCATTGTAGTAAAATAAAAATAGCGACTTATCGGCAGGCGTTGCAAAAGCGCCGTATAGAAAATAAAGCAGTAAGTACTGGGGAGACACCACAATAAGGTTATCTTGAATGATAGATATCGCTAGCTCTTTAAACATAGTGCTAAAAAAATGTATGTCGTTCGTCTTGAATATAGGGGGACTATAGTCCATGTAGGGCTCACATATCTCAGTCAGGTGAAGGCCCATTTCTTTTATGACTTCTTCCGGGTTGTACGTCGCTAACACCAGCGCGGGATAGGCTTTGGGCATATCCACGGTAAGTGTTATGTTTTTATCATTCTTATGGTAGGAGTAAGATGGTTGTGGAAATTCTGTTTTCCACTCCGGGACTTTGCAGGTAATTCTCAGCTCATTTAGAGTCTGGTACAGGAGGGCGTATGCCGCAAAGCCGTGTATGGCCACTTGTTTAAAGGGAATTGAAAACGTTTTACTTTCGTATGTCGACTTCACAGGAACAACGGGAATGGGGTAATATTTTTCTATGAGGTTATACCGCTGCAAATCCTTTTTAAACCTGCTAAAAACATCTTCCCTTGGTGGGTTATCAAAAGGAAAGCAAAATGCTAGGTGTAGCCCGGCCCGCTGGTAATCGGGGTGAATGATTTTAAGGTTTTTATACGTTAATGTGGGTATGGTGTTAAAGATATTGGGGGGCATATATGAAAGATCAGCAACCCACACAAAGTCCGTGCGCACCCGCATGGTCTGCACATGGATGGCGCGCACCGTGCCCACCTGCTTGAAGCCCTTTTCATACAAAATGTCAGCAAGTTCGTAGGCGTCCTCAACGTGGTTGGGGGAAAACATATCAAAGTCGGGTCTTTCTCCCTCGGGATAAATTGAGCTGCCTTTAAGATGCAGGGCATAATCAATGGCAATCCCCCCGTACAAAATAAGCTTTTTCTTTATGATAAATTCGCGGACCACCTCCAAAGCCGCCTCAATCTCCACGGCATTTGCCTCACGTTTTTGAGCAATGAGCCGGTACTTAGAAACATTAAAATCAGTCTTTAGTAAAGACGTCATAAATAGTGTTTAATATATATTAAAGGTTTGAATAAAATACTAAATAGTAAAAATGGATGCCCTATTAAAGGAAATAGAAAAGTTATCGCAGCCATCCTTGCAGAAAGAAAACAATGATGTATGCGATCTCTGTTTTATGCAAATGAAAAAAATTTCTAACTATCAGCTTTTATGCGAAGAGTGCGGTCAGCTGAAGGACTGGTTTGAACCTGAATATAATGAAAAATTCACGGTATATTCTCGTCTAAAGATCGTGGGTGCCAATAGTTCCTATCACCAGCGCGATTTGGACAAGGCCAACTCAAGTGACTATAGCTCCTTGCAATTTCATCACATTTTAGAGGAGCTCAAATCCCTAAATGTTAAGTATATGGATGCGGGGCAAAAGCCCTTTCCTATTCAGGTGTTAAAAGAAACTGCTCACAGTTATAACCAAGTACAACAACATCGGGTCATACGCAGCATTACAAAGCTTCAGATCTTAGCCAGTATTCTACGTAGCATTTGTTTAAAATTAAACATTGCTTGTACGGTGGCAGACGCCGCGAGGTTTACTCAACTTAATACCAAAGGGATCTCAAGGGGCATGGATCTTCTGCGCTCCCTATTTGTAGACAATAAAATTACTTTAAACGTTGATTTAAACCCTATAGACAGCTTTATTAATAGTACCTACAGTGCCTTACAAATTAAACAAATCCACCAAGAACTGCAGGAGGAAAATGTTTATAATTTAAAAGAAATTGTTAAGAGCTTTATATTATACGCGGATGAGAAGAACATCGGCGTCGATCTTAACAGGAGAACCGTTGTGATTGCTACGATGTATAATGTTTTACGCCGTGCCTACTACCCCATAGAAATTGATACGGTGGTGTATCAATGTAAAATACGAAAAAATACAATTACACGTGCTCTTAAAATGTATGAGGATTACTACTCCCACTTTAAGTCTCTTTATGAGCAGTATCATTTAAACGCGGCAAAAAAATTAATTTAAACTAAACGTTTAAACTAAATGTTTAAACTAAACGTTAAAACTAAACATTTCGACTAAAGTTTAAAACCTAGTCTAACAGCGGGATGCCCATTTCCCTGGGGTTCCATATTTCAACAATTTTTTGACCTTCGGGTGTTACCTTGATGCAGCGCATGACGAGCAGTGGAATTTTCCTATTAAAGAGTTCTTGCTTAGCTATATCAATAGGACTGCTATATTTTTTTTTAAGCATTGTAGATCCATTAATTGCCAATTGTTGCGCTCTAACGGCGACCAACCTTGTGGCCTCAAAGGTGGTTAAAACGTTGGAGGTAATGCGCTCGTTATCGGGTATAATGACCAATGTTTGCGACGAGGCCTGCACAAAGCCCTCGCAGATGGACGGAGACTCCACGATCTCGTCCTTGTCCTCGGACTCCTCCTCACTGTCGACGAGGTTCTCCTCTTCCGTTTCCACATATTCCTCCACGAGGTCATCCATGATAAGATCCTCGTTGTCATTATCAGCCATATTACACTGTTATCAAATGTACTGTTTAATACGCAAATGGATTTACTACGTTTTAATTGTATGTCTTCATGTGCAGGCTCTAGTGGAAAGTAATTTTCTCACAATTTTTGGCACCGTTACACTTGTGCCCACAAAAACCCGCGATTTTTTTATTTTATATTACTTTTGGAAGTACGAGTTTAACCAGTCGCTTTCAAACCTTATGCGTCTATCTCGCCAAAAAACGCTCACAGCGGTGTTGGATATTACCTTTAAAAAAATAACATTAATTTTTACCACAGAGGGCGTATTGCGTATGGATTCTACGAATAAGCCAGGCGTGCCACTCGATATAGACCCCCAGTTCATTGACCTTGATAGTATTTTAATGGAACTGGATCATTAGGACCTCTCCCGCCCATTTAAATTTTTAGTTTCTACAATAATAAAATGCGCGAGGAATCATGGGAAGACCACGATACCATTCAGCTCACCGCTCAGCGCAAATACCTCGCCGAGGTGCAAGCTCTAGAGACCCTTTTGACTCGAGAGCTTTCAGTCTTTCTCACAGAGCCAGGCAGCAAAAAAACAAATATTATTAATAGAATCACAGGAAAAACCTACGCACTTCCCAGCACAGAGCTACTAAGACTCTACGAGCATCTCGAGCAATGTCGCAAGCAAGGCGCCCTCATGTATTTTTTGGAAAGACAGGGGACCTACTCGGGTCTCATGTTGGACTATGACCTTAAACTCAATACAAATGCTGTTCCCCCGCTGGAACCCCCCGCGCTATCACGGCTTTGCCATCGAATATTTGTGCATATAAAAAACAGCAGTGTGCTGCCTGAGGGCAGCCATAAAATCCACTTCTTTTTTACATTAAAACCTGAAGTGGTTCAGGGCAAATATGGGTTCCATGTGCTCATTCCTGGTCTCAAGCTGGCGGCTTCTACCAAAAAAAGCATTATAGGATCCCTACAGCACGATGCCACCGTACAAAAAATTCTACACGAGCAGGGCGTTACAAATCCTGAGTCCTGTCTGGACCCCCACTCCGCCTCCGTTCCCTCGCTCCTCTACGGCTCCTCCAAACTAAACCACAAGCCCTACCAACTGAAAACCGGCTTTGAGTTAGTCTTTGATAGCTCTGATCCCGACTACATTCCCATTCATCAAATAAAAAATTTAGAATCTTATAATTTAGTTTCTGAGTTGAGCCTTACGAATGAACAGGGAAGCCTTGTAAGACCTGTCTATTGCGCGGCAGACATTGCCGCTGAGAAGGAGGAAGAGATCCCGACCGAGGATCACTCGCTCTCCATATTAATGCTACATGATCCCGAAGCCCGGTATTTACATAAAATTTTAAATCTGCTTCCTCCGGAGTATTATGTAGAGTACCCCCTATGGAGCAACGTCGTATTCGCTTTGGCCAATACATCCGCTAACTATCGGCCCCTCGCCGAATGGTTTTCGCAAAAATGCCCTGAAAAATGGAATACGGGAGGAAAAGAGAAACTAGAAAAACTTTGGAATGATGCCTCGCACCACACTGAAAAGAAAATCACCAAGCGGTCCATTATGTACTGGGCCCACAAACATGCCCCCCAGCAATACAAAGAAATTGTAGAACAAGGCTACTTTTCCATTCTCGCTGAATATGTGTATAGCTATAACGGCATGCTTGAGCACTACATGATCGCCAAAGTCATCTATGCTATGATGGGCAACAAGTTTGTAGTGGACGTGGATTCAAACGGGAAGTACGTTTGGTTCGAATTTGTGCTACCGGGCCAGCCAATGAATCAGGGAGAAATATGGAAGTGGCGCAAGGAGGTAAACCCGGATGAGCTGCACATCTATATTTCCGAAAACTTTTCAAGGGTGATGGACCGAATCACGGAGCACATCAAATACCACCTCAGTCAACCCCATGAAAGCAATATTTTAAATTATTATAAAAAACTATTAAAAGCCTTTGAACGCTCTAAAAGTAAAATCTTTAATGACAGCTTTAAAAAGGGAGTTATCAGGCAAGCTGAGTTTTTATTTCGCCAAAGAAGCTTTATTCAAACTCTGGATACCAATCCCCACCTACTGGGGGTTGGCAACGGGGTTCTCTCCATTGAGACCATCCCGGCTAAGCTCATTAATCATTTTCACGAGCATCCCATTCATCAGTACACACACATATGTTATGTGCCCTTTAATCCCGAAAACCCCTGGACAAAACTATTATTGAATGCACTCCAAGACATCATCCCAGAACTTGATGCTAGGCTGTGGATCATGTTCTACCTAAGCACGGCCATATTTCGCGGCCTGAAGGAGGCTCTGATGCTTTTGTGGCTTGGAGGCGGCTGCAATGGAAAAACTTTTCTAATGCGACTTGTGGCCATGGTATTGGGCGATCACTATGCCTCCAAGCTCAACATCAGCCTTCTTACAAGCTGCAGAGAAACCGCGGAAAAACCCAACAGTGCCTTTATGCGGCTTAAGGGGCGGGGATATGGGTACTTTGAGGAAACCAACAAAAGCGAGGTTCTAAATACGTCGCGGCTGAAGGAAATGGTAAATCCGGGCGATGTCACCGCTCGAGAGCTTAATCAAAAACAGGAAAGCTTTCAGATGACGGCCACCATGGTCGCCGCGTCCAACTATAACTTCATCATTGACACGACGGACCACGGCACATGGAGAAGACTGCGGCATTATCGGTCAAAGGTGAAATTCTGCCATAACCCCGACCCCAGTAACCCCTACGAGAAAAAGGAAGATCCTCGCTTTATTCACGAGTACATCATGGATCCAGACTGCCAAAACGCATTCTTCAGCATACTCGTCTATTTTTGGGAGAAGCTACAGAAGGAATACAACGGGCAGATTAAAAAAGTGTTTTGTCCCACCATTGAGAGCGAAACGGAGGCGTACAGAAAGTCACAAGATACGCTACATAGGTTTATCACAGAAAGAGTCGTGGAGTCGCCCTCGGCAGAAACTGTGTACAACCTATCCGAGGTCGTGACGGCCTACGCGGAATGGTACAACGCCAACATTAACGTAAAGCGCCATATTGCCCTTGAGCTATCCCAGGAGTTAGAAAACTCTGTGCTAGAAAAATACCTTCAGTGGTCTCCCAACAAAACGCGAATTCTAAAGGGTTGCCGTATTTTGCATAAATTTGAAACGCTGCAGCCCGGCGAATCCTACATTGGGGTGTCCACGGCCGGCACACTCCTAAACACACCCATATGCGAGCCAAAAAATAAATGGTGGGAATGGTCTCCTAATCTCTCTGCCCCTCCTGAGAAAGAAGCGTCTGCACCAACTCCTTAGGAAATATCCTTAGAAGCATGTCTTTCGGCAGAGCCATTACCGGTAGCAAAAAAGCAACATTGAGTATATTATATGCCTTAGCCTGCTCATAAGCGTCCTTTTTTTTCATGGTATTTTATGTTTTTATATATTTTTAATTATTTTTTAAATACGATGAACAGTTCGTGCTCCGAAGGCTGTTTACTAAAAATCGGTGTGAATCCGCATTCTTTAAATATGGTTTCCCATTCGGGGATGGTATGGAAATCCATGTCTCTACGAATAGTATGGTGCCCAAGCGCGTCCTGCAGGCTGTGAAGCCAGAAGGCTTCCTGACCTTTATGAAGGTCATACACGATAAGAAAACCATCAGGTTTCAACAGATGGTAAAGCTTGTTAAAATCGTTTATCGTAAGATGATGCGCCGCCATAGGTAACCCTATGAGCTCCACAGAGTTTTCATGCTGGACATCGTCCATATCGGTATAAAACGTTTCACAATAAATGAGACGCTTAAACGAGTATTGATGACAAACATTTATTTCCAAGTAGGTTTGCACCACGTTTTTAGGTATATCGGGAATCATGTTGATTAAGGTTGTTTCGGGAAACTTAATCATCTGACTAGGCTTCATTTTCAACTCTTTAAAGGATTTCCCGGAGAAGTGAAAATGGGTCTTTACGTATTTATGTAAAAATACCTGAATGGGGAGAGGGGGCTCCTCCTCTTCGTCCTCGACGCCTCCCAAAATATTTGGAATTTCCTGACGTGGCGAAAGAAAGTTTATGTCCACGTTTACGAATCCATCGAGGACGGACACAAAGCTTGGCTCTAATCTCCATTCCATATACTGTTTAGAAACGGGAGATAGCATAATCCTAGGCGTTACAATGCACGAAGGATTTTTAATCACCGCATCGTGGTAAGAAAAGTGTATTCCATTTCTTCCAGTATAAAGAAGCCTATGTTTGTCGTAGCAGAAACAATTAAGGCGGTATGCCTCATACATACACTGTTTCAAAGTACAAACACGTTTTAAAAAGGTTTCTGCATTGGCGGAGGCCAAGCGGTTTTGCCATTGGTGGAAGGGGTTCAATCCTACAATGGCCAGCTCGTTTAAAATATCTTCGCGGCGCGCCAAAATCTGCACCATAGAAGAATATTTTAGCATTTTTTTTTCGCACCATTCGCGAAGATGTTTAGCTACATTATTAACCTTATTATTGATAAAGTATACGATGGCATGTTGGAAGCCTTCAAAAATAAAGAGCCCCTCCAAAAGATCATCTGCCAATAGAAGATGGATGTTGGTGTAAGCATTGTCAATATTTTGTAGAAACGGCGGAATGCCTGCCAAAACCGCTTCAGCAAGCATAGCTCCGTTCCGTTGTTTACTGTCCAATAGATTCGTAAGTTTTTTGTCCGCAACAGACACGACGGCTAGGATGGTTGCAATGTCAGAAATGGCGGCTTGCCAGAAATAACCCGAAAAGCACATGCGCGCTTCTTCTATAGATAAAAACGAAAAGCGAGAGGCAATGTCTCCGAGCTGCGTGAGTTGAAGACCTTTTTCTCCTCTGGTTAAAAGGCCTGCCACAATGGCTCGCTCAATGGCTGATGCCAGCGCATCCGTGGGGGGAGGATCCAGCATATCAATCTCCTCTGCCTTAAACACGCCTTCCTTATTTTTTTTAATCGTTTCTACGACAATGCTAAGAAAAATGGCCCCAGGGCCTTCCGTAATGATTTCAGGATACTGCTGCGCTGGTATTTGCTCAAAGACGTGTTTTGTGTAAAGCGGGTAAAAGTGCCCAGGAAATACTCTCCCTACGCGTCCCTTTCTTTGCTCGATACGGCTTTGAGCCGCGGGGCGCGTAATGAGCCCTCCCGCCCATTCAGGGTAGTAGGTTTCAATGCTTCTGTTCCACCCGGGATCTATGACGTACTTCAGCGTTTCAATGGTAAGGCCCGTTTCCGCAACAACCGTGGAAACAATGACCCTTCTTAAAGGTTTTTCCACTTTAGCGGTTAAGGGATTTTTCACCCACAGATTCTTAATTTCCGCTTTCAGGCCAAGGTAGGCCTCATTTTCCTGCGCAATCGCCTCACTATCGATCGGCAAAATCAACATTAACGGCAGCTTTTCTTTGGCAAGGTCCATATTTGCATTATTCAGCAACATCGAAAGGAAGCGTATTTCAGCCATACCGGGCATGAAAATTAAAATATCTGCTTCCGTGGGACGATCATGAATGTTTTCTTTATGAATAGTGAGAGCCGTTTCGCAGGCGGTCTTAATGTAGTTGTTGGTGTTATACAGCGGCCAGTGGGTTTCCACACCGTACTGTCGTCCTTCCACCAAAATAATGTTTTCTTTTCCGATACCAAAATAGGTTGAGTATTTATGGGTATCAATGGTGGCGGAGGTTAAAATTACAAAGGGAATACGCAGCGCCCCTATGCTTCCTCTTTGCAACATGCGCTGAAGCATACTTTTAATATACATGAGCATAAGGTCGATGCCTAGGGCTCGCTCATGGGCCTCATCTATAATCATAAAGGCATAGCGGGAAGCTATCTCATCATCCGTCATTGTATGTAGCTGCGCCAACAGAACCCCCGCGGTTGCATAGATAAGGCCCCGATTGGGTTTTTCCGTCAGAGGCTTCGTCTGGTAGCCCACTGTTTGGCCTAATATCATGTCGGGGTAGTGGGTTGAGGCGCCGATGTCTTTGGCGAGGGTCACTGCGGTTAGGACTCTTGGCTGGGTACAAATAACCGAGCGTCCCAAGTATTTTTGGAAAGAATGCGTGTTTTCATTTCTCAGAATTCTGAACACGTGTACGGGCAGGGCCGTGGATTTTCCGGATCCAGTGCGTGACTTTATAATGAGCACCCGGTCTGCGAGGGAGGTTGGAATGGCCCCTCCAAACTCCGGGAGACGTTGTTTTATCCACGTGATGATGTAATGAATAGGAACATCATTCTTGTGCTCAGCGGGCACGTTATAGAGATGACCAGGCTCCAATAAAGTCGGTTTTCCCATATTCTATTGTTTTAAGGATTGATTGTTCATAAATATTTTTATACTCTGACCAAGAAATTATTTTTTTATTAAGCCGGTTATTTACGTTGTTATGGAACGCGAAGGTCCAGTACTGAAAGTCCTCCGAGTTGTTTAATGTCAAGGGATTTTTTGTAAGATACGAAAAGGCGTGGTGCTGACACCTGGTGCATGGCAGAGACTCGATAAAGTTCAGTATCCATTGGATGGCTTCATATTTTTCTTTCCAGCTAGGAGCGTCTGAAAAAGAGATAGCATATAGATGCAAGGATCGCCAGTATTTAGGTCCCCAATGCAACATTTATAACCTTTTGAAAAATCTCATTCCATATAGAGGTAAATATTTTTTTTCCATGGAGAATTTTTTTGCACTCTTGAAGGGATTGCGCCACATCGTCAAATGTTTTTTGTTTTCCATGTATTTTGGCGTAATTCCAGCCAGTATCTGTGTCATGGTCCTTAATGTCATCCGCTAACTGAAAGGCATGTCCAAAACAGTGGGCAGCCCTTTCAATCATCCCAATGTCTTCAATCGTTCCTGTGCCCAAAACCCAGCCCATAATAAACGCGATCTTAAAAAAGGGAATGGTTTTTTCTGGAGTGTCTACTAACTGACCGGAACCCGCGCTGTTTAGAGAGTGGCTTACAAAGGTACACAGCAGCGCTCCCAGTTGGTTGGGATCCGGAAACCTTGGACAGTGTTCCTTAATCCAGTCGATTTGCCGGCAAATGTTTTGAAATCCTTGCATGGTTAGCGCCAGAGCGCTCATCTGCGCCTTGGCTACGCCAAAGCGGGCCCACACTGTATCTTTATTTCGCCGCTTCACATCGTTGTCAAAGGAGGGCATATCGTCGATAATCAAAGAAGCTACGTGAAAGTACTCCACTGCTAGGGCAGCCTCTGCCGGATAAATAGGCGCCCCAAAGGAATGTTGCAACTGACAGGCCCGAACAATTTCCATCAGGATAATGGGACGGATATACTTCCCACCTCTTAGAGCGTAAGAGCAAGGCTCTGTTAGTTGTCCCTTAAAGTCCCCATCTTCAATAGCATTGTTTAAGATGGTCTCAAACTCTTTACTAAAGGTTTTATAATTTTTAGGATTCAGTGGATGTATTCCATGAAAAAGCGCGACACTACGCGGAGCTGTGATTCTAAAATACTTAGGTTTGCGCGTATAGGATATTAAAATAATAATAAGAACTACAATGATGGAGATATAGATGAGATGCAACATGCTGAGTTGTCTCCCCGCAGGGAATGGTCCTTTTCCGCACTGGTTAACGGTACTGAGGAGGCGTTGAAATCTTTAGGAAAGGTGCTGTCTAGTTTGGAATCTCCAATTCCTCCCGTATATTTAGGTATATAATTATTGTGCCTAGAAATTGTTTGCTTTGAGGTATCAAAATATTCAGCCTGACCGCTATTTCTTTTAGAATAATTCGGTATAGGGCTTGAGTAGTTGGCAATACTCTTAAACCGGGGCACCAAGGTAACAATATTTTCCATATAATGGGTTTGATACGCTTTGTTTAAAAATGGGCTTACCGGCTTTATGCTTGTTAGTTGTGCATTGAGTACCGGTATGTCTTCTAGGATTTGTGGCTTTATAGAATGATTAGCAAACACAGAATGTAGTATATTAGCTACTTGTAGCATGTGTCTATTTGCGGAAAATTCCTGGTATTCTCTGCCGTGTTGCGAATCTTTGGGCGGAAGGGGACCAAGCATCGGCACGCCCGTGTAGGTACTGGTGGATTTTATGAGTTCCTGCTCTATGTTCGGTTTGACATGTGGATTTCCTAAAGGAATACCTCTACCTGCAATCCCTTTTTCTACCGACGCAGGTAGATTGTGCGCTAAACACAAAATATTGTACACGTCTTTGTGCGGAATATATCCGTTATAGTGCTGGCCCGGCATCTGATCGCCAAGGTGCTGCTCATGCTTAATGGTACCCTTTGTTTTGAGTTTAGGAATATCCTCGTACGAAAAAAATTTTGTGTGCTCGCTGAACCTCGTAGACGGAACCGAACTATTTTTTGGGTTTTTTAAGGAAGGCAATGAGGAAGGCTGGGTCAGACAATTTTTCTGTGTGCCCTTTAAGCTAGCCACCTGCGGAAATGTTTTTTTTTCCGTACGAACAACATTGCGCCTAATTAGGTTTTCCGTATGGGTTGAAAAAGCAGGACGATGATTTTTAAAATGATTAAAAAGTTTATTTTTTGGAATGGAGCTGTACGGCTCCAGATCTTGCGCATCGCCGTAACCAATGTTTTTGTGCTGAGGGTTCAGCATAAAAGAAAAGTTACGTAGATCACTGAGTTGCAATCCCTTTTCAGCCTTTTCAGGACTATTAGTGTATTCATTGTATACAGGCGCAGCTCCATTTTTGTTGCCGCAGTACCGGGAATTTAGTATATTATCAGAATACCGGTTATGACGCGGCAAATCGCTTTCCCAAAGAGGTGGATCTGACCTATAATCGGCTAACAGCTTTGAAGCATAATCATGATACATTGTATATAAAAGTTAATTATTATATTGAGAAGGCATAATTACCTCTTGTAGGGGTACAAGAGGCTTTGAATCAGCCGAACTGGCCGGCTTTGAATCGGCCGGCTTTGGACCGGCCGGCTTTGGACCGGCCGGCTTTGGACCGGCAGGTATCTTTTTAGGTTGATCTTCTTCTAGCTCATTAGACACGGATGGGGGAGAAATAGGAGGAATAATTTCATCTCCGCCCTTATATTTGTCGTGGATAGAAGAAACAATTACATCCATGTTTGATTTATTATAAATGTCGTTTAACTGGTGATTTAAAACATAATAATGCAAAAATAATAGGGCTACAATGCATATATATACGTAAATAGCCGTCTTCGTTTTTCGTTTTTTATCCACCGGCGGATTACAAATTGCAAAAAATACAACTAATACCACCGCTGTAATGATTAAGGCCACAATGAAAGGATTTTGAAAGGATGTTTTGAATGGTTCGCACGTATAAATTTTTTCTCCTAAATTATTGATACCCGCAATAAAATCTACATTCATTTTATATATTTATAAATTATGAAAAATTTAAAGTTACATCTCCGCCGGACCAATCATTGCTAAAATTTGAAGATTCTTCAAAAAGGCCCGACTGGTTGAATGTCTTCTGCTCAGGTTTCCAAAAATTTTCCAAGAATGGATTTTGAACAATAGGCTCATCTTGATTTTCTTCTTCAAGGATATTTTCTTTGGTATCAAGAACAGCTTCTTTAAACTCAGGTGTATCTTGATTAAACTCAGGTTTATCCTGATCAATCGCAAGAATATTTTCTTCTTCAGGTATATCCTGTTTAATCGCAAGAATATTTTCTTCCTCAGGTTTATCCTGATCAATCGCAAGAATATTTTCTTCCTCAGGTTTATCCTGACCAAACTCAACAATATCTTTCTCGCTAAATCCGTTTTTAGTGTGAAGTTCTTGGTTTTGAAGAGAATTATCAAAATCTAGTTTAGTTGTTGTTCTAGATCGTGGCACAGGATAGTTATCTGGTGGTTTACTTACTATAGTCCTCGAATGTGGCACGGGATAATTGTTTGGTGACTTGCTGGTTAGCTCTTGGCTTGTTAATAGTTCTTGTTTTCTCAATAATTCCATCTCTACTACTTCTTTTTGATCCGCCGGTGTCTCTTTTTGGTATTCTTCATTAGAAAAATGTTCAGAGGGTAATGTTTCAATAAACTTTGTGAGTGGATAGCTGCTCTTTGATGTAGAAGAGCGTTGAATTTGCTGATAAAGGAGTTGAACAAGTCGCCGGTATTCACTCTGTCTTTTTTCATATTTTTTACGTAGCGTGGAGAGATCCGCTAAGAGCGACTTTTTTTCAGATGTTAATTCTTCAATTTGATGAAGAAGGCTGCGATTGTATGAACTAAGTCTTGCATACGTTTCTTCTAATTCTGTCTCCGGCTCCACATAGGCCTGTTTTCGCAAAAATTTGTTGTATAGTTCCATTCTTTTTTTGAGCAGAAAGGTAAGACTATAATCTTGCATTTCTTTCGTAACTTTATGGTAGTTTTCTTTCCGGTTTTTGATAATAAAGGGCAGCATTTTTTCTGTTGTGATAAAGGTGCCCAAATTGCTAATGTAGTCGCACAGTAGCAATTCCAAGATAGATTCTTTCTTTTCAAGGCTTATAGATTGGCTGTATTCTTTAGGTATGAAAGAATCAACAATCGTTGTTATGAAGTTTGAAAAGTTTAATGTTTTGCTGTTAATTTGGGTAATGTTACAAAAATATTTGTAAAAACTATCTAGCATTTTTTCATAAAGTTTTTTATTTTGTTTAACCCCTAAAATATAGCCCTTTACTTGATACTGATATTCCGTAACAATGGAATGTTTTTTGTATAGTGCATTTTTGTATAAAAAGTTATAAAAAATGTTGATAAAATACGCACCAAGGGTTTCAAAAATACTTATAACGTGGGATTCTTCCTGATCCATTATATCATATGTAATATTATTTTAATAAAAAATTACTGACTAATAACATGCAAAAAAAATATGTTTAAACTTATTTTAAGCTAGCACTTATTTAAAAGCTTGTTTTAAACACGTTTTAAATTGTATGTTAATACAGTTAAAAATTAAGCCGAAATTTGCTCCAATAAGGATTACTTTTATCAATGACCACCTCTTTACTATAAACGGCTTTACATAATTTTAATAAAGCTTTGGAGCCAAAGCTGAAGGCAGTGGGAAGCGGCACTGTACTATGGTAAAAATGTTGCCGATTTTCATCCTCGCGGATGTACACAAGTTTCCTATATCCTTTAAACACAATATGGCTAATTTCTTCCACATACTCCTTATCCTGTTTGGAATAGCGGTTGCTTTGTCGGGAAAAATTCGACATACAAATAGAGGCATTTGTAAAAATGGAAACAAATGCGTTTTTACGAAGATTGGCGGGTAAATCGGTGTCATCTTGGCAGCAAATAATCATCGAAATAAAACAGTGACGATTTTGGTAAAAAAACTTTTTAAAAATTTCTTTTGTAAATAATGGGTGCAGTTCGGCCGCGCAGTCGTCTAATATTAAAAGTAAACGAGGATTAAGATTGATATAGTTTAACGTAAACTTTTCATCCTCTGTAAGGCATAAGTTTTTATACATATGAATGTTCTGTATAATAATTTTTTTTAAAAGTTGCTGATAAAGCGATGTAATCTTTTCTTCTTTTTTTTGGTCCGTTTGTTCAGCCTTTAAGCACTCCACTTTTGCAATATTTTTGTTTTCCTTTTGCTGTATATCGATCGGAAGTTTATGATACAATGTTTTTAGCATATCGATGTTGTTTACTCGACTGTAGATGGAGGACATCATAGTTTGCCGCTGCCAGATGGCCTCCAAAAAGCGTTCAGCGCCCTTGTTGTCATTTTTTTTTTGCTTATCGGCGAGCCACAAGCGGTAGTGAATTAGAGTTGGATGTACAAAACCCTCATATGAACGATTTGAGGGTTCCGAGGGGGCAACCACTAAAATTTGTTCAATATGGGGTTGCAGGATTTTCATAATATGTTTAACGTACACGGTTTTGCCTGTTTTTGAGGGGCCATATAGCACAGTTGTTTTATCTATAAAATGATGTGCTTTGAACTGTAGTTCAGGAATCAGCTTCCCTGAATGGGTCGTTAGGGCCATCTCTATATTATTACAATTCTGCTTTTGTATATAAAATTTCTTTTTCGAGTTTATTATTATTGTTGACCCACATATCTACCCGTATCGTATCATCAGGCACATTGAGCATTTCAAGCGCATTATTTAACTGTTTTTTTGTTTTTATCAGCTCGCTTTCTTCATCGGGGGTTAAATTTTCTTTACTAAGCAGTTGCTTAATTTTTTCTTCGCAGTCGTCTATAAAATCATACTCTCGAGCTTTTTTGATATTTCCAGATGCTTTTTCTAGGTTTTTTAGCTCCTTAAAGGAAAGCAGTCCCTTAATCCCGCTATCCGTGTGAAAGGTTGAATTATAGATGGAGAGCCCCGGAGCATCCGGGCCGGTTTCTTGTATATTTTTTGCTTTTTTGTGGTAAATGGTATTTCGTAGAATCTCTTTTCCTATCTTTAGGTCTTCCTCATGACGGTCCAAAATCCGTTTTATTATTTCATTATTTTGATTAAAATAATTGTAGCGCTCTCTGTTGGCCTTAAAGCTTCCCAGGAGTGTCCAGTTGCCTAATTGAATGGATGAAACCTCTGAGAAAATCTGGTCTTTATATTTATAATAAAATTCATCAACCTTTTGTTGGTTGCTGCTATCCACCACATCATAAATAATGAAGGCAAACTCTAGGTCGGGCTTTTCTGGGTAGATGCTTTCCGTAGCGGCCCGCAACTCTTCGTAATTATCCTCAATGTAATAATTCCACTTATAAAAAGTATCCTGAGGTGGAATATGCTGCGAAAGATATCTAGTAATTTTTGTGTTAAAGAGAATGGGTTTAAACGCCCTCGGATTTTCAAGCATATGTTTAATGCTTTGATGAAGTTCTATATTTTGTAATATGTGGGCTGCTGCCCTATAGCCCTGTGGGGTTTGGGTAATTGCATCAATATCGGCCTGAAGCTCATTAGGCACATTTAATGTTTTTTGCATGATGTGTAAAGGGATGCGCTCAGGATCTGCTAAATCGGTGTATTCTGTGCTTGCACAGGTGTTTGCACAGGTGCTTGCACAGGTGTCTACATTGGTATCTGCACACATGCTTGCACAAGTGTTTACATTGGTATCTGCACAAGTGCTTGCACAGGTGTCTACATTGGTATCTGCACAAGTGCTTGCACCTGTGCTTGCACAAGTGCTTGCACAAGTGCTTGCACAAGTGCTTGCACAAGTGCTTGCACAAGTGCTTGCACAGGTGTCTACATTGGTATCTGCACAAGTGCTTGCACAAGTATACGCACTTTGAGCATGAAGATTAGGATCAAACACAAAATGTTCTCGTAAAAAGCTATCGATCGTTGTTTTAGCTTCCTTGCTTTTCTGCGTCTGGGTTTTGCAGCTATCCGCTATAGATAAAATTGTATTTACTACCGATTCAGAGGGAACATCATTAGTTTCCTGTTTCAAAGTATCAACTAACGTTATTAGCTCACTGAGAAGAGTTTTGGTCGTGTGGGTAGGTTTTGAATAGGAAGGCATCCATTCCTGCAGAGCTTTGAAGACATATCCAATAAAGCTAGTCATTATAAGACGTCGAATATACTGCTCCCGCAAATTTGTAAAAGAGCAAAAGGCCACCCTGCTATCATTTTTGAACTGTTTGTAAGGGTTCGTCCTTTGATAAAGCTGTTTAAGCGTTTCTTCGGATATTTCAGTAGAGGGATCCTCCAATACGTTTTTGAGAAGCTCATCAATATTAAATTCTGCCATATCTTAGAGTTTATTATATACATATTAAAGCTTTAATATAAGGGGGGTATAACAATGGACGAAATCATCAATAAATACCAAGCTGTTGAAAAACTTTTTAAGGAAATTCAGCAAGGATTGGCCGCGTATGATCAATACAAGACCTTAATTAGTGAAATGATGCACTATAATAATCATATCAAGCAGGAGTATTTTAACTTTTTAATGATTATTTCACCTTATCTTATTAGGGCGCATAGCGGAGAAACGCTGCGAAACAAAGTAAATAATGAAATTAAACGTCTTATTTTGGTTGAAAATATCAATACCAAAATATCTAAAACGCTGGTAAGTGTTAATTTTTTACTACAGAAAAAACTTTCAACGGACGGGGTGAAAACGAAAAACATGTGGTGCACCAATAATCCCATGCTGCAGGTAAAAACAGCCCACAACCTTTTTAAGCAACTATGCGACACACAGTCCAAAACTCAATGGGTACAAACCTTAAAATACAAGGAATGCAAGTATTGTCATACCGACATGGTGTTTAACACCACGCAGTTTGGGCTGCAATGTCCTAACTGCGGTTGTATTCAAGAATTGATGGGAACCATTTTTGATGAAACACATTTTTACAACCATGATGGGCAGAAAGCAAAGTCAGGTATCTTTAACCCTAACCGTCACTATCGGTTTTGGATAGAACATATTCTTGGTAGAAATCCAGAACAAGAGTTGGGGACCAAACAAGATCCCTGCGGAACCAAGGTGTTGCAACAACTAAAAAAAATTATTAAGCGCGATAATAAATGCATCGCGCTTTTGACGGTCGAAAATATTCGAAAAATGTTAAAAGAGATAAACCGCACAGACTTAAATAATTGTGTTTCTCTTATATTGCGTAAACTTACCGGAGTAGGGCCGCCTCAAATATCAGAGTCGATTTTACTACGAGGCGAATACATATTTACAGAGGCAATTAAGATACGGGAAAAAGTGTGTAAAAAAGGGCGTATTAATAGGAATTATTATCCGTATTATATATATAAAATTTTTGACGCCATTTTGCCTCCAAATGATACCACGAATCGACGCATTTTACAATATATACATTTGCAAGGAAATGATACGCTAGCTAATAATGATAGTGAGTGGGAATCTATCTGTATGGAGCTCCCTGAAATAAAATGGAAGCCCACAGATCGAACCCATTGTGTTCATTTTTTTTAAAGATGAAGATTTTTTAGATGATTTTTTTTAGTTTTTTAAAAAGACGAAAAAATTTTTTAAAAGACGAATATTCTTAATCCCCGCAAATTACTTTTTTTTTAGGTACTGTAACGCAGCACAGCTGAACCGTTCTGAAGAAGAAGAAAGTTAATAGCAGATGCCGATACCACAAGATCAGCCGTAGTGATAGACCCCACGTAATCCGTGTCCCAACTAATATAAAATTCTCTTGCTCTGGATACGTTAATATGACCACTGGGTTGGTATTCCTCCCGTGGCTTCAAAGCAAAGGTAATCATCATCGCACCCGGATCATCGGGGGTTTTAATTGCATTGCCTCCGTAGTGGAAGGGTATGTAAGAGCTGCAGAACTTTGATGGAAACTTATCGATAAGATTGATACCATGAGCAGTTACGGAAATGTTTTTAATAATAGGTAATGTGATCGGATACGTAACGGGGCTAATATCCGATATAGATGAACATGCGTCTGGAAGAGCTGTATCTCTATCCTGAAAGCTTATCTCTGCGTGGTGAGTAGGCTGCATAATGGCGTTAACAACATGTCCGAACTTGTGCCAATCTCGGTGTTGATGAGGATTTTGATCGGAGATGTTCCAGGTAGGTTTTAATCCTATAAACATATATTCAATGGGCCATTTAAGAGCAGACATTAGTTTTTCATCGTGGTGGTTATTGTTGGTGTGGGTCACCTGCGTTTTATGGACACGTATCAGGGAAAATCGAACGCGTTTTACAAAAAGGTTGTGTATTTCAGGGGTTACAAACAGGTTATTGATGTAAAGTTCATTATTCGTGAGCGAGATTTCATTAATGACTCCTGGGATAAACCATGGTTTAAAGCGTATATTGCGTCTACTGGGGCGTCCAGGTATAAAACGCGACTGGCGTATAAAAAGTCCAGGAAATTCATTCACCAAATCCTTTTGCGATGCAAGCTTTATGGTGATAAAGCGCTCGCCGAAGGGAATGGATACCGAGGGAATAGCAAGGTTCACGTTCTCGTTAAACCAAAAGCGCAGCTTAATCCAGAGCGCAAGAGGGGGCTGATAGTATTTAGGGGTTTGAGGTCCATTACAGCTGTAATGAACATTACGTCTTATGTCCAGATACGTTGCGTCCGTAATAGGAGTAATATCTTGTTTACCTGCTGTTTGGATATTGTGAGAGTTCTCGGGAAAATGTTGTGAAAGGAATTTCGGGTTGGTATGGCTGCACGTTCGCTGCGTATCATTTTCATCGGTAAGAATAGGTTTGCTTTGGTGCGGCTTGTGCAAATCATGAATGTTGCATAGGAGAGGGCCACTAGTTCCCTCCACCGATACCTCCTGGCCGACCAAGTGCTTATATCCAGTCATTTTATCCCCTGGGATGCAAAATTTGCGCACAAGCGTTGTGACATCCGAACTATATTCGTCCAGGGAATTTCCATTTACATCGAATCTTACGTTTTCATAAAGTCGTTCTCCGGGGTATTCGCAGTAGTAAACCAAGTTTCGGTACGCATTCTTTGTGCCGGGTACAATAGGTCTTCCAAAGGGATCTACAAGCGTGTAAACGGCGCCCTCTAAAGGTGTTTGGTTGTCCCAGTCATATCCGTTGCGAGGAAACGTTTGAAGCTGACCATGGGCCCCCATCTGGGCCGTGCCCTGAATCGGAGCATCCTGCCAGGACGAATGACATGCACCCAATATATGGTGGCCCACCATATCATGGAAAAAGTCTCCGTACTGGGGAATACCAAAGGTAAGCTTGTTTCCCAAGGTGGGGGTACCCGTATGCGGGCGTACTTTATTGTATTCAAACCCTACTGGAACATAAGGCTTAAAATGCGCATTAAAATGAACCAAATGTGTTTCTTCGATTTGACTCAAAGTGGGTTCGGGGTCGGGTTTCCCATAACTTTTGTTCACATTTTTAATGTTAGAAATCCTGCTATTAAGCAAGTCTTGGGCCAATATAATCTTGTCGGCCTTCCCATCGTTAGCAATAAGACAAAAAGCTCCTCCTGATGCCATATATAATGTTATAAAAATAATTTATTGTTTTTATTAAATATGGCGGTTTATGCGAAGGATCTTGATAATAACAAAGAGTTAAACCAAAAATTAATTAACGATCAGCTTAAAATTATTGACACGCTCTTGCTAGCAGAAAAAAAAAACTTTTTGGTGTATGAATTGCCTGCCCCTTTTGACTTTTCCTCCGGCGACCCTTTGGCCAGTCAGCGCGACATATACTATGCCATCATAAAAAGCCTCGAGGAGCGCGGGTTTACTGTCAAAATATGTATGAAAGGGGATCGTGCCCTCCTTTTCATCACTTGGAAAAAAATACAATCCATTGAGATAAACAAAAAAGAAGAATATCTGCGCATGCACTTCATACAAGACGAAGAGAAAGCATTTTATTGTAAATTTTTAGAGTCTAGATGAGCTTTTACGCAATGTTGTACAGTGTTGTATATATGTCTTGTAAGCATTTGTTGTAGAGTAATAAGTAAAAGATAAATAAAAATGACTATTAAAATAAAGCCCAAACCATTAAAAATATTTTTATCTGTTAGATTTAATTTAATAAATGGCTCATGGAATGTGTGGTGCGCCGCTGCATGAGGTGCGGCCACGGCCGCATGGGATGTGGTCGCATAAGATGTAGCTACATGGGATGTGGCATTTGCTTGCATGTAAGGATCGTGATGTGTTGGGTCTTCATCCCAGCAATAATCGCCATCTTTATCTAGCTGAATTGTATACCCCATTATATATCACTTATTATTTTTTTTTAATGTTTCATGAATTTCATTATAGGCGGTGAAAGGGTCCTCAGGCCCCTTCTGTAAAAGATTATAGAGATCTTCGGACGCTTTATGTTTCGTGCGAATTAAGGCGGGATATAACAAAAGAGAGGGCCCCAGTTCCAAACAAATTTTACTTAGCGGGCTCATATTTTGCACCAAGTTTCCCACTACTTGCGATGTTTCATAACGCATTTTAAAGAGCTTTATCATAAAAGTGTTATGCAGGCCGGTGTAGTCTGGCCTATAGTTAAGGAAGGGGATTTCTCTGGTACCGTCAAACACGATCTCAAGTCCTCTAGCAAGCCCGATCAAAATTTCTTCAGCAATGGATGAGTATCTAATTCCTACATTACGAAGCGTAAGCATTTCTATAACATCATCTATTTCCTGCATAGAGGAATCTATTGTAGGAATTTTAATATCATCTGTGCTGATTTGTTCATTCCCAAGATAGGTAAGCAGCATATTAATTTTTTCTAGCTTTACTAGCTTAGTCTTACGCTCATAATCATGATCTTTTTTATAAAAAGAGTTGGGATCACCGTTGGACCGTAGATGATTAATAAGGCGGTCTACTTGCTTTGTACTAGGTTTAATACTTTTTTCACTATACTCGCTTTCAGCATAGTGGTTTTTACGATCTCTTTTAGAAATAGCTGTTTTTTGAGATGCCTCAGACTCTGCATATTTTTTTCTATGCGTAGAAAGAGAATAACCGCGGTCATTACGTGAACTACTGTTGCATGCAAGGCCTCGGCGCGTCTTACCGCTGCGCACACTGCCATTGCGTATACTGCCATCGCGCACACTGCCGCTGCGTATACTGCCATTGCGTATACTGCCGCTGCGTATGCTGCCGCTGCGTATGCTGCCGCTACATACACTATCACTACATATGCTGTCAGTACATACGCTATCGCGGCGTATGCCGCCGTGTACCTTATCGCCGCCCCTACCCGAGGGTTTTTTAGATATAATACTGTGTGGGGAGTCAAGCGAAAATTCAGGGTCATTAAAGTTAATGCCCAATGACTTTGCCAATCCATTAAGCTCTTCATCAAAATGATCGGTAGGAAAACTTTGTTGCTTGCCCATGACCTGTTTTTCAAGTTCCTCCAAATTGGCTTGCTCATTTATATGGAGATTATTCATAAGCGTCGTAATTCCAGCAAGATTTGCTCCTTCTAAAAATGTGGTGTCCTCCATCGGATATACTATACTATTTAAAAGCTTTTAAATAAAAATGTGTTTGGAAGAAATGCTCTCTTCAAGCGTGTGTAGCTCAGATATAAATGCCTCCTCAGAAAGCTTTCCACCATACTCCTTTCTCATCGTATAGGAGGGCGCCGGTTTAATGTAGGAAATCCACTGGGAGGTAAAAAACCGGTACAACATATTTAGCAGCTCGCGGGCCTCCCACCTTTTGGGCTCCGTATAGTGCACATCAACATAAGAGGCGGCGCATGAAAAGCTGCAAAAGTTGCCGAGAACGCCCATCTCAATCTCTCCTCGCTCATTTTCACGCATATAGGTGGGCACGAATTTTGGGACAGTCTTGAAATAGAGATGACATGTCCAGCATTTAAAGCTAGAATGGGTAACCCATTTGGAAACAGTGGTGAATACGGAGGGTAGCTTTTTTTCGACCTCGGCTTCATCGTCATTCGTATTTAACGTATCGGTGGCAGTTTTTTTGGATTGCAAGCATTCTTCAATGGTAATCCCGGATAAGTATAAAATATTAGGACAATTAGTTTCCATAATTTTGATAGTTATTTTTATACAACATGGATTTAATTAAAGATAAATGGAGGACGAAACGGAACTGTGTTTTCGGTCAAACAAGGTGACGAGGCTTGAAATGTTTGTCTGCACATACGGGGGAAAAATTACCAGCCTTGCATGTTCGCATATGGAGTTAATTAAAATGTTGCAAATTGCTGAGCCGGTGAAGGCATTGAACTGCAACTTTGGCCACCAGTGCCTACCGGGCTACGAATCTTTAATAAAGACTCCGAAAAAAACTAAAAACATGTTGCGCCGTCCGCGCAAAACAGAAGGCGATGGGACTTGCTTCAATAGTGCCATTGAAGCCTCCATTTTGTTTAAGGACAAGATGTATAAATTAAAATGTTTTCCTAGTACCGGGGAAATTCAGGTCCCGGGCGTCATTTTTCCGGATTTTGAAGACGGAAAAAACATTATACAGCAGTGGGTAGACTTCTTGCAACATCAACCCATTGAAAAAAAAATCCAGATTATTGAATTTAAAACGATTATGATTAATTTTAAGTTTCAAATAAACCCAGTGTCTCCCCGCGTCATCATTCATTTAAAAAAATTTGCAGCTTTGTTGGAACACATCCCTACTCCATATCCCATACGTGAAATAAAGCCTCCATTAGAAGACTCAAAAGTATCCGCAAAATTTATGGTCAGTCCGGGAAAAAAAGTACGCATTAATGTTTTTCTTAAAGGTAAGATAAATATTTTAGGCTGCAACACAAAGGAATCCGCGGAGACCATTTATACGTTTTTGAAAGATCTTATCAGCGTACATTGGCAAGAAATTTTGTGCGTGTTACCGGTACCCGATTAAAGAATGTTTTCATTAATAAGGTAATCGACTATGCTAAAAAGAATAACAAGAAAAATACCTTGAAGAACTATACCAAAGTAGGTAGGTTTTCTGCATGTCACGGCATGGTTAAAATTGCTAATAATGTAGTCCACAAAAGCATTGCTCAATACGACTAAAAATAGTAAAAAAAGGATAAGTGCTCTTTTTATATCCATATACTTTAAAACTTATTTTTTACACTAATAATTTCCTGCGGCCGCAATATAAACTGTAGGTCATCTATAACGCCCAGACCTGTTAAAAGTAGAGTACTATGTTTTAAGGGATTTAAAATATCCGCCGCAAGAATGTGAATATAATTTTCAAAGTGGTTTACAGGAATGCGTAAGCGTTTTTTTTTGCACTGCGGTTGGTTTAGGGTCGAATACTGGCAGGAGGTATATATATTAATAAGACCGCGGTCGATGGTTTCAATATCTTCATAGAATTCAATGCGCGGCGTCAAAAGTTTTTTAAGATGTTGACATAACTCATCATACGTGTAGGACTGGAGGGGGGAAAGAAGGGTGTAGTCAAAGTTAAAAATGTTTTTTTGAAGAACCTTTAAAGCATGTTCCGCGTCCGTGGTTTCCAAAATATGTTTTATGGTATGAATGTCATTTAAATCTACAAAGTCTGACAGCTTTGTGTAGAACTCGGTGACGGAGGTTATTTTCTGGAAATCGGTTTTTTGAAAAAGATTTTCAATGTGTTTGCGGGTTGAGTTGCTTTGCAGTCCATACAAGACATCAAAAAATTCAATCAGCAAAAACTTATACAAATGGTTAATATAAAAAGCTTTGTTGGCCTTATTCTGCTGAGGATATGGTTCCTCTAGGGGATATAGAATGGCTTGGTCTATATCCCTAGGATCAATAGTCAATGTTGCGATGGGAAGCTTTTCCAGCGTAGCGGGAAGAGTTTGGGTTGGAGCGTAGTAAAAGTATAGCCCGGTTTTTCCCTCTGAAAGAAAGCCCACAAATTCTTTTTTTATATTTTGCAGCACCGCTGAGGGTACGATTTCGTACTGTTTATACTGTTTGTTGAAAAGGGTAATAAATTTCCAGGTTTCTTCAAAGCTTGCAATCTGGGTGGGCCGCAGATCAAAGTCGATGGGAATGTCGTCATGAATGTAGGATGATAGTCTTATAGGAAAATAAATAGGGCGATCGGTGTCTGAATCGATAAGTAAAGCATAACAAAAGTTATGCCTGTTGATAAGTTTTTTACCAACCGTGTAGCCGGGAATGTTTTTCACGTCATGGATATCCCACCAGTTATCCTTGCACATAAACTCGCTCATAGACTGGATGACCTCCATCACAGGGTCATCTTCGGTAAAAATATACTGGGCCTCACTGTTTTTCAGAAATCTTTTTTGCTGGGTGATGGCCATTGGGTAGATCCCTTCGTCCGTGTCAAAGATAATGGCTATCTTCTTCGATGGGCTAAGAATTTTTTGTATTGTGCTGGGGGACACCTCAAACCCGATGTCGCCCTGTTTATCTTTAAAAAAGACACAGTGAAGGTCGTAGCATATGGCAACAAGGTCCAGAAAGATGTCCTGCCATGTGGTGTCCCATTGAAGCAGTTGGTTTTTTTGTTCAACAAAGGTTTGTAAGATAAGGTTTGCCAGCTCCGCGCCGCTGGAAAACATGTTGCCGGCCCCATTCCCCAAAATATAGTACTGCGGTGTGTTGGCCGCCTTTGCAATTTCAATGGCAAGGGCCTTGGGGGCAAGATCCAAAATTCGAGCAAGGGAATAAAAAAGCCCGGCATTGCTAATTCCAAGCATGGTTTGCTCCACCCCCACAATGCAAAAAATGTCGGGCTCTTTTATCGTATTTAAAAACAGTTCATCTGCTATCTGGTGGGGTAGAAAGGCAATCCGGTTCACCGGTATTTTTTTTCCATAGGACAAGGTATGACGCGATGTTTGTGTATTAAGATCCTCCAGGTCTTGTTCTACAAACGTGTGCTTGGTGAGGCAGGTATTGTTAATATAGAACCGCTTTGTGCCCAGCAGGGCCTTCGTCTTTTGGCAGCACGGCAGACAGTAATTTAGGGGGTGGCGGCCTTCTAGTAGGCTTAGATGAGGGTAGTCAGGATGCGGGCAGCTATAGTAGGCAGGTACCCCCTCCGTGAAATTCCAATACTTTACTAGCTCCTTGCGCTTGGCTGGCGGCATGGACTTCACCTCGGCCTCTGAGTAAATGACGGGTGGCCGTGGGTGCTGGCATAGGACGGAGTAAACCGTTGCCTGCGTGTCGTACTTGCGCAGGTCATACAGGTCGGGGTCCTGTTCTTGAAGCGCACGTAGCTGAGAGGCTCCCTTTCCTTGTTGTTTATCGTGCAGTTGAGAGAGTTTATTAACCAAAATTTTGTCAGGCCCGGTGATCAAGTTATCTAAAAACACAAATAGGTAAACCCAAAGATAGTTAAACTCTTCCTGGGTAATGTTAAACATTTCTATTTTGATATCTGTAACCCTATGGTAGATGCGAATGTTGCGGCCGCCGTAGATTGTTTCCCACCGGGCCGCAACATTTGTGTCAAAGAGGTACGCATACGTGTTTTGGAGCAACGCAACATTGATGTCCATTTTGCGCCCCGGACCGGAGGAAATAATGATCATCCGTTCGATTTCGTGGGGATCATACGAATAAATCCCCTTTTTAAATAAAAAATTGTAGACCCCGGTTTGCTGGAGGCCCCGCACGGAAATAATCCCTGCTTGCTCGTATTCCCGCCAACGACTTTTGAGCTCGGTAAATCCCTTGCTAGAAAGCGTATAGGGCCAAAAGGTGGACACCGACATGGAGCTGATAGAAATTTGGATGTCCTCGTTGGAGGGAAGGGGCAGACTCCCTCCACGAGGAAACGCGGCAGGCCCCATATCATTAATTGTATGAATAATAGGATTTATGAAATTATTTAGGGTGGACACCACGGAGTTAAAGTCGTGGCGCTCGTTTTCTGACCAATTGCTTTCGATAAAGTAGTGCCCATTATTTTGTATGGTAAGAATAAAGGCCTTTTTATTGATAAAGCGTATTAAAATAATAGTGGGTACACGGAATGTTTTATTGCTGAATTTTTCAGGCTCCGTGGAAGTTATGTGGTGTTTGGAAACCACGGTGGGACCTGTTTTACTATAAAAGAACACCACCAGCTGAGGAATATCGGGAGTAGCTGGAAATAGGTCGAAAACATTGCGCACATTAATTTGAATATTTACGAGGGGTGAAATTTTAATCATTGCCGAGGTGACGGCCAACGTGCCGCGTGTTAGTCTATTCCCCTCGTACTTGGCAATGACTTGTTGTGCTCTGGCATACGTAAAGTTTATTAGTTTTTGCTCTAGGAGAAGCCTCTTTTTAAGACTGGTCAAGGATGGAGAAAGAGCAGGATACTGTTTTTCCATTTGTAAGGGAGATTGTACCAATAGTTTAAAGGCATCGGGGGAAAGAAGAGGCCAATACTTCATAATAAGGCCGTAATAGAGTAAGTCAAATTGGTAATTATCCTCTATGGCAATGGAGATTTGGCGCCGCATGGGGGCCACTAGCGTGTTGAGGTCTGCTACAAAGATGTGATGAATGTTTTTTATGAGCTGGAAGCTGTCGAGCGCTTCCACATAGAGCTCATCTTTTTGACTTTCCATAGATGCGTCGATGTTCACCCCACCCACCTGTTGAAACTCCTTTTTGTAGTCGCGAATGTCTAACGCCACCCCGCTACCGCTTAACAATAGGCGATACGTTACCTGAAGCGCATTGTTTTGAAAAAAGAAAATGTGTTGTCTATAAGGGGGGATCCCTGTGGCAACGTAAATTTTTTCTCGAATGTCTTTAAAAGTGTCTTCAGGGAAAATACTATACTCGCTATACATCGTCTCAATTTCTGGCATCATCACGTTTGTCTCCTCGCCACGATCCTCCACAAAAAGTTTTTCAAACTCATCTAAATCATCGCTATCTCCACCCACCACGTATTGGGAAAGCTTTTTCTCCCAATCCTCGCCGTAAAAATTTTGTAAAATTTCTTTGTCCTTAGGGGTTCGCTGCAGGTCTTTGCGGCAGGCCTGTAACACGTTTGCAGGAACGGATCCCAAAAAAATAAACGTCTTCGTGTACTCATTTTCCACAGGATTATAAAGAGTAACTCGTAGAGGATTTGTTAAAAAGTCATTTTGGAAATCCATTATACCCGGTATAGAAAATAAAATTTAAAATAAAAAACGGATGATATCTATCATGGACCGTTCTGAGATTGTTGCACGGGAGAACCCGGTGATTACCCAACGAGTTACAAATCTCCTACAAACCAATGCTCCTCTACTATTCATGCCCATTGATATCCATGAAGTACGATATGGAGCCTACACACTTTTCATGTATGGTTCCCTCGAAAACGGTTACAAAGCAGAAGTAAGGATTGAAAACATCCCAGTTTTCTTTGACGTACAGATTGAGTTCAATGATACAAACCAGCTTTTTTTAAAGTCGCTACTGACGGCTGAAAATATTGTGTATGAACGGCTGGAGACGCTCACCCAGCGTCCTGTAATGGGGTACCGCGAGAAGGAAAAAGAGTTTGCACCATACATTCGAATATTTTTTAAAAGCCTGTATGAGCGACGAAAAGCCATTACTTACTTAAATAATATGGGCTACAACACGGCCGCGGACGACACAACCTGTTATTACCGAATGGTTTCCCGAGAATTAAAACTACCTCTTACAAGTTGGATACAGCTTCAGCACTATTCCTACGAGCCTCGCGGCTTGGTACACAGGTTTTCCGTAACCCCCGAGGATCTTGTTTCCTATCAGAATGATGGCCCCACAGACCACAGCATCGTTATGGCCTACGATATAGAGACCTATAGCCCTGTTAAGGGAACCGTTCCGGACCCAAATCAGGCAAACGACGTGGTGTTCATGATATGCATGCGCATTTTTTGGATTCACTCCACAGAGCCTCTAGCGAGCACGTGCATCACCATGGCACCCTGCAAAAAGTCCTCAGAGTGGACCACCATTCTATGCTCCTCTGAAAAAAATTTGTTGTTAAGCTTTGCTGAACAGTTTAGCCGCTGGGCTCCTGATATATGCACAGGGTTCAATGATTCTCGGTACGACTGGCCCTTTATCGTTGAAAAATCTATGCAGCACGGTATTCTAGAAGAAATCTTTAACAAAATGAGCCTTTTCTGGCACCAAAAGCTGGATACCATTCTAAAATGCTATTACGTAAAGGAAAAGAGAGTCAAAATCTCGGCCGAAAAATCGATCATTTCCTCCTTTTTGCATACCCCTGGATGCCTACCCATTGATGTCCGCAACATGTGTATGCAGCTTTACCCTAAAGCCGAAAAAACAAGCTTGAAAGCGTTTTTAGAAAATTGTGGGTTAGATTCGAAGGTAGACCTGCCGTACCATCTCATGTGGAAGTATTATGAAACACGAGACAGCGAAAAAATAGCCGACGTGGCCTATTACTGCATTATAGATGCCCAGCGCTGTCAGGACCTTCTGGTGCGCCACAATGTTATCCCCGATCGCAGAGAGGTAGGAATTCTGTCATACACCTCGCTGTATGACTGTATCTACTACGCGGGAGGACACAAGGTATGCAATATGCTCATTGCCTATGCCATCCATGATGAATACGGCCGTATTGCTTGCAGTACCATTGCCCGAGGTAAGCGGGAACACGGAAAATATCCCGGCGCCTTTGTGATAGACCCCGTTAAAGGGCTTGAACAGGATAAACCCACCACAGGTCTCGACTTTGCGTCGCTGTACCCCTCACTCATCATGGCCTACAACTTTTCGCCAGAAAAATTTGTAGCCTCTCGGGATGAGGCAAATAGCCTCATGGCCAAGGGTGAATCTCTTCACTACGTCTCCTTTCACTTTAACAATCGTCTCGTGGAAGGATGGTTTGTGCGGCATAATAACGTTCCTGATAAAATGGGATTGTACCCAAAAGTACTCATCGATCTACTTAACAAACGGACCGCCCTTAAACAAGAGCTTAAAAAACTAGGTGAGAAAAAAGAATGTATCCATGAATCCCATCCTGGGTTTAAGGAACTACAGTTTCGCCATGCCATGGTAGACGCGAAGCAAAAGGCGTTGAAAATTTTCATGAACACGTTTTACGGCGAGGCAGGTAACAATTTGTCGCCCTTCTTTCTGCTTCCTCTAGCCGGAGGAGTCACCAGTTCGGGTCAATATAATCTTAAACTTGTCTATAACTTTGTTATCAATAAAGGTTACGGCATCAAGTACGGTGACACCGACTCATTATACATTACATGCCCAGATAGTCTTTATACAGAGGTAACAGACGCATATTTAAACAGCCAAAAAACGATAAAACATTATGAGCAACTCTGCCACGAAAAAGTGCTTCTGTCTATGAAAGCCATGTCTACACTATGCGCCGAGGTGAATGAATACCTGCGACAAGATAATGGCACCAGTTACCTACGTATGGCCTACGAGGAAGTACTCTTTCCTGTGTGCTTTACAGGCAAGAAAAAGTATTATGGTATTGCTCATGTAAACACACCCAATTTTAATACAAAAGAATTATTCATCCGCGGAATAGATATCATTAAGCAGGGTCAAACAAAACTCACCAAAACGATAGGAACGCGAATTATGGAAGAATCCATGAAACTACGCCGCCCTGAGGACCATCGCCCCCCTCTTATTGAAATCGTTAAAACGGTTTTGAAGGATGCTGTGGTTAACATGAAGCAGTGGAATTTTGAAGACTTCATCCAAACAGATGCGTGGAGACCGGACAAAGACAACAAAGCAGTCCAAATCTTTATGTCTCGCATGCACGCTCGGCGTGAGCAACTAAAAAAACACGGCGCTGCAGCATCGCAATTTGCTGAGCCCGAGCCGGGAGAACGCTTCTCCTACGTTATCGTGGAAAAACAGGTACAGTTTGATATCCAGGGCCACCGCACAGATTCCTCCAGAAAGGGGGACAAGATGGAATACGTCTCTGAAGCAAAGGCTAAAAATCTTCCTATTGATATATTGTTTTATATCAACAACTATGTTCTAGGCTTGTGCGCGAGATTCATTAATGAAAATGAAGAATTTCAACCCCCTGACAACGTCAGCAATAAGGATGAATACGCTCAGCGCCGAGCTAAATCCTACCTACAAAAATTCGTGCAATCCATTCACCCTAAAGACAAGTCTGTCATTAAGCAAGGCAATGTTCATCGACAGTGCTACAAATACATTCACCAAGAAATTAAAAAAAAAATAGGCATCTTTGCCGACCTTTATAAGGAATTTTTTAACAACACCACAAACCCCATCGAAAGCTTTATTCAAAGCACTCAGTTTATGATACAATACTTTGATGGAGAACAAAAAGTAAACCATTCTATGAAAAAAATGGTTGAACAGCATGCTACGGCTAGTAATCGAGCTGGTAAGCCCGCTGGTAATCCAGCCGGCAATGCGCTGATGCGGGCTATATTTACGCAGCTGATTACGGAAGAAAAAAAAATTGTACAAGCCTTATACAATAAGGGGGATGCAATACACGATCTTCTCACCTATATCATTAACAATATAAATTACAAAATTGCCACGTTTCAGACGAAACAGATGTTGACGTTCGAGTTTTCCAGTACTCATGTAGAACTGCTATTAAAGCTGAATAAAACGTGGCTTATTTTGGCTGGAATTCATGTGGCAAAAAAACATCTGCAAGCTTTTTTGGATTCATATAACAATGAATCGCCGTCTAGAACATTCATTCAGCAGGCTATAGAGGAAGAATGTGGCAGTATTAAACCATCTTGCTACGACTTTATTTCCTAATACTTCTTAAGAAACTCTTTAAACAAGGACTTCGCATGGTCAAAGGTTCTAAACCCATGGCCCTTATGATTCGCCAAAAAAGCGGTTTCATCAAGATTTTCTAACCCTTTCACGGATGAAGAAATAAGGTGTTCGGCCTCGTTTGCCCATTTTCTATGATTTTTTTTCACCTCGGGTTCTAGATCTGTTTTCTCCATATACTCATTGTGGTCATATTTTTTTTTGGGAGGAGGCGTGGGTGGAGGAATGGGTGGAGGAAGTACACCCGACTTTCCCGCTTCAACCGTTTTATAAAAAAATAGAAGCATAATACAAAGAATAAGGACTATCGCAAATATGATAACCAGTGTCCCAGTCGAGGGCATTTTGTTATATAAGTAACGTTTTTTTTTATTTTTTATAATTCGAATGAAGAACCATGTTGAATAGTCTTCTACTCAAAGACATTTTGTTATACGGTAAATGAGAATTTATAAAATCCGAATATCACTATCATACTGTTTATCTGAGAAGGTCTCACTGGGTCCTGTGATGGAGAACCCATACTCTGTAATGCTGGGGTTTATAATGTGGTCAGGACTGACAAGCACATTTCTGAACTGCGAGAGTTCTAGGTTTAGACGCAGTCGTAATAGTCGCTGTATATTTGTAATAAATATTAGATTGCGTATGAGGCGAGTGTCAAAGCGATCCTTTCCAATTTGTACTAAGGTGGGCTTTTGTATTCCAACTCCCACTTGTTTAACGATGGACCAGGGTCCTTCTTCCCGATTTTGTTCCGTGATATAGGTCAGCACACTATTTTCTGTATATGAGGTATGATGTCGCATATTAATACCTGGTGCCATTCCAACTGGCGGTTGTGCAATTCGGGCTGTACCGGGACCCAACCATCGTGGAGTTTTATAAACATATCGTTCTAGCGTATTTAAAAATTCCTTAAGGTTATTTACGAGTAGCATGAAGGGTGCTATTAAAACAGGTGGATGGTTTATAACCATTGTCATAAACCATTGCATTGCTTCAATATCATTTTGTAATGCTTGACGGGGAGGCGGGGCAGGTAATCCACGTATGTTGAATAAAGCGGTTAATTGTGCACCGGCTGTTTGGGGCGTAATATTTTGTATTAAATTTATCATCGAATTGGCTTGCCCGGCATTTCCTATAAGATCGATTAAATTGGTTATTTGACCTCGATATTGTTGTACCCAGTTTTGAATGGCAGCGATGATCTCAGGGGTTGGATTGTTTTGAATTTCAGGTGTTTGTATTAGATTATTCACTTCTCTTCGTGTATCTTCAAGCTGAGTCCTAAATGCATTTAACTCGCCTATAATTTGGTTTCTATCAATAACATTTCTTAAACCTCGAACTGTTTCAGCCAATCGTATAGTACGCACAATTTCATGTAAGGCCTGGTTTATGTATATTGACATGGGATGGCCCCACCGCTCACGTCCACGTTGAATACCTGCGGCCAAACTAGGACCTGCCTCGTCATAATCAAATTGTGTAGGATAAAGGCTTCCAAATAGCACTTTATTGAAAATTTGGTCAGAAAGAAATTTAGGGCGGCCCATATTTAGCGCGTTGTCCCCTCTAAAGATGCGTGACATGTATCCGGCGTTGCCTTTGGATAGTAACTCATTCCCATATTGAGTAATAGAGACCGAGACATAGGGGTTTATAAGAAGTTTTAGCATAAATTCTCGAGTATTTATGGGGGGACGATTCGGAATGTTTAATACCTCTGCAACATCTGGTTGAGGAGCCGTGGTGTCCAGAGATCGTACTTTTTCAGCCGAAATGCCGTACATAAGACAAGCAATTTCTTCAAAACTATAGTCATAGTTGTAAATATTGGCAAGTGGTATAGATCGCATCAGCGCATTTACATTGATAGGTATAATATTCATATCAAACAAGTTAAATATGCGCTCGCGCTCTCTATTAGAGCCAAGAGTGCGTGTTTGACCTTTCGGCGACACTATTTTGTGAATATGATTGATTTGCTCCTCTTGGTAAGAGCTTTCCACGAAGGAAATTACGTCTTGCAATGTTTTACGAAGCGAATACACTGCATTCATCCCTATTCCCGCTGTTATAATGGGTTTATCGTCTCTGTTCTCGCTAATAAGATTAACTCCACCAAAAGTATTTTCATTGTACATCATCACTGTTTTAAAACTACGGATATTTATGATAAATCGGAGAGCCTGAATGGCGTGGGTATAAAAGTGTTCAAATCGCGTGGGAGTAATTTGTTCGCGAGCAACTACCGTTTCATTATAGTTTTTCATGATAAGCTGTACTCCGGGCATATCTGAGAGCTGTACCGGATCATTTCCCAGTAATTTTCTTGTGCCGTATAGTAGTTTAAACTCGGGGGAGCCGCTTTCAAGGTTCGGGTAAAGAAGAGGATCATATACCTCATTATTTTCTATTCTTAGGTCATGTAAATAATAGAGCGAAAGTGAAAATGGCATAAGAGGCTCCTTATTGTACCGGGACATATAGTTTTGAATGAAGTGTTCTTCTGTTTCAAGATAGATGGGATGATCGGTAAGCTCGTGCAGGACCTCCATGGCAGAATCTGCCAGAGTGTGAGAGCCTCTAATGATCCCGTCGATCACTGCGACCAGTCGCTTTCGCACAACATCGCTCGTATTATTTTGTGCGTCTCCTAGGGGCATAAGCGTAACATTGGGACGAAATACGCCGCCAATTCCCCGCAGGGCCGCCTGACCGACGGATAGTCCTGTCGCAGGAACATTGTTATTATTATAATAAATAACGGAATCATTATTGGCTCCCAAGAGTGCCGTCAGATTAGGGCGAGCTAGTTGGACATTTGTGTATTGTATAAATTGTTTTAGAAGCTCTCCCTGGCTAATAAGAATATTAAACATTTTGTTAAATAGTGGAAGATTGGCTCTATAATTTTCTTTAAGGTAAATGGGAATTTCTGTTAAAGTAGAAATAAGATGCTGACTCAGGCCCTGGCGATTGGTATCCTTAATAAGCCGCTGAAGTATAAGTCCCAAAGACAGAAGAAGCACCGACTGCTCTGTGGGGTCGCCCCTATGGCCAAAGGCGATGTTATCACGTGCTAGGTCGGGATGAGTGTACCCCAGCTCCATCACCGCTTGGCTAAAGTTCCCGTTGGCGAATGCATTGATAAGATTAAGATATATTTTTCCGCTGGGAGCGTCATAAAACCGGGCAATGTACGAGGCTACAAGCTGGTTAAACACCATCATCATACTACGATTATTTTGAATACCATAGTCTGATCCATATAGGCGATAACGTCGAAGGTTGTTTGCGGCATCATTGACGTTGGCATAGGTTCTGGGCGCCATATTGTCCCAGTAGCTAAGAGTATTTTCCTCCTGGGCATTGTTGGTACGAATAAGATTGGAGAGTCTAAAGTCTCCTAGTACCACTTGCTCTACACGGAGTCCAGAGTTATTCTCCAAAGCATCGTAAAATACGAGTCTACTAAATACTCTTCCATATTGTTCAAAGCGCTCAGAGGGTTGGGGATTGTTATTTATTTGAATATTAGCTGCATCTCTTCTTTGTACCCCACCTCGAAGTTGCAGTACATTATAGGGTTTTGTAAGCAAGATGTAGGTTTTATTAATGATTTGGTTAACTCCCTCCAGGCCTAATTCACCGCCAGGAAGCGGCCTTCCCCCGGCATCGGTGGGTGGTTTAATAAGCTTGTCAATCAAATGTTCTTCCAGCCAGTAAAATGAGCCAGGATTGGATCTATTTTCGTAGTATTGAATAATGTTTTTATCAATATGTGGGCGTAGAAGATTAAGAAAATACTTAGTGTCGGCCATCAAAGAATCAATTAAGGAAATAAGACCCGTAAAATCTAAGTGCACTTGAGCGGTGCTAGTTTCGGGAAAGCGAACTTGAACCATTTTGTTAAAACTAGAGGTCATTTCGAAGATATTGGTCAACAGGAGCTGCATGATTCGCTGATTGTCTACCAAATATCTTGCGGCTAATTCTTGCTCCGCACGAACTCCTCCACCAGCAGGAATACCCACATATGGTACAATCCAGGCAAAAAGAGTCTCTGTGACTAAATTTTGGTCTTGGGGTGCAGTCGCATTGGTAGTTGGATCAGGATACACCCTAGAAAGCCGCACATCATTTTCCTTAACAACCAATCCTGGATTTCTAATCTCAGAGATGGCCCCGTGTTTTCTTCCGAGCCAGTCAATAAGATTGGCTCGGTTCACGTTAGCGGCTTGCGTTTCTCGTAACCATTCAATGATGCTTTTTTGAATCGTATCTAGGTCTAAACCTTTAATGTTATTACGAAAGTTATTAAGAAGTACGTAAATAGCACTTAATAAGTTAAGACCTGTAATAACGGTTTCATGAAACAGAAATATTTTGTTAACATCTGTATCTGCCAGTGACTCAGAGCCTTGAATAAGTTTTGAAACAATTTGAATTTTGTCGGTATGCTCCTTTTTGAGTTCATTGATAGCCTGGCGAATAAGTTCCTGGTAGGAAACTTTGCCCAATTCTTGTTGTAGGCTGGGATCTTCAAACATCTCACTAAGCTGTTTCCTAAATTTTTGTACCAAGTCCCACTGGGAGTTGGGCTGCAGCATTCCTGTTTGGACATCCACAGAGTCTATATTGTATAGTGCCGGGCGCCACTTGGGGGTAGGTTGGGTTGAAGGACTAATAAACCTATCGGAGGGAAGTAATTGCGAGGATTGTGTATAGCCGTCCTCATCAGGAAGAATGGAGTAGTTGGTTTGATTCATCATTCCGAAGTCATTCATAGTTCGCGCTTCCTGAACAATGCGTTGAAATTTTTCCCATTCAGTGCGTGTGATAACGCCGAACCTGCGGTTTATTTCATTTACAAAATGGATAAGCGCTTTTTTGGTTGCTTCTTGTTCACCATACTCTAAGTTAAAGTGTTGGTAAATGACGTTTATTTCTTTGATAAGCTGACGAATTTCGGTTTCTGAGTAGTCACCAATGTTAATAAGCTCAATAGGACGCATAAAGATAATGCGAATAAGTCCTGAGAATATTCCTTCCAGCTCAGGAAGCATCGAGATCTGTACATTTTCATCTCTGAAGGAAAACAGTTTTTGATAAAACTCGGCGAGGCGAGGAAGGCGGAAGTAAAGTTCCGCTGCCTCGGGAATTACCTCAGGTTCTAGCTCATCGGCGCCTCCCAGTATCATACGTGTGGGTATAAGTTTGTACACAGGCTCAGGCCGTTCAAACATATCATAAATGCCTAATACAATGAAAATCTTAGCGGCCATACTTTTCAGCATGAAGGTGAAGAAGACGTCCTCGGTTTCCCAGCGGGTTGATAGGGCGTCGTTAACTCTCACAGTAGAGAGGTAGACCCGCTGAGCCGCTTCCTCGGGAGTTCGTGCAAGCGCTACCCTTTGCCCTCCAATTTCAGATTGATTTAGATTTTTAAGTCCCACGGAAAGCGCAGAATGTTGAATATATTCAAGCAAGGTTTTATAGATCTGCAAGGGCGACATGGGCACCATTTGCCGCAGCTCCTCTCCCCCAAGCATGTCTCCAATCCGGGCAAAAGCATTGATGATATTTTTAAGCGCCTGAAAGTTGGAAAGAGAACGCCCGATAAGGTCGCGAATGTTTTTAGCCTGGCTTGCTCTGACGGGACGGAGGGTACCAACGCTTCGGCCTTGTTGGATTTCAGCCGCAACTTTTTCGTAGTAGTGGCCCGCAGGAGCATTATCCGTAAAGACGTTGGAGTCGTTACCTGCGGAGGTGGGAAAACTTTCAAAGACTTGTGCAAGCGTGTCCCCTGTTGCCTCGGTGAACCATCGTCCTATAATGCGCACGCCATCCAACATCTGCTGGACTGTTTGAATAGAATCTATGTTGTTTACAAACGTTTTGGTAATGTTTTTAAGATAAAGGTCTAGCCCTTCCAGAGCTCGATAGAAGCGGCGTTTTACATCGTACTCCAGCTCGATGGCGCTTACGGTTGCCTTCCAGTCTACTTCCTGGGCACCTCCAGGATTTGGGCCCACGTGTCCTCTGGCAAGATCTACAGCCGGAGAATTAATGCGCGCATTTTTTTCCGTATCCAACTGCATGAGGCGTCCCGCAATAGCATCTCCGAGAATAGTGGCATAGTTTTCCTCGTAGGATTGAAACTCCTGTTTGTTATGCGTTAAATTGGAGTAAATCTGGGCCACATAATAGTAATACATAAAGGTGTTAATTGCCTGGTTGAGGTCAACCTGCGATCGCGCGGCCTTGCTGAGCCCAAGCTCTTCAACTGTTAGGGCAGCACCGCCTACCCTTGTACACTCGCAGTCCTCCTCGCCTCCATACTTTTTTTGCACAATATCGGTATAAAAATCAATAATCTGTAGCAAGCGAGAGCAGGAGTCATAAAGATTTTTAAAATTAGGGTCGGTTTTAGATATCTCCTCCAAAACATTTTTAACAAGCGTAAGCTGTGTTAAGAAGGTTTCGCGTTCTTCTCGTGCGGCCGCATTGGTGTAAAAGCCGATAAGACTTAGATCAAGTGCGATGGTGCCCATATCATTAATGCGCGAAAGAGCATCTCGAAGCCTCGTTATGTTCGGCGTCAAGGCAATTTCTTTAACAAGTTTGATGCCTATTTTTTTCACATTTTCCAAAAAGTCGTTATAGGCTTGTGTGCTTTTATTCAAAAATTCCATGAGGATGTGCTTTCTATCCAGTCTTTGCGCTTCAATCCTCCTATCTAGTGGCGTTTTCTCCTCATCGCCCCCCTTTTTGGCACAACTGTTCTCAAGGATTTTGTGGCGTTCATTAAAGGTCTGTCGCAACAGGTTCACGGCTTTTTCAAACTCAGCAATGTTTTCTGCGGAGACAAGACCACTAAACCTTTTGAGGTCAAGCTCCTTGTCAAACTCCGCCCAGTTTTTGCTTTGAAGGTACTGTTCAACCTTGAGTCCTACTTTCTGGAGAGCCTTATTAATTTTATTCGCAACAGACGCAGCAATACCTAGATTACAAAGTGTGTACGAAAGTACTTTTCCAAAATTTTTGGTTCCCAAGACACTATTTGTATCATTTAAAAGTTTAATAATATCCACCTCATCCGTCTGCAGTTTATCAAGTTCCTTTTGGGTGGGAGTTAAAATATTGTCAATAAAATTCGTTAAAATGTTGATTTGCAGGTTTTGTTCATTTAAAAGTCGACGATATACTGCTTCAATCATGGTGACTGCATTAATGACTTCCTCATTGGGGGCTGCTTTGGTTACCTCCGTCACCATGCGCTCGTGAAGTTGCTTAATGGCGTCGTTTAACAGCTTGATATTTTCAAGTGTATTTTCTATACTGCCGTGTACATCAAGATACTCTGCGCGCAGTCCATGAGTTAGGGAGTTAATGTACAGAACTATTTGTCGACATATACTGGCGGCCCCTTCGGTGGTATCTATAAGCTTATCCTGACCTAAATCAATAAATTCCTGGTTAATGGCGTCTGCAATCATTTTACAGACGGTCTCCTGTTTTTCCGCATTTTTTACAAAGGTGGAACCGGCTCGAGGATCGGGCAGTTGTTTTTTGATATCTTTAAGAATATCTTCGATGGGCTGCTTTGTGTCTACTTTGAACCCTATTTTGGCAATCGCCCTGATAATTCCTTCTATAATCCGCAGCTTTGCTTTACTCGATACGGAGTCTATGTGATAATCTTTAATGTGTTGTACAGGATTTTTGTCCCCCCCGCCATTAAAATATCCTCCCCCTGAAAAAGGACGAGTTTGTCTTTGTATATGATCCTGTAACTTCGCATATATATTTGCTTCTGATGAAGGCAGTGGTCTACTAGAGGTTGAAGATCCACGGTTACCCATTATAATAAAAAAAAATAAAGATTTAAAACTACAAATATTTTGCTGTTTATAAACCCAATCATATAAGACTAACTAAAACATTAAATGTAGGTGAGATAAAAGCTTATTTTTTTTTTAAAAGTTTAATAACCATGAGTCTTACCACCTCTTTTTCTTCTTCCTTTAGAGGGGTTCCATAAATGGTTTGAATAAAATTATGTGCTCTAATAACCTTGTTAAAATCAGGTGCCTTTCCATATTGTTCAATATGTTGCACAGTCTTTTGTGCAAGCATATACAGCTTGGAGTCTTTAGGTACCTCCGATGAGGGCTCTTGCTCAAACAACGTTTCAAAGGAGGATGTGCATTCATTGGTTTCATTATCATTTTTTTCATGAATGTTCTCCGAAGATGCTGAGGATTCCGTCTCCTCTTCAAACAGCACATGCAGAATCATATTCCATTCTTCTTGAGCCTGATGTTCAGTATACCCTTGCCCTGCATATATACGAGCAGATTTCACAATATCATACTTAACAGTACTAAGCAATGTTTTTATAGCGGTCGTAACAATTCTACCGCTATTGATAATCTCAACAGAAAACCAATTATACAGGCTACCCGCATGAAACACAACTTGTGAAGATGATCTTAAATCCGTTTTGAAGATGACCTCCATTTTCATGGATATATTTAAAATAAAATCCATTCAATTTTAAAATTATAAAATAATAAGAAGATGCCCTCTAATATGAAACAGTTTTGCAAGATTTCTGTATGGCTACAGCAGCACGATCCAGATTTATTAGAAATTATCAACAACTTATGTATGCTTGGCAATTTATCCGCGGCAAAGTACAAACACGGAGTTACCTTCATTTACCCCAAACAGGCAAAGATCCGCGATGAAATAAAAAAACATGCCTACTCCAATGACCCTTCACAAGCCATAAAGACCTTAGAATCACTCATCCTTCCATTTTACATTCCCACTCCAGCGGAGTTCACCGGGGAAATCGGCTCCTACACCGGAGTGAAATTAGAGGTTGAAAAAACGGAGGCGAATAAAGTTATTTTAAAAAATGGAGAAGCGGTCCTAGTACCGGCGGCCGATTTTAAGCCCTTTCCTGATCGCCGACTAGCGGTCTGGATCATGGAGTCAGGCTCTATGCCCCTGGAGGGTCCCCCCTATAAGCGGAAAAAGGAGGGTGGGGGGAATGACCCGCCGGTTCCTAAGCATATCTCGCCGTATACTCCGCGCACGCGTATTGCCATTGAGGTGGAAAAGGCCTTTGATGACTGTATGCGTCAAAACTGGTGTAGTGTCAATAATCCCTATCTTGCCAAGTCGGTCTCCTTGCTGTCTTTCTTGTCGCTCAACCATCCCACCGAGTTTATTAAGGTACTGCCGCTTATAGACTTTGACCCCTTGGTGACCTTTTATCTACTTCTTGAGCCCTATAAAACGCATGGGGATGACTTTTTAATTCCGGAAACCATTTTATTCGGCCCTACCGGATGGAATGGTACAGATCTGTATCAAAGTGCCATGCTGGAGTTTAAAAAGTTTTTTACCCAGATTACTCGCCAAACCTTTATGGACATAGCCGATTCGGCTACTAAGGAGGTAGATGTTCCCATATGTTACTCGGATCCCGAAACCGTACATTCCTATGCCAATCACGTGCGTACTGAAATTTTGCATCACAATGCCGTCAATAAGGTTACAACACCTAACCTCGTCGTGCAGGCCTATAATGAGCTCGAGCAAACCAATACCATACGACATTACGGCCCTATTTTCCCGGAAAGTACCATCAACGCACTGCGTTTTTGGAAAAAGCTGTGGCAGGATGAACAGCGATTTGTTATCCACGGCCTGCACCGCACGTTGATGGATCAACCCACCTATGAAACCTCTGAGTTTGCAGAGATCGTTAGAAATTTACGGTTTTCGCGTCCCGGCAATAACTATATAAACGAGCTTAATATTACAAGTCCCGCTATGTACGGCGACAAGCATACCACCGGAGATATTGCGCCCAATGATAGATTTGCCATGTTGGTGGCCTTTATCAACAGTACTGACTTTTTATACACCGCGATTCCCGAGGAAAAGGTAGGGGGGAATGAAACCCAAACCAGTAGCCTTACAGACCTAGTTCCAACACGGCTACACTCTTTTTTAAATCATAATCTAAGCAAACTTAAAATCTTAAACCGCGCGCAGCAAACGGTTAGAAATATTCTTTCAAATGATTGTCTTAATCAACTGAAACATTATGTTAAACACACGGGAAAAAATGAAATACTAAAGTTACTTCAAGAATAAGTATGTTGATACCTGTGGTGTGTTTTACCTGTGGGTTTCCTATTGGAACCTACGCGGCAATTTTTGACAAGGCTCGTACCGAGTATATTAAAACCAAAATGGGCGGAACATTGCCGCAAAATATCCCATTAGATGCTTCTCTCCAGATTGAGTTAAAAGACCTCATTACAGCTCTGGGAATCCCAATGCGGGTGTGTTGTCGCACTCATTTAATTACTACGTTGGATTATCGTAAATATTATTAATATCTAAAATTGAAAAAATATTTTTAATGTTACTAGTAAAAATGACTACACACATCTTTCACGCAGATGATCTCCTACAAGCATTGCAACAAGCAAAAGCAGAAAAAAATTTTTCATCTGTATTTTCTTTAGATTGGGATAAATTACGCACAGCGAAGCGTAATACAACGGTTAAATATGTTACGGTCAATGTCATAGTAAAAGGCAAAAAAGCTCCGCTAATGTTTAACTTTCAAAATGAAAAACATGTAGGAACCATTCCTCCCAGTACCGATGAAGAGGTTATACGGATGAATGCTGAAAATCCAAAGTTTTTGGTGAAAAAACGTGACAGGGATCCCTGTTTGCAGTTCAACAAATACAAAATCTCGCCGCCATTGGAAGATGATGGTCTCACTGTTAAAAAGAATGAGCAGGGTGAAGAAATATACCCCGGCGACGAAGAAAAATCTAAGTTGTTTCAAATTATTGAACTGTTAGAAGAAGCCTTTGAAGACGCTGTGCAAAAAGGTCCTGAAGCCATGAAAACGAAACATGTTATAAAATTAATTCAAAGAAAAATTTCTAATAGCGCGGTTAAAAACGCAGACAAACCTTTGCCGAATCCTATCGCACGCATTCGTATTAAAATCAATCCCGCTACAAGTATACTAACACCAATATTGCTTGATAAAAATAAGCCCATTACTTTACAGAATGGTAAAACAAGCTTTGAAGAGTTAAAAGATGAAGACGGCGTTAAGGCCAATCCGGATAATATTCATAAGCTTATAGAATCGCATTCTATACATGATGGCATCATTAATGCTAGATCTATTTGCATCAGCAATATGGGCATTTCATTTCCGCTTTGCTTGGAAATGGGAGTTGTAAAAGTTTTTGAAAAAAATAATGGGATTGATGTGAACTCCATTTATGGCTCAGACGATATTTCAACTCTTGTTAATCAGATTGCTATTGCTTAAACAATTTGCTCAAAACAAGCTTATAAACGTTTCTTAGGTATGCGATACGTAAATCCTAATTCTTTAATAAGTTCTTTTTCAGTAGTGATTTTTAGAGGTACTAAAGTTTGATTTTTAAATAATCCATACTGATTTAGCTTATAATTCTTTTTTTTTAACGCAGCTCGAATTCTTATTAAATAAGAAACGGGACCCGTAAAATGAAGTACTGCGTATGGCTTTTCCTCGGCTAAGGCCGTAAAAAGATCAAGTTGATATGTGTTTTTTTTCCATTCAATAAAAAGTACACACTTTCGTTCTCCGCAGACTTTTACAGAAAAAGAAAGATCCTTTATGCGAATGTTGGGCAGGACGTGTTTTAAAAGTTTTTTTTCTGGAACAATAATAAGAAGATCCACGTCATTAAGCATTTTCTCTTCGCGTCTTAAGCTACCAACAGCAACGATGTTTTTTGATAAAATTTTTATAAGTTGTCCATTATATTCAAACGCAAGTCGGGAGCGTAAGTCATTTACAATTTTTTTTCCTTGAATAAGCGTTAACATTTTATATTTAATATTAAAATCTTTTCATTTTATATATTATATACGCAAAATGGCACTTGATGGTTCAAGTGGTGGAGGCTCTAATGTAGAAACATTACTTATAGTAGCAATCATTGTGGTTATTATGGCAATCATGCTTTACTATTTTTGGTGGATGCCCCGCCAGCAAAAAAAATGTAGCAAGGCTGAAGAATGCACATGTAATAACGGAAGCTGTTCCCTAAAAACAAGTTAAAACATGCAATTATATGCATGCATATAAACGCATGCATATAAACGCATACATATAAAATGCGTAAATACTATATAAAAAACTATAACATATCAATCAAGGAATCAACACTTTTATAATTTTCCGTAATATATTTTTCATCCATAATGATGTCAGAGTACATGGTCCCTATGCGAGGAACAGAGCCCATAAGGGTAGGCGCGGCAATACCGTAAATGGGATTCACGGCGGAGTCAACCGCAGCATCTGTCAAGACCTGGACTGGAGACGACAAGGCCATTCGCAACAACACGTTGGAAGGCTCTCTTGCATTAAGCCCTGCCTTTTCTAGAGAGGTAACCTGTCCCGTTCTTGTCATGAGATCTGCGTACATGAGTAAATGACGATGGTTGGGACCCTTGTCCCCCATAACCGTTCTAATTTCACTAATAATTTTTTGCCGTGCCGCTTCTATGCCGTAAAGCTCCATGGTGTCTCCTATAGAGGACGATACGATGGTGTATGGGTCGATGTTATCATCAAGCATTGCGCCAAAAATATTAGTCCCGTTTGTTTTGATGGCGTAGATATTGTCTAGTCTTACCAGTTTCCCCTGGGCATCCACACGGTGGCGCATAAGCTTAACAACATTCGCATTTTTGATGCCTGGTATTCCTCTAATCGTGCTATTTAATAGTTTATCCACCACATTTACGGCAATTTTTTCATCCGTAGCCATTCGGGTATTGGTACTGCGTCTAAAGGCGCTTTCCCGTAGGTATATGCGAATAATGATGGGAATCCCTGAGGCCGTGTTTTCCACAGAATGCATGATGTAGGTGTTGGGGTGTTTAGCTCTTAGACTATTAATAATACTTTCTAGACTAATGCTTTTTAATATCATGGTTGTTTTGTTTAATTCCAAGCGGATACACCAGTTTGCAATATCCTCTGGGGGCTGTAGTAGAGGATGGTTTTCCAGAAAATCCGTCATCCATTCCACATCACTTGCAAAATCGGGGTACATCACATTTTTTTTTGTGCTTGAATACGTTTCGTACAATAGGTGCCACTGCAATATCAACCGTTCGAACGTTATAAGCTCTATGCTGTTAGCAATTTCTTGCGCATATGTTTTATTTGTTTCCACTTCCGGGTTCTTTAGACGTAAAAGCATTTCAGAGGATTGTTCAGCCTCTACGGGCTTCGCGCTAAAGATCTCCTGGGGCCGCACAATTCCCGACTTGTTGGTTCCCCCGGCCACGGACCGGTGGTGGGAGTCCAGCATATATTGTGTCAAGGGCTCTGATACGGACTGCGCCGCCAGGATTCCCACTGCCTCACCGTAGTTAATAAGACTTTGAGTATATTGTAGCCTTATGAGGTCCAGGATGGCACTCATCTGCTCGCAGGTAATGTTTAATGTTTTAACGGTTGCCAGTTCGATGCGAATAAGCATGCGCATCAGAGAGGCAGCCCGTTTAAGATAAACGGGTATGGGCGTTTGTAGTCGTTCCTGAATGTTGTTAATAAACACGTATGGAAGATTTTTGCAAAACGTTTTGACCATCGCGTATTTTTGTAGAATACTTTTTTCGTCGAAGGGAAGCACGCCACTGGTGGAGCTCAGTAGAATGTTTTTTACGATGCTGGCCACGTTTACCGGCACCTGTCTAACATCTGTAAGCAGCTGACTGAAATTAAAATTTTCGACGTTTAGGAAGATCTGTCGATATTTATCTCTATCCTTTTTAAGGCGTGAAAATTCTTCTTCAAACAAGGGCGATTGTATCCCGGTGTACTTGAATTTGTCTTCAAGTTCCTGGTCCGACAGCATGATGGTTTCAAACCGTACGGTTTCAAGCTGGCGCGCATCAAGGCCGTCCTCTCCGTACAACTGCTGCACAAGACGCGTATCGATGGAAACCCGTCGGTAATAATCCACAATACAGGATTGAAGGCCAAAGATGGCTTTACGGTTGGCATAGCCTGTGGATGATGTCGATAATGCTTTGTTGATCAAGTCGAATCTTCCATTCATTTCCCCAAAGATAAATTCAGGGGAGGTAAGGCCCGCAATATAGCTGTTGCAGATGAACCCGTAGGCCTGCGCCTCCAGGGCAAACCTGGGGTAGTACACCAGGGTCCTACCGAAGGAAAACTGGGGTTGAATGCGTTGTGTATTAATTTCAATTTGGCCGATGCCCGCCATGATGTGAATCATATTGGGGTTTGAGCCCTTGGCGCCAGTGGCCACCATCTGAAAAAGCCCATTGGTTTCCGGATTAATGGAATTCATAATCGGCTTTAAAATTCTATCGGGAAATTTAAGCGCATTCAGCTGCAATTTTTCGTAGAAGTCATGCGTTGTCAGGCCTATAGGCGGCATGATGTCTCCATGAAGCAGCCGGTTGTTTATTTCCTCCGACTCAAGCAGCAGTTCATTGATAATTTCTTGGACCTCCTGATGTGCCTCCGGGGTTAGGAGCATGTCGGCCGTGGACACTGTGAATCCGGCGTTGCGCACGTAGTTTAGGGCGAGCTGCTGGGTCGCAAATATCATTTTCAAGGCCTGCTGCGGCCCATACCTACGCGAAATAAGGTGATAGATTCCACCGGAGGAACCCGCTCCGACGGCCTTTTTGTCAAGGACGCCTTCAATGAGTTCGCCGTTGCGTATTTGTGTAGAGATGTCCTGCTTGTTATAATGCATGTAGGGTGCATACACTTCTGAGTACCATGTGGGGGCTCGTTGATAATTGATGGGGGTCTGCCTCAGTAGCATAGATACAACCGATTTGCCATCCAGCAGGTCAGTTGGGGAGTAGTTGGCAAAACAAGGTGGGTCGGTTTGGGTTGTTTGAAACAACCCCATGGCGTGCAGCTTGTTCATCACATTTTTCCCCATGGGGGTGTTCGTGCGTGTAAGCAAAAAGCTTCCCACCGTGGAGTCCTGCACCTGCCCATTAACGGGACCCGAGCTCTTTGTGGAAATGAACCAGTTTCGCACAGAACAAAGTAGTTCGGCCTCAACGCGGCTCATGACGCTCCAGGGAACCCAGAGATTCATCTGATCCCCGTCAAAGTCCGCATTATACCAGGCACATGCGCTGACATTCATTTGAAACGTAGAAATTTTTGGGTTTTCAAAAACGACAATCCGGTGAACCCCTATGCTGCTTCGTTCGAGAGAAGGCTGGCGATTAAAAAACGCGACGTCGCCAGTGACGACGTCACGGTAAAGGATGTCTCCTACCTCCAGCCTAAAGTCTTGTTTGAGACCCTCAATGTCGTGAACGGATTGTGTTATTTGCTTATACACTCTTGAACAACCAGGGTACTGGCGCTTTCCATTTAAAAAATAGGGCATTAATCTATTAATATTATAATGTTGCACTGTTTCCGCAACTTGCAGCGTTCGTGCAAAGGAAATGGGATAGCCAACCTCGTCCAGGTGAAGGTCTGAGTTCCCGCAGATGGTGGACCGGCTGATCGACCATACCTGGCTGCCCAGTAGGGATTTACGAATTCTTCCCTCCTTGCGAGGAAGTCTTCGCATGATGGAGGGAGCAGGGCGTGCCCCCATGACGATCCCACGCTTTCCCGTGCCTCCCTGGGTTGCGGTGGTGGAAACGGAATCCAACAAAAAGTTATAGTAAAGTTGCTGTATGGTTTGCAAATTGCGGTCAATATTTAAAGGTATTTTTTGGCCGCGCACGATTTGTAGGTCCTTCGGGATCAGCAGATTCTTTCGAACCAGATACTGAATCACGTTGTTAATGTCGTGAAAGCTTTGGGGGCCTGACCCGATTCCCAATCTGATGCCAGGTCGTATGCTGATGGGGGGGATCTGAATGGCCTTAAGCACAAGTTTTTCGGGATGGGAGTTTTTACTTCGCCCCAGTTTTACAACGGTGTCGTAGGTTACGCGCGAAAAAATCTCTCTGATGATCTGCGGGTACAGTTTGTCAATCTTGCCCTGCTGATCCGCCCAAAAGGTAAAATAATCTTCCGAGTCCTTAACAATTTTGGGGTGTACTGCCTTACAGACGTAGCACTGCTTTCCTTCGGTTTGGCTTGAAGCCGCTTCAATAAGACGCTTAGGCCTAATAAGATGCTCGTACCTCTTTAGGTCAACAATGGGAGCCCCGCAGTTGAGGCATATAACCCTTAACCATCGTCGTATTTCGGCGATGAAGAGCGGCTGAAGCACCGGAGCATGCATCTGCAGTATCCCAGGGTGTCCCATACATTGCTTGCGCTGGTGTGAGCAAGTGATGCATTTATAATGGTGATCGGTGGTTCCCATTCGCGCATCATAGATACCCCCCTCGGCGGGAAGGGTGCCCTCAAATAAATTAGAAATGGTAACCTCCATAACGCCTTGCCTCTTATGGTCATTGTCACCGGCAATATTGAACTGAACGGCGGCTATTTCGGCATATCCAGCCTCCATATTTTTGCTAAATACATAATAAAACTTCAAATGTTAAAAAAAAAATAACATCGGTTGGCATATTTTTTGTTAAAATCAAATGTTAAATGATTTCTAAAACATTTATCGGTTCACGAAAACCTACCGCACGGGCCTGAAGAGGAATGCCAGTTTTGGGGGAAAGCTCGGCATATTCCACGGTAAGCTCTTTTCCATAAAGATGTTTTTTAAATAAGGCGGGCGTGAGTTTTTGAAAAAGAGCATAACGATCCGCGTACGTCAAATGCTTAGGAGTCACTACAAACCGCTTTTTGTTTGGCAGTTCACAAACCCATAAAATGGCGCCTAAGTCCTTTCCCTTTTTTCCCTGAGTATAGTCCACCAAAATAAATTCAGCGTCTAGCAGCGGTTTCAGCTTGGCAAGATGCGCTGAGTGGTAGTTGTTGTATCCCGGCTCATAGGGCCCATTGGCATTGCGTACGATGGCTCCCTCGTAGCCCTCCTTAATAAACTGCGCCTTAAGCCTAAGGGCCTCATCCACATTCTTCACGCTAAAATTTTCAACTTGGTGGATAAAGGTAAGATCTTCCTTCTGTTTAAAAATATTTGTTAACAGCTGCTGTCTCTTGTTGGAAGGCATTTGAAGCTGATCACTCCAAAAGCAGTCAAACACGTAAAAGTGCAGCTCGGAGGAATCTGTCTTCGCATTCGCCTGCCCCGCGATCCATTGCAGAGGTTTGCGGTGTAAATAAAGCTCGCCATCCAAATATACTCTCACGTCTATAAATAAATAAAGCTGTTTGAGCTCTTTTTTAATATTGTCAAGACCTAAAAATTCCTTTTCCGTGCGCGAATACAAGAGAATGCAGCCATCGCCCTGCTGGCAGGCCACAGCTCGAACGCCATTGCGCTTGCGCTGCACGATGGGATCTGTTTCTTCTTCAAAAAATGTCTTAGGAATTATATTAAAATATTTTACCAGCATAGGGGGGATCATTCCTCTATTTGTGTGGGCTCCCCGCTTTTGTCTGGCATGGCGATTATATTTACTAAGGGCATCCTTGAATGCCTGGTGGACTACCGTTGTGGCATTTTTTTTACCCAAGTTTTTTCCCTCGGTAACACGTGTCATTTTTGATATCCGCACCGCCCCTTCTTCCACAAAAAATTTTGTGAAAATTTCAGCAACGGCGTCTTTTACATCTGTGGAAAACATCTCATCTGTGATGGGAATGATTGTGTTGTGCTGCACCACTTGCACACAAATAATCCATGAGGCCTTTTTTCCGCTTTTCGTTTCAGACTCAATCGGAGGAAAACAAAAAATGTTGTTTGAATATTGCCCAGGAAATTGATTTAGCATGGTTTTAACAATAAAATAAGCCTATCAATTTTTTTATAATTTGAATAGTTATTCCAAATTCAATATGGCTTCTTTAGATAATTTAGTGGCACGATATCAGAGGTGCTTTAATGACCAGTCTCTTAAAAATAGTACTATTGAACTTGAAATACGTTTTCAACAGATAAATTTTTTATTATTCAAAACCGTATATGAGGCACTTGTGGCACAAGAGATCCCTAGCACCATCTCCCACAGCATCCGCTGCATCAAAAAAGTTCACCATGAAAACCACTGCCGGGAAAAAATTTTGCCGTCGGAAAATCTTTACTTCAAAAAACAGCCTCTCATGTTTTTTAAGTTTTCAGAGCCTGCATCTCTGGGCTGTAAGGTCTCGCTGGCCATCGAGCAGCCCATTCGTAAATTTATCTTGGACTCCTCCGTTCTCGTTCGGCTCAAAAATCGTACGACCTTTCGGGTATCTGAACTTTGGAAAATAGAGCTTACCATTGTAAAGCAGCTGATGGGAAGCGAGGTCTCTGCAAAACTTGCCGCTTTCAAAACGCTTCTGTTTGACACCCCAGAGCAACAAACGACAAAAAATATGATGACGTTAATAAACCCAGATGGCGAATATCTTTACGAAATAGAAATAGAGTATACAGGAAAGCCCGAATCCCTAACGGCGGCAGATGTTATAAAAATTAAAAACACGGTGTTGACACTTATTTCTCCGAACCATTTAATGCTAACAGCCTACCACCAGGCCATTGAATTCATTGCCTCCCATATACTGTCCTCAGAAATCCTTCTTGCTCGTATTAAGAGCGGGAAGTGGGGGCTTAAACGCCTCCTCCCCCGGGTGAAATCCATGACCAAAGCGGATTACATGAAATTTTATCCGCCCGTTGGCTACTATGTAACGGACAAAGCAGATGGAATTAGAGGCATCGCCGTCATTCAGGACACGCAAATTTATGTGGTTGCAGACCAGTTATACAGCCTAGGTACCACCGGCATTGAACCCCTTAAACCAACCATTTTGGACGGTGAATTTATGCCTGAAAAAAAAGAATTTTATGGGTTTGACGTCATCATGTATGAGGGCAATCTATTGACGCAACAGGGGTTTGAAACAAGAATTGAGTCTTTAAGCAAGGGCATTAAAGTCTTACAAGCGTTTAACATAAAAGCAGAAATGAAGCCCTTTATTTCGCTAACAAGTGCAGATCCCAACGTGCTCCTCAAAAACTTTGAAAGCATTTTTAAGAAAAAAACTCGCCCATATTCTATTGATGGCATCATTTTAGTAGAACCTGGCAATTCTTATCTAAATACAAACACCTTTAAGTGGAAGCCCACCTGGGATAACACATTAGACTTTTTGGTGCGAAAATGTCCGGAGAGTTTAAACGTACCAGAGTACGCGCCCAAAAAAGGGTTTTCCCTGCATCTACTATTTGTAGGCATCTCCGGAGAGCTTTTTAAAAAATTAGCGCTAAATTGGTGTCCAGGATATACGAAACTATTCCCCGTTACACAGCGCAACCAAAACTACTTTCCAGTACAGTTCCAGCCATCGGATTTTCCATTGGCATTTCTTTATTACCACCCAGATACCTCGTCATTTTCTAATATAGATGGAAAGGTCCTTGAAATGCGTTGTCTTAAGAGAGAAATCAATCACGTCAGCTGGGAAATTGTAAAAATCCGGGAGGATAGGCAGCAGGATCTTAAAACCGGCGGGTATTTTGGCAATGATTTCAAAACAGCCGAACTCACATGGCTTAACTATATGGATCCCTTTTCCTTTGAGGAGCTGGCAAAGGGCCCTTCTGGAATGTACTTCGCCGGTGCCAAAACCGGCATATACCGCGCTCAAACAGCACTTATTTCCTTTATTAAACAAGAAATCATCCAAAAAATAAGTCACCAATCCTGGGTTATCGATCTTGGAATAGGAAAAGGGCAGGACCTAGGACGTTACCTGGACGCAGGGATAAGGCATCTTGTTGGGATCGATAAGGATCAAACCGCGCTTGCGGAGCTTGTTTATCGAAAATTTTCGCATGCTACGACCCGACAGCACAAGCACGCTACCAACATTTACGTGTTGCATCAAGACCTCGCAGAGCCTGCGAAAGAAATCAGCGAAAAGGTACACCAAATTTACGGGTTTCCCAAGGAGGGAGCTTCTTCCATTGTTAGCAACCTGTTTATTCACTATCTTATGAAAAACACGCAGCAGGTGGAAAACCTGGCCGTTCTGTGCCATAAGCTTCTTCAGCCGGGGGGAATGGTGTGGTTTACCACCATGTTGGGAGAACAGGTCTTAGAATTACTTCATGAAAATAGAATAGAGCTCAATGAAGTATGGGAGGCTCGTGAAAACGAAGTGGTCAAATTTGCTATTAAACGTCTCTTTAAAGAGGATATATTACAGGAAACTGGGCAAGAAATTGGAGTCCTGTTACCCTTCAGCAATGGCGACTTCTACAATGAATATCTTGTGAACACAGCGTTTTTAATTAAAATATTTAAACATCACGGCTTTTCCCTAGTTCAAAAGCAGTCCTTTAAGGACTGGATTCCAGAATTTCAAAACTTTAGTAAAAGTTTGTATAAAATTCTTACAGAAGCCGATAAAACTTGGACAAGCCTTTTTGGGTTTATTTGTCTGCGCAAAAATTAAATATTTTTTCATAAGAAGTACTACCCAGGTTTTAAAGAAATAGCTAAAAATATCATATGGATACTGCCATGCAGCTTAAAACGTCTATTGGTTTAATTACATGTCGTATGAACACCCAAAATAACCAAATAGAAACTATTCTGGTTCAAAAACGTTACAGCCTTGCTTTTTCAGAATTTATTCATTGTCATTACTCTATAAATGCTAATCAAGGTCATCTGATTAAAATGTTTAATAACATGACAATTAATGAACGACTGCTTGTCAAAACACTGGATTTTGACCGCATGTGGTATCATATTTGGATTGAAACTCCAGTCTACGAACTATACCACAAAAAATACCAAAAATTTAGGAAAAATTGGCTTCTCCCGGATAATGGGAAAAAGCTTATTTCATTAATCAACCAAGCAAAGGGCTCAGGAACACTTCTATGGGAAATCCCTAAGGGTAAGCCGAAGGAAGACGAGTCGGACCTTACCTGTGCCATACGGGAGTTTGAAGAAGAAACCGGGATTACCCGCGAATATTACCAGATTCTCCCAGAGTTTAAAAAATCTATGTCATACTTTGACGGTAAAACAGAATATAAGCATATCTACTTCCTTGCAATGTTATGTAAGTCGTTGGAGGAACCCAATATGAATCTTTCTTTACAATACGAAAACCGAATTGCCGAAATTTCTAAAATTTCTTGGCAAAATATGGAGGCTGTACGTTTTATTAGCAAACGCCAGTCATTAAACCTGGAGCCTATCATCGGGCCTGCATTTAATTTTATTAAAAACTATTTACGATACAAGCACTAGGATGCCGCATTAAAATGCCACATAAGGTAATACACTAGGAATGTCGCACACGCACAAGAATACAACGTCGCCGGAGATTTATTATCTAGTACACGTTTTATGTATGTACAATCCGCCTTCATTTAATATATTGAGCGGATGTACTATGTATTTATTTTAACAAAAAACATTATTTTTTTTTAATCTTCATCATCTGTTTTTATAAACTCAGTAATATCAAAAGTAGCTTGTGGGGTTTCAGAGGGTTCACCTTGGTTATCCTCCGTGAGGATAACATGTTCTTCAGGTTCGTCGTCACTGGAGAACCCATCATTTAATTCCTCTTCACTCAACATCTGTAAAAAATCTTCCAAGCTTTCGCTATCGTTAAAATCCTCATCATCCATAAGAATAATGGTACCTTCCTCATCGTTTCCTCCTTGTTTCGTGTCTAAATAGGCCTGCATGGCATTTGCAAAAGTATCAAAATAGGCTGAGTCAGATTGCTGTTCCAAAATATGGCCTTGCGTATTAAATGTGGTTGCATCGTTGTTAAATGCTTGCAAATACAGTAAGGGATTTATATCCATTATTATTAAGCAAAAAAAATTTAAATTATTTTTCGACCGATGTTAGGTAAAATTAAACAATTGCTATAGGTGTTAAGCAATGTTTATTGATTTTAAGTACTCAACAACCATGATGTAAATACTATACAGCACTTTTGGATTTTTAATCAAATCCAGATTAATACTAACTTCTTTTGTGATACAGTTCGTAATAATAGTATCCTGCTCATCGTTTTGTAAGATTTCTTTTAATATATTTTTTTTTACCGGGATACTAAGCAATTGATTATTTTCTTTTAAAAACTCCTTTTGATATTCAATCGTCTTATTCATTGAATATTTGTATATAACTATAATTACAAATGTTCAATGAATTGTTATTCATGTCGGGAGATGGCTATTTAAAAATCATGTCCTATTTTTCTTTGCTCAATAAGCATCCAAATATTTTCATGGCGTTTTATTAATTGTTCATTATTGAACGTATCACAAAGATCATTTATAAATTGCAGATAGTTTATTATTTCTTTCAAGAGAGTAACAAACATTACTTCAGCAGAACATATAATAGGTAATTCAGTGGCGTTAAAAGAATTTTGATCTTGTTGATACGCCAATGGCGAGGACTTAAGGAGATTTGGGGGTCTTGCCCAAAACCCTAGGCTGCTGTTCTTGTTTTTTAGGGCGTCATAAAGAAATGAAAGCACATTGCAAGGCTTAAGCCGCGACATCTCCTTCCCCTTGGGCCCTTTCCATATTTTTAGATCTAAGATCTCATCCGAGCTTATAGAGTAGGTATAGTAAAGTTTTTCAAAAAAGCATATCTGCTTGAAGTCTTTTTTAGAACGACTTTCAAGAAGCATTTCTATAATGTTAACAAGTTTTGTTAGGTTTAAGGCCTGTTCCTGTGTAAGCTCCTCTTGCACGTGATAGACTGAAAAAGTGTGCTTAGGAATGAAAATACTCCCCGTGGCACTGGCCTGTTGTCTGCCAGGTATATAGTACACGCTGCTGTTAGCAAGCTGTACCGGCACAATTTGCCCCACTTCTGCAACATTATTTTGCGATTCGGACGAGGGTATGACAATAGTTACGGGTTCAGTCAATAGGCTTTCGCCGAGAATAATATTACTGTCATTTTTAATAATTTTAACGGCCGCTATTAAATCAAAGGCATTTAAGTAAGAAACAACAGCAGAAAATCTTACATGCATATATCCTCTTCCGCTATTATTCGTACGCATAATAAAACAAGGGGAGCGTTGTATAACGCCAGTAATATTAAGAATAAAACTGTTTTTGAAACACTTACCCACATAAATGTTTTCAAGCTCCTTCAAAAGATGAGCCTCCACATTTGTACAAAAATTGGTAGGATCATCAATATTCAACGTTGTCTCAAAAATTTTTTGGTCGATCATATCTATAATATATTCTGTCTATTTCAATTTAAATAATATACGAATAAATAACGAGATTATTTTATTAAATAAGCAATGGTGTATACACTTTGTATTTACTTTGAGATATACTTTGTGTATCACAACGTGCCCTAAGATGTGTGCACAAGTGACGGCATTTTGTCGTTAAAAAGGTAAAACCAGCGGATTCCATCCTGCATTCCATTTGGTTGATTACGAGCCTCCATTTCTTTTTGCAAAAGGTTATTGCGAATGAGTAAGCAGAGCTTGATGGCACTAATCTTTGTAAGGTTTAAACTTATGCCCAATTGGTCAGCAATTTTTTGTTGCTCCTCCCGTCCGCGTGTTTCGCATACGGCTCCCCGGTTTAGCATGCGAATATCAGTAATCTCATTCTTTTTTAAAACCTGGATAGGTGGGCGGATTTTAAATTTAAGGGCCTTTCCCTTGCTTTCCATATAGCCTATGACGATGTCGTTTTCTTTTCGTTTAACATTAATATTAAGCATATAAAGCGGAATTTCATGCCAGGTTTTATCTTCTCGCGAGGTAATAAGTCGCACGGAGTCCTCCGTGGCATAGCCCACTAGAGTGTTGTCATCCCCAGGCACGTGGCTTATAATTTTAAAAATGTCCGGAAATGGCTGAATATCTTTTTTTGAAAAAGCGATGAAAAACTTTTTATAAACCTCGACAAGGGCCCCCATACCTGCAAGATTATCTATAATAAGTGCTTCTAGCATCGTATAGTGAAATGAAGCGGGATAATGGATGAGTACCTGCTCCATTGGCTCATCCTGAAAATCCTTCTGAAACTTTTCATACAATACTTGAAAGGGTTCTTTGGTCTGCGAGTGTTCGAGGTATTTGGTAATACGGATGCTGTGCATCGCGGGAAGCTGAAAATCCCGAATATATGTTTCAATATCTAATACCGGTTCCTTTTTATGGTTAAGCACCGCAGCGACGTACAAATGCTCAGGCTTTGCCGGCACATGCATAATGGTGCAAAGACGATTCTGTATCCATAATTCCTTGCACTGGTTTTTTGAGTAGCATAGAGAAATGAGCGCCAGCGCGAAGTTGTCCTCTGAGAAGAGTTTATTATCGATGGTAATTCCCTGTATGAGCTTGGGAGTGGAAACAGCCTTCCATAGCTCGGAGTACGTCCACACGGGGCGTGCCATAAACAAAGATATAATAATATTAGAAATTGTTTTTACCTCTTGCTCCCCGTATCCATAGGCCTCAAAGGTATTGAGGACGGTGGCTCCGACGTTTGCCGGCGTGATGGATGGACTAAGGGGCAGACTTTCCAACATAGGCTTATCAATCTTAATCTGGTTGGTGAACCCATCAATGGCGTGCTTTCGCAGCGCCTTATCCCCCTCCTGTATTAAAATGTATTCTTTTAATTTTTGTGCGTACTTAGCGAGCTCTGGCCCTCCATCGGGTGTTGTCGATACGTACAAATAAATTGTCACGTTGCGCTCACTGGGGGGGAGCTCCATGTGTGAATTTTTTCGCACCACCCTCCCAAATACCTGAATAAGCCGGGGAATATCAAGGGGCAATGACATAATCATCTCGTACCGCACGGCCTGAAAGTTCAAACCCTCCACAATCACCTTGGACCCGATGAGAATACGCAGCTGGTGGCCTTCCAGGTTGGACGAGGCGTTAAAAAGAGCCAGGCTTCGTTCGCGTACAGCGGGCTCTATTTCGCTGTGCAGAATGGTGAACCGTACTGGAATAAACTGATGGTCGCTATGTGTGTGCTCATCGCGAATCGCGGCGCAGATGGAGCAGCGGGTCGTTCCCACAGGGGACGAAACTTCATTTAAAATGCCATTACTTTGTAAAATTTCTTGCAAGATAAGAACCCCCGACATGCGGACCCGATTGTGGTAAATTAAAATTTTCCCCCGGCCTTGCCGAATAATGGAAAGAATGTCTTTCATCATTTGAGTGTATTTTCCGCTATAAAAGGCCAATCCCGAGATGTGCGTTGGTGGCTGCAGCGACAAAAAGCTGCCACTCACATTAAAGGGGGCTCTACGCGAAGGCTCAATAATCTGTACCCCGTTTTCCAGAAGCCAGTCTGTGCTTGCCATAGAAAGGGCGGTGGGGGTTTCCGTCGAGTTAAACAGGCCGTAAGCCTTGGGTTCCGTTTGTTTTGAAAATTTTGGGTTGGGAAACACCATGTCATAAATGCTGTACGCATTACTCGAGATTTTAGGGTCAGGGCCCAGCTGTTTAAGCGTTTCAAGCTGATACTCAGACATGGGGCATTCGATGAAATGTAAGTACGGCAATGTTTCGTCTTTATAGGACAACATCTTTCCGGCAAATATTCTTTCGGGGTAAAAATTGGTGTTGGTATCCAACAAAAAAGATACCCTTCCGGTGCTCAGTCTTTCCACAAGAGCTAGGGCGTCCTTTTTCCATTTAACGGAATGCCCACTGCTGTCAAACAGTTGCTGGCGCTGGAGGGGCTGGCCGTTGGGCAGCTCATGCCGCGGAACCAAAAGGTTTAACAGGTCGACGTATTCCATGACACTCCCGGTTACGGGCGTTGCCGACATGAAGACGGCCCTGGGGGCCTGGTGAGGTGGAAAGGCATCCAGGACATACTGTAAAGCGATGCCATAATTATTTCGTTCCTGGATATTGTACACGTTGTGTATTTCATCCGCAATGAGCAGTCCTCCCCTAAGTTGCTCCATGATTTTTTGATTCACCCGGATGAGGCCGTTTGTCTCGGCCTCGCTAATTTTTTGCACGAACTGAGATATATCGTTCTCATTCAATGTATCTTCTGCTTCGTCAGAACGATGAAACAGAGAAAGCACATCAAAGTTTTTCTCTTCACCCTTACTCGTAATATTGAAAAGCTTGGATGCAAATTCCTTATAGCCGTAAAACTGAAAAAAGCCTCCGCGGTTTCTATCGGTTAAACGGCGCTTTAACGTACTAACGAACCCATTTAGATGCCGTGATTCGACCGACGTGGTGCTGCCAGACTGCTTTGCAATGTGAAGAAGCCGGTGTAGCTCAGCGACCTCCTTGTAAGAAACAAATCCCAGCTCAGGACGTCTTAGCATTTCTGTTTGAATGATGGCGCGTGTAAAGCCTACCACAAAAATCCAGGGCGCATTTTCAATAAAATTCATGTAGTGGTTCATAAATTGACGCGCGATGGCAATCGCGGCAATGCTTTTTCCCGTCCCGGTCTGCCAGTTTAATAAAAGACGCGAGTAGGGCGTGTTGGGATTTTGAAAGTTTTGGACGAAAAGCTGGGCATTATGCAATTGGAGACCCTTGATGGAAGGAAAGGGCGACGCGTAGGGGTCACACGGAAAAAACGCTCGCCCCCCCTTCTCGCAGCCAGGCCCACCGATCTGGACAAAATGAGCCCGCAGATCACGAATGAGCTCTTTTTGGTCGACAGGAGGGGAAATCAACGATTTAAACTCCTTTCTTCGCGCCAACTGCTGCAAAAAGTCTGCGGCATCCAATTCGGGATACGCCATATTATCATAAAAAAAATAAACCTTTTTATGAAAACTTTTATGTGATTCTGTATTGCAATTGTTTTTTATGAATACTGTAAATAAGCGTATCAACTTGTTTTTCTAACGAAGAGGCGTTATTCTTTTTTTCTGGATATAAAATAATAATAAGTATAATAATTAAGACTAAACAGCAGGCAATCACTATCAAACTCATATTATACTTACTTTTTTATAAAAAGTATTATATCTTATGAATGCGCAAGTTCAGCTAATTGTTCGTCGCTTGGAATGTGGGACTGCAGGGAGGTGGAGTTTTTCCTTTTTCTAAAGAATACCGGGAAATGGTGGTGAGGCTCAGGTTGTTGTACATAGTAGCTAGGAGGAGGTTTAGGTATGCTCGACTTGCAGTCAATAGTCCGGTTATAGTAAACGATGGCAACGATGATAAGAATAATAATGAGCAAAATCAAAATGCCCAGGAGAATCGCAGTTGTTCCGGGATATTTGGCGATTGTATGGGCTAAAAGGCCTTGGGTGCTTTGTTTAATTCCCTCGCGGGTTGACAGGTTATGAGAAAGCAGTGGAGACGTTTCAGTGTCCATTTATTACAATTGAACAGTTATATTAATCTCAAATAAAATATAACACAAAATTAATTATGGCCATGCAAAAGTTATTTACGTATATTTACGAGTTTATTGAATATCGTAAGATGGTGCTGTTGGAAGAAAAGGTACCATATGATAAGTTTGTTCAAATGGTACTTAATACAGGATTTTTTCGTATTAACGCGGAGACGCTGAATCACGGAATCGTATCCGTGTTTATCTTTGGAGCAAATGGCAAGTACGTTCACCACGGAGGCGACATGAGAACGCTTTTAACGAATACGCTTAATGAAAAAAAACATTATGAAGAATTAATTTTAATCGTTGATAAGCCCGTTTTAAGCAAAAAAAATATTTTAGATATAATCGTCGAGCAGCGCGCTGCAAATCCCACGATTGTAATAAACATATATCCCTACCACCTGTTCTGCATTAACATTCCCAAGGTGAGTGCCATTCCTAAACATAAACTAATTACTCAGGAGGAGGCGCAGGAGTTTTTAGGTCGCGAATATCTGCAACCGCAGGACCTCATGCAAATTAGCGCGTCAGACCCCCCGGTGGTCTGGCTGGGAGGAAGACCGGGAGACTTTGTGCAAATTGAGCGGCCCTCAGAGACAGCTATGCACGCTGTTGTTATCCGCTTTATCACCAAGTCCAAAATTTGAGTCCCGTGTTTAAAGATGACAGACAGCTAAGTAAGCATATCTGTAAAATTGTCGATGTCCTCTGTGGATAGAGCGCTTTCCTCTGAGCAGCAGATTTTTTCATACGTCTCCATGGGAGATGGCGAGGCTTTAATAGTATGTAGGTCACGTAAGAACTGTTGTATGATGGGATATTTGTCTTTTAAAAACTGGGGATGTTTCATAACTGGAATTATTTGAAAGATAAAGACCTTCCATCCAAAGTAGCCAACCACATTTGGCATTTCGGGACACGCGGTTTCATAAGGCATAGAATAGTGAATAGTGTACTGATCTTTTTGATACAGCGTTTCAAGTAGTTGGCGAAATGTTTCCGCGTCGAGCGTGCCAAAATCTTGAGGAGCCTCGGTGTGCTCCTGTGTAGAGCAGATCGTGATGATTCCCCAGGCAAGCGGGAGCATGGACTCTGGAGGGTGGATATCCGTATTGGTCTCATTATTCGATCCCAGCTGATGAATGCCGCACACGCGAAACATGGCCTCGACGTAGATGCCCATAGAGATAGGCGGCGAAAGGGCAAGACCGGATTGTATTTGCGGCATATAGTAGGAGGGCACCGAGTTTTTTATTTTTCGGTTGAATGGGGACTTTATTTCTACCAGCACGGGGATGCGTTTCGTGGCCTCATAGCGTACGTTGTTAAAAATTGTTTTGATTTCCCAGGACTGTTGAGTGTATCCCAGCGTTAGGTGACAAAACCCATCGGGGCTATTACTATGTCCGGGGTATCCCAAATAGGTCCCATCAATATGAATATTGTCACCTATGACGGTGGTTTGGCAGAACAACTCAAGCAGATCTTTACTAACTCGCTCAAAAAGGGTTCCCCAGCTACAAGCAGCGCGGTTCAAATTCTTCTTAAAAAGATTTGCTTTTTCCGCCAAGATTATATAATAGCTTTTGTAAGGGTTTAAACCTAAAACGCTAGCAAGGTCAGAGCCACCCACCTGAGTGCGACGAATAGCATGCCAGGCATCGGAGCGCTGCTGAGGAGAGTCTTTAAACAGGCGTACAAAGGTTTCCATTATACTTGTTTTAACAGGAATTCAATATAAAAAGTCAACACAGTTTGCAATTTTTCCAATCTCAAGATATAGCCATACATTTTTTTTTCCAATTGGCGAATATGTTTAAGCTCATGTGTTTCAATATTAGCATCCGGAAATTTAAATGCATAAAGATGTTCAAAGGCCTGATTTATACACGTATCAAAGGATCTGTGGTATGTTATTAGCTTCAGCATGTGTGCCAGATCTTCAAGATGGTCTAAATTTATACGGTTTTCCACGTGGTGGATCATGTCTGCCACATCTTGAGCCCCCATCCAGGGGATCACAAGGTACTCCCCCTTAAAGATGATTCGTCGTTTTTTTAAAAAATCATGAAAACGTTTTAAAGCTTCAAGAAAGGGGCAGTTGGGCTTTGACCCCAAAATGCTGACGACGATATCCTCGGGCATGATGTATTCGCAGTGAGGATAGTAGTTTACGGACTCTAATTCAGCGGCCCGCCGTTTTATTTCGTATCTTGCCCAGTTATTCAGAGAGTACTCCACGCCTCCGACCACAACAGACATCCTATCTATTAAAAAATAACAATAAAAACCTTATGAAATCTATGTATAGTGGCCGCTAAAATGTCTATATTAGAAAAAATTACGTCAAGTCCCTCTGAATGCGCAGAGCATCTTACAAACAAAGATAGCTGTTTAAGTAAAAAAATACAAAAAGAGCTCACCTCTTTTTTGGAAAAAAAAGAGACACTCGGTTGCGATTCGGAGTCCTGCGTAATTACCCACCCCGCCGTGAAGGCCTATGCGCAACAAAAGGGACTGGACCTCTCCAAAGAACTGGAGACTCGGTTTAAAGCGCCAGGACCCAGAAACAACACGGGTCTTCTTACAAACTTCAATATTGATGAAACGCTGCAGAGGTGGGCCATAAAATACACCAAGTTTTTCAACTGTCCTTTTTCCATGATGGACTTTGAGAGGGTCCATTATAAATTTAATCAAGTGGATATGGTAAAGGTATATAAGGGAGAAGAGCTACAATATGTAGAAGGCAAAGTGGTCAAGCGTCCTTGTAACACCTTCGGATGCGTTTTAAACACGGACTTTTCAACGGGCACTGGAAAACACTGGGTAGCCATCTTTGTGGATATGCGGGGCGACTGCTGGAGCATCGAATATTTTAATTCGACGGGAAATTCTCCTCCAGGTCCCGTTATTCGTTGGATGGAACGGGTCAAACAGCAGCTATTAAAAATACACCACACCGTGAAAACGCTTGCAGTTACCAACATTCGTCACCAACGGTCGCAGACCGAGTGCGGCCCCTACAGCCTGTTTTACATCAGGGCACGCCTCGACAACGTGTCATACGCCCATTTTATATCCGCTAGGATTACCGACGAAGACATGTATAAGTTTAGAACCCATCTGTTTCGCATCGCATAAACTAATAAAGTTTGAATTCTTTATAGGAATAAAAATGGAAGCGTTTGAAATCAGCGATTTCAAAGAGCATGCGAAGAAAAAAAGCATGTGGGCTGGCGCCCTCAACAAAGTCACTATTTCGGGTCTTATGGGGGTCTTTACCGAAGATGAGGACCTTATGGCGTTACCCATTCACAGAGACCACTGCCCCGCTTTGTTAAAAATTTTTGACGAGATCATCGTAAATGCCACGGATCATGAAAGAGCTTGCCATAACAAAACAAAAAAGGTAACTTACATTAAAATTTCGTTTGATAAAGGTGTGTTTTCTTGCGAAAACGATGGCCCGGGAATCCCCATTGCAAAGCATGAGCAAGCCAGTCTTATCGCCAAGCGCGATGTGTATGTTCCCGAGGTGGCTTCATGTCACTTTTTAGCCGGAACGAACATCAATAAGGCCAAGGACTGTATCAAGGGGGGAACCAACGGCGTCGGGCTGAAGCTCGCCATGGTGCATTCGCAGTGGGCCATTCTTACCACCGCCGACGGCGCGCAAAAGTATGTTCAACATATCAACCAACGCCTAGATATCATTGAGCCTCCTACCATTACACCCTCCAGGGAAATGTTTACACGTATCGAGCTCATGCCCGTATACCAGGAACTAGGGTACGCGGAGCCTCTGTCTGAAACAGAGCAGGCGGATCTTTCCGCCTGGATTTACCTTCGCGCCTGCCAATGCGCGGCCTACGTGGGAAAAGGCACCACCATTTATTACAATGATAAGCCTTGCCGCACGGGCTCTGTGATGGCGCTAGCCAAAATGTACACCCTGTTGAGCGCGCCTAATAGCACGATACATACGGCGACCATTAAGGCCGACGCAAAGCCCTATAGCCTGCACCCCCTGCAGGTTGCGGCGGTCGTGTCCCCCAAGTTTAAAAAATTTGAACACGTGTCCGTTATCAACGGGGTAAATTGCGTAAAAGGAGAACATGTCACCTTTTTGAAAAAGACTATTAATGAAATGGTCGTTAAAAAATTTCAACAAACGATTAAAGATAAAAACCGCAAAACAACATTACGAGACAGCTGTTCAAACATCTTTATCGTTATAGTGGGTTCCATTCCAGGAATAGAATGGACCGGCCAGCGGAAGGATGAACTTAGCATCGCGGAAAATGTTTTTAAAACGCATTACTCCATTCCTTCTAGTTTTTTAACAAGTATGACAAAGTCTATCGTGGATATTCTTCTGCAATCCATTTCTAAAAAAGATAACCATAAACAGGTCGACGTAGACAAATATACGCGTGCCCGCAATGCGGGAGGAAAAAGGGCGCAGGACTGCATGCTACTCGCGGCGGAAGGGGATAGCGCACTTTCCCTGCTGCGCACGGGACTAACCCTGGGAAAGTCCAACCCAAGCGGGCCCTCCTTTGACTTCTGCGGCATGATCTCCCTGGGAGGAGTCATCATGAATGCCTGCAAAAAGGTGACAAACATTACAACGGACTCTGGAGAAACCATTATGGTGCGCAACGAACAGCTTACCAATAATAAAGTGTTGCAGGGAATCGTGCAGGTATTGGGTCTAGACTTCAACTGCCATTACAAAACACAGGAAGAGCGAGCAAAGCTGAGATACGGCTGCATTGTTGCGTGCGTTGATCAAGATCTGGATGGGTGTGGAAAAATCCTTGGACTGCTGCTGGCCTACTTTCACCTGTTTTGGCCTCAGCTTATTATCCATGGTTTCGTAAAACGACTGCTTACCCCGCTGATACGTGTGTATGAAAAGGGTAAGACCATGCCCGTGGAATTTTACTATGAACAAGAGTTTGATGCCTGGGCAAAAAAGCAGACCAGCTTAGCCAACCATACCGTAAAATATTACAAGGGATTGGCGGCGCATGACACCCATGAAGTAAAAAGCATGTTCAAACATTTTGACAACATGGTGTACACGTTTACCCTGGATGACTCAGCAAAGGAGTTGTTTCATATTTATTTTGGCGGGGAGTCGGAGTTGCGAAAAAGAGAGCTTTGCACCGGCGTGGTGCCGCTCACCGAAACCCAGACGCAGTCCATTCATAGTGTCCGACGAATTCCTTGCAGCCTGCATCTGCAAGTAGATACCAAGGCTTACAAGCTGGATGCCATCGAGCGGCAGATTCCCAACTTCTTAGACGGGATGACGCGGGCGCGGCGCAAAATTTTAGCCGGGGGGGTGAAATGCTTCGCCTCCAACAACCGTGAACGAAAGGTTTTTCAGTTCGGGGGCTACGTTGCAGATCACATGTTTTATCACCATGGCGACATGTCGTTAAACACAAGTATTATAAAAGCCGCCCAGTATTACCCAGGCTCCTCCCACCTCTATCCGGTATTCATAGGCATAGGAAGTTTTGGCTCCAGGCACCTGGGAGGAAAGGATGCAGGATCCCCAAGATACATCAGTGTGCAGCTTGCGTCTGAATTTATTAAAACAATGTTCCCCGCGGAGGACTCATGGCTTCTCCCCTACGTCTTTGAGGACGGCCAGCGGGCGGAACCAGAGTACTACGTGCCTGTGTTGCCGCTTGCTATTATGGAGTACGGCGCCAACCCATCGGAGGGCTGGAAGTACACCACTTGGGCCCGGCAACTGGAAGACATTTTGGCCTTGGTGAGGGCCTACGTCGACAAAGACAACCCAAAACACGAGCTACTGCACTATGCAATAAAACATAAGATTACTATACTCCCGCTGCGGCCCTCCAATTACAATTTCAAGGGCCATTTGAAGCGGTTTGGCCAATACTACTACAGCTACGGCACGTACGTCATCTCAGAGCAGCGAAATATAATTACTATTACGGAGCTTCCTCTGCGTGTTCCTACGGTTGCATACATCGAAAGTATAAAAAAATCGAGTAACCGCATGACATTTATTGAAGAAATCATCGACTACAGTAGTTCAGAAACTATTGAAATTCTGGTGAAATTAAAGCCAAATAGTCTTAACCGTATCGTGGAAGAATTTAAGGAGACTGAAGAGCAAGATTCCATAGAAAATTTTCTGCGCCTGCGCAATTGTTTACATTCACATCTAAACTTTGTAAAACCTAAAGGTGGCATTATCGAGTTTAACACGTATTATGAAATTTTGTATGCGTGGCTACCTTACAGGCGTGAGCTTTACCAAAAGCGTCTTATGCGTGAGCACGCGGTGCTTAAGCTGCGCATTATCATGGAAACTGCTATTGTACGCTACATCAATGAGTCTGCAGAGCTAAATCTTTCCCATTATGAGGATGAAAAGGAGGCAAGCCGCATTCTAAGCGAGCATGGATTTCCCCCGCTGAACCACACGCTGATCATTTCCCCTGAGTTTGCCTCTATAGAGGAACTCAATCAAAAAGCACTGCAGGGCTGTTATACCTATATACTATCTTTGCAGGCTCGAGAATTGCTTATCGCAGCCAAAACTCGTCGGGTGGAAAAAATAAAAAAAATGCAAGCTCGTCTTGATAAGGTTGAGCAGCTTTTGCAAGAGTCTCCCTTTCCCGGCGCCAGCGTATGGCTGGAGGAAATTGATGCGGTGGAAAAGGCTATTATAAAAGGAAGAAATACTCAGTGGAAATTTCATTAAACGCTACCGGTTTTATGATGTCCAATAGGTGTTAAGCAATCAGTTCATCAACATTTTTTTCAAGAATTTGAAAAGTTTGGATAATGTTCTGAATACTTTTTTCTAAAAGAGTTATCAAATCTTCTTGTGAGGCCTTATGAATAATTGTTAATACCATTTCTTGCTTATGGGGAACACACTGATACCCCACAAAGCTAATATCAGGAATCATTTCATAAATATATGTTTTTAGCAGATTTCCGATGGTATGGGTTTCATCTTTTATCGTGATAATGGCCTTTGTTTTTTCCTCATCCATGGAAAACAGCACAAGTTCCGGCTGCGGCTCTTCAAAGTTTTCATAAATTTTTTGAATGCTTTGGATTCGGCCAATAATGATCCGGCAGGCGTTTTTTAAATACGTGCGAACGGCCTGGTTGATATGTGGCAGCGGCACCGCTGGAAAGCAAAGCCCCAGGCGGTGGTGACGCGGGTCTGAGGTCATAGAGCTTTGCTTGTAACCGCTAAGCGCCATATATTCTTTTTTATCCGTTGGGTACTGTTCAATGTCAAGGTGGGAAAAATGTGTTTTAACGGCAAGATTAAAGGCGGCATGCTTTCGTCCTATGCCCTTTTTAATATAGATATCCTCTATAATCAACGATTTTCCGGGTTGTAGGAAGCCAATCTCAAAGGTAGGATTAAAAATCGGGTATTTAAGCTTAGGGCCTGCCACCTGGATGAGATCGCGGCTATAGATGGTTTTAACCTCACAGCTATTGTTTAAACTCCGCAGAGCAAATACCAGTGTCTCGTTTTTCGCATAAATCGGAATGAAATTAATGCGGTTTCTAATAAATTGTTCCGTCATAAACAGGTCCGTGGAATCCTCGATCTTATACCCACCGGGCTTAATATCTAGCATATAATTGGGAATTTCATCTTGCAAGACCCGCGACAGGCCGTGGACCGCGGCTCTGCTAATGCCCTTAAAGTCCATAACAACATTGACCGGGACGAGGGGCAACTGCTCCTCGAGCTGAAATAGTTTTTTGGCCGCATTTTTAATAAAGAGGTTGGAAAAGTCTATCAAAAACGGTTTGATTTCCACGTTTTGGAAAATTTTTTCCATTTGTATTATAAATATATCTATATATATTCAAATTATGGTAGTTTATGACTTGCTCGTTTCTTTAAGTAAGGAATCCATAGATGTGCTACGGTTTGTAGAGGCAAACCTTGCGGCGTTTAACCAGCAGTATATTTTTTTCAATATCCAAAGAAAAAACTCGATCACGACACCCCTTCTCATTACGCCGCAGCAGGAAAAAATTTCGCAAATTGTTGAGTTTTTAATGGATGAATATAATAAGAACAATAGAAGGCCCTCCGGGCCGCCGCGTGAGCAGCCCATGCACCCATTATTGCCGTATCAACAATCCTCGGACGAACAGCCCATGATGCCGTATCAACAGCCCCCGGGGAATGATGATCAGCCATATGAGCAAATATACCATAAAAAACACGCGTCGCAGCAAGTAAATACTGAACTGAACGATTATTATCAACATATTCTTGCATTAGGCGATGAAGACAAAGGTATGGACAGCATGTTAAAACTTCCAGAAAAGGCAAAAAGGGATAGCGATGATGAGGACGACATGTTTTCTATAAAAAACTAACGACGTAACAATTAAACAAAAAATAAAAATCATTATAAAATGAATCTTGAATACGTCCAAGTTGTTCAAAAATTTAATCAAGTACTCCTAGAACTTACCAAAAAAGTATGTACCGTTGTGGGCGGGAGCAAACCCACCTATTGGTATCACCACATTAGAAGGGTTTGCTCAGAATGTCCATCCATGCCGATGAGTATGATAGGTCCGTATCTGAATGTCTATAAAGCCCAAATTCTAACAAGGGACAAGAATTTTTTTATGAATTTCGATCCCGCGCATAATGAGTACACCTTTATCATTCAAAAACTAAAAGAAGCAGCCCGAAATATGCCGGAAGACGAATTAGAACAGTACTGGGTAAAACTTTTATTTTTACTTAAAAGCTACATAAAATGTAAGCCCTTTATTAATTAAAGAATTGATGCATAACTAATAAATGGCCGGTCGTGTTAAAATAAAACAGAAAGAGCTCATAGACTCTACTGTAAAAAACAAAAATGTGATGAATCTGTTCCATGAAATTATAGGCTCAAAAGGCAATATTAATTTTAGCGTTGTCTGGCCCAAGTTTAAAAAAATCAAACAGAGCGTTTATGACTACATTTCCACTCTTTCTGTGCTGGAAAAAGCAAACGTTATGCAAAACTTTGAAGCTGATAAGAAACTGTTGGAACTTTTTGTACAAAAGCTGTGGGCTGCCTATGAAGGCTATTTCAAATATCCCGAGATTGAAAAATATGAGGTGGAAGGCCAGGTAAATTTCAATCTCGTACCTCAGTGCGTCCTCGAAAAGTTTAGCCAGTTGTATAGGATAAGAATCAATTCAGAGCTTGTCACACTCATCCTAAACAGCTGTGCCTTTATGAGTAAATATAACGATTATATTCTCAAAAAAGATCCCTACATACTAACCATAACCCCCGGCCTATGCTTTTCCCCCATTCCCAACTTCGAGGACCTAAATTTTAAACATCTTTACAACAGTGATAAAAATTCTCAGCATGACAAAGAGTTTATCATGTTTATATTATATAAGCTTTATACGGCTGCCCTAGGAGTGTACAATGCCATCTCGATTCCAGACATCGACGTAGAAGACCTTGAAAATATCATCCTATCCTCGGTGAGCCAGATTAAAAAACAAATTCCGCGCTGCAAAGACGCCTTCAACAAAATTGAATCTTCGGTACACCTGCTGCGCAAAAATTTTAACACATATTACAGTGACTATGTGGGCTCAGGCTACAACCCAACCATCATTATGGAACAGTACATTAAAGACATATCACAGGATTCCAAGAACATATCACCACGCATTTCCTACCAGTTTAGAACCATCATCAAGTATTACCGCGACATGATCGCCACCAGGCATCAAACGATGGACCCCCAGGTATTAAACCTCGTAAAGCACGTCGAAAAGAAATTAGATATGCTTGATAGAGAAAAAAATTAGTATATATAGTTATGGTGAATCTTTTTCCTGTTTTTACCTTAATTGTGATTATTACAATTTTAATTACGACTCGAGAACTATCCACCACGATGCTTATTGTTTCTCTTGTAACAGATTATATTATTATTAATACACAGTATACGGAACAGCAGCATGAAAACAATACATTTTCCATGCCGCAAAAAAATTCTTTTAACGAATCTTATAATAAAGACAAAAAATCTAATACACATATTCCCTACCAGTGGCTGGCGCCTGAACTAAAGGAAGCTGAGAGCAAGTACTGGTGGGGCAATTATGATCCTCATAGCGAGCCCGTTCTCGCTGGCGCATCTTGAATATCTTCATACGTGGCACGTCACCATCAAAAACATTGCCCAACAACACGGGCTTGATATAAAGGTGGCCATTGTGGTCTCAACATCGCATTTAAATAATTTTTTGCCAATTTCCGGGGCGCTTAACATCGAATGTATAACCTTTCCCAGTTGCGGCATCAAGGAGATAGACCTCCTATGGGCGCGCATTAAACTATTTCAACATTACTGCGCCATCGGTGCCCGTCTTTTATGGCTGGTAAGTGCTGACATCAGGCCCCCTGTTTCAGCGTGGCCAGCCATCGCTGACAGTCTAAAAAAGGGAGCAGATGCGGTGGTTATTCCCTATCCCTCCCGATGGAACAATCTCATACCTACCGTCATCAAAGAAATAGTTGTCCACCAAAAAAAATGCCTTGTGGCGGTGGATGCACGCCACCTTGATACAGATACCCAGATTGTGGGGGCCGGGATGGGCTGCATCGTCCTAACCCTAAAGGCCCTTATGGTGCGTCTAAGTATTGGCAAACAGCCCGTTAAGATACTGTGGCCCGACCTTCACGGCACTGCCGAGGGCATTCCTCTGGAGGGGGTGGAGGTTGGCTGGTTTTTAAACGCTTATGCGCATAAATTAAATATACGCTGCCTAGGGGCTGATCATATTGCGCAGCACTTAACTTAATTCTTTATTTAAAAAGCCCACGCATCCAGTAGCGGCCTACATTAAGGGCCTACGCACATAAATATACGCTGGCTAGAAGTATGCCTTCATTTAAACCATTGAATTATTTATATAATGGCTGTAAACATTATTGCAACAAGAGCCGCGCCAAAGATGGCCAGCAAAAAAGAGCATCAATACTGCCTGCTAGACTCCCAGGAAAAGCGTCATGGGCATTATCCCTTTTCATTTGAATTAAAGCCTTATGGGCAAACAGGCGCAAATATCATAGGAGTACAGGGCTCGCTTACCCATGTTATCAAAATGACAGTATTTCCATTTATGATTCCTTTTCCTTTACAAAAAACTCATATAGATGATTTTATTGGTGGACGCATTTATTTATTTTTTAAGGAACTGGACATGCAAGCAGTTTCTGATGTAAATGGAATGCAATACCACTTCGAGTTCAAGGTTGTTCCTGTAAGCCCCAACCAAGTAGAGCTTCTTCCTGTGAATAATAAATATAAATTTACATATGCTATACCGGTGGTGCAATACCTTACCCCAATCTTTTATGATCTTTCGGGACCGCTAGATTTCCCATTAGATACTCTTTCGGTCCATGTGGATAGCCTCTCCAATCATATACAGCTTCCTATCCAAAACCACAACCTAACAACGGGTGACCGTGTTTTTATTTCTGGATATAAACACCTGCAAACGATTGAATTATGTAAAAATAACAAGATTTTTATCAAATATATACCGCCGCTTTCATCCGAAAAAATAAAACTATATATACCAAAAAACCGAATCAGAATTCCACTATACTTTAAATCTTTAAAAAACGTCTAAGTAATAACATTTTTATAGTCTACTCCTAGTTCCGAAATAGGCTGAATTTCTTTTTTAAGTCCTTTAAACCAAGGATGTGATACAAGACTCTTAAAGGAAAGCCGCTTATTTTCATTAATTGTTAAACATTCCGTGATAAACTGTTTTCCCGTCTCTGAAATGTTCTCGGGAATATAATTTTCCCGTTTCAGAATATCATTTAAATAAAAATTTTCTGCACGAAATCTAAAAAGATTAACCGCGACCATACCTATCGTCCACACAGTTAAAGGAAGCTGGTAATAATAACCATAATAATAAAATTCTGGACACACGTATTCCCATGTTCCAAACATATTATATTGGGGACGGGTTTCGTCTAATCTAACAGCGCTTCCAAAGTCAATGACCTTAATGATCTTTTGATTTATGTCTATAATAAGGTTCTCATCCTTAATATCCCCATGGATAAAGCCCTTCTCATAAATGTTTTGTATAATAAGAATAAGCTGGAATATTATTTTTTTGGCTTCGGTTTCCTCAAGTTTTTTAAAGTAATGATAATGAAGTAGATCAACACTATTTGGAATATATTCTATAATTAGTATATGATACATAGCATTTTCGGTATATTCGATAAGCTTAATAACACCGGGAGTATCTTGCAGGGCTTTCAACACGATGACTTCATTTCCTGGAATTTCTTTTTTAGAAACGTACTTAAATATAATGGGTTGCCCTACTTGATGACCCAAAAAGACGTTATTTCTGCCACCCTCAAACATGGGTCTCGTCGCAATGAAATACATGTGCTGCGTTGTGGAGATCCTTTCCACCTTTGCTGTAGGATAAAACGCATATTGTGCCTGGGGGGTTTTTAACATTTTTTTAAGCTGTTGTTCCGGCCTGGACATGTTTTATTAGCTTTATATATAAAGGGTTAGAAGGTTTAATTTCAATATATGCCTTAATGATGGGATTATATTCGTAAAAGGTATAGCCTAATCCTACGTCTTTGTTTTTTTGGTAAAAAAACTGTTTGCCCTCGTAGGATATGCTATAGGCTTTTACTTCGGCTTTTACAAGCGGTTGGCAGGGATTGGGCAAACGTAAATCGCGTTCAAAGTTTTCATGAAAAAGCAAAGCATTTGTGGGCTGACACATCAGACAGCCGCTTTCGCCGTTGAAGGCACATTCAATGGCCGCCCTTTTTAGTAAATCGCGGAAAGCAGAATTAAGATGGCTCTTTTCAAGCCCCCTTTCGTGAAAACGCTCATCAATCGTTTTTTGTTCCTGACTGCCTTCGGGAATACTATAAAACATTTTTTGATTAGCCACCGCGATGTACAAAAAAGGCTGCACGGTTTTCTCCTCGGGCGGTAGCGCATCGTGGCTACCAATGCGTATAATGCGCGCCTTCACTTGATCCTCTCGGGCCTTATCCCAGTACGGCTCTAGGATATGAACCTGCCGCCCGTATTTGAGATCCAATCCCTCAGCTCCCGTTTTAGAGACGAGTAAAATTTTAATAACCTCTCCGTGTATATTCAGCGGCGAATTCCAAACCTGCTGGATCATGTCGCGCTCTTTAGATAAAATTTTCCCTGTAATAAGCGTAAATCGTGTTATTTTGGAGGACAGGACTAACGTATGGGTCGGCCCATCTTCGGCAAAGTTTTTCACCATAAGATCTTTCCCATCCTTATGAAGGAGGATGGTGTTGTGCCCTTCTTCCAATACTTTTAGGGGCTGAAGGCACTGGTAGCCCTCTATTTCTAAAAAGCGGGCCACGACGTGAAGGCCCAATTCCACAAACTGTGAGTAAATGAGCACAGGGCCCGGAGACGTTTTAATATTTTTTAGCATGCGCACTATTTTGGGACTAGAATTTTCTGTGAAGGCCTCTTTGGGCAGCTGCTGAACAGCCTCTGATAATTTTTCATCCTCCTTTACTGTTAGCATTTCGGACGCGAAGATGCTGATCATACGGGAACGCACATAGTAGGAGGAGCCTGACTCTTGCTCCGATCCTGGCAGGCAGAGGGCGGCGGCATTAATTTTTTCATACATTCCTGAGCTGGCGTGCTTTTCCGCGTTTTCAACGTCTCGGGCCAGCAGATATTGCCTATACTGCTCGGGTGACATTTCAACCTTTTCTATAATAAGAGGAAGCTCTGTGGGGAATAGCTTGTTGAGCTCATTCTGGTTTCCAGCGTAGCTTATCATACCCACTAGGCGGTTTAGTAGTTTGTCCGCGTTTAAAGGGCTATTCGTTGTTTTATTGACATAAGCGGTGTAGAATCTTTCATAGTGAAGAGGTAATAAGATTCGCCCGCTTAGCATATTAAAACAGGGCACCATTTCAAAGGGGTCCTTCGAACACGGGGTGCCTGTTAAAAACAGAATACGAATATTTTTAGCTTGCATAATATTATTGTACAGCTGGCGGGCATTTGTTTTATCATTGGCGCTATTGATAATTCCTCTAAAGAGGTTGTGTGCCTCGTCAACGATGAGCAGGCAACCGTTTAGGGACCCTCCCGCCTTTATGATCTGCTGCCCCATGTTGTAAGCGTCTAGGGACACAAACCTGAAGCGCCGCGAGATTTTTTGTAGCTCTTTGGAGTGATCCGTCGTTTCCGGATATAAAAGTTTAATAAGCTTTAGCAAAGACTGTTGGAAGTTTGAGTGCAACGACTTGGGTGCGATCAGAATCGGGTTGTAAATATGTGAAAGTGAGATGGCAAGCGACAGGCTCAAAATGGTTTTCCCCATGCCCATCTGGTGATAGATGAGGAGGCCCCGTGTGTTTTCCCCCTGGCCTATCCCAAATTTAGGATCCGAAAAGGCGGTGTAAATTAAAAACTGGTAGTATTTCAGGGCTCGTGCAAAGCGGGCAGTGAGTGAGGTGTCTTTGCTTTCCTGAAGCTCTTTATATTTTTCATATACCTCTTTTAGGTATGCTTCTATTTGGACGGGGAAGGAGGTGTTGTTGTGCACGCAAGACATGACTCGTTATAAGGATCCCATATTAAAACTTCATTAGAAGAATAGGGCTGCTGATAGCTAGCGCTGCACTTAAAAATGGGGTAGCCCTTTTTCTTGTAAATCCGGTGCCTGTCGTAGACCTGGCTAGAAAGCGGGCTTAGTGTATCTTTAATGTCCACAACGATGCGTACCTTTTTTTCATCCGATCCCTGCCGGGTAATACGTCCCAAGATTTGCTCCATGTTGTTTCTGCGGGGCGTTGCCATGATGATCGATGTCATATGCTTGAAGGAAATGCCTCTACGCCCGTAGCCATAGGTCAGCAAGATAATGGAAGCGCTGTGTGCCTGAGAAAGAGCGGTATTTGAAACCCCGCCGCATAGGAGCGCCACCTCCGGAACGATAATTTGAACATCTTTGAATTCTTTGGAAAGCGCCTGATAAAAAATTTCTAAAAGTTTGCGAAATTCCACGAAAATGATGATGCCATACGGCTCATCGGTCCCCCATTTGTGAGGCTCAGCGGTATGCAGGGAGTAAAGCCGCTTTGCCTCATTTACGACAAGTTGTATACGCGAAGGATCTTGAAGTAGTTTATCAATGGTGGCAATGGCCGATACCTTTTCATTAATATACACAGGGCTAACGAAGTCAGGATGTCCCTGATATTCGATTTCCCTCACGTACCCGGAAAAGGTTGTGGTGGGACTTACAGTCCTCTGGGGCTGTCCTAGATGGTGAATAATAATCTTGTCCATACCATCGGGCCGGTCCAGGGGTGTAGCGGACAGTCCTAATATCCGACTAAGTTGTATTTTCCAAAAAATTTTGTAATTCTCCGGCGAGTGTAATTCATGTGCCTCATCTAACACGACTAGACCAAAGGGCTCAAAGAACTGCTCAGGCTTCTTGCGCAGGGTATTAATGATTCCCACGATGACGTCGTACTCTTTGCTCGTCATGTCCTTTTTCTTGCACGCTGCATTATTGTAAGCAGCTACACGTAGGTGGGGCAGGAGCAATGTTAGCTCGTCGATCCACTGTATTTGAATCGCCTTGGTGGGCACGATGACCAGGGTAGGGTACAAAAGTTTTTGAATAATGCTGATCGCAATACGCGTTTTCCCCAAACCGGTATTTAGATGTAGGTAAAAGCGCCCATAGGGGGACAGGAGCTTTTTATGAATCTTATCGACCATTTCTTGCTGGTAGTTAAATAGTGGAAATTCTGTTTCAACGCATGGGAGGGCCCGCAGCGACACGGGGCGCGTCGTGTAAACCATGTTAAACATTTCAAACTGCTTTTGCAGCAATATGGGAAAATAAATGTATTCCCCCTGCAGCGTGAAGGCAGTTTCCTGTCTTATGGCTATGTGCTTTGGCTGCCCGGGTAATGCCCGCGCCGTAACGGTGAGCGCCTTAAGAACGCGCCCGAAATCATGTTGTAATTTACTTTGTAGCTTCTTATAATTTATTCCTATTCCAGCAAAGGATATAATGGCCTCCATTCTCACGCTGGACGGGTTATATGCAGAGGTTCCAAAATTCTTACCAGAGGCGTTACGAGAGGGCTGTGCTGGCAAGAATCCTCTAAGCTTTTATATTCAACAAATTTTAAATTTAATGGGATGTGACGGTAACGAGTACCATGTTCTTTTTACCAGCAGCTCCGAGGAAGCAAATACTCATATGATCATGGCCGCCGTGCGTCGCCATTTGCTGCGGACGCAGCAAAGGCCTCATGTCATTATCGGAGCAGCCGAGCCCCCTAGCGTCACCGAATGTGTGAAGGCATTGGCGCAGGAAAAACGCTGCGTATACACCATCATCCCCCTAAAAAATTTTGAAATAGATCCTGTTGCGGTATACGATGCCATACAAAGCAATACCTGCTTAGCGTGCATTTCAGGCACTAATGCTGTTGTCAAAACGTTCAACAAACTCCAGGACATCAGCAACGTGTTAAAAGGTATTCCCCTGCACTCAGAAGTGAGTGATCTTGTTTATCAAGGATGTATTCAACAAAATCCGCCCGCTGATAGTTTTTCAATAAATAGTCTCTACGGCTTCCTGGGAGTCGGTGTTTTGGGAATGAAGAAAAAGGTCATGCAAGGATTGGGGCCGCTCATTTTTGGAGGAGGGCTGAGAGGCGGAAGCCCTAATATACCCGGAATTCATGCCATGTATAAAACGCTAACCCAGCAAAGGCCTTCTATGAAAAAAATAAATACAATACATACGCTGTTCATGAAAACTTTAAAAAAACATCAGCATGTATATCTACCCATAGGGGGCGTGTCTGCAGAGGACACGTCTGCAGAAAACATATCTACAAAAGACATGCCTGTTGAAGGCCCGAAGGGACTCCCGGGCTATATTTTATTTAGCGTTGGCCGTCGCGCCGAGGAGCTACAAAAAAAAATTTTCACTAAATTTAATATAAAGGTTGGCCGTGTTGTTGACTTACAAGAGATACTGTTTCGTATCAAAATACCCCAAAAATACTGGGAGACATTATTGTTCATCCAATTAAGAGATAATTTGACCAAAGAGGACATAAAAAGAGTTATGGTTGTTTTGATGCATTTAGATACCATCACTCCTCGTGGCTCTCTTCCTCCTCCGAGCCACTCTTCTTCTTTTTCTTAATCGTTTTTGTTTGTTCTATAATAAGGGAAAAGAACTCCGTGGGATCTTGTTCCCCGTACAGGTTATCTGCGACCATAAGGATGCTTAGAATGGTAAACAGGTGAGAATACATAAGGGTTTGCGTTTTAAGAAAACCCTGACGTTGAATCATAATTGAAAACACCTTGCAAAGCCGACTCATCAGTTGTTCTGTAATGGCGTTAAGCATTTTCTGGAATTTTTCTTGGTTTTCGGGTGTGATTTTATATTCATGTAGAAAGTGTTTCACACCTGAGGAGAAGAATCTTTCCTCCTTCGAGAGCCCATCTTTGATGATGGGAAGTTCCTTGATCAGGGCAAACCATTCCTCCTCTTGGGCTTGCGGATTCTGAAGATACTGATGGCAGATATGGTTTAGAATGGTGCACACGTAGCTAATAAGCTCTGAGCTGATTCTTTGGTTGGTTTTCAAATGTTGGCGAAAGTAGTTTTTCACCGAAGTGCATGTAATAAACGTCTTCATTTTCTTATAATATACAACAGTATGTTGAGTCTTTAATTTAAAATTACAAGGAGTTTTCTAGGTCTTTATGCGTATAGGTGTTTCTTTGTCGTAAATTTTCAATAGCCGACATTGTTTGTGAAGCAGTGTTCTGAGTAGTGACTGTCGTGTAAGGCTCAGCCGGATGAGCAGGAGCACTCGCGGCCGCAGGTGCGGCCGCCGGCCCGCCAGTTGCCATGACTAGTCTGTCCGTAACTGGGTTGTCCGTAACTGGTTTGTTTGTTGCTGGTCTGTTTGTTGCCGGTCTGCCCGTGACTGGCTTGCCTACACTTGCTGTAGTCGCTCCAGCTGGTTTAGAGGTACCTGGTTGTGGAGTGACTTCTACCCACTGCTGATCTTGATAAGGATTTATAAACTGTATATCTTCCTCCTCAATAGCAGCAGCTTTTTTCTTTCTTGAAGAGAATAGATAGATTAGAACGATGATAATGATGACTAAGACCACGATAGCAATGAGAATAGTATACATATGTGTGGAGAAGAAGCTTGGTGTAGTGACTGGTGACAAACACTCACCATAATGCCGCGGATAAACCGGTTGAAAAAATTCAGAATCCATTTAAGATACTATTATAAATAATATATAAAAATGTTGTGGCGCAATGAAATTACAGAATTTATGGACCAACTTTCCAAGTATTCTCAAGAAATCTTAAAAACGTTTAAGCAATTGCGTCCTAGTGAATATAAACAATACAATGAATTTTTAACACAAGTTACACCGTTGCTGCAAAAAACCCCTGAAAAAATTCCAGAGTTGGTTGACCATATATTCAATTACCTAGACAACGTTGAAAAAATTTGTGAGCTCCTCGTGAATGCTAGCTCAATTATTATTAGTTCAAAAATACGAGAACAAGTAAAACACGGAATGAGCTTCAGCTATAAAGCCGACCTCGACTCCTTGGCGGACATTCTCTCTCAAAAACAGTACGTGCTTATGCATCTTTCAAAAAATATTGCGGCCGAGTATTTTAATACGTGTTTAAACCAAGGGAAATCCAAGTTAGATCTCAAAGCTGCCTCTGTATTTTATAGTAGTCGTTCCCGAACGGCAAGCTCAGCAGAACTCTATAGAAAAATGCTATACGCCTATGGTTCACCGCAGGAAATTAATTATTATACTGAAAAAGCCCGAAATAAGACGTTGGATGTGGAGGAGAGCGACAGCATGGCCATCATCGAACGAACGGCCCGACACAACCTTTCCCTTATGCACCCGCTAGAAGCCATGGGGCTTACCTTTGGGGCAACCAACACGGACGCCGACCCGGAGGATCTGAAGGACAAAACGGTGATAAATTTAACGCTCCCGCAGGCAACAGAAAGCATCACCTACCATCTTAAATCCCTAATGCAGCTAAAAAAAGTAAGTACGGCTTCAGGACTAAATACAAACATTTTGAAAGCATTTGATAATATTATTTCCACCCCTGTGAAAAAAAATAAAATGGCCTCCAAGTTGGCGCCCGGGATGGATGTCGTGTTCACTAGCGATAACGGAAAAACATTTTTTACTAAAAACATTTTAAGCAAAAACATGCTAGCGGGGCCCAAAGAGCGGGTGTTTGCATATAATAATCTCATTAGTAATTTAAATAACTCCTGTTTCATACAAAATCACAACGATTTTTTAAGACAGCAGGACTCTTGGCCCTTCTATGACGCGCACAATTTTACCAACAAGTTTTTAATGCAGCCTATTTTTTCGGGGCAGACCCGTCCTCGGCTTCAGGGAGCCATGGAGGCGGCGCATGTGGAAACGCATCTCACGGCATTTTTACAAAGTATTCAGCCCTCTAGGCCACAAGATCCCTCTGTTTTGGCTTCCCCCAAGTTATCTGCTCTAATCTTGAACTAAAAACAGCCTTTCTTGGACTTAAATGATGGTCTACCAGTTTTTGAAATAACTTAGAGAACTATGAAGATTTTCATGAAATTTAAATTAGAGATTTGCAAAGGTTACTTGCGGTCATTTTCTGTTGAATTAAATAATTATTCGAATAGTATAATGTCTGAAGATATTCGTCGTGGTCCTGGCAGACCGCCAAAGAAAAGGGTTGTTCCCAACTTTGAGCGCAAGGGCATTCTGGAAAAACCAGTTCGGCCACAAAGCCGTCTCGAGTTTTCCTATGATAACCCGCTGATATTTAAAAATCTTTTTATTTACTTTAAAAACCTTAAAAGTAAAAATATTTTGGTGCGATGTACCCCCACCGAGATTACCTTTTTTTCACGTGACCAGTCGCAGGCAAGCTTTGTTATTGCCACCATCGACGGAAAAAACGTGAACCATTATTACGCCAGTGATGTCTTTTGGCTAGGCATCAACAGAGAGCTCGTTGAAAAAATGTTTAACAGCATTGATCGCTCTTTTTTAAAAATTACCATCGTTCACCGCTATGACAAGCCTGAAACCCTGTTTTTTATCTTTACGGATTTTGACATTGACAAGGAGTGCACGTATCAGATTACGGTCTCGGAGCCCGAGCTCGATATGGACCTTATCGAAATGGAAAAAAGCATCAGTGAAGAAAGACTCAAGAACTATCCTCTGCGCTGGGAGTTTACCTCCAAGCAGCTCAAGAAAACATTTAGCGACTTATCAAACTACACCGAGCTCGTGACCATTGAAAAACTCGGCGGCGATACGCCGCTGCACCTGTATTTCCAAAAGTTTAACTCCATCTCATACCACGAGATGTATAAATCTTCCAACAAGATCAACCTGACCTCGACCATTCCTAAGTCGCAGGTGTTCCAGATAAATGTTAAAATTGCTCACATCAAGTCGCTGGCCTCGGCTATGGTCACCGACAAGATCCGCATTCTGTGCGAAGAAAATGGGAACCTAATCTTTCAATCGGAAATGGATGCCCTTATGTTAAATACGATTACCTTGAACACCACGATATAGTTCGGTAACATTAGATGTTCTAATATTTAGCATCTAAATAATACGCTGTAGTCCGGTCAGGGTTGCGTCACAGTTTTCCCATTTTTTTGCCTCGTCGGCGGTGGCCACCGTTGCCCTATCATTTACGCCCGGTAAGACAAAGCTAAAGGCGTTCAGCGGGGCTTGGCAATGCCCGCCCAGCGTGAAGGAGCTCGGAGGATTTTGCGCATCCCGAAATCCCTTAGCCATGTTGTTTAACACTTCGGTTACGTCAATCGAGTGAAGGGATCCCTTGGGATCCGTGAATGTAAAGACGCAGTTTCTAAAGCGCATGTATGCGATGGACGATTCATCGGGGGTTTTGAAGGTAACAGTGTTCCCCTTGCTGTACTTAAAGGGGGACCATCCGGTAAAATTATACCAAATGAAAGCAATAATAATTAAAATAACCAACACAATAGTTATAGACAACACAAAGTCTGTAGTGCCGCCCATTATTAAATAAAAATATTTTAGACCGCCGGCTTAAAATTTACTTATTGCTCATAGCTTAAGTCTATTTTATTCATAGCTTAAGTTTATTGCTCATGGCTTAAGTCTATTGCTTATAGCTTAAGTCTATTTTATTCATAGCTTAAGTCTATTGTTCATGGCTTAAGTTTGTTGCTCATAGCTTAACTCCATTACTGATAGCTTACTGATCATGACTTAAATAAAAATATTTTGCCCGCTTAAAAATTGTTTAGGTTTGAAAAAATAAGAGATGGAGGGGGCAACTTATCGTCATTGTGTTTACCCCCACTGGAAGACATCAAACGGTAAATAATTATAAGAATCAAAATGATTAATATAAGGGTTAAAAAAGGATGATTCATCACATTAATTAAAAACGTATTTATAACGCTGTTGCAGTTGAAATTTTGGTATAGGTCGGAAATATTGCCCGAGCCTCCGTATTTTGCAATGTTCTGACATATGGTGAGTCCGGAGGGGCACTGCTTGTTGGTCAAAATATTTCTTTGCTCCGTTGTTTTATAGGCATTTTTATTTCCATTACACGGAGCAAACGCACATTCAGCCCATAGGGTGCCGGAGTTCACACAGGCACAATACTGGCTATACGCATACTCATCCTTTGAGCACAATCCCTGTTTATCGCATATGCTCCCAATAATATTGTCATCCTCCGCCGTTTGTTGATTTGTATGCGAGCGTAAAATAGCGGCCCAGGCCTTGGGCTCCTTTTTTTGCAGCTCGGAAATCGAAGGGCCTGTACAGCTAAAGTCGACCCAAATATCATTGCATTTCGTGGAAACTGGCATGCAAGACATAATTGAAATAATTAATAAGTATATATCATGGCAACAAATTTTTTTATTCAACCTATCACCGAAGAAGCTGAAGCATACTACCCACCTTCCGTGATAACGAATAAACGGAAGGACCTGGGGGTAGACGTATACTGTTGCTCCGACCTAGTGCTTCAACCTGGACTAAATATTGTTCGCCTGCATATTAAAGTAGCATGCGAACACATGGGCAAAAAATGCGGTTTTAAAATCATGGCGAGAAGCAGTATGTGCACCCATGAACGGCTGCTCATCCTTGCAAACGGAATTGGTTTAATAGACCCGGGTTATGTGGGCGAGCTCATGCTCAAGATCATTAATCTTGGCGACACCCCGGTCCAAATATGGGCCAAAGAATGTTTGGTGCAGTTGGTGGCCCAAGGTGACCATGTGCCTGACCATATCAACATCCTAAAAAGAAACCAAATATTTCCGCTGTTTGCGCCTACCCCAAGAGGCGAGGGTAGATTTGGGAGCACGGGCGAGGCCGGGATTATGAGAACTTAATTTTATTTTTTTTCTTAACATAATGGGAGGCTCTACAAGCAAAAATTCCTTTAAAAATACGACCAACATTATCAGCAATTCCATTTTCAATCAGATGCAAAGTTGTATTTCCATGTTGGATGGCAAAAATTACATAGGCGTATTCGGTGATGGAAATATTTTAAACCACGTTTTCCAGGATTTAAACTTATCATTAAACACAAGTTGCGTGCAAAAGCACGTAAACGAGGAAAATTTCATTACAAATCTTTCGAACCAAATTACTCAAAATTTAAAAGACCAAGAAGTTGCGTTAACCCAATGGATGGACGCAGGAACTCACGATCAGAAAACGGATATAGAAGAAAATATAAAGGTAAACTTAACAACCACACTTATTCAAAACTGCGTTTCATCCCTGTCGGGTATGAACGTGCTGGTGGTGAAGGGGAATGGCAACATTGTTGAAAACGCAACTCAGAAGCAGTCGCAGCAAATCATCTCTAACTGCTTGCAGGGGAGCAAGCAGGCCATAGACACCACAACCGGCATCACTAACACGGTAAATCAGTACTCACACTACACCTCAAAAAACTTTTTTGACTTCATTGCAGACGCAATTTCGGCTGTTTTTAAAAACATCATGGTCGCGGCTGTAGTTATCGTTCTAATCATCGTAGGGTTTATAGCCGTCTTTTACTTTTTGCATTCACGGCACCGCCATGAGGAGGAAGAAGAAGCTGAACCACTCATAAGCAACAAGGTATTAAAAAATGCTGCCGTTTCGTAATAATTTAATTAAAAGTAAAAAAAAAAGGTATTGTTATAGTGATGGCAGATTTTAATTCTCCAATCCAGTATTTGAAAGAAGATTCGAGGGACCGGACCTCTATAGGTTCTCTAGAATACGATGAAAATGCCGACACGATGATACCGAGCTTCGCAGCAGGCTTGGAAGAGTTTGAACCCATTCCCGACTATGACCCTACCACATCAACTTCCCTGTATTCACAATTGACCCACAACATGGAAAAAATCGCAGAGGAAGAGGATAGTAATTTTCTACACGATACTAGGGAGTTTACTTCACTGGTCCCCGATGAGGCAGACAATAAACCGGAAGATGACGAAGAAAGCGGTGCAAAACCTAAAAAGAAAAAACATTTGTTTCCAAAATTAAGCTCGCATAAATCGAAGTAAAAATTGAAGCGAAAAAAAGTAGAAAAAAAATGTTTGGAGCTTTTGTAAGCCACCGTTTGTGGTCAGATAGTGGTTGTACGACCACCTGCATCACAAACAGCATTGCTAATTATGTAGCCTTCGGCGAACAAATTGGATTTCCCTTTAAATCAGCTCAGGTATTTATTGCCGGCCCTAGAAAGGCTGTGATAAATATTCAGGAAGATGATAAAGTTGAGCTTTTAAAGATGATTGTTAAGCACAATCTTTGGGTTGTTGCTCATGGAACCTACTTAGATGTGCCCTGGTCCCGTAAGAGTGCGTTTGTTACACATTTTATACAACAAGAACTACTTATATGCAAGGAAGTCGGTATTAAAGGGTTAGTTTTACACCTAGGCGCTGTGGAGCCTGAACTTATTATGGAAGGACTAAAAAAAATTAAGCCGGTTGAGGGGGTTGTCATTTACCTGGAAACCCCGCATAACAAACATCATACATATAAATACAGTACAATTGAGCAGATCAAAGAATTGTTTTTACGGATACGAAATACCAGGTTGAAACAGATTGGTTTATGCATTGATACGGCTCACATCTGGTCTTCCGGTGTCAACATCTCCAGCTATAATGACGCGGGGCAATGGCTGCGCTCGCTGGAAAACATTCATTCCGTGATCCCACCAAGCCACATTATGTTCCACCTAAATGATGCCGCCACAGAATGCGGAAGCGGTATAGACCGACATGCAAGTCTTTTTGAAGGAATGATTTGGAAATCATATAGCCATAAAATAAAGCAAAGCGGTTTATATTGTTTTGTTGAATACGTTACGCGACACCAGTGTCCGGCTATATTGGAGAGAAACCTCGGGTCTTCCATGCAATTACAAACCGCTTTAACCGCAGAATTTACTACATTAAAATCGTTATTAAAATAAGGATGAGTTTTAGCGAATGTCCCTTAGTTATTAGTGCATGCAAAAAATTTCTACAAAAGCGTATTACAATAGAGAATGAAGCACTTATAAATGCCTTAATAACCGCTTTAGCGCAGACCAGCACGTTGAATGATCTTTGTTTATTACCTATTCAAACCTATTTGCTTAGTTATAAAAATGCTTTTGAGTGGATACACTTCGTATGTATTGCAATCACCACTATTTTGGATAATAAGTATAACTGGAAGGACTGTACGGTAGATATTAATTATATTTTTCTCCATGTAACCTATATTTACAATATTAAAACCAAGGAATACCTAGACTACTGTTCTTAAACTTTATTTTTTCTATATTTACGCCAAAGAGAATATTTAAAGTTTTTTTTGAAAAAAAATAATATATGTAGATAAAATTCAGTTACATGATATATGTGTAAACATGTGTGGTAAACAACATATGGTTATGCTTTATAAGATAAATGCGCATAATATATGTAAACAAAATATGGTTATGTGTTAAATGCATATAAATGTATTTTAACGTATATCTTGTGATAATGGATATATGCATTTATTAAAAGAGGCTGTATTTATTATAAATCTTGCTAAGGATGCCATTGTCAACATATATCCCATGTTGGACAAATTGCGTTGCGATCCAGTTCTTTTTTTTTGATTTTGTTTAATGCTATCCTTTTTGAAGGGATGGTTGTCCACCATATTTATTCGATGTTCAATGAATAGGTCTGCTTTTTCGTAAGGCAGTGAAGGTCGTTCCAAGACTCCTTGAACGAAGGACGTGTTTTCTTGGATCCACTTAAAAAGCACGTGGCATTCAAAAACAGGACAGTGATTGGATCCTTGGATATGCTTTGGACAGCCAATGCTTGAAGAGATGTAGTCCCTTTTCTTTAGGACAAGCTTCTCCACGCTGGGGCAACAGAGATCGTTCAAGTTCTGGACGGTCGCATTTGGAATGTTGAAACTTCGTATCCATTCACCCTCGGGTCCTCCCTTATGAAGAAGGAGTATTTGCTCATGGTCCTTAGTAATCTTAACCAAATGTTGGAAGATCATTTTTTTACCTGCTTTAAAGGCCTGAAGGGTGTCAGTTGGCAAAGCTATTGAATTCGGGAGTGGGCTTTCATCAAGCGTGAAATGGTGAATGTGACGCGACTGGAAAGAAAACGACCGTTGATTTATTTTTTCAAAGATTGGGTCGATTCCGCCATGAAAGAACAGCTGCAAGATTTTAGAAGGCGTATTTTTTTCCCAATAAAAAATGACCACTTCTCGTGGGATTAAAATCGTCTGTGTCCCATTTTCATTATATAATTGGCCCATAAAGCCATCAACGTCAATCAACACCAAAAGCATGGTATAGAGAGCTTTTAGAACCGGAGTTCGTTAAAAAAATACAAAGTTCGTTTAAAACGTGTAATGTTACTAAAAAAATGTAATGTTTAAATGATAATGATACCACATGCATTAATGAAAAAAACTTTTAAATTTTTGTTTTAATATTTGCATGAAAATGGAAACATTTTTAGTCTGTTTATTTCACAATGCAGATGGTTTACATCAACAGATTCAGGAAATTTTGTATTTATTGCGGATGCATATTTACGAAACAAATCTTTACTTAAAGCAGGAACTATCACGGCTTATATATCCAAATAGGCAACTTTCTTTTGTGTTACTTATGCCCCTTTCCCTTCTAAGAAACTGGGATGACATTGAATATTTAACGGACGTTGTAGATGATAAGCAGACTCTACATTACGCGGCAAATTTGCTGACAAACTACGTTCTACATCTATCCATGTTTCAAAAGCTGACAAAACCATACTTCCTTTTAGCGGTCAAGCGGGTCAGCGAAAAACTCAACAAAAAGCAGCGACATTCATTTTACGAGGTATTGGTAACCTCCGAAACCTTGAATAATTATGAAAACCTATCTAAAAACATTTTAAATACGTTGATGTTTGCCGTGCGCTACGTATTTAAACCTACGCCGAACTATTCAGAAATTCTCGCAGAGTTGGAAAAAAAAAATAAAATTCACCATATTATTTTTAATATGGTAATTACGGATTTTGCGCAAATCCGTGAACAACAAATGGATAAACATCTGTGTGAAACAAATAATGAGCTTCGTCAGGAATGTAAAGAAACTATTTTTGATTTAAAGGTGGTAGGAAATGTTTAGCCAATAAACTCATGCCCGCATTTTTTACAGGTACAAAATATCGTGGATGGCTCATCGAGGGCGCGTGTTTGTACTTCTCTGTAGGTACACATACGCTGCTTGCAGTTGGGACACTTATAAAGTTGTGACGTCTTTTCGGCGACCTTTTGCTGCGAACGTAGAGTAATTTCTGTCTTCTCCTTTAAGGCGGCAGAGGGGCAAAGCTCGGCGAACGTCATGCTACCAATTGCCTCCGGTTTTAGCTCGCCAGAAATTAGCTTATTAAGGGCATCGTTATCCTGTTGTTGGTGACTTTTTTTTTCGCAGTTAATAATATGATTGATCGTCCCACAACGGGTTGAATATTCTTCTAAAAAGGTTTTTTCTTGTTGCTGGTACGTATAATGATAACACGAGGCCTCGATTTTTTGCGCGTATTCGGTGCATAAATCAGTATGTTCCTTAAAAAACATATGTTTTTGAAGCGTTCTAAAAAACATCATTTGGATGATATCACGCATTTCCAAAATAATATAGGGTTCTAGTCTTTTGGAATCTTTCATAACTAGATCGGTGGTAATATTCTTAGTCATACAATTTATTAAAAATGGTTTAATATATTGTAAATATTTTTTAGGCGTGTCAGCCTGTAAAAAACATTCTTGTTCAATCTTATTTGTAAGGATAGTATTTTGCAAATACTTATTTAGCAAAAATACGATAGAATCGCGGGCTATATGCATTTTCATATAATTTTTTTTTTAAAATTTAATACAAAAAAAAGAAGTATAGACTCTTCTTCTAGTCCGGTTAGTTCGTTGGTTGCCTCAACATGGAGACTCAGAAGTTGATTTCCATGGTTAAGGAAGCCTTAGAAAAATATCAATACCCTCTTACTGCTAAAAATATTAAAGTAGTGATACAAAAAGAGCACAATGTCGTCTTACCTACAGGATCTATAAATAGCATACTGTACAGTAACTCAGAACTTTTTGAGAAGATTGATAAGACAAATACCATTTATCCCCCGCTTTGGATACGGAAAAACTAATTGTAACCAGTAGTACATTTAAGGATAGTTTAAGCAGTAAATGTAGAATAACACAGTTAAGCAATAAATAACAAGTATATAGGAATATATAGGAATATATAGGAATATATAGAAATATATAGAAATAGCTAAGCTTAATACTAATTCAGCTTTTTTTTTAACTAAAACCTGAATAGATGCGAAGTAGCGGACATATACATACTAAAATAAGCCATACATTTACTTTCTTCTTGAACATGAAACCTTTTTTTCTTCTGTTGTTGGTATATAAACAATAGGACTGTTTGCTGAGGTTGTATGATCTTCTACAACTGCTGTCTCAGGATGACGATGTTTTTTTAAACTAAAAGTGTAGGATGGAATGAGTGGAATATAGTTATGGCTCGACTTATCCTGTTTCGTACAGGAATATTTTTTACAAATAGAACGCAACAAGCATATGAATAAAAACAGAAATGATATACAGGAGCATAAAATAGATATGAACACTAAGGGGTAGCAGCTTTTATAACGTTCCGTATTTTTCTTAGCTATCAATTGATTTACCGTAATATTTATCTCGGGAAACTTTGTTCTACAATATTTTGTTTGGTATTCCAGAAACTCATGTCCTGGCTTATTCCCGCAGCTTAAAAAATGATACAAAAATGTGTTATTGTTACTAAAATTAATTCTTCTTAAGAAAAACTGCGGAAGACGCTTTAGGTACGTCTGTTCCTGTTTTAGTAGGAAGTAGTATAAGGGACAATTTCTTTTTCCACACATTAGATTATTGTAATATAGGTAGGTTGGGGTGTTGGAGCGAATAAGTTTTCTGAGTATGTTATAATCTATGACTTGTAAATCGTTATACCTTAGGTCCAAAAACTTGAGTTCTTTACCAAAGCCACCTGCAATTTCAGAAATATTTTTCATCCCGCAGCGGATAATACGGATGTCCTGAAACGTCTTTAAAATACTTGTATTGTAGTGAATACTTATGTTATTTTTTTGTAAATAATCTATGTCATGACAAGTGCATGAAATGCCAGCAGCATTGCTTGGTATAGTATTATATGCAGGAAGAACTATACTACTATTGAGAATAGTCACATTGTACTTATACCATGTATTATTTTCTGATATAAAGTATTTGCAGGTGACCTGTGGTTTAATCCTACCTGTTAAGCCACTTCCTAAAAAAACAAAAAATATGAAAACCCTTAGCATCCTGTATATACTATTAAAAATTTATAAAATTTTCTGTTTAAATTTCATTTAGACAAAAAAAATAATATATATACATCAGCAAGAAATTATATACAGATTATATAATTTTCTGATTTTTTTTTGCCACAATAAGCATCATTATATGCATTAAAATCTCAATACTAAACACTAAAATCTAAATTCTAAGCATTAAATTCTAAGCATTAAATTCTATGCACTAAACTGTAAGCACTAAAATCTAAGTAACTAAAATCAACACTAAATGTATGCAACCTAAAATGTAAAGCATTACTCATCATCCTCCTCTTCTTCATCCTCATCATCATAGGTTAAGATATATGTGTCATCCTCCATTTCTTCACATTCATCTTCATAAGCATCACTGGGTATTGGTGGAACATTGGATGCAGCATTTTTAAAATATTCTATGTCTTCTGGTGAACACTCATCTAATGATTTTTTGACAGTCCTTTTAACTTCCATGGGATATGATTCCAAATCCTCTTTATATAAGAGTTTACGGTAGCTTTTAGCTGCATCCACATTTGCTGGAGAATCTGGATTTGGCTCATTGAGCAGTGAAATTACACTAAGAAGAATGGTATCAATCTTTTGAGCCGGAGACCAAGTCATTCCCTGTTCTTCAGCATTGTCTCCGTGTAAGATAGAGATACATAGTTTTCCATCAGAGTAAATATTAGGATGCCACATTTCAGAGGTGAATGTTAATCTGGGTGGTGCATATGGGTATTCTGGAGGAAAGGCGATTTTTGCCTTGAATAAGCCTCCCTCATAAAAAGTGTCAGGTGGGCCCCTTAAGATCACATCCCATTCAGTCATATCCTTCTCATTCACCGAAATTTTGAAATTCTCAGAGGGATTCTCTATCAGGTGTCTGTACTCTGCTATTAAAAACCTGGAAACCATGGTTATTTAATATTAATTAAATTCCCTGGTTTATTCCTCCTTAAAAGTAGATGAACCTCTTTTGTTTTTTATTGGGTTCATTTTTACTAAATTTATGAACTGGAAAAAACTTTAACGGCATAATTATCAAATGCGAAGGGGGATCCGTATAAAATCCTAGCTTGCCGGTAATGGCTATTAAGTTAAATTTGGTACCAGTAACACTAATATTTAAAAAGCCCTGATCATTAACTTTCCACATTAAAAGATTATTATATTCGAATGTTTGTCCAATATGGACAACTTTGTCACCAGATGTTACATTTGATTTGGTTGTTAGTGGCTGAAGCTTGGCACAATCAAAAATAAGCCCATTAACACTAAGATATAGAGGAGTGGGTTGATCTATTTTCTCATAGTTTAATATTCCATCTTTCCACGTAATAGCTTGATAATTATCCGCAGCAATGAGTTGAAATTTTATAAATAGTACAGGGGTTTTAGTTGTCGTTATACATTTAAAGGGTGTTTTATAAAAATAAAAAATAATAATTGTTAAAAGTATGATAATAATCGCCAAAATAATTTCATACATTTTTTATAAGAATTATACATAGTATGGTATTTAAAATATTAGCTAAATTTAAAAAAACTTCATGATTTTTAAAACAGGGAAAAAGGGGATTAGGTTGAATAAAAAAGGTAAGCACTTGTCTATATATTTTTTTTACAATGTTGCCTTGAGTCGCATTTTTAACTGGCTGGGGAGTATCAGAGTGGAATATCACTGTAGTAGGTCTATAAGGTCTTGTTAAAATATGATCGGTCATTGTTTTCGTACTAGTGTCATTTAGGGTCGACCTGATAGCTCGATATAAAGTTATAGGGGATAACCTATCAAATACAGTCTTATCTGTGCTGAAATGTATATCGTCTTCTTTATCACTAATAATATTAGGAATGGCTGTCATTAAATAATTACTACTTGTTGTTGTGGGTGAAATAGTTGTACTGGTATTATTGGAAATGGCTGTCATTAAATAATTACTACTTGTTGTTGTGGGTGAAATAGTTGTACTAGTATTATTAGAAATGGCTGTCGTTAAATAATTACTACCTATTACAAGTAAACTAATGCTAACTACATTTTTAACCTCAATAAACCTAAAAAGCCATACTAAATACCTAAACAACATCCTGTTATAATATGAGCAGAAAAAAAAAATAAGTATAATTAGGGAATTATTCTTATTCGCTTACTATTAAGAATAATTCAGAATCTTATTTAGTTAGAAACTATCATAAAGTGAATAGGACTCATCGTCGGATGAAGATTCCGTTTCAGAGATAGTTTCTTTTTCTTCCTCAGAATAATCTGTTCCTACAATAGAATCGGTGTCATCCTCAGAAAGAGAAGTATTTAAATATGGACTATCTATAGCAATATCCTCTTCTATCTCGCAATCCTCCTCCTCCATTTCCATAGTGTGTAGGAGAATATTTTTATCATCATGCTCACTTCTTTTTTTGTTGAAAGATGAACCGTCCTCAATACGGTTCATGTTAAGTTCCTTCATCTTATGTATAATTTCCGTAATCCGTGATGTTTTTGACATGTAAGATGGTTTTAAGGTTATATCCACAATAACAGGAGAATCTCTATCATTTTCATTTGATAAACTTTGATCTTTGATTTCTTCGTCTAAAATTCTTGTCTTTTTTTGGGTACTAGATGAAATAGAGGAATTCATATTCTGAAACGATATATCAAGGGGAGCTGGACGCTTTTTTCCAATTAAACCGTTTTTCGAGATACTATGATTAGATGAATGATCTTTAGCCAAGCTGTCCTTGGATATACTATAGTTAGATATTTTACCTTTAAATAATATTCTTCTATACAAGTTATTCTTAGGTAAAGAATTAGTATGGATTCCTATATTTTTATCTGAAGGAGTGTCCATATCGGAGAACGTCCTCTTACGAATATTTTGACCACGAGCCATTTCATCCACTATAGGCAGTATTTTGGCTGGCTATGGTTCTTTGTTGTGACAATTCTATGAGATTTGATTGCAAATCAATTTTTAGTTTTAAATATATTGGTACCTAGGACAAAGAAAGTATATATAGCCAATAATTATTCCACTAAATTGATTTCCAGACTGATGGGTATGGAGCCATGTTGTCTCTGCAGACGATCGCAAAAATGGCCGTAGCAACAAACACCTACTCCAAGTATCACTATCCAATACTGAAGGTCTTTGGGCTGTGGTGGAAAAACAATACGCTAAATGGCCCTATTAAAATATGTAACCATTGCAACAACATAATGGTAGGAGAATATCCTATGTGTTACAATCATGGAATGAGTCTGGATATAGCTTTGATTCGGGCAGTAAAGGAGCGTAATATATCCTTAGTCCAGCTTTTCACCGAATGGGGGGGAAATATTGACTATGGGGCACTTTGTGCTAACACTCCATCTATGCAAAGATTATGTAAAAGTTTGGGAGCCAAACCACCAAAGGGCCGAATGTATATGGATGCTCTTATACATCTTTCAGATACCTTGAATGATAATGATCTGATTAGGGGGTATGAGATTTTTGATGATAATAGCGTGTTGGATTGTGTCAATCTCATACGACTCAAAATAATGCTTACCTTGAAGGCCCGTATACCTCTCATGGAACAACTAGACCAAATTGCCTTAAAACAACTTCTGCAGCGATACTGGTATGCCATGGCTGTACAACACAACTTAACAATCGCTATCCACTATTTTGATAATCATATTCCTAATATAAAGCCATTTAGTCTGCGCTGTGCTTTGTATTTTAATGATCCCTTTAAAATCCATGATGCTTGCAGAACTGTAAATATGGATCCTAATGAGATGATGAACATTGCTTGTCAACAGGATTTAAACTTTCAAAGCATTTACTATTGTTATCTTTTAGGGGCTGATATTAATCAGGCTATGCTAATGTCTTTAAAGTATGGTCATCTTTCTAATATGTGGTTTTGCATAGATTTGGGGGCGGATGCCTTTAAAGAGGCAGGGGCGCTTGCTGAGAAAAAAAATAAAAGAGTGTTACAACACATATTAGGTCTTAATATCTTTAAGCGAGAGTTGATTCCCCCCTGTAAAGATCCTGATCCTTATCAAATCCAAATTCTGTTAAAAAACTACATTCTAAAAAATGTCTCAACTGTTTTTACATATTATTGCCAGTAGCCATTGTTTATATCAGAAAATAACCCATTTGTTTATCTTTTTTTGTGGGGCAACCATTAAGACCCGACGCAAAAAAAGATTAATCTTTTATCAGATACCTAAAACGTTCTATAAGGGAGTCTATGAGATGGATCATATTTTGATGGTCATAGTAAGAAGCAAGCTTTTTGGCGAAAACAACGGAGTTAAAGAATTTAACCCGCTCATGTTTGGATAGGACTTTTAACAGCGAGCCAAAACAGTATTTAAAAATTTGGCAATAGTTTTTTTGGGATGCAATAAACAAACACTTGATCAGTGCCCGCTTCACTTTCTGATCAGACATGTTTGCCGCATAACAGGCCTTTTTAAACTTAGTAATATAATTATGTTCCGCAAGCACCATTAACAAGGGAACGATGGGAAGCTGCTTTTCTTGGTGAAATTTACGTAAATATTCGATGGCCACCGCTTGGACGACTGTGTAATTTACTAAGTTAGAAATGATAGCTTTCATGGTTGTAAAAATATACATAGGATTTTCTTTTTCTGTATACAGTTTGAAAAGCTTATGATTACGTGAAATGATGGCCATTTTTAATACAAGATGGTATAGTGTATCTTTAGGTAAAAATGCCTTGCAAGCCGCGATGATGTCGATGTTGTCTCCATGAACAGCGATAGAAACTAATGTTTCCAATCTAAATGTTTTTATCTGCATTAATAGAAGAATGCAGTCAATGTTATTATACTTAATAATACTGTAATACACCGAATCAATGACCGTCATCTGAGAATCAAGCTGACTTATTAGTAAATTTAACGTTTTTTTGGAGGCATGACCTTTGATCGCGGCACTAAGTGCACACAGTATAGCAAAATTGTTAAATACATTTTGATTTAGGAGAAGGAGTAATATTTTCCTTCGGTTATAGTACGCAGCATCTGTGATGATTATTGGCCGATAAATGTTAAAATGTGTTAACAGCTTTTTAAAAAAACGGAAGTAATTTTTTTGGATCGCTGTTTGCATCATCGAAATAATGAGATAATCAGGGTATATAATGGGTAGGTCACATGCTACCTCTAACAAAGAATAGTCGCCCAATCTAAAGGCTGTGTTGAAAAGCGTACTATCATCATACGTATCGAGTACCCCTGCTGTTACAAACCAAGCGATAAGATGAATGTGCCGTTCCTTGCAAGCTATCGCAAATAGGGAGTTTCCTATGGAATGTCGAATAATGTACTCCCTATTTTTTTCCAAAATGTTTGGAAAATTGTATAGCGTTGCGGCATACAGTAGACACTCCATTCTGGCGTTATAATTTTTACTTTTACATATGAATAGGTGGAAGAACTCGAATAATTCTTGAGAACTTGTTAAATGCATAATATGGTGATATTTTGGTGTCGTTAAATGGTATGAGAAAATGCATTCTAATACATCTTTTCGGTTATGCTTTAGCGCCTGAGCTAAGGCATATTCAGGCTCGACCCATAGGACTAGTGTTTCTATAATTGAGATATTCGCCTGCTTTGCCAGGGCATACTTTAAGACGCTCCGGTTAGAAAAAATGTTGTTATGAAGATGGATAACCGTATCCATTTTTACGATGGGACCATTCCAGTATAGTCCTAAATGCTGTAGCAGATCTTTTGTTAGTTGTGAAGCGTTCTCGGGTGTCATATAAATATGTTGCAGGGCTTTTTTCTGTAAGGAGAACATTTCGTCGTAATCGTACAAAAAAAAATTAAAATTTGGGCATGGATGATTCAAACATAACAAAATCAAGATTTTATAACAGTTTGCATTAACCTATACATATATGCAAGTAAATGAGATATTATCTATCATAACGAATCAAGGGATATTTGTATATATCAGGAGTTTCTGAAATAAAGATATGAAGATTATCATAGTAGTATCCATCAATCACAATGCAACTTCCTTTAAGGCATAATTTAGTAAACTCAGCACTCCCATCTTCTGGATGCTTTACAACTAACATTAAAAACTCCTCAGTCATATTATCTGTAATAAAATAAGATCCTCCTGGAGCCATTTGTAGCATGTCTCTTATTCCTACAAAATCTTTTTTGGGATGGTAAAAACTCAGCAGTTTCAAACTCTTTTTTAGTTTTTTTTCCTGGTATTTAAGCCATTTGTTATAAAACAGTTTTCTTATGAAAATGCATTTGAAAATATTGGGAATGTTTAACCATGCTTCTTCCGAGCACATCTCCAGATACTTACTTTCTTTGTTTCCCATGTCTAATTTATTGCTCACTAAGTTAGTAATGAATCTATTTTAATAATCTACTTTACTAATCTATCTTAATAACCTATCTTATAATCTATCTTAATAACCTAATTATAACCTATTTATAATTGGCTAATGCTGCCGGCATTTCATGCCTATCTAAACAACTCCTACTAAGCAATCTACTATTACATATATAGATTCACTTTTTATATTTGTAAATCATGAGAATTATAAAATCATTACTCATTTTTATTGTAAATTAGTGGGTATTTGTAAAAATCTTCAAACGTTTTAAGATAGTTTTCTAGAGAGAAGTAATCTTTGCCATCAATATATAATGCTTTTCCTTTAAACTCCAGTTTTGCTATGTTTAGTGAGCCGTTTCTAGATCTTTTTGGGCAATAAATAGATTTTCATTGGTTGCATCGTCCGTAAGCAGAAAGGTACCACTAGGCACGTTAAAAAACATACGTTCTATTTCATGGTCGGATTTTTGAGAATAGAAAAAATCTAATTTTTTAATCCGCGTTAACTCTTTTTTATCAATCTTTCCAGACTGTTTTATATATACTTTATTGCAAATCTTACAATCCTCTATGGCTTCATTATACTTATTTTGCTTATCCTCTATTGACATGTCCGTATTTGATAGGTAACTTCCGTTAAGGCGGTTCCCCATGGTTTTAGATAGATTTTTAATTCAGTTGTATACTTTTATTATGAGGCTAAAATATAGAAGTTTGATCCTAAAAAAATAAAAAGATTTTGTACATTTATTTATGGTTTATAGCGGTATAGAGGCCGATAAAAGGTATCCGGGTAGTCTCCTATGATATCGTCAATTTTGGTATAATAACAGTTGTTATGGTAGTATTGTCCAAACCGAGTATGTATGCGCCGGTGAAGCGTCCGCCCGCTAATGGTACAGTTCCAGGTTAAGACAATCATATCACACCCAAAAAGAGAGGAAACAGCATAGGTGCCCAAAGGTTCATTATATAACATACGCCGCATATATTTTAGTTTTTTTTCTCCATGGTAATAATCACAGGTTTTCATGTCCTGCTTAATAGGATGATTCCCCATGTATGATAATATATAATAAATTTAGTTTTTAGCTTTTTCAAAAAATTGGGCGCTCGAAACTAAATTTTCCTTATCACAGCGTTTGGAGAAAGCGTATTTAAAGATATATCTTCTTCTAACAAGACTGCAAAAAAAATCTTACCCCTTATTTTTATAATGTTCATCATAGCGTTTGAAGATATCAGAAGGTGCCAGGTTTTATAAAAATATCCTTTAGGATTTATAACGATACAAGGGTCTATAAAATATATGCGGGTATAATCTTATAAAATCATCGATTTTTTCATAATATTCTCCGTTTATACAATAAAGATCATAACAGATATTGATGCGTAGATGCATTATTCGCGTGTTCGTTGGGCAGCTAAAGGATATCACAACGTAGTTTTTTTTAAGAAAAGACGAAACTACATAAGTCCCTAAGGGTTCATTGAATAGTAAACGCCATATTTGTTTTAAATTTTGTTGTTCACCATAGTAGTATTCGCACTTTTTCAAGTCTTTTTTAATAAGCCTATTCCCCATGTATGCTTATAAATAAAAATTTAGAAATGTGCTATATTATTTGTTGATGAATCATGAACACGTCTTATATGTTGATATGTTACTTTAAAAACATTTGTATTTTCAACAGACGCGTTCTATTCTTATTAAGAATGATGCCGTCTTTATTTTAAACCTTGGTTTAAAATTTAAAGAAGTATTTATAAACTATAATCATGGGAACTTTTTCAGTAACTGCCTCTGCAAAAAGTGACGATGCTGTTTGTAAGTATTTAGAAGAACCAATAGATGAAAATTACAGAAACATATTAAGAAATGAGCATGTTAAAAAAAATTTAAATGAGGCTCTGAATCGACATATTACTACCTATAATCCAGTAGTTGATTGGTGTAATAACTATTCAACATTTTCATCTCAGGATTTCGATGAATATAAAATTTATATACATAGCGATCTTATGGATGGACGACCTCGTCCAAAAAAAAACATGGTGTGTCATCATGTAATGTTTGTTAGTTTTATATAAACGCAAAAATATTCTTCTAGGAGATGTTGATATACTACCTATTGAATTCAATATATTAAAGTACATTTCTGGCTATTCCCATTACGGTATTATTATTACTATTTTTAAGAGCTAGATGTGGATTTAAGTAATAATAACATTCTCCCGTTCCTCCTAGAGACACCTCATCAAATTCCCATCCTATGCAACCTTTATGTTGTAAACATAATGATTGACAGCATTCATCTTCTTTTGACCAAGTCGTCCAAATCCTACCAAGATCTATACGTGTTTTTCCAAATGGAGATTGAAGATCAGCAGTAGTGGCATTAAACCTATAAAAACCAGGTGCATAATCACATGAACGGATCGTAGGATCTAATTTAATATCTTTTATATCTTGTTTTACTGCTTCTAGACAACTTTTATCAGTACATGTTCCACGTACACAGTGGTGTCCTTTATCCTTACAATCCGTATCTGTCTTACATTTTTTTTTCGGCGGTTTATGTTTCAGATGGTAAAAACCCAGTATTAAAATAATCACAAGAATAATTCCTATAAGTACTTGAACAACAGGATAAAACATTTTAATATTAAATATATTTTTTAATTAAATGAATAGATTTAATCCAAGTAGTATTAAAATTTTTTAGAAATAGTGTTCTACAAATAATGAAATGAATGGTCCAAAAAAAATAAGGTGTACAATAATGTAATATATTGTTAGGCTAAGTAAATTTAATATTTTAAAGTATTTGGAAAAATATTTTTTAACATATGATGTCTAGGAATATTTTTTAGACATTTAAAACCATATAGTTACTTTATTTATTACACTGAACTTGAAAAGACTTATTACCTAAAATATTAATAGATGAAGTAATATTGTGTAATTGAGTCCATAACATGGGTGGGAAACAAAAATCTCGTAATATGAAAAATAAACATCCTAAAAAGAGTGCAATTGTTATAAGTTTATGTAACTTTATTTTAAAGTAAGAATATAAAAATATGAGTACAAGAGGAATAGGGGCCATTACTAACATTGGCTCCAACATCCTGTTGTCTACAAAAAAAAATATTTTTTTTAGCAAAAAAAAATCCATGGAAGGATATTAATACACATAATTATTTGACATCACATTAGTGTACTTACCAAATAGTAATATACAACCATCCTAATATTCACCTTTATGAAATGATCCCAACCTATACGGTAAAATAGTATAGGTTTTAATAAAGAAAAAAGATATTCTGTGGTTTTTATTTTTGTATAGTGTGTGAATACAAAATAAAATCCCAAATTTTAACCTTTTCTTTTTTTTTCTATACAGGATGTTAGAAATAGTATTGGCAACGCTGCTAGGCGACCTGCAGCGGCTCCGGGTTCTTACCCCTCAGCAGCGGGCAGTTGCCTTCTTTCGAGCCAATACTAAGGAGCTAGAGGACTTCTTATGCTCAGATGGGCAGTCTGAGGAGGTACTGTCTGGCCCCCTTCTTAACCGTCTACTAGAACCCTCAGGCCCTCTTGATATTTTAACCGGATATCACCTATTTCGTCAGAATCCCAAGGCAGGTCAGTTGCGCGGCCTTGAGGTCAAGATGCTTGAACGGTTATACGATGCTAATATTTACAATATACTGTCTCGGCTGCGGCCTGAAAAAGTTCGCAACAAGGCTATTGAGCTATACTGGGTTTTCCGAGCTATCCATATTTGTCATGCTCCTTTAGTTTTAGATATTGTACGATATGAGGAACCGGACTTTGCTGAACTGGCCTTTATTTGTGCTGCTTACTTTGGTGAACCTCAGGTAATGTATTTGCTCTACAAATATATGCCTCTGACCCGCGCAGTTCTTACGGATGCCATCCGGATAAGTCTTGAGAGCAACAACCAGGTAGGGATTTGCTATGCTTACTTGATGGGAGGCAGCCTCAAGGGACTAGTCTCCGCCCCACTGCGTAAACGTCTGCGCGCCAAACTACGCTCGCAGCGCAAAAAGAAGGACGTTCTTTCACCCCACGACTTCTTACTGCTGCTCCAGTAGCTTTTTTTGCCGCAGGAGCACCGCGGATAGGAGCTCCTCCACGCTCGCGATCCGGCGCTGGAAGCGGAACCGATCGACCGCCACCTGCTCCCAGGGACCCTTGCGCTCGATGTCGTCGGCTTCCCACACCTCGACGGCTGTGGCAAAATGGACATGCTTCGCGTCGTTCGTCCGTTTTTTGCGCCGCCTCCCCATTATTCTTCCTGTAAGATTAGTGTTTAATACCTATAATAACATAATTTTAAGATTTAATATACCAAAACTTAAACTATTTTTGTATAGTAACTATTAGCATGTCTACACATGATTGTTCTCTAAAAGAGAAACCGGTTGATATGAACGATATATCTGAGAAATCAGTTGTCGTGGATAATGCACCCGAGAAACCAGCTGGAGCGAATCATATACCTGAGAAGTCGGCCCGCGAAATGACATCATCAGAATGGATTGCTGAATATTGGAAAGGTATAAAACGTGGAAATGACGTGCCATGTTGTTGTCCAAGAAAAATGACCAGTGCAGACAAAAAGTTTTCAGTATTTGGTAAGGGATCCCTAATGCGCTCCATCCAGAAGAATAATTAAAAAAAATATTTTTTTTAGCAAGTTTTTAAACTATTTAAATAAATGTGGTAAAAAAATTCACATAATAATTAAAGTGAACGTGTTAGAATTAATATTTTTTTATAATCGGATATAATATCCATTAAATCAATAAATGATAGTGTTGCTACCACACTAAACAATAACAAACAGAAACGCACGATACCTTTCCTCATGATTTATAATAGCGTGTTATCTAAAGATTTTTTTGAAAAAAATATTAAATTTTAGTTGATTATTTTTTTCAGTTACAACATTGCTTTAGAAAAAATACCTAATTACTACATAGCAAATAAAGCGAGCGCATTGTTACAAACAACATTTTTTTGCGCCTGGATACTCCTATATATGAGAACTATAATACGGTATATTAATCCTATTACCAACATTGTCAATAATAGTATGTAGGCAATGACATACTTTAAATACCAAATATCCATGGTTATTTCTAAAAATCTTGAAAAAACGTTAAATTTTAGATCGGTCACCTACGACAGTAATACTAATTTTAATAATTGATGACTGAAATCATAATATAATGCCGTGCGAAAAATAATTATTTTTCGGTTAAAGATACCATTACATAAAAAATATGCCATCTACTCTACAAGTGCTTGCTAAAAAGGTATTGGCCTTAGGGGAGCATAAAGAAAATGAACATATATCTAGAGAATATTATTATCATATATTAAAGTGTTGCGGTTTATGGTGGCATGAAGCTCCGATTATACTTTGTTATGATGGGAGTGAGCAAATGATGATAAAGACTCCAATCTTTGAAGAAGGCATATTACTTAATACTGCATTAATGAAAGCTGTACAGGAGAATAATTATGAATTAATAAAGTTGTTTACTGAATGGGGAGCAAACATCAATTATGGATTAATTTCCATTAATACCGAGCATGCCCGGGATCTATGTCGAAAATTAGGAGCTAAAGAAATGCTTGAAGGAAATGAATTTATACAAATTATATTCAAAACATTAGATGATACCACCAGTAGTAATATAATTTTATGTCATGAATTATTCACCAACAATCCTCTTTTAGAGAATGTAAATATGGGGGAAATGAGGATGATAATTTATTGGAGGATGAAAAATTTAACGAACCTATTATTAAATAATGACTCTATTAGTGAAATATTAACTAAATTCTGGTATGGTATAGCAGTAAAATATAATCTTAAGGATGCGATCCAATATTTTTACCAGAGATTCATGGACTTCAACGAGTGGCGAGTAACATGTGCTCTTTCTTTTAATAATGTGAATGATCTTCATAAGATGTATATAACAGAGAAGGTTCATATGAATAATGACGAAATGATGAATCTAGCCTGCAGCATTCAAGACAGAAATTTATCAACCATTTACTATTGTTTTCTATTGGGGGGCTAACATCAATCAAGCAATGTTAACCTCAGTATTAAATTATAATATTTTTAACTTATTCTTTTGTATAGACTTAGGGGCTGATGCCTTTGAAGAGGGTAAGACCCTGGCGAAACAAAAGGGGTATAATGAAATAGTGGAAATCTTATCATTAGATATCATTTATAGTCCAAATACTGACTTCTCATCAAAAATAGAACCTGAACATATTAGTTCTTTGTTAAAAAACTTTTATCCAAAAAATCTGTTCGCTTTTGATCGTTGCAACCCCGGTTTATATTATTCTTAGAGGACCGCTACAAAAATTATTTTTTTTTCTTGATCAAAGCTCCAAAATAATTATTAGATTAAAGTCGCCTATAGCAGCAGCCCACTCCAAAAAAAGTATTTTATAGTACAAAAAACACGAAAAATAGTTTGCGGCCGGCGGCAAACTATTTGTTGTTGTCTAAAACTTAATGTTTTTTTAATATTTTTAAATGCAACCATGGATTGTTGGACTATCAGGGAGAAGAACTATAGCTACATCATATTGTCAATACTGGTAATACTATTAATATGGTATCTTATACTTAACTATTGTCGATCGAAAAAAAATGCAGTTACAAACAACATGCCGCCACCATACACGGTGTCAAGTAGCTGTTCTCAATAATAGGGTTGATTGACGCTCTTCGTAATAATATGTTGATTGACGCATCATAAAATGCTGTGGTTGATTAATATGTTGATTGTCGCCTACTTTATTATATAAGTAATGATTTTTGTATAAAATACGGGTTTGTGAGGGCTTTATTTTTTCTTATTAGAACAAAGCATGCAATTTAAGGCCTACAGCAAGAGTAATTTAACACCTACAACAGTAATTTTAAGGTCAGTAATAATGTTTAATTAAGGCCTGACCACTAAAACTTAAACGATTTTGTAAAAAAAAATGTCTACTCCACTTTCTCTACAGACTCTTGTTAAAAAAGTGCTGGCCACACAGCACATATCTAAAGAACACTACTTTATTTTGAAATATTGTGGTTTATGGTGGCATGAAGCGCCGATTACGATTTGCATTGATGAGGATAGCCAAATATTGATAAAATCGGCAAGCTTCAAAGAAGGCTTATCTTTAGATATCGCATTAATGAAAGTCGTGCAAGAAAATAACCATGATTTAATAGAGTTGTTTACCAAGTGGGGTGCAGATATCAACTCTAGCTTAGTTACTGTTAATACGGAGTATACCCGGAACCTTTGTCAGAAATTAGGCGCAAAGGAAGCTTTGAATGAAAGGGATATTTTACAAATATTTTATAAAACACGTCATCTTAAAACTAGCAGTAATATTATTTTATATAATGAATTGTTTTCTAATAATCTCCTTTTCCAAAATATAGAGAGATTGAGTTTAATAGTTTATAGGGGCTTGAAAAACTTATCAATCAACTTTATATTGGATGATATTTCATTTAGCGAAATGTTAACTAGATACTGGTATAGTATGGCGATATTATATAACCTTACTGAAGCCATCCAATATTTTTATCAACGATATAGGCATTTTAAAGATTGGCGGCTTATATGTGGGCTTTCTTTTAACAATTTGTCTGACCTTCATGAAGTATATAACTTAGAGAAGACGGATATAGACATTGATGAAATGATGAAGTTGACCTGTAGTACGTATGATGGTAATTATTCGACTATTTATTATTGTTTTATGTTGGGGGCTGACATCAATCGGGCAATGTTAACCTCGGTAATAAACTTTCATATTGGTAACTTGTTCCTTTGTATAGATTTAGGAGCTGATGCTTTCGAAGACAGCATGGAACTAGCAAAACAAAAGAATAATAATATATTAGTAGAAATATTATCATTTAAAAATTATTATAGTTCAAATACCTCTCTTTTATCAATAAAAACGACAGATCCGGAAAAAATTAATGCCTTATTAGATGAAGAAAAGTATGAGTCAAAAAATATGTTAATGTATGAAGAATTATCTCATTGATACAAAATTATTTTTTATAACAGAACTCTCTGATGGTGACAAATCTCCGATAGGAATATATGACGTAACATAATTATTTTTTTCGCCCAGAAAAAAATTATAAATGTTATTATTGCCAGCACTTTTATCAACTATACGTACAAAAAGGTGTTGACCAAAAAAATAATTTTTTTTCTTGATCAAAGTATGTAAACGCCCGCTTACAGCAAGGATCTTAAGTGAGAGCCATTAAATTTTATTGATAGCTGCTTGCCACCAGTAGAATACGGCCAAACCACCTAACAGGAAATACAAGGCGGCCCTTCGGCCAATAAGGTGGATAAAAATCACGCATAAGACGGTTGTAACATAGCACTTTAGTGCGAATATCAGGAATGCCAATAGCATGTAGATAAGGCACCAAACATCGCAGCTATACATGGCTAAAGATCAACCAGAAAAGGTTTAAATTTTAACGCCGGCCCAAAACTTAAACTTTTTTTGATATTTTTAAGTGCAGCCATGGATTGGTCCGGCCATAGGATGACCTATGCCTACGTGGCATTCTCATTGATGGCAATAGCAATAATATGGTATATTCTACTTATCTATTGCCGATCGAAAAAAAATGTTGTTACAAGCGGTAATACGCTCGCTTTAGCGCCAATATCGCATATGTGAAAAATGTTCGCCGAAAAAAACATTAAAATTTAGAACCGCCGCGGCATCTCAGGGGCGGCAACATTTTTTTTTATATGGATATTGTCACACACCACCTCATCTATGACGCAATATATTACTGCTAATATCAGGTTCCCCAATAGTATGTAGAGAAACCACACAAGATAGATATTCATGGCGATTTTTGACGAAAAAACATTAAGTTTTAGCTTCTTTGACGCCTGTGTACTAATAATGTTTAACGCCTGTAGTATAATAATTGATACCTACAGCAGTAATTGATACCTACGGCGATAATGTCTCTCTGGCCGCCCCAAAAAAAAGTATTTACGGTAGGGTTTATTACCGGCGGCGTAACACCAGTTATGGTCAATTTTGTCTGGCCCGCCGCCCAGCCGCAAAAAAAAAATCAATTACAACCGCAAAAAAAAATATTTCCGGCCGCGGCGTTTCAAAAAATAATCTTTGCGAAATAATTCCGCATCTTGTGAAATGAACGCCTACAGTAATAATTTTAATCTTTGACACCTACAGCAGTAGTAATAATTTTAATCTTTAACGCCTGCAGCAGTACTAATATTTTTAATCTTTAACGCCTACAGCAGTAGTAATAATTTTAATGTTTAACGCCTACAGCAGTAGTAATAATTTTAATCTTTGACGCCTACAGCAGTAGTAATAATTTTAATGTTTAACGCCTACAGCAGTACAATAATTTTAATGTTTAACGCCTGCAGCAGTACTAATATTTTTAATCTTTAACGCCTGCAGCAGTACTAATATTTTTAATCTTTAACGCCTACAGCAGTAGT

**Complete genome sequence of ASFV‑ΔM448R:**

TGCTGTAGGCGTTAAACATTAAAATTATTACTACTGCTGTAGGCGTTAAACATTATTACTGCTGTAGGCGTAGCTAAATTTTAATGTTTTTTCGTAAAAAATCGCCAGTCACAAAAAAAAGCAGCCATGTATATCTGTGATGTTTGGTGTTTTATCTACATGCTATTGGCATTCCTGATATTCATGTTAAAGTGCTATACTATAACCATCTTATGTGGGCTCTTTATACATCTTATTGGCAGGAGGCCCACTATATATATCCTGTTAAGTAGTTTAGCTGTATACTATTTAATGTTTTTCGCTTGATCAAGAAATATACAAAAATCTAAGTGCACCATTTTATGGGCATCAATCAATCACAGCATTCTATGGGCGTGAATCAATCACACCATTTTATGGGCGTCAATCAATCACACCATTTTATGGGCATCAATCAATCACAGCATTCTATGGGCGTGAATCAACACATTATTGGGGACAGCTACCTGATATCGTGTATGGTGGTGGCACCGTGTTGTTTGAAGGCGTGTATGGTATTAGCGCCATGTTGTTTGAAGGCAGCGCGTTAGTAACAACGTTTTTTTTCGTATGACAACAGATATGTATAAGATAGCAAATTAATGCTATTGTCATCAATGAGAAACATAAGTAGATATAGACCTGCCTCTTGGTAGTCCAATGATCCATGTTGCACCTAAAAATCCCAAAAAAAAACATTAAGTTTTGGAGGGTAAGATTGGTTTTTCACCATTGGTAAAGATTATTATTCTAAATGTTTACCCCATAGATGTGAAACAATGATTCTTCATATATTAACATATTTTTTGACTTATACTTTTCTTCATCTAGTAAGGCGTTAATTTTTTCCGGATCTGTCGTTTTTATTGATAAAAGAGAAGAGTCTGGACTGTAATTTTTAAATAATAAGATATTTATTAATATCCAATTATTCGTTTGACTCGCTATTTCCATGCTCTCTTCAAACGCATCAGCTCCTAAATCTATACAAAGGAATAAGTTACCTTCACAAAAATTCATTACCGAGGTAATCATTGCCCGATTAATGTCAGCTCCCAACATACAACAATAATATATAGTTGTATAATTACAATCATACATACAGGCCAACTGCATCATTTCATCAATGTCTATATTCGTCTTCTCTTTGTTATAAATTTCATGAAGGTCAAAGACGTTGTTATAAGCAACCCCACATATTAACCGCCAATCTTTAAAATGACTATATCGTTGATAAAAATATTGGATGGCTTCAGTAAGCTTATATAGTATCGCCATACTATACCAATACCTAGTTAGCATTTCGTTGAATGAAATATTATCCAATGTAAAGTTGATTGATAATGTATCTAGTTCACCAAAAATTCTTAATTTCAGTTGAGCATTATTTAGGAATAGGGGATTATCAGATAATAATTCATGGCATAGAATAATATTACTGCTAGTTTTAACATACTGTACATTATAAAATATTTCTAAAATTTTATTTTCACTCAAAGCTTTCCTCGCACCTAACTTTTGGCATAGGTCCTGGGTGCACTCCATATTGACAGTAACCAACCCAAAGCTGATGTCTGCACCCCATTCGGTAAACAGCTCTATTAAACCATGATTGTTTTCCTGTACAGCCTTCATTAATGCAACATTTAATGTTAAACCATGTTTAAAACTTGCTGTTTTTATTAATATTTGTTTATCTATACAAGTATGATGAATCGTAATTGGGGCTTCATGCCACCACAAACCACAACGCTCTAAAATACAATAATCATCTTTGAACACAGGCTGTGTAGCTAGTACTTTTTTAGTAAGTGCTTGTAAAGTAGATGGCATCTTCTATCTGCAAAATAATTATTTCCGAAAAAAAAATCAAATTAAAATACTAAATTCTATTTTTTTTTAATAAAGCCTGTAAATTATATAATAAATCTCGCCCACCGTATTATTTTCGGACACAACTTTTTATACCTCATTATATTTTTAGATCTATAGTTTTTTAACAAGGCATTAATTTTTTCTGGATCTGTCGTTTTTAAAGATAAAAGAGAGACGTTTGAATTATAAAAATCTTTAAATGATAATATTTCTACTAATATATCATGATTCTTTTGTTTTGCTAATTCTAAGCTCTCTTCGAAAGCATTAGCTCCTAAATCTATACAAAAGAACAAGTTATTAGTATAAAAGTTTTTTACCGAAGTAACCATTGCCCGATTGATGTTAGCCCCTAATACAAAACAATAGAAAATGGTTAAAAAATTGCTATCTCTCATACAGGCCAGATACATCATTTCATCAATATTCATATCAACCTTTTTTATATGATATATTTCATGAAGATCAGACACGTTATTAAAAGAAAGCCCACATATTAGCCGCCAATCTTTAAAATGCCTATAACGTTGATAAAAATATTGGATAGCTTCAGTTAGGTGATATCGTACCGCCATACTATACCATAACCTAGCTAGCATTTCGTTCAATGTTATTTCATTCAATATAAAGTTGATCGATATCTTCTCTAGAAAACAACAAATTATTATTTTTAATTCCTCTATATTCTGGAAAAGGGGATTATTAGATAACAATTTATGGCATAGAATAATATTACTACTAGTTTTAATACGATGTATTTTATAAAATATTTGTACAATATCCATTTCATTCAAAATTTTTGCGCCTAACTCCCGGCAGAAATTCCAAGTATGCTCCGTATTGACAGTGACTAAGCTAGAGTTGATGTCTGCACCCCATTCGGTAAACAACTCTATTAGATCATAGTTGTTTTCCTGCACAGCTTTCATTAATGCGAGATTTAACTCTAAACCATCTTTAAAAATTGCTGATTTTATCATCAATTGATTATCCTCATTAGTAGAAAACATAATTGGAGCTCCATGCCACCACAAACCACAATATTTCAAAATAAAGTAGTGATTTTTGGATATGTGCTGTGTGGCCAGTATTTTTTTAGCAAGAGCCTGCAGAGAAAGTGGAGTAGACATTTTTTTTTGCAAAATGGTTTAAGTTTTAATGTTTTCAAGAATACAGATTGGATAAATTAGGTTGACATTAGTAACAGAAAGTATTCAATATTATGTAGACCTATAAAAAATGAGACCCTCCAAAAAATAATAAACAACACAAAAAAAATATGTTTAATATTAAAATGACAATTTCTACATTGCTTATTGCTCTTATTATACTAGTTATTATTATTTTAGTCGTATTTTTATATTATAAAAAACAACAACCACCAAAAAAGGTCTGTAAAGTAGATAAAGATTGTGGTAGTGGAGAGCATTGTGTTCGTGGAACATGCAGCACATTGAGCTGCTTAGACGCTGTAAAAATGGACAAACGAAATATTAAGATAGATTCTAAGATTTCCTCATGTGAATTCACTCCCAATTTTTACCGTTTTACGGATACTGCTGCCGATGAGCAGCAAGAATTTGGAAAAACACGGCATCCTATAAAAATAACTCCATCTCCAAGTGAATCCCATAGCCCCCAAGAGGTGTGTGAAAAATATTGTTCATGGGGAACCGATGACTGTACAGGTTGGGAATATGTTGGTGATGAAAAGGAGGGAACATGTTATGTATATAATAATCCACATCACCCGGTTCTTAAATATGGTAAGGATCACATCATAGCCTTACCTAGAAATCATAAACATGCATAAATAAATACATTAGGCTCATCGTATCCTTTTTAAAATCCATAAATATTCGTTTGATATATGCTGAAATTTTTATAAAAAAAAATAACTATTTCTTATAAATCATCTAGAAATAGTCCTCGTTTTGATCGGTTTATATCTTATAATATTGTGCATCGATGCACAACTGCTTTTTTTGGTCCTTCTGGAACATCATTATATTTTCTTTCATTAATATACCATTCAGATGTAAATGTTGAATAATTTTTATGGCAACAATCTACCACTGAATTATATTTAGTAACATCTAATACATCGCTTGTTTTATCAGGCTCAGCTTTATAATCTTGATAATTTTTGTTATCAGCTTCTAAAGCTCCATCATTATTTTTCAAAGAAGTGTCCATAATCATGTTTGGTAAAAACACTTTAAGTTTTAATATGATATTTAAAATGGTTGTTATATAAATTTACCGCTTACAGGTAATCTTTATTCAGTATCATAAACTATACTTTTGATGGTTCAGTATTCTGTAAATCGGTATTTTGTGATTCAGTATTCTGTAAATCGGTATTTTGTGAATCAGTACATTTATTATCATTAATATTTTTAGGCTGATTTTCCAATGTTTTATTGTTGCAATGAGCCTGCTCCTCCTTTAACGGGGAAGTGTCTGTTGGAGTCATCTGTTTAGGAAGAGTATCATCCATATCTATTATGAAGAAAATATATAAATATTGATATACAATCAAAAATATTTTTGATCACGTCTTTGTTATCTATCGATATTGTTGATAACGTCTTGAATAACCTACATCATTTTTTTACATAAAAAAATAGATATAATTTTTATTATATCTCAATTATCTTAAGATAATTATCAAAGTAACTATTTTAAAGATAATTATCAATACAACAAATATCATAAGCTAACATATTTTTCGAATAATAGTTTTTTAGTAAAGTATTAATCTTTTCAGGATTAGTTTCTTTTGATAATAAGATAGGATTCGCTTTATAAATTTTTAAAGATAATATATTCACAATGATAGAATAACCGTATATATCTGCTAATGTCTTACTGTGTTCAATAACATTAGCCCCTAAATCCATACAAAAGAACATATTTTCAATACAAAAGTTTTTTACCGAGATTAACATTGCTCGATTAGCGTTGGCTCCCAATGCAAAACAGTAGTAAATGGTCAAAAAATTATTGTCGCGCATACAGGCCAGCTCCATCATTTTATTAATACTCATATGAATTTTCGTTGTGTTACATATTTCATGAAGGTCAAACACATTGTTGAAAGAAAGTGCACAAATTAATCGCCATTCATCAAAATGCCTGTATTCTTGACAGAAATATTGAATAGCTTCTTTAAGATTATATTTTACCGCTATGCCATACCAATATTTGGTTAGCATCTCACTAAATGAGATCTCATTTAACATAGAATTTGTTGGTAAATCCTTCAACTCCCAATAAATAATCATCCTTAAATCCACCATGTTTACATTTTGTAAAAAAGGGTTATTAGAAAATAATTCATGACACAAAATGACATTACTACTTGTTATTTTACACTTTGTTTCAAAGAAAAATCGTAAAATTTCACTTGTCTCAAGCTCTTCTTTAGCCCCCAATTTTCGGCATAGGTTTCGAGTATGCTCGTTATTAATAAAAAGTAACCCATAATTAATATTTGCGCCCCATTCAGTAAACAACATGATTAGATCTTCATTGTTTTCCTTAACTGCCAATACCAATGCAGTATTAAGCTTTATACCCTCTTTAAAGCATAATGTCCTTATCATTATTTGATTATCATCATCTATATAAATTGAGATAGGAGCTTCATGCCACCATAAACCACAACGCTCTAAAATATAATAATCATCTTTAGATACGTGTTGCGTGGCCAATGCCCTTTTAGCAAGTGCTTGTAAAGTCGATGGCTGCATGTTTATTTTGTTAAAAAAAATCAAATTATCGGGTAAACATAAGGATCAACCCGTAGTTAATATTTGCAGTGACATTTTTAACAATGAATTATGATAAAAAAATAATTCATGACTATCTATCATGGAACCCATCTTTAACATTAAAGAAAAACCAGATCATCATTTTTTTTTGTTGTATTAGGACTTCTTCAAATTATTACCCACATTTTATCTAAAAAAATAAAACTACATAATATCTTGTTTCTTCATCAAATTATCATACCATTTAAAGTGTGTAGGTTGGGAACATTCCATAATATGGGTATCAAAGTGTTTATATATTTTTTCATAGGAACATTTATTTGGCAGATGTGTTGTCCAACAATCATATCTAATAAAATCATTTTCACCTATGGGGGGATGATTCTTAAAAGCCTTATTCTTACAGATGCCATTTTGACAGTCCCAGCAAAAGTTACAATATTTTCCATGAGTACACCAATGTTCAAGCTCTCTTTTGGGAGGAATGCTGCCAATTTTATGTTTTTTATCTTCTAACTCTCCATACAGCATCAGTTGGGAAAGCAGAAAGGAGATTACCAGGAAGACCAGCAAATACATAACAGTCTGTAAACTACGTTTGCGAATGTAATTCACAGCTAAAATACATCCCGCAAGATAAAATCCATAGGTTAACAATTTCTGCCATTTTCGTATATCAGCCTCATGCCTTTCATGGTTTATGTGTTGTGGACATTCTGTTCGGTACGTTTTATGAGGCTTTATAGAAGTTATGTGGTAGATACAGAAATCATTGCGACGATAAATACTGCAGTTAGCTATATAGTCATTTTCAAGAATGGGAGAATGGTTTTTAAAAGCCTTGTTCCTACAGATACCATCTTGACAGTCCCAACAGAATCTACAATGGTTTGCATAAGTGCACCAGTATCCAAGCTCCTTTTCAGGTGGGGTACTTGTTCGTTCCAGGGGCTCTATCTCATATGTATAAAGAAGAGTTGGAATAGATAGTAGGGTGAATATTTGCAGACCAAGCATGGCTACTTGTGAACAAGTGGCTGCTCGTCAGCAAATAGCTGCTCGTCAGCAAATGGCTGCTCGTCAGCAAATGCTGCTTGTGGGTAAGCCAATAAATGGGCCATACCCTTGAAAGGAGAATTCAGTTTTGATAAAAAAAATAACGAGTTTTCTAATAACCCGGTCAAGCATTTATTAAATGAGTGGCATCAGATGTCTGTATCGTGCATTCTACTTGGAAAATGGGCCCATCTCTAATATATTTACACTGACGGTGAATCATACAGTGTTTCATGGGATAGCTATGCTCCTGTACAGAAGACATATCTTTAAGAACTTTATTCTTACAAAGACCATCTTGACAAGTCCAGCAAAACCGACAATTTTTCACATATTGACACCAGTATCTTAGCTCCTCTTCCAGGAGATTGTCGGTCGAAAAACCCTGTAGACTAGTTAGACCAGCTAGCAGCAAGCCGAGGTAACTAAAGAACCTCATTGTAGTGTTATATTACGAAAAAATATGTTAAAATTTGGAAAAAAAAGCCCTTTTTATAGATCTGGAAAAAAATTTTCACAAATCTAATTAAAATCCTCACAGATCTTCCTTTTCATAAATTTTCCTTCCCAATCGGTGTGGACGATTGTGAGGTACTGGATTAGAACAATCCATTACATGGTAATGTCCATTTCCTTCATCATATGTACACTGGTTATACCAGCGAGAAACCTCACAAGATGTCAAATAACTGTTCTCAATAATCCATGGCATGCTCCTATTCACCTTGTTCTTGCAAATTCCATGTTCACATTCCCAGCAAAACTTGCAGCTTTCCATGTAAGTGCACCAGTATCCAAGTTCTTCTTGTGGAGGATTATCCGTTGAACGAAGATGCCCTTCTGCCTGAGTAGGTAGTCCTAAGACCTGATTGGCCAGCAGGCCAAGAATTCCCAAGAAGATCACCAACATTGTTACGGCTGGCTGAACAGCTGGCTAAATAGCTGCTGAATAGCAAACCAAGTGACTCGCCCTCTCTACTCTTAATATGAAAATTTAAGATTCGGTCCAGCATTTTTCCCATGTTTTACAGGGAAAAGATATTTTTATTCTATGAATGCACATGGTTCCGCACATTAAAAAAATAAATTATTTAATTTTGGCTAGCCGGGGTAACTAAAAACTCATTGTAGTGTTATATTACGAAAAAACATGTTAAATTTTGGACAAAAAAGCCCTTTTTTATAGGTCTGGGAAAAAAAATCAGATCTAATTAAAAACCTCACAGATCTTCCTTTTCATAAATTTTCATTAACAATCGGTGTGGACGATTGTGAGGTACTGGATCAGAACAATCCATAACATGGTAATGCCCATTTCCTTCACTATATGTGCACTGGTTATACCAGCGAGAAACCTCACAAGATGTCAAATAACTGTTCTCAATAATCAATGGCATGCTCTCATTCACCTTGTTCTTGCAAATTCCATGTGCACATTCCCAGCAAAACTTGCAGCTTTCCATGTAAGTACACCAGTATCCAAGTTCTTCTTGTGGAGGATTATCCGTTGAACGAAGATGCCCTCCTGCCTGAGTAGGTAGTCCTAAGACCTGATTGGCCAGCAGGCCAATAACTCCCAGAATAATCACCAGCATTGTGCTCAACCAGCAACGGCTAGCAACGACTAGCAACTGACTAGCAATAGCTAGAAATGGCTAGCAACCAGTAGTGGCTAACACTCTACTCTTTATAAGAAAATTTAAAATTCGATTAGATTTTTTTGGATTGAAAAAGAACAAAACGCTTATATTCTTTTCCTAGCGAGAAAAAATAAGCTAGTTTAAGATATGATTTCCCTTACTAACGGTTTAATTTTTGGCAAGGGTATAAGGTAAAATACATTTAGCTGAAAAAAATAAGCTTATGGTGTATAAACCACCATAAGTTTATTTAATTAAAATGTTAAACTCTGTGATAAGACTGGAATCTTAGGCAGGCTTGATGTGGAGAACAGCATGAAATACAAGAGTGCCTGCTACATGAATAAGTTCTCTCAAACCGAGGATGGTCATACTCACATCTATGAAATCCTGGTCTAGGAGATTCATTTGATGCATGATGGCCGCACCCACACTTATGAGGCACTGAAGAACTAAAGGGTTTAATTCTGATCTGAATGGTGCTATATAGGATGATGGCAATCCATATCAAGATCAGAGCAATCAAAATCACCTCCTCAAGAAGCATGATGTAGCCTTAAATCTTAGGCTGCCTTAAATCTTAGACCGCTTTAAATCTTAAGCCGCCTTAAATCTTAGGCCCTCACTATCTTTAATGAAGAAGTTTAAATTTTGATCCCCCTTTTTCAAGGCCGATATAAAAGAAAAAATAAAGTTTATAACAATCTAATTCATAGGTCATCTCTTTCATAGATCTTCATGTATTCTCTATGTGGATAAGTATGGGATGTTGGATTTGCACAGTCCATTTCATGATCCAGATATTGTCTAGAACCTTCATAATATCTACATTCGCCATTCCAGCGGGAAACCGTGCAATTTATAATCCAGTCATTTTGATGAATAACTGGCCAATCTGCTTGAATCCTGTTTCGGCAGATACCGTGGACGCATTCCCAGCAAAAGTCACATTGATTTGCGTAGGTGCACCAATAAACCAGCTCATGTTTAGGAGGATAACGGGTTGGTAGTAGATCTTCTAATTTACGTATAGGTGCGGCCTGAAGGATAATACCCCCCCCCCCCCAGTAGTACTAGAATTAGCACCTTCATAGTGGCTACCCTATACTAGACCTCTAAGTTGAAGACAAAGAACTAAAATTTAGAGCCGTTTAATTATTACTAATAATGATATTTTATTGTCTATAATAGGATTCCAATTAAAAATGATGATTTTTAACAAGAAATAATTTTATAAAAAATTAATATATTTTGTAATAAACTTTATTTCTAATGACTGTTAAAATAAGGAAACTATCCTTAGTTAGTCGAGGAAGATGGTTAGGTTATTTCGCAATCCGATAAAATGTATATTTTATCGTAGGTCTCGTAAAATCCAGGAAAAAAAATTACGGAAGAGCTTAAAAAAGCTAAATTTTTATCATCCTCCAGAAGATTGTTGTCTAATATATCGTTTGCTAGAAAATGTTCCTGGGGGAACTTACTTTATTACAGAAAATATGACGAATGAGTTAATAATGGTCGTAAAGGATTCGGTGGATAAAAAAATTAAAAGCGTTAAATTATATCTTTATGGTAGTTATATTAAGATTAATCAGCATTATTATATTAATATTTATATGTATCTTATGAGATATACCCAAATTTATAAATATCCCTTAATTTGTTATAATAAATATTATAACATCTAAGTAAATATTCTTGGAATGGATTTTCTTATAGAATGATTACAGGATAGGTCAGCGACAAGATTAATAACAAATTTGCTATTTTAATATTTTTGTTAAATAAATGAACGTTAATGTTACCCGTTGCGGAAAAAAACAAATTTATAATTACACATCATTAATTACATGTCGTTTTAACTCTATGAACCATTCTAAATCTTTGGGTTGTGAACAATTCATGTTATGTTGATAGTGTATCCTGAAATGGGCTTCGTACATACACCGGTCATGCCACCGGGAAACTGTACAATTAACAATATAATCATTTTGAGTAATAATAGGGTGGTCACTAAACACTTTATTTTGCACATTCCATCTTTACAGGTCCAGCAGAAGTCACAGTGTTTTGCATAGGTGCACCAGAACTTGAGATCCCTTTCAGGAGGCCTACGCATTTGCATCGGATTATCTGTGGTAAGAGGTAGGTTCATTATTATGTTCGTCATCAAAATTCCTAAAAGAACATAGAGGCCAAGAAAGATAAGCAGTCTTGTAGCGGCTTGCATTCGCATTCGTGAATACTGTTTGCGAACATAGCCTATGAGGGCAATAGTAGCTATCATACAAAGGCAAGTATGTTTGATATTCTCAACGTCAATGACCCTATTCTCCTTTACTTGCATTAACTCATCAAACCAATCATAATATGTGGGATTTGTACAGCTCATGATGTGAAAGCGGCGTATCCTAGAGTCTGTAAAGTAGCTACATCTATTATTATAGCGGGAAACCCTACATATTTGTATGTAATCATTTTTTTTGATGAGAGGATGTTTTTCAAAAACCTTATTTTTGCAAATCCCGTGTCGACAATTCCAGCAGAAGTCGCACGATTTTGCATAGGTGCACCAATACTCAAGCTCTCCCTTTGGAGGTCTCCGGGTCATTGGTAACTCTTCTGTTCCTGGAAAGGGTTGGCTTTGAATGACCGGCTGCATGACCGCCAATACCAAAAGGAGCACAATCACCTTCATGGCTGCACCGTATAGATCGCGACTCAAAAGGTATGAAAACCTTACCCTCAATATAGAGTTTAAGTTTTAATCCTGATAATGTATCTGTTTATAAAAAAAAATTTTCACTCATGTATGAATTCTTATACGAATCATAATATGTAGGCTGAGAATAATTATATACGGTGTTGCAGGCTCATTAAAATTTTGTTACCACAAAAAATAAATGCTGGATCTTTAAGATATATATATCTATTAATGACTAAACCCTTTATATGCTGTAGGTTGAGAACAATCCATATAATGAATATAGGGTGATTTGGGTTTAATAAAATATATACAACGGTCAAAATAGCGGGCAATACTACATTGACTAATATAATCATTTTGTTTAATAAGAGGCATATCATCCCAAACTTTATTTTTACAAATACCATTCCTACATTCCCAGCAGAAATCACAGTGTTTTCCATACGTGCACCAGTATTCAAGCTCTCTTATAGGAGGTGTATAAGTCCTTGGTAAATTTTGTTTCATATAAAAGATGGAAAGGGGTCGGTTTAAACCCGGCTGAGATAGCCAAATCAAAATACATAAAAGAGCAAGTAGTTTCATAGCGGTATTTAGGTGTAAATTTTTATAGTACGCAAATACAATGTAACCTACAAATGCAATACTAAATACAAGGTAAAAACAACAATGTCTTATAATGGTTGGCCAATAATCACCCCCCCCCATTTTTCCATGAATATTTCATTTCCTGTATAGGGTCTAGGATGTGAACATTCCATGCTATGATGATTAGGTATTTTAACTGATATTTCATATAAACAGGATTGAGTCTTGGAATCACGAAAAACTCTACAGTTTACAATAGAATGATTGGAGTCAATGAAACGAGATTCCGTTATCTTATTTTTGCAAATGCCATCTTGACAGTCCCAACAGAAGTCGCATTGTGGTACATACGTACACCAATATGAAAGCTCACTCTTGGGAGGATGCTGGGTTCTTGGTAAGTCTGGTAATTCATGTGCGAGAATGAGGACTGAGTGGCCCAACAAAAGTCCCAGAAGAACCTTCATGTTGCGTCTAAATAACACCTGCACTTACAAAAAAAAAATTTAAATTTTGAATATAACACAAAAAACCACCTTAAAATTTCTTATATTATTTCTTGGATCTGCCCGACGTCATACAATGTATTAAAATTATAGACCAATCATCTTTGTATATAGATTTTAGTTATTTTGCTTGTTGTATCAACTTAACTGCTAGTGAAGAAAATGGATAAAAACTTTCTGTATTTTTATAGGTTGAAATCATTTTACGCACATCACTAGGATCTAATATTTTATTTTGAAGAACTGAATGTGGGCTTAAAATTTTTTTCTTAGAAAAGAGTAGAATCATAATATTGCTATGTTTTTGTTTAATGGTTTCTTGTATCTTTTTTGTATACGGGTTGGCACCCAAACCTATACAAAAATATACATTACTCAAATAACTACCTTCTATACATAATCTTGTTTCCCCACTTATTTTCCTATTTATTTCCCTATTTATGGAATTAAAGGATATCAATCTCTCTAAGGCACGGTCAAGGTCTGCGCCTAAGGCAAAACAATAATATATACCTAATTTATTCCCAGGGCGTGCACAGGCAAGAAACATCATGACGTTTAGAGATAAACGTATATTTTCCTGAAAATACGCATGATGAACTTCATCAATATTACCTAAGTATATGGCCGTTTGTAAACGCCAAAGATCTAAATGAGGAAATTTTTTACTAAGATAACGAATGGGTTTTGTAAGTTTAAAATCTATGGCGAACTTATACCAAAATTTTAATACAAGTGTATTTCTCGTCATTTCTTCTTCTTTTTCATCTAAATATAAGATAAAACGATTGTAAACAAAGTCTATCAACACGTGAAAATCATGGCTGTCAAAACTGTCGAGAATCGAAATATTGTCATAATAAATATCAATCGCCAGTAAAACCTTTTTTTGTTTGATAAGATAAACAAACATATTATACAACCCTACATCTAAAAATTCTGGATCGGCTCCTAGTTGGATACACAGGTCTTTAGTCTGCTTCGTTTTGGCACACATAATGCCAAAATTAATTTCAGCACCCCATGAAAGAAATATCTTGATTAGATCAGTCTGGTTTTCCTTCACAGCTTTTACTAAGGCTCTGTCAAGCTCATAACTGTTGACATCAGAGCATGACATAGAGCCATCGGTTACCATTTTACATTGCTTACAAAAACCTATGGGTCCGTTTTCCCACCATAGTCCAAGCTGTTGTAGAATAAAAATATCATCCTCATGATACTTTGAAAAAGCCTTGGTTTCTATCAAGACTTTTTTAGTAAGAACCTGTAAAGAGTTCATCGTATTATTATGAATAACAGGAGTAAACGTAATCAATTATAAAAAGTGACTTTTTTGAAAAATCTTCAAATGGTTGAAAACGATAATGTACATGTTCATACAAAAAATATATTCAGTGATGTCCAAAATCAAAATTTAATTTTCTATGTAAAAAGTACAGACGTTGCCTATTCGGGTTAAATTGTTTATTTTAAACTTTAATTAACCATTTGAGTTAGCGATGTTTGATTTATCTTCCATGCTCATCCGGGGGGAGTTAACGATGTTTAATATTTCTTCCATGCTTATCAAAGGGGGGGGGTCTTAATCGTTCTAATACTATTGTTGTGGATTGTTGAACATAATGAAAACTTTATAGATGCTAAAATAATTGAATCTAAAAATAGTAGTATACTTTAATCAAAATTTGATTTCTCATAAAAAAGGTACACATCATTCATTTAAGTTTCATGATGTTTAGTTCATTACTTCCTACAATTACTGGGGGGGGTCCCTCATAGCTTTAGTATTATTGTGGCTTACTGACTATTATGTAGAATTCATAGAAGCAACAATATATGTTACTAATGCTACATTGTAAATTATAAAATACATACTAAACTAATTTCAGTATTTTTTTTTGTTCATATAAGTTAAGGTACAAAAATGATTAAACACTGCAAAAAAAGAAAATCATAATGCTATTATACATAGTGATCATAGTGGCTTGTATCATTTCTAAACTAGTTCCAAATGAATATTGGGCAATACATCTATTTTTCATCATTATGATTTTTATGGTATATATGTATGAAAAGTTAGATATACATCAAAAATATCAGTTCTGGAATTATACGATGTCAGGCTTATCTGGACATAACGTACAGGTAATATGTAAGTGTTACTAAATACTATGAAGTATCTATTTTTTTTACAACAAAAAAAATAGATGTAATCGGTTTTATCATTTAGGTGTGTATTTCTTTAGCATCTTTCAAGAATTCATTGTTTAGCGGTATAAAAACAATGAATGATCATCATATTCTATTTAACAATCTCCTAAATAAATGAACATCTTTTTCATTTTAACATGATCACCAATAGTCATTTTGCGAAAAGGCATACATATGATCAATATCAGACCTACAATGAATGTTTCCATAATATCCCTTTATCGCAATAATTCTATTTTTGCATTCCGATATCTCATCATCTGTGCTATTATATGTTTCCATAACTGTTTCATCATCAAACATAAATCCTATTAAATAAGCAAAGCGCTTTAATCCCGGATAGATTTTCACCATTTTTCTAAGAGCCGTGTATAGCTTGTAATAAATGGCCAGAAATATGCAATAAAGCGTAGAAAGAGAGTAATTTTTGGCATAAAAGGTTTTGAAGGTTTGATGAATGGCTAAATCGCAGATAATATAAGATACGATATTAAAGCGCACCTGTTCACGCAGATTTGTTGAAAACTTCGTAGAAAGATTCAACAAATAAAAGGTTATTAATAGTTGCTCATCATTCTCCTTATACGACATCGTCAGACGCTCTAAGATTTTACTACTGGACACATCTGCCACATGTTGAACATTTAAAGCCTGTTCTTCTTCTGTGTTACGACAAAAGAGCCGCGCATATTCAGGTGAAGCTCCCCAGGATAACAACGTCCGTGTCACGGCTAAATTTTTTTTGACGATGACTTTCATCAGAAATAAGTCTTTATTTTTGCATTGATCACTATGCGAATTTGTATAGTTGACGCCGTTGCATTGAGTACATTGATATAATGTTTTGCAATTCCAGCGTAGCCCTAAATGGTATAAAAGGACTGTATTTTCGACGCAAGCATGCTGATTAACGATGTTTTTGAGACAACACGTTGTTAAGGACACCATATTGTCTCCAATTTGTTGGATAAAAGTCTTTACTAAAAAAATAGGTTTTTAGTTTTAACAATCGAGATTTTATTATTTGGATATAGCATCATCAAAAAGATTTATGTTAAAATTTAATTTGTCTTACAAAAAGTACAGATTCAATAATTGGTTTAACTATTTAGTTTAACTATTTAGTTAAATTACTTGGTTAGCAATGTTTGAGCTTTCTTCCATTCTTATTCGAGGGGGGGGGGTCCTAATAGTTTTGATACTTTTGTTGTGGATTGTTGATGAAAACTGTACAGATGCTAAAGCAATGGCATATAATATTAATTGTACTGTTTAATGTGAGTTTATAAGTATGTATCAAAATTTATTTTTTTTTATAAAAGTACAGAAATTAATGTTAGCTTAAACTTTAGTTTAGCTATTTGAGTTAATGATGTTTAACCTATCTTCCATGCTTATCAGGGGGGGGGTCCTAATTGTTTTACGTTATAAATTTGATCAAAATTTAATTTCTTATAAAAAAGGTACACATCAACATTGCTCATTTAAGTTTCATAATGTTTGATTCATTACTTCCTACAATTACTGGGGGGGGGGTCTTTAATAGCATTAATATTATTATGGTTTGCTGACTATTTGGAATTTATAGAAGCAAAAATAGATACTTTTACTAATAGCAGTGCATTATAAGTGATCAAAATTTAATATCTCATAAAAAAGGTACACATCAACATCATTCATTTGTTTCATGATGTTTGATTCATTACTTTCTACAATCACTGGGGGGGGGTCTCTCATAGCTTTAGTATTGCTATGGTTTACTAATTATTATGTAGAATTTATAGGAGCAGATATATATGGTGGTAATTGTACTCTATAAGTTTATAAAAATTTCAGTATATTTTTTTTGTTAATATAACAGAAATGATTTAAATTACAAAAAAAAGAGAATCATCATGCTATTATACATTGTTATTATAGTAGCATATGTAAGTTATAAATTGGTTCCAAAACAATATTGGCCAATACTCATGTTTATGGCTTACATGGTATATACCCATGAAAAATTAGATATAAATGAAAGATCCGGATTTTGGAAATATATTATAGCAAAACTATTCAGATGCCACGGATGTGAAATATGTAAGTGATATTAAAAACATAATACGAAGTGTAATAATTGACTATTTTTTGTTGAATGGAAACTGTTTTTGAGTAATATGTTGAACATAATGGTTTTTATAGATGTTAAATCATTTGATTGTAGTAATTGTACTAAATGATGTGAGATATAAACCTAGTTCAATCAAAATTTAATTTTTTTATAAAAAGCTACAGAGTGTTTTATTAAACGTAGCTTATTTAAAAAGTTACACAATGTTAAAATCTCTACTTACTTTAATTCTTTGTGGGGTTTTATTAACTTTATCCATATTATGGCTTATTACTTACCATGTAGAACTTATAGAAGCAATAGATGATTTCTACGACTGAAATATAGAATAGTTCATTTTCTATTTGTAAAATAATGATTTATATTCTCTCCTAAAAATACGATCGACCCTTTATATGCTTTGAAAACGAATATTAACAACTTGATTTTTTTTTCTATAAATAAACTATAAATGAAAATAGTAAAACTCATAGAGTCTTATAAGTGAACATCCATCATGTTACTCAAACGTTGGACTATTAAAAGATACTCCGTGTGCATTATTGCTTTTAATCAGTATGATTACTTTATACGAAGCCGCTATTAAAACGCTTATCACACACCGAAAACAGATTTTAAAACACCCCGATAGCCGTGAAATTTTACTAGCTTTGGGGTTGTACTGGGATAAAACTCATATTCTTGTTAAATGTCGTGAATGTGGGAAAATGAGTCTTACCGGAAAACACAGTACAAAATGTATTAACATTAATTGTCTACTTATTCTTGCCATAAAAAAAAAGAATAAGCGTATGGTTGATACCTTGATAAGAATGGGTGCGGATGTAACATATATACATCTTTTAAAGAATAAGATAAAACTGTCATACAACCAGCTGTCTATGCTTAAAAGCAACTCGCAGATTTCGTTGAAGGAGCTTCATGCTATATGCTACCTTTTATATGGTCGGCTTCCCAAAAAAATTAAACAAGGGATGCAACTGTGTAAAACAATGGCGGGACTATGTGGTGAACTTTTATGTGCATTTTTAGCTCCGTAAATGTAATATATGTATATAAAACAAATAGATAATACCAAAATATATTCTATGTACATAACATCCGGAAAATTATTTTTTTTTCTCATACCCTTAAATATAAAAATATTGGGTTTCTTCACTAAACTTTAGAGGTAAAAATTTTTCTTTGTTTTGCATCATCATGTATGGGTTTAGGTTATCCCAGGGATTGTTTATTTGAATATTTCCTAAATAGGAACACAACGCCATGATCATATATCTTTCATTCTGGTAAGCTTTTTGATACATCTTCAAAGATGCCGTACCTCCGAGTGTGTAACAGCAAACAAACGTCCGTACTTTTCCATGGGTCGCAGCCCATTCCATTCCGTAGCTCAGCATCCTTTGCTGTATTTTTTTATTCGCTTTATAAAAAAAGTATTTCATCCATTCCACGTTCTCATAAAAACAGGCACTTAAAAAGAGCACCAGGTTTAGTGTAGTCTTATTATAGAATGTAGGAATGTATGTTTTAGTTATTTTTTTCAACGCCTGTTTCATACCATGTTTTACCGCCATAAAAATACAAAACCAATACCAACTTTTTCTATAAAAGGTTTTGCTGTACACATGTAAACGAGCAAAATATATTTCAAACTCTAAATTCTTTTTATAAAAAAACTCGAGACAGTCATTTATGTTACGACATCTTCTAAAAACCTCAAAAACAGTAATTAATTCACTGTCGCTGTAGAAATGTTCATAAGCTAACTGTTTAATGTCTTTAGGGGTCAATTCTTTTTTTGGGAGCAGTGGTTTGAGATTCGGCAAAGGTCGTCTAAAGTAGTGAGCGAACTTTTCATTCGCTCCCCAACACAAAAGCCGATAAGCTAGCATGTAGTTATCACATTTTACCGCGTAAATGAGTAAATAGTTTATATTGATACATGTACCATGTTGCTGTCTGTTTGGGCATATGTTGCCGCATTCTGAACACTTATGAATGAGATCATAGTTCTTACAACATAACCCCAAATGGGTTAATATGTCTTTGTCATGTTTTAAAAACTCGATATGATTCTTTAATGTTTTGAGCGCAATGTTAAATAAACTCAGCATTTTAATAAAATGAGGTGTTTTAGTATTATGTTTAGTATAAAATTTAGCGGTATGTTTGCATGATGCTAAATAAACTTAACGTTCCTACTAAACCAAAAAAAAATCAAATTGACTGACTCATAGAGAATTTGACGATGTTGGTAGGTAATTTTTTAACATGGTATATATTTTTTTAGGATCCGTTATATTAGGTAATAAAAGAGGACGTGCCGTTAAAGTATTTTGCTTAAGATCCTTTAAATCCTTACAAAAATATAGATTGTTCGTATGATAATGCCACTGTGTTACAGTGATGGCTTGATCAATATCAGCTCCCAAGACAAAACAGTAGTATATCGTTAAAAAGTTGTAATCTTGCATACAAGCCAACTGCATCATTTTATCGATGTCCATATGAACGATCTTTTGCTCGTATATTTCATGAAGGTCAAATACATTGTTGAAGTAAATGGCGCACATGAGTCGCCACATACTAAGGTGCCCATATGTTTGATAGAAAAAGAAGATAGCTCTTTTAAGCTTATATTTTACTGCTATGGCATACCAGTATTTAACGAATATGTTCATGGGTACATTATCCAAGATATAAAATATGAAAAGCTTTAACTCTCGATGAATCTCCTCTCCCATTTCCTGCACATTTAGAGCTTCCAACATAGGATTTTTATCAAATATTTCATGACATAAAATAATGTTATTACTCGTTTTATGACGCATTAAACCGGCGAAAATTTCCTTATTATTTAAACCATCTTTAGCTCCTAACTTTCGACACAGCTCCTGAGTTTGTTCCGTCCTAGCACAGGTCAGCCCATAATAAATGTTTGCTCCCCACTCGGTGAACAGCCTTATTACGTCATAGTTATTTTCTTTTATGGCCATGATTAATGCCACATCAAGATGAAGAAGTTCCCCCTTAAAGGGGGTTGAGCTTAAAATAACGTAATTACAGTAGTGACATAAGCTAATGGGCTTGTTTTGCCACCATAAGCCACAATATTTTAAAATATAATGATACTCCTCAGGCACGCTCTGTTTGGCCACAGCCTTTTTGGCCAGGGTTTGCAAGGAGAGCATGATAACTTCTTGAAAAAAAAACTCAAATTAAGTTCCTACTTTTTTAAAATATTAGTATGGACAGATCTACCATCATATGAAGGAATTCTTTCATCGTTAAACACTGAAGAGATAATACTTTCATCGTATAGAGAATATCATGTCAATCCATATATTGAATGTTATATATCATTAAACCCATCATTAATATAGTGTTTATGTGCTATGGACAGGTTTTTTGAATGATAATCTTTTAACATACGTTTTATAACTTCGGGATCAGTTTCTTTTAAAGATAAAGAATCATTCATGTTATAACAATTTAATGATAACATGCTGGCAATGAACGAGTTGTCTTTTTGATGCGCTAGAGTCTTTCCCTCCTCAAAGGCATTGGCGCCTAAGTCTATACAAAAGAATATGTTTCCGATATTATAGAACTGAATAGAATGAAACATGGCCTGATTGATATCAGCCCCTAAGACGACGCAACAGTAATAAATCGTTAAATAGTTATAGTTCTTGCGACAGGCCCACTTTAGCATTTCATTCATGTCTATGCGAATCCTCTCCTTTTCGTACACTTCGTGAAGTTCAAACACATTATTGTAAAAAAGGGCGCACATAAGCCGCCACCGATGTAGATGAGCATATCTCTGATAAAAATAGCAAATCGCCTCCTTAAGGTTACATTCTATTGCCATCGCGTACCAATATTTAGTAAACATCTCGCTTAATATATCGGTTTCTACCATTAATCCCTCCAGTTGTTCATAAATCATTCCCTTTACTTCAAAACGATTTATGGTATCTAAAATGGGATTATTAGAAAATACCTCATGGCAGAAAATGATGTTACTGCTAGTTAGATCACGTTTCAATGTGTAAAAAAATCGTAAAATTTCCTGGTCATTTAACTGTTCTTTGGCACCTAGCTGCCTGCACAGGTCTCGGGTGTGCTCCGTGTTGACAGAAAGCAAACCGTAGTTGATGTTTGCACCCCACTCGGTGAACAATTCTATTAGATCGTGATTGTTTTCCTCCACAGCTTTCACCAAGGCCGCGTTAAGATTTGTGCCGTTCTTAAAATACGGCGTCCATATTTTCTTTTGATGATACATGATAGGGCCATTATGCCACCATAGACCGCAGCACTTCAAAAAATGAGGATGGCATTTGGCCGGATACTGGCTGGCCAGCACCTTTTTGGTGAGAGTCTGCAGAGAGAGGACCATATTTCTTTTTTTTGAAAAAATCAAATTAAAAAAATCATGCTTGTTTAGCATACATGTAATATTGTTATAATTACGTTATAATTACGTTATAATTACGTTATAACTATATTATAACAATGGTATAACAATGGTATAACAATGTTATAACAATGTTATAACGATGTATCATTGATGTCATCATTCAACTAGGCCAACATACTTTTTAATTTATAGTTTTTTAATAGATGATATATTTTGCTAGGATCTGCTTCTTTTAACGTTAATAGCGAGGAGTCTGCACTATAAATGTCTAATGATAAATGATGAGATATCAAATAGTAATTCCGTTGCTCTGCTAGGGCCTTTGCCTCTTCAAAGGCGTCGGCTCCCAGATCTATACAAAAGAACAAGTTATCCATATTATAAAATCGTACGCAGGCAAGCATAGCTGAATTAATATTAGCTCCTAAGAGAAAACAATAATATATGGTTAAAAAATTGTTATCTTTTGTGCAGGCCATCCGCATCATTTCATCCACGTCCATGCGGATCTTTTCCTTTTCATACAAATTATGTAGGTCAAACAGCTTATTAAAACAAAGAGCACAGATTAACCACCACGTATTTAGATACTTAAAATGTTGGTAAACATAAGAAATGGCCTCCCTAAGATTATCCTGCAATGCCACTATAAAACAGTATATCGTTAACATATCACCATCCGACATATTACTTAATATGTCGGTGTCTTCTACTAACCTTTTCAACTTCCAATATATGGATGACCTTATTTCCCTTATAATGACATAGGCTGGAAAGGGATTATCATTAAAAAGTTTAAGACATAAGATAATATTACTGCTAGTAGTGCCAGGGTGTATTAATTTAAAGAACATGTGCATAATCTTCTTTTTATCCACGCGGTACTTGGCTCCTAATTCCCAGCAAAATTCTCGAACAGGCGGCGTATTGGCGCAAATTAACCCATAGTTGATGTCTGCGCCCCATTCTGTAAACAGTTTTATTAACTGATAGTTGTTTTCCTTTGTAGCCAACATTAGTGCCGTATTAAGGTCCAAGCCGTCTGCAAAGCTTGGCAGCTTTATCAGCATATGTTTGCAATCAAGGGAAATTGGGGCCTTATACCACCATAGTCCGCAGCGTTCTAAGATAACATGGTACTCAATAGATACTTGCTGTCTGGCTAGTACCTTTTTGGCGAAGGATTGTAAGGAAGGAAACATCCTGTTTCTTTTTTTTTTAAAAATCAATTATCTTTGTTCATAATCAAGAAAAATCCCCATATTTATTGAGTGATAATTTTTTAACATGCAATTTATTTTTTCAGGGTCCGTAACGATCGACAACAGAGAAATAACCGGATTGTAATGCTTTAATGATAAGGCATGGGCTATCAGATAATTTTCCTTTTGTTCTGCCAAAGCTTTGCCCTCCTCAAAGGCATCGGCACCCAGGTCTATACAAAAGAACAGGTTTCCAAGATTATAGTTTTGTATGGAAACAAGCATGGCTTGATTGATGTTGGCTCCCATGATAAAACAGTAGTAAATGGCCGAATAGCTATAATCTTGGATGCAGGCTATGTGCATCATTTCATCAATATCCATGCGGACCCTTTCTATTTCGTACAGCTCGTGAAGGTCGAACACGTTGTTGTAAAAAAGGGCGCACATGAGCCGCCACCTATGTAGACGCGGGTATTTCTGGTAAAAGTAGCGGATAGCATCTTTGAGGTCATAGTCCACCGCTATCGCGTACCAGTATTTGGTTAAAACAGTGCTAAAGCTATCATCATGGTCCAGCATGAAGGTTATCTCCATGAGCCCTCTTAACTCCCACATGATTTCCCCCCTCAGATCCAGATTATCTATAATCCTTAAATTGGGGTTATTGGAAAACACCTCGTGGCAAAAGATAATATTGCTACTGGTTTTATCGCGCGTTGTATCAAAGAAAATTTTTAAAATATACTCTCTTTCTAAATATTCTTTGGCTCCCAGCTCTTTGCACAGATCACGGGTATTTTCCGTGAGAGCACAAATCATTCCATAGTTAATATCTGCACCCCATTCAGTAAACAGCTTTATCAAGTCATGATTATTCTCCTTCACGGCTTTCATCAGTCCTATGTTTAACTCGATACCTTGACTAAAACAGGTTGACCTTATAAATAATTTATTGCGTCGAATATGAAGCATAATGGGGCCATTATGCCACCACAGGCCACAACACTTCAGGACATGATATTGATCTACCGGTATACACTGCCCGGCCAGTACTTTCTTCGTGAGGGATTGCAGGGAAGGCAACATGCCTTTCCATCCTTTGACGGAAATCAAATTATCTACTAATAACTATCAGTGTTTATATTAAGTATTTAGATATTATCCCGGGCTGGATACGTAGTATCGCTATTCACATGTACTTCCAACTCTAGCCGGAGCCTGCAGGGTCATTTATTTTTAATATTGATTCTTTTTTGTATTTAATCATTTAGAGAAGGTCATCATAGGAGCCAGATGTTCTCTCTCCAGAACTTATGTCGAAAAACATTACCTAACCGTAAACTTCCTGAATTTTTTGACGAATATATATTACAACTGCTGGGATTATACTGGGAAAACCATGGAACTATTCAACGAGCAGGAAACAACTGTGTGCTTATACAGCAACATACCCTCATTCCCGTAAATGAAGCCCTGAGAACAGCAGCATCTGAAGAAAATTATGAGATCGTGAGCCTTTTATTAGCGTGGGAGGGGAACCTTTACTATGCTATTATAGGGGCTCTAGAGGGCAACCGCCACGACTTAATTCGTAAATATGATGACCAAATCAAGGACCATCATGAAATTCTGCCATTCATTGACGATCCAGTCATATTTCACAAATGCCATATCATGCGGCAATGCTTTTTTGATTGTATTTTATATCAAGCTGTAAAATATAGTAAGTTTCGCGTTCTTCTTTACTTTAAACATAGATTAGAGGATGATTTGCCCTTCACTCATTTACTTATTGAAAAGGCATGTAAAGATCATAATTATGAAGTTATTAAATGGATATATGAAAACCTACATATCTACAATATGATAGATACCTTTGAATGTGCTATTGCCCATAAGGATCTACATCTATATTGTTTGGGGTATAGATTTATATATAACAGAATCGTACCCGATAAGTATCATCATTTAGATATTCGCATGCTTTCAAGCCTACAACTCCTACATAAGGTGGCAGCCAAAGGATACTTAGATTTTATCCTAGAAACCTTAAAGTATGATCATAATAAAGATAATATAAATATTATTCTAACACAAGCTGCAACCTATAACCATAGAAAAATTTTAATCTATTTCATTCCTCAATCAACCCACGCACAGATAGAACAATGTTTACTAGTGGCGATAAAAGCAAAATCTTCCAGGAAAACCTTGAACTTACTACTGTCTCACCTAAACCTTTCCATCAACCTCATCAAAAAAATAAGCCATTATGTTGCCACTTACAATTCAACAAATATAATAGGCATTCTGAGTATGCGGCGGAAAAAGAAGATATATTTAGATATCATATTGACAAAATTTGTAAAAAAAGCTATTTTTAATAAGTTTGTCGTTCGATGTATGGATACATTTTCTATAAACCCGGAAAGAATCCTTAAAATAGCCGCGCGAATAAATAGGATGATGTTAGTGAAAAAAATATCTGAACATGTTTGGAAAAATCATGCGGTTAGACTTAAATACCTTAAACATGCGGTACACACGATGAAGCATAAAGATGGGAAAAATAGACTCATGAACTTTATCTATGATCGCTGTTATTACCATATGCAAGGGGAAGAAATCTTTAGCCTCGCAAGATTTTATGCAATCCATCATGCACCAAAGTTGTTTGACGTTTTTTATGATTGTTGTATCCTAGATACGATACGATTCAAAAGCCTTCTTTTAGATTGTTCACATATCATAGGTAAAAACGCTCATGATGCTACCAATATCAACATCGTGAACAAGTATATCGGCAACCTGTTTGTTATGGGAGTTCTTAGCAAAAAAGAAATCTTACAGGACTATCCATCCATTTATTCTAAACAATACATGCCTTAGTTTATTTTTTTTGCGGCCGAAACATTATTCTTACCCTAGAAAACGCTTATAGTCATCTTAAATCATAGGTAAGGAAGATCATCATATTTTTTGAAACGTAATTTTTTAACGCATGATCTATGATTTCAGGGTCCGTGCTTTTAGGCAACGGGGTGGTGGCCGGACTATAAATCTTTAGGGATAAAATGTTCTTTATAAGCTCATACCCTTCCCCTAAAGCTGTAGTACCCTCTTCGAAAACATCAGCCCCCAGATCTATACAAAAGAACATGTTTTCTATATTATAGTACTGTATTGAGCTAAGCATGGCTTGATTGATGTTGGCGCCCAGGACATAGCAGTAGTACATGGTTGAAAGGTTGTGGTCTTTGATGCAGGCGATCCGCATCATCTCTTCTATGTCCATATGGATCTTGTCCTTTTCATACGCCTCATGAAGGTCAAACACATTATTAAAACAAAGAGCACATGTTAACCGCCACGTATTCAGGTGTGTATATTTTTGGTAAAAATACTGTATGGCCTCTTTCAGGTTATAGCGTATGGCTATAGCGTACCAGTATTTGAGTAGTAATGTACTGAGCGAAAACTCATTATTTAGCAGATCGGTTTTTACTATTAACTCCCTTAACTCCCAGAAAATTTCTATCCTCATTTTTATATTATTTACTTTTTGTAATATCGGATTGTTGGAAAACACCTCATGGCATAAAATAATGTTACTACTAGTTTTATGAAACTTTAGATCTATAAAAATTTGTAAAATTTCTTCTTCATTCAAGGTTTCCTTGGCACCTAGCTCTCGACAGAGGTCCCAGGTGTGCTCCGTGTTGACAGATACCAGCCCGTAGTTGATGTCCGCCCCCCACTCTGCAAACAGTTTTATAAGGTTGTAGTTGTTTTCCCTTACAGCCTTCACTAACGCCGTATTTAGGTTTAAGCCCTCTTTAATACCTGCTGATTTTATGAGCCTTAGGTTATGATCAAACGTGATCGGAGCATCATGCCACCATAGGTCATAACACTTTAAAAGATAATGTTGGTTCGTGGGCACGCATTGTCCAGCCAACACCTTTTTGGTCAGAGATTGCAGGGAAGGCAACATGTCTCTTCATCTTTTAAAAAAAAATCAAATTAATTAGCCGAATAAATTTTTCTTTCGAGGGCTTTTTAAAAGAGCTCTTTAAGAGCTCTTTAAGAGCTTTTTAAGAGATTAAAAAATTATTCTTGCTGGCATTCTGCCAAGTATGCGGCATTCCTATCATCTATAGTATATTATGAGAATATTCCCAAATGATGGATAAGTTTTTTGATTTATAATCTTTTAATAAACTGCTTATTTCTTCGGGGTCCTTTAAGTTTAGTGGCAAGGAAGCATCTGAGCTGTAAATATCCAAAGCCAAACTATGGCTCAGAAAATTATAACCTTTTTGTTCCGCTATGGCACGACCCTCTTCAAAGGCATTACCACCCAAATCTATACAGAAAAATATATTACCGATGTTATAATATTGTACTGAAGTAAGCATAGCTTGGTTGATGTTGCCCCCCAGCGCGTAACAGTAATATATTGTTAATGGATTGTTATCCTTGGTAGAAGCCAGACATATCATGTCATGGACGTCTATTTGGATGTTTTCCTTGTGGTACATCTCATGAAGCTCATATATTTTGTTATAATACAGGAGACATTTTAATCGCCATTCATTAAGATCCGTATATTTCTCATCTAGAAAACAAATGGCGTCCTTACAATCGTATTGTACTGCTTTGGCGTACCAATACTTCACTAGTAAACCATTTAACTCGTCCGTTTCTTTTATTTCTATGAGCCCCCATAGTCTTTTATAAATTAAGCCCCTTAATTGTATAACAAATTTGTTTTCTAAAATAGGATTATTCATAAAAATTTCATGGCACAAAATAATACTGCCGCTGGTTTTATTGTGCATTATCCTGGTAAAAATACGGAAAATATCGTTGTCCTCTAGAGTTTCTTTGGCGCCTAGCTGTCTACACAACTCTCGGATGTGCTTCGTATTGATAGAAAGCAAACCATAGTTGATATTTGCGCCCCACTCTGTAAAGAGCTTTATCAGACTATAGTTGTTTTCCTTAACAGCTATTATTAATGCCACACGAAGGTCTATATCTTCTCCTAAAAATCCTGATTTTATTTGTATTCGGCCACGATCCATACAAAGCTTGAGAGGAGCATCATGCCACCATAGGCCACAATATTTCAAAATGCAGTGTTCATCTATTGACAAACACTGGCTGGCTATCGTCTTTTTGACGAGGGTCTGCAGAGAGAGCGGCAACGACATGTTTCTTTTTCACCAAAAAAAAATCAAATGTTCTCGTCTTTAAAGGTTAATTCATGTTCTTAAAATGTTCATTTCATGATAGTGATTAATAATATGGTTTAATAACGCTAGAAGGCTTGTTTATAAGACAGTCATAAGCAGTCTATAAGACAGTCTATAAGCAGTCTATAAGACAGTCTATGACTTAGTCTATAACTATAATTTCTGGATGGGCTGTAAGATACTCTTCGGCTCGTTTCAGATTTTTTGAAGTATATGTCTTTAGCATATCATATATTTCCTGGGGTTCGGTTACATCTAATACCAAGGTCACATCACGGCTGAAAAGCTGCTTTACTAAGAAAATGTTGCTCAAGTTATACATATAAGCTTTGTGCGCAATGAGTTGTGCCCTATCAAAATCGGCAGCCCCCCAAATCAATACAGAAAAACATGTTTAAAGTATTATTGTTATAGATAGAAAGATTCATGCCATAATCGAGACTAGCCCCCAACCTATGACAGTAATAAATGGCCGCGTAATTTTTTTCCCGCAAGCAAGCAAATTTCATCATCAGATTAGGGCTGATGCAAATCTCTTTTTCACGACACAACTCGTGTATGTCAAAAATGTTATTAAAATAAAGGCTACAAGCTACCCGCCAATAGAGGTGATTTTTATGCCTTTTATAGAAATAGTGAATAGCCTTTGTAAAATTATGTCGTAATGCCAGGGCAAACCAAAACTTTGTTAATAGGTGGTGCGCCGTATCCCCCGTCAACGGAATGTTTGAACAGGTGTACGTAACTGTGTCTAAAGTGGTTCTAGTTACGGTTTCCAAGAGTGGATTATGACAAAACATGTCATAACCCAGCAGAACTCCTGCACAGGATTTTAGCCTGGCCACTTCTTTTAAAATTTCCAGAAGACGGGGTTCGGATACAGGCGTTAAGCCTCCCAGTTCCGCACACAGCCGCTTTAGATACACGGCAGGAACACGTATAAGCCCATATTCAGGATTTGCGCCCCAATCCACAAATAAACGTATAAGTTCAAGATTATCGCTCTTCACGGCCTTTACTAGCGCCGCTTCGAGACAAAGATCATCCTCAGAAAAACACTGTAAATGTTTATACGAAAAAACTTGCTTACAATTGTTACATAGGTGAATAGGACCTAAATCCCACCACAAACCAAAACGCTGCAACGTATAATCATAGTCACTTGAAAGATAATTGCATGCCACAACTTTTTTGGCCAACGTTTGTAAAGACAACATACTAAGTTTAAAACATCTTAAATCTAAGCTAGCTAACTTTCAAGAAAACCCTCTATCCCTAAGAATATATCTTATAACTAGACTTATAGCAGTAAAAATCAACTTTGGTTATTCTTTTTAATATAAAACGTCTAATTACTTGCAAAGGACTATAAAGCCCATTTTCCTCAGCTAGAATTTTTATTTTTTAATGAAGTAGGGGGATATGTTTTCCCTTCAAGACCTTTGCCGAAAGCATCTTTTTATTCTTCCCGATGTTTTTGGCGAGCATGTACTACAACGATTAGGACTGTATTGGAGATGTCACGGCTCCCTTCAACGCATAGGAGACGACCACATACTCATACGACGGGATCTCATCCTTTCCACCAACGAGGCCTTAAGAATGGCGGGAGAGGAAGGAAACAATGAAGTAGTAAAGCTCTTGTTACTGTGGAAGGGAAATCTTCATTACGCCGTCATAGGAGCCTTGCAGGGTGATCAATATGACCTGATCCATAAGTATGAAAACCAAATCGGCGACTTTCATTTTATCTTACCATTGATTCAAGACGCGAATACGTTTGAAAAATGCCACGCTTTAGAACGTTTTTGTGGTGTTTCATGTCTGCTAAAACATGCTACAAAATACAACATGCTCCCTATTCTCCAAAAATACCAAGAAGAGCTGTCTATGAGAGCGTATCTTCACGAAACCCTATTTGAACTAGCATGCCTATGGCAGAGGTATGATGTCCTTAAATGGATAGAGCAAACCATACATGTTTACGACCTAAAGATTATGTTTAATATTGCCATCTCCAAGAGGGATCTGACTATGTACTCCTTAGGATATATTTTCCTTTTTGATAGAGGGAACACCGAAGCTACGTTGCTAACGCAACATCTCAAGAAGACAGCGGCCAAAGGGCTCCTCCACTTTGTGCTAGAAACGTTAAAATACGGCGGCAACATAGATACCGTCCTGACCCAAGCCGTAAAGTACAATCATAGAAAACTTTTAGATTATTTTCTGCGTCAACTACCTCGTAAACATATTGAAAAACTTTTGTTGCTGGCCGTGCAGGAAAAGGCTTCTAAAAAAACATTGAACTTACTGTTGTCACATTTAAACTACTCCGTGAAACGCATCAAAAAACTACCGCGCTATGTGATAGAGTACGAGTCCACCTTGGTGATAAAGATTTTATTAAAAAAAAGAGTGAACCTGATAGATGCCATGTTGGAAAAGATGGTAAGATATTTTTCTGCGACGAAAGTGAGGACGATCATGGATGAGCTTTCGATTAGTCCGGAAAGAGTCATTAAGATGGCTATACAGAAAATGAGAACGGATATCGTAATCCATACTTCTTATGTTTGGGAGGATGATCTAGAACGTCTTACTCGTCTTAAAAATATGGTATACACCATAAAGTACGAACATGGGAAAAAAATGTTAATTAAAGTCATGCACGGCATATACAAAAACTTATTATACGGCGAAAGGGAAAAAGTCATGTTTTATTTAGCCAAGCTCTATGTTGCTCAAAACGCGGCCACCCAATTCAGAGACATTTGTAAGGACTGTTACAAACTGGATGTGGCACGGTTTAAACCGCGGTTTAAGCAACTAATATTAGACTGTTTAGAAATTATTACTAAAAAATCTTGCTATAGTATCCTGGAAATCTTAGAAAAACATATTATTTCCCTGTTTACTATGAAAGTTATGACTGAAGAAGAAAAAAACCTATGTTTAGAAATATTATATAAAGTAATTCATTATAAAACAATACAATGTTAAAATTCAATAGATATCCATCATTAATATTGATTATATTTTCGAATATTATCTTCTATGGTGCAAGATAATCATCTAGCGCGTGAAACATGTCCTCTTCTCTTCAGGAACTTTGTCGAAAAAAGCTGCCTGACTGCATACTTCCAGAGTTTTTTGACGACTATGTATTGCAACTGTTAGGACTGCACTGGCAAGATCATGGTTCCCTTCAGCGTATCGAGAAGAACCAGATACTTGTTCAACAGGAACCCATCCATATCAATGAAGCACTCAAAGTAGCAGCATCGGAAGGGAACTATGAAATCGTAGAGCTGTTGTTGTCATGGGAGGCAGATCCCCGCTACGCCGTCGTAGGAGCCCTAGAAAGCAAATACTATGACCTGGTTTACAAATACTATGACCAAGTTAAAGACTGCCATGATATCTTGCCGCTGATTCAAAATCCGGAAACATTCGAAAGATGTCATGAGTTAAACAGCACCTGTTCACTGAAATGCTTATTCAAGCATGCTGTGATAAATGACATGCTGCCGATTCTTCAAAAATATACAGACTATCTGGATAGGTGGGAGTATTGCAGCCAGATGCTGTTCGAACTGGCATGTAGTAAAAAAAAATATGAGATGGTTGTGTGGATAGAGGGAGTTCTAGGCGTCGGCAAAGTTACATCTCTTTTCACCATTGCGATTAGCAACAGAGACCTACAGCTGTATTCTCTGGGCTACTCAATTATCCTTGAGAATTTGTACTCCTGTGGACAGGACCCCAAGTTTTTACTAAATCATTTCCTGCGAGACGTTTCAATAAAAGGGCTTCTACCCTTTGTAATCAAAACCATAGAATATGGTGGAAGCAAGGAGATAGCCATAACTCTGGCTAAAAAATATCAGCATAAACATATTTTGAAATACTTCGAAACCTGGGAAAGCTAGGTTCAGTATGGTGTACTCACTATTGTAGTGAATCGTATCCTGTAAATTTTGTAAAAAAGCTTAAACTTTTGACCACATCATATTGTTTTAGAAATCTCAAACCAGTGAACAACAGTCTTATCATACATTAAAATTCCAGTAAAATTTATATTTTTTTTGGTAAACAAATGTTTTCTCTTCAAGACATCTGTCGGAAACATCTTTTTCAACTTCCTGACGCTTTTGATGAATATATATTACAAGCGCTAGGACTATACTGGGAAAAACACGGATCTCTTCAACGAATAAGAAAGGACGCTGTGTTTGTACAGCGAAACATCGTCCTTTCTACCAATGAGGCCCTGAGAATCGCAGCCTCAGAGGGAAACGAAAGGGTAATAAAACTTCTGTTATCATGGGAGGGAAATTTTCATTATGTGATCATAGGAGCTCTAGAGGGTGACCAATATGACCTAATTCATAAGTATGATAGTCAAATTAAAGACTACCACATGATTTTATCATTGATCCAAAATGCAAATACCTTTGAAAAGTGTCATCAGTTATCCAATAGTAATATGTGGTGTCTTATACAGAATGCTATAAAATATAATATGCTCCCTATTCTCCAAAAACACAGAAATATTCTGACACATGAGGGAGAGAATCAGGAATTGTTTGAGATGGCATGTGAGGAACAGAAATATGACATAGTTTTATGGATAGGACAAACCCTAATGTTAAATGAGCCGGAGTTTATTTTTGATATCGCCTTCGAACGGATAGATTTTTCTTTATTAACAATGGGTTATAGCCTTCTTTTTGATAACAAGATGAGTAGTATAGACATTCATGATGAAGAAGATCTTACTTCATTACCAACAGAACACCTCGAAAAAGCAGCCACTAAGGGATGTTTCTTCTTTATGCTAGAAACTTTAAAACATGGTGGAAATGTAAATATGGCAGTCTTATCTAAAGCTGTTGAGTATAATCATAGAAAAATTTTAGACCATTTTATTCGGCGGCAAAAATGTTTATCACGTGAAGAGATTGAAAACCTATTATTAACCGCCATAACCAATTGTGCATCCATAAAAACGTTAAACTTACTCTTGTCTTACCTAAACTATTCCGTAAAAAATATCATTGGAAAAATAGTACAACATGTCATAAAAGATGGTGATTATACCATCATATTACTTTTAAAAAAAAAGAAAATAAACCTAGTGGAACCTGTTTTAACAGGTTTTATAGATTATTACTATAGCTATTGTTTTATAAAACATTTTATCCAAGAGTTTGCTATTCGTCCGGAAAAACTGATTAAAATGGCCGCGCGAAAAGGTAAACTAAATATGATTATCGAATTCCTTAACGAAAAATATGTTCATAAAGATGATCTTGGAACTATATTTAAATATCTCAAAACCCTAGTATGTACCATGAAACATAAAAAAGGAAAAGAGACATTAATTGTTCTTATTCATAAAATATATCAAGATATTCATCTGGAGACTAAAGAAAAATTTAAATTATTAAGATTTTATGTCATGCATGATGCAACTATCCAATTTCTATCTATGTGCAAAGACTGTTTTAATTTAGCCGGTTTTAAACCATTTGTTTTAGAATGTTTGGATATTGCTATTAAAAAAAATTACCCTGATATGATACAATATATAGAAATTCTATCGAAATCTGAGTAAAATTTATTTTTTTGATCAGAGTAAGAAAATGTTCTCCCTCCAGGAGATCTGTCGAAAGAACATCTACTTTCTACCTGACTGGCTCGGTGAGCATGTGATTCAGCGACTAGGTCTGTACTGGGAAAAACATGGTTCTCTTCAGCGAATCGGAGACAACTATGTACTTATACAACAGGACCTCATCATCCCCATCAATGAAGCCCTAAGAATGGCAGGGGAGGAGGGGAATGATGAGGTGGTACAACTCCTATTACTATGGGAGGGAAACATTCATTATGCCATCATAGGAGCTTTGGAGAGTGACCATTATAGCCTAATACGTAAGCTCTATGACCAAATCGAAGACTGTCACGACATCCTTCCCTTGATTCAAGACCCAAAACTCTTTGAAAAATGCCATGAATTAGATAAATCTTGTAACATTTTATGTCTCGTATTACACGCCGTAAAAAACGATATGCTTTGCATTCTTCAAGAGTATAAAATGCATCTAAGTGGAGAGGATATTCAAGTGGTGTTTGAAACAGCATGCCGTTCACAAAAAAACGATATTGTGTCATGGATGGGACAAAATATTGCAATATACAACTCCGGAGTTATTTTTGATATTGCCTTTGATAAGATGAATGTGTCCTTATTATCTATAGGGTACACGCTTCTTTTCAATCATCATATAAATAATACGAACGAAAATATTAATTCTTTATTGACACAACATCTTGAATGGGCTGCCGGCATGGGCCTTCTTCATTTTATGCTGGAAACTTTAAAGTATGGCGGGGATGTAACGATAATAGTTTTGTCTGAGGCCGTAAAATATGACCACAGAAAGATTTTAGATTATTTTCTCCGTCGAAAAAACTTGTACCAAGAAGATCTTGAAGAACTATTATTGTTGGCGATACGTGCAGATTGTTCTAAAAAGACCTTAAACTTGTTATTATCTTACTTAAACTATTCCATAAACAATATCCGTAAAAAAATATTACAATGTGTAAAAGAATATGAAACGACCGTTATTATAAAAATTCTATGGAAAAGAAAGATAAATCTGATAGAGCCCATTTTGGCAGACTTTATAGGATATCATAGCTATACCTATATGGTAGATTTTATGCGCGAGTTTTCCATCCATCCGGAAAAAATGATCAAAATGGCTGCGCGAGAATCGAGGGAGGACTTAATCATAAAATTTTCCAAAAAAGTTTGCAAAGAGCCTAAAGATAGACTTCACTATCTCAAAAGCTTAGTGTATACTATGCGACATAAAGAAGGCAAACAACTGTTAATTTATACAATCCATAACTTATACAAAGCTTGTCATCTAGAGAGTAAAGAAATGTTTAATTTGGCACGATTTTATGCACGGCATAATGCAGTGATCCAGTTCAAATCGATTTGTCACGATCTCTCCAAGCTGAATATTAATATCAAAAACTTGTTGTTAGAATGTTTAGGTATTGCTATTAAAAAAAATTACTTTCAACTTATCAAAACAATAGAAACGGATATGCGTTATGAGTAAAATTTTTGGATAAGGGAAGATTCTACCAAACTAACTAAGACCTTTCGCTAGAATGTATCTTATTGTTAATATAGATTAGGTATGACGTGAAAAAATAGATTAGGTAGGTTGTGAAAAACAGATTAAACTTAAAATTATGTGTATTATGTAAAATTTTAGAAATAAAAATTTATTTTTTTTATTGAGGGTACGGAAAATGTTCTCCCTACAGGACCTCTGTCGGAAGAACACTTTCTTCCTTCCAAATGATTTTAGCAAGCATACTCTACAACGGCTGGGGTTGTATTGGAAAGAGCATGGATCCGTCCATCGAATAGAAAAGGACAGCATAATGATACAGAATGAATTGGTTCTTTCTATCAATGATGCTTTACAGCTTGCAGGAGAGGAGGGGGACACAGATGTGGTACAGCTCTTGTTACTATGGGAGGGAAATCTGCATTATGCCATCATAGGAGCTTTGAAGACTGAGAATTATAATCTAGTATGTGAGTACCATAGCCAAATTCAGGACTGGCATATTCTCCTACCCTTGATTCAAGATCCAGAAACATTCGAAAAATGTCATGATTTAAGCCTTGGATGTGACCTTATATGCCTTCTCCAACATGCTGTAAAATGTGATATGCTTTCTATTCTTGTTAAATATAAGGAGGATTTACTAAATGTAAGGATTAGGCATCGTACCCAATCCCTGTTTGTTTTGGCATGCGAAAATCGGAGATTTGAGATTATTGAATGGATAGGTCAAAATCTGTCAATTCCTGAACCTGAGGCCATTTTTAGCATTGCTATTGTTACAAAAGATGTAGAACTGTTTTCCTTAGGATATAAAATTATTTTTGATTACATGCAAAGACAAGGAATTTTTCAATTAACCAATGTAGTTCGCATGCTTCTGCTAAATCGTCATATTGGTATGGCAATAGAAAAAGGACTTTTACCTTTTATTCTGGAAACTTTAAAATATGGTGGTAGTGTAAAAAGAGCTTTATCTTATGCAGTAATAGATAATAAAAGAAAAATTATAGACTATCTTGTACGCCATGAAAATATACCCCGAGGAACTATTGAAAGACTTTTGCATCTAGCTGTGAAAAAACAATCTTCCAGGAAAACTTTGAACTTGTTGCTATCTTACATAAATTACAAGGTGAAAAATGTTAAAAAGCTGGTAGAGCATGTAGTAGATCACAAATCCACTCTTGTGTTAAAAATTTTATTGGAAAAAAAGGAAAATCTAGTGGATGCTGTTTTAACAAGACTTGTAAAACATTCTACATATTTCCAGGTGAGAGAATTTATCCAGGAGTTTTCCATCAGCCCAGAAAAATTCATTAAAATAGCTGTGCGGGAAAAGAAAAATGTGTTAATCGAGGCTATTTCTGAAGATATTTGGGAAAATCCCACAGAAAGAATTACTTATCTCAAACAGATAGTGCACACCATAAAATATGAAAGTGGAAGGCGGTTTTTGATAGACATCATTCACAGCATTTACCAAAGTTACTCACTAAAACACGAAGATATTCTTAAACTGGCAACATTTTATGTCAAACACAATGCAATCACCCATTTTAAAGATCTCTGCAAATATCTTTGGCTGAACAGAGGAACAGAAAGTAAGAAACTGTTTTTAGAGTGCTTGGAAATTGCTGATGAGAAGGAGTTTCCTGATATTAAAAGTATTGTGAGTGAATATATTAACTACTTGTTTACTGCAGGAGCTATTACCAAGGAAGAAATCATGCAAGCTTATGCTTTAGAGTATGACATGTATTAAATTTCTGAAGGTAGACTAAAATATACTATATATTAAAAAATCCAAAACAGCCATTTTTAACTAACTTCTTCTTAAAAACTCTGGATAAAAATTTATTTTTTTTAATTTGAGTAGGGAAAATGTTCTCCCTTCAGGACCTCTGTCGGAAGAACACCTTCTTCCTTCCAGATAATTTTAGCAAGCATACCCTGTATTTGCTGGGGTTATACTGGAAGGGACATGGATCTATCCAAAGAACAATGAATGTTGGTGTACTGATAGAGCATAATCTTAATCTTTCCATCAATGAAGCCTTAATCCTTGCAGGAGAAGAGGGAAACAATGATGTAGTACAACTCTTATTGCTATGGGAAGGAAATCTTCATTATGCCATCATAGGAGCTTTGAAGACTGAGAAATATGGCTTAATATGTGAGTACCATAGCCAAATTCAGGACTGGCATGTTCTCCTCCCCTTGATTCAAGATCCAGAAACATTCGAAAAATGTCATGATTTAAGCCTTGAATGTGATCTTTCATGCCTTCTCCAACATGCTGTAAAATATAACATGCTTTCTATTCTTGTTAAATATAAAGAGGATCTATTAAATGTACTATTTAGGCAACAAATTCAAGGACTATTTATTTTAGCATGTGAACATCGGAGGATTGAGATTCTTACGTGGATGGGTCAAAATCTGCCAATTCCTGATCCTGAGCCTATTTTTAGCATTGCTGTTGTCACAAAAGATTTAGAAATGTTTTCCTTAGGGTACAAGATTGTTTTTGAATACATGGAAAATCAAGGACTATTTCATTTAACCCAGGTAGTTCGTATGGTTATGCTAAATCATCACCTTGGCATGGTAATAAATAAAGGACTTTTACCCTTTGTGCTGGAAACTTTAAAACATGGTGGGAATGTAAATAGAGCCTTATCTTATGCTGTCACACAAAACAAAAGAAAGATTTTAGACCATGTTGTTCGCCAAAAGAATATACCCCATAAAACCATTGAAAGAATGTTGCATCTGGCTGTAAAAAAGCATGCTCCCAGGAAAACTCTGAACTTGTTACTATCTTACATAAATTACAAGGTGAAAAATGTTAAAAAGTTGTTAGAACATGTAGTGAAATACAACTCTACTCTTGTGATAAGAATCTTGTTAGAAAAAAAGAAAAACCTGCTGGATGCTACTTTGACAAGATATGTCAAAGATTCTACATACTTTCAGGTGAAAGAATTTATGCAAGACTTCTCCATCAGCCCAGAAAAATTCATTAAAATAGCTGTGCGGGAAAAGAGGAATGTGTTGATCAAGGGTATTTCTGAAGATATTTGGGAAAATCCCGCGGAAAGAATCAGGAATCTTAAGCAGATAGTGTGTACCATAAAATATGAAAGTGGAAGACAATTCCTGATAAATATCATTCACACCATTTACCAGAGTTATTCTTTGAAACCTGAAGAAATTCTTAAACTGGCAACATTTTATGTCAAACACAATGCAACCACCCATTTTAAAGATCTCTGCAAATATCTTTGGCTGAACAGAGGAACAGAAAGTAAGAAACTGTTTTTAGAGTGCTTGGAAATTGCTGATGAGAAGGAGTTTCCTGATATTAAAAGTATTGTGAGTGAATATATTAACTACTTGTTTACTGCAGGAGCTATTACCAAGGAAGAAATCATGCAAGTCTATGCTTTGGAGTATGCCATGTATTAAATTTCTGAATAAGTAAGCAATAGATAGGTTTTAGAATATGCTGTATTAAGTTAGTTTCTGAATAAGTAATTAATAGATAGATTTTAGTTTATGTAAAAATGTTAACATTTGTTAATAAGTTTTAGATAATTACTATTTTAGAGTTACTATTTTAGATTTTACCATTTTAGCTATTATTATCTTAAATAATCACTATTTTAGATAGGTCCCCGTATTAAAAACCAAATTAACCATTATCTATGTTTTTAATAATACTTTTTAAAAACCCTCCATAAAAATTTATTTTTTTTTCATAAAAGTAGAGAAAATGTTCTCCCTACAGGATCTCTGTCGGAAGAACCTTTTTCTTCCACTTGAGCCCTTAGGCAAGCATGTGGTTCAACGGCTGGGATTATACTGGGAAGGCCATGGTTCACTTAAACGAGTGGGTGATTGCTTTATATGTGTAGACAAGATTTGGATCCTATCCATCCATAAGGCTATACAAATTGCAGCCTCGGAAGGAAATGAGAACATTGTCAAGCTTTTCTTACTGTGGAAGGGGAGTCTACAATATGCCATCATAGGAGCCTTAGAGGGCAGGCAATATGATCTGATTCAAAAATATTACAACCAAATTGGGGACTGCCATGAGATTCTACCACTGATTCAAGATCCAGAAATTTACGAAAGATGCCATGAATTAAATGTTACATGTACCTTTCAATGCTTATTTCAACATGCTATAAGAGATAACATGCTGCCCATTTTCCAAAAATATGGAGAAGATCTGAATGGAAACAGAAGAATGGTTCAACTTCTATATGAAATGGCATGCCGATTACAAAATTATGATATCATCAAATGGATAGGATTTAACCTGCATGTTTATAACTTGGAAGCCATTTTTAGCATTGCTTTTGTTAGAAAGGATTTAACTTTGTATTCTTTAGGCTACATGCTTCTTCTGGGTAGAATGAGTACTGAAGATAGAAACTTTATTTCAATCATAACACGCCATCTTGAATACGCATCAAAAAAGGGACTTTTTGACTTTGTACTAGAATCTTTGAAATACGGAGGTCAAGTGGATACAGTGTTGTTTCAGGCTGTAAAATACAACCATAGAAAAATTTTGGCCCATTTTATTCATGAAATTCCGCGTGAAACAGTTGAAAAGCTGATACTCCATGCTGTAGAATCGCGGGCCTCCAGGAAAACATTCAACCTGCTTTTATCTTCCATAAACTACTGTGTAAACCCTTTTGTCAAAAAACTACTGCACACCGTGGTGAAACACAAGTACATGCTTATCATAAAGCTTTTGCTCGAGCGGCCCAAAAAGAAGATAAACCTGGTAGATGCTGCTCTATTCAAACTTGTAAAATACTCTACTTATGCAGAAATAGTAAAATTCATGAAAGAGTTTTCTGTGGACCCAGAAAGGGTGGTCAAAATGGCAGCACGACTCATGAGAGTGGACCTGATTAAAAAGATTTCTAACGATGCATGGGAAAATAAACTAGAGAGAATCAAGCACCTTAAACAGATGGTAAATACCATGAACCACAGAAATGGAAAAAATCTATTGATGTACAATATTCACAATATTACTGGATATACCTGCTTGAACACCAAAGAAGCATTTAACTTAACAAGATTTTATGCTGTCCACAATGCAACATGTTTGTTTAAAGAAATGTGTAAAAGCTGTTTTGTACATGATAAAATACAGTTCAGAGAATTGCTTGAAGATTGTTTACATATTGCTAATAGGCATGATTATATCCAGATTGCAGAAACCGCAGATGAATGTATCAAATATATAGATCTTATTACACCTAAGTAAATCATGAAAATATATCAAGTAAATCCAGATTAAATCAGGCTAATTGTAAATAGTTGTAGATACCATATAATGAATGTTTTATTAGGATAGTAGTTAATAGTTTAGTTAAGACAGTAGTTCTTTCTGTTAAGATAGTAGTTCTGTTAAGATAGTAGTTTAGTTATGATAGTGGTTTAGTTAAGACAATAGTTTTGTTAAGACAGTAGTTCTGTTAAGTCAATAGTTCAGTTAAGTCAATAGTTTTGTTAAGTCAATAGTTCTGCTAATACATTAGTTCTGTTAAGATAATAAAAATTTATTTTTTTTCATCAAGGTAGAGAAAATGTTCTCCCTTCAGGAGCTCTGCCGGAAGAACATTTACATTCTTCCTTACCCCTTGGGTAAGCATGTACTTCAACAACTAGGGCTGTACTGGAAGGGACATGGATCTCTTCAACGAATCGGAGATGACCATGTACTCTTACAACAGGACCTGATCTTTTCCATCAATGAGGCCTTAAGAATGGCGGCAGAAGAAGGAAACAATGAAGTAGTAAAGCTCTTGTTACTGTGGGAGGGAAACCTTCATTATGCCATCATAGGAGCTTTAGAGGGCGACCGATACGACCTTATCCATAAATATTATGAACAAATTGGGGACTGCCACAAGATTCTTCCTTTAATCCAAGACCCGCAAATCTTTGAAAAATGCCATGAATTGAGTAACTCCTGTAACATTCGATGCCTTTTAGAACATGCAGTAAAACACAACATGCTTTCTATTCTTCAAAAACATAAGGATCAAATAAGATTACACATGGCATTAACCCAAATACTATTTGAATTGGCGTGCCATGAACGTAAGAATGACATCATTAGATGGATCGGTTATTCCCTGCACATATACCATCTAGAGACTATTTTTGATGTTGCATTCGCCCATAAAAATTTATCCTTATACGTTTTAGGGTATGAACTTCTCATGCACAAAGTAAATACAGAGGCTGCAAATATAGATTTACCCAATTTGCTATCATATCACCTTCGAACTGCGGCGGCAGGAGGTCTTCTTAATTTTATGTTAGAAACAATAAAGCATGGTGGGTGTGTGGATAAAACCGTTCTATCCGCGGCTATCAGGTACAAGCATAGGAAAATTGTGGCTCATTTTATTCATCAGGTTCCCCGTAAAACCGTTAAAAAACTGCTACTCTATGCTGTGCAGGCTCGGGCCCCCAAAAAAACACTAAATCTACTTTTATCTTCCTTAAACTATGCCGTGCACACCATCACCAAACAACTCGTACACAATGTCATCAACTACAGTTCCACGCTTGTCGTAAAGCTTTTACTCATGCGGCGAAAAAGGAAGTTAAACCTAGTAGATGCCGTTTTAGCCAGACTTGTAAAATATTCCACCTATACAGACATTGTACAATTCATGGGTGAGTTTTCTGTGAGCCCAGAAAGGGTGATCAAAATGGCTGCACGGGAATCCAGGACCTTTCTGATTGAAATGATCTCCAAAGCTGCTTGGGGAAATCACCCACAGACGTTGATTCATCATCTCAAACACCTAACCAATACCATGAAGCCTCAATCTGGAAAAGACCTCATCATATATACCATCCACTATATTTATCTAAACTCTAATATGCTGGTAGCGGAGGAGGAAAAAAATATTTTTAAATTAGCAAAATTTTATGCGAATCATAACGCGGTAAACAGGTTTAAACAAATTTGTGAAGACTATTATATGTTAGATGCACGATTTAAAACACTTATTTTAGAATGTTTTGAAATTGCCGTCCAGAAAAACTATCCTAGAATTGCAAATATTGTGGATGACTATATTCGATTCCTTTTTTACAGGGGAAATATAACCGAGGAAGAAATTCGTGAAGCCTATTCTTTAAAAGATGCTGAGGTTTATGTAGATTTAAAATGGTTACAACAAGGAGAAATGGTTTAAACTAAATCCGGTTTAAACTAAATTCGGTTTAAACTACATTTGGTTTATCATTAGTCATTGAAACCATCGAAAAAAAGTATTTGTTTATCCCCATAAACTCATCTTTTTTTTGTCTCAAAGTTTGACACTAAAATTCAGTGTTTTATAGTGTTTATAATTAAGTGTTTTGCATGCATTGCAGAAATTTTCATCTTTTTTAATTGGTTCAATACCACATGTCATACAATATGTTGTTTGATTATCAAGATTAACTTTATGAAAGGAAAGTAAGTGAGCCGCAAATTTAAAAGTAAAATATCTTTCATTTAAAATGATCTTATGAATGTATTTTCGATAAGGAGGAATAAAAGCATTTGCCAAAATAAATCGCATAAAGGGCTTGGAAAAACCCATATCTTCTAATCTTTTGTGGGTATAAACCCTATTTTTGTGTTTTACAAAAACTTCATTGTTATAATAGTCGTTATAGCTATCAATCATTTTTTTAAGTCCTATAATGCCCAAGGTTGCACGCATAAAGCCGCAGTTTCTACTCCAAAAGCCATGCACCTGTAAAGGGTGCTTTTCATATAACCAATTACAAAATTTCATTCCGCAACAGTAGCATGTTATTTCAGTGGGGGATGTATAGAAAAATCCGGCATTCGAAAATTTTTCATAATTTTTTATGCCATGGATTGCGAAGCTTTGATTTCGTGCATCTATGGAGCTATAGCCTACATATTTAGGTTTTACTTCAAATAATCGCAAAGAGATGTATGGATCTATTGTATTTATTTTAGGAAACATTTCATAATTTTAAATTCTTATATATAATATAAAAAAAATTACAAACATTTGTAATGATCATCCTCAATTGAAAGCTGAGTTGTAGACTTTATTTTTCTAATTATACGAAGAAGGTAGGTTCTCATAAAGCCTTCAAGATGACTATTGATGTTTCCAATACATTTTCTCAATGAGTTCATAAACCCAGACATTTTGCTAATGGCTTGGCAAAGTGCCAACAAGTTGTCCACAAAGTACTGGTAGATTGCCTACTAGCTATAGCTAGCTATAGTGAGCCAACCTCTCTGTATTTATTTTATATATTTCATTTTTTAATAGATTTAATATTTTTATAAAAAAATATTTAGTTTTTTATACAAGAATGTCGACAAAAAAAAAGCCCACAATTACCAAGCAAGAGCTTTACTCCTTAGTAGCGGCAGATACCCAGTTAAATAAAGCATTGATTGAAAGAATCTTTACAAGCCAGCAAAAAATAATCCAAAATGCTTTAAAGCACAATCAAGAAGTTATTATACCACCCGGAATCAAGTTCACCGTCGTTACAGTGAAAGCTAAACCTGCTCGCCAGGGCCATAACCCCGCAACAGGAGAGCCTATTCAAATTAAAGCCAAGCCTGAACATAAAGCCGTAAAGATACGAGCATTGAAACCTGTTCATGATATGTTAAATTAAATAATAAAGCCATCATCATCGTCGTATTCTTCTTTATCATTATTATCTTCAATACATTTTTGCCAATCGAAATCGAATAAATTCAGATCCTGGACATTTAAATACTTATCATCGTACATTTTAATATAATTTAAACATGAGTTGTTGTCAAAAACTTTTATCGTTTTTGTTAAAATCATTGTATGAATAATTTCCTTATTAAGAGTTGCCGGAATAATACAAAGCCTATTTTTAGGTACATCATTCATGATAATAGTAAAATTAGTAAAAATTGTTTCTTGTTTTTCTTTTGTTTCAAATAAACGTTGTAAGGTTAAAGGTTTTTCATTCAATGGTTTCTTTGAAGATAAAAAGAATGTATAATCCGGTTTAAAGATATTTTTGGTTTCAATCGTGATTCCATCTGCTTGAGCATATGCTAAACCAGACCAAATATAATGGTCCACTATTACAATAAAATTTAGTTTAAGTAGTGCTGCAATTTCTGCGCTAAATTCACTATGATGTTTTGTAAATAATTTATGCAATTGTTCCGATGACATTTCTGTCATTTTATTTAACACCTGCAATATAAGGCCACCGGTGGTCGTGTCTGGATTAGGAAAACGTATACATACAGCATTATAATCCATGCATTCTAATGCCTTTTTTAATCTCATTGCCTGTGTGCTTTTTCCCACACCATTGATTCCCTCGATGGTAATAAGTATTCCACGCATGATTAATAAATGGGTAAAAAGAGTTCAGTTTTTAACATTTCTTACAAATCTATTTTTATACAACATTGTACAACACTACATTAACCGCATACGATGTTATAGCTTCATTAAATATTTGCTTTTATATAATCTTTACCGACCTATATTTGGCAGATCACTGCAGATGGCCATAACTAAGATAAAAATTATTTCAGATGCTACTGCGGCAGTATTATTAAAATCATGTGCGGCAATGTATGACGTCTTAATAGATAAAAAATTTAAGTAAAACAAATTTGAATAAAAAAAATAATAGTTATGATGGCGTTGTTACACAAAGAAAAGCTTATAGAGTGCATCGAAAATGAGGTGCTTAGCGGTGGTACAGTATTGCTCCTAGTAAAGAATATTGTTGTGTCAGAAATTTCATACATTGACAATAGTTATAAATATTTTACCTTTAATGCCAATCATGATCTGAAAAGCAAAGAAGATCTTAAAGGAGCAACATCCAACAACATTGCTAAAATGATTTATAATTGGATTATAAAAAATCCTCAAAATAATAAGATTTGGAGTGGTGAGCCGCGCACTCAAATTTATTTTGAAAATGATTTATATCATACGAATTATAATCATGAATGTATAAAAGATTTTTGGGATGTTTCAACCTCAGTCGGTCCTTGTATCTTTAATGATCGTAGCATTTGGTGTACTAAATGCACATCCTTTTACCCATTTACCAACATTATATCGCCCAATATATTCCAATAAATTAGATATCTTTTACTACTATTAAAATAGTTAATAACCTTATAGGATAATTAGGTACTTTATTACGATGAATTAGTTTATGTTATTTTATAATTAGGTACTTTATTACGATGAATTAGTTTATGTTATTTTATAATTAGTTACTTTATTACGATGAATCTTTATTAACGATTCTTTTATTAATGAATTATCATAAGATAAATAATTATTTTTTTCTCCATATATCGCATAATAGGTCTGGTATGGGCTAAAAGTATGTTTCAAACTATTTACAATAGAATTTCTGTTAAGAAAACATACATAATTTGAATAAAATTTTTTTAAATATCACCGAAACATCCAACATGGTGTTAATAGAGTTTTTAACAGGTTTCTTCTATTTATATGGAAAGAGACTGTTTTCCATTAGCAAAGTCATGGACATGATATGTCTAGACTATTATACCATTATTCCTGCTCCTCTGGCGATGATGTTAGCGGCAAGAATAAAAAACTATGACCTCATGAAACGACTGCACGAATGGGAAATCTCTGTTGACTACGCTCTACTTGTAGTAGATGATGTGCCGAGTATCGACTTTTGCTTAAGTCTTGGCGCTAAATCCCCGACTAGAGCACAAAAAAGACAACTGTTGAGGGACAACACGTTTAATCCCGTGTATAAGTATCTTATGAACTGTTCCGGTTTTCCAACAAGAAGAGAAAAAAACATTCCTTGTGATGTTCAATGCGAAAGACTGCAAAAAAACATTATAAAAGAACTGGTATTCAACTGCTCCGTGCTGCTTGAAATGGTACTGCACACAGAAAGAGAATATGCATACGCCCTACACTGTGCTGCAAAACATAACCAATTGCCCATCCTCATGTATTGTTGGCAACAATCCACAGACGCGGAATCCATTTTGTTGAAAACCTGCTGTTCTGATAAGAACATCAATTGTTTTAATTATTGTATTCTATATGGCGGCGCCCAGAATTTGAATGCTGCAATGGTGGAAGCGGCAAAGCATGATGCCCGGATGCTGATAAACTACTGTGTCATGCTTGGTGGAAGATCCTTAAACGAAGCAAAAGAAACGGCTGCCATGTTTGGACACATTGAATGCGCACAACACTGTTTTGAACTGCAGTCTTATGTCATGGACGCATTGAATGCGGACGACGCTGATTAAAGCGACAATCTTACGTCATGAATGACTGTCTTTTGAGTATCTATACTTACATTATATTTTTTTATGAAAAAAATATAAAGGTTGTATACAAAGGTATACAAGAAATTTGGATCATTAAACAATAATTAATTTAGACACAGGAAAACGATCTAGATCGATCAAAAAGCTATTTTTTTGCACACAGAATATTTAGATAATTAAGAGATTACTTTCCATACTTGTTCAGTTTTTTTACACACAGGAAGTTTGGATTCTGTTCAGGAAGTTTTTCATAGACATTATTTTTACAGCCAGTAATAATAATTTTGGGCTTTTTCTTAAACCACCGGTGGAAAACATCCAGCTTGTAAAGAGGGAAATGCATGTAGAGAGGTTTTTGGTAGTCATGGTTAAGAGATTTGACTAACTCCATATTTCCTGTAAAGACTGCCCAGTCCCAAGCAGTAAAGCCTCTATGATAGTCTTTTTGAGTCGGATCTGCTCCAAATTTTATGAGAGAAAGCACATTTGAAGTACGGCCCCGTATTGCGGCCTTCATCACAGGAGTCATCCCATTAAAATTCGGTAAACAAATTTTGGTCCCATTTTTTCCGAAATAGCCCAACACCTCTTCCAGGATTAAATGATTTTTTTTCTCAGCTAAATAATGTAAAGCAGAGTTTCCATCTTTATCCCTCCTATGAGGGTTAATTATTTCTCCGGGATAAGATTCTTGTTCAAAAAGAAATTTTAAAAAGTCTATACGTCCGTAGATGCATATCCACATGAATACCGAGGATCCATTTTTATCGCATCTATTGACAATCCACGGATCTGTTTTAAAAAATTCCTCAAATAGTGTAAGATTCCCATTTCTGATATGTTTTTTAATCCATTTAACAAACAAGTTTTCTATCTCCCTTTCTGGAAACATGTGTTCCATTTTGAATGTCGCCCCACCCCATTATATAATTTTACTCCTTTAATTTTTAATGTCCTTTTTTTTCGGACTTCTTTGGATAAGCTGTTTATTACCATCTTTAAATGCCTTATAGCGGGGAGGAGCCAGGCCGCTTTCCCATATGTGCGGTAATTCTTGGTGTTTATGCTTGCCTTTGGCATATCCAGGCCAGTATTTTTCGATATATTCGGGATTTGTTTTTACATATTCTTTAAAGGTCCGATAGGCTTCTTGAATACAGGTAGGCTCGCCTGTATAATTTCCGTGTTCATCTTCCTTTAAAAAGCCATTAACCCTGTCCTTTCTCCACTTAAGATTGTGCTTTCCAAAAATACGGTCAAGATCTTCGGCCTGCTGGGGTGGGATCATAAACCCCTTTTTAGGTCGAAGCTTTTTATTTTTTCCATAGCTTCGGCCATCGCGTTGCGAAACAGTGGTTAGGACGCCCGACAGCCTTTCCATGGGCGTCGCGTCCAATCCTATCCATCCACCCTGATGAATATCAATGGCAACAAGCTCTCCTTTATTTTGGGCAAGCCAAGTTTCCAAGAATGCCATGCTTTCTTCCCAGGGATAAGGCCCGCCAACACCACGGGTTGTCCAATCTTGCAAGGACTCCAGGTCCGACACCTGGTAAGGCTCTAAAGAAGACGGTTCCTTGTTTTTGTACTGCAAATAGGATTTAATGACCCATTTATACCATGTGTCGAACCGCAGCGTGGCGCCTCCAAAGTGAAAGCCGTCGTTGATTTTAGGATATCTGCAACATATTTCAACCGTACGTTTGAGTTCTGCAAAGGCGGCCTTCCAAGGAAGTCTCTCGCTGCGGGTAAGGCGGTCTATTTTGCCCTGCGTACCATAGCGTATGGCGTGACGTGCCAATTGCAACAATTCTGACACCGATCCGTGGGCCCCGATCCAGTTTATCGGATAGGCAACCTCTGAAGGGTTTAAAAGATGCTCGTAAAAGCGTGGATCTTCAGACGCCAAGGCGTCTGCAAAAGGGATAATACTAGAAAACCTGTCTAGGCATACGTTTTCTGTGTTTACTTCTAAAGGTAGAAAAATGGTTGCGTGAGGCTTTTGAACCTGCTTGTTCAGCGGTCTGCATATGCTTTGAATAATGTCTCTAGGACTATGACGCGGCGCTGCAAAAAATACCGCGTTTAGTTCCGGAACCTCTACGCCCTCTTGAAAGAGTCGACAGTTTAATAAAATAACGGGTTCCTTTGAGGAACAAAATTCTGTAAATGTTTTGAGGATAACCTGTCGCGGCAGGGTTGAGTGAGCTATCAGGGCATAGACCCCTTGGTCTACCAACTCCGCGTATAGCTCCCTGGCCTGTTTAATATCACGGGTGAATACCAGCATTTTAGGAGCCGGTATATTGGTTTTTAAATAGGCTAAGGCCATTATAATTTGCTTTACTATGATCTGTTTCGTGGTCTCCTCTCTGTTACTCGGTTGGTGAGCCAATTTGGGCGCGGCTACCATTTGTAATTCAAAATCATTTACATAGCCGGCCTCTATGCCTTCTCGCAGATAGTAGCGAAAGGCAACGCCGCCAAAAAGTTCACGATTTTTCATGGAAAGCGGGGTGTCGTACCTGGGCGTTGCCGTTAAAAAAAGTCGGTGCCCTTTTTTAAAGTTGAGCAACACATGGGTAAAGGGCCGTGTCTCCCATTCACCGCAAATCCGGTGACATTCATCGCTAATAATAAGATCGAAATCATCCACCAGTAGCGTGGAGGATTGGTAGGTGGCAATCACAAGAAGAGAAGGGGCCTCCCGTATCCGTTTTGCAATAAAGACAGGATTGGTGGTCATTTCTATATTGTCGTGATTTAGCACAATGCGGGTCTGGTCAGACCCCACAAGCAAAACGTTCTTCAAAGAAATTCCATACTGATAGAGTTTTTCCAGAGTCTGCCGTAGTAGGGACAGGCCCGGCACCAGGTACAAAACTTTTCCTTGAAGATAATTGGAGAGGATAAGATAGGCGACGCGAGTTTTGCCGCAGCGGCAGGCCATCTGCAGAATGGCCCTTCCACTTCGCCGCAGCTCCTGATAGCCCATATTGGCCGCCTCCTTCTGATAAAGCCGATCCTCGATTGCAGTCCGTGTCTCATCTGTAGAAAAAAATAATACGTCATCTGCGAAATGTTCTTCATCTTCCACAGGAGTTATCACCAGGTGTCTCAGTTTCTCCTTGCTTATCAGCGGATCAGAGGGCAAAGATGGCTCAACCACTATCGTGGAATCATTCATCTCATAAGTGGGAGAATCACACAAAGTATAGCTTATGTCCAGACAGTTTGCAACATCCTCAGCCAATTGTTTTATTTTTTCGGGTAAAAGACATACGAGTTCTTTGTTTTTGACGCGAAAAAACTGTGCGCAATACAACACCCCTGCTTCAATTTTTTGCGCATCCTTCTTTGTAGATGTTTCCAATGTGAAACAATACTTCCATTCATCCGTAAAACAGGTTGTATAAGATCCATCATGGAGCCTAGCGGCCAAGTTTCCTGTGTGCCCAACTTTATGTAAGGATTGGGCCTCCAGCCAGGGATGAACCGCCACGTAAAATCCTGCGCACATGCTATATCAAATTGCAGTTTCTTAATAACTGTACACAGGATCTGAAAAACATGTGATTACAAAATTTAGATAAGAAATATTTAATATTAAAAATCACGGAATACATGTCACTGTGTAGAGAGAAAGCCAAAAACTCCTCTTGACCGCCGTGGGAAATCATCCAGGGTAGTAGGTTGTGTTTCATGAAGTTGTATGCCGTAGTGATCACCGTGGACTCCAGATGGTTATTGGCGTCTTTGCAATACTTTGCCATCTTGGCAGAAAAGACGATAAATCCACAAATTCTACCCCAGTTGATAAGATCCTTAAACAGCTCAGTCACAACCCCAGTAAACTGGGTTTTAATTTCTTGGACACTCGTAAGAGAAAAGGTAATTGTAACCTGTTTGTTCAAACACTCATCATAATAGGTTAAAATTTTTTTTATTTGTTGTTGATATGGGCTAAGCTCATGCTCTGAAATATCATTAATGTAATATTTAATATAACCCACTAGTATTTCATTAATGATATTATGATATATTAACTCTTCTCCCTCCATAGCGGCACCCTATATTTTTTTATTTAGGTTTCAATGTTATCACAATTGCGATACAATTGTGATACAATTGTGATACAATTGTGACACGACTGTGTTGTATACAACAAATGTTAGGCCACGTATAGCAACCTATATGTTAAGAAATATTTTTATCCCAACATTGGTTGGAAACGAGCAGCCGCAAAGAAGTCATTAAAATAAGCCATTTAAAGATTTAGAATTTATATGTATACAACTGTACAATGGAAGCAGTTCTTACCAAACTCGACCAGGAGGAAAAAAAGGCTCTCCAAAATTTTCATCGTTGTGCTTGGGAAGAAACTAAAAATATTATAAACGATTTTCTTGAAATCCCTGAGGAACGATGCACCTATAAATTCAACTCATACACAAAAAAAATGGAGCTTTTATTTACCCCCGAATTCCACACCGCCTGGCATGAAGTTCCTGAGTGCAGAGAGTTCATATTAAACTTTTTGAGACTCATTTCGGGACATCGAGTGGTATTAAAAGGCCCTACATTTGTTTTTACAAAAGAGACCAAGAACCTGGGCATTCCTAGTACCATCAATGTTGACTTTCAGGCCAACATTGAAAATATGGATGATCTACAGAAGGGAAATCTCATCGGCAAGATGAATATCAAAGAAGGCTAAATAAAACAACTAACATCAAAAAACATTAAAGGCTATGTTGTGGACGATGCCTTTGTCTCAATAGTTTCGAGGTCATCCAATAACTCATGTAACGTAAAAAAGTTGGTCCATTTTTTTGAAAACATTAAAAGACGTTCGTCTTCATAAATAAAAAAGTCATTCGAAGGAAAAATGATATACTCAATACCATAGTCTTGTAATATTTTTTTTAGGTCTCTCAGGGTCCAGGGATTTACCAGGCTTCTACGCGAAGTGAGCATCATAAAAATATCTAATATTTTTTGCGCCATGAGCCAGCGCGGATTCTCATTGGCCCACAAATCAACAATAATTCTCTTATCAACCGTGAGCATTCCTACTTGATTCGAAGAAATGATTAGATGCCCGGCGGTCCACCCCATAAGTAGATAACGCAGCGTTGTAGAAATGTCACATATGGAAGGCATTCCTCCACAACATGAACCCAAATTAGGATGCGTGTGAAACACAAACATAGCAGGCTTGTTGGCCACCCTGCTATAAATATCAGCAGGCATCATAGCCTCGCTGCCAAAATAAATGTTCTCTCCTGCCCTATAGGGGCTTGGAATGATTTCCACTATCTCGGGTACACCGTTTATCATATTAATGCGGCCGCACCATTCACGGTCATCGTCCAAAAATTTTTTGATGGCACCCCGAACATTGTCCCAATTAAGCAACAGAGTGTTCACAATCTCATTACGCTCCGCCCAGTATTCCTTGAAACTTCTTTTAGACTTGCTGAGCTGTTCCCAGGATTCGAACTCGGTCCAATGTTTTTTTTCTTTTGGGGAAGACTTCCCTTTTGAAACATTTTTTGCGGCTCCACCATCTACACCATGATTTTCCAAAATAATCTCCTTCATCGTTTGAGTTATATGGGCATTGCTAAGCACCTTAGTGGTAACCTGTTTACCTATGTGATTTAGCAGAAAACCAAGTTTGTCCATTTGTGTCTCAACCATTTATTCTTAACAAAACAAAAAAAATTAAAAATCATCGTCGTTTAAAAAGAGTTTGAAGGCAAACGCATCATCCTTAACACAGTTCTGATACTGCGTAGGTCTTAACTCGAAAAAGTTGGTTTTTTCTACTTCATTAAGAAAGAATTTAGTCATCTGAGGAAAAGGGTTTCCCACTTTATAAATACTTTTGCACTGCATCATGAAGCACAAATTATCTGTAAAGTAGCGTATATATTGAAACAGCATTTCTTTTGAAAAACCGGGAACTCTTCCTCTTGCCTTGTCAAAGGCATAGTTAATGAACTCATCCACCAACTCCACAGCCTCCTTCAAAATTTTGTGAATGATCTTTTCCTCGGGAATGTTATACACGTAATTCGAGATAAGAAAACACGCAAAACTACAGTGCATCCCCTCATCGCGTGAAATAAACTCATTATAGCTTACAAGCCCCGGCATAATATTCTGTTCCTTAAGAAACTGGATCGCCACAAAGTGGTTTTGAAATAAAATGCCTTCTACGGCGGCGAAGCCCACCAGCCGCTCACCCAGAGTGTTCCTGTCGGGGTCCATCCACTGCCGCACCCACTGCGCCATTTTTTTTATGATAGGGTGTTTTTCAATGCCGCTAAAGATGCGCTGTTGTTCCTTCTCATCCGGGATCAGCGTTTTTACCTGTATTGAGTAGGCTTCACTATGAACACACTCTTGGGCAGCCTGCATTGTATAAAAGTATAACACTTCCTTTACTTTAATTTCGCGCATAAAATTGGTTAAAAGGTTTTCGATAACAATTTCGTCGGCAACAACAAAGAAGGCTAAAATTTGTTTATAAAATTCGCGCTGTGGCTTTGGCATGGCTTCCCAATCATCAATGTCCTTACACATGTCTACCTCCTGCGCCGTCCACGTCAAACTTTCTAATTTTTTATACCAGTTCCAACATTCGGGGTGCTGAATAGGAAAAATAGTGAAACGTTGGGAATTTTCAATTAGTAATTCCTCCATATTTGAAATAAATATTAACATCTTCAAATTTATTGGCTGCCATGGAGACGTTTTTTATTGAGACGTTGGCATCTGATGTGTATGGAAAGGCGTTGAATGTTGATTTAGATAGACTATCGCAGGCGCAGGTTAAATATACCCTTCAAGAGCTTATTTCCTACTGCAGCGCTCTAACCATTTTACATTATGACTATTCAACCCTTGCGGCGCGACTTTCGGTGTACCAGCTGCACCAGTCAACGGCCTCCTCCTTCTCAAAGGCGGTGAGACTGCAGGCCGCACAATCCTGCTCACGCCTGTCCCCCCAGTTTGTGGACGTCGTTTACAAGTACAAAGCCATTTTTGACAGCTACATTGACTATAACAGAGATTACAAGCTGTCCCTCCTGGGGATAGAAACCATGAAAAATTCCTATTTGTTAAAAAATAAAGATGGGGTCATCATGGAACGCCCGCAGGACGCCTATATGCGCGTCGCCATTATGATCTATGGAATGGGAAAAGTAGTTAATATAAAGATGATTCTGCTGACCTATGACCTGCTTTCCCGGCACGTCATCACACACGCGTCGCCCACCATGTTCAATGCAGGCACCAAAAAGCCGCAGCTTTCCAGCTGCTTTCTGCTAAATGTAAATGATAATTTAGAGAATTTATACGATATGGTCAAAACGGCCGGCATCATTTCAGGCGGCGGCGGTGGAATAGGGCTGTGCTTGTCAGGAATACGCGCAAAAAATAGCTTTATTTCTGGCAGTGGTCTTAGAAGCAACGGCATTCAAAACTATATTATGCTACAAAATGCCTCACAATGCTACGCGAACCAGGGAGGCCTACGCCCTGGGGCCTATGCGGTCTACTTAGAACTATGGCACCAAGACATCTTTACATTTCTGCAGATGCCCCGCTTGAAAGGACAAATGGCTGAACAGAGGCTTAATGCCCCCAATCTCAAGTACGGTCTATGGGTCCCCGACCTATTTATGGAAATACTTGAAGACCAAATACATAACAGAGGCGACGGCACCTGGTACCTCTTTTCACCGGATCAGGCCCCCAATCTACATAAGGTTTTTGATTTGGAACGGTCACAGCACGAAAACGCACATCGCGAATTTAAAAAGCTTTACTATCAGTATGTTGCTGAAAAAAGGTACACCGGTGTTACGACGGCCAAGGAAATCATTAAAGAGTGGTTCAAAACAGTTATCCAGGTGGGAAACCCCTATATCGGATTTAAAGATGCCATAAATCGTAAAAGTAATCTTTCACATGTAGGCACTATCACGAACTCCAATCTTTGTATTGAAGTCACAATCCCCTGCTGGGAGGGTGATAAGGCTGAACAAGGCGTTTGTAACCTGGCCGCGGTAAATCTAGCCGCCTTTATACGTGAAAATGGCTATGACTACCGTGGGCTCATAGAAGCATCAGGCAACGTCACAGAAAATTTAGATAATATTATAGATAATGGCTACTACCCCACAGAAGCCACGCGGAGAAGCAATATGCGTCACCGACCTATTGGCATCGGGGTCTTTGGCCTAGCCGACGTGTTTGCGTCTTTAAAAATGAAATTTGGTTCACCCGAGGCCATTGCCATGGATGAGGCCATCCATGCGGCCCTATACTACGGGGCCATGCGACGATCCATAGAACTTGCAAAAGAAAAAGGAAGTCATCCCAGCTTTCCGGGGTCTGCGGCCTCAAAGGGTCTACTGCAACCCGACCTATGGGTTCGCTGCGGTGATTTAAGTTCCTCCTGGGAAGAACGCGTGGCACAGACGACGCAGGGTGTGTTAACGCGGAAAAGCTGGTGGCAACTACGGCTGGCGGCCATGCAGGGAGTTCGAAATGGATATCTCACGGCCCTTATGCCCACCGCAACCTCCTCAAATTCTACAGGAAAAAACGAATGTTTTGAGCCCTTTACCTCCAATCTATATACACGTAGAACGTTAAGCGGGGAGTTTATTGTTTTAAATAAGTATTTAATAGACGATTTAAAAGAAATTGATCTTTGGACAGAAGCCATTCAACAGCAGCTACTAAATGCAGGAGGTAGCATTCAGCACATTTTGGATATACCGGCCGAGATCCGTGATCGGTATAAAACCTCTAGGGAAATGAATCAAAAAATTTTAACAAAACACGCGGCCGCACGAAACCCCTTTGTGTCCCAAAGTATGTCTTTGAACTACTACTTTTATGAACCTGAACTAAGCCAGGTACTTACGGTGCTCGTCCTAGGCTGGAAAAAAGGTCTAACCACCGGTTCCTATTACTGTCATTTTAGCCCTGGAGCGGGTACCCAAAAAAAGATTATAAGAAACTCTGAGAAAGCGTGTAATGCGGACTGCGAGGCGTGTCTTCTGTAGGAGTCTCGCGGTAAAAGAGCAGCGGGGACCATATGGCAAACCCCAATAAGAGGATAATGAATAAAAAAAGTAAACAGGCATCCATTAGTTCCATATTAAATTTTTTTTTCTTCTATATAATGGAATATTTTGTTGCGGTAGACAATGAAACCCCCTTGGGGGTTTTTACTTCTATAGAGCAATGTGAAGAAACGATGAAACAATACCCCGGCCTCCATTATGTCGTTTTTAAGTATACTTGTCCGGCGGATGCAGAAAATACAGATGTTGTATATTTAATACCCTCGTTAACCTTGCACACCCCCATGTTTGTAGACCACTGCCCAAATCGTACCAAACAAGCACGACACGTATTGAAAAAAATAAACTTAGTGTTCGAGGAAGAGTCTATTGAAAATTGGAAGGTTTCAGTAAATACTGTATTCCCCCATGTTCACAACAGATTATCTGCGCCGAAATTTTCCATCGATGAGGCCAATGAAGCCGTAGAAAAGTTTTTGATACAAGCAGGGCGACTCATGTCTCTGTAAATGTCTCCTTTATGGGCGATGTCTCTGTAAGTGTCTCCTCCTTTACTGAGGAAGTCTCTGTTATGGGCAAGAGGTTTGAAACAACGCAGGGGCTCTGCTTAATCTGCTGTCTCACAAAGGGAATCAAACTACCTGCTTTCGTATTTTTAATGTAGTAATTACCCTTGTTATGGTGAATTTTAAGACCATAGCGTAGACCCAGCACTTTATTAATGAATTTTAAAATTGTTTGGGGATCCGTTTTACTAGGCTTTTTAAGCTTAAACTCAAAGCTGACCGCGCTTAAATCATACTGAACAAATTCATCAACGAGTTTTGTCATCAATTGTTCATTGGTCAATATATTAGGGTCCTGAACGCATTTAAAGCCGCACTTAGTTAACAGCATAATGGCGTACATATGGGATTGAAAGCTATAATTAAATTGCAGATCATGATGCTCTGCATGTTGCATGGCCCATTGATGGAAGTTTAATTCCTGAGTTTGTAACATAGTGAGCGATTCGTATACTGTTTTTCCGCGGCTTATTTGGACACGGCCAGTGTAGTTCTGTTTTGTCATAAAACTATTGTATTGTTCAACAAATTTGGGGGTAATTTTATGACCGTGCCATGCATAAAATTCGAGTAGTTTATATTTTTCATACGCAAATAGGTCTTGCTGGTCTACTGTGATGCCTTCCTTTAAGTTTTGTTTAATTTGTAAAGCTTTATTGGCATCAATGGTTTCAGCCGAGGCAATGTTTACATAGTCCTGGTGCTTAATTTCCATTTTAATGCTTGTATACTGTTTGACTGTCTCCAGCTTTTCACCCGTCAGTATAAACACCTTAGCGCCGGTGTCGGCGACCTGGTTAATAAATCGTGTTATAAAGTGATTTTTTGATAGATGTTGTATCCGCATTGTTTCGAGCCATAGATGGTAGTATGGAGTTTTATAATATACCGGCCTACCTGTTTCCTTACTATACGTGAAGGAAAGCTGGTGATTGTTTATGGTCTGAAAGAGGGTGTCACGTTTTTGTAACGTGAACATTTCAATGTCTTCGATGGTTTCTGGGTAGTAATTTTGTTTCCCCTGTAAGCAGATTTTATAACACTTACTTTTTAATTCACGCACGCGGCCCAACATTTGGCAACATGTTTCTACGTCACACGACATATTGTTAAAAAAGCCGTATAAAACATCAAATCTCTTATCTTCGTACGAAACACCCGCCGAAATCGTGGGCGTATAGATAAGGATATCAACGAGCCCCCAATAATACGATACATTATTAAAATGGGATTCCCGTTCATGAGCAGTGCTTTTAGAACTATAAAACCCGATTTTTTTTTCCGGAAACTTTTTTTGGATAAATGATTGCAACAGCCGGGCCTCCATTAATGAATTTGTAGGGATAACAATTTTTTTGTCTTCTAGCAAATCCTTTAAAAGGTTATTTAACCAAGTTTCTCGTGAAGAGGTGAAATAATACGTGTCATGCTGGGCCCTTTTATATTGATTCCAGTGAAAGAAGATAGGAACATCCCCGCGAAAACGCTGTAGAATATTATACGTTCGATTTCCTAGGTTTGCGTCCAAGCATATAACATAATTTGCCGTTTCGAGCATCCACATGAAAATGGCAAAAGAGGGAGCAAAGTATTTGTGCAGGCCGCTATTGAATTGATTAAAAATCGATTCTACTTCATCCAAAATAAGTAGGTCCACAGGCTCGGCTGTGGAGGTTAGCCGGAAAAGTGATTCCACCTGAACGATGACTCTTTCGTAGCTGTCCAAATCTCCAGTTACTTCACTGTATAATGTGAAATTTGGTAGCCGGGACTGTATATTTTTTGAGAAGATCTGTCGAAATGTCACAAACCGTATGGTTTGTTGTTTTGAAATAGAATTATTGCCGTAGTATTTTTGCAAATAGTTGCGCAGTTGGACGGTTTTACCTATTTTCATTTGAGCCTTTACAACAAGCGTAGGGACTCGTTCATATTCTCGCATACTACTTTCATCATAGATGTGTTTTTGAGTATCAGGCAGTTCTTCAAAGAGAATGGACTCATGGACCTCTATGCTCTTTGTCATCACTTGGTCCACATATGTTTCCACAAAATTAGTTGTACCGGAAAGGCTGCCCATGAGAAGGCTATGTTTATTGTCATGGCGACAGTGTTGATACACTTTGTTTCCCGTGACTCTTAAAATTAGGGTATTATCCTTATCATGCATACGCTTACATATTTCGCAGTAACTTGGACTTGTACGTTTAAACAATACTAAATTTTTATGAACACGGAGGAAGCAATGATTTTTACATAGTGTTCCTGCAAATTTTAATACCTCTTCAAGTTCACTTTGTTGGATAGTATCGCAGGAACTCGGTGTTGTTTCTTTTACATTTGTGAAGATACAAGGTAAACACGTCGTTTCAAAGGGGGTTGCTATGAGGGTATCACTCTTTTTCGTGGTTGTACTGGTCTCAAACACCTCTGCAAGCTCTTCATTAAACATTTTAACACGCATGCTACCTTTTTTATGAGACCCTATGATGCGAAAATTTTGAATGCTTTTGTTGACCTGAGGGTCAACAAAAGGATAAACGTGTTTGGGAAGATTTTCTAACACTTTGGATGTAAAGGCTTTGGCCTCATTATTGTTTAATACTGAGTATGTATAAAGTATGATATGAAAGGAGTATTTAAGTTCTCGCTTTTTATTTAACCCGATAGAATCTGTTAGCAAAATTTGTTCACGCGTTAGATTGATGTTATAAGGTAAAGAATATGTCTCGTAAAATACATCTATGATAACGTTAATTATCATGTCAAGGATGTCATAGACATTGTCATCGTCATAGACACTGTCATCGTCATTGACATTGTCATTATCATCAGAGTATGACTTATTTACCGGGAAATCGATGTCAAATTTTAAGCGCTGAGGCAAAAATCCAAATACCACTTCGTGGAAACACTTCTGCTCAAAGGGCTGAGCCGCCTCCCACTCCCAAAAGTCATCACGATTTGAAAAAACCCTAAAAAGATTATTATATTCATCTCGCACCACGAAGTGATTCTTTAAGGTTTCGAGAGAATATTTATCCTCTACGGCTTCTCCTTGGGAGTTACAGCGAAGAAACTTGAATGTTTCTTGCATTTTGATAATTAAAATTAAATCAATTATGATGCGTCAGTGGCCGCTGACGCAGCCGTCATAAATAAAGCGGCGGCCGTATTATAACGACTAGTTGGCCGCTATAGGACGAGCCATATAAAAATGAATTCTTTTAATTAGAGTTAAGTATTGTTGATTGTATAATTCATTATGGTTGAGCCACGCGAACAGTTTTTTCAAGACCTGCTTTCAGCAGTGGATCAACAAATGGACACTGTAAAAAATGACATAAAAGACATCATGAAAGAAAAAACATCTTTTATGGTGTCATTCGAAAACTTTATAGAACGTTACGATACCATGGAAAAAAATATTCAAGACCTTCAGAATAAGTACGAAGAAATGGCGGCCAACCTTATGACCGTCATGACGGATACAAAAATTCAGCTTGGAGCCATTATCGCCCAACTTGAGATTCTGATGATAAATGGCACTCCACTTCCGGCAAAAAAAACAACGATTAAGGAGGCTATGCCCCTACCTTCATCAAACACGAACAATGATCAAACGAGTCCTCCCGCCTCAGGCAAAACAAGTGAAACACCTAAAAAAAATCCCACGAATGCAATGTTCTTCACGCGTAGCGAATGGGCATCCTCGAAAACTTTTCGAGAAAAGTTTTTAACACCAGAAATTCAGGCCATATTGGATGAGCAGTTTGCAAACAAGACCGGGATCGAAAGATTGCATGCCGAGGGTCTTTACATGTGGAGAACCCAATTCTCTGACGAACAGAAGAAAATGGTCAAAGAGATGATGAAGAAGTAATATTTTTGGTAAAAATATTTTTATCAAAATTTTTTTACCAAATAATAAAAAATATTTTTTACTTTTTTTTCTTCATAATATACATAGAATGCCTACAAAAGCTGGCACAAAAAGTACCGCAAATAAAAAAACAACGAAGGGCTCCTCCAAATCTGGTTCTTCCAGAGGCCACACCGGCAAAACCCATGCTTCTTCGTCCATGCATTCCGGGATGCTCTATAAAGATATGGTAAATATTGCTAGATCTAGAGGCATTCCGATTTACCAGAATGGATCGCGTCTTACTAAAAGTGAATTGGAGAAAAAAATTAAACGGTCAAAATGAATATAATCAGGAAACTTAAGCCTGGAACAATTAGCCTTGTGCTGGGACCCATGTTTGCCGGCAAAACTACGTTTCTTATTCATTGCATTTACATGCTCGAACGTTTGGAAAAAAAAGTAGTCTTCATAAAATCTACCAAAAACACCCGAGACAAAACTATTAAAACACACTCCGGTATACAGCTACGACCCAAACAATGTAAAATCATAGAAAGCACACAGTTATCTGACGTGGGTTCTCTCACCGATATCCATGCAGTTGTCGTAGATGAAGCGCATTTTTTTGACGATTTAATCACATGCCGCACTTGGGCAGAGGAAGAAAAAATTATTATTCTTGCGGGACTCAATGCTTCCTTCGAGCAGAAAATGTTTCCGCCCATCGTTCGTATTTTTCCTTACTGCAGCTGGGTTAAGTATATTGGCCGCACCTGTATGAAATGTAACCAACATAATGCATGCTTTAATGTGCGTAAGAACGCAGACAAGACGCTTATCCTTGCGGGAGGAAGTGAACTGTACGTAACATGTTGTAACAACTGTCTAAAAAATACATTTATTAAGCAGTTGCAACCTATTAAATATTAAAAATCTTATACAATAATGGATCATTATCTTAAAAAATTACAAGATATTTATACGAAGCTCGAGGGTCATCCCTTTCTTTTTAGCCCGTCGAAAACCAATGAAAAAGAGTTTATTACTCTGCTAAACCAGGCCTTGGCCTCAACGCAGCTTTACCGCAGCATACAACAGCTGTTTTTAACGATGTATAAGCTAGATCCCATTGGGTTTATTAACTATATTAAAACGAGTAAACAAGAGTATTTATGCCTGTTAATTAATCCTAAACTCGTTACTAAGTTTTTAAAAATAACGAGCTTTAAAATTTACATTAATTTCAGGCTGAAAACTTTTTATATAAGTCCTAATAAGTATAATAATTTTTACACCGCTCCCTCTGAAGAAAAGACTAACCATCTTCTAAAAGAAGAAAAAACTTGGGCAAAGATTGTTGAAGAAGGAGGAGAAGAATCCTAAGTCGCTTACATTTTTTTTTGCTATTTTTATAGAATGTACACGCATGTTGATGTTGTCGGAATAGCTGAAGCCTCAGCGGCCCTCTACGTGCAAAAAGATAGGGATCGCTACTTAGACGTGCTAACAACCATTGAAAACTTTATTTACCAACACAAATGCATCATAACAGGGGAAAGCGCCCACCTACTCTTTTTAAAAAAAAATATTTATCTTTACGAATTTTACTCCAACAATGTGGCGGAGCACAGCAAGGCTTTGGCGACCCTGCTTTATAAACTTGATCCGGAATACCTCACTCGTTACACAGTACTCATTACCAAAATTCCCAACCATTGGTATGTGATTAACGTAGATCAGCGAGAATTTGTGCGCCTATATGCCATCCCGGCAGTTAAACAACACTTACCGATTCCCATTTTACCCTTCTATTGCACCAGCGCACTCACCCAGCAAGAATTGTTTTGTTTAGGACCTGAACTGCAGTTAATACAAATATATTCCAAGCTCTGTAACCCCAACTTTGTCGAGGAATGGCCTACGTTGCTCGACTACGAAAAAAGCATGCGGATGTTATTTTTAGAACAGTTTCCGCAAAGATTGGAAATGACGGGCGGGAAGAAGGAGGAGAAGGAAAAGCATGAAAGTATCATTAAAAAAATAATACTAGAAATGGTCTCTACCCGTCAGCGAATCGTTGTTGGGGGTTACATACAAAAAAACCTGTACAACCATGTACTCAAGAATAGAAATCGTTTACAGCTTATTACGAGCTTAAATATTTATGAAGAAAAAGATATCATCCAGCAATTTTGTGATTCAAATGGACTGAAGATCAAAATACGTATCAACAATCCGCTCTTGCCTACAAATCCGGAATTACGGCGTTTGACTATTTATTTTAATCATAATAATGATGATGATCAGTCATATCTAATAGTAGATATGTACAACACGGGAAGCTATGAGCTAGTGCCTACAAATCAGATAAACACGCTTGATGGCAGCTTTTTAATAGGAACACCCTTCGTGCAAGCGCGATTTTTGTTGGTAGAGATCTGGGTGCTTATGCTTATTGCGCAGCAAACTAAAAAGGACACCAAAAAAATAATACAATTTTTTATAAATCAATATGAAATGCTTATGAATAGTCCTTGGCCCAGTATGGAGGCCCTTTTTCCCTCAAGCAGTAAAAGATATTTAGGCAACTATGTAGACCCTAACGCGCTCATAAAGTGGGCACAACTCAAATTAAAAAGAATACCGCCTTTTTATCCTGGAAAGCCGGATGAAGAATCATGTTAAGCCGATTAAAAAATCATGTTAAGCTGGTTGAAAAATCATGTTAAGCTGGTTGAAAAACTCTTGGTGAAAGCACGGATGTAATATTAACATTGGCCGCTCGCATTTCGTGTTGAAATACGATGGAAGAGCGACGGCTATCTACCATGCCGATATCGGCCTGGACATCACAGTTCATGCACTTGTAGATGGGATGACTCGCGTTATAGATGGCAGGCTCGCCACAGTTTCTACAGATGTAGGAGATGCAGCCATCCGAGTCGTCGTGCGATTTTTCTATGATGGTTTGCATGGCGCCCTGCGCCGTAAGCACCCAATGCTCCATTTCTCCCAGACGAAGACCTCCGTGCGATCGTTTGCCGTCCAACGGCTGGCCTGTGAGGGCATCCGTGGGCCCATAGCTTGCAACGGCGTATCGGTCATCCAGCACAAATTTTTGCAGGCGCTGGTGATAGGTCGGTCCTATGAAGATGGCCGCATCAAAGTACTCGCCGGTCTGGCCGTTGAACATTTTTTGGCATCCATTGAAGCGTAGACCTTCTTGCGCCAGTCTTTCTGAAAGAAGCTGCACATTAATAGGCAGGAATGCGGTGCCGTCTGTTACCACCCCCTGTAGGGCATTTGCTAGACCAACCGTGGTTTCTATCATTTGACCGTTGGTCATTCGGGAGGGATGTGAGTGGGGGTTTACAATGAGGTCGGGCTGCAATCCGTCCTCTGTGAAGGGCATGTCTGAAGTGGGCAGGGCCAGCGCCGCAATGCCCTTGTTCCCGCTGCGAGAACTCATTTTGTCGCCTATATTGAGATTTCTTTCATAGCGCAGGCGCATGAGGCCAAAGATCTCGTCATTAGGCCCATGGGGACGCATCACAGCATCCACGACGGCCGGCTCATCGAAGCCGTACATGACAGACCGGTCGATGTATTTGTTGAGTTCGTCTTTTTCGCCCCGTATTTTGGCCACTTTTCCTATAATGATGTCGCCCTTTTTGACCACCGTTCCTACGGGCACGAATCCATCTACAAGCTTTTCGTAATTAGCACCAGGCTTAAGATTTTTGGTGATTAAAGGGTCGGGCTTCCCAAACGACTCTATATCGCTTTCTAATTCTACTTTTTCTTCTCGGTAGAAGGTGCCGGCAAAGCCGCCCCTGTCAATAAAGGACTGCGACACGATCACAGAGTCCTCCTGATTGTAGCCGCCGTAGATCATATAAGCCACAATGGTATTAAGCCCGTTGGGTATGACATAGTTATGTGCTATGGTCTTTACAAGCGGCATTTCATTGTAAAACTGGAAGAAGCGGTTCATGTCGACACGATATGGCCAGCTAAAGCAATACCAGCCCCCCGTTTGCCGGCCTTGGTTTGTTTCATAGGTAACACGCGCAGGTTGGGTACAGTTTGCGTAGGGGGACACTAGGGCGGCAAGGCCCAAAATAGCTTGGGGCACGTCCACGTGTGTGAAACGACGCGTTACATCATGTTTATGTTTGCGTAGCTCGATGATGGAGAAGGCAACAAGACAGTTTTCCGCTTCTTCGGGGGTAATGAATTCACAGATGCCCTGCGCTACGAGATCTTCAAGTGTAAGTGTTCCGGCTAAAATGTCTTTTGCCATTTGAGACGTAAATCGCGTATTTTGAATGAAAGGGATTTTATGTTTTTCCCAGTCTTTATCGCCTTTTTTTCTGGCCTCTGCGGCCTTGTAACAGGCTTGATTGTATTTTTCAATATTATTATCTACAATGAGCAGGGGGCGGGTCAGCCTACCGACGTCCAACCAGAATTCTACTTCGTCTACCATGCTATCCCAGTAGATGGTGGTATGGGGATGCACAATCTTGCCCTCACGGCGAAACATTCTATACCGCTGAGCAAGCTCAAAGGCGTTGGTGCAGCAGCCAATCCATTCTCCGTTGATAAATACGCGCGCTAGGCCCTTTCGTACAATGTCCTTGTTGGAAACATCGGCTAACTGTTGAATGGCCGGATCTGATAGAAGGCGTTGTTTTAACGAAAGTACTTCTCCAGCGGTACAGACATTGGCGGTAATGGCTAACTGTTTAGACATACCTACTTTTTCACCAGTATCGGCTGACTGGGCTACGCAGATGTATCCAGGATAAGATGCGTGCACGCGACGCATCATGTCAGCCCTTTCTGTTTGTTTGGATGCGTTGGTGGTGTTATGAGTATTTACCGTACGCAACGCTGAAATCGTATTTAATAAATTTTTTCTTTCCAAACTTTGAGTAGATACTCTGTTTACAATGGGGCGCTGTCGCACCATGATGGTTTTATTTCCTGAAATGATAGACTGTTCCATACTGCGATTGAGATCGGAGGCGGTATTTTTTGATAAAGCGGCAGAAAATGCCTCGATAATGTTTCGCTGGGTTAGCTCCTCAAAAGCTGTTTGTTTAAGAAGTTCTTTGAACCCATTGATGATGGGTGCTATCACGGATGTATTAAAAATAGCCTTAAAGGCCTTAGCAAGCGAGACCCCTGAGCCGTGCACCCGCTTGGTGCGGTAGCTATCACGGTCCGTGGGTGGAAACACATTCATAATGACAAGAAGTATTTTATGAATAAGCAGGCCTAAAAAGCGCAGTTTTCGTACACGTGTATCTGCGGTTTGGCCCATGTGTGGCAACAATATTTTGTCTAAAATAGTAAGTTGTCTTTCATTCAAGTATTGTACCGCATTTTCATCGCTTTTGTAAGCAGATGGGTTTGAGACAAATTTGGAAACCTTCTCAGATAAAAACTGGATAATTTTTTCTCGGTTCAGCTCGTGTTGGACCGGTTGAAATATGGGGTCTAAAACATGAATGGATTTTTCCAGAATTTCTATCATGAAGGTATTCACAGGGGAGTTGGATTCCAGATCGAATACCACTTGCTCAATGATGCTGTCATCGCCTGTCATTCCAAACATGCGAAAGATGAGATACCAAGGTATGCGAAGTTTTGAGAACTTGGTGCTATTGATTTCAATGGTAATGGCACCAGTAGTCATGTAGCGTATAATAATTTGAGAGCTATTTTCGAAGGCACCTCCTGGTTGAGAGATAAACTCGCCGCGAATGATTTCATTATTCCCTTGTTGCATGGTATGGTAATGGATGTGAAGCGTGTTAAAGCGGATGTTTTCTAAGAGGTCTACGACCCATTCCCCGCCTCGGGCTATAAAGTAGCCGCCGGGTTCATTAGGGTCTTCTCCTATTTCTTTTTTTGCGGTTTTTGATAGGTGATGAGTGTGGCAGCGGTTGCTGCCCCGCATGATGGGAAATGTGGACACCTGAAATGGAGGAATACTTGCTCGTTTTACCTCCTGCCGACCATTGCTGTAGTGCGCCGTTAAAATAACCTCGGCGGCTAGATTAACAGGGCCCGAATAGGAAAGGCCACACAGGCGTGCCTTATTAGGTAGTAAATTTATCTTGTTTCCCTGTGAATAGTTTCGATGTTGCGGGCGTTCAATGTTCACATCTGTAAAGTTAAATTGGATCTGAACCGATTCCCGAAGCTTATCTATTTCAGTATGGTCGCGTTGGTCTTTATAAGTAATATCCACGTTAAACATTTGTTTTACAATTTGCGGGATTCCATTGTCCATAAGATCGTCGAAGCTTTTGATGTTATACCCTATCAATCCTGTAGAGTTTACTGCAGCGGAGATAAAGCTCAGCATATCAGCCTCTGTAAGCTCCTCATTATCCACGGTTTCAATGGGGCCGTAGGTTATTTGCGGCCGCAAGGGTTCCATAATTATGAAGTACTACATTAATATTCAGTTATTCTTTAAAATAAATCTTTATTTATAAATCTTATTTATAATATAAGAATGCCTTATTCAAGAGATATCACAAAGTTTATCACGGCAACGGAACCAGAGGTGGGTCTCCCCCTGTTGGCGCTGCAACACTCCAAGTCCGTCATAGGGGTTATTCTTCTTGTAATAAGTTTGTTATTTATTTTCATTGGCATTATTATACTATCGGTGAGTAGTAGTCATACCACAGCAGCCTCTATATTTATCGTATTGAGTCTTATCCTAGGTGGCGGTGGTTTTTTTCTTATTTATAAAGATAATTCTTAACCCACATAAAATTTGAAAAAATATAAAGTAAGAAAATGTCCAATTACTATTATTACTATGGCGGGGGGAGATATGATTGGTTAAAAACAGTAGAACCCACTAATTTTTTAAAAATCGGGTTGCCTTACCAGGCACACCCATTACACCTCCAACATCAGGCAACTACTACTCCCCCATCTATCCTAGAAAAATTTAAACGAGCAGATATTCTTCTTAATGAGGTGAAGGCCGAAATGGACCCACTCATGTTACAACCAGAAACCGAAAAAAAATTATACCAGATATTGGGTAGTATTGATATGTTCAAAGGTCTGCGAAAAAAAGTAGAATTTACGTACAATGCTCAAATTGTTACGAATGCTTGGCTTAAAATGTATGAGCTGCTAAATACCATGAATTTTAATAATACATCTCAGGCATTTTGCAATTGTGAGCTTCCAGGAGGGTTTATAAGTGCAATTAATCATTTTAATTATACAATGATGCATTACCCTACTTTTAACTGGGTAGCCTCCTCCCTTTACCCCAGTTCGGAAACAGATGCCCTGGAAGACCACTATGGTCTTTATCAGTGCAATCCGGATAACTGGCTGATGCAATCTCCTTTACTAAAAAAAAATATAGATTATAATAACGGGGACGTAACCATCGCTAGCAATGTAAAAAACCTAGCGCTTAGAGCCACACAAAGGCTGACGCCCATCCATCTATATACGGCTGATGGGGGTATTAATGTAGGACATGACTACAATAAACAGGAAGAATTAAATCTTAAGCTTCACTTTGGTCAAGCCCTTACGGGTTTGTTGAGTCTTAGCAAAGGCGGAAACATGATACTCAAACACTATACCTTAAATCATGCATTTACTCTTTCTTTAATATGTGTATTTTCTCACTTTTTTGAGGAACTATACATTACCAAACCTACCTCCTCTCGGCCCACAAACTCTGAAACCTATATTGTGGGTAAAAACAGATTACGCTTATTTACCCCCAAGGAAGAACAAGTCCTTCTAAAACGGCTAGAATTTTTTAATGATACGCCCCTCGTAGACCTAAGTCTTTACCAAAATTTACTTGAAAGCGTTTACTTTGCCGTAGAAACAATACATCTAAAACAACAAATAGAATTTCTAAACTTCGGAATGAAATGTTATCGACATTTTTATAACAAGATTAAACTACTTAACGATTATTTAGCTCCGAAAAAAAAGATTTTTCAGGATAGGTGGCGTGTGCTTAATAAGCTTTATGTTCTTGAAAAAAAGCATAAACTTAAGCTTTGTGCCTCCTAGGGATCTGTTGCTTAATTTAACAGATGCAATCTTAACAGATGTAAACTAAAAAGTGTGTTCATACAAGGATTGTATTTATGAATATTTATTAACATATAAGGTTGTGATGTAACACTGTATAACCTATATAACTACACTATGAAGCACGGCGTATAATAATTTATATTGAACACGATGTTGACTCATTTATTTGCAAACAAATATTTGTTTGCAAGACGTTTGCATGCATTTACTAATATGTTGTTGACTAGTTTATTTGCAAACTAGATGTTTGATTGCAAACTAGATGTTTGCACGTATTTATTTGAACTAATATACACTCCTTGTTTTATTTGTTATATACACAGCATACATAAGTGTATATTGTTTACACTTATGTTTATAACTCGACGTAATAACATTTTACACGCTTTTTTTTTGCAAATCTTAATAATATTGTATGATAAATCAAACAATGTCTTATATATGTGGTTTATTATTTTAGGCGCCGCAAGATGTACTCCATTCTCATTGCATGCTTGGTGTTATTACTCTGTCTAGTTATATATGTCGGTCATCGTGCCGATCATGCACGAAAATATTTAGAAGGAATGTGGCATGGAGATCCGGTTTTTCTAAAACAGTCGGGGCTACAATCCTTTTATCTCTACATACAACCTGACCATACATGTTTTTTTAGCATTGTGAATAAAAATGGTGAAAAGCTGATGGAAACCAAAATACCTTGTACGATAACAAATAAAATATATATGTTTTTTAAACCTATTTTTGAATTTCATGTTGTGATGGAAGACATACATAGCTACTTCCCTAAGCAGTTTAACTTTCTGTTAGATAGTACAGAAGGTAAACTTATTTTAGAAAACAATCACGTTATTTATGCTGTATTGTATAAGGATAATTTCGCCACCGCACTAGGAAAAACGGTTGAAAAATATATAACACAAAATTAATCATGTTTTCTAACAAAAAGTACATCGGTCTTATCAATAAGAAGGAGGGTTTGAAAAAAAAAATAGATGATTATAGTATATTAATAATTGGAATATTAATTGGAACTAACATCTTAAGCCTTATTATAAATATAATAGGAGAGATTAATAAACCAATATGTTACCAAAATGATGATAAGATATTTTATTGCCCTAAAGATTGGGTTGGATATAATAATGTTTGTTATTATTTTGGCAATGAAGAAAAAAATTATAATAATGCAAGTAATTATTGTAAGCAATTAAATAGTACGCTTACTAATAATAATACTATTTTAGTAAATCTTACTAAAACATTAAATCTTACTAAAACATATAATCACGAATCTAATTATTGGGTTAATTATTCTTTAATTAAAAATGAGTCAGTACTATTACGTGATAGTGGATATTACAAAAAACAAAAACATGTAAGTTTATTATATATTTGTAGTAAATAATATTTTTAATTACTTAAAATTTTTATATATAAGTTTTTGATACTATATTATAAAACATATGTTCATAAAATGATAATACTTATTTTTTTAATATTTTCTAACATAGTTTTAAGTATTGATTATTGGGTTAGTTTTAATAAAACAATAATTTTAGATAGTAATATTACTAATGATAATAATGATATAAATGGAGTATCATGGAATTTTTTTAATAATTCTTTTAATACACTAGCTACATGTGGAAAAGCAGGTAACTTTTGTGAATGTTCTAATTATAGTACATCAATATATAATATAACAAATAATTGTAGCTTAACTATTTTTCCTCATAATGATGTATTTGATACAACATATCAAGTAGTATGGAATCAAATAATTAATTATACAATAAAATTATTAACACCTGCTACTCCCCCAAATATCACATATAATTGTACTAATTTTTTAATAACATGTAAAAAAAATAATGGAACAAACACTAATATATATTTAAATATAAATGATACTTTTGTTAAATATACTAATGAAAGTATACTTGAATATAACTGGAATAATAGTAACATTAACAATTTTACAGCTACATGTATAATTAATAATACAATTAGTACATCTAATGAAACAACACTTATAAATTGTACTTATTTAACATTGTCATCTAACTATTTTTATACTTTTTTTAAATTATATTATATTCCATTAAGCATCATAATTGGGATAACAATAAGTATTCTTCTTATATCCATCATAACTTTTTTATCTTTACGAAAAAGAAAAAAACATGTTGAAGAAATAGAAAGTCCACCACCTGAATCTAATGAAGAAGAACAATGTCAGCATGATGACACCACTTCCATACATGAACCATCTCCCAGAGAACCATTACTTCCTAAGCCTTACAGTCGTTATCAGTATAATACACCTATTTACTACATGCGTCCCTCAACACAACCACTCAACCCATTTCCCTTACCTAAACCGTGTCCTCCACCCAAACCATGTCCGCCACCCAAACCATGTCCTCCACCTAAACCATGTCCTTCAGCTGAATCCTATTCTCCACCCAAACCACTACCTAGTATCCCGCTACTACCCAATATCCCGCCATTATCTACCCAAAATATTTCGCTTATTCACGTAGATAGAATTATTTAATATGTACTATATATTAATTATTTAACCTTTCAAGCTGGTCTTCATTTAAATTTAAAATCCACTAATAAAATGTATTTTCTAGTAGCAGATCATCGAGAACATCATGTGATTCCTTTTCTTAAAACCGATTTCCATCACATGCATCAAAATCCTATACAAAAAAATCAAGCTCTCCTAGAAATCAAACAGCTTTTTACTGGAGATTATCTCATCTGCAAAAGCCCTTCTACCATTCTGGCCTGTATTGAACGAAAAACCTACAAAGACTTTGCGGCTTCTTTGAAAGATGGACGTTATAAAAATCGCCAAAAAATGCTGTCGCTGCGAGAACAAACCAACTGTCAACTTTATTTTTTTGTAGAAGGCCCGGCATTTCCTAACCCTCAAAAAAAAATTAATCACGTTGCCTATGCAAGCATTATTACTGCTATGACGCATCTTATGGTTAGAGATCATATTTTTGTCATTCAAACGAAAAATGAGGCCCACAGTTCCCAAAAGCTTGTGCAGCTTTTTTATGCCTTTTCTAAGGAAATGGTGTGCGTCGTTCCCACCTCCCTCACCCCCACGGATGAAGAGCTATGCATCAAGCTATGGTCTTCTCTTTCTGGTATTTCAGGCGTGATAGGTAAAATCTTGGCAAACACTTGTTCCGTAGCTCATTTGGTTCATGGAAAGCTTTCATCGCAGAATATTGATCAGTTAAAAACTCCCTCCAACCGACCATTCCCCAAAAAAGTAAAACGTATGCTTATAAGCATTAGCAAAGGAAATAAGGAGTTAGAAATAAAATTGCTCTCGGGGGTTCCCAATATCGGGAAAAAATTAGCTGCCGAAATTTTAAAAGATCATGCGCTTCTTTTTTTTCTAAATCAGCCCGTAGAATGCTTGGCAAATATACAAATCGTTCAAAAAACCCGTACGATTAAGTTGGGAATGAAGCGAGCCGAAGCGATTCATTATTTTTTAAACTGGTGTGGCTCTGCCCATGTAACCGATGATAGCCAAAATATCACAGAGGCGTCGCGGTCCACAATGCAGGTCGCGACGCAGTCCGCCGCAATACAGCCCGCTGCAACGCAGCCATTGCACGAAGTATCAGATGATGCATCATCAGATGCTTCATCACCCGTAGGGTATCAAACATTATCTAAAGAAATGTTATTGAACACAGCCTGATGTTAATAATTCACTACATCTAAAGAAATGTTAACCTCGATACTAAAAAGTCATTGAACACAACTACTGGGGCGCTAAGTTGTCCAACACATCTAAAGAAATGTCAACATCCTCGATGCTAAAAGGGTCATCGAGCCGGTCAATAATGTCTTCCCCAAAAAGTCCGGGAGAACTGTAGGCCGAGATGTCGTCCATGGAGCTATCTTCCCCAGAGCACACAAAGTCCTCTCCAAAAATCATAAAGTTAAATGCACCGGGCTTACTTAACAGCTTTTCGCTTTGAATAATAGTGTTGAGTTCTGTCAGCGCAAACTCTCTCACAATATTCACAACCCAGGAGGGCTCTTTAATTTCATACAGCGTTAAGAAACTTATACATAAAAATTCTATAGAGTAAAGCAAGGCGCTGGCAGGATCTGTTACCCGTAGGTGTTTAAATGTAGTGTGATATTCATTCACAACGTTAGGCAGCACCTTTTCCAAATCCTCCTTTTCCTCGTACGACAGGTGCTTTACAAGCCTTTCAACATGTATAGGAGGCTTGTTAAATGTACTAACGTGCCGCAAACAGTTATAATTATATAAGAAAATACGTACGGCAGAGTCGACCGCCATGAGCCTTGGATCATCCATTGAGGTAGGTGGTGGCGGGGCACCCTGGCCTTCCCTGATGTCTGCGTAGGAGCGCCCCTCCATGGCCCCTATGGCCTCTATCACAGCAGGACTGATATCCAAAATCTTGGCCGTCTTGATTATTTTTCCGTAATCGAAAGTCCATGGCTCCTGTGGAGGCTTGGGTTGTGTTTCGGTGGAGGGCGTGGTCATATCTTTCTTTATTTGAATAGAACGGATCGACATCTTTTCCTTATCGTACTGGTCTTTATAATTATTATAATAGTCATGAACTAATTCGGGTTGAGAAAGATGATCGTATATAATATAGGTAAAAAGTCCGCACTTGACACATTTTTTATCCTGGAAGTCGTGTAATCCTCCCTTGGGGCAGCGTGACTCGTAGAAGGCATAAAAGGTGTTAAATTCTAAGCTCGCCTTTAGGGCTGTTTGGACCTTTTTTATGTTTAATTGCCCCACCTCATGTTGTAGCACGTGGCATACAGAACAGCGTAGATCGGCAAGTGCATAATGGTTGTCAATTTTTTTTATGACGTCTTTGCGTGTTACTTCAATCTCGGCGGGTTTCTGCGAACTGTCTACGGCCTTGTAAACGTAAATGGTCCACTTATGAGGAAGCCCCCTTTCATCGTATAGGGTTGAAATGGGAAGCCTTTTATACTCAAACAGCCGAGTCCGTTGGTCGGCTCTTCCTGTGTTAGGATCAAATATGTTATAAAATCCTTGCTGAGCAAGCAGGGCCTTTTGCTCGCCATAAGCATTTTCGTACGTTTTGAATTCTGCAAGTTCGGAGTTAAAATTAGGTGCATTTTGTAAATACTTAAGAAATAATTCATAGGCTCTAAGGTAAATGAGAGTTGAGGTTTTTTCCTCATCCCGTCCTCCCCACCACACCCGCAGGCTTTCTTCTTGAAAATAGATGTCATTCAGACGCGTCAACTGCGTAAAATCAGGCCGATATTTAGAGGTATAAATTTTATCATAAAATTCTTTTTGCGATAATAGCTCGGCCGGGGTACGTCCTATCACGGTTTTAAACTCATATTCAGCCTCCTTGGGAGTCCGTGGTTTGTGCATAGGGATGCTGCCGTCAATACGGGCCACTGTGGCAGCATAATCATACATGGGGTCCAGCAGAATCTCTGTCAAAAGTACCTTGGTGTCGTCCTGCACGCTAAGCCCTTGTAGCCCATTTTGGTGGATAATTTTTTTGAAAGCCTCCCGAAAATTATTAGCAATCCACTGATCCGTAATCTCAGATAGCTGATTTATTATACCGCTATATTGCTGCATCATTTTCTCCAAAAGAAAGGTCACGTATGCATTCAAAGAGCTATCCGCCTTCATTCCATGAATGGTAATCGTAAGAAATTCTTTATTTTTTTGCGAGCTATAAATGAGATTCAAAATATAGGCATAGATGTAGATCACAGCATACAGCTGCGTTAAAGGATCGTAATCCTCTTCCTTTTTAATATTTTCGATGCTATACACGAGCGGCAGGCAGACATTTACGGCTATATTGGCAAACTGTTTCACGTCTACAAGCTTTCCAAAGTGGATAAACGTGCAGGCCTTCATGGTTTCCTGCCAAATAAAAACACGGAGCTTACTATTAAGATCGCCGATGATGCCCACATCTGCCGTACGATCCTCTTGAATAAAATGGGCCAGCTCTTCGCCACAAATTTTGCAAAAGTAGGAGTAAATAAGCCCCTGGTTGTTTTCTTTCTCCTTGTTTATTCCTGAAAATTTCATTAGCTTGGTTCGCATGGTGTCGTAGGACGCTTCTGCCGCTTGAAGCTGTATAAGCATGTCCACATGGGGACAAAGCAGCTTAAACCCGCAGGCTTTGCATAGATTCCAATTGGTGGTATTGTTTTTTTCCTTGTAGAGTACACGAATACTTTCTAATACTTTTAATAACTCCGCGTATTGAAGACCCGAACGCAACTGTTTTACCAGCTTGAGATGAGCACATGCATTTTTTTCTTGGAGTTCCCACTGTTTTTTAATGTTTAGGTATTCTGTTGTAATAAGTTCTGCCTCCTGTTTCCCACAGGCTTTAATGACTTCTTGAAGGATGCTGTTAGGGTCATCCACTTTACCCTCCATTGTAAGAATTTCACGTATAGCATCCGACTGCACCCTACCTATTTTTTCTTCCATAATTTTAAAATACTGTCTCGCCTGGGTAATGACCTCTGTGAGCTTCATGTCCACCTGCTGCAGAATCATTTGCTCCTTTTCACGCTGTTCAGCATGTTGTAAAAACTTTTGTTCTACAGGGTTCCAAAGCACCTCCAAATAGCCTGCTCTATATAGGTCATAAAGCAAGGGCATGTATCCCGATGTAAAAACCGGGGACACCGAGTACATCGTAGACAACTCTTTTAAAAAAAATATCACGCGCTTAATGTTCTCCTCCGGTTCAATCTCCTCGGTTTCAACGATATTAGATATATGACTGCCCTGATCCTCACGGTCTAGCTTTCGGTGTACCATCTCCTCTGCTAGCCGATTAATGAGCCAGCTATGCCCGCCGCTCCGCAAAAACTTATAAAGTTCGATATACTGGTGCGTAAACTGGATGATGTTTTCCTTGGTGGTTACGACAACCCCTTCTCCGTTTTTTTTCCAGGTTTCTTGATCCACGCATTTCATAAATACTCGAATAAAATTGGTCAAATTGGCTCCTGAGGCGACGTAGCCCAAGGTTTCAGGCGAGAAGGAGCCTATCTCAGCCATACGCATAAAACACTGCGGGGAAAAAGTTTTTAGCCGCAACTTAAGTCCATAGATTTCAATGGGGGCTTCTGCGGGAACGGCCAGGTGCGTCCCATTAATTAAAAAAATTTCTTTGCGTGTGCTAGGGCGAACACGTAATTCCTTTTTTTTTTCACTCACGATGGGGACCACATCGGGGTCTACCAGCAGTTGACGTATGTAGGCCTCTATGGGCATGGATAGATCGGGCAGCTTTGACTGCTCGGCGCGAACATGGTTCACAAAATCTTTTAGAGTGAAAAGAAAGTCTATTAAACGTATGTTTTTTATATCATTAGACCCTTTAAGGGTAGAGTAGATTTCATCCACTAGTGCCTCGATTTCCTCATTATTGAGCGATAAGATATCTGTGCCACGGTGGACTATTTGCGCGATCGTAATTACTTCCTCCATTAGATAGAAACTGAATATTATATTTAAAATAAATACAAAGGGTCGCCGGAGGAAAAGTCAAAATGGGCAGGTAGTTCGTACACCAAAAAGTTTTTTTTTTCTGCTAGCAAAAGCGTGTCAATGATTTTAAGCTGATCGTTGATCAATTTTTGGTTTAACTCTTTGTTATTATCAAGGTCCTTCGCATAAACCGCCATATTTAATAAAAACAATAAATTATTTTTATAACATTATATATATGTCTAGAGTGAGCAAGGGCGAGGAGCTGTTCACCGGGGTGGTGCCCATCCTGGTCGAGCTGGACGGCGACGTAAACGGCCACAAGTTCAGCGTGTCCGGCGAGGGCGAGGGCGATGCCACCTACGGCAAGCTGACCCTGAAGTTCATCTGCACCACCGGCAAGCTGCCCGTGCCCTGGCCCACCCTCGTGACCACCCTGACCTACGGCGTGCAGTGCTTCAGCCGCTACCCCGACCACATGAAGCAGCACGACTTCTTCAAGTCCGCCATGCCCGAAGGCTACGTCCAGGAGAGAACCATCTTCTTCAAGGACGACGGCAACTACAAGACCCGCGCCGAGGTGAAGTTCGAGGGCGACACCCTGGTGAACCGCATCGAGCTGAAGGGCATCGACTTCAAGGAGGACGGCAACATCCTGGGGCACAAGCTGGAGTACAACTACAACAGCCACAACGTCTATATCATGGCCGACAAGCAGAAGAACGGCATCAAGGTGAACTTCAAGATCCGCCACAACATCGAGGACGGCAGCGTGCAGCTCGCCGACCACTACCAGCAGAACACCCCCATCGGCGACGGCCCCGTGCTGCTGCCCGACAACCACTACCTGAGCACCCAGTCCGCCCTGAGCAAAGACCCCAACGAGAAGCGCGATCACATGGTCCTGCTGGAGTTCGTGACCGCCGCCGGGATCACTCTCGGCATGGACGAGCTGTACAAGTAACTGTGCCTTCTAGTTGCCAGCCATCTGTTGTTTGCCCCTCCCCCGTGCCTTCCTTGACCCTGGAAGGTGCCACTCCCACTGTCCTTTCCTAATAAAATGAGGAAATTGCATCGCATTGTCTGAGTAGGTGTCATTCTATTCTGGGGGGTGGGGTGGGGCAGGACAGCAAGGGGGAGGATTGGGAAGACAATAGCAGGCATGCTGGGGATGCGGTGGGCTCTATGGTCTAATACGGCCAAAGCCGCGGGTTTTTTAATAAACTAACATTTAAAAAAACTGTTTTATTAAAAATTATAATACTTTTATTATATATGGAACATCCATCTACAAACTATACTCCCGAACAGCAACACGAAAAATTAAAACATTATGTTTTAATCCCTAAACACCTTTGGTCTTATATTAAATACGGAACGCATGTCCGGTACTACACCACACAAAATGTTTTCCGAGTCGGTGGCTTTGTGCTTCAAAATCCCTACGAAGCCGTTATAAAAAATGAGGTAAAAACAGCAATAAGACTGCAAAATAGTTTTAACACAAAAGCGAAAGGGCATGTAACGTGGGCCGTCCCATATGATAATATTAGCAAGCTATATGCCAAACCAGATGCAATTATGCTTACCATACAAGAAAATGTTGAAAAAGCTCTTCATGCTTTAAACCAAAACGTACTGACGCTCGCATCAAAAATACGTTAAATATAATTTTTGTAGAGGATAAAAAGCTATTTTAGCTAAAAAATAATTCATATACGTTTATGCAGAGGAAGAACGGTGGCTTTCAAATTCAGATTGCATCCACGTAGACCGTAGCGTTTTTTTTGCTTCTGGTTTATATCGTAAACCGTAATAAACATCATCATTTGTATCCGTTGGATCTTTTTCCCACTCCGGATAAAAAATCGGTTTTCTTTTTTTTTGGTCGTTTTTTGCAGTAAGCTGTAAATTAAGGGAATATAGCTTATCGAAAAGTTGTTCCTGATCCATATAAATAGCAGCATATATTAAAAAAAAATAAAAAAAGACGCTTCAACGAGTCAGTACCACTGCTTGCCAACGATTTACGTTGGTTGGTGCATTATGGTGATATAGTAATGAGTGCCTGCACAAGTGCTTGCACAAGTGCCTGCACAAGTGCTTGCACAAGTGCTTGCACAAGTGCTTACACAAGTGCTTGCACAAGTGCCTGTACACATTACTGCATCGCCAAAGCACCTGCAATGCCTACTTCCTCAACAGAGTACGATAACTAAATGCTTTTAAGCACCGCTTGCGTCGATGTGTCCTTCGGGGCAATCGGGTTCAATTGGATCCAATATTATTAGTCATAATTACCTAATACTTATTCAATTTTATCTTTTTTACCTTGTAAGATTTAAACAGCGTTTTAGCTTGTTTAAAGCAACGTTTAAAACAAGCTAAAATGCTGTTTAAAACAACGTTTTAAACAAGTTAAAACAAATAAGCTTATAAATATACCATGACAAAATTAGCCCAATGGATGTTTGAGCAGTATGTCAAAGATTTAAACCTAAAAAATCGGGGGTCCCCCTCGTTCCGCAAATGGCTCACATTGCAACCCTCACTGCTGCGCTATTCGGGTGTGATGCGTGCTAACGCCTTTGACATCCTAAAATATGGCTATCCTATGCAGCAGTCAGGTTATACGGTTGCTACGCTTGAAATCCACTTTAAAAATATTAGGTCTTCCTTTGCCAACATTTACTGGAACCGTGATAGCGAGGAGCCTGAGTACGTCTGCTGTTGTGCCACCTATCAATCACACGATGGCGAATACCGGTATCGATTTGTTTGGTACCAACCCTTCATAGAGGCTTATAATGCCATAGAGGCGGCCCTGGATCCCCTGGAAACCATTATCCTGAACCTCATTGCGGCACGAGATCTAGACTTCGTTGTTCACATATTTCCTTATAATAAGGGCCATGAAGACTATTTGGCCTCCACGCAACTTATTCTCAAAATCTTTATTGCGACGCTTTTAATGGACATTTTAAGAATTAAAGACAACACGTTGGACGTTCACTTAAATTCCGACTATATTATTGTGATGGAGCGGCTTTGGCCTCACATAAAGGATGCCATAGAACACTTTTTTGAAGCCCATAAGGACTTACTAGGGTACTTAATTGCCTTTCGCAATGGGGGGAACTTTGCAGGAAGTCTTAGACCCTCCTGTGGGCAAAAGATTGTTCCCCTAACGATTCGAGAGGTCCTACAAATGAATGATATTAATTTAGCCGTATGGCGGGAGGTGTTTATTATGCAGGAATGTTCCGACTTAGTCATCAATGGGATAGCGCCCTGTTTCCCCATTTTTAACACGTGGACGTATTTGCAAGGCATTAACCAGATTTTTTTTGAAAACACGTCTTTGCAGGAGAAATTTAAAAAAGATTTTATTGCCCGAGAGCTTTCCAAAGAAATTATCAAGGGCCAAAAAACGTTGAATGACAAGGAGTTTAAAAAGTTAAGTCTACATCAAATCCAGTACATGGAATCCTTTTTACTTATGTCAGATGTTGCCATTATGATTACCACAGAGTACGTTGGCTACACCCTTCAATCCCTGCCGGGTATTATTTCGCGATCCAGCTATTTATCCCCCATCGTGAAAAACATTTTGATGGACGAAGACTCTTTTATGTCCCTACTATTTGACCTATGCTATGGCGCCTACGTGTTGCATAAAAAAGAAAATGTGATTCACGCGGATTTGCACCTGAACAATATGACCTACTACCATTTCAACCCAACCAGTTTTACAGATCGCAACAAACCAGGAAAATACACCTTAAAGGTCAAGAATCCTGTGATTGCCTTTATAACCGGGCCCAAAGTCGAAACCGAAACGTACGTGTTCAAGCACATAGATGGGTTCGGCTGCATCATTGACTTTAGCAGAGCCATTATGGGGCCTAACCATGCAATTAAGCTTGAGCGGCAGTACGGCCTCGCTTTTGTAAACACCTTTTACCGCAATCAAAGTGAGCATATCTTAAAGGTATTACGGTACTATTTCCCTGAAATGTTAACCAATCGCGAAAACGAAATACAGGGGGTGATTTTATCAAACTTTAATTTCTTTTTCAATAGCATTACTGCCATTGATTTTTACGCCATTGCTAGAAACCTACGTAGTATGCTTTCTTTGGACTATTTACACACCTCTGAGGTGAAACGAAACGTAGAGATTTCGCAAACATTTTTGGATACATGTCAATTTTTGGAGGAAAAGGCCGTGGAATTTTTGTTTAAAAATCTTCATACTGTCTTATCTGGCAAGCCGGTTGAAAAAACGGCCGGGGATGTGCTTTTACCCATCGTATTTAAAAAATTTTTATACCCAAATATTCCTAAAAATATATTACGGTCTTTTACCGTAATAGATGTATACAATTATAATAATATAAAGCGTTATTCCGGCAAAGCTATACAAACGTTTCCACCCTGGGCTCAAACCAAAGAAATCTTGACGCACGCCGAGGGTCGTACATTTGAAGATATTTTTCCTAGAGGAGAATTAGTTTTTAAAAAGGCTTACGCAGAAAACAACCATTTGGACAAAATTTTACAGCGTATTCGTGAGCAGCTTGCTAATGAAAATTTGTAAGGCTTGCAGTTCTTGTATGGTCAGAACCTATGTCGATGGAAACATTATTTTTCGCTGCAGCTGCGGCGAAAGCGTTCAAGGGGATAGTCAAAACTTGCTCGTCTCTAGCAAGGTGTACCACACCGGGGAAATGGAAAATAAGTACAAGATTTTTATTAAAAATGCACCCTTTGACCCCACGAATTGCCAAATAAAAAAGGATTGCCCGAATTGTCATTTAGACTATTTGACACAAATCTGTATTGGAAGCCAAAAAATCATTATATTGGTGTGCCGCTGTGGCTATATGAGCAACAGAGGATAAACCATATCATCCCACCGATTTGTGACATTCCTTTAAAACCGTCCGCCTAAATAGTTTTCACACCTTTGGTGGCAGACTATTTTATAAAAAATAATGTTGGTTCATGAAGATAAAGTGTGCCAAAGAAACTTTTATAAACAAATGATTAATGTAGGTGCTAGTCGTGTGTACTTAAACAGGGTATTCTATAGCCAAGTATTTTCTATAGCCAGTATTAGTCAAGCATTTAGATGTCAGGGTATTTTTATAGCCAGTATTTTTCTATAATATGTACAAACTATTCCAGTAAACATATGTGTGTTCTTTATTGAACAGCATCATGGCATTAACAAGTTTATTAAACCGCTCTAATGGGCATTAAATGACAACTCGGTGCTTAGCAAAAGGGCCTATACCTTCTAGCAATTAGGGCCGGGAGGCATTCCCAGCTTTTTTCTATAATCAGCCATACAGTACCCCTGAGCCTCATACATGGGAATGAGGTCCTTCCATTCCTTGTTGGGATCGGCGGGCCAGCTCTCAAATGAGGTGTGAATGTAAGGGTCCTGTTCTTTTTCCTTAATGAAGCGTTTAATCTCCATTTGATGTTGTTTACTTTTTTGTTTGCGGCGGAGCGTGTTCCGCACCAATACGTAAAAAATACCAAGAATCACGCATAAAAGAATTATTAAAAAAAATATCATCATCGCGGGGTTTAAAAAACGATCCCATGCAACAGGAATCGTTCTTAAAACCTTGTCTGGCAGGGCTGTAAACATGAAGTCTCCTCCTATAATCGGGGTGGGGCTGTAGCCTAACAGTTCAAGGTCCTGTCGTTCTAGATACTTATTGGCGAACTGCCCACCCTTTGCCCCCGTTTTTTTATTAATCAAGCAGCGCTGCATTTTCCACCATTCTAAATCTTCAGGAGAAAGCTCAATGCCATATATCAATTTTAACGTTATTGCATCTTTTTCAATATCCTTATCAATTTGGCTGAGCTTTTGAGCTTTAAGCGGGTCTAGTGTGTACTTCCATTTAAACTTAGTGTCCTGTAGTTTGGCTACATGAAATACGGAACATTTTGGTGGGGCCTTTGTGACGCCCTTACACTGCGGAAGTTTATCATTAGGACAGGCGCATAGATGAGACTGCGCCACAGCATCGCGAACTACATCGCAGACGGAGTACATTTTCCTCCTATGTTAAACAATAAATTTTTTTCATAGCTGAAATTTGTGGGCCTATCTTTTCCCTTGCCCGGATAATAATTATAAGGGAGTGTTGAAACATCTGGGAGAGAATTGCTTAAAAAATGGGTTTTTGGGAGGGGTAACTGCGACTGTTGTACGTCGTTGGCCAGGGAGATTCTATATGCCGGGCTAAAGGTGCAACGTTCCTGTGAACAACTTAGTACGCGCGTTGTTAATACAAATGGACTGGTATTAGCAAACCTCGTAAACTCTTCCGGACTTGTTTGTTTTTGTATGATGTTTAGCAGGGAGTCTGCCTTTTCGAGAATCCAAAGCGTCGCATTGTAGTAAAATAAAAATAGCGACTTATCGGCAGGCGTTGCAAAAGCGCCGTATAGAAAATAAAGCAGTAAGTACTGGGGAGACACCACAATAAGGTTATCTTGAATGATAGATATCGCTAGCTCTTTAAACATAGTGCTAAAAAAATGTATGTCGTTCGTCTTGAATATAGGGGGACTATAGTCCATGTAGGGCTCACATATCTCAGTCAGGTGAAGGCCCATTTCTTTTATGACTTCTTCCGGGTTGTACGTCGCTAACACCAGCGCGGGATAGGCTTTGGGCATATCCACGGTAAGTGTTATGTTTTTATCATTCTTATGGTAGGAGTAAGATGGTTGTGGAAATTCTGTTTTCCACTCCGGGACTTTGCAGGTAATTCTCAGCTCATTTAGAGTCTGGTACAGGAGGGCGTATGCCGCAAAGCCGTGTATGGCCACTTGTTTAAAGGGAATTGAAAACGTTTTACTTTCGTATGTCGACTTCACAGGAACAACGGGAATGGGGTAATATTTTTCTATGAGGTTATACCGCTGCAAATCCTTTTTAAACCTGCTAAAAACATCTTCCCTTGGTGGGTTATCAAAAGGAAAGCAAAATGCTAGGTGTAGCCCGGCCCGCTGGTAATCGGGGTGAATGATTTTAAGGTTTTTATACGTTAATGTGGGTATGGTGTTAAAGATATTGGGGGGCATATATGAAAGATCAGCAACCCACACAAAGTCCGTGCGCACCCGCATGGTCTGCACATGGATGGCGCGCACCGTGCCCACCTGCTTGAAGCCCTTTTCATACAAAATGTCAGCAAGTTCGTAGGCGTCCTCAACGTGGTTGGGGGAAAACATATCAAAGTCGGGTCTTTCTCCCTCGGGATAAATTGAGCTGCCTTTAAGATGCAGGGCATAATCAATGGCAATCCCCCCGTACAAAATAAGCTTTTTCTTTATGATAAATTCGCGGACCACCTCCAAAGCCGCCTCAATCTCCACGGCATTTGCCTCACGTTTTTGAGCAATGAGCCGGTACTTAGAAACATTAAAATCAGTCTTTAGTAAAGACGTCATAAATAGTGTTTAATATATATTAAAGGTTTGAATAAAATACTAAATAGTAAAAATGGATGCCCTATTAAAGGAAATAGAAAAGTTATCGCAGCCATCCTTGCAGAAAGAAAACAATGATGTATGCGATCTCTGTTTTATGCAAATGAAAAAAATTTCTAACTATCAGCTTTTATGCGAAGAGTGCGGTCAGCTGAAGGACTGGTTTGAACCTGAATATAATGAAAAATTCACGGTATATTCTCGTCTAAAGATCGTGGGTGCCAATAGTTCCTATCACCAGCGCGATTTGGACAAGGCCAACTCAAGTGACTATAGCTCCTTGCAATTTCATCACATTTTAGAGGAGCTCAAATCCCTAAATGTTAAGTATATGGATGCGGGGCAAAAGCCCTTTCCTATTCAGGTGTTAAAAGAAACTGCTCACAGTTATAACCAAGTACAACAACATCGGGTCATACGCAGCATTACAAAGCTTCAGATCTTAGCCAGTATTCTACGTAGCATTTGTTTAAAATTAAACATTGCTTGTACGGTGGCAGACGCCGCGAGGTTTACTCAACTTAATACCAAAGGGATCTCAAGGGGCATGGATCTTCTGCGCTCCCTATTTGTAGACAATAAAATTACTTTAAACGTTGATTTAAACCCTATAGACAGCTTTATTAATAGTACCTACAGTGCCTTACAAATTAAACAAATCCACCAAGAACTGCAGGAGGAAAATGTTTATAATTTAAAAGAAATTGTTAAGAGCTTTATATTATACGCGGATGAGAAGAACATCGGCGTCGATCTTAACAGGAGAACCGTTGTGATTGCTACGATGTATAATGTTTTACGCCGTGCCTACTACCCCATAGAAATTGATACGGTGGTGTATCAATGTAAAATACGAAAAAATACAATTACACGTGCTCTTAAAATGTATGAGGATTACTACTCCCACTTTAAGTCTCTTTATGAGCAGTATCATTTAAACGCGGCAAAAAAATTAATTTAAACTAAACGTTTAAACTAAATGTTTAAACTAAACGTTAAAACTAAACATTTCGACTAAAGTTTAAAACCTAGTCTAACAGCGGGATGCCCATTTCCCTGGGGTTCCATATTTCAACAATTTTTTGACCTTCGGGTGTTACCTTGATGCAGCGCATGACGAGCAGTGGAATTTTCCTATTAAAGAGTTCTTGCTTAGCTATATCAATAGGACTGCTATATTTTTTTTTAAGCATTGTAGATCCATTAATTGCCAATTGTTGCGCTCTAACGGCGACCAACCTTGTGGCCTCAAAGGTGGTTAAAACGTTGGAGGTAATGCGCTCGTTATCGGGTATAATGACCAATGTTTGCGACGAGGCCTGCACAAAGCCCTCGCAGATGGACGGAGACTCCACGATCTCGTCCTTGTCCTCGGACTCCTCCTCACTGTCGACGAGGTTCTCCTCTTCCGTTTCCACATATTCCTCCACGAGGTCATCCATGATAAGATCCTCGTTGTCATTATCAGCCATATTACACTGTTATCAAATGTACTGTTTAATACGCAAATGGATTTACTACGTTTTAATTGTATGTCTTCATGTGCAGGCTCTAGTGGAAAGTAATTTTCTCACAATTTTTGGCACCGTTACACTTGTGCCCACAAAAACCCGCGATTTTTTTATTTTATATTACTTTTGGAAGTACGAGTTTAACCAGTCGCTTTCAAACCTTATGCGTCTATCTCGCCAAAAAACGCTCACAGCGGTGTTGGATATTACCTTTAAAAAAATAACATTAATTTTTACCACAGAGGGCGTATTGCGTATGGATTCTACGAATAAGCCAGGCGTGCCACTCGATATAGACCCCCAGTTCATTGACCTTGATAGTATTTTAATGGAACTGGATCATTAGGACCTCTCCCGCCCATTTAAATTTTTAGTTTCTACAATAATAAAATGCGCGAGGAATCATGGGAAGACCACGATACCATTCAGCTCACCGCTCAGCGCAAATACCTCGCCGAGGTGCAAGCTCTAGAGACCCTTTTGACTCGAGAGCTTTCAGTCTTTCTCACAGAGCCAGGCAGCAAAAAAACAAATATTATTAATAGAATCACAGGAAAAACCTACGCACTTCCCAGCACAGAGCTACTAAGACTCTACGAGCATCTCGAGCAATGTCGCAAGCAAGGCGCCCTCATGTATTTTTTGGAAAGACAGGGGACCTACTCGGGTCTCATGTTGGACTATGACCTTAAACTCAATACAAATGCTGTTCCCCCGCTGGAACCCCCCGCGCTATCACGGCTTTGCCATCGAATATTTGTGCATATAAAAAACAGCAGTGTGCTGCCTGAGGGCAGCCATAAAATCCACTTCTTTTTTACATTAAAACCTGAAGTGGTTCAGGGCAAATATGGGTTCCATGTGCTCATTCCTGGTCTCAAGCTGGCGGCTTCTACCAAAAAAAGCATTATAGGATCCCTACAGCACGATGCCACCGTACAAAAAATTCTACACGAGCAGGGCGTTACAAATCCTGAGTCCTGTCTGGACCCCCACTCCGCCTCCGTTCCCTCGCTCCTCTACGGCTCCTCCAAACTAAACCACAAGCCCTACCAACTGAAAACCGGCTTTGAGTTAGTCTTTGATAGCTCTGATCCCGACTACATTCCCATTCATCAAATAAAAAATTTAGAATCTTATAATTTAGTTTCTGAGTTGAGCCTTACGAATGAACAGGGAAGCCTTGTAAGACCTGTCTATTGCGCGGCAGACATTGCCGCTGAGAAGGAGGAAGAGATCCCGACCGAGGATCACTCGCTCTCCATATTAATGCTACATGATCCCGAAGCCCGGTATTTACATAAAATTTTAAATCTGCTTCCTCCGGAGTATTATGTAGAGTACCCCCTATGGAGCAACGTCGTATTCGCTTTGGCCAATACATCCGCTAACTATCGGCCCCTCGCCGAATGGTTTTCGCAAAAATGCCCTGAAAAATGGAATACGGGAGGAAAAGAGAAACTAGAAAAACTTTGGAATGATGCCTCGCACCACACTGAAAAGAAAATCACCAAGCGGTCCATTATGTACTGGGCCCACAAACATGCCCCCCAGCAATACAAAGAAATTGTAGAACAAGGCTACTTTTCCATTCTCGCTGAATATGTGTATAGCTATAACGGCATGCTTGAGCACTACATGATCGCCAAAGTCATCTATGCTATGATGGGCAACAAGTTTGTAGTGGACGTGGATTCAAACGGGAAGTACGTTTGGTTCGAATTTGTGCTACCGGGCCAGCCAATGAATCAGGGAGAAATATGGAAGTGGCGCAAGGAGGTAAACCCGGATGAGCTGCACATCTATATTTCCGAAAACTTTTCAAGGGTGATGGACCGAATCACGGAGCACATCAAATACCACCTCAGTCAACCCCATGAAAGCAATATTTTAAATTATTATAAAAAACTATTAAAAGCCTTTGAACGCTCTAAAAGTAAAATCTTTAATGACAGCTTTAAAAAGGGAGTTATCAGGCAAGCTGAGTTTTTATTTCGCCAAAGAAGCTTTATTCAAACTCTGGATACCAATCCCCACCTACTGGGGGTTGGCAACGGGGTTCTCTCCATTGAGACCATCCCGGCTAAGCTCATTAATCATTTTCACGAGCATCCCATTCATCAGTACACACACATATGTTATGTGCCCTTTAATCCCGAAAACCCCTGGACAAAACTATTATTGAATGCACTCCAAGACATCATCCCAGAACTTGATGCTAGGCTGTGGATCATGTTCTACCTAAGCACGGCCATATTTCGCGGCCTGAAGGAGGCTCTGATGCTTTTGTGGCTTGGAGGCGGCTGCAATGGAAAAACTTTTCTAATGCGACTTGTGGCCATGGTATTGGGCGATCACTATGCCTCCAAGCTCAACATCAGCCTTCTTACAAGCTGCAGAGAAACCGCGGAAAAACCCAACAGTGCCTTTATGCGGCTTAAGGGGCGGGGATATGGGTACTTTGAGGAAACCAACAAAAGCGAGGTTCTAAATACGTCGCGGCTGAAGGAAATGGTAAATCCGGGCGATGTCACCGCTCGAGAGCTTAATCAAAAACAGGAAAGCTTTCAGATGACGGCCACCATGGTCGCCGCGTCCAACTATAACTTCATCATTGACACGACGGACCACGGCACATGGAGAAGACTGCGGCATTATCGGTCAAAGGTGAAATTCTGCCATAACCCCGACCCCAGTAACCCCTACGAGAAAAAGGAAGATCCTCGCTTTATTCACGAGTACATCATGGATCCAGACTGCCAAAACGCATTCTTCAGCATACTCGTCTATTTTTGGGAGAAGCTACAGAAGGAATACAACGGGCAGATTAAAAAAGTGTTTTGTCCCACCATTGAGAGCGAAACGGAGGCGTACAGAAAGTCACAAGATACGCTACATAGGTTTATCACAGAAAGAGTCGTGGAGTCGCCCTCGGCAGAAACTGTGTACAACCTATCCGAGGTCGTGACGGCCTACGCGGAATGGTACAACGCCAACATTAACGTAAAGCGCCATATTGCCCTTGAGCTATCCCAGGAGTTAGAAAACTCTGTGCTAGAAAAATACCTTCAGTGGTCTCCCAACAAAACGCGAATTCTAAAGGGTTGCCGTATTTTGCATAAATTTGAAACGCTGCAGCCCGGCGAATCCTACATTGGGGTGTCCACGGCCGGCACACTCCTAAACACACCCATATGCGAGCCAAAAAATAAATGGTGGGAATGGTCTCCTAATCTCTCTGCCCCTCCTGAGAAAGAAGCGTCTGCACCAACTCCTTAGGAAATATCCTTAGAAGCATGTCTTTCGGCAGAGCCATTACCGGTAGCAAAAAAGCAACATTGAGTATATTATATGCCTTAGCCTGCTCATAAGCGTCCTTTTTTTTCATGGTATTTTATGTTTTTATATATTTTTAATTATTTTTTAAATACGATGAACAGTTCGTGCTCCGAAGGCTGTTTACTAAAAATCGGTGTGAATCCGCATTCTTTAAATATGGTTTCCCATTCGGGGATGGTATGGAAATCCATGTCTCTACGAATAGTATGGTGCCCAAGCGCGTCCTGCAGGCTGTGAAGCCAGAAGGCTTCCTGACCTTTATGAAGGTCATACACGATAAGAAAACCATCAGGTTTCAACAGATGGTAAAGCTTGTTAAAATCGTTTATCGTAAGATGATGCGCCGCCATAGGTAACCCTATGAGCTCCACAGAGTTTTCATGCTGGACATCGTCCATATCGGTATAAAACGTTTCACAATAAATGAGACGCTTAAACGAGTATTGATGACAAACATTTATTTCCAAGTAGGTTTGCACCACGTTTTTAGGTATATCGGGAATCATGTTGATTAAGGTTGTTTCGGGAAACTTAATCATCTGACTAGGCTTCATTTTCAACTCTTTAAAGGATTTCCCGGAGAAGTGAAAATGGGTCTTTACGTATTTATGTAAAAATACCTGAATGGGGAGAGGGGGCTCCTCCTCTTCGTCCTCGACGCCTCCCAAAATATTTGGAATTTCCTGACGTGGCGAAAGAAAGTTTATGTCCACGTTTACGAATCCATCGAGGACGGACACAAAGCTTGGCTCTAATCTCCATTCCATATACTGTTTAGAAACGGGAGATAGCATAATCCTAGGCGTTACAATGCACGAAGGATTTTTAATCACCGCATCGTGGTAAGAAAAGTGTATTCCATTTCTTCCAGTATAAAGAAGCCTATGTTTGTCGTAGCAGAAACAATTAAGGCGGTATGCCTCATACATACACTGTTTCAAAGTACAAACACGTTTTAAAAAGGTTTCTGCATTGGCGGAGGCCAAGCGGTTTTGCCATTGGTGGAAGGGGTTCAATCCTACAATGGCCAGCTCGTTTAAAATATCTTCGCGGCGCGCCAAAATCTGCACCATAGAAGAATATTTTAGCATTTTTTTTTCGCACCATTCGCGAAGATGTTTAGCTACATTATTAACCTTATTATTGATAAAGTATACGATGGCATGTTGGAAGCCTTCAAAAATAAAGAGCCCCTCCAAAAGATCATCTGCCAATAGAAGATGGATGTTGGTGTAAGCATTGTCAATATTTTGTAGAAACGGCGGAATGCCTGCCAAAACCGCTTCAGCAAGCATAGCTCCGTTCCGTTGTTTACTGTCCAATAGATTCGTAAGTTTTTTGTCCGCAACAGACACGACGGCTAGGATGGTTGCAATGTCAGAAATGGCGGCTTGCCAGAAATAACCCGAAAAGCACATGCGCGCTTCTTCTATAGATAAAAACGAAAAGCGAGAGGCAATGTCTCCGAGCTGCGTGAGTTGAAGACCTTTTTCTCCTCTGGTTAAAAGGCCTGCCACAATGGCTCGCTCAATGGCTGATGCCAGCGCATCCGTGGGGGGAGGATCCAGCATATCAATCTCCTCTGCCTTAAACACGCCTTCCTTATTTTTTTTAATCGTTTCTACGACAATGCTAAGAAAAATGGCCCCAGGGCCTTCCGTAATGATTTCAGGATACTGCTGCGCTGGTATTTGCTCAAAGACGTGTTTTGTGTAAAGCGGGTAAAAGTGCCCAGGAAATACTCTCCCTACGCGTCCCTTTCTTTGCTCGATACGGCTTTGAGCCGCGGGGCGCGTAATGAGCCCTCCCGCCCATTCAGGGTAGTAGGTTTCAATGCTTCTGTTCCACCCGGGATCTATGACGTACTTCAGCGTTTCAATGGTAAGGCCCGTTTCCGCAACAACCGTGGAAACAATGACCCTTCTTAAAGGTTTTTCCACTTTAGCGGTTAAGGGATTTTTCACCCACAGATTCTTAATTTCCGCTTTCAGGCCAAGGTAGGCCTCATTTTCCTGCGCAATCGCCTCACTATCGATCGGCAAAATCAACATTAACGGCAGCTTTTCTTTGGCAAGGTCCATATTTGCATTATTCAGCAACATCGAAAGGAAGCGTATTTCAGCCATACCGGGCATGAAAATTAAAATATCTGCTTCCGTGGGACGATCATGAATGTTTTCTTTATGAATAGTGAGAGCCGTTTCGCAGGCGGTCTTAATGTAGTTGTTGGTGTTATACAGCGGCCAGTGGGTTTCCACACCGTACTGTCGTCCTTCCACCAAAATAATGTTTTCTTTTCCGATACCAAAATAGGTTGAGTATTTATGGGTATCAATGGTGGCGGAGGTTAAAATTACAAAGGGAATACGCAGCGCCCCTATGCTTCCTCTTTGCAACATGCGCTGAAGCATACTTTTAATATACATGAGCATAAGGTCGATGCCTAGGGCTCGCTCATGGGCCTCATCTATAATCATAAAGGCATAGCGGGAAGCTATCTCATCATCCGTCATTGTATGTAGCTGCGCCAACAGAACCCCCGCGGTTGCATAGATAAGGCCCCGATTGGGTTTTTCCGTCAGAGGCTTCGTCTGGTAGCCCACTGTTTGGCCTAATATCATGTCGGGGTAGTGGGTTGAGGCGCCGATGTCTTTGGCGAGGGTCACTGCGGTTAGGACTCTTGGCTGGGTACAAATAACCGAGCGTCCCAAGTATTTTTGGAAAGAATGCGTGTTTTCATTTCTCAGAATTCTGAACACGTGTACGGGCAGGGCCGTGGATTTTCCGGATCCAGTGCGTGACTTTATAATGAGCACCCGGTCTGCGAGGGAGGTTGGAATGGCCCCTCCAAACTCCGGGAGACGTTGTTTTATCCACGTGATGATGTAATGAATAGGAACATCATTCTTGTGCTCAGCGGGCACGTTATAGAGATGACCAGGCTCCAATAAAGTCGGTTTTCCCATATTCTATTGTTTTAAGGATTGATTGTTCATAAATATTTTTATACTCTGACCAAGAAATTATTTTTTTATTAAGCCGGTTATTTACGTTGTTATGGAACGCGAAGGTCCAGTACTGAAAGTCCTCCGAGTTGTTTAATGTCAAGGGATTTTTTGTAAGATACGAAAAGGCGTGGTGCTGACACCTGGTGCATGGCAGAGACTCGATAAAGTTCAGTATCCATTGGATGGCTTCATATTTTTCTTTCCAGCTAGGAGCGTCTGAAAAAGAGATAGCATATAGATGCAAGGATCGCCAGTATTTAGGTCCCCAATGCAACATTTATAACCTTTTGAAAAATCTCATTCCATATAGAGGTAAATATTTTTTTTCCATGGAGAATTTTTTTGCACTCTTGAAGGGATTGCGCCACATCGTCAAATGTTTTTTGTTTTCCATGTATTTTGGCGTAATTCCAGCCAGTATCTGTGTCATGGTCCTTAATGTCATCCGCTAACTGAAAGGCATGTCCAAAACAGTGGGCAGCCCTTTCAATCATCCCAATGTCTTCAATCGTTCCTGTGCCCAAAACCCAGCCCATAATAAACGCGATCTTAAAAAAGGGAATGGTTTTTTCTGGAGTGTCTACTAACTGACCGGAACCCGCGCTGTTTAGAGAGTGGCTTACAAAGGTACACAGCAGCGCTCCCAGTTGGTTGGGATCCGGAAACCTTGGACAGTGTTCCTTAATCCAGTCGATTTGCCGGCAAATGTTTTGAAATCCTTGCATGGTTAGCGCCAGAGCGCTCATCTGCGCCTTGGCTACGCCAAAGCGGGCCCACACTGTATCTTTATTTCGCCGCTTCACATCGTTGTCAAAGGAGGGCATATCGTCGATAATCAAAGAAGCTACGTGAAAGTACTCCACTGCTAGGGCAGCCTCTGCCGGATAAATAGGCGCCCCAAAGGAATGTTGCAACTGACAGGCCCGAACAATTTCCATCAGGATAATGGGACGGATATACTTCCCACCTCTTAGAGCGTAAGAGCAAGGCTCTGTTAGTTGTCCCTTAAAGTCCCCATCTTCAATAGCATTGTTTAAGATGGTCTCAAACTCTTTACTAAAGGTTTTATAATTTTTAGGATTCAGTGGATGTATTCCATGAAAAAGCGCGACACTACGCGGAGCTGTGATTCTAAAATACTTAGGTTTGCGCGTATAGGATATTAAAATAATAATAAGAACTACAATGATGGAGATATAGATGAGATGCAACATGCTGAGTTGTCTCCCCGCAGGGAATGGTCCTTTTCCGCACTGGTTAACGGTACTGAGGAGGCGTTGAAATCTTTAGGAAAGGTGCTGTCTAGTTTGGAATCTCCAATTCCTCCCGTATATTTAGGTATATAATTATTGTGCCTAGAAATTGTTTGCTTTGAGGTATCAAAATATTCAGCCTGACCGCTATTTCTTTTAGAATAATTCGGTATAGGGCTTGAGTAGTTGGCAATACTCTTAAACCGGGGCACCAAGGTAACAATATTTTCCATATAATGGGTTTGATACGCTTTGTTTAAAAATGGGCTTACCGGCTTTATGCTTGTTAGTTGTGCATTGAGTACCGGTATGTCTTCTAGGATTTGTGGCTTTATAGAATGATTAGCAAACACAGAATGTAGTATATTAGCTACTTGTAGCATGTGTCTATTTGCGGAAAATTCCTGGTATTCTCTGCCGTGTTGCGAATCTTTGGGCGGAAGGGGACCAAGCATCGGCACGCCCGTGTAGGTACTGGTGGATTTTATGAGTTCCTGCTCTATGTTCGGTTTGACATGTGGATTTCCTAAAGGAATACCTCTACCTGCAATCCCTTTTTCTACCGACGCAGGTAGATTGTGCGCTAAACACAAAATATTGTACACGTCTTTGTGCGGAATATATCCGTTATAGTGCTGGCCCGGCATCTGATCGCCAAGGTGCTGCTCATGCTTAATGGTACCCTTTGTTTTGAGTTTAGGAATATCCTCGTACGAAAAAAATTTTGTGTGCTCGCTGAACCTCGTAGACGGAACCGAACTATTTTTTGGGTTTTTTAAGGAAGGCAATGAGGAAGGCTGGGTCAGACAATTTTTCTGTGTGCCCTTTAAGCTAGCCACCTGCGGAAATGTTTTTTTTTCCGTACGAACAACATTGCGCCTAATTAGGTTTTCCGTATGGGTTGAAAAAGCAGGACGATGATTTTTAAAATGATTAAAAAGTTTATTTTTTGGAATGGAGCTGTACGGCTCCAGATCTTGCGCATCGCCGTAACCAATGTTTTTGTGCTGAGGGTTCAGCATAAAAGAAAAGTTACGTAGATCACTGAGTTGCAATCCCTTTTCAGCCTTTTCAGGACTATTAGTGTATTCATTGTATACAGGCGCAGCTCCATTTTTGTTGCCGCAGTACCGGGAATTTAGTATATTATCAGAATACCGGTTATGACGCGGCAAATCGCTTTCCCAAAGAGGTGGATCTGACCTATAATCGGCTAACAGCTTTGAAGCATAATCATGATACATTGTATATAAAAGTTAATTATTATATTGAGAAGGCATAATTACCTCTTGTAGGGGTACAAGAGGCTTTGAATCAGCCGAACTGGCCGGCTTTGAATCGGCCGGCTTTGGACCGGCCGGCTTTGGACCGGCCGGCTTTGGACCGGCAGGTATCTTTTTAGGTTGATCTTCTTCTAGCTCATTAGACACGGATGGGGGAGAAATAGGAGGAATAATTTCATCTCCGCCCTTATATTTGTCGTGGATAGAAGAAACAATTACATCCATGTTTGATTTATTATAAATGTCGTTTAACTGGTGATTTAAAACATAATAATGCAAAAATAATAGGGCTACAATGCATATATATACGTAAATAGCCGTCTTCGTTTTTCGTTTTTTATCCACCGGCGGATTACAAATTGCAAAAAATACAACTAATACCACCGCTGTAATGATTAAGGCCACAATGAAAGGATTTTGAAAGGATGTTTTGAATGGTTCGCACGTATAAATTTTTTCTCCTAAATTATTGATACCCGCAATAAAATCTACATTCATTTTATATATTTATAAATTATGAAAAATTTAAAGTTACATCTCCGCCGGACCAATCATTGCTAAAATTTGAAGATTCTTCAAAAAGGCCCGACTGGTTGAATGTCTTCTGCTCAGGTTTCCAAAAATTTTCCAAGAATGGATTTTGAACAATAGGCTCATCTTGATTTTCTTCTTCAAGGATATTTTCTTTGGTATCAAGAACAGCTTCTTTAAACTCAGGTGTATCTTGATTAAACTCAGGTTTATCCTGATCAATCGCAAGAATATTTTCTTCTTCAGGTATATCCTGTTTAATCGCAAGAATATTTTCTTCCTCAGGTTTATCCTGATCAATCGCAAGAATATTTTCTTCCTCAGGTTTATCCTGACCAAACTCAACAATATCTTTCTCGCTAAATCCGTTTTTAGTGTGAAGTTCTTGGTTTTGAAGAGAATTATCAAAATCTAGTTTAGTTGTTGTTCTAGATCGTGGCACAGGATAGTTATCTGGTGGTTTACTTACTATAGTCCTCGAATGTGGCACGGGATAATTGTTTGGTGACTTGCTGGTTAGCTCTTGGCTTGTTAATAGTTCTTGTTTTCTCAATAATTCCATCTCTACTACTTCTTTTTGATCCGCCGGTGTCTCTTTTTGGTATTCTTCATTAGAAAAATGTTCAGAGGGTAATGTTTCAATAAACTTTGTGAGTGGATAGCTGCTCTTTGATGTAGAAGAGCGTTGAATTTGCTGATAAAGGAGTTGAACAAGTCGCCGGTATTCACTCTGTCTTTTTTCATATTTTTTACGTAGCGTGGAGAGATCCGCTAAGAGCGACTTTTTTTCAGATGTTAATTCTTCAATTTGATGAAGAAGGCTGCGATTGTATGAACTAAGTCTTGCATACGTTTCTTCTAATTCTGTCTCCGGCTCCACATAGGCCTGTTTTCGCAAAAATTTGTTGTATAGTTCCATTCTTTTTTTGAGCAGAAAGGTAAGACTATAATCTTGCATTTCTTTCGTAACTTTATGGTAGTTTTCTTTCCGGTTTTTGATAATAAAGGGCAGCATTTTTTCTGTTGTGATAAAGGTGCCCAAATTGCTAATGTAGTCGCACAGTAGCAATTCCAAGATAGATTCTTTCTTTTCAAGGCTTATAGATTGGCTGTATTCTTTAGGTATGAAAGAATCAACAATCGTTGTTATGAAGTTTGAAAAGTTTAATGTTTTGCTGTTAATTTGGGTAATGTTACAAAAATATTTGTAAAAACTATCTAGCATTTTTTCATAAAGTTTTTTATTTTGTTTAACCCCTAAAATATAGCCCTTTACTTGATACTGATATTCCGTAACAATGGAATGTTTTTTGTATAGTGCATTTTTGTATAAAAAGTTATAAAAAATGTTGATAAAATACGCACCAAGGGTTTCAAAAATACTTATAACGTGGGATTCTTCCTGATCCATTATATCATATGTAATATTATTTTAATAAAAAATTACTGACTAATAACATGCAAAAAAAATATGTTTAAACTTATTTTAAGCTAGCACTTATTTAAAAGCTTGTTTTAAACACGTTTTAAATTGTATGTTAATACAGTTAAAAATTAAGCCGAAATTTGCTCCAATAAGGATTACTTTTATCAATGACCACCTCTTTACTATAAACGGCTTTACATAATTTTAATAAAGCTTTGGAGCCAAAGCTGAAGGCAGTGGGAAGCGGCACTGTACTATGGTAAAAATGTTGCCGATTTTCATCCTCGCGGATGTACACAAGTTTCCTATATCCTTTAAACACAATATGGCTAATTTCTTCCACATACTCCTTATCCTGTTTGGAATAGCGGTTGCTTTGTCGGGAAAAATTCGACATACAAATAGAGGCATTTGTAAAAATGGAAACAAATGCGTTTTTACGAAGATTGGCGGGTAAATCGGTGTCATCTTGGCAGCAAATAATCATCGAAATAAAACAGTGACGATTTTGGTAAAAAAACTTTTTAAAAATTTCTTTTGTAAATAATGGGTGCAGTTCGGCCGCGCAGTCGTCTAATATTAAAAGTAAACGAGGATTAAGATTGATATAGTTTAACGTAAACTTTTCATCCTCTGTAAGGCATAAGTTTTTATACATATGAATGTTCTGTATAATAATTTTTTTTAAAAGTTGCTGATAAAGCGATGTAATCTTTTCTTCTTTTTTTTGGTCCGTTTGTTCAGCCTTTAAGCACTCCACTTTTGCAATATTTTTGTTTTCCTTTTGCTGTATATCGATCGGAAGTTTATGATACAATGTTTTTAGCATATCGATGTTGTTTACTCGACTGTAGATGGAGGACATCATAGTTTGCCGCTGCCAGATGGCCTCCAAAAAGCGTTCAGCGCCCTTGTTGTCATTTTTTTTTTGCTTATCGGCGAGCCACAAGCGGTAGTGAATTAGAGTTGGATGTACAAAACCCTCATATGAACGATTTGAGGGTTCCGAGGGGGCAACCACTAAAATTTGTTCAATATGGGGTTGCAGGATTTTCATAATATGTTTAACGTACACGGTTTTGCCTGTTTTTGAGGGGCCATATAGCACAGTTGTTTTATCTATAAAATGATGTGCTTTGAACTGTAGTTCAGGAATCAGCTTCCCTGAATGGGTCGTTAGGGCCATCTCTATATTATTACAATTCTGCTTTTGTATATAAAATTTCTTTTTCGAGTTTATTATTATTGTTGACCCACATATCTACCCGTATCGTATCATCAGGCACATTGAGCATTTCAAGCGCATTATTTAACTGTTTTTTTGTTTTTATCAGCTCGCTTTCTTCATCGGGGGTTAAATTTTCTTTACTAAGCAGTTGCTTAATTTTTTCTTCGCAGTCGTCTATAAAATCATACTCTCGAGCTTTTTTGATATTTCCAGATGCTTTTTCTAGGTTTTTTAGCTCCTTAAAGGAAAGCAGTCCCTTAATCCCGCTATCCGTGTGAAAGGTTGAATTATAGATGGAGAGCCCCGGAGCATCCGGGCCGGTTTCTTGTATATTTTTTGCTTTTTTGTGGTAAATGGTATTTCGTAGAATCTCTTTTCCTATCTTTAGGTCTTCCTCATGACGGTCCAAAATCCGTTTTATTATTTCATTATTTTGATTAAAATAATTGTAGCGCTCTCTGTTGGCCTTAAAGCTTCCCAGGAGTGTCCAGTTGCCTAATTGAATGGATGAAACCTCTGAGAAAATCTGGTCTTTATATTTATAATAAAATTCATCAACCTTTTGTTGGTTGCTGCTATCCACCACATCATAAATAATGAAGGCAAACTCTAGGTCGGGCTTTTCTGGGTAGATGCTTTCCGTAGCGGCCCGCAACTCTTCGTAATTATCCTCAATGTAATAATTCCACTTATAAAAAGTATCCTGAGGTGGAATATGCTGCGAAAGATATCTAGTAATTTTTGTGTTAAAGAGAATGGGTTTAAACGCCCTCGGATTTTCAAGCATATGTTTAATGCTTTGATGAAGTTCTATATTTTGTAATATGTGGGCTGCTGCCCTATAGCCCTGTGGGGTTTGGGTAATTGCATCAATATCGGCCTGAAGCTCATTAGGCACATTTAATGTTTTTTGCATGATGTGTAAAGGGATGCGCTCAGGATCTGCTAAATCGGTGTATTCTGTGCTTGCACAGGTGTTTGCACAGGTGCTTGCACAGGTGTCTACATTGGTATCTGCACACATGCTTGCACAAGTGTTTACATTGGTATCTGCACAAGTGCTTGCACAGGTGTCTACATTGGTATCTGCACAAGTGCTTGCACCTGTGCTTGCACAAGTGCTTGCACAAGTGCTTGCACAAGTGCTTGCACAAGTGCTTGCACAAGTGCTTGCACAGGTGTCTACATTGGTATCTGCACAAGTGCTTGCACAAGTATACGCACTTTGAGCATGAAGATTAGGATCAAACACAAAATGTTCTCGTAAAAAGCTATCGATCGTTGTTTTAGCTTCCTTGCTTTTCTGCGTCTGGGTTTTGCAGCTATCCGCTATAGATAAAATTGTATTTACTACCGATTCAGAGGGAACATCATTAGTTTCCTGTTTCAAAGTATCAACTAACGTTATTAGCTCACTGAGAAGAGTTTTGGTCGTGTGGGTAGGTTTTGAATAGGAAGGCATCCATTCCTGCAGAGCTTTGAAGACATATCCAATAAAGCTAGTCATTATAAGACGTCGAATATACTGCTCCCGCAAATTTGTAAAAGAGCAAAAGGCCACCCTGCTATCATTTTTGAACTGTTTGTAAGGGTTCGTCCTTTGATAAAGCTGTTTAAGCGTTTCTTCGGATATTTCAGTAGAGGGATCCTCCAATACGTTTTTGAGAAGCTCATCAATATTAAATTCTGCCATATCTTAGAGTTTATTATATACATATTAAAGCTTTAATATAAGGGGGGTATAACAATGGACGAAATCATCAATAAATACCAAGCTGTTGAAAAACTTTTTAAGGAAATTCAGCAAGGATTGGCCGCGTATGATCAATACAAGACCTTAATTAGTGAAATGATGCACTATAATAATCATATCAAGCAGGAGTATTTTAACTTTTTAATGATTATTTCACCTTATCTTATTAGGGCGCATAGCGGAGAAACGCTGCGAAACAAAGTAAATAATGAAATTAAACGTCTTATTTTGGTTGAAAATATCAATACCAAAATATCTAAAACGCTGGTAAGTGTTAATTTTTTACTACAGAAAAAACTTTCAACGGACGGGGTGAAAACGAAAAACATGTGGTGCACCAATAATCCCATGCTGCAGGTAAAAACAGCCCACAACCTTTTTAAGCAACTATGCGACACACAGTCCAAAACTCAATGGGTACAAACCTTAAAATACAAGGAATGCAAGTATTGTCATACCGACATGGTGTTTAACACCACGCAGTTTGGGCTGCAATGTCCTAACTGCGGTTGTATTCAAGAATTGATGGGAACCATTTTTGATGAAACACATTTTTACAACCATGATGGGCAGAAAGCAAAGTCAGGTATCTTTAACCCTAACCGTCACTATCGGTTTTGGATAGAACATATTCTTGGTAGAAATCCAGAACAAGAGTTGGGGACCAAACAAGATCCCTGCGGAACCAAGGTGTTGCAACAACTAAAAAAAATTATTAAGCGCGATAATAAATGCATCGCGCTTTTGACGGTCGAAAATATTCGAAAAATGTTAAAAGAGATAAACCGCACAGACTTAAATAATTGTGTTTCTCTTATATTGCGTAAACTTACCGGAGTAGGGCCGCCTCAAATATCAGAGTCGATTTTACTACGAGGCGAATACATATTTACAGAGGCAATTAAGATACGGGAAAAAGTGTGTAAAAAAGGGCGTATTAATAGGAATTATTATCCGTATTATATATATAAAATTTTTGACGCCATTTTGCCTCCAAATGATACCACGAATCGACGCATTTTACAATATATACATTTGCAAGGAAATGATACGCTAGCTAATAATGATAGTGAGTGGGAATCTATCTGTATGGAGCTCCCTGAAATAAAATGGAAGCCCACAGATCGAACCCATTGTGTTCATTTTTTTTAAAGATGAAGATTTTTTAGATGATTTTTTTTAGTTTTTTAAAAAGACGAAAAAATTTTTTAAAAGACGAATATTCTTAATCCCCGCAAATTACTTTTTTTTTAGGTACTGTAACGCAGCACAGCTGAACCGTTCTGAAGAAGAAGAAAGTTAATAGCAGATGCCGATACCACAAGATCAGCCGTAGTGATAGACCCCACGTAATCCGTGTCCCAACTAATATAAAATTCTCTTGCTCTGGATACGTTAATATGACCACTGGGTTGGTATTCCTCCCGTGGCTTCAAAGCAAAGGTAATCATCATCGCACCCGGATCATCGGGGGTTTTAATTGCATTGCCTCCGTAGTGGAAGGGTATGTAAGAGCTGCAGAACTTTGATGGAAACTTATCGATAAGATTGATACCATGAGCAGTTACGGAAATGTTTTTAATAATAGGTAATGTGATCGGATACGTAACGGGGCTAATATCCGATATAGATGAACATGCGTCTGGAAGAGCTGTATCTCTATCCTGAAAGCTTATCTCTGCGTGGTGAGTAGGCTGCATAATGGCGTTAACAACATGTCCGAACTTGTGCCAATCTCGGTGTTGATGAGGATTTTGATCGGAGATGTTCCAGGTAGGTTTTAATCCTATAAACATATATTCAATGGGCCATTTAAGAGCAGACATTAGTTTTTCATCGTGGTGGTTATTGTTGGTGTGGGTCACCTGCGTTTTATGGACACGTATCAGGGAAAATCGAACGCGTTTTACAAAAAGGTTGTGTATTTCAGGGGTTACAAACAGGTTATTGATGTAAAGTTCATTATTCGTGAGCGAGATTTCATTAATGACTCCTGGGATAAACCATGGTTTAAAGCGTATATTGCGTCTACTGGGGCGTCCAGGTATAAAACGCGACTGGCGTATAAAAAGTCCAGGAAATTCATTCACCAAATCCTTTTGCGATGCAAGCTTTATGGTGATAAAGCGCTCGCCGAAGGGAATGGATACCGAGGGAATAGCAAGGTTCACGTTCTCGTTAAACCAAAAGCGCAGCTTAATCCAGAGCGCAAGAGGGGGCTGATAGTATTTAGGGGTTTGAGGTCCATTACAGCTGTAATGAACATTACGTCTTATGTCCAGATACGTTGCGTCCGTAATAGGAGTAATATCTTGTTTACCTGCTGTTTGGATATTGTGAGAGTTCTCGGGAAAATGTTGTGAAAGGAATTTCGGGTTGGTATGGCTGCACGTTCGCTGCGTATCATTTTCATCGGTAAGAATAGGTTTGCTTTGGTGCGGCTTGTGCAAATCATGAATGTTGCATAGGAGAGGGCCACTAGTTCCCTCCACCGATACCTCCTGGCCGACCAAGTGCTTATATCCAGTCATTTTATCCCCTGGGATGCAAAATTTGCGCACAAGCGTTGTGACATCCGAACTATATTCGTCCAGGGAATTTCCATTTACATCGAATCTTACGTTTTCATAAAGTCGTTCTCCGGGGTATTCGCAGTAGTAAACCAAGTTTCGGTACGCATTCTTTGTGCCGGGTACAATAGGTCTTCCAAAGGGATCTACAAGCGTGTAAACGGCGCCCTCTAAAGGTGTTTGGTTGTCCCAGTCATATCCGTTGCGAGGAAACGTTTGAAGCTGACCATGGGCCCCCATCTGGGCCGTGCCCTGAATCGGAGCATCCTGCCAGGACGAATGACATGCACCCAATATATGGTGGCCCACCATATCATGGAAAAAGTCTCCGTACTGGGGAATACCAAAGGTAAGCTTGTTTCCCAAGGTGGGGGTACCCGTATGCGGGCGTACTTTATTGTATTCAAACCCTACTGGAACATAAGGCTTAAAATGCGCATTAAAATGAACCAAATGTGTTTCTTCGATTTGACTCAAAGTGGGTTCGGGGTCGGGTTTCCCATAACTTTTGTTCACATTTTTAATGTTAGAAATCCTGCTATTAAGCAAGTCTTGGGCCAATATAATCTTGTCGGCCTTCCCATCGTTAGCAATAAGACAAAAAGCTCCTCCTGATGCCATATATAATGTTATAAAAATAATTTATTGTTTTTATTAAATATGGCGGTTTATGCGAAGGATCTTGATAATAACAAAGAGTTAAACCAAAAATTAATTAACGATCAGCTTAAAATTATTGACACGCTCTTGCTAGCAGAAAAAAAAAACTTTTTGGTGTATGAATTGCCTGCCCCTTTTGACTTTTCCTCCGGCGACCCTTTGGCCAGTCAGCGCGACATATACTATGCCATCATAAAAAGCCTCGAGGAGCGCGGGTTTACTGTCAAAATATGTATGAAAGGGGATCGTGCCCTCCTTTTCATCACTTGGAAAAAAATACAATCCATTGAGATAAACAAAAAAGAAGAATATCTGCGCATGCACTTCATACAAGACGAAGAGAAAGCATTTTATTGTAAATTTTTAGAGTCTAGATGAGCTTTTACGCAATGTTGTACAGTGTTGTATATATGTCTTGTAAGCATTTGTTGTAGAGTAATAAGTAAAAGATAAATAAAAATGACTATTAAAATAAAGCCCAAACCATTAAAAATATTTTTATCTGTTAGATTTAATTTAATAAATGGCTCATGGAATGTGTGGTGCGCCGCTGCATGAGGTGCGGCCACGGCCGCATGGGATGTGGTCGCATAAGATGTAGCTACATGGGATGTGGCATTTGCTTGCATGTAAGGATCGTGATGTGTTGGGTCTTCATCCCAGCAATAATCGCCATCTTTATCTAGCTGAATTGTATACCCCATTATATATCACTTATTATTTTTTTTTAATGTTTCATGAATTTCATTATAGGCGGTGAAAGGGTCCTCAGGCCCCTTCTGTAAAAGATTATAGAGATCTTCGGACGCTTTATGTTTCGTGCGAATTAAGGCGGGATATAACAAAAGAGAGGGCCCCAGTTCCAAACAAATTTTACTTAGCGGGCTCATATTTTGCACCAAGTTTCCCACTACTTGCGATGTTTCATAACGCATTTTAAAGAGCTTTATCATAAAAGTGTTATGCAGGCCGGTGTAGTCTGGCCTATAGTTAAGGAAGGGGATTTCTCTGGTACCGTCAAACACGATCTCAAGTCCTCTAGCAAGCCCGATCAAAATTTCTTCAGCAATGGATGAGTATCTAATTCCTACATTACGAAGCGTAAGCATTTCTATAACATCATCTATTTCCTGCATAGAGGAATCTATTGTAGGAATTTTAATATCATCTGTGCTGATTTGTTCATTCCCAAGATAGGTAAGCAGCATATTAATTTTTTCTAGCTTTACTAGCTTAGTCTTACGCTCATAATCATGATCTTTTTTATAAAAAGAGTTGGGATCACCGTTGGACCGTAGATGATTAATAAGGCGGTCTACTTGCTTTGTACTAGGTTTAATACTTTTTTCACTATACTCGCTTTCAGCATAGTGGTTTTTACGATCTCTTTTAGAAATAGCTGTTTTTTGAGATGCCTCAGACTCTGCATATTTTTTTCTATGCGTAGAAAGAGAATAACCGCGGTCATTACGTGAACTACTGTTGCATGCAAGGCCTCGGCGCGTCTTACCGCTGCGCACACTGCCATTGCGTATACTGCCATCGCGCACACTGCCGCTGCGTATACTGCCATTGCGTATACTGCCGCTGCGTATGCTGCCGCTGCGTATGCTGCCGCTACATACACTATCACTACATATGCTGTCAGTACATACGCTATCGCGGCGTATGCCGCCGTGTACCTTATCGCCGCCCCTACCCGAGGGTTTTTTAGATATAATACTGTGTGGGGAGTCAAGCGAAAATTCAGGGTCATTAAAGTTAATGCCCAATGACTTTGCCAATCCATTAAGCTCTTCATCAAAATGATCGGTAGGAAAACTTTGTTGCTTGCCCATGACCTGTTTTTCAAGTTCCTCCAAATTGGCTTGCTCATTTATATGGAGATTATTCATAAGCGTCGTAATTCCAGCAAGATTTGCTCCTTCTAAAAATGTGGTGTCCTCCATCGGATATACTATACTATTTAAAAGCTTTTAAATAAAAATGTGTTTGGAAGAAATGCTCTCTTCAAGCGTGTGTAGCTCAGATATAAATGCCTCCTCAGAAAGCTTTCCACCATACTCCTTTCTCATCGTATAGGAGGGCGCCGGTTTAATGTAGGAAATCCACTGGGAGGTAAAAAACCGGTACAACATATTTAGCAGCTCGCGGGCCTCCCACCTTTTGGGCTCCGTATAGTGCACATCAACATAAGAGGCGGCGCATGAAAAGCTGCAAAAGTTGCCGAGAACGCCCATCTCAATCTCTCCTCGCTCATTTTCACGCATATAGGTGGGCACGAATTTTGGGACAGTCTTGAAATAGAGATGACATGTCCAGCATTTAAAGCTAGAATGGGTAACCCATTTGGAAACAGTGGTGAATACGGAGGGTAGCTTTTTTTCGACCTCGGCTTCATCGTCATTCGTATTTAACGTATCGGTGGCAGTTTTTTTGGATTGCAAGCATTCTTCAATGGTAATCCCGGATAAGTATAAAATATTAGGACAATTAGTTTCCATAATTTTGATAGTTATTTTTATACAACATGGATTTAATTAAAGATAAATGGAGGACGAAACGGAACTGTGTTTTCGGTCAAACAAGGTGACGAGGCTTGAAATGTTTGTCTGCACATACGGGGGAAAAATTACCAGCCTTGCATGTTCGCATATGGAGTTAATTAAAATGTTGCAAATTGCTGAGCCGGTGAAGGCATTGAACTGCAACTTTGGCCACCAGTGCCTACCGGGCTACGAATCTTTAATAAAGACTCCGAAAAAAACTAAAAACATGTTGCGCCGTCCGCGCAAAACAGAAGGCGATGGGACTTGCTTCAATAGTGCCATTGAAGCCTCCATTTTGTTTAAGGACAAGATGTATAAATTAAAATGTTTTCCTAGTACCGGGGAAATTCAGGTCCCGGGCGTCATTTTTCCGGATTTTGAAGACGGAAAAAACATTATACAGCAGTGGGTAGACTTCTTGCAACATCAACCCATTGAAAAAAAAATCCAGATTATTGAATTTAAAACGATTATGATTAATTTTAAGTTTCAAATAAACCCAGTGTCTCCCCGCGTCATCATTCATTTAAAAAAATTTGCAGCTTTGTTGGAACACATCCCTACTCCATATCCCATACGTGAAATAAAGCCTCCATTAGAAGACTCAAAAGTATCCGCAAAATTTATGGTCAGTCCGGGAAAAAAAGTACGCATTAATGTTTTTCTTAAAGGTAAGATAAATATTTTAGGCTGCAACACAAAGGAATCCGCGGAGACCATTTATACGTTTTTGAAAGATCTTATCAGCGTACATTGGCAAGAAATTTTGTGCGTGTTACCGGTACCCGATTAAAGAATGTTTTCATTAATAAGGTAATCGACTATGCTAAAAAGAATAACAAGAAAAATACCTTGAAGAACTATACCAAAGTAGGTAGGTTTTCTGCATGTCACGGCATGGTTAAAATTGCTAATAATGTAGTCCACAAAAGCATTGCTCAATACGACTAAAAATAGTAAAAAAAGGATAAGTGCTCTTTTTATATCCATATACTTTAAAACTTATTTTTTACACTAATAATTTCCTGCGGCCGCAATATAAACTGTAGGTCATCTATAACGCCCAGACCTGTTAAAAGTAGAGTACTATGTTTTAAGGGATTTAAAATATCCGCCGCAAGAATGTGAATATAATTTTCAAAGTGGTTTACAGGAATGCGTAAGCGTTTTTTTTTGCACTGCGGTTGGTTTAGGGTCGAATACTGGCAGGAGGTATATATATTAATAAGACCGCGGTCGATGGTTTCAATATCTTCATAGAATTCAATGCGCGGCGTCAAAAGTTTTTTAAGATGTTGACATAACTCATCATACGTGTAGGACTGGAGGGGGGAAAGAAGGGTGTAGTCAAAGTTAAAAATGTTTTTTTGAAGAACCTTTAAAGCATGTTCCGCGTCCGTGGTTTCCAAAATATGTTTTATGGTATGAATGTCATTTAAATCTACAAAGTCTGACAGCTTTGTGTAGAACTCGGTGACGGAGGTTATTTTCTGGAAATCGGTTTTTTGAAAAAGATTTTCAATGTGTTTGCGGGTTGAGTTGCTTTGCAGTCCATACAAGACATCAAAAAATTCAATCAGCAAAAACTTATACAAATGGTTAATATAAAAAGCTTTGTTGGCCTTATTCTGCTGAGGATATGGTTCCTCTAGGGGATATAGAATGGCTTGGTCTATATCCCTAGGATCAATAGTCAATGTTGCGATGGGAAGCTTTTCCAGCGTAGCGGGAAGAGTTTGGGTTGGAGCGTAGTAAAAGTATAGCCCGGTTTTTCCCTCTGAAAGAAAGCCCACAAATTCTTTTTTTATATTTTGCAGCACCGCTGAGGGTACGATTTCGTACTGTTTATACTGTTTGTTGAAAAGGGTAATAAATTTCCAGGTTTCTTCAAAGCTTGCAATCTGGGTGGGCCGCAGATCAAAGTCGATGGGAATGTCGTCATGAATGTAGGATGATAGTCTTATAGGAAAATAAATAGGGCGATCGGTGTCTGAATCGATAAGTAAAGCATAACAAAAGTTATGCCTGTTGATAAGTTTTTTACCAACCGTGTAGCCGGGAATGTTTTTCACGTCATGGATATCCCACCAGTTATCCTTGCACATAAACTCGCTCATAGACTGGATGACCTCCATCACAGGGTCATCTTCGGTAAAAATATACTGGGCCTCACTGTTTTTCAGAAATCTTTTTTGCTGGGTGATGGCCATTGGGTAGATCCCTTCGTCCGTGTCAAAGATAATGGCTATCTTCTTCGATGGGCTAAGAATTTTTTGTATTGTGCTGGGGGACACCTCAAACCCGATGTCGCCCTGTTTATCTTTAAAAAAGACACAGTGAAGGTCGTAGCATATGGCAACAAGGTCCAGAAAGATGTCCTGCCATGTGGTGTCCCATTGAAGCAGTTGGTTTTTTTGTTCAACAAAGGTTTGTAAGATAAGGTTTGCCAGCTCCGCGCCGCTGGAAAACATGTTGCCGGCCCCATTCCCCAAAATATAGTACTGCGGTGTGTTGGCCGCCTTTGCAATTTCAATGGCAAGGGCCTTGGGGGCAAGATCCAAAATTCGAGCAAGGGAATAAAAAAGCCCGGCATTGCTAATTCCAAGCATGGTTTGCTCCACCCCCACAATGCAAAAAATGTCGGGCTCTTTTATCGTATTTAAAAACAGTTCATCTGCTATCTGGTGGGGTAGAAAGGCAATCCGGTTCACCGGTATTTTTTTTCCATAGGACAAGGTATGACGCGATGTTTGTGTATTAAGATCCTCCAGGTCTTGTTCTACAAACGTGTGCTTGGTGAGGCAGGTATTGTTAATATAGAACCGCTTTGTGCCCAGCAGGGCCTTCGTCTTTTGGCAGCACGGCAGACAGTAATTTAGGGGGTGGCGGCCTTCTAGTAGGCTTAGATGAGGGTAGTCAGGATGCGGGCAGCTATAGTAGGCAGGTACCCCCTCCGTGAAATTCCAATACTTTACTAGCTCCTTGCGCTTGGCTGGCGGCATGGACTTCACCTCGGCCTCTGAGTAAATGACGGGTGGCCGTGGGTGCTGGCATAGGACGGAGTAAACCGTTGCCTGCGTGTCGTACTTGCGCAGGTCATACAGGTCGGGGTCCTGTTCTTGAAGCGCACGTAGCTGAGAGGCTCCCTTTCCTTGTTGTTTATCGTGCAGTTGAGAGAGTTTATTAACCAAAATTTTGTCAGGCCCGGTGATCAAGTTATCTAAAAACACAAATAGGTAAACCCAAAGATAGTTAAACTCTTCCTGGGTAATGTTAAACATTTCTATTTTGATATCTGTAACCCTATGGTAGATGCGAATGTTGCGGCCGCCGTAGATTGTTTCCCACCGGGCCGCAACATTTGTGTCAAAGAGGTACGCATACGTGTTTTGGAGCAACGCAACATTGATGTCCATTTTGCGCCCCGGACCGGAGGAAATAATGATCATCCGTTCGATTTCGTGGGGATCATACGAATAAATCCCCTTTTTAAATAAAAAATTGTAGACCCCGGTTTGCTGGAGGCCCCGCACGGAAATAATCCCTGCTTGCTCGTATTCCCGCCAACGACTTTTGAGCTCGGTAAATCCCTTGCTAGAAAGCGTATAGGGCCAAAAGGTGGACACCGACATGGAGCTGATAGAAATTTGGATGTCCTCGTTGGAGGGAAGGGGCAGACTCCCTCCACGAGGAAACGCGGCAGGCCCCATATCATTAATTGTATGAATAATAGGATTTATGAAATTATTTAGGGTGGACACCACGGAGTTAAAGTCGTGGCGCTCGTTTTCTGACCAATTGCTTTCGATAAAGTAGTGCCCATTATTTTGTATGGTAAGAATAAAGGCCTTTTTATTGATAAAGCGTATTAAAATAATAGTGGGTACACGGAATGTTTTATTGCTGAATTTTTCAGGCTCCGTGGAAGTTATGTGGTGTTTGGAAACCACGGTGGGACCTGTTTTACTATAAAAGAACACCACCAGCTGAGGAATATCGGGAGTAGCTGGAAATAGGTCGAAAACATTGCGCACATTAATTTGAATATTTACGAGGGGTGAAATTTTAATCATTGCCGAGGTGACGGCCAACGTGCCGCGTGTTAGTCTATTCCCCTCGTACTTGGCAATGACTTGTTGTGCTCTGGCATACGTAAAGTTTATTAGTTTTTGCTCTAGGAGAAGCCTCTTTTTAAGACTGGTCAAGGATGGAGAAAGAGCAGGATACTGTTTTTCCATTTGTAAGGGAGATTGTACCAATAGTTTAAAGGCATCGGGGGAAAGAAGAGGCCAATACTTCATAATAAGGCCGTAATAGAGTAAGTCAAATTGGTAATTATCCTCTATGGCAATGGAGATTTGGCGCCGCATGGGGGCCACTAGCGTGTTGAGGTCTGCTACAAAGATGTGATGAATGTTTTTTATGAGCTGGAAGCTGTCGAGCGCTTCCACATAGAGCTCATCTTTTTGACTTTCCATAGATGCGTCGATGTTCACCCCACCCACCTGTTGAAACTCCTTTTTGTAGTCGCGAATGTCTAACGCCACCCCGCTACCGCTTAACAATAGGCGATACGTTACCTGAAGCGCATTGTTTTGAAAAAAGAAAATGTGTTGTCTATAAGGGGGGATCCCTGTGGCAACGTAAATTTTTTCTCGAATGTCTTTAAAAGTGTCTTCAGGGAAAATACTATACTCGCTATACATCGTCTCAATTTCTGGCATCATCACGTTTGTCTCCTCGCCACGATCCTCCACAAAAAGTTTTTCAAACTCATCTAAATCATCGCTATCTCCACCCACCACGTATTGGGAAAGCTTTTTCTCCCAATCCTCGCCGTAAAAATTTTGTAAAATTTCTTTGTCCTTAGGGGTTCGCTGCAGGTCTTTGCGGCAGGCCTGTAACACGTTTGCAGGAACGGATCCCAAAAAAATAAACGTCTTCGTGTACTCATTTTCCACAGGATTATAAAGAGTAACTCGTAGAGGATTTGTTAAAAAGTCATTTTGGAAATCCATTATACCCGGTATAGAAAATAAAATTTAAAATAAAAAACGGATGATATCTATCATGGACCGTTCTGAGATTGTTGCACGGGAGAACCCGGTGATTACCCAACGAGTTACAAATCTCCTACAAACCAATGCTCCTCTACTATTCATGCCCATTGATATCCATGAAGTACGATATGGAGCCTACACACTTTTCATGTATGGTTCCCTCGAAAACGGTTACAAAGCAGAAGTAAGGATTGAAAACATCCCAGTTTTCTTTGACGTACAGATTGAGTTCAATGATACAAACCAGCTTTTTTTAAAGTCGCTACTGACGGCTGAAAATATTGTGTATGAACGGCTGGAGACGCTCACCCAGCGTCCTGTAATGGGGTACCGCGAGAAGGAAAAAGAGTTTGCACCATACATTCGAATATTTTTTAAAAGCCTGTATGAGCGACGAAAAGCCATTACTTACTTAAATAATATGGGCTACAACACGGCCGCGGACGACACAACCTGTTATTACCGAATGGTTTCCCGAGAATTAAAACTACCTCTTACAAGTTGGATACAGCTTCAGCACTATTCCTACGAGCCTCGCGGCTTGGTACACAGGTTTTCCGTAACCCCCGAGGATCTTGTTTCCTATCAGAATGATGGCCCCACAGACCACAGCATCGTTATGGCCTACGATATAGAGACCTATAGCCCTGTTAAGGGAACCGTTCCGGACCCAAATCAGGCAAACGACGTGGTGTTCATGATATGCATGCGCATTTTTTGGATTCACTCCACAGAGCCTCTAGCGAGCACGTGCATCACCATGGCACCCTGCAAAAAGTCCTCAGAGTGGACCACCATTCTATGCTCCTCTGAAAAAAATTTGTTGTTAAGCTTTGCTGAACAGTTTAGCCGCTGGGCTCCTGATATATGCACAGGGTTCAATGATTCTCGGTACGACTGGCCCTTTATCGTTGAAAAATCTATGCAGCACGGTATTCTAGAAGAAATCTTTAACAAAATGAGCCTTTTCTGGCACCAAAAGCTGGATACCATTCTAAAATGCTATTACGTAAAGGAAAAGAGAGTCAAAATCTCGGCCGAAAAATCGATCATTTCCTCCTTTTTGCATACCCCTGGATGCCTACCCATTGATGTCCGCAACATGTGTATGCAGCTTTACCCTAAAGCCGAAAAAACAAGCTTGAAAGCGTTTTTAGAAAATTGTGGGTTAGATTCGAAGGTAGACCTGCCGTACCATCTCATGTGGAAGTATTATGAAACACGAGACAGCGAAAAAATAGCCGACGTGGCCTATTACTGCATTATAGATGCCCAGCGCTGTCAGGACCTTCTGGTGCGCCACAATGTTATCCCCGATCGCAGAGAGGTAGGAATTCTGTCATACACCTCGCTGTATGACTGTATCTACTACGCGGGAGGACACAAGGTATGCAATATGCTCATTGCCTATGCCATCCATGATGAATACGGCCGTATTGCTTGCAGTACCATTGCCCGAGGTAAGCGGGAACACGGAAAATATCCCGGCGCCTTTGTGATAGACCCCGTTAAAGGGCTTGAACAGGATAAACCCACCACAGGTCTCGACTTTGCGTCGCTGTACCCCTCACTCATCATGGCCTACAACTTTTCGCCAGAAAAATTTGTAGCCTCTCGGGATGAGGCAAATAGCCTCATGGCCAAGGGTGAATCTCTTCACTACGTCTCCTTTCACTTTAACAATCGTCTCGTGGAAGGATGGTTTGTGCGGCATAATAACGTTCCTGATAAAATGGGATTGTACCCAAAAGTACTCATCGATCTACTTAACAAACGGACCGCCCTTAAACAAGAGCTTAAAAAACTAGGTGAGAAAAAAGAATGTATCCATGAATCCCATCCTGGGTTTAAGGAACTACAGTTTCGCCATGCCATGGTAGACGCGAAGCAAAAGGCGTTGAAAATTTTCATGAACACGTTTTACGGCGAGGCAGGTAACAATTTGTCGCCCTTCTTTCTGCTTCCTCTAGCCGGAGGAGTCACCAGTTCGGGTCAATATAATCTTAAACTTGTCTATAACTTTGTTATCAATAAAGGTTACGGCATCAAGTACGGTGACACCGACTCATTATACATTACATGCCCAGATAGTCTTTATACAGAGGTAACAGACGCATATTTAAACAGCCAAAAAACGATAAAACATTATGAGCAACTCTGCCACGAAAAAGTGCTTCTGTCTATGAAAGCCATGTCTACACTATGCGCCGAGGTGAATGAATACCTGCGACAAGATAATGGCACCAGTTACCTACGTATGGCCTACGAGGAAGTACTCTTTCCTGTGTGCTTTACAGGCAAGAAAAAGTATTATGGTATTGCTCATGTAAACACACCCAATTTTAATACAAAAGAATTATTCATCCGCGGAATAGATATCATTAAGCAGGGTCAAACAAAACTCACCAAAACGATAGGAACGCGAATTATGGAAGAATCCATGAAACTACGCCGCCCTGAGGACCATCGCCCCCCTCTTATTGAAATCGTTAAAACGGTTTTGAAGGATGCTGTGGTTAACATGAAGCAGTGGAATTTTGAAGACTTCATCCAAACAGATGCGTGGAGACCGGACAAAGACAACAAAGCAGTCCAAATCTTTATGTCTCGCATGCACGCTCGGCGTGAGCAACTAAAAAAACACGGCGCTGCAGCATCGCAATTTGCTGAGCCCGAGCCGGGAGAACGCTTCTCCTACGTTATCGTGGAAAAACAGGTACAGTTTGATATCCAGGGCCACCGCACAGATTCCTCCAGAAAGGGGGACAAGATGGAATACGTCTCTGAAGCAAAGGCTAAAAATCTTCCTATTGATATATTGTTTTATATCAACAACTATGTTCTAGGCTTGTGCGCGAGATTCATTAATGAAAATGAAGAATTTCAACCCCCTGACAACGTCAGCAATAAGGATGAATACGCTCAGCGCCGAGCTAAATCCTACCTACAAAAATTCGTGCAATCCATTCACCCTAAAGACAAGTCTGTCATTAAGCAAGGCAATGTTCATCGACAGTGCTACAAATACATTCACCAAGAAATTAAAAAAAAAATAGGCATCTTTGCCGACCTTTATAAGGAATTTTTTAACAACACCACAAACCCCATCGAAAGCTTTATTCAAAGCACTCAGTTTATGATACAATACTTTGATGGAGAACAAAAAGTAAACCATTCTATGAAAAAAATGGTTGAACAGCATGCTACGGCTAGTAATCGAGCTGGTAAGCCCGCTGGTAATCCAGCCGGCAATGCGCTGATGCGGGCTATATTTACGCAGCTGATTACGGAAGAAAAAAAAATTGTACAAGCCTTATACAATAAGGGGGATGCAATACACGATCTTCTCACCTATATCATTAACAATATAAATTACAAAATTGCCACGTTTCAGACGAAACAGATGTTGACGTTCGAGTTTTCCAGTACTCATGTAGAACTGCTATTAAAGCTGAATAAAACGTGGCTTATTTTGGCTGGAATTCATGTGGCAAAAAAACATCTGCAAGCTTTTTTGGATTCATATAACAATGAATCGCCGTCTAGAACATTCATTCAGCAGGCTATAGAGGAAGAATGTGGCAGTATTAAACCATCTTGCTACGACTTTATTTCCTAATACTTCTTAAGAAACTCTTTAAACAAGGACTTCGCATGGTCAAAGGTTCTAAACCCATGGCCCTTATGATTCGCCAAAAAAGCGGTTTCATCAAGATTTTCTAACCCTTTCACGGATGAAGAAATAAGGTGTTCGGCCTCGTTTGCCCATTTTCTATGATTTTTTTTCACCTCGGGTTCTAGATCTGTTTTCTCCATATACTCATTGTGGTCATATTTTTTTTTGGGAGGAGGCGTGGGTGGAGGAATGGGTGGAGGAAGTACACCCGACTTTCCCGCTTCAACCGTTTTATAAAAAAATAGAAGCATAATACAAAGAATAAGGACTATCGCAAATATGATAACCAGTGTCCCAGTCGAGGGCATTTTGTTATATAAGTAACGTTTTTTTTTATTTTTTATAATTCGAATGAAGAACCATGTTGAATAGTCTTCTACTCAAAGACATTTTGTTATACGGTAAATGAGAATTTATAAAATCCGAATATCACTATCATACTGTTTATCTGAGAAGGTCTCACTGGGTCCTGTGATGGAGAACCCATACTCTGTAATGCTGGGGTTTATAATGTGGTCAGGACTGACAAGCACATTTCTGAACTGCGAGAGTTCTAGGTTTAGACGCAGTCGTAATAGTCGCTGTATATTTGTAATAAATATTAGATTGCGTATGAGGCGAGTGTCAAAGCGATCCTTTCCAATTTGTACTAAGGTGGGCTTTTGTATTCCAACTCCCACTTGTTTAACGATGGACCAGGGTCCTTCTTCCCGATTTTGTTCCGTGATATAGGTCAGCACACTATTTTCTGTATATGAGGTATGATGTCGCATATTAATACCTGGTGCCATTCCAACTGGCGGTTGTGCAATTCGGGCTGTACCGGGACCCAACCATCGTGGAGTTTTATAAACATATCGTTCTAGCGTATTTAAAAATTCCTTAAGGTTATTTACGAGTAGCATGAAGGGTGCTATTAAAACAGGTGGATGGTTTATAACCATTGTCATAAACCATTGCATTGCTTCAATATCATTTTGTAATGCTTGACGGGGAGGCGGGGCAGGTAATCCACGTATGTTGAATAAAGCGGTTAATTGTGCACCGGCTGTTTGGGGCGTAATATTTTGTATTAAATTTATCATCGAATTGGCTTGCCCGGCATTTCCTATAAGATCGATTAAATTGGTTATTTGACCTCGATATTGTTGTACCCAGTTTTGAATGGCAGCGATGATCTCAGGGGTTGGATTGTTTTGAATTTCAGGTGTTTGTATTAGATTATTCACTTCTCTTCGTGTATCTTCAAGCTGAGTCCTAAATGCATTTAACTCGCCTATAATTTGGTTTCTATCAATAACATTTCTTAAACCTCGAACTGTTTCAGCCAATCGTATAGTACGCACAATTTCATGTAAGGCCTGGTTTATGTATATTGACATGGGATGGCCCCACCGCTCACGTCCACGTTGAATACCTGCGGCCAAACTAGGACCTGCCTCGTCATAATCAAATTGTGTAGGATAAAGGCTTCCAAATAGCACTTTATTGAAAATTTGGTCAGAAAGAAATTTAGGGCGGCCCATATTTAGCGCGTTGTCCCCTCTAAAGATGCGTGACATGTATCCGGCGTTGCCTTTGGATAGTAACTCATTCCCATATTGAGTAATAGAGACCGAGACATAGGGGTTTATAAGAAGTTTTAGCATAAATTCTCGAGTATTTATGGGGGGACGATTCGGAATGTTTAATACCTCTGCAACATCTGGTTGAGGAGCCGTGGTGTCCAGAGATCGTACTTTTTCAGCCGAAATGCCGTACATAAGACAAGCAATTTCTTCAAAACTATAGTCATAGTTGTAAATATTGGCAAGTGGTATAGATCGCATCAGCGCATTTACATTGATAGGTATAATATTCATATCAAACAAGTTAAATATGCGCTCGCGCTCTCTATTAGAGCCAAGAGTGCGTGTTTGACCTTTCGGCGACACTATTTTGTGAATATGATTGATTTGCTCCTCTTGGTAAGAGCTTTCCACGAAGGAAATTACGTCTTGCAATGTTTTACGAAGCGAATACACTGCATTCATCCCTATTCCCGCTGTTATAATGGGTTTATCGTCTCTGTTCTCGCTAATAAGATTAACTCCACCAAAAGTATTTTCATTGTACATCATCACTGTTTTAAAACTACGGATATTTATGATAAATCGGAGAGCCTGAATGGCGTGGGTATAAAAGTGTTCAAATCGCGTGGGAGTAATTTGTTCGCGAGCAACTACCGTTTCATTATAGTTTTTCATGATAAGCTGTACTCCGGGCATATCTGAGAGCTGTACCGGATCATTTCCCAGTAATTTTCTTGTGCCGTATAGTAGTTTAAACTCGGGGGAGCCGCTTTCAAGGTTCGGGTAAAGAAGAGGATCATATACCTCATTATTTTCTATTCTTAGGTCATGTAAATAATAGAGCGAAAGTGAAAATGGCATAAGAGGCTCCTTATTGTACCGGGACATATAGTTTTGAATGAAGTGTTCTTCTGTTTCAAGATAGATGGGATGATCGGTAAGCTCGTGCAGGACCTCCATGGCAGAATCTGCCAGAGTGTGAGAGCCTCTAATGATCCCGTCGATCACTGCGACCAGTCGCTTTCGCACAACATCGCTCGTATTATTTTGTGCGTCTCCTAGGGGCATAAGCGTAACATTGGGACGAAATACGCCGCCAATTCCCCGCAGGGCCGCCTGACCGACGGATAGTCCTGTCGCAGGAACATTGTTATTATTATAATAAATAACGGAATCATTATTGGCTCCCAAGAGTGCCGTCAGATTAGGGCGAGCTAGTTGGACATTTGTGTATTGTATAAATTGTTTTAGAAGCTCTCCCTGGCTAATAAGAATATTAAACATTTTGTTAAATAGTGGAAGATTGGCTCTATAATTTTCTTTAAGGTAAATGGGAATTTCTGTTAAAGTAGAAATAAGATGCTGACTCAGGCCCTGGCGATTGGTATCCTTAATAAGCCGCTGAAGTATAAGTCCCAAAGACAGAAGAAGCACCGACTGCTCTGTGGGGTCGCCCCTATGGCCAAAGGCGATGTTATCACGTGCTAGGTCGGGATGAGTGTACCCCAGCTCCATCACCGCTTGGCTAAAGTTCCCGTTGGCGAATGCATTGATAAGATTAAGATATATTTTTCCGCTGGGAGCGTCATAAAACCGGGCAATGTACGAGGCTACAAGCTGGTTAAACACCATCATCATACTACGATTATTTTGAATACCATAGTCTGATCCATATAGGCGATAACGTCGAAGGTTGTTTGCGGCATCATTGACGTTGGCATAGGTTCTGGGCGCCATATTGTCCCAGTAGCTAAGAGTATTTTCCTCCTGGGCATTGTTGGTACGAATAAGATTGGAGAGTCTAAAGTCTCCTAGTACCACTTGCTCTACACGGAGTCCAGAGTTATTCTCCAAAGCATCGTAAAATACGAGTCTACTAAATACTCTTCCATATTGTTCAAAGCGCTCAGAGGGTTGGGGATTGTTATTTATTTGAATATTAGCTGCATCTCTTCTTTGTACCCCACCTCGAAGTTGCAGTACATTATAGGGTTTTGTAAGCAAGATGTAGGTTTTATTAATGATTTGGTTAACTCCCTCCAGGCCTAATTCACCGCCAGGAAGCGGCCTTCCCCCGGCATCGGTGGGTGGTTTAATAAGCTTGTCAATCAAATGTTCTTCCAGCCAGTAAAATGAGCCAGGATTGGATCTATTTTCGTAGTATTGAATAATGTTTTTATCAATATGTGGGCGTAGAAGATTAAGAAAATACTTAGTGTCGGCCATCAAAGAATCAATTAAGGAAATAAGACCCGTAAAATCTAAGTGCACTTGAGCGGTGCTAGTTTCGGGAAAGCGAACTTGAACCATTTTGTTAAAACTAGAGGTCATTTCGAAGATATTGGTCAACAGGAGCTGCATGATTCGCTGATTGTCTACCAAATATCTTGCGGCTAATTCTTGCTCCGCACGAACTCCTCCACCAGCAGGAATACCCACATATGGTACAATCCAGGCAAAAAGAGTCTCTGTGACTAAATTTTGGTCTTGGGGTGCAGTCGCATTGGTAGTTGGATCAGGATACACCCTAGAAAGCCGCACATCATTTTCCTTAACAACCAATCCTGGATTTCTAATCTCAGAGATGGCCCCGTGTTTTCTTCCGAGCCAGTCAATAAGATTGGCTCGGTTCACGTTAGCGGCTTGCGTTTCTCGTAACCATTCAATGATGCTTTTTTGAATCGTATCTAGGTCTAAACCTTTAATGTTATTACGAAAGTTATTAAGAAGTACGTAAATAGCACTTAATAAGTTAAGACCTGTAATAACGGTTTCATGAAACAGAAATATTTTGTTAACATCTGTATCTGCCAGTGACTCAGAGCCTTGAATAAGTTTTGAAACAATTTGAATTTTGTCGGTATGCTCCTTTTTGAGTTCATTGATAGCCTGGCGAATAAGTTCCTGGTAGGAAACTTTGCCCAATTCTTGTTGTAGGCTGGGATCTTCAAACATCTCACTAAGCTGTTTCCTAAATTTTTGTACCAAGTCCCACTGGGAGTTGGGCTGCAGCATTCCTGTTTGGACATCCACAGAGTCTATATTGTATAGTGCCGGGCGCCACTTGGGGGTAGGTTGGGTTGAAGGACTAATAAACCTATCGGAGGGAAGTAATTGCGAGGATTGTGTATAGCCGTCCTCATCAGGAAGAATGGAGTAGTTGGTTTGATTCATCATTCCGAAGTCATTCATAGTTCGCGCTTCCTGAACAATGCGTTGAAATTTTTCCCATTCAGTGCGTGTGATAACGCCGAACCTGCGGTTTATTTCATTTACAAAATGGATAAGCGCTTTTTTGGTTGCTTCTTGTTCACCATACTCTAAGTTAAAGTGTTGGTAAATGACGTTTATTTCTTTGATAAGCTGACGAATTTCGGTTTCTGAGTAGTCACCAATGTTAATAAGCTCAATAGGACGCATAAAGATAATGCGAATAAGTCCTGAGAATATTCCTTCCAGCTCAGGAAGCATCGAGATCTGTACATTTTCATCTCTGAAGGAAAACAGTTTTTGATAAAACTCGGCGAGGCGAGGAAGGCGGAAGTAAAGTTCCGCTGCCTCGGGAATTACCTCAGGTTCTAGCTCATCGGCGCCTCCCAGTATCATACGTGTGGGTATAAGTTTGTACACAGGCTCAGGCCGTTCAAACATATCATAAATGCCTAATACAATGAAAATCTTAGCGGCCATACTTTTCAGCATGAAGGTGAAGAAGACGTCCTCGGTTTCCCAGCGGGTTGATAGGGCGTCGTTAACTCTCACAGTAGAGAGGTAGACCCGCTGAGCCGCTTCCTCGGGAGTTCGTGCAAGCGCTACCCTTTGCCCTCCAATTTCAGATTGATTTAGATTTTTAAGTCCCACGGAAAGCGCAGAATGTTGAATATATTCAAGCAAGGTTTTATAGATCTGCAAGGGCGACATGGGCACCATTTGCCGCAGCTCCTCTCCCCCAAGCATGTCTCCAATCCGGGCAAAAGCATTGATGATATTTTTAAGCGCCTGAAAGTTGGAAAGAGAACGCCCGATAAGGTCGCGAATGTTTTTAGCCTGGCTTGCTCTGACGGGACGGAGGGTACCAACGCTTCGGCCTTGTTGGATTTCAGCCGCAACTTTTTCGTAGTAGTGGCCCGCAGGAGCATTATCCGTAAAGACGTTGGAGTCGTTACCTGCGGAGGTGGGAAAACTTTCAAAGACTTGTGCAAGCGTGTCCCCTGTTGCCTCGGTGAACCATCGTCCTATAATGCGCACGCCATCCAACATCTGCTGGACTGTTTGAATAGAATCTATGTTGTTTACAAACGTTTTGGTAATGTTTTTAAGATAAAGGTCTAGCCCTTCCAGAGCTCGATAGAAGCGGCGTTTTACATCGTACTCCAGCTCGATGGCGCTTACGGTTGCCTTCCAGTCTACTTCCTGGGCACCTCCAGGATTTGGGCCCACGTGTCCTCTGGCAAGATCTACAGCCGGAGAATTAATGCGCGCATTTTTTTCCGTATCCAACTGCATGAGGCGTCCCGCAATAGCATCTCCGAGAATAGTGGCATAGTTTTCCTCGTAGGATTGAAACTCCTGTTTGTTATGCGTTAAATTGGAGTAAATCTGGGCCACATAATAGTAATACATAAAGGTGTTAATTGCCTGGTTGAGGTCAACCTGCGATCGCGCGGCCTTGCTGAGCCCAAGCTCTTCAACTGTTAGGGCAGCACCGCCTACCCTTGTACACTCGCAGTCCTCCTCGCCTCCATACTTTTTTTGCACAATATCGGTATAAAAATCAATAATCTGTAGCAAGCGAGAGCAGGAGTCATAAAGATTTTTAAAATTAGGGTCGGTTTTAGATATCTCCTCCAAAACATTTTTAACAAGCGTAAGCTGTGTTAAGAAGGTTTCGCGTTCTTCTCGTGCGGCCGCATTGGTGTAAAAGCCGATAAGACTTAGATCAAGTGCGATGGTGCCCATATCATTAATGCGCGAAAGAGCATCTCGAAGCCTCGTTATGTTCGGCGTCAAGGCAATTTCTTTAACAAGTTTGATGCCTATTTTTTTCACATTTTCCAAAAAGTCGTTATAGGCTTGTGTGCTTTTATTCAAAAATTCCATGAGGATGTGCTTTCTATCCAGTCTTTGCGCTTCAATCCTCCTATCTAGTGGCGTTTTCTCCTCATCGCCCCCCTTTTTGGCACAACTGTTCTCAAGGATTTTGTGGCGTTCATTAAAGGTCTGTCGCAACAGGTTCACGGCTTTTTCAAACTCAGCAATGTTTTCTGCGGAGACAAGACCACTAAACCTTTTGAGGTCAAGCTCCTTGTCAAACTCCGCCCAGTTTTTGCTTTGAAGGTACTGTTCAACCTTGAGTCCTACTTTCTGGAGAGCCTTATTAATTTTATTCGCAACAGACGCAGCAATACCTAGATTACAAAGTGTGTACGAAAGTACTTTTCCAAAATTTTTGGTTCCCAAGACACTATTTGTATCATTTAAAAGTTTAATAATATCCACCTCATCCGTCTGCAGTTTATCAAGTTCCTTTTGGGTGGGAGTTAAAATATTGTCAATAAAATTCGTTAAAATGTTGATTTGCAGGTTTTGTTCATTTAAAAGTCGACGATATACTGCTTCAATCATGGTGACTGCATTAATGACTTCCTCATTGGGGGCTGCTTTGGTTACCTCCGTCACCATGCGCTCGTGAAGTTGCTTAATGGCGTCGTTTAACAGCTTGATATTTTCAAGTGTATTTTCTATACTGCCGTGTACATCAAGATACTCTGCGCGCAGTCCATGAGTTAGGGAGTTAATGTACAGAACTATTTGTCGACATATACTGGCGGCCCCTTCGGTGGTATCTATAAGCTTATCCTGACCTAAATCAATAAATTCCTGGTTAATGGCGTCTGCAATCATTTTACAGACGGTCTCCTGTTTTTCCGCATTTTTTACAAAGGTGGAACCGGCTCGAGGATCGGGCAGTTGTTTTTTGATATCTTTAAGAATATCTTCGATGGGCTGCTTTGTGTCTACTTTGAACCCTATTTTGGCAATCGCCCTGATAATTCCTTCTATAATCCGCAGCTTTGCTTTACTCGATACGGAGTCTATGTGATAATCTTTAATGTGTTGTACAGGATTTTTGTCCCCCCCGCCATTAAAATATCCTCCCCCTGAAAAAGGACGAGTTTGTCTTTGTATATGATCCTGTAACTTCGCATATATATTTGCTTCTGATGAAGGCAGTGGTCTACTAGAGGTTGAAGATCCACGGTTACCCATTATAATAAAAAAAAATAAAGATTTAAAACTACAAATATTTTGCTGTTTATAAACCCAATCATATAAGACTAACTAAAACATTAAATGTAGGTGAGATAAAAGCTTATTTTTTTTTTAAAAGTTTAATAACCATGAGTCTTACCACCTCTTTTTCTTCTTCCTTTAGAGGGGTTCCATAAATGGTTTGAATAAAATTATGTGCTCTAATAACCTTGTTAAAATCAGGTGCCTTTCCATATTGTTCAATATGTTGCACAGTCTTTTGTGCAAGCATATACAGCTTGGAGTCTTTAGGTACCTCCGATGAGGGCTCTTGCTCAAACAACGTTTCAAAGGAGGATGTGCATTCATTGGTTTCATTATCATTTTTTTCATGAATGTTCTCCGAAGATGCTGAGGATTCCGTCTCCTCTTCAAACAGCACATGCAGAATCATATTCCATTCTTCTTGAGCCTGATGTTCAGTATACCCTTGCCCTGCATATATACGAGCAGATTTCACAATATCATACTTAACAGTACTAAGCAATGTTTTTATAGCGGTCGTAACAATTCTACCGCTATTGATAATCTCAACAGAAAACCAATTATACAGGCTACCCGCATGAAACACAACTTGTGAAGATGATCTTAAATCCGTTTTGAAGATGACCTCCATTTTCATGGATATATTTAAAATAAAATCCATTCAATTTTAAAATTATAAAATAATAAGAAGATGCCCTCTAATATGAAACAGTTTTGCAAGATTTCTGTATGGCTACAGCAGCACGATCCAGATTTATTAGAAATTATCAACAACTTATGTATGCTTGGCAATTTATCCGCGGCAAAGTACAAACACGGAGTTACCTTCATTTACCCCAAACAGGCAAAGATCCGCGATGAAATAAAAAAACATGCCTACTCCAATGACCCTTCACAAGCCATAAAGACCTTAGAATCACTCATCCTTCCATTTTACATTCCCACTCCAGCGGAGTTCACCGGGGAAATCGGCTCCTACACCGGAGTGAAATTAGAGGTTGAAAAAACGGAGGCGAATAAAGTTATTTTAAAAAATGGAGAAGCGGTCCTAGTACCGGCGGCCGATTTTAAGCCCTTTCCTGATCGCCGACTAGCGGTCTGGATCATGGAGTCAGGCTCTATGCCCCTGGAGGGTCCCCCCTATAAGCGGAAAAAGGAGGGTGGGGGGAATGACCCGCCGGTTCCTAAGCATATCTCGCCGTATACTCCGCGCACGCGTATTGCCATTGAGGTGGAAAAGGCCTTTGATGACTGTATGCGTCAAAACTGGTGTAGTGTCAATAATCCCTATCTTGCCAAGTCGGTCTCCTTGCTGTCTTTCTTGTCGCTCAACCATCCCACCGAGTTTATTAAGGTACTGCCGCTTATAGACTTTGACCCCTTGGTGACCTTTTATCTACTTCTTGAGCCCTATAAAACGCATGGGGATGACTTTTTAATTCCGGAAACCATTTTATTCGGCCCTACCGGATGGAATGGTACAGATCTGTATCAAAGTGCCATGCTGGAGTTTAAAAAGTTTTTTACCCAGATTACTCGCCAAACCTTTATGGACATAGCCGATTCGGCTACTAAGGAGGTAGATGTTCCCATATGTTACTCGGATCCCGAAACCGTACATTCCTATGCCAATCACGTGCGTACTGAAATTTTGCATCACAATGCCGTCAATAAGGTTACAACACCTAACCTCGTCGTGCAGGCCTATAATGAGCTCGAGCAAACCAATACCATACGACATTACGGCCCTATTTTCCCGGAAAGTACCATCAACGCACTGCGTTTTTGGAAAAAGCTGTGGCAGGATGAACAGCGATTTGTTATCCACGGCCTGCACCGCACGTTGATGGATCAACCCACCTATGAAACCTCTGAGTTTGCAGAGATCGTTAGAAATTTACGGTTTTCGCGTCCCGGCAATAACTATATAAACGAGCTTAATATTACAAGTCCCGCTATGTACGGCGACAAGCATACCACCGGAGATATTGCGCCCAATGATAGATTTGCCATGTTGGTGGCCTTTATCAACAGTACTGACTTTTTATACACCGCGATTCCCGAGGAAAAGGTAGGGGGGAATGAAACCCAAACCAGTAGCCTTACAGACCTAGTTCCAACACGGCTACACTCTTTTTTAAATCATAATCTAAGCAAACTTAAAATCTTAAACCGCGCGCAGCAAACGGTTAGAAATATTCTTTCAAATGATTGTCTTAATCAACTGAAACATTATGTTAAACACACGGGAAAAAATGAAATACTAAAGTTACTTCAAGAATAAGTATGTTGATACCTGTGGTGTGTTTTACCTGTGGGTTTCCTATTGGAACCTACGCGGCAATTTTTGACAAGGCTCGTACCGAGTATATTAAAACCAAAATGGGCGGAACATTGCCGCAAAATATCCCATTAGATGCTTCTCTCCAGATTGAGTTAAAAGACCTCATTACAGCTCTGGGAATCCCAATGCGGGTGTGTTGTCGCACTCATTTAATTACTACGTTGGATTATCGTAAATATTATTAATATCTAAAATTGAAAAAATATTTTTAATGTTACTAGTAAAAATGACTACACACATCTTTCACGCAGATGATCTCCTACAAGCATTGCAACAAGCAAAAGCAGAAAAAAATTTTTCATCTGTATTTTCTTTAGATTGGGATAAATTACGCACAGCGAAGCGTAATACAACGGTTAAATATGTTACGGTCAATGTCATAGTAAAAGGCAAAAAAGCTCCGCTAATGTTTAACTTTCAAAATGAAAAACATGTAGGAACCATTCCTCCCAGTACCGATGAAGAGGTTATACGGATGAATGCTGAAAATCCAAAGTTTTTGGTGAAAAAACGTGACAGGGATCCCTGTTTGCAGTTCAACAAATACAAAATCTCGCCGCCATTGGAAGATGATGGTCTCACTGTTAAAAAGAATGAGCAGGGTGAAGAAATATACCCCGGCGACGAAGAAAAATCTAAGTTGTTTCAAATTATTGAACTGTTAGAAGAAGCCTTTGAAGACGCTGTGCAAAAAGGTCCTGAAGCCATGAAAACGAAACATGTTATAAAATTAATTCAAAGAAAAATTTCTAATAGCGCGGTTAAAAACGCAGACAAACCTTTGCCGAATCCTATCGCACGCATTCGTATTAAAATCAATCCCGCTACAAGTATACTAACACCAATATTGCTTGATAAAAATAAGCCCATTACTTTACAGAATGGTAAAACAAGCTTTGAAGAGTTAAAAGATGAAGACGGCGTTAAGGCCAATCCGGATAATATTCATAAGCTTATAGAATCGCATTCTATACATGATGGCATCATTAATGCTAGATCTATTTGCATCAGCAATATGGGCATTTCATTTCCGCTTTGCTTGGAAATGGGAGTTGTAAAAGTTTTTGAAAAAAATAATGGGATTGATGTGAACTCCATTTATGGCTCAGACGATATTTCAACTCTTGTTAATCAGATTGCTATTGCTTAAACAATTTGCTCAAAACAAGCTTATAAACGTTTCTTAGGTATGCGATACGTAAATCCTAATTCTTTAATAAGTTCTTTTTCAGTAGTGATTTTTAGAGGTACTAAAGTTTGATTTTTAAATAATCCATACTGATTTAGCTTATAATTCTTTTTTTTTAACGCAGCTCGAATTCTTATTAAATAAGAAACGGGACCCGTAAAATGAAGTACTGCGTATGGCTTTTCCTCGGCTAAGGCCGTAAAAAGATCAAGTTGATATGTGTTTTTTTTCCATTCAATAAAAAGTACACACTTTCGTTCTCCGCAGACTTTTACAGAAAAAGAAAGATCCTTTATGCGAATGTTGGGCAGGACGTGTTTTAAAAGTTTTTTTTCTGGAACAATAATAAGAAGATCCACGTCATTAAGCATTTTCTCTTCGCGTCTTAAGCTACCAACAGCAACGATGTTTTTTGATAAAATTTTTATAAGTTGTCCATTATATTCAAACGCAAGTCGGGAGCGTAAGTCATTTACAATTTTTTTTCCTTGAATAAGCGTTAACATTTTATATTTAATATTAAAATCTTTTCATTTTATATATTATATACGCAAAATGGCACTTGATGGTTCAAGTGGTGGAGGCTCTAATGTAGAAACATTACTTATAGTAGCAATCATTGTGGTTATTATGGCAATCATGCTTTACTATTTTTGGTGGATGCCCCGCCAGCAAAAAAAATGTAGCAAGGCTGAAGAATGCACATGTAATAACGGAAGCTGTTCCCTAAAAACAAGTTAAAACATGCAATTATATGCATGCATATAAACGCATGCATATAAACGCATACATATAAAATGCGTAAATACTATATAAAAAACTATAACATATCAATCAAGGAATCAACACTTTTATAATTTTCCGTAATATATTTTTCATCCATAATGATGTCAGAGTACATGGTCCCTATGCGAGGAACAGAGCCCATAAGGGTAGGCGCGGCAATACCGTAAATGGGATTCACGGCGGAGTCAACCGCAGCATCTGTCAAGACCTGGACTGGAGACGACAAGGCCATTCGCAACAACACGTTGGAAGGCTCTCTTGCATTAAGCCCTGCCTTTTCTAGAGAGGTAACCTGTCCCGTTCTTGTCATGAGATCTGCGTACATGAGTAAATGACGATGGTTGGGACCCTTGTCCCCCATAACCGTTCTAATTTCACTAATAATTTTTTGCCGTGCCGCTTCTATGCCGTAAAGCTCCATGGTGTCTCCTATAGAGGACGATACGATGGTGTATGGGTCGATGTTATCATCAAGCATTGCGCCAAAAATATTAGTCCCGTTTGTTTTGATGGCGTAGATATTGTCTAGTCTTACCAGTTTCCCCTGGGCATCCACACGGTGGCGCATAAGCTTAACAACATTCGCATTTTTGATGCCTGGTATTCCTCTAATCGTGCTATTTAATAGTTTATCCACCACATTTACGGCAATTTTTTCATCCGTAGCCATTCGGGTATTGGTACTGCGTCTAAAGGCGCTTTCCCGTAGGTATATGCGAATAATGATGGGAATCCCTGAGGCCGTGTTTTCCACAGAATGCATGATGTAGGTGTTGGGGTGTTTAGCTCTTAGACTATTAATAATACTTTCTAGACTAATGCTTTTTAATATCATGGTTGTTTTGTTTAATTCCAAGCGGATACACCAGTTTGCAATATCCTCTGGGGGCTGTAGTAGAGGATGGTTTTCCAGAAAATCCGTCATCCATTCCACATCACTTGCAAAATCGGGGTACATCACATTTTTTTTTGTGCTTGAATACGTTTCGTACAATAGGTGCCACTGCAATATCAACCGTTCGAACGTTATAAGCTCTATGCTGTTAGCAATTTCTTGCGCATATGTTTTATTTGTTTCCACTTCCGGGTTCTTTAGACGTAAAAGCATTTCAGAGGATTGTTCAGCCTCTACGGGCTTCGCGCTAAAGATCTCCTGGGGCCGCACAATTCCCGACTTGTTGGTTCCCCCGGCCACGGACCGGTGGTGGGAGTCCAGCATATATTGTGTCAAGGGCTCTGATACGGACTGCGCCGCCAGGATTCCCACTGCCTCACCGTAGTTAATAAGACTTTGAGTATATTGTAGCCTTATGAGGTCCAGGATGGCACTCATCTGCTCGCAGGTAATGTTTAATGTTTTAACGGTTGCCAGTTCGATGCGAATAAGCATGCGCATCAGAGAGGCAGCCCGTTTAAGATAAACGGGTATGGGCGTTTGTAGTCGTTCCTGAATGTTGTTAATAAACACGTATGGAAGATTTTTGCAAAACGTTTTGACCATCGCGTATTTTTGTAGAATACTTTTTTCGTCGAAGGGAAGCACGCCACTGGTGGAGCTCAGTAGAATGTTTTTTACGATGCTGGCCACGTTTACCGGCACCTGTCTAACATCTGTAAGCAGCTGACTGAAATTAAAATTTTCGACGTTTAGGAAGATCTGTCGATATTTATCTCTATCCTTTTTAAGGCGTGAAAATTCTTCTTCAAACAAGGGCGATTGTATCCCGGTGTACTTGAATTTGTCTTCAAGTTCCTGGTCCGACAGCATGATGGTTTCAAACCGTACGGTTTCAAGCTGGCGCGCATCAAGGCCGTCCTCTCCGTACAACTGCTGCACAAGACGCGTATCGATGGAAACCCGTCGGTAATAATCCACAATACAGGATTGAAGGCCAAAGATGGCTTTACGGTTGGCATAGCCTGTGGATGATGTCGATAATGCTTTGTTGATCAAGTCGAATCTTCCATTCATTTCCCCAAAGATAAATTCAGGGGAGGTAAGGCCCGCAATATAGCTGTTGCAGATGAACCCGTAGGCCTGCGCCTCCAGGGCAAACCTGGGGTAGTACACCAGGGTCCTACCGAAGGAAAACTGGGGTTGAATGCGTTGTGTATTAATTTCAATTTGGCCGATGCCCGCCATGATGTGAATCATATTGGGGTTTGAGCCCTTGGCGCCAGTGGCCACCATCTGAAAAAGCCCATTGGTTTCCGGATTAATGGAATTCATAATCGGCTTTAAAATTCTATCGGGAAATTTAAGCGCATTCAGCTGCAATTTTTCGTAGAAGTCATGCGTTGTCAGGCCTATAGGCGGCATGATGTCTCCATGAAGCAGCCGGTTGTTTATTTCCTCCGACTCAAGCAGCAGTTCATTGATAATTTCTTGGACCTCCTGATGTGCCTCCGGGGTTAGGAGCATGTCGGCCGTGGACACTGTGAATCCGGCGTTGCGCACGTAGTTTAGGGCGAGCTGCTGGGTCGCAAATATCATTTTCAAGGCCTGCTGCGGCCCATACCTACGCGAAATAAGGTGATAGATTCCACCGGAGGAACCCGCTCCGACGGCCTTTTTGTCAAGGACGCCTTCAATGAGTTCGCCGTTGCGTATTTGTGTAGAGATGTCCTGCTTGTTATAATGCATGTAGGGTGCATACACTTCTGAGTACCATGTGGGGGCTCGTTGATAATTGATGGGGGTCTGCCTCAGTAGCATAGATACAACCGATTTGCCATCCAGCAGGTCAGTTGGGGAGTAGTTGGCAAAACAAGGTGGGTCGGTTTGGGTTGTTTGAAACAACCCCATGGCGTGCAGCTTGTTCATCACATTTTTCCCCATGGGGGTGTTCGTGCGTGTAAGCAAAAAGCTTCCCACCGTGGAGTCCTGCACCTGCCCATTAACGGGACCCGAGCTCTTTGTGGAAATGAACCAGTTTCGCACAGAACAAAGTAGTTCGGCCTCAACGCGGCTCATGACGCTCCAGGGAACCCAGAGATTCATCTGATCCCCGTCAAAGTCCGCATTATACCAGGCACATGCGCTGACATTCATTTGAAACGTAGAAATTTTTGGGTTTTCAAAAACGACAATCCGGTGAACCCCTATGCTGCTTCGTTCGAGAGAAGGCTGGCGATTAAAAAACGCGACGTCGCCAGTGACGACGTCACGGTAAAGGATGTCTCCTACCTCCAGCCTAAAGTCTTGTTTGAGACCCTCAATGTCGTGAACGGATTGTGTTATTTGCTTATACACTCTTGAACAACCAGGGTACTGGCGCTTTCCATTTAAAAAATAGGGCATTAATCTATTAATATTATAATGTTGCACTGTTTCCGCAACTTGCAGCGTTCGTGCAAAGGAAATGGGATAGCCAACCTCGTCCAGGTGAAGGTCTGAGTTCCCGCAGATGGTGGACCGGCTGATCGACCATACCTGGCTGCCCAGTAGGGATTTACGAATTCTTCCCTCCTTGCGAGGAAGTCTTCGCATGATGGAGGGAGCAGGGCGTGCCCCCATGACGATCCCACGCTTTCCCGTGCCTCCCTGGGTTGCGGTGGTGGAAACGGAATCCAACAAAAAGTTATAGTAAAGTTGCTGTATGGTTTGCAAATTGCGGTCAATATTTAAAGGTATTTTTTGGCCGCGCACGATTTGTAGGTCCTTCGGGATCAGCAGATTCTTTCGAACCAGATACTGAATCACGTTGTTAATGTCGTGAAAGCTTTGGGGGCCTGACCCGATTCCCAATCTGATGCCAGGTCGTATGCTGATGGGGGGGATCTGAATGGCCTTAAGCACAAGTTTTTCGGGATGGGAGTTTTTACTTCGCCCCAGTTTTACAACGGTGTCGTAGGTTACGCGCGAAAAAATCTCTCTGATGATCTGCGGGTACAGTTTGTCAATCTTGCCCTGCTGATCCGCCCAAAAGGTAAAATAATCTTCCGAGTCCTTAACAATTTTGGGGTGTACTGCCTTACAGACGTAGCACTGCTTTCCTTCGGTTTGGCTTGAAGCCGCTTCAATAAGACGCTTAGGCCTAATAAGATGCTCGTACCTCTTTAGGTCAACAATGGGAGCCCCGCAGTTGAGGCATATAACCCTTAACCATCGTCGTATTTCGGCGATGAAGAGCGGCTGAAGCACCGGAGCATGCATCTGCAGTATCCCAGGGTGTCCCATACATTGCTTGCGCTGGTGTGAGCAAGTGATGCATTTATAATGGTGATCGGTGGTTCCCATTCGCGCATCATAGATACCCCCCTCGGCGGGAAGGGTGCCCTCAAATAAATTAGAAATGGTAACCTCCATAACGCCTTGCCTCTTATGGTCATTGTCACCGGCAATATTGAACTGAACGGCGGCTATTTCGGCATATCCAGCCTCCATATTTTTGCTAAATACATAATAAAACTTCAAATGTTAAAAAAAAAATAACATCGGTTGGCATATTTTTTGTTAAAATCAAATGTTAAATGATTTCTAAAACATTTATCGGTTCACGAAAACCTACCGCACGGGCCTGAAGAGGAATGCCAGTTTTGGGGGAAAGCTCGGCATATTCCACGGTAAGCTCTTTTCCATAAAGATGTTTTTTAAATAAGGCGGGCGTGAGTTTTTGAAAAAGAGCATAACGATCCGCGTACGTCAAATGCTTAGGAGTCACTACAAACCGCTTTTTGTTTGGCAGTTCACAAACCCATAAAATGGCGCCTAAGTCCTTTCCCTTTTTTCCCTGAGTATAGTCCACCAAAATAAATTCAGCGTCTAGCAGCGGTTTCAGCTTGGCAAGATGCGCTGAGTGGTAGTTGTTGTATCCCGGCTCATAGGGCCCATTGGCATTGCGTACGATGGCTCCCTCGTAGCCCTCCTTAATAAACTGCGCCTTAAGCCTAAGGGCCTCATCCACATTCTTCACGCTAAAATTTTCAACTTGGTGGATAAAGGTAAGATCTTCCTTCTGTTTAAAAATATTTGTTAACAGCTGCTGTCTCTTGTTGGAAGGCATTTGAAGCTGATCACTCCAAAAGCAGTCAAACACGTAAAAGTGCAGCTCGGAGGAATCTGTCTTCGCATTCGCCTGCCCCGCGATCCATTGCAGAGGTTTGCGGTGTAAATAAAGCTCGCCATCCAAATATACTCTCACGTCTATAAATAAATAAAGCTGTTTGAGCTCTTTTTTAATATTGTCAAGACCTAAAAATTCCTTTTCCGTGCGCGAATACAAGAGAATGCAGCCATCGCCCTGCTGGCAGGCCACAGCTCGAACGCCATTGCGCTTGCGCTGCACGATGGGATCTGTTTCTTCTTCAAAAAATGTCTTAGGAATTATATTAAAATATTTTACCAGCATAGGGGGGATCATTCCTCTATTTGTGTGGGCTCCCCGCTTTTGTCTGGCATGGCGATTATATTTACTAAGGGCATCCTTGAATGCCTGGTGGACTACCGTTGTGGCATTTTTTTTACCCAAGTTTTTTCCCTCGGTAACACGTGTCATTTTTGATATCCGCACCGCCCCTTCTTCCACAAAAAATTTTGTGAAAATTTCAGCAACGGCGTCTTTTACATCTGTGGAAAACATCTCATCTGTGATGGGAATGATTGTGTTGTGCTGCACCACTTGCACACAAATAATCCATGAGGCCTTTTTTCCGCTTTTCGTTTCAGACTCAATCGGAGGAAAACAAAAAATGTTGTTTGAATATTGCCCAGGAAATTGATTTAGCATGGTTTTAACAATAAAATAAGCCTATCAATTTTTTTATAATTTGAATAGTTATTCCAAATTCAATATGGCTTCTTTAGATAATTTAGTGGCACGATATCAGAGGTGCTTTAATGACCAGTCTCTTAAAAATAGTACTATTGAACTTGAAATACGTTTTCAACAGATAAATTTTTTATTATTCAAAACCGTATATGAGGCACTTGTGGCACAAGAGATCCCTAGCACCATCTCCCACAGCATCCGCTGCATCAAAAAAGTTCACCATGAAAACCACTGCCGGGAAAAAATTTTGCCGTCGGAAAATCTTTACTTCAAAAAACAGCCTCTCATGTTTTTTAAGTTTTCAGAGCCTGCATCTCTGGGCTGTAAGGTCTCGCTGGCCATCGAGCAGCCCATTCGTAAATTTATCTTGGACTCCTCCGTTCTCGTTCGGCTCAAAAATCGTACGACCTTTCGGGTATCTGAACTTTGGAAAATAGAGCTTACCATTGTAAAGCAGCTGATGGGAAGCGAGGTCTCTGCAAAACTTGCCGCTTTCAAAACGCTTCTGTTTGACACCCCAGAGCAACAAACGACAAAAAATATGATGACGTTAATAAACCCAGATGGCGAATATCTTTACGAAATAGAAATAGAGTATACAGGAAAGCCCGAATCCCTAACGGCGGCAGATGTTATAAAAATTAAAAACACGGTGTTGACACTTATTTCTCCGAACCATTTAATGCTAACAGCCTACCACCAGGCCATTGAATTCATTGCCTCCCATATACTGTCCTCAGAAATCCTTCTTGCTCGTATTAAGAGCGGGAAGTGGGGGCTTAAACGCCTCCTCCCCCGGGTGAAATCCATGACCAAAGCGGATTACATGAAATTTTATCCGCCCGTTGGCTACTATGTAACGGACAAAGCAGATGGAATTAGAGGCATCGCCGTCATTCAGGACACGCAAATTTATGTGGTTGCAGACCAGTTATACAGCCTAGGTACCACCGGCATTGAACCCCTTAAACCAACCATTTTGGACGGTGAATTTATGCCTGAAAAAAAAGAATTTTATGGGTTTGACGTCATCATGTATGAGGGCAATCTATTGACGCAACAGGGGTTTGAAACAAGAATTGAGTCTTTAAGCAAGGGCATTAAAGTCTTACAAGCGTTTAACATAAAAGCAGAAATGAAGCCCTTTATTTCGCTAACAAGTGCAGATCCCAACGTGCTCCTCAAAAACTTTGAAAGCATTTTTAAGAAAAAAACTCGCCCATATTCTATTGATGGCATCATTTTAGTAGAACCTGGCAATTCTTATCTAAATACAAACACCTTTAAGTGGAAGCCCACCTGGGATAACACATTAGACTTTTTGGTGCGAAAATGTCCGGAGAGTTTAAACGTACCAGAGTACGCGCCCAAAAAAGGGTTTTCCCTGCATCTACTATTTGTAGGCATCTCCGGAGAGCTTTTTAAAAAATTAGCGCTAAATTGGTGTCCAGGATATACGAAACTATTCCCCGTTACACAGCGCAACCAAAACTACTTTCCAGTACAGTTCCAGCCATCGGATTTTCCATTGGCATTTCTTTATTACCACCCAGATACCTCGTCATTTTCTAATATAGATGGAAAGGTCCTTGAAATGCGTTGTCTTAAGAGAGAAATCAATCACGTCAGCTGGGAAATTGTAAAAATCCGGGAGGATAGGCAGCAGGATCTTAAAACCGGCGGGTATTTTGGCAATGATTTCAAAACAGCCGAACTCACATGGCTTAACTATATGGATCCCTTTTCCTTTGAGGAGCTGGCAAAGGGCCCTTCTGGAATGTACTTCGCCGGTGCCAAAACCGGCATATACCGCGCTCAAACAGCACTTATTTCCTTTATTAAACAAGAAATCATCCAAAAAATAAGTCACCAATCCTGGGTTATCGATCTTGGAATAGGAAAAGGGCAGGACCTAGGACGTTACCTGGACGCAGGGATAAGGCATCTTGTTGGGATCGATAAGGATCAAACCGCGCTTGCGGAGCTTGTTTATCGAAAATTTTCGCATGCTACGACCCGACAGCACAAGCACGCTACCAACATTTACGTGTTGCATCAAGACCTCGCAGAGCCTGCGAAAGAAATCAGCGAAAAGGTACACCAAATTTACGGGTTTCCCAAGGAGGGAGCTTCTTCCATTGTTAGCAACCTGTTTATTCACTATCTTATGAAAAACACGCAGCAGGTGGAAAACCTGGCCGTTCTGTGCCATAAGCTTCTTCAGCCGGGGGGAATGGTGTGGTTTACCACCATGTTGGGAGAACAGGTCTTAGAATTACTTCATGAAAATAGAATAGAGCTCAATGAAGTATGGGAGGCTCGTGAAAACGAAGTGGTCAAATTTGCTATTAAACGTCTCTTTAAAGAGGATATATTACAGGAAACTGGGCAAGAAATTGGAGTCCTGTTACCCTTCAGCAATGGCGACTTCTACAATGAATATCTTGTGAACACAGCGTTTTTAATTAAAATATTTAAACATCACGGCTTTTCCCTAGTTCAAAAGCAGTCCTTTAAGGACTGGATTCCAGAATTTCAAAACTTTAGTAAAAGTTTGTATAAAATTCTTACAGAAGCCGATAAAACTTGGACAAGCCTTTTTGGGTTTATTTGTCTGCGCAAAAATTAAATATTTTTTCATAAGAAGTACTACCCAGGTTTTAAAGAAATAGCTAAAAATATCATATGGATACTGCCATGCAGCTTAAAACGTCTATTGGTTTAATTACATGTCGTATGAACACCCAAAATAACCAAATAGAAACTATTCTGGTTCAAAAACGTTACAGCCTTGCTTTTTCAGAATTTATTCATTGTCATTACTCTATAAATGCTAATCAAGGTCATCTGATTAAAATGTTTAATAACATGACAATTAATGAACGACTGCTTGTCAAAACACTGGATTTTGACCGCATGTGGTATCATATTTGGATTGAAACTCCAGTCTACGAACTATACCACAAAAAATACCAAAAATTTAGGAAAAATTGGCTTCTCCCGGATAATGGGAAAAAGCTTATTTCATTAATCAACCAAGCAAAGGGCTCAGGAACACTTCTATGGGAAATCCCTAAGGGTAAGCCGAAGGAAGACGAGTCGGACCTTACCTGTGCCATACGGGAGTTTGAAGAAGAAACCGGGATTACCCGCGAATATTACCAGATTCTCCCAGAGTTTAAAAAATCTATGTCATACTTTGACGGTAAAACAGAATATAAGCATATCTACTTCCTTGCAATGTTATGTAAGTCGTTGGAGGAACCCAATATGAATCTTTCTTTACAATACGAAAACCGAATTGCCGAAATTTCTAAAATTTCTTGGCAAAATATGGAGGCTGTACGTTTTATTAGCAAACGCCAGTCATTAAACCTGGAGCCTATCATCGGGCCTGCATTTAATTTTATTAAAAACTATTTACGATACAAGCACTAGGATGCCGCATTAAAATGCCACATAAGGTAATACACTAGGAATGTCGCACACGCACAAGAATACAACGTCGCCGGAGATTTATTATCTAGTACACGTTTTATGTATGTACAATCCGCCTTCATTTAATATATTGAGCGGATGTACTATGTATTTATTTTAACAAAAAACATTATTTTTTTTTAATCTTCATCATCTGTTTTTATAAACTCAGTAATATCAAAAGTAGCTTGTGGGGTTTCAGAGGGTTCACCTTGGTTATCCTCCGTGAGGATAACATGTTCTTCAGGTTCGTCGTCACTGGAGAACCCATCATTTAATTCCTCTTCACTCAACATCTGTAAAAAATCTTCCAAGCTTTCGCTATCGTTAAAATCCTCATCATCCATAAGAATAATGGTACCTTCCTCATCGTTTCCTCCTTGTTTCGTGTCTAAATAGGCCTGCATGGCATTTGCAAAAGTATCAAAATAGGCTGAGTCAGATTGCTGTTCCAAAATATGGCCTTGCGTATTAAATGTGGTTGCATCGTTGTTAAATGCTTGCAAATACAGTAAGGGATTTATATCCATTATTATTAAGCAAAAAAAATTTAAATTATTTTTCGACCGATGTTAGGTAAAATTAAACAATTGCTATAGGTGTTAAGCAATGTTTATTGATTTTAAGTACTCAACAACCATGATGTAAATACTATACAGCACTTTTGGATTTTTAATCAAATCCAGATTAATACTAACTTCTTTTGTGATACAGTTCGTAATAATAGTATCCTGCTCATCGTTTTGTAAGATTTCTTTTAATATATTTTTTTTTACCGGGATACTAAGCAATTGATTATTTTCTTTTAAAAACTCCTTTTGATATTCAATCGTCTTATTCATTGAATATTTGTATATAACTATAATTACAAATGTTCAATGAATTGTTATTCATGTCGGGAGATGGCTATTTAAAAATCATGTCCTATTTTTCTTTGCTCAATAAGCATCCAAATATTTTCATGGCGTTTTATTAATTGTTCATTATTGAACGTATCACAAAGATCATTTATAAATTGCAGATAGTTTATTATTTCTTTCAAGAGAGTAACAAACATTACTTCAGCAGAACATATAATAGGTAATTCAGTGGCGTTAAAAGAATTTTGATCTTGTTGATACGCCAATGGCGAGGACTTAAGGAGATTTGGGGGTCTTGCCCAAAACCCTAGGCTGCTGTTCTTGTTTTTTAGGGCGTCATAAAGAAATGAAAGCACATTGCAAGGCTTAAGCCGCGACATCTCCTTCCCCTTGGGCCCTTTCCATATTTTTAGATCTAAGATCTCATCCGAGCTTATAGAGTAGGTATAGTAAAGTTTTTCAAAAAAGCATATCTGCTTGAAGTCTTTTTTAGAACGACTTTCAAGAAGCATTTCTATAATGTTAACAAGTTTTGTTAGGTTTAAGGCCTGTTCCTGTGTAAGCTCCTCTTGCACGTGATAGACTGAAAAAGTGTGCTTAGGAATGAAAATACTCCCCGTGGCACTGGCCTGTTGTCTGCCAGGTATATAGTACACGCTGCTGTTAGCAAGCTGTACCGGCACAATTTGCCCCACTTCTGCAACATTATTTTGCGATTCGGACGAGGGTATGACAATAGTTACGGGTTCAGTCAATAGGCTTTCGCCGAGAATAATATTACTGTCATTTTTAATAATTTTAACGGCCGCTATTAAATCAAAGGCATTTAAGTAAGAAACAACAGCAGAAAATCTTACATGCATATATCCTCTTCCGCTATTATTCGTACGCATAATAAAACAAGGGGAGCGTTGTATAACGCCAGTAATATTAAGAATAAAACTGTTTTTGAAACACTTACCCACATAAATGTTTTCAAGCTCCTTCAAAAGATGAGCCTCCACATTTGTACAAAAATTGGTAGGATCATCAATATTCAACGTTGTCTCAAAAATTTTTTGGTCGATCATATCTATAATATATTCTGTCTATTTCAATTTAAATAATATACGAATAAATAACGAGATTATTTTATTAAATAAGCAATGGTGTATACACTTTGTATTTACTTTGAGATATACTTTGTGTATCACAACGTGCCCTAAGATGTGTGCACAAGTGACGGCATTTTGTCGTTAAAAAGGTAAAACCAGCGGATTCCATCCTGCATTCCATTTGGTTGATTACGAGCCTCCATTTCTTTTTGCAAAAGGTTATTGCGAATGAGTAAGCAGAGCTTGATGGCACTAATCTTTGTAAGGTTTAAACTTATGCCCAATTGGTCAGCAATTTTTTGTTGCTCCTCCCGTCCGCGTGTTTCGCATACGGCTCCCCGGTTTAGCATGCGAATATCAGTAATCTCATTCTTTTTTAAAACCTGGATAGGTGGGCGGATTTTAAATTTAAGGGCCTTTCCCTTGCTTTCCATATAGCCTATGACGATGTCGTTTTCTTTTCGTTTAACATTAATATTAAGCATATAAAGCGGAATTTCATGCCAGGTTTTATCTTCTCGCGAGGTAATAAGTCGCACGGAGTCCTCCGTGGCATAGCCCACTAGAGTGTTGTCATCCCCAGGCACGTGGCTTATAATTTTAAAAATGTCCGGAAATGGCTGAATATCTTTTTTTGAAAAAGCGATGAAAAACTTTTTATAAACCTCGACAAGGGCCCCCATACCTGCAAGATTATCTATAATAAGTGCTTCTAGCATCGTATAGTGAAATGAAGCGGGATAATGGATGAGTACCTGCTCCATTGGCTCATCCTGAAAATCCTTCTGAAACTTTTCATACAATACTTGAAAGGGTTCTTTGGTCTGCGAGTGTTCGAGGTATTTGGTAATACGGATGCTGTGCATCGCGGGAAGCTGAAAATCCCGAATATATGTTTCAATATCTAATACCGGTTCCTTTTTATGGTTAAGCACCGCAGCGACGTACAAATGCTCAGGCTTTGCCGGCACATGCATAATGGTGCAAAGACGATTCTGTATCCATAATTCCTTGCACTGGTTTTTTGAGTAGCATAGAGAAATGAGCGCCAGCGCGAAGTTGTCCTCTGAGAAGAGTTTATTATCGATGGTAATTCCCTGTATGAGCTTGGGAGTGGAAACAGCCTTCCATAGCTCGGAGTACGTCCACACGGGGCGTGCCATAAACAAAGATATAATAATATTAGAAATTGTTTTTACCTCTTGCTCCCCGTATCCATAGGCCTCAAAGGTATTGAGGACGGTGGCTCCGACGTTTGCCGGCGTGATGGATGGACTAAGGGGCAGACTTTCCAACATAGGCTTATCAATCTTAATCTGGTTGGTGAACCCATCAATGGCGTGCTTTCGCAGCGCCTTATCCCCCTCCTGTATTAAAATGTATTCTTTTAATTTTTGTGCGTACTTAGCGAGCTCTGGCCCTCCATCGGGTGTTGTCGATACGTACAAATAAATTGTCACGTTGCGCTCACTGGGGGGGAGCTCCATGTGTGAATTTTTTCGCACCACCCTCCCAAATACCTGAATAAGCCGGGGAATATCAAGGGGCAATGACATAATCATCTCGTACCGCACGGCCTGAAAGTTCAAACCCTCCACAATCACCTTGGACCCGATGAGAATACGCAGCTGGTGGCCTTCCAGGTTGGACGAGGCGTTAAAAAGAGCCAGGCTTCGTTCGCGTACAGCGGGCTCTATTTCGCTGTGCAGAATGGTGAACCGTACTGGAATAAACTGATGGTCGCTATGTGTGTGCTCATCGCGAATCGCGGCGCAGATGGAGCAGCGGGTCGTTCCCACAGGGGACGAAACTTCATTTAAAATGCCATTACTTTGTAAAATTTCTTGCAAGATAAGAACCCCCGACATGCGGACCCGATTGTGGTAAATTAAAATTTTCCCCCGGCCTTGCCGAATAATGGAAAGAATGTCTTTCATCATTTGAGTGTATTTTCCGCTATAAAAGGCCAATCCCGAGATGTGCGTTGGTGGCTGCAGCGACAAAAAGCTGCCACTCACATTAAAGGGGGCTCTACGCGAAGGCTCAATAATCTGTACCCCGTTTTCCAGAAGCCAGTCTGTGCTTGCCATAGAAAGGGCGGTGGGGGTTTCCGTCGAGTTAAACAGGCCGTAAGCCTTGGGTTCCGTTTGTTTTGAAAATTTTGGGTTGGGAAACACCATGTCATAAATGCTGTACGCATTACTCGAGATTTTAGGGTCAGGGCCCAGCTGTTTAAGCGTTTCAAGCTGATACTCAGACATGGGGCATTCGATGAAATGTAAGTACGGCAATGTTTCGTCTTTATAGGACAACATCTTTCCGGCAAATATTCTTTCGGGGTAAAAATTGGTGTTGGTATCCAACAAAAAAGATACCCTTCCGGTGCTCAGTCTTTCCACAAGAGCTAGGGCGTCCTTTTTCCATTTAACGGAATGCCCACTGCTGTCAAACAGTTGCTGGCGCTGGAGGGGCTGGCCGTTGGGCAGCTCATGCCGCGGAACCAAAAGGTTTAACAGGTCGACGTATTCCATGACACTCCCGGTTACGGGCGTTGCCGACATGAAGACGGCCCTGGGGGCCTGGTGAGGTGGAAAGGCATCCAGGACATACTGTAAAGCGATGCCATAATTATTTCGTTCCTGGATATTGTACACGTTGTGTATTTCATCCGCAATGAGCAGTCCTCCCCTAAGTTGCTCCATGATTTTTTGATTCACCCGGATGAGGCCGTTTGTCTCGGCCTCGCTAATTTTTTGCACGAACTGAGATATATCGTTCTCATTCAATGTATCTTCTGCTTCGTCAGAACGATGAAACAGAGAAAGCACATCAAAGTTTTTCTCTTCACCCTTACTCGTAATATTGAAAAGCTTGGATGCAAATTCCTTATAGCCGTAAAACTGAAAAAAGCCTCCGCGGTTTCTATCGGTTAAACGGCGCTTTAACGTACTAACGAACCCATTTAGATGCCGTGATTCGACCGACGTGGTGCTGCCAGACTGCTTTGCAATGTGAAGAAGCCGGTGTAGCTCAGCGACCTCCTTGTAAGAAACAAATCCCAGCTCAGGACGTCTTAGCATTTCTGTTTGAATGATGGCGCGTGTAAAGCCTACCACAAAAATCCAGGGCGCATTTTCAATAAAATTCATGTAGTGGTTCATAAATTGACGCGCGATGGCAATCGCGGCAATGCTTTTTCCCGTCCCGGTCTGCCAGTTTAATAAAAGACGCGAGTAGGGCGTGTTGGGATTTTGAAAGTTTTGGACGAAAAGCTGGGCATTATGCAATTGGAGACCCTTGATGGAAGGAAAGGGCGACGCGTAGGGGTCACACGGAAAAAACGCTCGCCCCCCCTTCTCGCAGCCAGGCCCACCGATCTGGACAAAATGAGCCCGCAGATCACGAATGAGCTCTTTTTGGTCGACAGGAGGGGAAATCAACGATTTAAACTCCTTTCTTCGCGCCAACTGCTGCAAAAAGTCTGCGGCATCCAATTCGGGATACGCCATATTATCATAAAAAAAATAAACCTTTTTATGAAAACTTTTATGTGATTCTGTATTGCAATTGTTTTTTATGAATACTGTAAATAAGCGTATCAACTTGTTTTTCTAACGAAGAGGCGTTATTCTTTTTTTCTGGATATAAAATAATAATAAGTATAATAATTAAGACTAAACAGCAGGCAATCACTATCAAACTCATATTATACTTACTTTTTTATAAAAAGTATTATATCTTATGAATGCGCAAGTTCAGCTAATTGTTCGTCGCTTGGAATGTGGGACTGCAGGGAGGTGGAGTTTTTCCTTTTTCTAAAGAATACCGGGAAATGGTGGTGAGGCTCAGGTTGTTGTACATAGTAGCTAGGAGGAGGTTTAGGTATGCTCGACTTGCAGTCAATAGTCCGGTTATAGTAAACGATGGCAACGATGATAAGAATAATAATGAGCAAAATCAAAATGCCCAGGAGAATCGCAGTTGTTCCGGGATATTTGGCGATTGTATGGGCTAAAAGGCCTTGGGTGCTTTGTTTAATTCCCTCGCGGGTTGACAGGTTATGAGAAAGCAGTGGAGACGTTTCAGTGTCCATTTATTACAATTGAACAGTTATATTAATCTCAAATAAAATATAACACAAAATTAATTATGGCCATGCAAAAGTTATTTACGTATATTTACGAGTTTATTGAATATCGTAAGATGGTGCTGTTGGAAGAAAAGGTACCATATGATAAGTTTGTTCAAATGGTACTTAATACAGGATTTTTTCGTATTAACGCGGAGACGCTGAATCACGGAATCGTATCCGTGTTTATCTTTGGAGCAAATGGCAAGTACGTTCACCACGGAGGCGACATGAGAACGCTTTTAACGAATACGCTTAATGAAAAAAAACATTATGAAGAATTAATTTTAATCGTTGATAAGCCCGTTTTAAGCAAAAAAAATATTTTAGATATAATCGTCGAGCAGCGCGCTGCAAATCCCACGATTGTAATAAACATATATCCCTACCACCTGTTCTGCATTAACATTCCCAAGGTGAGTGCCATTCCTAAACATAAACTAATTACTCAGGAGGAGGCGCAGGAGTTTTTAGGTCGCGAATATCTGCAACCGCAGGACCTCATGCAAATTAGCGCGTCAGACCCCCCGGTGGTCTGGCTGGGAGGAAGACCGGGAGACTTTGTGCAAATTGAGCGGCCCTCAGAGACAGCTATGCACGCTGTTGTTATCCGCTTTATCACCAAGTCCAAAATTTGAGTCCCGTGTTTAAAGATGACAGACAGCTAAGTAAGCATATCTGTAAAATTGTCGATGTCCTCTGTGGATAGAGCGCTTTCCTCTGAGCAGCAGATTTTTTCATACGTCTCCATGGGAGATGGCGAGGCTTTAATAGTATGTAGGTCACGTAAGAACTGTTGTATGATGGGATATTTGTCTTTTAAAAACTGGGGATGTTTCATAACTGGAATTATTTGAAAGATAAAGACCTTCCATCCAAAGTAGCCAACCACATTTGGCATTTCGGGACACGCGGTTTCATAAGGCATAGAATAGTGAATAGTGTACTGATCTTTTTGATACAGCGTTTCAAGTAGTTGGCGAAATGTTTCCGCGTCGAGCGTGCCAAAATCTTGAGGAGCCTCGGTGTGCTCCTGTGTAGAGCAGATCGTGATGATTCCCCAGGCAAGCGGGAGCATGGACTCTGGAGGGTGGATATCCGTATTGGTCTCATTATTCGATCCCAGCTGATGAATGCCGCACACGCGAAACATGGCCTCGACGTAGATGCCCATAGAGATAGGCGGCGAAAGGGCAAGACCGGATTGTATTTGCGGCATATAGTAGGAGGGCACCGAGTTTTTTATTTTTCGGTTGAATGGGGACTTTATTTCTACCAGCACGGGGATGCGTTTCGTGGCCTCATAGCGTACGTTGTTAAAAATTGTTTTGATTTCCCAGGACTGTTGAGTGTATCCCAGCGTTAGGTGACAAAACCCATCGGGGCTATTACTATGTCCGGGGTATCCCAAATAGGTCCCATCAATATGAATATTGTCACCTATGACGGTGGTTTGGCAGAACAACTCAAGCAGATCTTTACTAACTCGCTCAAAAAGGGTTCCCCAGCTACAAGCAGCGCGGTTCAAATTCTTCTTAAAAAGATTTGCTTTTTCCGCCAAGATTATATAATAGCTTTTGTAAGGGTTTAAACCTAAAACGCTAGCAAGGTCAGAGCCACCCACCTGAGTGCGACGAATAGCATGCCAGGCATCGGAGCGCTGCTGAGGAGAGTCTTTAAACAGGCGTACAAAGGTTTCCATTATACTTGTTTTAACAGGAATTCAATATAAAAAGTCAACACAGTTTGCAATTTTTCCAATCTCAAGATATAGCCATACATTTTTTTTTCCAATTGGCGAATATGTTTAAGCTCATGTGTTTCAATATTAGCATCCGGAAATTTAAATGCATAAAGATGTTCAAAGGCCTGATTTATACACGTATCAAAGGATCTGTGGTATGTTATTAGCTTCAGCATGTGTGCCAGATCTTCAAGATGGTCTAAATTTATACGGTTTTCCACGTGGTGGATCATGTCTGCCACATCTTGAGCCCCCATCCAGGGGATCACAAGGTACTCCCCCTTAAAGATGATTCGTCGTTTTTTTAAAAAATCATGAAAACGTTTTAAAGCTTCAAGAAAGGGGCAGTTGGGCTTTGACCCCAAAATGCTGACGACGATATCCTCGGGCATGATGTATTCGCAGTGAGGATAGTAGTTTACGGACTCTAATTCAGCGGCCCGCCGTTTTATTTCGTATCTTGCCCAGTTATTCAGAGAGTACTCCACGCCTCCGACCACAACAGACATCCTATCTATTAAAAAATAACAATAAAAACCTTATGAAATCTATGTATAGTGGCCGCTAAAATGTCTATATTAGAAAAAATTACGTCAAGTCCCTCTGAATGCGCAGAGCATCTTACAAACAAAGATAGCTGTTTAAGTAAAAAAATACAAAAAGAGCTCACCTCTTTTTTGGAAAAAAAAGAGACACTCGGTTGCGATTCGGAGTCCTGCGTAATTACCCACCCCGCCGTGAAGGCCTATGCGCAACAAAAGGGACTGGACCTCTCCAAAGAACTGGAGACTCGGTTTAAAGCGCCAGGACCCAGAAACAACACGGGTCTTCTTACAAACTTCAATATTGATGAAACGCTGCAGAGGTGGGCCATAAAATACACCAAGTTTTTCAACTGTCCTTTTTCCATGATGGACTTTGAGAGGGTCCATTATAAATTTAATCAAGTGGATATGGTAAAGGTATATAAGGGAGAAGAGCTACAATATGTAGAAGGCAAAGTGGTCAAGCGTCCTTGTAACACCTTCGGATGCGTTTTAAACACGGACTTTTCAACGGGCACTGGAAAACACTGGGTAGCCATCTTTGTGGATATGCGGGGCGACTGCTGGAGCATCGAATATTTTAATTCGACGGGAAATTCTCCTCCAGGTCCCGTTATTCGTTGGATGGAACGGGTCAAACAGCAGCTATTAAAAATACACCACACCGTGAAAACGCTTGCAGTTACCAACATTCGTCACCAACGGTCGCAGACCGAGTGCGGCCCCTACAGCCTGTTTTACATCAGGGCACGCCTCGACAACGTGTCATACGCCCATTTTATATCCGCTAGGATTACCGACGAAGACATGTATAAGTTTAGAACCCATCTGTTTCGCATCGCATAAACTAATAAAGTTTGAATTCTTTATAGGAATAAAAATGGAAGCGTTTGAAATCAGCGATTTCAAAGAGCATGCGAAGAAAAAAAGCATGTGGGCTGGCGCCCTCAACAAAGTCACTATTTCGGGTCTTATGGGGGTCTTTACCGAAGATGAGGACCTTATGGCGTTACCCATTCACAGAGACCACTGCCCCGCTTTGTTAAAAATTTTTGACGAGATCATCGTAAATGCCACGGATCATGAAAGAGCTTGCCATAACAAAACAAAAAAGGTAACTTACATTAAAATTTCGTTTGATAAAGGTGTGTTTTCTTGCGAAAACGATGGCCCGGGAATCCCCATTGCAAAGCATGAGCAAGCCAGTCTTATCGCCAAGCGCGATGTGTATGTTCCCGAGGTGGCTTCATGTCACTTTTTAGCCGGAACGAACATCAATAAGGCCAAGGACTGTATCAAGGGGGGAACCAACGGCGTCGGGCTGAAGCTCGCCATGGTGCATTCGCAGTGGGCCATTCTTACCACCGCCGACGGCGCGCAAAAGTATGTTCAACATATCAACCAACGCCTAGATATCATTGAGCCTCCTACCATTACACCCTCCAGGGAAATGTTTACACGTATCGAGCTCATGCCCGTATACCAGGAACTAGGGTACGCGGAGCCTCTGTCTGAAACAGAGCAGGCGGATCTTTCCGCCTGGATTTACCTTCGCGCCTGCCAATGCGCGGCCTACGTGGGAAAAGGCACCACCATTTATTACAATGATAAGCCTTGCCGCACGGGCTCTGTGATGGCGCTAGCCAAAATGTACACCCTGTTGAGCGCGCCTAATAGCACGATACATACGGCGACCATTAAGGCCGACGCAAAGCCCTATAGCCTGCACCCCCTGCAGGTTGCGGCGGTCGTGTCCCCCAAGTTTAAAAAATTTGAACACGTGTCCGTTATCAACGGGGTAAATTGCGTAAAAGGAGAACATGTCACCTTTTTGAAAAAGACTATTAATGAAATGGTCGTTAAAAAATTTCAACAAACGATTAAAGATAAAAACCGCAAAACAACATTACGAGACAGCTGTTCAAACATCTTTATCGTTATAGTGGGTTCCATTCCAGGAATAGAATGGACCGGCCAGCGGAAGGATGAACTTAGCATCGCGGAAAATGTTTTTAAAACGCATTACTCCATTCCTTCTAGTTTTTTAACAAGTATGACAAAGTCTATCGTGGATATTCTTCTGCAATCCATTTCTAAAAAAGATAACCATAAACAGGTCGACGTAGACAAATATACGCGTGCCCGCAATGCGGGAGGAAAAAGGGCGCAGGACTGCATGCTACTCGCGGCGGAAGGGGATAGCGCACTTTCCCTGCTGCGCACGGGACTAACCCTGGGAAAGTCCAACCCAAGCGGGCCCTCCTTTGACTTCTGCGGCATGATCTCCCTGGGAGGAGTCATCATGAATGCCTGCAAAAAGGTGACAAACATTACAACGGACTCTGGAGAAACCATTATGGTGCGCAACGAACAGCTTACCAATAATAAAGTGTTGCAGGGAATCGTGCAGGTATTGGGTCTAGACTTCAACTGCCATTACAAAACACAGGAAGAGCGAGCAAAGCTGAGATACGGCTGCATTGTTGCGTGCGTTGATCAAGATCTGGATGGGTGTGGAAAAATCCTTGGACTGCTGCTGGCCTACTTTCACCTGTTTTGGCCTCAGCTTATTATCCATGGTTTCGTAAAACGACTGCTTACCCCGCTGATACGTGTGTATGAAAAGGGTAAGACCATGCCCGTGGAATTTTACTATGAACAAGAGTTTGATGCCTGGGCAAAAAAGCAGACCAGCTTAGCCAACCATACCGTAAAATATTACAAGGGATTGGCGGCGCATGACACCCATGAAGTAAAAAGCATGTTCAAACATTTTGACAACATGGTGTACACGTTTACCCTGGATGACTCAGCAAAGGAGTTGTTTCATATTTATTTTGGCGGGGAGTCGGAGTTGCGAAAAAGAGAGCTTTGCACCGGCGTGGTGCCGCTCACCGAAACCCAGACGCAGTCCATTCATAGTGTCCGACGAATTCCTTGCAGCCTGCATCTGCAAGTAGATACCAAGGCTTACAAGCTGGATGCCATCGAGCGGCAGATTCCCAACTTCTTAGACGGGATGACGCGGGCGCGGCGCAAAATTTTAGCCGGGGGGGTGAAATGCTTCGCCTCCAACAACCGTGAACGAAAGGTTTTTCAGTTCGGGGGCTACGTTGCAGATCACATGTTTTATCACCATGGCGACATGTCGTTAAACACAAGTATTATAAAAGCCGCCCAGTATTACCCAGGCTCCTCCCACCTCTATCCGGTATTCATAGGCATAGGAAGTTTTGGCTCCAGGCACCTGGGAGGAAAGGATGCAGGATCCCCAAGATACATCAGTGTGCAGCTTGCGTCTGAATTTATTAAAACAATGTTCCCCGCGGAGGACTCATGGCTTCTCCCCTACGTCTTTGAGGACGGCCAGCGGGCGGAACCAGAGTACTACGTGCCTGTGTTGCCGCTTGCTATTATGGAGTACGGCGCCAACCCATCGGAGGGCTGGAAGTACACCACTTGGGCCCGGCAACTGGAAGACATTTTGGCCTTGGTGAGGGCCTACGTCGACAAAGACAACCCAAAACACGAGCTACTGCACTATGCAATAAAACATAAGATTACTATACTCCCGCTGCGGCCCTCCAATTACAATTTCAAGGGCCATTTGAAGCGGTTTGGCCAATACTACTACAGCTACGGCACGTACGTCATCTCAGAGCAGCGAAATATAATTACTATTACGGAGCTTCCTCTGCGTGTTCCTACGGTTGCATACATCGAAAGTATAAAAAAATCGAGTAACCGCATGACATTTATTGAAGAAATCATCGACTACAGTAGTTCAGAAACTATTGAAATTCTGGTGAAATTAAAGCCAAATAGTCTTAACCGTATCGTGGAAGAATTTAAGGAGACTGAAGAGCAAGATTCCATAGAAAATTTTCTGCGCCTGCGCAATTGTTTACATTCACATCTAAACTTTGTAAAACCTAAAGGTGGCATTATCGAGTTTAACACGTATTATGAAATTTTGTATGCGTGGCTACCTTACAGGCGTGAGCTTTACCAAAAGCGTCTTATGCGTGAGCACGCGGTGCTTAAGCTGCGCATTATCATGGAAACTGCTATTGTACGCTACATCAATGAGTCTGCAGAGCTAAATCTTTCCCATTATGAGGATGAAAAGGAGGCAAGCCGCATTCTAAGCGAGCATGGATTTCCCCCGCTGAACCACACGCTGATCATTTCCCCTGAGTTTGCCTCTATAGAGGAACTCAATCAAAAAGCACTGCAGGGCTGTTATACCTATATACTATCTTTGCAGGCTCGAGAATTGCTTATCGCAGCCAAAACTCGTCGGGTGGAAAAAATAAAAAAAATGCAAGCTCGTCTTGATAAGGTTGAGCAGCTTTTGCAAGAGTCTCCCTTTCCCGGCGCCAGCGTATGGCTGGAGGAAATTGATGCGGTGGAAAAGGCTATTATAAAAGGAAGAAATACTCAGTGGAAATTTCATTAAACGCTACCGGTTTTATGATGTCCAATAGGTGTTAAGCAATCAGTTCATCAACATTTTTTTCAAGAATTTGAAAAGTTTGGATAATGTTCTGAATACTTTTTTCTAAAAGAGTTATCAAATCTTCTTGTGAGGCCTTATGAATAATTGTTAATACCATTTCTTGCTTATGGGGAACACACTGATACCCCACAAAGCTAATATCAGGAATCATTTCATAAATATATGTTTTTAGCAGATTTCCGATGGTATGGGTTTCATCTTTTATCGTGATAATGGCCTTTGTTTTTTCCTCATCCATGGAAAACAGCACAAGTTCCGGCTGCGGCTCTTCAAAGTTTTCATAAATTTTTTGAATGCTTTGGATTCGGCCAATAATGATCCGGCAGGCGTTTTTTAAATACGTGCGAACGGCCTGGTTGATATGTGGCAGCGGCACCGCTGGAAAGCAAAGCCCCAGGCGGTGGTGACGCGGGTCTGAGGTCATAGAGCTTTGCTTGTAACCGCTAAGCGCCATATATTCTTTTTTATCCGTTGGGTACTGTTCAATGTCAAGGTGGGAAAAATGTGTTTTAACGGCAAGATTAAAGGCGGCATGCTTTCGTCCTATGCCCTTTTTAATATAGATATCCTCTATAATCAACGATTTTCCGGGTTGTAGGAAGCCAATCTCAAAGGTAGGATTAAAAATCGGGTATTTAAGCTTAGGGCCTGCCACCTGGATGAGATCGCGGCTATAGATGGTTTTAACCTCACAGCTATTGTTTAAACTCCGCAGAGCAAATACCAGTGTCTCGTTTTTCGCATAAATCGGAATGAAATTAATGCGGTTTCTAATAAATTGTTCCGTCATAAACAGGTCCGTGGAATCCTCGATCTTATACCCACCGGGCTTAATATCTAGCATATAATTGGGAATTTCATCTTGCAAGACCCGCGACAGGCCGTGGACCGCGGCTCTGCTAATGCCCTTAAAGTCCATAACAACATTGACCGGGACGAGGGGCAACTGCTCCTCGAGCTGAAATAGTTTTTTGGCCGCATTTTTAATAAAGAGGTTGGAAAAGTCTATCAAAAACGGTTTGATTTCCACGTTTTGGAAAATTTTTTCCATTTGTATTATAAATATATCTATATATATTCAAATTATGGTAGTTTATGACTTGCTCGTTTCTTTAAGTAAGGAATCCATAGATGTGCTACGGTTTGTAGAGGCAAACCTTGCGGCGTTTAACCAGCAGTATATTTTTTTCAATATCCAAAGAAAAAACTCGATCACGACACCCCTTCTCATTACGCCGCAGCAGGAAAAAATTTCGCAAATTGTTGAGTTTTTAATGGATGAATATAATAAGAACAATAGAAGGCCCTCCGGGCCGCCGCGTGAGCAGCCCATGCACCCATTATTGCCGTATCAACAATCCTCGGACGAACAGCCCATGATGCCGTATCAACAGCCCCCGGGGAATGATGATCAGCCATATGAGCAAATATACCATAAAAAACACGCGTCGCAGCAAGTAAATACTGAACTGAACGATTATTATCAACATATTCTTGCATTAGGCGATGAAGACAAAGGTATGGACAGCATGTTAAAACTTCCAGAAAAGGCAAAAAGGGATAGCGATGATGAGGACGACATGTTTTCTATAAAAAACTAACGACGTAACAATTAAACAAAAAATAAAAATCATTATAAAATGAATCTTGAATACGTCCAAGTTGTTCAAAAATTTAATCAAGTACTCCTAGAACTTACCAAAAAAGTATGTACCGTTGTGGGCGGGAGCAAACCCACCTATTGGTATCACCACATTAGAAGGGTTTGCTCAGAATGTCCATCCATGCCGATGAGTATGATAGGTCCGTATCTGAATGTCTATAAAGCCCAAATTCTAACAAGGGACAAGAATTTTTTTATGAATTTCGATCCCGCGCATAATGAGTACACCTTTATCATTCAAAAACTAAAAGAAGCAGCCCGAAATATGCCGGAAGACGAATTAGAACAGTACTGGGTAAAACTTTTATTTTTACTTAAAAGCTACATAAAATGTAAGCCCTTTATTAATTAAAGAATTGATGCATAACTAATAAATGGCCGGTCGTGTTAAAATAAAACAGAAAGAGCTCATAGACTCTACTGTAAAAAACAAAAATGTGATGAATCTGTTCCATGAAATTATAGGCTCAAAAGGCAATATTAATTTTAGCGTTGTCTGGCCCAAGTTTAAAAAAATCAAACAGAGCGTTTATGACTACATTTCCACTCTTTCTGTGCTGGAAAAAGCAAACGTTATGCAAAACTTTGAAGCTGATAAGAAACTGTTGGAACTTTTTGTACAAAAGCTGTGGGCTGCCTATGAAGGCTATTTCAAATATCCCGAGATTGAAAAATATGAGGTGGAAGGCCAGGTAAATTTCAATCTCGTACCTCAGTGCGTCCTCGAAAAGTTTAGCCAGTTGTATAGGATAAGAATCAATTCAGAGCTTGTCACACTCATCCTAAACAGCTGTGCCTTTATGAGTAAATATAACGATTATATTCTCAAAAAAGATCCCTACATACTAACCATAACCCCCGGCCTATGCTTTTCCCCCATTCCCAACTTCGAGGACCTAAATTTTAAACATCTTTACAACAGTGATAAAAATTCTCAGCATGACAAAGAGTTTATCATGTTTATATTATATAAGCTTTATACGGCTGCCCTAGGAGTGTACAATGCCATCTCGATTCCAGACATCGACGTAGAAGACCTTGAAAATATCATCCTATCCTCGGTGAGCCAGATTAAAAAACAAATTCCGCGCTGCAAAGACGCCTTCAACAAAATTGAATCTTCGGTACACCTGCTGCGCAAAAATTTTAACACATATTACAGTGACTATGTGGGCTCAGGCTACAACCCAACCATCATTATGGAACAGTACATTAAAGACATATCACAGGATTCCAAGAACATATCACCACGCATTTCCTACCAGTTTAGAACCATCATCAAGTATTACCGCGACATGATCGCCACCAGGCATCAAACGATGGACCCCCAGGTATTAAACCTCGTAAAGCACGTCGAAAAGAAATTAGATATGCTTGATAGAGAAAAAAATTAGTATATATAGTTATGGTGAATCTTTTTCCTGTTTTTACCTTAATTGTGATTATTACAATTTTAATTACGACTCGAGAACTATCCACCACGATGCTTATTGTTTCTCTTGTAACAGATTATATTATTATTAATACACAGTATACGGAACAGCAGCATGAAAACAATACATTTTCCATGCCGCAAAAAAATTCTTTTAACGAATCTTATAATAAAGACAAAAAATCTAATACACATATTCCCTACCAGTGGCTGGCGCCTGAACTAAAGGAAGCTGAGAGCAAGTACTGGTGGGGCAATTATGATCCTCATAGCGAGCCCGTTCTCGCTGGCGCATCTTGAATATCTTCATACGTGGCACGTCACCATCAAAAACATTGCCCAACAACACGGGCTTGATATAAAGGTGGCCATTGTGGTCTCAACATCGCATTTAAATAATTTTTTGCCAATTTCCGGGGCGCTTAACATCGAATGTATAACCTTTCCCAGTTGCGGCATCAAGGAGATAGACCTCCTATGGGCGCGCATTAAACTATTTCAACATTACTGCGCCATCGGTGCCCGTCTTTTATGGCTGGTAAGTGCTGACATCAGGCCCCCTGTTTCAGCGTGGCCAGCCATCGCTGACAGTCTAAAAAAGGGAGCAGATGCGGTGGTTATTCCCTATCCCTCCCGATGGAACAATCTCATACCTACCGTCATCAAAGAAATAGTTGTCCACCAAAAAAAATGCCTTGTGGCGGTGGATGCACGCCACCTTGATACAGATACCCAGATTGTGGGGGCCGGGATGGGCTGCATCGTCCTAACCCTAAAGGCCCTTATGGTGCGTCTAAGTATTGGCAAACAGCCCGTTAAGATACTGTGGCCCGACCTTCACGGCACTGCCGAGGGCATTCCTCTGGAGGGGGTGGAGGTTGGCTGGTTTTTAAACGCTTATGCGCATAAATTAAATATACGCTGCCTAGGGGCTGATCATATTGCGCAGCACTTAACTTAATTCTTTATTTAAAAAGCCCACGCATCCAGTAGCGGCCTACATTAAGGGCCTACGCACATAAATATACGCTGGCTAGAAGTATGCCTTCATTTAAACCATTGAATTATTTATATAATGGCTGTAAACATTATTGCAACAAGAGCCGCGCCAAAGATGGCCAGCAAAAAAGAGCATCAATACTGCCTGCTAGACTCCCAGGAAAAGCGTCATGGGCATTATCCCTTTTCATTTGAATTAAAGCCTTATGGGCAAACAGGCGCAAATATCATAGGAGTACAGGGCTCGCTTACCCATGTTATCAAAATGACAGTATTTCCATTTATGATTCCTTTTCCTTTACAAAAAACTCATATAGATGATTTTATTGGTGGACGCATTTATTTATTTTTTAAGGAACTGGACATGCAAGCAGTTTCTGATGTAAATGGAATGCAATACCACTTCGAGTTCAAGGTTGTTCCTGTAAGCCCCAACCAAGTAGAGCTTCTTCCTGTGAATAATAAATATAAATTTACATATGCTATACCGGTGGTGCAATACCTTACCCCAATCTTTTATGATCTTTCGGGACCGCTAGATTTCCCATTAGATACTCTTTCGGTCCATGTGGATAGCCTCTCCAATCATATACAGCTTCCTATCCAAAACCACAACCTAACAACGGGTGACCGTGTTTTTATTTCTGGATATAAACACCTGCAAACGATTGAATTATGTAAAAATAACAAGATTTTTATCAAATATATACCGCCGCTTTCATCCGAAAAAATAAAACTATATATACCAAAAAACCGAATCAGAATTCCACTATACTTTAAATCTTTAAAAAACGTCTAAGTAATAACATTTTTATAGTCTACTCCTAGTTCCGAAATAGGCTGAATTTCTTTTTTAAGTCCTTTAAACCAAGGATGTGATACAAGACTCTTAAAGGAAAGCCGCTTATTTTCATTAATTGTTAAACATTCCGTGATAAACTGTTTTCCCGTCTCTGAAATGTTCTCGGGAATATAATTTTCCCGTTTCAGAATATCATTTAAATAAAAATTTTCTGCACGAAATCTAAAAAGATTAACCGCGACCATACCTATCGTCCACACAGTTAAAGGAAGCTGGTAATAATAACCATAATAATAAAATTCTGGACACACGTATTCCCATGTTCCAAACATATTATATTGGGGACGGGTTTCGTCTAATCTAACAGCGCTTCCAAAGTCAATGACCTTAATGATCTTTTGATTTATGTCTATAATAAGGTTCTCATCCTTAATATCCCCATGGATAAAGCCCTTCTCATAAATGTTTTGTATAATAAGAATAAGCTGGAATATTATTTTTTTGGCTTCGGTTTCCTCAAGTTTTTTAAAGTAATGATAATGAAGTAGATCAACACTATTTGGAATATATTCTATAATTAGTATATGATACATAGCATTTTCGGTATATTCGATAAGCTTAATAACACCGGGAGTATCTTGCAGGGCTTTCAACACGATGACTTCATTTCCTGGAATTTCTTTTTTAGAAACGTACTTAAATATAATGGGTTGCCCTACTTGATGACCCAAAAAGACGTTATTTCTGCCACCCTCAAACATGGGTCTCGTCGCAATGAAATACATGTGCTGCGTTGTGGAGATCCTTTCCACCTTTGCTGTAGGATAAAACGCATATTGTGCCTGGGGGGTTTTTAACATTTTTTTAAGCTGTTGTTCCGGCCTGGACATGTTTTATTAGCTTTATATATAAAGGGTTAGAAGGTTTAATTTCAATATATGCCTTAATGATGGGATTATATTCGTAAAAGGTATAGCCTAATCCTACGTCTTTGTTTTTTTGGTAAAAAAACTGTTTGCCCTCGTAGGATATGCTATAGGCTTTTACTTCGGCTTTTACAAGCGGTTGGCAGGGATTGGGCAAACGTAAATCGCGTTCAAAGTTTTCATGAAAAAGCAAAGCATTTGTGGGCTGACACATCAGACAGCCGCTTTCGCCGTTGAAGGCACATTCAATGGCCGCCCTTTTTAGTAAATCGCGGAAAGCAGAATTAAGATGGCTCTTTTCAAGCCCCCTTTCGTGAAAACGCTCATCAATCGTTTTTTGTTCCTGACTGCCTTCGGGAATACTATAAAACATTTTTTGATTAGCCACCGCGATGTACAAAAAAGGCTGCACGGTTTTCTCCTCGGGCGGTAGCGCATCGTGGCTACCAATGCGTATAATGCGCGCCTTCACTTGATCCTCTCGGGCCTTATCCCAGTACGGCTCTAGGATATGAACCTGCCGCCCGTATTTGAGATCCAATCCCTCAGCTCCCGTTTTAGAGACGAGTAAAATTTTAATAACCTCTCCGTGTATATTCAGCGGCGAATTCCAAACCTGCTGGATCATGTCGCGCTCTTTAGATAAAATTTTCCCTGTAATAAGCGTAAATCGTGTTATTTTGGAGGACAGGACTAACGTATGGGTCGGCCCATCTTCGGCAAAGTTTTTCACCATAAGATCTTTCCCATCCTTATGAAGGAGGATGGTGTTGTGCCCTTCTTCCAATACTTTTAGGGGCTGAAGGCACTGGTAGCCCTCTATTTCTAAAAAGCGGGCCACGACGTGAAGGCCCAATTCCACAAACTGTGAGTAAATGAGCACAGGGCCCGGAGACGTTTTAATATTTTTTAGCATGCGCACTATTTTGGGACTAGAATTTTCTGTGAAGGCCTCTTTGGGCAGCTGCTGAACAGCCTCTGATAATTTTTCATCCTCCTTTACTGTTAGCATTTCGGACGCGAAGATGCTGATCATACGGGAACGCACATAGTAGGAGGAGCCTGACTCTTGCTCCGATCCTGGCAGGCAGAGGGCGGCGGCATTAATTTTTTCATACATTCCTGAGCTGGCGTGCTTTTCCGCGTTTTCAACGTCTCGGGCCAGCAGATATTGCCTATACTGCTCGGGTGACATTTCAACCTTTTCTATAATAAGAGGAAGCTCTGTGGGGAATAGCTTGTTGAGCTCATTCTGGTTTCCAGCGTAGCTTATCATACCCACTAGGCGGTTTAGTAGTTTGTCCGCGTTTAAAGGGCTATTCGTTGTTTTATTGACATAAGCGGTGTAGAATCTTTCATAGTGAAGAGGTAATAAGATTCGCCCGCTTAGCATATTAAAACAGGGCACCATTTCAAAGGGGTCCTTCGAACACGGGGTGCCTGTTAAAAACAGAATACGAATATTTTTAGCTTGCATAATATTATTGTACAGCTGGCGGGCATTTGTTTTATCATTGGCGCTATTGATAATTCCTCTAAAGAGGTTGTGTGCCTCGTCAACGATGAGCAGGCAACCGTTTAGGGACCCTCCCGCCTTTATGATCTGCTGCCCCATGTTGTAAGCGTCTAGGGACACAAACCTGAAGCGCCGCGAGATTTTTTGTAGCTCTTTGGAGTGATCCGTCGTTTCCGGATATAAAAGTTTAATAAGCTTTAGCAAAGACTGTTGGAAGTTTGAGTGCAACGACTTGGGTGCGATCAGAATCGGGTTGTAAATATGTGAAAGTGAGATGGCAAGCGACAGGCTCAAAATGGTTTTCCCCATGCCCATCTGGTGATAGATGAGGAGGCCCCGTGTGTTTTCCCCCTGGCCTATCCCAAATTTAGGATCCGAAAAGGCGGTGTAAATTAAAAACTGGTAGTATTTCAGGGCTCGTGCAAAGCGGGCAGTGAGTGAGGTGTCTTTGCTTTCCTGAAGCTCTTTATATTTTTCATATACCTCTTTTAGGTATGCTTCTATTTGGACGGGGAAGGAGGTGTTGTTGTGCACGCAAGACATGACTCGTTATAAGGATCCCATATTAAAACTTCATTAGAAGAATAGGGCTGCTGATAGCTAGCGCTGCACTTAAAAATGGGGTAGCCCTTTTTCTTGTAAATCCGGTGCCTGTCGTAGACCTGGCTAGAAAGCGGGCTTAGTGTATCTTTAATGTCCACAACGATGCGTACCTTTTTTTCATCCGATCCCTGCCGGGTAATACGTCCCAAGATTTGCTCCATGTTGTTTCTGCGGGGCGTTGCCATGATGATCGATGTCATATGCTTGAAGGAAATGCCTCTACGCCCGTAGCCATAGGTCAGCAAGATAATGGAAGCGCTGTGTGCCTGAGAAAGAGCGGTATTTGAAACCCCGCCGCATAGGAGCGCCACCTCCGGAACGATAATTTGAACATCTTTGAATTCTTTGGAAAGCGCCTGATAAAAAATTTCTAAAAGTTTGCGAAATTCCACGAAAATGATGATGCCATACGGCTCATCGGTCCCCCATTTGTGAGGCTCAGCGGTATGCAGGGAGTAAAGCCGCTTTGCCTCATTTACGACAAGTTGTATACGCGAAGGATCTTGAAGTAGTTTATCAATGGTGGCAATGGCCGATACCTTTTCATTAATATACACAGGGCTAACGAAGTCAGGATGTCCCTGATATTCGATTTCCCTCACGTACCCGGAAAAGGTTGTGGTGGGACTTACAGTCCTCTGGGGCTGTCCTAGATGGTGAATAATAATCTTGTCCATACCATCGGGCCGGTCCAGGGGTGTAGCGGACAGTCCTAATATCCGACTAAGTTGTATTTTCCAAAAAATTTTGTAATTCTCCGGCGAGTGTAATTCATGTGCCTCATCTAACACGACTAGACCAAAGGGCTCAAAGAACTGCTCAGGCTTCTTGCGCAGGGTATTAATGATTCCCACGATGACGTCGTACTCTTTGCTCGTCATGTCCTTTTTCTTGCACGCTGCATTATTGTAAGCAGCTACACGTAGGTGGGGCAGGAGCAATGTTAGCTCGTCGATCCACTGTATTTGAATCGCCTTGGTGGGCACGATGACCAGGGTAGGGTACAAAAGTTTTTGAATAATGCTGATCGCAATACGCGTTTTCCCCAAACCGGTATTTAGATGTAGGTAAAAGCGCCCATAGGGGGACAGGAGCTTTTTATGAATCTTATCGACCATTTCTTGCTGGTAGTTAAATAGTGGAAATTCTGTTTCAACGCATGGGAGGGCCCGCAGCGACACGGGGCGCGTCGTGTAAACCATGTTAAACATTTCAAACTGCTTTTGCAGCAATATGGGAAAATAAATGTATTCCCCCTGCAGCGTGAAGGCAGTTTCCTGTCTTATGGCTATGTGCTTTGGCTGCCCGGGTAATGCCCGCGCCGTAACGGTGAGCGCCTTAAGAACGCGCCCGAAATCATGTTGTAATTTACTTTGTAGCTTCTTATAATTTATTCCTATTCCAGCAAAGGATATAATGGCCTCCATTCTCACGCTGGACGGGTTATATGCAGAGGTTCCAAAATTCTTACCAGAGGCGTTACGAGAGGGCTGTGCTGGCAAGAATCCTCTAAGCTTTTATATTCAACAAATTTTAAATTTAATGGGATGTGACGGTAACGAGTACCATGTTCTTTTTACCAGCAGCTCCGAGGAAGCAAATACTCATATGATCATGGCCGCCGTGCGTCGCCATTTGCTGCGGACGCAGCAAAGGCCTCATGTCATTATCGGAGCAGCCGAGCCCCCTAGCGTCACCGAATGTGTGAAGGCATTGGCGCAGGAAAAACGCTGCGTATACACCATCATCCCCCTAAAAAATTTTGAAATAGATCCTGTTGCGGTATACGATGCCATACAAAGCAATACCTGCTTAGCGTGCATTTCAGGCACTAATGCTGTTGTCAAAACGTTCAACAAACTCCAGGACATCAGCAACGTGTTAAAAGGTATTCCCCTGCACTCAGAAGTGAGTGATCTTGTTTATCAAGGATGTATTCAACAAAATCCGCCCGCTGATAGTTTTTCAATAAATAGTCTCTACGGCTTCCTGGGAGTCGGTGTTTTGGGAATGAAGAAAAAGGTCATGCAAGGATTGGGGCCGCTCATTTTTGGAGGAGGGCTGAGAGGCGGAAGCCCTAATATACCCGGAATTCATGCCATGTATAAAACGCTAACCCAGCAAAGGCCTTCTATGAAAAAAATAAATACAATACATACGCTGTTCATGAAAACTTTAAAAAAACATCAGCATGTATATCTACCCATAGGGGGCGTGTCTGCAGAGGACACGTCTGCAGAAAACATATCTACAAAAGACATGCCTGTTGAAGGCCCGAAGGGACTCCCGGGCTATATTTTATTTAGCGTTGGCCGTCGCGCCGAGGAGCTACAAAAAAAAATTTTCACTAAATTTAATATAAAGGTTGGCCGTGTTGTTGACTTACAAGAGATACTGTTTCGTATCAAAATACCCCAAAAATACTGGGAGACATTATTGTTCATCCAATTAAGAGATAATTTGACCAAAGAGGACATAAAAAGAGTTATGGTTGTTTTGATGCATTTAGATACCATCACTCCTCGTGGCTCTCTTCCTCCTCCGAGCCACTCTTCTTCTTTTTCTTAATCGTTTTTGTTTGTTCTATAATAAGGGAAAAGAACTCCGTGGGATCTTGTTCCCCGTACAGGTTATCTGCGACCATAAGGATGCTTAGAATGGTAAACAGGTGAGAATACATAAGGGTTTGCGTTTTAAGAAAACCCTGACGTTGAATCATAATTGAAAACACCTTGCAAAGCCGACTCATCAGTTGTTCTGTAATGGCGTTAAGCATTTTCTGGAATTTTTCTTGGTTTTCGGGTGTGATTTTATATTCATGTAGAAAGTGTTTCACACCTGAGGAGAAGAATCTTTCCTCCTTCGAGAGCCCATCTTTGATGATGGGAAGTTCCTTGATCAGGGCAAACCATTCCTCCTCTTGGGCTTGCGGATTCTGAAGATACTGATGGCAGATATGGTTTAGAATGGTGCACACGTAGCTAATAAGCTCTGAGCTGATTCTTTGGTTGGTTTTCAAATGTTGGCGAAAGTAGTTTTTCACCGAAGTGCATGTAATAAACGTCTTCATTTTCTTATAATATACAACAGTATGTTGAGTCTTTAATTTAAAATTACAAGGAGTTTTCTAGGTCTTTATGCGTATAGGTGTTTCTTTGTCGTAAATTTTCAATAGCCGACATTGTTTGTGAAGCAGTGTTCTGAGTAGTGACTGTCGTGTAAGGCTCAGCCGGATGAGCAGGAGCACTCGCGGCCGCAGGTGCGGCCGCCGGCCCGCCAGTTGCCATGACTAGTCTGTCCGTAACTGGGTTGTCCGTAACTGGTTTGTTTGTTGCTGGTCTGTTTGTTGCCGGTCTGCCCGTGACTGGCTTGCCTACACTTGCTGTAGTCGCTCCAGCTGGTTTAGAGGTACCTGGTTGTGGAGTGACTTCTACCCACTGCTGATCTTGATAAGGATTTATAAACTGTATATCTTCCTCCTCAATAGCAGCAGCTTTTTTCTTTCTTGAAGAGAATAGATAGATTAGAACGATGATAATGATGACTAAGACCACGATAGCAATGAGAATAGTATACATATGTGTGGAGAAGAAGCTTGGTGTAGTGACTGGTGACAAACACTCACCATAATGCCGCGGATAAACCGGTTGAAAAAATTCAGAATCCATTTAAGATACTATTATAAATAATATATAAAAATGTTGTGGCGCAATGAAATTACAGAATTTATGGACCAACTTTCCAAGTATTCTCAAGAAATCTTAAAAACGTTTAAGCAATTGCGTCCTAGTGAATATAAACAATACAATGAATTTTTAACACAAGTTACACCGTTGCTGCAAAAAACCCCTGAAAAAATTCCAGAGTTGGTTGACCATATATTCAATTACCTAGACAACGTTGAAAAAATTTGTGAGCTCCTCGTGAATGCTAGCTCAATTATTATTAGTTCAAAAATACGAGAACAAGTAAAACACGGAATGAGCTTCAGCTATAAAGCCGACCTCGACTCCTTGGCGGACATTCTCTCTCAAAAACAGTACGTGCTTATGCATCTTTCAAAAAATATTGCGGCCGAGTATTTTAATACGTGTTTAAACCAAGGGAAATCCAAGTTAGATCTCAAAGCTGCCTCTGTATTTTATAGTAGTCGTTCCCGAACGGCAAGCTCAGCAGAACTCTATAGAAAAATGCTATACGCCTATGGTTCACCGCAGGAAATTAATTATTATACTGAAAAAGCCCGAAATAAGACGTTGGATGTGGAGGAGAGCGACAGCATGGCCATCATCGAACGAACGGCCCGACACAACCTTTCCCTTATGCACCCGCTAGAAGCCATGGGGCTTACCTTTGGGGCAACCAACACGGACGCCGACCCGGAGGATCTGAAGGACAAAACGGTGATAAATTTAACGCTCCCGCAGGCAACAGAAAGCATCACCTACCATCTTAAATCCCTAATGCAGCTAAAAAAAGTAAGTACGGCTTCAGGACTAAATACAAACATTTTGAAAGCATTTGATAATATTATTTCCACCCCTGTGAAAAAAAATAAAATGGCCTCCAAGTTGGCGCCCGGGATGGATGTCGTGTTCACTAGCGATAACGGAAAAACATTTTTTACTAAAAACATTTTAAGCAAAAACATGCTAGCGGGGCCCAAAGAGCGGGTGTTTGCATATAATAATCTCATTAGTAATTTAAATAACTCCTGTTTCATACAAAATCACAACGATTTTTTAAGACAGCAGGACTCTTGGCCCTTCTATGACGCGCACAATTTTACCAACAAGTTTTTAATGCAGCCTATTTTTTCGGGGCAGACCCGTCCTCGGCTTCAGGGAGCCATGGAGGCGGCGCATGTGGAAACGCATCTCACGGCATTTTTACAAAGTATTCAGCCCTCTAGGCCACAAGATCCCTCTGTTTTGGCTTCCCCCAAGTTATCTGCTCTAATCTTGAACTAAAAACAGCCTTTCTTGGACTTAAATGATGGTCTACCAGTTTTTGAAATAACTTAGAGAACTATGAAGATTTTCATGAAATTTAAATTAGAGATTTGCAAAGGTTACTTGCGGTCATTTTCTGTTGAATTAAATAATTATTCGAATAGTATAATGTCTGAAGATATTCGTCGTGGTCCTGGCAGACCGCCAAAGAAAAGGGTTGTTCCCAACTTTGAGCGCAAGGGCATTCTGGAAAAACCAGTTCGGCCACAAAGCCGTCTCGAGTTTTCCTATGATAACCCGCTGATATTTAAAAATCTTTTTATTTACTTTAAAAACCTTAAAAGTAAAAATATTTTGGTGCGATGTACCCCCACCGAGATTACCTTTTTTTCACGTGACCAGTCGCAGGCAAGCTTTGTTATTGCCACCATCGACGGAAAAAACGTGAACCATTATTACGCCAGTGATGTCTTTTGGCTAGGCATCAACAGAGAGCTCGTTGAAAAAATGTTTAACAGCATTGATCGCTCTTTTTTAAAAATTACCATCGTTCACCGCTATGACAAGCCTGAAACCCTGTTTTTTATCTTTACGGATTTTGACATTGACAAGGAGTGCACGTATCAGATTACGGTCTCGGAGCCCGAGCTCGATATGGACCTTATCGAAATGGAAAAAAGCATCAGTGAAGAAAGACTCAAGAACTATCCTCTGCGCTGGGAGTTTACCTCCAAGCAGCTCAAGAAAACATTTAGCGACTTATCAAACTACACCGAGCTCGTGACCATTGAAAAACTCGGCGGCGATACGCCGCTGCACCTGTATTTCCAAAAGTTTAACTCCATCTCATACCACGAGATGTATAAATCTTCCAACAAGATCAACCTGACCTCGACCATTCCTAAGTCGCAGGTGTTCCAGATAAATGTTAAAATTGCTCACATCAAGTCGCTGGCCTCGGCTATGGTCACCGACAAGATCCGCATTCTGTGCGAAGAAAATGGGAACCTAATCTTTCAATCGGAAATGGATGCCCTTATGTTAAATACGATTACCTTGAACACCACGATATAGTTCGGTAACATTAGATGTTCTAATATTTAGCATCTAAATAATACGCTGTAGTCCGGTCAGGGTTGCGTCACAGTTTTCCCATTTTTTTGCCTCGTCGGCGGTGGCCACCGTTGCCCTATCATTTACGCCCGGTAAGACAAAGCTAAAGGCGTTCAGCGGGGCTTGGCAATGCCCGCCCAGCGTGAAGGAGCTCGGAGGATTTTGCGCATCCCGAAATCCCTTAGCCATGTTGTTTAACACTTCGGTTACGTCAATCGAGTGAAGGGATCCCTTGGGATCCGTGAATGTAAAGACGCAGTTTCTAAAGCGCATGTATGCGATGGACGATTCATCGGGGGTTTTGAAGGTAACAGTGTTCCCCTTGCTGTACTTAAAGGGGGACCATCCGGTAAAATTATACCAAATGAAAGCAATAATAATTAAAATAACCAACACAATAGTTATAGACAACACAAAGTCTGTAGTGCCGCCCATTATTAAATAAAAATATTTTAGACCGCCGGCTTAAAATTTACTTATTGCTCATAGCTTAAGTCTATTTTATTCATAGCTTAAGTTTATTGCTCATGGCTTAAGTCTATTGCTTATAGCTTAAGTCTATTTTATTCATAGCTTAAGTCTATTGTTCATGGCTTAAGTTTGTTGCTCATAGCTTAACTCCATTACTGATAGCTTACTGATCATGACTTAAATAAAAATATTTTGCCCGCTTAAAAATTGTTTAGGTTTGAAAAAATAAGAGATGGAGGGGGCAACTTATCGTCATTGTGTTTACCCCCACTGGAAGACATCAAACGGTAAATAATTATAAGAATCAAAATGATTAATATAAGGGTTAAAAAAGGATGATTCATCACATTAATTAAAAACGTATTTATAACGCTGTTGCAGTTGAAATTTTGGTATAGGTCGGAAATATTGCCCGAGCCTCCGTATTTTGCAATGTTCTGACATATGGTGAGTCCGGAGGGGCACTGCTTGTTGGTCAAAATATTTCTTTGCTCCGTTGTTTTATAGGCATTTTTATTTCCATTACACGGAGCAAACGCACATTCAGCCCATAGGGTGCCGGAGTTCACACAGGCACAATACTGGCTATACGCATACTCATCCTTTGAGCACAATCCCTGTTTATCGCATATGCTCCCAATAATATTGTCATCCTCCGCCGTTTGTTGATTTGTATGCGAGCGTAAAATAGCGGCCCAGGCCTTGGGCTCCTTTTTTTGCAGCTCGGAAATCGAAGGGCCTGTACAGCTAAAGTCGACCCAAATATCATTGCATTTCGTGGAAACTGGCATGCAAGACATAATTGAAATAATTAATAAGTATATATCATGGCAACAAATTTTTTTATTCAACCTATCACCGAAGAAGCTGAAGCATACTACCCACCTTCCGTGATAACGAATAAACGGAAGGACCTGGGGGTAGACGTATACTGTTGCTCCGACCTAGTGCTTCAACCTGGACTAAATATTGTTCGCCTGCATATTAAAGTAGCATGCGAACACATGGGCAAAAAATGCGGTTTTAAAATCATGGCGAGAAGCAGTATGTGCACCCATGAACGGCTGCTCATCCTTGCAAACGGAATTGGTTTAATAGACCCGGGTTATGTGGGCGAGCTCATGCTCAAGATCATTAATCTTGGCGACACCCCGGTCCAAATATGGGCCAAAGAATGTTTGGTGCAGTTGGTGGCCCAAGGTGACCATGTGCCTGACCATATCAACATCCTAAAAAGAAACCAAATATTTCCGCTGTTTGCGCCTACCCCAAGAGGCGAGGGTAGATTTGGGAGCACGGGCGAGGCCGGGATTATGAGAACTTAATTTTATTTTTTTTCTTAACATAATGGGAGGCTCTACAAGCAAAAATTCCTTTAAAAATACGACCAACATTATCAGCAATTCCATTTTCAATCAGATGCAAAGTTGTATTTCCATGTTGGATGGCAAAAATTACATAGGCGTATTCGGTGATGGAAATATTTTAAACCACGTTTTCCAGGATTTAAACTTATCATTAAACACAAGTTGCGTGCAAAAGCACGTAAACGAGGAAAATTTCATTACAAATCTTTCGAACCAAATTACTCAAAATTTAAAAGACCAAGAAGTTGCGTTAACCCAATGGATGGACGCAGGAACTCACGATCAGAAAACGGATATAGAAGAAAATATAAAGGTAAACTTAACAACCACACTTATTCAAAACTGCGTTTCATCCCTGTCGGGTATGAACGTGCTGGTGGTGAAGGGGAATGGCAACATTGTTGAAAACGCAACTCAGAAGCAGTCGCAGCAAATCATCTCTAACTGCTTGCAGGGGAGCAAGCAGGCCATAGACACCACAACCGGCATCACTAACACGGTAAATCAGTACTCACACTACACCTCAAAAAACTTTTTTGACTTCATTGCAGACGCAATTTCGGCTGTTTTTAAAAACATCATGGTCGCGGCTGTAGTTATCGTTCTAATCATCGTAGGGTTTATAGCCGTCTTTTACTTTTTGCATTCACGGCACCGCCATGAGGAGGAAGAAGAAGCTGAACCACTCATAAGCAACAAGGTATTAAAAAATGCTGCCGTTTCGTAATAATTTAATTAAAAGTAAAAAAAAAAGGTATTGTTATAGTGATGGCAGATTTTAATTCTCCAATCCAGTATTTGAAAGAAGATTCGAGGGACCGGACCTCTATAGGTTCTCTAGAATACGATGAAAATGCCGACACGATGATACCGAGCTTCGCAGCAGGCTTGGAAGAGTTTGAACCCATTCCCGACTATGACCCTACCACATCAACTTCCCTGTATTCACAATTGACCCACAACATGGAAAAAATCGCAGAGGAAGAGGATAGTAATTTTCTACACGATACTAGGGAGTTTACTTCACTGGTCCCCGATGAGGCAGACAATAAACCGGAAGATGACGAAGAAAGCGGTGCAAAACCTAAAAAGAAAAAACATTTGTTTCCAAAATTAAGCTCGCATAAATCGAAGTAAAAATTGAAGCGAAAAAAAGTAGAAAAAAAATGTTTGGAGCTTTTGTAAGCCACCGTTTGTGGTCAGATAGTGGTTGTACGACCACCTGCATCACAAACAGCATTGCTAATTATGTAGCCTTCGGCGAACAAATTGGATTTCCCTTTAAATCAGCTCAGGTATTTATTGCCGGCCCTAGAAAGGCTGTGATAAATATTCAGGAAGATGATAAAGTTGAGCTTTTAAAGATGATTGTTAAGCACAATCTTTGGGTTGTTGCTCATGGAACCTACTTAGATGTGCCCTGGTCCCGTAAGAGTGCGTTTGTTACACATTTTATACAACAAGAACTACTTATATGCAAGGAAGTCGGTATTAAAGGGTTAGTTTTACACCTAGGCGCTGTGGAGCCTGAACTTATTATGGAAGGACTAAAAAAAATTAAGCCGGTTGAGGGGGTTGTCATTTACCTGGAAACCCCGCATAACAAACATCATACATATAAATACAGTACAATTGAGCAGATCAAAGAATTGTTTTTACGGATACGAAATACCAGGTTGAAACAGATTGGTTTATGCATTGATACGGCTCACATCTGGTCTTCCGGTGTCAACATCTCCAGCTATAATGACGCGGGGCAATGGCTGCGCTCGCTGGAAAACATTCATTCCGTGATCCCACCAAGCCACATTATGTTCCACCTAAATGATGCCGCCACAGAATGCGGAAGCGGTATAGACCGACATGCAAGTCTTTTTGAAGGAATGATTTGGAAATCATATAGCCATAAAATAAAGCAAAGCGGTTTATATTGTTTTGTTGAATACGTTACGCGACACCAGTGTCCGGCTATATTGGAGAGAAACCTCGGGTCTTCCATGCAATTACAAACCGCTTTAACCGCAGAATTTACTACATTAAAATCGTTATTAAAATAAGGATGAGTTTTAGCGAATGTCCCTTAGTTATTAGTGCATGCAAAAAATTTCTACAAAAGCGTATTACAATAGAGAATGAAGCACTTATAAATGCCTTAATAACCGCTTTAGCGCAGACCAGCACGTTGAATGATCTTTGTTTATTACCTATTCAAACCTATTTGCTTAGTTATAAAAATGCTTTTGAGTGGATACACTTCGTATGTATTGCAATCACCACTATTTTGGATAATAAGTATAACTGGAAGGACTGTACGGTAGATATTAATTATATTTTTCTCCATGTAACCTATATTTACAATATTAAAACCAAGGAATACCTAGACTACTGTTCTTAAACTTTATTTTTTCTATATTTACGCCAAAGAGAATATTTAAAGTTTTTTTTGAAAAAAAATAATATATGTAGATAAAATTCAGTTACATGATATATGTGTAAACATGTGTGGTAAACAACATATGGTTATGCTTTATAAGATAAATGCGCATAATATATGTAAACAAAATATGGTTATGTGTTAAATGCATATAAATGTATTTTAACGTATATCTTGTGATAATGGATATATGCATTTATTAAAAGAGGCTGTATTTATTATAAATCTTGCTAAGGATGCCATTGTCAACATATATCCCATGTTGGACAAATTGCGTTGCGATCCAGTTCTTTTTTTTTGATTTTGTTTAATGCTATCCTTTTTGAAGGGATGGTTGTCCACCATATTTATTCGATGTTCAATGAATAGGTCTGCTTTTTCGTAAGGCAGTGAAGGTCGTTCCAAGACTCCTTGAACGAAGGACGTGTTTTCTTGGATCCACTTAAAAAGCACGTGGCATTCAAAAACAGGACAGTGATTGGATCCTTGGATATGCTTTGGACAGCCAATGCTTGAAGAGATGTAGTCCCTTTTCTTTAGGACAAGCTTCTCCACGCTGGGGCAACAGAGATCGTTCAAGTTCTGGACGGTCGCATTTGGAATGTTGAAACTTCGTATCCATTCACCCTCGGGTCCTCCCTTATGAAGAAGGAGTATTTGCTCATGGTCCTTAGTAATCTTAACCAAATGTTGGAAGATCATTTTTTTACCTGCTTTAAAGGCCTGAAGGGTGTCAGTTGGCAAAGCTATTGAATTCGGGAGTGGGCTTTCATCAAGCGTGAAATGGTGAATGTGACGCGACTGGAAAGAAAACGACCGTTGATTTATTTTTTCAAAGATTGGGTCGATTCCGCCATGAAAGAACAGCTGCAAGATTTTAGAAGGCGTATTTTTTTCCCAATAAAAAATGACCACTTCTCGTGGGATTAAAATCGTCTGTGTCCCATTTTCATTATATAATTGGCCCATAAAGCCATCAACGTCAATCAACACCAAAAGCATGGTATAGAGAGCTTTTAGAACCGGAGTTCGTTAAAAAAATACAAAGTTCGTTTAAAACGTGTAATGTTACTAAAAAAATGTAATGTTTAAATGATAATGATACCACATGCATTAATGAAAAAAACTTTTAAATTTTTGTTTTAATATTTGCATGAAAATGGAAACATTTTTAGTCTGTTTATTTCACAATGCAGATGGTTTACATCAACAGATTCAGGAAATTTTGTATTTATTGCGGATGCATATTTACGAAACAAATCTTTACTTAAAGCAGGAACTATCACGGCTTATATATCCAAATAGGCAACTTTCTTTTGTGTTACTTATGCCCCTTTCCCTTCTAAGAAACTGGGATGACATTGAATATTTAACGGACGTTGTAGATGATAAGCAGACTCTACATTACGCGGCAAATTTGCTGACAAACTACGTTCTACATCTATCCATGTTTCAAAAGCTGACAAAACCATACTTCCTTTTAGCGGTCAAGCGGGTCAGCGAAAAACTCAACAAAAAGCAGCGACATTCATTTTACGAGGTATTGGTAACCTCCGAAACCTTGAATAATTATGAAAACCTATCTAAAAACATTTTAAATACGTTGATGTTTGCCGTGCGCTACGTATTTAAACCTACGCCGAACTATTCAGAAATTCTCGCAGAGTTGGAAAAAAAAAATAAAATTCACCATATTATTTTTAATATGGTAATTACGGATTTTGCGCAAATCCGTGAACAACAAATGGATAAACATCTGTGTGAAACAAATAATGAGCTTCGTCAGGAATGTAAAGAAACTATTTTTGATTTAAAGGTGGTAGGAAATGTTTAGCCAATAAACTCATGCCCGCATTTTTTACAGGTACAAAATATCGTGGATGGCTCATCGAGGGCGCGTGTTTGTACTTCTCTGTAGGTACACATACGCTGCTTGCAGTTGGGACACTTATAAAGTTGTGACGTCTTTTCGGCGACCTTTTGCTGCGAACGTAGAGTAATTTCTGTCTTCTCCTTTAAGGCGGCAGAGGGGCAAAGCTCGGCGAACGTCATGCTACCAATTGCCTCCGGTTTTAGCTCGCCAGAAATTAGCTTATTAAGGGCATCGTTATCCTGTTGTTGGTGACTTTTTTTTTCGCAGTTAATAATATGATTGATCGTCCCACAACGGGTTGAATATTCTTCTAAAAAGGTTTTTTCTTGTTGCTGGTACGTATAATGATAACACGAGGCCTCGATTTTTTGCGCGTATTCGGTGCATAAATCAGTATGTTCCTTAAAAAACATATGTTTTTGAAGCGTTCTAAAAAACATCATTTGGATGATATCACGCATTTCCAAAATAATATAGGGTTCTAGTCTTTTGGAATCTTTCATAACTAGATCGGTGGTAATATTCTTAGTCATACAATTTATTAAAAATGGTTTAATATATTGTAAATATTTTTTAGGCGTGTCAGCCTGTAAAAAACATTCTTGTTCAATCTTATTTGTAAGGATAGTATTTTGCAAATACTTATTTAGCAAAAATACGATAGAATCGCGGGCTATATGCATTTTCATATAATTTTTTTTTTAAAATTTAATACAAAAAAAAGAAGTATAGACTCTTCTTCTAGTCCGGTTAGTTCGTTGGTTGCCTCAACATGGAGACTCAGAAGTTGATTTCCATGGTTAAGGAAGCCTTAGAAAAATATCAATACCCTCTTACTGCTAAAAATATTAAAGTAGTGATACAAAAAGAGCACAATGTCGTCTTACCTACAGGATCTATAAATAGCATACTGTACAGTAACTCAGAACTTTTTGAGAAGATTGATAAGACAAATACCATTTATCCCCCGCTTTGGATACGGAAAAACTAATTGTAACCAGTAGTACATTTAAGGATAGTTTAAGCAGTAAATGTAGAATAACACAGTTAAGCAATAAATAACAAGTATATAGGAATATATAGGAATATATAGGAATATATAGAAATATATAGAAATAGCTAAGCTTAATACTAATTCAGCTTTTTTTTTAACTAAAACCTGAATAGATGCGAAGTAGCGGACATATACATACTAAAATAAGCCATACATTTACTTTCTTCTTGAACATGAAACCTTTTTTTCTTCTGTTGTTGGTATATAAACAATAGGACTGTTTGCTGAGGTTGTATGATCTTCTACAACTGCTGTCTCAGGATGACGATGTTTTTTTAAACTAAAAGTGTAGGATGGAATGAGTGGAATATAGTTATGGCTCGACTTATCCTGTTTCGTACAGGAATATTTTTTACAAATAGAACGCAACAAGCATATGAATAAAAACAGAAATGATATACAGGAGCATAAAATAGATATGAACACTAAGGGGTAGCAGCTTTTATAACGTTCCGTATTTTTCTTAGCTATCAATTGATTTACCGTAATATTTATCTCGGGAAACTTTGTTCTACAATATTTTGTTTGGTATTCCAGAAACTCATGTCCTGGCTTATTCCCGCAGCTTAAAAAATGATACAAAAATGTGTTATTGTTACTAAAATTAATTCTTCTTAAGAAAAACTGCGGAAGACGCTTTAGGTACGTCTGTTCCTGTTTTAGTAGGAAGTAGTATAAGGGACAATTTCTTTTTCCACACATTAGATTATTGTAATATAGGTAGGTTGGGGTGTTGGAGCGAATAAGTTTTCTGAGTATGTTATAATCTATGACTTGTAAATCGTTATACCTTAGGTCCAAAAACTTGAGTTCTTTACCAAAGCCACCTGCAATTTCAGAAATATTTTTCATCCCGCAGCGGATAATACGGATGTCCTGAAACGTCTTTAAAATACTTGTATTGTAGTGAATACTTATGTTATTTTTTTGTAAATAATCTATGTCATGACAAGTGCATGAAATGCCAGCAGCATTGCTTGGTATAGTATTATATGCAGGAAGAACTATACTACTATTGAGAATAGTCACATTGTACTTATACCATGTATTATTTTCTGATATAAAGTATTTGCAGGTGACCTGTGGTTTAATCCTACCTGTTAAGCCACTTCCTAAAAAAACAAAAAATATGAAAACCCTTAGCATCCTGTATATACTATTAAAAATTTATAAAATTTTCTGTTTAAATTTCATTTAGACAAAAAAAATAATATATATACATCAGCAAGAAATTATATACAGATTATATAATTTTCTGATTTTTTTTTGCCACAATAAGCATCATTATATGCATTAAAATCTCAATACTAAACACTAAAATCTAAATTCTAAGCATTAAATTCTAAGCATTAAATTCTATGCACTAAACTGTAAGCACTAAAATCTAAGTAACTAAAATCAACACTAAATGTATGCAACCTAAAATGTAAAGCATTACTCATCATCCTCCTCTTCTTCATCCTCATCATCATAGGTTAAGATATATGTGTCATCCTCCATTTCTTCACATTCATCTTCATAAGCATCACTGGGTATTGGTGGAACATTGGATGCAGCATTTTTAAAATATTCTATGTCTTCTGGTGAACACTCATCTAATGATTTTTTGACAGTCCTTTTAACTTCCATGGGATATGATTCCAAATCCTCTTTATATAAGAGTTTACGGTAGCTTTTAGCTGCATCCACATTTGCTGGAGAATCTGGATTTGGCTCATTGAGCAGTGAAATTACACTAAGAAGAATGGTATCAATCTTTTGAGCCGGAGACCAAGTCATTCCCTGTTCTTCAGCATTGTCTCCGTGTAAGATAGAGATACATAGTTTTCCATCAGAGTAAATATTAGGATGCCACATTTCAGAGGTGAATGTTAATCTGGGTGGTGCATATGGGTATTCTGGAGGAAAGGCGATTTTTGCCTTGAATAAGCCTCCCTCATAAAAAGTGTCAGGTGGGCCCCTTAAGATCACATCCCATTCAGTCATATCCTTCTCATTCACCGAAATTTTGAAATTCTCAGAGGGATTCTCTATCAGGTGTCTGTACTCTGCTATTAAAAACCTGGAAACCATGGTTATTTAATATTAATTAAATTCCCTGGTTTATTCCTCCTTAAAAGTAGATGAACCTCTTTTGTTTTTTATTGGGTTCATTTTTACTAAATTTATGAACTGGAAAAAACTTTAACGGCATAATTATCAAATGCGAAGGGGGATCCGTATAAAATCCTAGCTTGCCGGTAATGGCTATTAAGTTAAATTTGGTACCAGTAACACTAATATTTAAAAAGCCCTGATCATTAACTTTCCACATTAAAAGATTATTATATTCGAATGTTTGTCCAATATGGACAACTTTGTCACCAGATGTTACATTTGATTTGGTTGTTAGTGGCTGAAGCTTGGCACAATCAAAAATAAGCCCATTAACACTAAGATATAGAGGAGTGGGTTGATCTATTTTCTCATAGTTTAATATTCCATCTTTCCACGTAATAGCTTGATAATTATCCGCAGCAATGAGTTGAAATTTTATAAATAGTACAGGGGTTTTAGTTGTCGTTATACATTTAAAGGGTGTTTTATAAAAATAAAAAATAATAATTGTTAAAAGTATGATAATAATCGCCAAAATAATTTCATACATTTTTTATAAGAATTATACATAGTATGGTATTTAAAATATTAGCTAAATTTAAAAAAACTTCATGATTTTTAAAACAGGGAAAAAGGGGATTAGGTTGAATAAAAAAGGTAAGCACTTGTCTATATATTTTTTTTACAATGTTGCCTTGAGTCGCATTTTTAACTGGCTGGGGAGTATCAGAGTGGAATATCACTGTAGTAGGTCTATAAGGTCTTGTTAAAATATGATCGGTCATTGTTTTCGTACTAGTGTCATTTAGGGTCGACCTGATAGCTCGATATAAAGTTATAGGGGATAACCTATCAAATACAGTCTTATCTGTGCTGAAATGTATATCGTCTTCTTTATCACTAATAATATTAGGAATGGCTGTCATTAAATAATTACTACTTGTTGTTGTGGGTGAAATAGTTGTACTGGTATTATTGGAAATGGCTGTCATTAAATAATTACTACTTGTTGTTGTGGGTGAAATAGTTGTACTAGTATTATTAGAAATGGCTGTCGTTAAATAATTACTACCTATTACAAGTAAACTAATGCTAACTACATTTTTAACCTCAATAAACCTAAAAAGCCATACTAAATACCTAAACAACATCCTGTTATAATATGAGCAGAAAAAAAAAATAAGTATAATTAGGGAATTATTCTTATTCGCTTACTATTAAGAATAATTCAGAATCTTATTTAGTTAGAAACTATCATAAAGTGAATAGGACTCATCGTCGGATGAAGATTCCGTTTCAGAGATAGTTTCTTTTTCTTCCTCAGAATAATCTGTTCCTACAATAGAATCGGTGTCATCCTCAGAAAGAGAAGTATTTAAATATGGACTATCTATAGCAATATCCTCTTCTATCTCGCAATCCTCCTCCTCCATTTCCATAGTGTGTAGGAGAATATTTTTATCATCATGCTCACTTCTTTTTTTGTTGAAAGATGAACCGTCCTCAATACGGTTCATGTTAAGTTCCTTCATCTTATGTATAATTTCCGTAATCCGTGATGTTTTTGACATGTAAGATGGTTTTAAGGTTATATCCACAATAACAGGAGAATCTCTATCATTTTCATTTGATAAACTTTGATCTTTGATTTCTTCGTCTAAAATTCTTGTCTTTTTTTGGGTACTAGATGAAATAGAGGAATTCATATTCTGAAACGATATATCAAGGGGAGCTGGACGCTTTTTTCCAATTAAACCGTTTTTCGAGATACTATGATTAGATGAATGATCTTTAGCCAAGCTGTCCTTGGATATACTATAGTTAGATATTTTACCTTTAAATAATATTCTTCTATACAAGTTATTCTTAGGTAAAGAATTAGTATGGATTCCTATATTTTTATCTGAAGGAGTGTCCATATCGGAGAACGTCCTCTTACGAATATTTTGACCACGAGCCATTTCATCCACTATAGGCAGTATTTTGGCTGGCTATGGTTCTTTGTTGTGACAATTCTATGAGATTTGATTGCAAATCAATTTTTAGTTTTAAATATATTGGTACCTAGGACAAAGAAAGTATATATAGCCAATAATTATTCCACTAAATTGATTTCCAGACTGATGGGTATGGAGCCATGTTGTCTCTGCAGACGATCGCAAAAATGGCCGTAGCAACAAACACCTACTCCAAGTATCACTATCCAATACTGAAGGTCTTTGGGCTGTGGTGGAAAAACAATACGCTAAATGGCCCTATTAAAATATGTAACCATTGCAACAACATAATGGTAGGAGAATATCCTATGTGTTACAATCATGGAATGAGTCTGGATATAGCTTTGATTCGGGCAGTAAAGGAGCGTAATATATCCTTAGTCCAGCTTTTCACCGAATGGGGGGGAAATATTGACTATGGGGCACTTTGTGCTAACACTCCATCTATGCAAAGATTATGTAAAAGTTTGGGAGCCAAACCACCAAAGGGCCGAATGTATATGGATGCTCTTATACATCTTTCAGATACCTTGAATGATAATGATCTGATTAGGGGGTATGAGATTTTTGATGATAATAGCGTGTTGGATTGTGTCAATCTCATACGACTCAAAATAATGCTTACCTTGAAGGCCCGTATACCTCTCATGGAACAACTAGACCAAATTGCCTTAAAACAACTTCTGCAGCGATACTGGTATGCCATGGCTGTACAACACAACTTAACAATCGCTATCCACTATTTTGATAATCATATTCCTAATATAAAGCCATTTAGTCTGCGCTGTGCTTTGTATTTTAATGATCCCTTTAAAATCCATGATGCTTGCAGAACTGTAAATATGGATCCTAATGAGATGATGAACATTGCTTGTCAACAGGATTTAAACTTTCAAAGCATTTACTATTGTTATCTTTTAGGGGCTGATATTAATCAGGCTATGCTAATGTCTTTAAAGTATGGTCATCTTTCTAATATGTGGTTTTGCATAGATTTGGGGGCGGATGCCTTTAAAGAGGCAGGGGCGCTTGCTGAGAAAAAAAATAAAAGAGTGTTACAACACATATTAGGTCTTAATATCTTTAAGCGAGAGTTGATTCCCCCCTGTAAAGATCCTGATCCTTATCAAATCCAAATTCTGTTAAAAAACTACATTCTAAAAAATGTCTCAACTGTTTTTACATATTATTGCCAGTAGCCATTGTTTATATCAGAAAATAACCCATTTGTTTATCTTTTTTTGTGGGGCAACCATTAAGACCCGACGCAAAAAAAGATTAATCTTTTATCAGATACCTAAAACGTTCTATAAGGGAGTCTATGAGATGGATCATATTTTGATGGTCATAGTAAGAAGCAAGCTTTTTGGCGAAAACAACGGAGTTAAAGAATTTAACCCGCTCATGTTTGGATAGGACTTTTAACAGCGAGCCAAAACAGTATTTAAAAATTTGGCAATAGTTTTTTTGGGATGCAATAAACAAACACTTGATCAGTGCCCGCTTCACTTTCTGATCAGACATGTTTGCCGCATAACAGGCCTTTTTAAACTTAGTAATATAATTATGTTCCGCAAGCACCATTAACAAGGGAACGATGGGAAGCTGCTTTTCTTGGTGAAATTTACGTAAATATTCGATGGCCACCGCTTGGACGACTGTGTAATTTACTAAGTTAGAAATGATAGCTTTCATGGTTGTAAAAATATACATAGGATTTTCTTTTTCTGTATACAGTTTGAAAAGCTTATGATTACGTGAAATGATGGCCATTTTTAATACAAGATGGTATAGTGTATCTTTAGGTAAAAATGCCTTGCAAGCCGCGATGATGTCGATGTTGTCTCCATGAACAGCGATAGAAACTAATGTTTCCAATCTAAATGTTTTTATCTGCATTAATAGAAGAATGCAGTCAATGTTATTATACTTAATAATACTGTAATACACCGAATCAATGACCGTCATCTGAGAATCAAGCTGACTTATTAGTAAATTTAACGTTTTTTTGGAGGCATGACCTTTGATCGCGGCACTAAGTGCACACAGTATAGCAAAATTGTTAAATACATTTTGATTTAGGAGAAGGAGTAATATTTTCCTTCGGTTATAGTACGCAGCATCTGTGATGATTATTGGCCGATAAATGTTAAAATGTGTTAACAGCTTTTTAAAAAAACGGAAGTAATTTTTTTGGATCGCTGTTTGCATCATCGAAATAATGAGATAATCAGGGTATATAATGGGTAGGTCACATGCTACCTCTAACAAAGAATAGTCGCCCAATCTAAAGGCTGTGTTGAAAAGCGTACTATCATCATACGTATCGAGTACCCCTGCTGTTACAAACCAAGCGATAAGATGAATGTGCCGTTCCTTGCAAGCTATCGCAAATAGGGAGTTTCCTATGGAATGTCGAATAATGTACTCCCTATTTTTTTCCAAAATGTTTGGAAAATTGTATAGCGTTGCGGCATACAGTAGACACTCCATTCTGGCGTTATAATTTTTACTTTTACATATGAATAGGTGGAAGAACTCGAATAATTCTTGAGAACTTGTTAAATGCATAATATGGTGATATTTTGGTGTCGTTAAATGGTATGAGAAAATGCATTCTAATACATCTTTTCGGTTATGCTTTAGCGCCTGAGCTAAGGCATATTCAGGCTCGACCCATAGGACTAGTGTTTCTATAATTGAGATATTCGCCTGCTTTGCCAGGGCATACTTTAAGACGCTCCGGTTAGAAAAAATGTTGTTATGAAGATGGATAACCGTATCCATTTTTACGATGGGACCATTCCAGTATAGTCCTAAATGCTGTAGCAGATCTTTTGTTAGTTGTGAAGCGTTCTCGGGTGTCATATAAATATGTTGCAGGGCTTTTTTCTGTAAGGAGAACATTTCGTCGTAATCGTACAAAAAAAAATTAAAATTTGGGCATGGATGATTCAAACATAACAAAATCAAGATTTTATAACAGTTTGCATTAACCTATACATATATGCAAGTAAATGAGATATTATCTATCATAACGAATCAAGGGATATTTGTATATATCAGGAGTTTCTGAAATAAAGATATGAAGATTATCATAGTAGTATCCATCAATCACAATGCAACTTCCTTTAAGGCATAATTTAGTAAACTCAGCACTCCCATCTTCTGGATGCTTTACAACTAACATTAAAAACTCCTCAGTCATATTATCTGTAATAAAATAAGATCCTCCTGGAGCCATTTGTAGCATGTCTCTTATTCCTACAAAATCTTTTTTGGGATGGTAAAAACTCAGCAGTTTCAAACTCTTTTTTAGTTTTTTTTCCTGGTATTTAAGCCATTTGTTATAAAACAGTTTTCTTATGAAAATGCATTTGAAAATATTGGGAATGTTTAACCATGCTTCTTCCGAGCACATCTCCAGATACTTACTTTCTTTGTTTCCCATGTCTAATTTATTGCTCACTAAGTTAGTAATGAATCTATTTTAATAATCTACTTTACTAATCTATCTTAATAACCTATCTTATAATCTATCTTAATAACCTAATTATAACCTATTTATAATTGGCTAATGCTGCCGGCATTTCATGCCTATCTAAACAACTCCTACTAAGCAATCTACTATTACATATATAGATTCACTTTTTATATTTGTAAATCATGAGAATTATAAAATCATTACTCATTTTTATTGTAAATTAGTGGGTATTTGTAAAAATCTTCAAACGTTTTAAGATAGTTTTCTAGAGAGAAGTAATCTTTGCCATCAATATATAATGCTTTTCCTTTAAACTCCAGTTTTGCTATGTTTAGTGAGCCGTTTCTAGATCTTTTTGGGCAATAAATAGATTTTCATTGGTTGCATCGTCCGTAAGCAGAAAGGTACCACTAGGCACGTTAAAAAACATACGTTCTATTTCATGGTCGGATTTTTGAGAATAGAAAAAATCTAATTTTTTAATCCGCGTTAACTCTTTTTTATCAATCTTTCCAGACTGTTTTATATATACTTTATTGCAAATCTTACAATCCTCTATGGCTTCATTATACTTATTTTGCTTATCCTCTATTGACATGTCCGTATTTGATAGGTAACTTCCGTTAAGGCGGTTCCCCATGGTTTTAGATAGATTTTTAATTCAGTTGTATACTTTTATTATGAGGCTAAAATATAGAAGTTTGATCCTAAAAAAATAAAAAGATTTTGTACATTTATTTATGGTTTATAGCGGTATAGAGGCCGATAAAAGGTATCCGGGTAGTCTCCTATGATATCGTCAATTTTGGTATAATAACAGTTGTTATGGTAGTATTGTCCAAACCGAGTATGTATGCGCCGGTGAAGCGTCCGCCCGCTAATGGTACAGTTCCAGGTTAAGACAATCATATCACACCCAAAAAGAGAGGAAACAGCATAGGTGCCCAAAGGTTCATTATATAACATACGCCGCATATATTTTAGTTTTTTTTCTCCATGGTAATAATCACAGGTTTTCATGTCCTGCTTAATAGGATGATTCCCCATGTATGATAATATATAATAAATTTAGTTTTTAGCTTTTTCAAAAAATTGGGCGCTCGAAACTAAATTTTCCTTATCACAGCGTTTGGAGAAAGCGTATTTAAAGATATATCTTCTTCTAACAAGACTGCAAAAAAAATCTTACCCCTTATTTTTATAATGTTCATCATAGCGTTTGAAGATATCAGAAGGTGCCAGGTTTTATAAAAATATCCTTTAGGATTTATAACGATACAAGGGTCTATAAAATATATGCGGGTATAATCTTATAAAATCATCGATTTTTTCATAATATTCTCCGTTTATACAATAAAGATCATAACAGATATTGATGCGTAGATGCATTATTCGCGTGTTCGTTGGGCAGCTAAAGGATATCACAACGTAGTTTTTTTTAAGAAAAGACGAAACTACATAAGTCCCTAAGGGTTCATTGAATAGTAAACGCCATATTTGTTTTAAATTTTGTTGTTCACCATAGTAGTATTCGCACTTTTTCAAGTCTTTTTTAATAAGCCTATTCCCCATGTATGCTTATAAATAAAAATTTAGAAATGTGCTATATTATTTGTTGATGAATCATGAACACGTCTTATATGTTGATATGTTACTTTAAAAACATTTGTATTTTCAACAGACGCGTTCTATTCTTATTAAGAATGATGCCGTCTTTATTTTAAACCTTGGTTTAAAATTTAAAGAAGTATTTATAAACTATAATCATGGGAACTTTTTCAGTAACTGCCTCTGCAAAAAGTGACGATGCTGTTTGTAAGTATTTAGAAGAACCAATAGATGAAAATTACAGAAACATATTAAGAAATGAGCATGTTAAAAAAAATTTAAATGAGGCTCTGAATCGACATATTACTACCTATAATCCAGTAGTTGATTGGTGTAATAACTATTCAACATTTTCATCTCAGGATTTCGATGAATATAAAATTTATATACATAGCGATCTTATGGATGGACGACCTCGTCCAAAAAAAAACATGGTGTGTCATCATGTAATGTTTGTTAGTTTTATATAAACGCAAAAATATTCTTCTAGGAGATGTTGATATACTACCTATTGAATTCAATATATTAAAGTACATTTCTGGCTATTCCCATTACGGTATTATTATTACTATTTTTAAGAGCTAGATGTGGATTTAAGTAATAATAACATTCTCCCGTTCCTCCTAGAGACACCTCATCAAATTCCCATCCTATGCAACCTTTATGTTGTAAACATAATGATTGACAGCATTCATCTTCTTTTGACCAAGTCGTCCAAATCCTACCAAGATCTATACGTGTTTTTCCAAATGGAGATTGAAGATCAGCAGTAGTGGCATTAAACCTATAAAAACCAGGTGCATAATCACATGAACGGATCGTAGGATCTAATTTAATATCTTTTATATCTTGTTTTACTGCTTCTAGACAACTTTTATCAGTACATGTTCCACGTACACAGTGGTGTCCTTTATCCTTACAATCCGTATCTGTCTTACATTTTTTTTTCGGCGGTTTATGTTTCAGATGGTAAAAACCCAGTATTAAAATAATCACAAGAATAATTCCTATAAGTACTTGAACAACAGGATAAAACATTTTAATATTAAATATATTTTTTAATTAAATGAATAGATTTAATCCAAGTAGTATTAAAATTTTTTAGAAATAGTGTTCTACAAATAATGAAATGAATGGTCCAAAAAAAATAAGGTGTACAATAATGTAATATATTGTTAGGCTAAGTAAATTTAATATTTTAAAGTATTTGGAAAAATATTTTTTAACATATGATGTCTAGGAATATTTTTTAGACATTTAAAACCATATAGTTACTTTATTTATTACACTGAACTTGAAAAGACTTATTACCTAAAATATTAATAGATGAAGTAATATTGTGTAATTGAGTCCATAACATGGGTGGGAAACAAAAATCTCGTAATATGAAAAATAAACATCCTAAAAAGAGTGCAATTGTTATAAGTTTATGTAACTTTATTTTAAAGTAAGAATATAAAAATATGAGTACAAGAGGAATAGGGGCCATTACTAACATTGGCTCCAACATCCTGTTGTCTACAAAAAAAAATATTTTTTTTAGCAAAAAAAAATCCATGGAAGGATATTAATACACATAATTATTTGACATCACATTAGTGTACTTACCAAATAGTAATATACAACCATCCTAATATTCACCTTTATGAAATGATCCCAACCTATACGGTAAAATAGTATAGGTTTTAATAAAGAAAAAAGATATTCTGTGGTTTTTATTTTTGTATAGTGTGTGAATACAAAATAAAATCCCAAATTTTAACCTTTTCTTTTTTTTTCTATACAGGATGTTAGAAATAGTATTGGCAACGCTGCTAGGCGACCTGCAGCGGCTCCGGGTTCTTACCCCTCAGCAGCGGGCAGTTGCCTTCTTTCGAGCCAATACTAAGGAGCTAGAGGACTTCTTATGCTCAGATGGGCAGTCTGAGGAGGTACTGTCTGGCCCCCTTCTTAACCGTCTACTAGAACCCTCAGGCCCTCTTGATATTTTAACCGGATATCACCTATTTCGTCAGAATCCCAAGGCAGGTCAGTTGCGCGGCCTTGAGGTCAAGATGCTTGAACGGTTATACGATGCTAATATTTACAATATACTGTCTCGGCTGCGGCCTGAAAAAGTTCGCAACAAGGCTATTGAGCTATACTGGGTTTTCCGAGCTATCCATATTTGTCATGCTCCTTTAGTTTTAGATATTGTACGATATGAGGAACCGGACTTTGCTGAACTGGCCTTTATTTGTGCTGCTTACTTTGGTGAACCTCAGGTAATGTATTTGCTCTACAAATATATGCCTCTGACCCGCGCAGTTCTTACGGATGCCATCCGGATAAGTCTTGAGAGCAACAACCAGGTAGGGATTTGCTATGCTTACTTGATGGGAGGCAGCCTCAAGGGACTAGTCTCCGCCCCACTGCGTAAACGTCTGCGCGCCAAACTACGCTCGCAGCGCAAAAAGAAGGACGTTCTTTCACCCCACGACTTCTTACTGCTGCTCCAGTAGCTTTTTTTGCCGCAGGAGCACCGCGGATAGGAGCTCCTCCACGCTCGCGATCCGGCGCTGGAAGCGGAACCGATCGACCGCCACCTGCTCCCAGGGACCCTTGCGCTCGATGTCGTCGGCTTCCCACACCTCGACGGCTGTGGCAAAATGGACATGCTTCGCGTCGTTCGTCCGTTTTTTGCGCCGCCTCCCCATTATTCTTCCTGTAAGATTAGTGTTTAATACCTATAATAACATAATTTTAAGATTTAATATACCAAAACTTAAACTATTTTTGTATAGTAACTATTAGCATGTCTACACATGATTGTTCTCTAAAAGAGAAACCGGTTGATATGAACGATATATCTGAGAAATCAGTTGTCGTGGATAATGCACCCGAGAAACCAGCTGGAGCGAATCATATACCTGAGAAGTCGGCCCGCGAAATGACATCATCAGAATGGATTGCTGAATATTGGAAAGGTATAAAACGTGGAAATGACGTGCCATGTTGTTGTCCAAGAAAAATGACCAGTGCAGACAAAAAGTTTTCAGTATTTGGTAAGGGATCCCTAATGCGCTCCATCCAGAAGAATAATTAAAAAAAATATTTTTTTTAGCAAGTTTTTAAACTATTTAAATAAATGTGGTAAAAAAATTCACATAATAATTAAAGTGAACGTGTTAGAATTAATATTTTTTTATAATCGGATATAATATCCATTAAATCAATAAATGATAGTGTTGCTACCACACTAAACAATAACAAACAGAAACGCACGATACCTTTCCTCATGATTTATAATAGCGTGTTATCTAAAGATTTTTTTGAAAAAAATATTAAATTTTAGTTGATTATTTTTTTCAGTTACAACATTGCTTTAGAAAAAATACCTAATTACTACATAGCAAATAAAGCGAGCGCATTGTTACAAACAACATTTTTTTGCGCCTGGATACTCCTATATATGAGAACTATAATACGGTATATTAATCCTATTACCAACATTGTCAATAATAGTATGTAGGCAATGACATACTTTAAATACCAAATATCCATGGTTATTTCTAAAAATCTTGAAAAAACGTTAAATTTTAGATCGGTCACCTACGACAGTAATACTAATTTTAATAATTGATGACTGAAATCATAATATAATGCCGTGCGAAAAATAATTATTTTTCGGTTAAAGATACCATTACATAAAAAATATGCCATCTACTCTACAAGTGCTTGCTAAAAAGGTATTGGCCTTAGGGGAGCATAAAGAAAATGAACATATATCTAGAGAATATTATTATCATATATTAAAGTGTTGCGGTTTATGGTGGCATGAAGCTCCGATTATACTTTGTTATGATGGGAGTGAGCAAATGATGATAAAGACTCCAATCTTTGAAGAAGGCATATTACTTAATACTGCATTAATGAAAGCTGTACAGGAGAATAATTATGAATTAATAAAGTTGTTTACTGAATGGGGAGCAAACATCAATTATGGATTAATTTCCATTAATACCGAGCATGCCCGGGATCTATGTCGAAAATTAGGAGCTAAAGAAATGCTTGAAGGAAATGAATTTATACAAATTATATTCAAAACATTAGATGATACCACCAGTAGTAATATAATTTTATGTCATGAATTATTCACCAACAATCCTCTTTTAGAGAATGTAAATATGGGGGAAATGAGGATGATAATTTATTGGAGGATGAAAAATTTAACGAACCTATTATTAAATAATGACTCTATTAGTGAAATATTAACTAAATTCTGGTATGGTATAGCAGTAAAATATAATCTTAAGGATGCGATCCAATATTTTTACCAGAGATTCATGGACTTCAACGAGTGGCGAGTAACATGTGCTCTTTCTTTTAATAATGTGAATGATCTTCATAAGATGTATATAACAGAGAAGGTTCATATGAATAATGACGAAATGATGAATCTAGCCTGCAGCATTCAAGACAGAAATTTATCAACCATTTACTATTGTTTTCTATTGGGGGGCTAACATCAATCAAGCAATGTTAACCTCAGTATTAAATTATAATATTTTTAACTTATTCTTTTGTATAGACTTAGGGGCTGATGCCTTTGAAGAGGGTAAGACCCTGGCGAAACAAAAGGGGTATAATGAAATAGTGGAAATCTTATCATTAGATATCATTTATAGTCCAAATACTGACTTCTCATCAAAAATAGAACCTGAACATATTAGTTCTTTGTTAAAAAACTTTTATCCAAAAAATCTGTTCGCTTTTGATCGTTGCAACCCCGGTTTATATTATTCTTAGAGGACCGCTACAAAAATTATTTTTTTTTCTTGATCAAAGCTCCAAAATAATTATTAGATTAAAGTCGCCTATAGCAGCAGCCCACTCCAAAAAAAGTATTTTATAGTACAAAAAACACGAAAAATAGTTTGCGGCCGGCGGCAAACTATTTGTTGTTGTCTAAAACTTAATGTTTTTTTAATATTTTTAAATGCAACCATGGATTGTTGGACTATCAGGGAGAAGAACTATAGCTACATCATATTGTCAATACTGGTAATACTATTAATATGGTATCTTATACTTAACTATTGTCGATCGAAAAAAAATGCAGTTACAAACAACATGCCGCCACCATACACGGTGTCAAGTAGCTGTTCTCAATAATAGGGTTGATTGACGCTCTTCGTAATAATATGTTGATTGACGCATCATAAAATGCTGTGGTTGATTAATATGTTGATTGTCGCCTACTTTATTATATAAGTAATGATTTTTGTATAAAATACGGGTTTGTGAGGGCTTTATTTTTTCTTATTAGAACAAAGCATGCAATTTAAGGCCTACAGCAAGAGTAATTTAACACCTACAACAGTAATTTTAAGGTCAGTAATAATGTTTAATTAAGGCCTGACCACTAAAACTTAAACGATTTTGTAAAAAAAAATGTCTACTCCACTTTCTCTACAGACTCTTGTTAAAAAAGTGCTGGCCACACAGCACATATCTAAAGAACACTACTTTATTTTGAAATATTGTGGTTTATGGTGGCATGAAGCGCCGATTACGATTTGCATTGATGAGGATAGCCAAATATTGATAAAATCGGCAAGCTTCAAAGAAGGCTTATCTTTAGATATCGCATTAATGAAAGTCGTGCAAGAAAATAACCATGATTTAATAGAGTTGTTTACCAAGTGGGGTGCAGATATCAACTCTAGCTTAGTTACTGTTAATACGGAGTATACCCGGAACCTTTGTCAGAAATTAGGCGCAAAGGAAGCTTTGAATGAAAGGGATATTTTACAAATATTTTATAAAACACGTCATCTTAAAACTAGCAGTAATATTATTTTATATAATGAATTGTTTTCTAATAATCTCCTTTTCCAAAATATAGAGAGATTGAGTTTAATAGTTTATAGGGGCTTGAAAAACTTATCAATCAACTTTATATTGGATGATATTTCATTTAGCGAAATGTTAACTAGATACTGGTATAGTATGGCGATATTATATAACCTTACTGAAGCCATCCAATATTTTTATCAACGATATAGGCATTTTAAAGATTGGCGGCTTATATGTGGGCTTTCTTTTAACAATTTGTCTGACCTTCATGAAGTATATAACTTAGAGAAGACGGATATAGACATTGATGAAATGATGAAGTTGACCTGTAGTACGTATGATGGTAATTATTCGACTATTTATTATTGTTTTATGTTGGGGGCTGACATCAATCGGGCAATGTTAACCTCGGTAATAAACTTTCATATTGGTAACTTGTTCCTTTGTATAGATTTAGGAGCTGATGCTTTCGAAGACAGCATGGAACTAGCAAAACAAAAGAATAATAATATATTAGTAGAAATATTATCATTTAAAAATTATTATAGTTCAAATACCTCTCTTTTATCAATAAAAACGACAGATCCGGAAAAAATTAATGCCTTATTAGATGAAGAAAAGTATGAGTCAAAAAATATGTTAATGTATGAAGAATTATCTCATTGATACAAAATTATTTTTTATAACAGAACTCTCTGATGGTGACAAATCTCCGATAGGAATATATGACGTAACATAATTATTTTTTTCGCCCAGAAAAAAATTATAAATGTTATTATTGCCAGCACTTTTATCAACTATACGTACAAAAAGGTGTTGACCAAAAAAATAATTTTTTTTCTTGATCAAAGTATGTAAACGCCCGCTTACAGCAAGGATCTTAAGTGAGAGCCATTAAATTTTATTGATAGCTGCTTGCCACCAGTAGAATACGGCCAAACCACCTAACAGGAAATACAAGGCGGCCCTTCGGCCAATAAGGTGGATAAAAATCACGCATAAGACGGTTGTAACATAGCACTTTAGTGCGAATATCAGGAATGCCAATAGCATGTAGATAAGGCACCAAACATCGCAGCTATACATGGCTAAAGATCAACCAGAAAAGGTTTAAATTTTAACGCCGGCCCAAAACTTAAACTTTTTTTGATATTTTTAAGTGCAGCCATGGATTGGTCCGGCCATAGGATGACCTATGCCTACGTGGCATTCTCATTGATGGCAATAGCAATAATATGGTATATTCTACTTATCTATTGCCGATCGAAAAAAAATGTTGTTACAAGCGGTAATACGCTCGCTTTAGCGCCAATATCGCATATGTGAAAAATGTTCGCCGAAAAAAACATTAAAATTTAGAACCGCCGCGGCATCTCAGGGGCGGCAACATTTTTTTTTATATGGATATTGTCACACACCACCTCATCTATGACGCAATATATTACTGCTAATATCAGGTTCCCCAATAGTATGTAGAGAAACCACACAAGATAGATATTCATGGCGATTTTTGACGAAAAAACATTAAGTTTTAGCTTCTTTGACGCCTGTGTACTAATAATGTTTAACGCCTGTAGTATAATAATTGATACCTACAGCAGTAATTGATACCTACGGCGATAATGTCTCTCTGGCCGCCCCAAAAAAAAGTATTTACGGTAGGGTTTATTACCGGCGGCGTAACACCAGTTATGGTCAATTTTGTCTGGCCCGCCGCCCAGCCGCAAAAAAAAAATCAATTACAACCGCAAAAAAAAATATTTCCGGCCGCGGCGTTTCAAAAAATAATCTTTGCGAAATAATTCCGCATCTTGTGAAATGAACGCCTACAGTAATAATTTTAATCTTTGACACCTACAGCAGTAGTAATAATTTTAATCTTTAACGCCTGCAGCAGTACTAATATTTTTAATCTTTAACGCCTACAGCAGTAGTAATAATTTTAATGTTTAACGCCTACAGCAGTAGT
