# Supplementary material for: African swine fever virus pM448R protein promotes STUB1-mediated ubiquitin-proteasome degradation of IRF1 to attenuate type III interferon induction
Source: J Virol. 2026 Jun 9;100(7):e00580-26. doi: 10.1128/jvi.00580-26 (PMC13386975; doi:10.1128/jvi.00580-26)
Supplement: Supplemental material — Fig. S1 and S2; Table S1. [file jvi.00580-26-s0002.docx]

**Supplementary data**


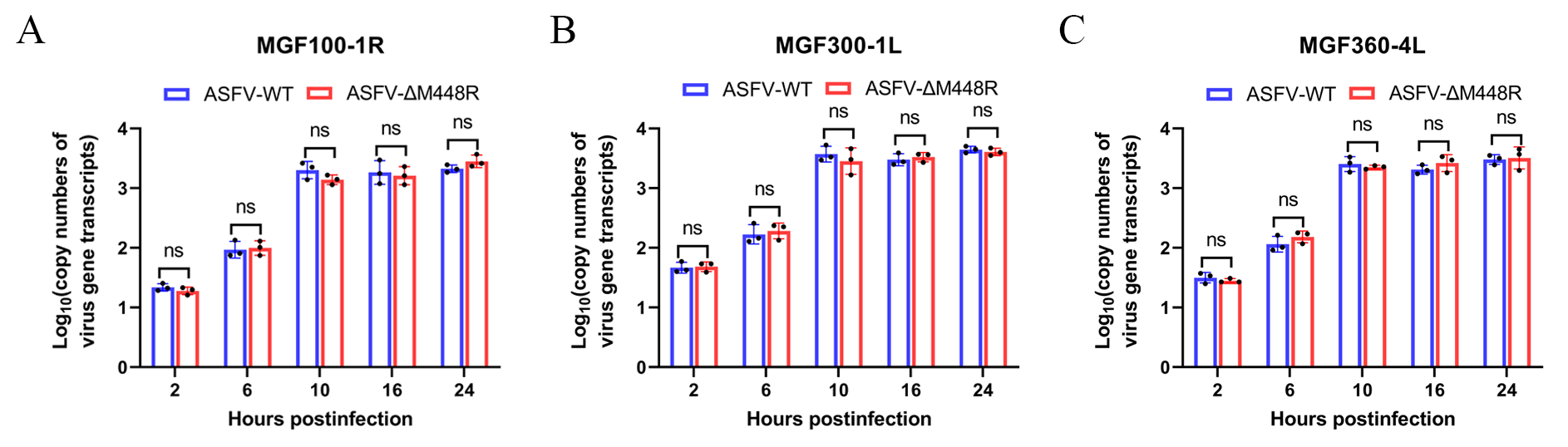


**Figure S1 Detection of transcriptional levels of MGF100-1R, MGF300-1L, and MGF360-4L in the M448R deletion virus and parental virus.**

(A-C)PAMs were infected with ASFV-JS-1 and ASFV-ΔM448R at a MOI of 1. Cellular RNA samples were collected at 2, 6, 10, 16, and 24 hours post-infection. The mRNA levels of ASFV MGF100-1R, MGF360-4L, and MGF300-1L were analyzed using qRT-PCR. Each experiment included three biological replicates.


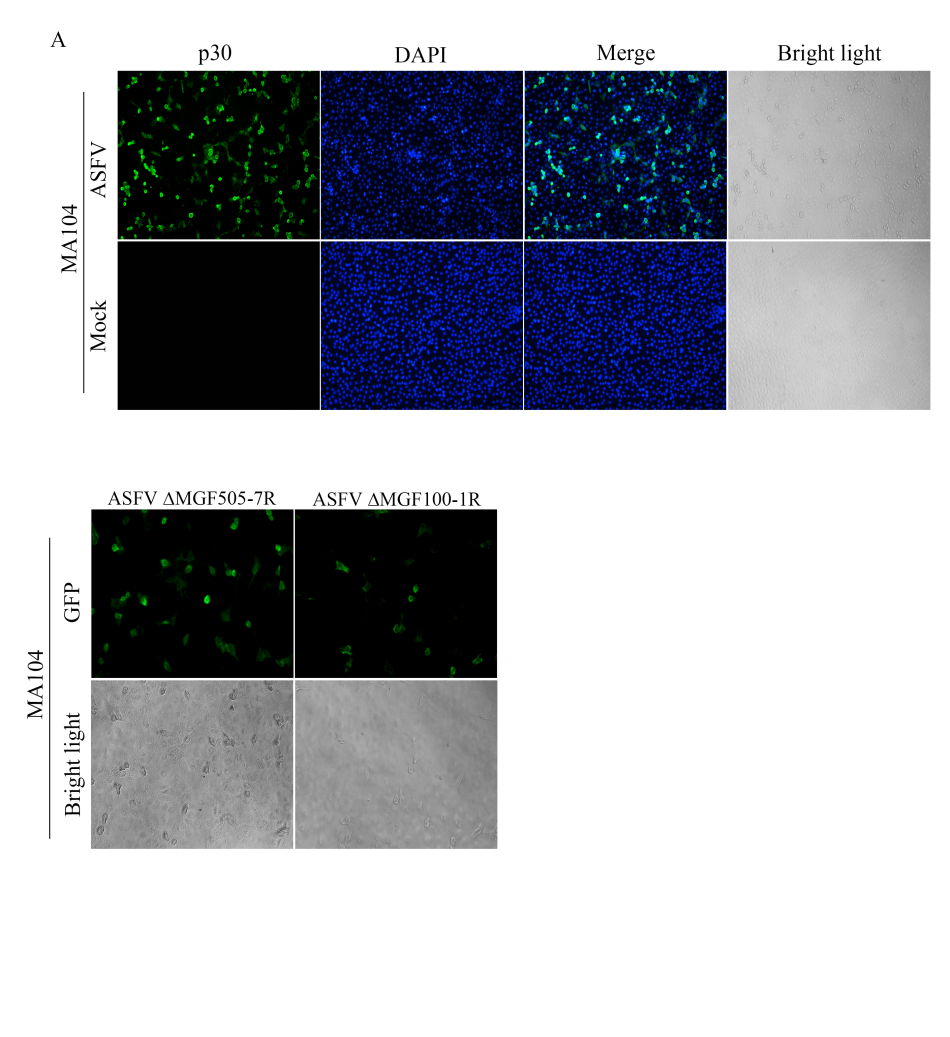


**Figure S2 p30 monoclonal antibody-specific detection**

**(A)** MA104 cells were infected with ASFV at an MOI of 1 for 24 hours, and then IFA was performed using a P30 monoclonal antibody.

**Table S1. The sequences of the primers used for PCR.**

| Primer | Sequence(5’-3’) | Description |
| --- | --- | --- |
| pIFN-λ1-F | GGCGACTGTGATGCTGGAC | Porcine IFN-λ1 gene |
| pIFN-λ1-R | GCAGCTCCAGTTCTTCAGTGAG |  |
| pIFN-λ3-F | CCACTTGGCCCAGTTCAAGTC | Porcine IFN-λ3 gene |
| pIFN-λ3-R | CACCTGCAGCTGCTTCAGG |  |
| pIFN-λ4-F | GCTATGGGACTGTGGGTCTT | Porcine IFN-λ4 gene |
| pIFN-λ4-R | AGGGAGCGGTAGTGAGAGAG |  |
| pGAPDH-F | ACATGGCCTCCAAGGAGTAAGA | Porcine GAPDH gene |
| pGAPDH-R | GATCGAGTTGGGGCTGTGACT |  |
